# Predictive performance of international COVID-19 mortality forecasting models

**DOI:** 10.1038/s41467-021-22457-w

**Published:** 2021-05-10

**Authors:** Joseph Friedman, Patrick Liu, Christopher E. Troeger, Austin Carter, Robert C. Reiner, Ryan M. Barber, James Collins, Stephen S. Lim, David M. Pigott, Theo Vos, Simon I. Hay, Christopher J. L. Murray, Emmanuela Gakidou

**Affiliations:** 1 Medical Informatics Home Area, University of California Los Angeles, Los Angeles, CA USA; 2 David Geffen School of Medicine, University of California Los Angeles, Los Angeles, CA USA; 3 Institute for Health Metrics and Evaluation, University of Washington, Seattle, WA USA

**Keywords:** Infectious diseases, Research data

## Abstract

Forecasts and alternative scenarios of COVID-19 mortality have been critical inputs for pandemic response efforts, and decision-makers need information about predictive performance. We screen *n* = 386 public COVID-19 forecasting models, identifying *n* = 7 that are global in scope and provide public, date-versioned forecasts. We examine their predictive performance for mortality by weeks of extrapolation, world region, and estimation month. We additionally assess prediction of the timing of peak daily mortality. Globally, models released in October show a median absolute percent error (MAPE) of 7 to 13% at six weeks, reflecting surprisingly good performance despite the complexities of modelling human behavioural responses and government interventions. Median absolute error for peak timing increased from 8 days at one week of forecasting to 29 days at eight weeks and is similar for first and subsequent peaks. The framework and public codebase (https://github.com/pyliu47/covidcompare) can be used to compare predictions and evaluate predictive performance going forward.

## Abstract (web summary)

Forecasts of COVID-19 mortality have been critical inputs into a range of policies, and decision-makers need information about their predictive performance. Here, the authors gather a panel of global epidemiological models and assess their predictive performance across time and space.

## Introduction

Forecasts and alternative scenarios of COVID-19 have been critical inputs into a range of important decisions by healthcare providers, local and national government agencies and international organisations and actors^1–4^. The total number of deaths in each county, and the direction of each county’s trajectory, are indicators of immense public interest, frequently discussed by the public and heads of state. Hospitals need to prepare for potential surges in the demand for hospital beds, ICU beds and ventilators^1^. National critical response agencies such as the US Federal Emergency Management Agency have scarce resources, including ventilators that can be moved to locations in need with sufficient notice^5,6^. Longer-range forecasts are important for decisions such as the potential to open schools, universities and workplaces and under what circumstances^7^. Much longer-range forecasts—6 months to a year—are important for a wide range of policy choices, where efforts to reduce disease transmission must be balanced against economic outcomes such as unemployment and poverty^8^. Furthermore, vaccine and new therapeutic trialists need to select locations that will have sufficient transmission to test new products in the time frame when phase three clinical trials are ready to be launched. Nevertheless, hundreds of forecasting models have been published and/or publicly released, and it is often not immediately clear which models have had the best performance or are most appropriate for predicting a given aspect of the pandemic.

Existing COVID-19 forecasting models differ substantially in methodology, assumptions, range of predictions and quantities estimated. Furthermore, mortality forecasts for the same location have often differed substantially, in many cases by more than an order of magnitude, even within a 6-week forecasting window. The challenge for decision-makers seeking input from models to guide decisions, which can impact many thousands of lives, is therefore not the availability of forecasts, but guidance on which forecasts are likely to be most accurate. Out-of-sample predictive validation—checking how well past versions of forecasting models predict subsequently observed trends—provides insight into future model performance^9^. Although some comparisons have been conducted for models describing the epidemic in the United States^10–13^, to our knowledge, similar analyses have not been undertaken for models covering multiple countries, despite the growing global impact of COVID-19.

This analysis, conducted by members of the Institute for Health Metrics and Evaluation (IHME) COVID-19 Forecasting team, introduces a publicly available evaluation framework, including full access to all data and code (https://github.com/pyliu47/covidcompare), for assessing the predictive validity of COVID-19 mortality forecasts. The framework and associated open-access software can be routinely used to track model performance and is updated in an ongoing fashion as new models are released. This will, over time, serve as a reference for decision-makers on historical model performance and provide insight into which models should be considered for critical decisions in the future.

## Results

### Model comparison framework

Seven models, which fit all inclusion criteria, were evaluated (Table 1). These included those modelled by DELPHI-MIT (Delphi)^14,15^, Youyang Gu (YYG)^10^, the Los Alamos National Laboratory (LANL)^16^, Imperial College London (Imperial)^17^, the SIKJ-Alpha model from the USC Data Science Lab (SIKJalpha)^18^ and the Institute for Health Metrics and Evaluation (IHME)^19^ (see “Methods” section for more details). Results are presented in the main text for two main predictive tasks: (1) predicting the magnitude of mortality and (2) predicting the timing of peak mortality (see “Methods”). Mortality is used as the key indicator both because of its relevance to decision-makers and also because it is the one indicator that is commonly reported across models. Magnitude results are presented in the main text for models that continued to produce forecasts at the time of publication of this paper, while peak timing results are presented for models released early enough to capture the first peak in most locations. Results for all historical models are shown in the Supplementary. The magnitude of mortality results in the main text is presented according to two main analytical approaches. In the “most current” approach, the most recent 4-week period allowing for the calculation of errors is selected for each extrapolation length. In the “month stratified” approach, models from October were used to calculate errors at each length of extrapolation, with all months shown in the Supplementary. In each case, errors were assessed from 1 to 12 weeks of forecasting (see “Methods” for more details).

**Table 1:** Models included in the study.

Model	Data access	Model type	Mortality input data	Model outputs	Geographies	Range	Model structure and assumptions
IHME	http://www.healthdata.org/covid/data-downloads	Mortality Spline + SEIR	JHU + local and national governments	Hospital and ICU admissions, ventilator, hospital beds utilisation; confirmed daily and cumulative cases; daily and cumulative mortality	164 countries*	June 1, 2021	Covariate-adjusted (population, testing, mandates, flu/pneumonia seasonality, mask use, etc.) SEIR model based on daily deaths estimates harmonised with testing, hospitalisation via a random knot spline.
UCLA-ML	https://github.com/uclaml/ucla-covid19-forecasts	SuEIR	JHU and NYT for US States	Daily and cumulative mortality, daily and cumulative confirmed cases. daily and cumulative ICU hospitalisations cases	27 countries*	May 16, 2021	SEIR model structure that also incorporates unreported cases and hospitalisations. Uses machine learning algorithms for parameter selection.
Youyang Gu	https://github.com/youyanggu/covid19_projections	SEIR	JHU	Daily and cumulative mortality; daily, active, and cumulative cases	73 countries*	November 1, 2020**	SEIR model with three R0 values corresponding to (1) Pre-mitigation (2) Post mitigation (3) Post reopening. Performs grid search to optimise parameter selection.
MIT - DELPHI	https://github.com/COVIDAnalytics/DELPHI	SEIR	JHU	Cumulative mortality; active cases, cumulative detected cases, active hospitalised cases; cumulative hospitalised cases	159 countries*	April 15, 2021	Standard SEIR model adjusted for effective meta-analysis driven parameters of contact rate, under-detection, hospitalisation, and societal-governmental response measures (4 phased non-linear parametric model).
Imperial-LMIC	https://github.com/mrc-ide/global-lmic-reports	SEIR	Euro-CDC	Daily and cumulative cases; daily and cumulative mortality; ICU incidence, ICU demand, hospital incidence, hospital demand	178 countries	April 29, 2021	Modelled using SQUIRE, an age-standardised SEIR model with parameters for healthcare capacity and disease severity. Incorporates mobility dependent R0 based on Google mobility data. Baseline scenario assumes current levels of mobility and interventions persist.
LANL-GR	https://covid-19.bsvgateway.org/	Dynamic Growth	JHU	Confirmed daily and cumulative cases; daily and cumulative mortality	156 countries*	March 27, 2021	Estimates cases driven by a dynamic growth parameter, adjusted based on trends in observed cases. Mortality driven by estimated CFR, assumed to be consistent over the forecast period.
USC SIKJalpha	https://github.com/scc-usc/ReCOVER-COVID-19	SIKJalpha	JHU	Confirmed daily and cumulative cases; daily and cumulative mortality	177 countries*	February 19, 2021	Application of SIKJalpha epidemiological model which models temporally varying infection rates and human mobility. Models CFR as a function of cases with different infection times.

All seven models included in the study are shown. The full list of models assessed for inclusion is shown in Supplementary Data 1. Range indicates the last date upon which forecasts are available in the most current version of each model.

*Includes state-level estimates for the United States.

**No longer actively producing forecasts at the time of publication.

**Fig. 1:**
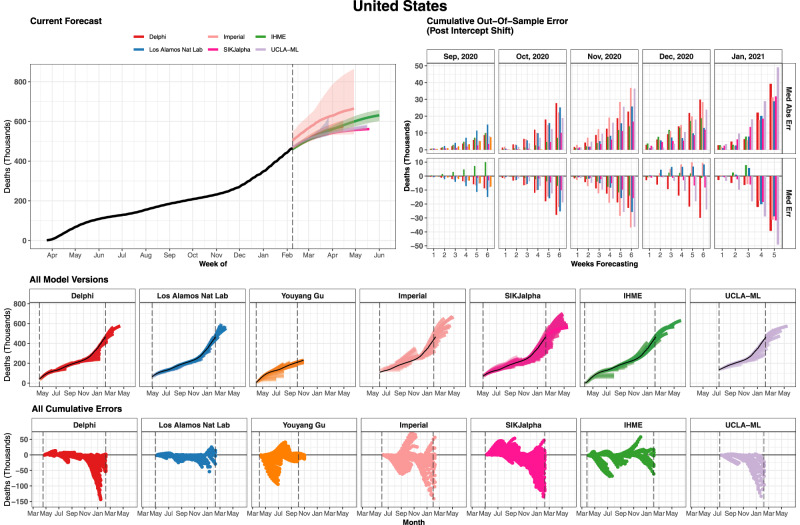
Cumulative mortality forecasts and prediction errors by model—example for the United States. The most recent version of each model is shown on the top left, as well as 95% prediction intervals when available. The middle row shows all iterations of each model as separate lines. The vertical dashed lines indicate the first and last model release date for each model. The bottom row shows all errors calculated at weekly intervals (circles). The top right panel summarises all observed errors, using median error (top) and median absolute error (bottom), by weeks of forecasting and month of model estimation. Errors incorporate an intercept shift to account for differences in each model’s input data. This figure represents an example for the United States of country-specific plots made for all locations examined in this study. Graphs for all geographies can be found in the Supplementary Information. Note that while certain models use different input data source than the other modelling groups causing apparently discordant past trends in the top-left panel. We plot raw estimates on the top-left panel; however, we implement an intercept shift to account for this issue in the calculation of errors. Delphi DELPHI-MIT (red), Los Alamos Nat Lab Los Alamos National Laboratory (blue), Youyang Gu (orange), Imperial   Imperial College London (peach), SIKjalpha USC SIKJ-alpha (pink), IHME Institute for Health Metrics and Evaluation (green), UCLA-ML UCLA Statistical Machine Learning Lab (purple).

The evaluation framework developed here for assessing how well models predicted the total number of cumulative deaths is shown in Fig. 1 for an example country—the United States, chosen as it has the highest number of reported COVID-19 deaths—and similar figures for all locations included in the study can be found in the Supplementary. Fig. 1, and similar figures in the Supplementary, also highlight the direction of error for each model in each location. When looking across iterations of forecasts, a wide range of variation can be observed for nearly all of the models. Nevertheless, in many locations, models largely reached consensus regarding trajectories in June–August 2020. Models diverged again when predicting trajectories for November 2020–February 20201, as some models predicted upticks related to seasonality, while others projected continued slow declines in mortality.

### Comparison of cumulative mortality forecasts

**Fig. 2:**
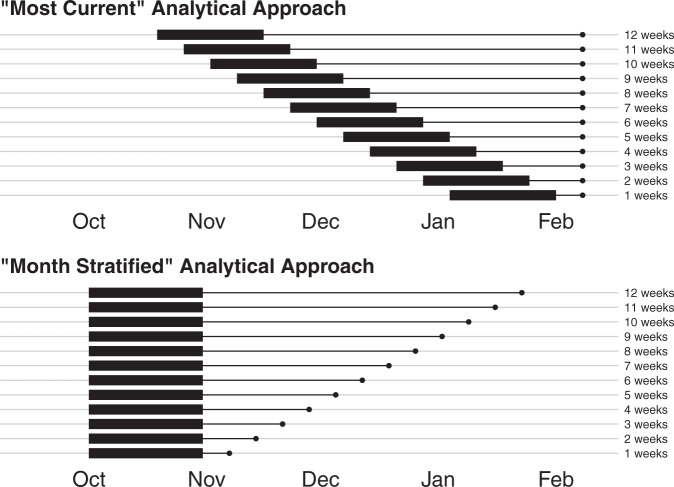
Illustration of the analytical framework. This figure highlights the analytical framework presented in the main text. Part A highlights the “most current” approach, which is used to select the data shown in Fig. 3. Part B highlights the “month stratified” approach used for Figs. 4 and 5. The *Y* axis shows the number of weeks of extrapolation for each scenario, while the *x* axis shows a range of model date—the date on which a model was released. The thick band in each plot highlights the 4-week window of model dates used for each extrapolation week value. The thin line shows the period for which each set of models is extrapolating before errors are calculated. In the top panel, the most recent 4 weeks of model dates are used for each extrapolation length. Therefore, for 1-week errors, models from January and February 2021 were used, whereas for 12-week errors, models from October and November 2020 were used. In the bottom panel, models from October are used in all cases. The analytic strategy highlighted in the top panel provides the most recent evidence possible for each extrapolation length. The strategy at the bottom allows for a more reliable assessment of how errors grow with increased extrapolation time.

**Fig. 3:**
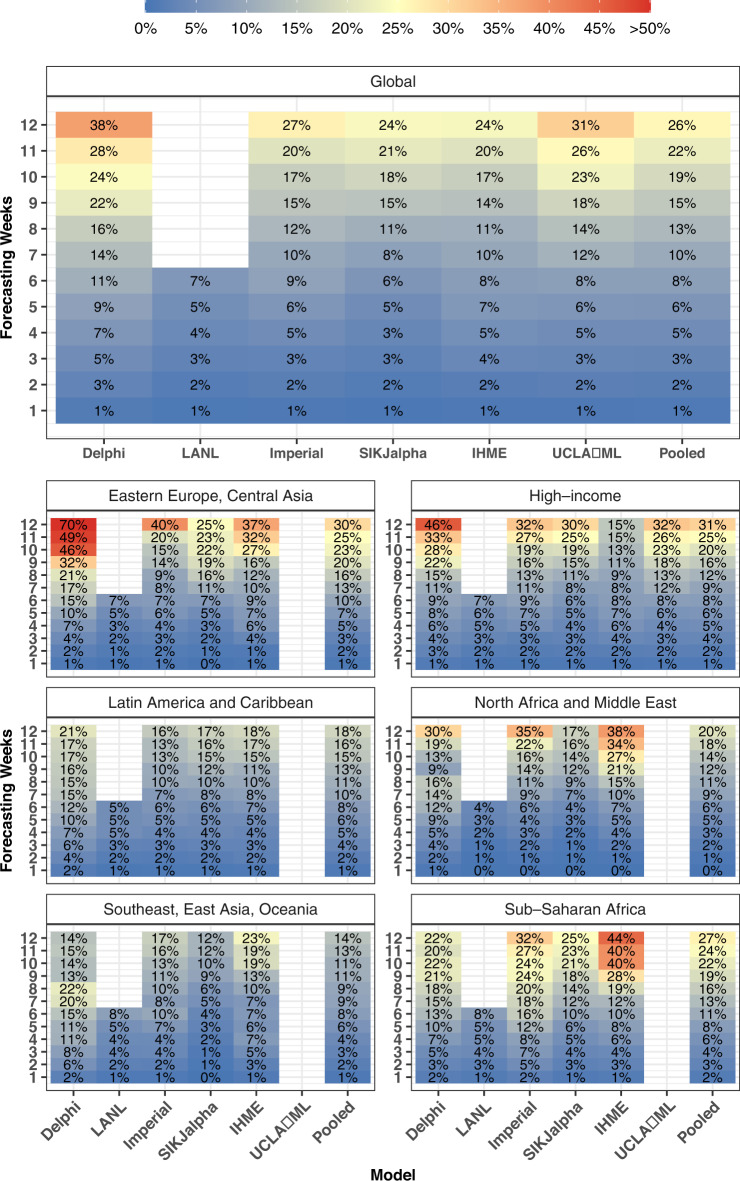
Most current—cumulative mortality accuracy—median absolute percent error. Median absolute percent error values, a measure of accuracy, were calculated across all observed errors at weekly intervals, for each model by weeks of forecasting and geographic region. Values that represent fewer than five locations are masked due to the small sample size. Models were included in the global average when they included at least five locations in each region. Pooled summary statistics reflect values calculated across all errors from all models, in order to comment on aggregate trends by time or geography. Results are shown here for the most recent 4-week window allowing for the calculation of errors at each point of extrapolation (see Fig. 2 and “Methods”). Results from other months are shown in the Supplementary Information. The colour reflects 50 for values above 50 to prevent extreme values obscuring the scale.

Figure 2 highlights the analytical framework, including the “most-current” and “month stratified” approached. Figure 3 highlights the most recent errors for each length of extrapolation. For all models, the most recent 1-week errors, reflecting forecasts created in January and February, were ~1%. The 12-week median absolute percent errors (MAPE), reflecting models produced in October and November, ranged from 23.6% for the IHME model, to 37.6% for the Delphi model. At the global level pooling across models, the most recent 6-week MAPE value was 8.0%.

**Fig. 4:**
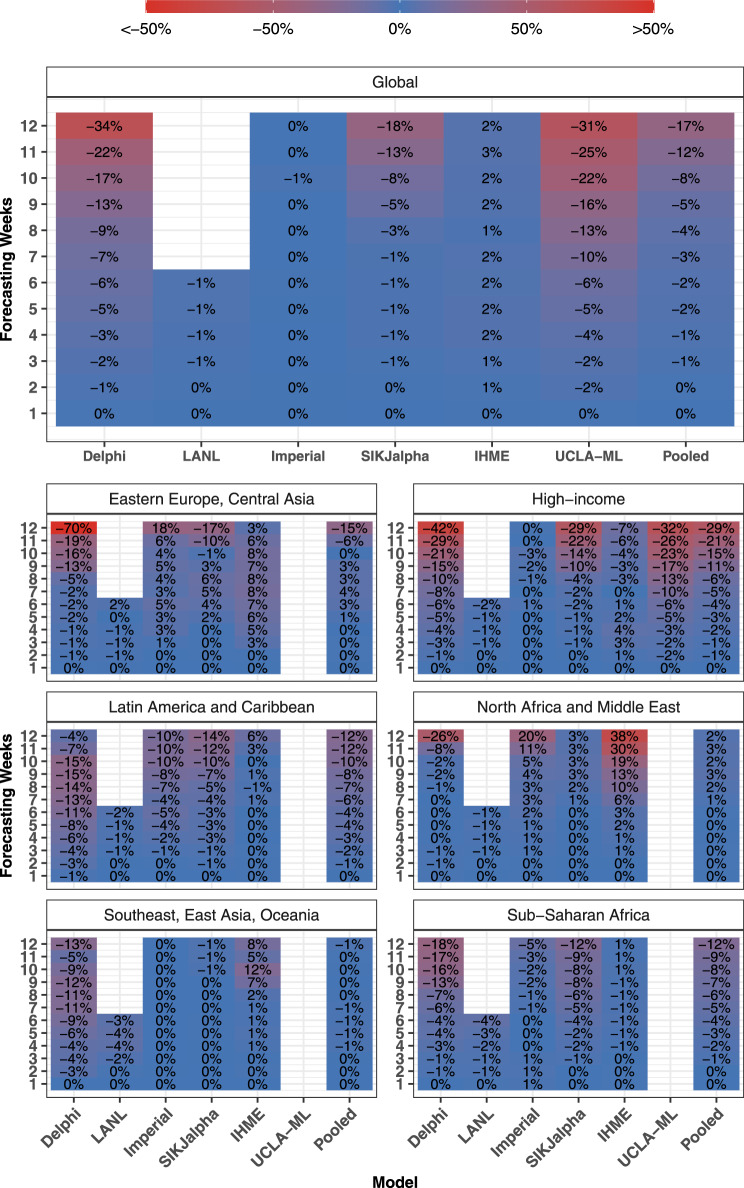
Month stratified October models—cumulative mortality bias—median percent error. Median percent error values, a measure of bias, were calculated across all observed errors at weekly intervals, for each model, by weeks of forecasting and geographic region. Values that represent fewer than five locations are masked due to small sample size. Models were included in the global average when they included at least five locations in each region. Pooled summary statistics reflect values calculated across all errors from all models, in order to comment on aggregate trends by time or geography. Results are shown here for models released in October, and results from other months are shown in the Supplementary. The colour reflects 50 for values above 50, and −50 for values below −50, to prevent extreme values obscuring the scale.

Systematic assessments of bias for all models produced in October are shown in Fig. 4 and Supplementary Fig. 2. The Delphi, LANL and UCLA-ML models from October underestimated mortality, with median percent errors of −9.2%, −9.1 and −10.2% at 6 weeks, respectively, the remaining models were relatively unbiased. In previous months, a number of models were shown to drastically overestimate mortality, especially for countries in Asia and Sub-Saharan Africa (Supplementary Fig. 2).

**Fig. 5:**
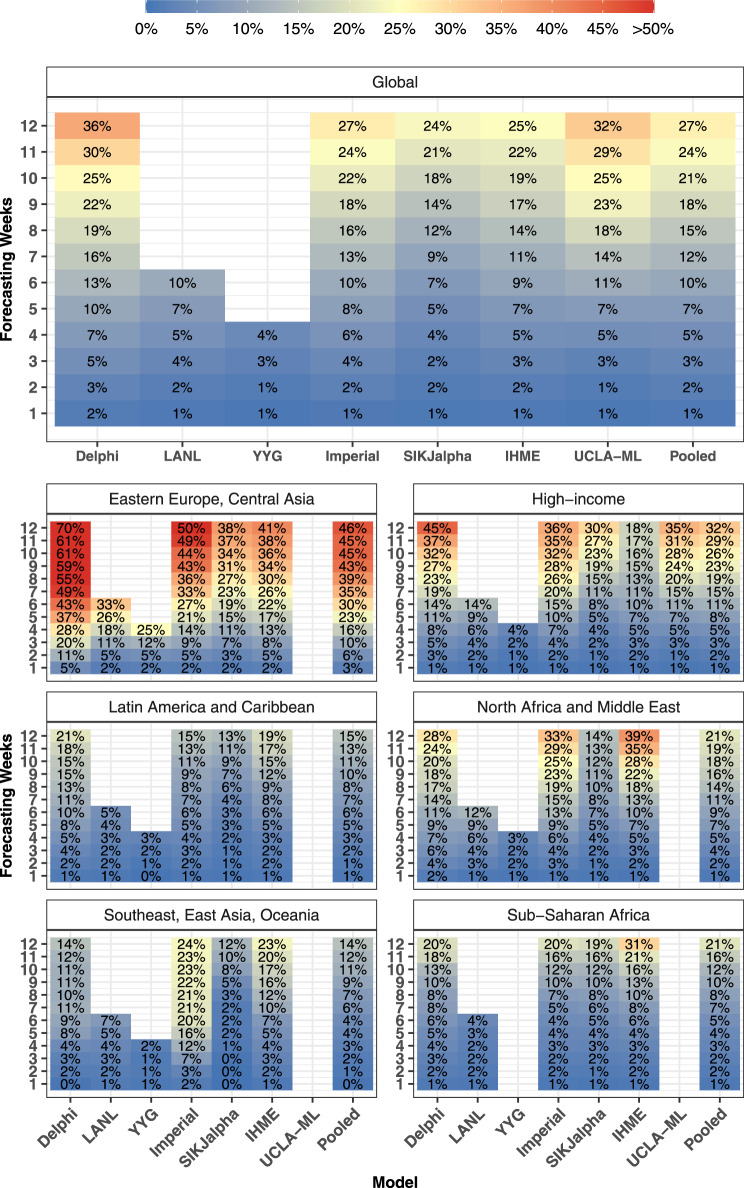
Month stratified october models—cumulative mortality accuracy—median absolute percent error. Median absolute percent error values, a measure of accuracy, were calculated across all observed errors at weekly intervals, for each model by weeks of forecasting and geographic region. Values that represent fewer than five locations are masked due to the small sample size. Models were included in the global average when they included at least five locations in each region. Pooled summary statistics reflect values calculated across all errors from all models, in order to comment on aggregate trends by time or geography. Results are shown here for models released in October, and results from other months are shown in the Supplementary Information. The colour reflects 50 for values above 50 to prevent extreme values obscuring the scale.

Overall model performance for models produced in October is shown for cumulative deaths by week in Fig. 5. As one might expect, MAPE tends to increase by the number of weeks of extrapolation. Across models released in October, the MAPE rose from 1.0% at 1 week to 26.9% at 12 weeks. Decreases in predictive ability with greater periods of extrapolation were similarly noted for errors in weekly deaths (Supplementary Fig. 3). At the global level, MAPE at 6 weeks was <10% for the IHME (9.4%) and SIKJalpha (6.9%) models, and <15% for all models. At 12 weeks, MAPE values were lowest for the SIKJalpha (23.7%) and IHME (24.6%) model, while the Delphi model had the highest MAPE (36.4%). Predictive performance between models was generally similar for median absolute errors (MAEs) (see Supplementary Fig. 4).

Figure 5 also shows that model performance varies substantially by region. Among models released in October, the largest errors were seen among regions with many countries in the Northern Hemisphere, such as Eastern Europe and Central Asia, with a 6-week MAPE of 29.8%, and high-income countries with 11.4%. Nevertheless, among models released in June–August, countries in the Southern Hemisphere had the highest errors. For example, among models from July, Sub-Saharan Africa and Latin America and the Caribbean, had the highest 6-week MAPE values of 24.7% and 21.6%, respectively. Individual model performance and availability also varied by region.

### Comparison of peak daily mortality timing

**Fig. 6:**
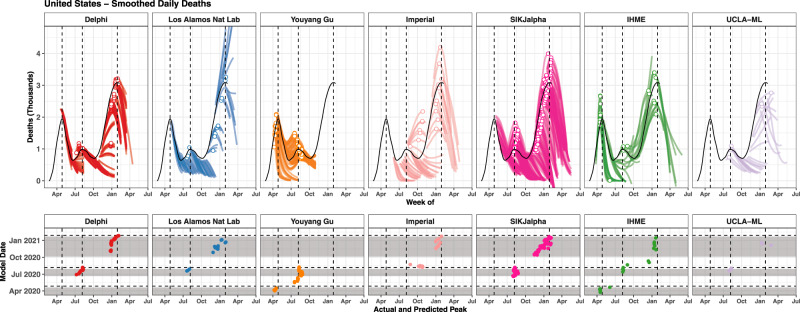
Observed vs predicted to peak in daily deaths—example for the United States. Observed daily deaths, smoothed using a loess smoother, are shown as a black line (top). The observed peak in daily deaths is shown with a vertical dashed line (top and bottom). All versions of each model are shown (top), and each model version that was released at least one week prior to the observed peaks has its estimated peak shown with a point (top and bottom). Estimated peaks are shown in the bottom panel (circles) with respect to their predicted peak date (*X* axis) and model date (*Y* axis). The grey bands represent the windows prior to each peak within which forecasted peaks were considered, which extend from when the time series began to increase, to 1 week prior to each peak. Values are shown for the United States, and similar graphs for all other locations are available in the Supplementary. Delphi DELPHI-MIT (red), Los Alamos Nat Lab   Los Alamos National Laboratory (blue), Youyang Gu (orange), Imperial   Imperial College London (peach), SIKjalpha   USC SIKJ-alpha (pink), IHME   Institute for Health Metrics and Evaluation (green), UCLA-ML   UCLA Statistical Machine Learning Lab (purple).

**Fig. 7:**
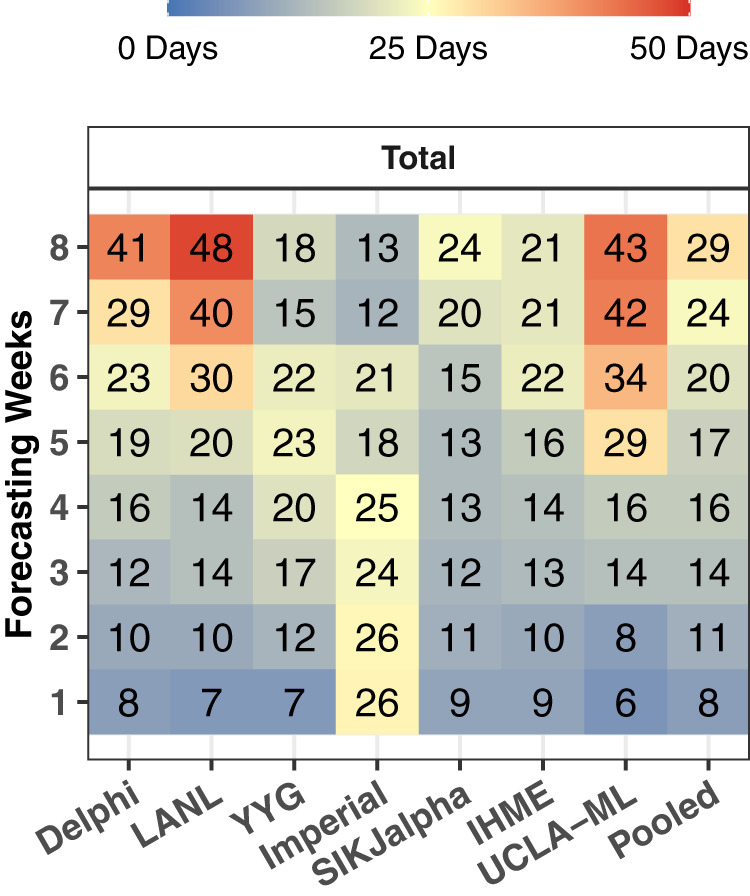
Peak timing accuracy—median absolute error in days. The median absolute error in days is shown by the model and the number of weeks of forecasting. Errors only reflect models released at least 7 days before each observed peak in daily mortality. One week of forecasting refers to errors occurring from 7 to 13 days in advance of the observed peak, while 2 weeks refers to those occurring from 14 to 20 days prior, and so on, up to 8 weeks, which refers to 56–62 days prior.

**Fig. 8:**
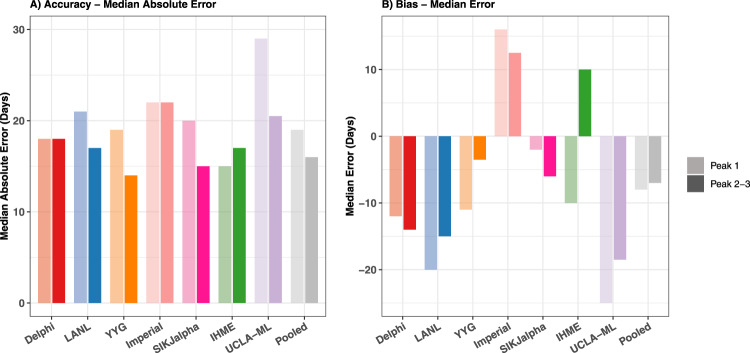
Peak timing accuracy by first or subsequent peak. Median absolute error (**A**) and median error (**B**) in days is shown by model and type of peak, either first or subsequent (second or third). Errors only reflect models released at least 7 days before each observed peak in daily mortality. Lighter bars reflect first peaks and darker bars reflect subsequent peaks. Illustrations of first and subsequent peaks can be seen for all locations in the supplementary daily death smoothing figures.

The evaluation framework for exploring the ability of models to predict the timing of peak mortality accurately—a matter of paramount importance for health service planning—is shown in Fig. 6 for an example location, the United States. Similar figures for all locations are shown in the Supplementary. Median absolute errors (MAE) for peak timing also rose with increased forecasting weeks, from 8 days at 1 week to 29 days at 8 weeks (Fig. 7). The MAE at 6 weeks ranged from 15 days for the SIKJalpha model to 34 days for the UCLA-ML model, with an overall error across models of 20 days (Fig. 7). There were no immediately obvious differences in peak timing performance from month to month (Supplementary Fig. 5). Figure 8 assesses the predictive performance for each model in predicting peak timing, stratified by first or subsequent peaks. Overall, peak timing prediction improved slightly between first and subsequent peaks, with median absolute error falling from 19 to 16 days. For both, models tended to predict peaks slightly too early, with a median error of −8 and −7 days for first and subsequent peaks, respectively, although some model-specific variation could be seen.

## Discussion

Seven COVID-19 models were identified that covered more than five countries, were regularly updated, publicly released and provided archived results for past forecasts. Taken together at 12 weeks, the models released in October had a median average percent error of 26.9%. Errors tend to increase with longer forecasts, rising from 1.0% at 1 week to 26.9% at 12 weeks. At 12 weeks of extrapolation, the best predictive performance among models considered at the global level was observed for the SIKJalpha and IHME models, with a MAPE of 23.7% and 24.6%, respectively, although the best-performing model varied by region. In the most current models, the 6-week MAPE across models was 8.0%, and all models had a MAPE of near or less than 10%. At 8 weeks of forecasting, on average, models predicting the timing of peak mortality with an average error of 29 days. In sum, numerous models showed surprisingly good performance, despite the complexities of modelling human behavioural responses and government interventions.

Although models largely converged in their predictions for the June–August 2020 period, forecasts began to diverge again among predictions made in September–November 2020 for the subsequent months. These later divergences were likely due to differences in model assumptions related to the effects of seasonality. Although major increases in COVID-19 mortality were seen in countries in the Northern Hemisphere during November 2020–February 2021, many models released in the preceding months did not explicitly consider seasonality. These models tended to estimate flat or decreasing trends in the winter months, whereas models that explicitly modelled seasonality predicted large magnitude increases.

We also observed substantial differences in average model predictive performance between regions, which can likely be explained by several factors. Data quality has been shown to vary substantially between countries, and many models were initially calibrated on data from early epidemics in China, Europe and the United States. Furthermore, different regions are at different stages of their epidemic at any given time. For many of the countries in sub-Saharan Africa for example, the challenge is predicting if, and when, large outbreaks will occur. It is therefore easier for a model to demonstrate large-magnitude errors when it predicts a completely different epidemic trajectory. Contrastingly, in some of the more established epidemics, it is easier to predict the nature of more stabilised, ongoing transmission dynamics. Similarly, the widespread surges in mortality for many nations in the Northern Hemisphere during November 2020–February 2021 drove a shifting regional pattern of error during this time. During June–August 2020, the highest errors were seen in sub-Saharan Africa—home to many nations with known data issues and low, albeit difficult-to-forecast mortality rates. However, during November 2020–February 2021, huge spikes in mortality occurred in Eastern Europe and Central Asia, as well as a number of other high-income countries, which many models failed to predict, resulting in the largest-magnitude errors occurring in these regions.

A forecast of the trajectory of the COVID-19 epidemic for a given location depends on four sets of factors: (1) attributes of the virus itself, including the transmissibility of the variant involved, (2) characteristics of the location, such as population density and the use of public transport, (3) individual behavioural responses to the pandemic, such as avoiding contact with others or wearing a mask and (4) the actions of governments, such as the imposition of a range of social distancing mandates. Given the complexity of forecasting human and governmental behaviours, especially in the context of a new pandemic, the performance of most of the models evaluated here was encouraging. Nevertheless, errors were observed to grow with greater extrapolation time for both magnitude of mortality and peak timing, indicating that governments and planners should recognise the wide uncertainty that comes with longer-range forecasts, and strategize accordingly. Hospital administrators may want to hedge on the higher end of the forecast range, while government policymakers may elect to use the mean forecast, depending on their risk tolerance.

We also note that the vast majority of COVID-19 forecasting models did not provide sufficient information to be included in this framework, as the research groups did not release publicly available and date-version forecasts. We would encourage all research groups forecasting COVID-19 indicators to consider providing historical versions of their models in a public platform for all locations, to facilitate ongoing model comparisons. This will improve reproducibility, the speed of development for modelling science and the ability of policymakers to discriminate between a burgeoning number of models^20^. Many of the models featured in this analysis were generally unbiased or tended to underestimate future mortality, while other models, such as the Imperial model, as well as many other published models that did not meet our inclusion criteria, tend to substantially overestimate transmission, even within the first 4 weeks of a forecast. This tendency towards overestimation among SEIR and other transmission-based models is easy to understand, given the potential for the rapid doubling of transmission. Nevertheless, sustained exponential growth in the transmission is not often observed, likely due to the behavioural responses of individuals and governments; both react to worsening circumstances in their communities, modifying behaviours and imposing mandates to restrict activities. Behavioural feedback in response to rising incidence has been shown to cause more linear growth in cases, as opposed to the exponential growth that could otherwise occur^21,22^. This endogenous behavioural response is commonly included in economic analyses; however, it has not been routinely featured in transmission dynamics modelling of COVID-19. More explicit modelling of the endogenous response of individuals and governments may improve future model performance for a range of models.

Modelling groups are increasingly providing both reference forecasts, describing likely future trends and alternative scenarios describing the potential effects of policy choices, such as school openings, the timing of mandate re-imposition or planning for hospital surges. For these scenarios, the error in the reference forecast—which we describe in this paper—is actually less important than the error in the effect implied by the difference between the reference forecast and policy scenario. Unfortunately, evaluating the accuracy of these counterfactual scenarios is an extremely challenging task. The validity of such claims depends on the supporting evidence for the assumptions about a policy’s impact on transmission. The best option for decision-makers is likely to examine the impact of these policies as portrayed by a range of modelling groups, especially those that have historically had reasonable predictive performance in their reference forecasts.

Given that a number of very different models demonstrated recent 6-week errors for cumulative deaths below 10%, it would likely be worthwhile to construct an ensemble of these models and evaluate the performance the ensemble compared to each component. Although from a logistical standpoint, creating an ensemble of the forecasts would be relatively straightforward, it would be more challenging to integrate such a model pool with scenarios assessing policy options, given that the models have highly different underlying structures. Nevertheless, the inclusion of the models shown here, and future models meeting criteria into an ensemble framework, is an important area for future research.

This analysis of the performance of publicly released COVID-19 forecasting models has limitations. First, we have focused only on forecasts of deaths, as they are available for all models included here. Hospital resource use is also of critical importance, however, and deserves future consideration. Nevertheless, this will be complicated by the heterogeneity in hospital data reporting; many jurisdictions report hospital census counts, others report hospital admissions, and still others do not release hospital data on a regular basis. Without a standardised source for these data, assessment of performance can only be undertaken in an ad hoc way. Second, many performance metrics exist, which could have been computed for this analysis. We have focused on reporting median absolute percent error, as the metric is frequently used, quite stable and provides an easily interpreted number that can be communicated to a wide audience. The relative error is an exacting standard, however. For example, a forecast of three deaths in a location that observed only one may represent a 200% error, yet it would be of little policy or planning significance. Conversely, focusing on absolute error would create an assessment dominated by a limited number of locations with large epidemics. Future assessment could consider different metrics that may offer new insights, although the relative rank of performance by the model is likely to be similar.

When compiling all available forecasts from various modelling groups, including estimates from a wide range of time periods and geographies, extra care must be taken to ensure comparability between models. We use various techniques to construct fair companions, such as stratifying by region, the month of estimation and weeks of forecasting, and masking summary statistics representing a small number of values. Nevertheless, other researchers may prefer distinct methods of maximising comparability over a complex and patchy estimate space. Furthermore, the domains assessed here—the magnitude of total mortality and peak timing—are not an exhaustive list of all possible dimensions of model performance. By providing an open-access framework to compile forecasts and calculate errors, other researchers can build on the results presented here to provide additional analyses.

COVID-19 mortality forecasts have been used in myriad ways by policymakers as they make difficult decisions about resource management under unprecedented circumstances. Understanding likely trajectories in COVID-19 mortality is helpful for communicating to the general public when peaks have been reached, prospectively managing or moving resources between regions, as well as decisions about social distancing measures, stay-at-home orders and closing schools, universities and workplaces^1,7^. It is therefore of paramount importance that decision-makers can quickly assess how robust each modelling group’s predictions have been historical. Furthermore, we believe a similar approach could be adopted in future pandemics, and for modelling other infectious diseases such as influenza.

Ultimately, policymakers would benefit from considering a multitude of forecasting models as they consider resource planning decisions related to the response to the ongoing COVID-19 pandemic. This study provides a publicly available framework and codebase, which will be updated in an ongoing fashion, to continue to monitor model predictions in a timely manner, and contextualise them with prior predictive performance. It is our hope that this spurs conversation and cooperation amongst researchers, which might lead to more accurate predictions, and ultimately aid in the collective response to COVID-19. As the pandemic continues worldwide and resurges in Europe and North America are continuing to take a massive toll, regularly updating models, and continually assessing their predictive validity, will be important in order to provide stakeholders with the best tools for COVID-19 decision-making.

## Methods

### Systematic review

A total of 386 published and unpublished COVID-19 forecasting models were reviewed (see Supplementary). Models were excluded from consideration if they did not (1) produce estimates for at least five different countries, (2) did not extrapolate at least 4 weeks out from the time of estimation, (3) did not estimate mortality, (4) did not provide downloadable, publicly available results or (5) did not provide date-versioned sets of previously estimated forecasts, which are required to calculate subsequent out-of-sample predictive validity. Seven models, which fit all inclusion criteria, were evaluated (Table 1). These included those modelled by DELPHI-MIT (Delphi)^14,15^, Youyang Gu (YYG)^10^, the Los Alamos National Laboratory (LANL)^16^, Imperial College London (Imperial)^17^, the SIKJ-Alpha model from the USC Data Science Lab (SIKJalpha)^18^ and the Institute for Health Metrics and Evaluation (IHME)^19^.

Of note, each model really represents a modelling group, as the models in question produced by each group have changed. These shifts are not universally well-documented, yet are discussed in some detail in web materials and related manuscripts of most groups (see Table 1). For example, the IHME model changed with nearly every iteration of estimates produced, to reflect the availability of new covariates and new understandings of COVID-19 epidemiology. The models from IHME can be grouped into three general categories. Beginning March 25, IHME initially produced COVID forecasts using a statistical curve fit model (IHME-CF), which was used through April 29 for publicly released forecasts^1^. On May 4, IHME switched to using a hybrid model, drawing on a statistical curve fit the first stage, followed a second-stage epidemiological model with susceptible, exposed, infectious, recovered compartments (SEIR)^23^. This model was used through May 26. On May 29, the curve fit stage was replaced by a spline fit to the relationship between log cumulative deaths and log cumulative cases, while the second-stage SEIR model remained the same^24^. This model is the basis for recently published work on US State level scenarios of COVID-19 projections in the fall and winter of 2020/2021^25^ and was still in use at the time of this publication.

In some cases, numerous scenarios were produced by modelling groups, to describe the potential effects of interventions, or future trajectories under different assumptions. In each case, the baseline or status quo scenario was selected to evaluate model performance as that represents the modelers’ best estimate about the most probable course of the pandemic. Table 1 summarises information about each model assumptions, methodologies, input data, modelled outputs and forecasting range. Forecast estimates from these models were collated and harmonised, and are available in an ongoing fashion in a public Github repository (https://github.com/pyliu47/covidcompare).

### Model comparison framework

In order to conduct a systematic comparison of the out-of-sample predictive validity of international COVID-19 forecasting models, a number of issues must be addressed. Looking across models, a high degree of heterogeneity can be observed in numerous dimensions, including sources of input data, frequency of public releases of model estimates, geographies included in the results and how far into the future predictions are made available. Differences in each of these areas must be taken into account, in order to provide a fair and relevant comparison.

#### Input data

A number of sources of input data—describing observed epidemiological trends in COVID-19—exist, and they often do not agree for a given country and time point^26–28^. We chose to use mortality data collected by the Johns Hopkins University Coronavirus Resource Center as the in-sample data against which forecasts were validated at the national level and data from the New York Times for state-level data for the United States^27,28^. We chose to mainly rely on the Hopkins data as (1) it was the most common input data source used in the different models considered, (2) it covered all countries for which modelling groups produced forecasts, (3) although some quality issues were noted, and managed in our analysis, largely quality was deemed acceptable and (4) data were made publicly available on a GitHub page and updated daily, which facilitates the maintenance of a timely comparison framework. Locations were excluded from the evaluation (including Ecuador and Peru) where models used alternative data sources, such as excess mortality, in settings with known marked under-registration of COVID-19 deaths and cases^29,30^. We adjusted for differences in model input data using intercept shifts, whereby all models were shifted to perfectly match the in-sample data for the date, on which the model was released (see Supplementary Note).

#### Frequency of public releases of model estimates

Most forecasting models are updated regularly, but at different intervals, and on different days. Specific days of the week have been associated with a greater number of reported daily deaths. Therefore, previous model comparison efforts in the United States—such as those conducted by the US Centers for Disease Control and Prevention—have required modellers to produce estimates using input data cut-offs from a specific day of the week^31^. For the sake of including all publicly available modelled estimates, we took a more inclusive approach, considering each publicly released iteration of each model. To minimise the effect of day-to-day fluctuations in death reporting, we focus on errors in cumulative and weekly total mortality, which are less sensitive to daily variation.

#### Geographies and time periods included in the results

Each model produces estimates for a different set of national and subnational locations and extrapolates a variable amount of time from the present. Each model was also first released on a different date, and therefore reflects a different window of the pandemic. Here, we also took an inclusive but stratified approach and included estimates from all possible locations and time periods. To increase comparability, summary error statistics were stratified by super-region used in the Global Burden of Disease Study^32^, weeks of extrapolation and month of estimation, and we masked summaries reflecting a small number of locations or time points. Models were included in the global predictive validity results only when they were present for all regions. Estimates were included at the national level for all countries, except the United States, where they were also included at the admin-1 (state) level, as they were available for most models. In order to be considered for inclusion, models were required to forecast at least 4 weeks into the future.

#### Outcomes

Finally, each model also includes different estimated quantities, including daily and cumulative mortality, number of observed or true underlying cases and various dimensions of hospital resource utilisation. The focus of this analysis is on mortality, as it was the most widely reported outcome, and it also has a high degree of societal, epidemiological and public health importance. We did not focus on forecasts of confirmed cases for several reasons. Certain models we wished to include did not provide an estimate of confirmed cases to subsequently assess predictive performance. Mortality, on the other hand, was available for all models. Furthermore, confirmed cases also depend on testing rates, which vary widely over time and across locations. Modelling-confirmed cases, therefore, represent different and perhaps larger challenges. Of course, death numbers also have limitations, but they are generally more reliable than case numbers, at least in the early stages of the pandemic, and in locations with limited capacity to test.

### Comparison of cumulative mortality forecasts

The total magnitude of COVID-19 deaths is a key measure for monitoring the progression of the pandemic. It represents the most commonly produced outcome of COVID-19 forecasting models, and perhaps the most widely debated measure of performance. The main quantity that is considered is errors in total cumulative deaths—as opposed to other metrics such as weekly or daily deaths—as it has been the most commonly discussed measure, to date, in academic and popular press critiques of COVID-19 forecasting models. Nevertheless, alternate measures are presented in the Supplementary. Errors were assessed for systematic upward or downward bias, and errors for weekly, rather than cumulative deaths, were also assessed. In calculating summary statistics, percent errors were used to control for the large differences in the scale of the epidemic between locations. Medians, rather than means, are calculated due to a small number of large-magnitude outliers present in a few time series. Errors from all models were pooled to calculate overall summary statistics, in order to comment on overarching trends by geography and time.

Results are presented using two analytical strategies in the main text. Both strategies are highlighted in Fig. 2. The “most current” approach is used to select the data shown in Fig. 3. The “month stratified” approach is used for Figs. 3 and 4. In the “most current” approach, the most recent 4 weeks of model dates are used for each extrapolation length. Therefore, for 1-week errors, models from October were used, whereas, for 12-week errors, models from July and August were used. This allows for the assessment of the most recent evidence possible for each set of errors displayed. Four-week periods are used to ensure that the results are not unduly biased by featuring only a small number of runs for each model.

In the “month stratified” approach, models from July are used in all cases. This strategy allows for a more reliable assessment of certain aspects of predictive validity, as the same models are being compared over time and geographies. For example, the month-stratified approach may provide a more comparable assessment of how errors grow with increased length of extrapolation. Models are shown for July in the main text—the most recent month allowing for assessment of errors at 12 weeks of forecasting—and errors stratified for all months are shown in the Supplementary.

In all cases, median absolute percent errors (MAPE) were used to capture the central tendency of the distribution of errors. MAPE was operationalized in the following manner, where *g* represents given geography (i.e. global, region, country or US state) and *t* represents a time period of exactly *n* weeks between forecast and ground truth, where *n* ranges from 1 to 12 weeks:

1 $${{{\mathrm{MAPE}}}}_{g,t={{\mathrm{median}}}({{\mathrm{absolute}}}({{{\mathrm{percent}}\;{\mathrm{error}}}}_{g,t}))}$$

where percent error represents the percent deviation between a forecasted level of mortality and the subsequently observed ground truth (actual level of reported mortality):

2 $${{{\mathrm{Percent}}\;{\mathrm{error}}}}_{g,t}=\frac{({{\mathrm{ground}}\, {\mathrm{truth}}}-{{\mathrm{forecasted}}})}{{{\mathrm{ground}}\,{\mathrm{truth}}}}* 100 \%$$

MAPE values calculated for a regional or global total include all the country-level data points from each time series contained in that geographic grouping. A MAPE corresponding to “6 weeks” includes only data points produced exactly 6 weeks prior to subsequently observed ground-truth data.

### Comparison of peak daily mortality timing

Each model was also assessed on how well it predicted the timing of peak daily deaths—an additional aspect of COVID-19 epidemiology with acute relevance for resource planning. Peak timing may be better predicted by different models than those best at forecasting the magnitude of mortality, and therefore deserves separate consideration as an outcome of predictive performance. In order to assess peak timing predictive performance, the observed peaks of daily deaths in each location were estimated first—a task complicated by the highly volatile nature of reported daily deaths values, and the presence of multiple peaks. Each time series of daily deaths was smoothed, and the dates of peaks observed in each location, as well as the predicted peaks for each iteration of each forecasting model were calculated (see Supplementary Note). A LOESS smoother was used, as it was found to be the most robust to daily fluctuations. The results shown here reflect only those locations for which at least one peak had passed at the time of publication, and for which at least one set of model results were available 7 days or more ahead of the peak date. Predictive validity statistics were stratified by the number of weeks in advance of the observed peak that the model was released, as well as the month in which the model was released. Results shown in the main text were pooled across months, as there was little evidence of dramatic differences over time (see Supplementary). There was an insufficient geographic variation to stratify results by regional groupings, given fewer data for peak timing relative to the magnitude of mortality, although that remains an important topic for further study.

Peak timing was assessed for each peak detected in each location, and only locations for which at least one peak could be detected were included in the framework. The supplementary peak timing figures show in detail which locations were found to have one or more peaks.

### Reporting summary

Further information on research design is available in the Nature Research Reporting Summary linked to this article.

## Supplementary information

Supplementary Information

Description of Additional Supplementary Files

Reporting Summary

Supplementary Data 1

## Data Availability

The forecasts from models used in this paper and observed death counts required to reproduce this analysis and its included visualisations are publicly available at (https://github.com/pyliu47/covidcompare). The repository version published in this paper is available at (10.5281/zenodo.4578760^33^). Source data for forecast estimates are as follows: DELPHI-MIT (https://github.com/COVIDAnalytics/website/tree/master/), Imperial College London-LMIC (https://github.com/mrc-ide/global-lmic-reports/tree/master/), IHME (http://www.healthdata.org/covid/data-downloads), LANL-GR (https://covid-19.bsvgateway.org/), USC SIKJalpha (https://github.com/scc-usc/ReCOVER-COVID-19), Youyang Gu (https://github.com/youyanggu/covid19_projections/tree/master/) and UCLA-ML (https://github.com/uclaml/ucla-covid19-forecasts). Observed mortality data were obtained from the 2019 Novel Coronavirus Visual Dashboard operated by the Johns Hopkins University Center for Systems Science and Engineering (JHU CSSE) (https://github.com/CSSEGISandData/COVID-19) and The New York Times (https://github.com/nytimes/covid-19-data).

## Code availability

All codes required to reproduce this analysis and its included visualisations are publicly available at (https://github.com/pyliu47/covidcompare). The version published in this paper is available at (10.5281/zenodo.4578760). All analysis were conducted using the R software language v4.0.2^34^.
