# Supplementary material for: Predictive performance of international COVID-19 mortality forecasting models
Source: Nat Commun. 2021 May 10;12:2609. doi: 10.1038/s41467-021-22457-w (PMC8110547; doi:10.1038/s41467-021-22457-w)
Supplement: Supplementary file 2 — Description of Additional Supplementary Files [file 41467_2021_22457_MOESM2_ESM.pdf]

Title: Supplementary Data 1

Description: This CSV file contains the data stemming from the systematic review process.
