# Supplementary material for: Predictive performance of international COVID-19 mortality forecasting models
Source: Nat Commun. 2021 May 10;12:2609. doi: 10.1038/s41467-021-22457-w (PMC8110547; doi:10.1038/s41467-021-22457-w)
Supplement: Supplementary file 3 — Reporting Summary [file 41467_2021_22457_MOESM3_ESM.pdf]

nature research

Corresponding author(s): EG

Last updated by author(s): Mar 3, 2021

Reporting Summary

Nature Research wishes to improve the reproducibility of the work that we publish. This form provides structure for consistency and transparency in reporting. For further information on Nature Research policies, see our Editorial Policies and the Editorial Policy Checklist.

Statistics

For all statistical analyses, confirm that the following items are present in the figure legend, table legend, main text, or Methods section.

n/a	Confirmed	
☐	☒	The exact sample size (*n*) for each experimental group/condition, given as a discrete number and unit of measurement
☐	☒	A statement on whether measurements were taken from distinct samples or whether the same sample was measured repeatedly
☒	☐	The statistical test(s) used AND whether they are one- or two-sided<br>*Only common tests should be described solely by name; describe more complex techniques in the Methods section.*
☒	☐	A description of all covariates tested
☒	☐	A description of any assumptions or corrections, such as tests of normality and adjustment for multiple comparisons
☐	☒	A full description of the statistical parameters including central tendency (e.g. means) or other basic estimates (e.g. regression coefficient) AND variation (e.g. standard deviation) or associated estimates of uncertainty (e.g. confidence intervals)
☒	☐	For null hypothesis testing, the test statistic (e.g. *F*, *t*, *r*) with confidence intervals, effect sizes, degrees of freedom and *P* value noted<br>*Give P values as exact values whenever suitable.*
☒	☐	For Bayesian analysis, information on the choice of priors and Markov chain Monte Carlo settings
☒	☐	For hierarchical and complex designs, identification of the appropriate level for tests and full reporting of outcomes
☒	☐	Estimates of effect sizes (e.g. Cohen's *d*, Pearson's *r*), indicating how they were calculated

Our web collection on statistics for biologists contains articles on many of the points above.

Software and code

Policy information about availability of computer code

Data collection	All code used for collation of data used in this analysis are publicly available at https://github.com/pyliu47/covidcompare. All analyses were conducted in R 4.0.2.
Data analysis	All code used in this analysis are publicly available at https://github.com/pyliu47/covidcompare. All analyses were conducted in R 4.0.2.

For manuscripts utilizing custom algorithms or software that are central to the research but not yet described in published literature, software must be made available to editors and reviewers. We strongly encourage code deposition in a community repository (e.g. GitHub). See the Nature Research guidelines for submitting code & software for further information.

Data

Policy information about availability of data

All manuscripts must include a data availability statement. This statement should provide the following information, where applicable:

- Accession codes, unique identifiers, or web links for publicly available datasets
- A list of figures that have associated raw data
- A description of any restrictions on data availability

The forecasts from models used in this manuscript and observed death counts required to reproduce this analysis its included visualizations are publicly available at (https://github.com/pyliu47/covidcompare). The repository version published in this manuscript is available at (DOI: 10.5281/zenodo.4578760). Source data for forecast estimates are as follows: DELPHI-MIT (https://github.com/COVIDAnalytics/website/tree/master/); Imperial College London-LMIC (https://github.com/mrc-ide/global-lmic-reports/tree/master/); IHME (http://www.healthdata.org/covid/data-downloads); LANL-GR (https://covid-19.bsvgateway.org/); USC SIKJalpha (https://github.com/scc-usc/ReCOVER-COVID-19); Youyang Gu (https://github.com/youyanggu/covid19_projections/tree/master/); UCLA-ML (https://github.com/uclaml/ucla-covid19-forecasts). Observed mortality data were obtained from the 2019 Novel Coronavirus Visual Dashboard operated by the Johns Hopkins University Center for Systems Science and Engineering (JHU CSSE) (https://github.com/CSSEGISandData/COVID-19) and The New York Times (https://github.com/

nytimes/covid-19-data).

Field-specific reporting

Please select the one below that is the best fit for your research. If you are not sure, read the appropriate sections before making your selection.

- [x] Life sciences
- [ ] Behavioural & social sciences
- [ ] Ecological, evolutionary & environmental sciences

For a reference copy of the document with all sections, see nature.com/documents/nr-reporting-summary-flat.pdf

Life sciences study design

All studies must disclose on these points even when the disclosure is negative.

Sample size: A census of all iterations of each forecasting model was included. This observational study did not use sampling.

Data exclusions: Models were excluded from consideration if they did not 1) produce estimates for at least five different countries, 2) did not extrapolate at least four weeks out from the time of estimation, 3) did not estimate mortality, 4) did not provide downloadable, publicly available results, or 5) did not provide date-versioned sets of previously estimated forecasts, which are required to calculate subsequent out-of-sample predictive validity.

Replication: All code and data used in this analysis are publicly available at https://github.com/pyliu47/covidcompare.

Randomization: Randomization is not applicable for the present study as there are no experimental groups used in this analysis.

Blinding: Blinding is not applicable for the present study as there are no experimental groups used in this analysis.

Reporting for specific materials, systems and methods

We require information from authors about some types of materials, experimental systems and methods used in many studies. Here, indicate whether each material, system or method listed is relevant to your study. If you are not sure if a list item applies to your research, read the appropriate section before selecting a response.

Materials & experimental systems

n/a	Involved in the study
x	Antibodies
x	Eukaryotic cell lines
x	Palaeontology and archaeology
x	Animals and other organisms
x	Human research participants
x	Clinical data
x	Dual use research of concern

Methods

n/a	Involved in the study
x	ChIP-seq
x	Flow cytometry
x	MRI-based neuroimaging
