# Supplementary material for: Predictive performance of international COVID-19 mortality forecasting models
Source: Nat Commun. 2021 May 10;12:2609. doi: 10.1038/s41467-021-22457-w (PMC8110547; doi:10.1038/s41467-021-22457-w)
Supplement: Supplementary file 1 — Supplementary Information [file 41467_2021_22457_MOESM1_ESM.pdf]

Supplementary Information for "Predictive performance of international COVID-19 mortality forecasting models"

Supplementary Note

In order to construct a framework to compare the out-of-sample predictive performance of COVID-19 mortality forecasting models, we conducted a comprehensive review of published and unpublished papers and models related to COVID forecasting. We identified models with date-versioned, publicly available mortality estimates for multiple countries, and create a code base to automatically download and process them into a common framework, as models update daily. We calculated errors for both the magnitude of mortality, and the timing of peak daily mortality. Finally, we calculated summary statistics describing the errors over a number of dimensions, including region, weeks of extrapolation, and month of estimation.

Comprehensive Review and Data Compilation

Conducting a comprehensive review of COVID-19 mortality forecasting is complicated by the unpublished nature of many models. In general, the pace of COVID-19 research has been rapid, to provide up-to-date evidence for an evolving situation. We therefore draw on both traditional and non-traditional sources to find models. We used PubMed to identify models published in journal articles, medRxiv to identify models published in preprint articles, and a collection of models curated by the Reich Lab and the US Centers for Disease Control describing forecasts for the United States, many of which also produce estimates internationally[16]. In PubMed and medRxiv we selected any articles with the terms "COVID", or "Coronavirus" present, as well as one of either term "Project" or "Forecast". At the time of publication of this article, n=686 articles had been screened. The systematic review framework was created to automatically download new citations for review, and subsequently be updated in a prospective manner, to stay current on future forecasting model efforts.

Each article or model was screened for 5 inclusion criteria:

1) Including a forecasting component (n=383),
2) and making projections for at least five locations (n=59),
3) and making projections for COVID-19 mortality (n=39),
4) and making projections at least four weeks into the future (n=35),
5) and providing publicly available, date-versioned estimates (n=8).

The seven models which fit these criteria are described in the main text (Table 1). All screened articles, including their inclusion and exclusion criteria, are described in Supplementary Data 1. The code used to compile candidate models and articles, and conduct the systematic review, is presented along with the remainder of the codebase.

For the seven models that were determined to meet all inclusion criteria, a codebase was developed to automatically download each date-versioned set of estimates as they became available. The model date (or estimation date) was assigned as the date on which the estimated became publicly available.

Locations were mapped onto the location hierarchy used by the Global Burden of Disease Study (GBD)[28] that categorizes all countries into regions, and regions into super-regions. When estimates were available at multiple geographic levels, the admin-0 (national) level results were used in all cases, except the United States, where both admin 0 and admin-1 (state) level results were used.

For models that provided only daily deaths, cumulative deaths were calculated as a rolling sum. Similarly, for models that provided only cumulative deaths, daily deaths were calculating by taking the daily first difference of cumulative deaths.

We chose to use mortality data collected by Johns Hopkins University Coronavirus Resource Center as the in-sample data against which forecasts were validated at the national level, and data from the New York Times for state-level data for the United States[20]. Data from both providers were mapped onto the GBD location hierarchy. For all analyses, the most recent set of input data were used, reflecting any potential revisions of historical trends.

Mortality Magnitude Predictive Validity

Before out-of-sample errors could be assessed for COVID-19 mortality, differences in input data sources between models were investigated and controlled. Estimates for the same locations, from different models, can differ greatly in magnitude of estimated mortality when they use input data sources that use different methodologies. To create a fair comparison, before errors were calculated, each model-, model-date-, and location-specific timeseries was shifted to match the “true” in-sample data for that model’s date of release. This was accomplished by calculating the timeseries-specific difference on the model date, and apply it to the entire timeseries as a fixed intercept shift. Subsequent forecasting errors were calculated using the resulting shifted time-series.

Out-of-sample errors were calculated for each timeseries at weekly intervals, beginning at one week, through six weeks of extrapolation. Summary statistics were first calculated across model-runs for each location, for use in country-specific graphics (Figure 1, for example). Summary statistics included the median absolute error, a measure of accuracy, and median error, a measure of bias. These were calculated separately by model, and by weeks of extrapolation.

Subsequently, errors were summarized between countries. Summary statistics included the median absolute percent error, a measure of accuracy, and median percent error, a measure of bias. Relative error statistics were used for all inter-country comparisons, to account for the substantial differences in magnitude of deaths between locations. Summary statistics were calculated in a stratified manner by regional groupings from the GBD[28], as well as weeks of extrapolation, and month of estimation. Pooled summary statistics were also calculated across models, to provide context about commonalities in trends in predictive performance over time and geographies.

Peak Timing

In order to calculate out-of-sample predictive validity statistics on how well each model predicted the timing of peak daily deaths, we smoothed daily death data, which are highly stochastic, applied an algorithm to detect peaks in both observed data and forecasted model estimates, and calculated errors in the difference in number of days between the observed and estimated peaks.

First, observed daily death data were smoothed to provide stable time-series that could be used for local maxima detection. We used various smoothers to accomplish this task, including a LOESS smoother with a span of 0.33, run separately for each location-specific timeseries, a 7-day rolling average, and a 3-day rolling average applied tenfold to the same timeseries. We chose to present results calculated using the LOESS smoother in the main text, as it was found to be the most robust method to daily stochasticity that could introduce false peaks. Although most models produced smooth timeseries of daily deaths, some also demonstrated stochasticity, and so all forecasted daily death timeseries were also smoothed with a LOESS smoother.

Peaks in smoothed, observed daily deaths were calculated according to the following algorithm. A peak *p* at time period *t* was defined as:

1) a local maximum in the time period *t*, and

2) *p* represents at least 5 deaths per day, and

3) *p* is at least 20% greater than the previous local minima, and

4) *p* is not exceeded in the next 30 days (*t* through *t*+30) by more than 20%, and

5) *p* it does not fall within the last seven days of the timeseries, and

Models were included for the evaluation of a given peak for a location if the model run was produced in the time window representing: A) after the onset of an upswing in deaths (date of positive second derivative in smoothed daily mortality), B) at least 7 days prior of the true peak. For locations with multiple peaks, windows were set relative to each peak (Supplementary Figure 6).

Peaks in forecasted trends were also identified with the same algorithm. Models included for consideration with respect to each peak were those released after deaths were already increasing for a particular location, yet that were released at least one week prior to the observed peak. These windows are identified in Figure 6, and Supplementary Figure 6. For a time-series in which no peak was identified using the above algorithm, for a location which did have a peak in observed data, the global maximum value was used. This captured errors among models that failed to ever predict a peak, despite a true peak being observed. Errors for locations with a true peak in observed data, for the model runs in which the model date was at least seven days prior to the true detected peak. Errors were defined as the difference between the date of the true peak and the estimated peak from each forecasting model, in days. Summary statistics included the median absolute error in days, as a measure of accuracy, and the median error in days as a measure of bias. Errors were stratified by model, and weeks of extrapolation, which was defined as:

$$Weeks\ of\ extrapolation\ =\ floor((peak\ date - model\_release\_date)/7)$$

Due to more limited data for peak timing compared to magnitude results (1 peak per time series maximum, as opposed to weekly contributions for magnitude) it was not possible to stratify results by geography.

Supplementary Figures

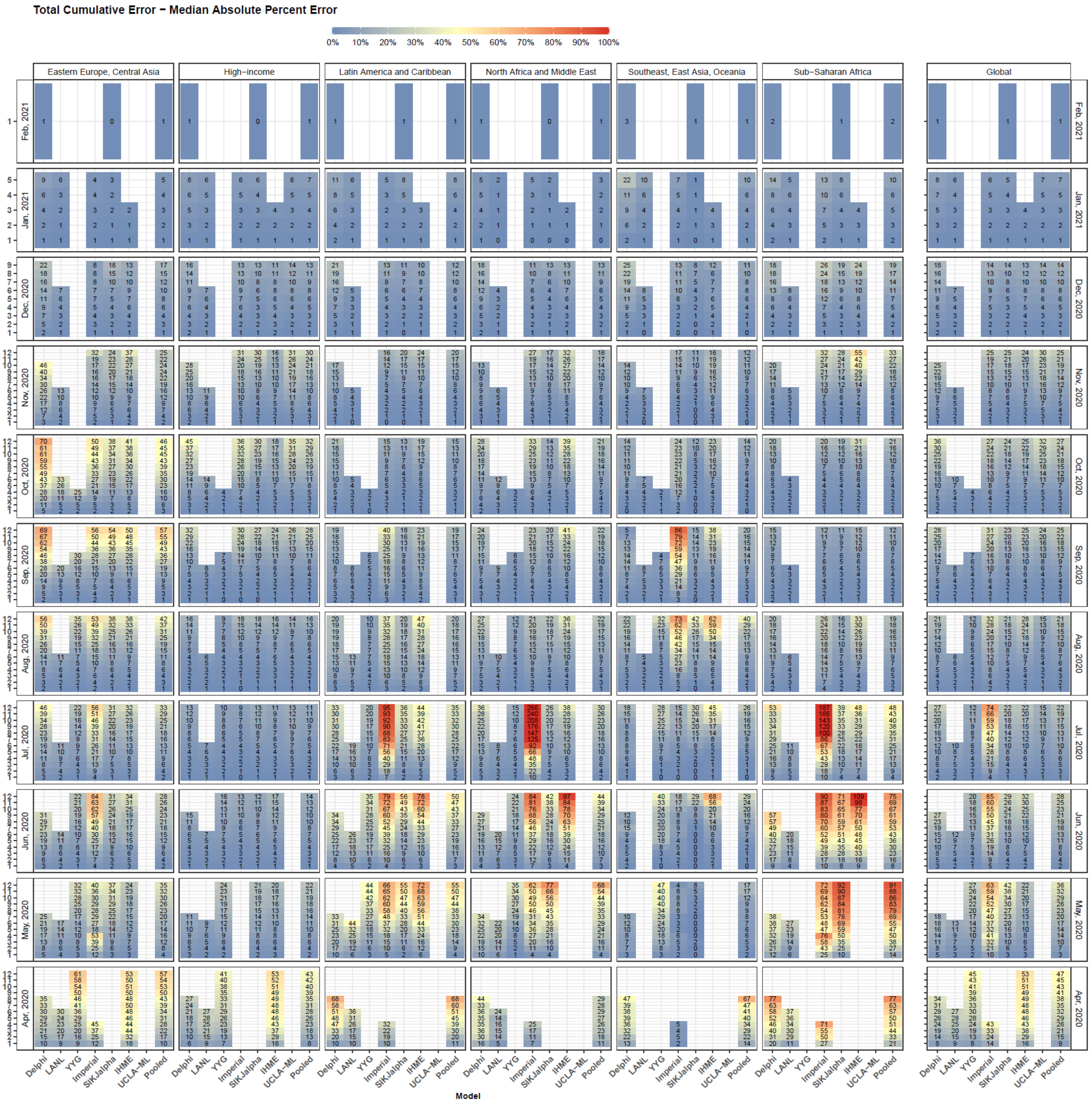

Supplementary Figure 1. Cumulative Mortality – Median Absolute Percent Error – Month of Estimation
Median absolute percent error values were calculated across all observed errors at weekly intervals, for each model, by month of estimation, weeks of forecasting, and super regional grouping used in the Global Burden of Disease Study. Values that represent fewer than five locations are masked due to small sample size. Models were included in the global average when they included at least five locations in each region. Pooled summary statistics reflect values calculated across all errors from all models, in order to comment on aggregate trends by time or geography.

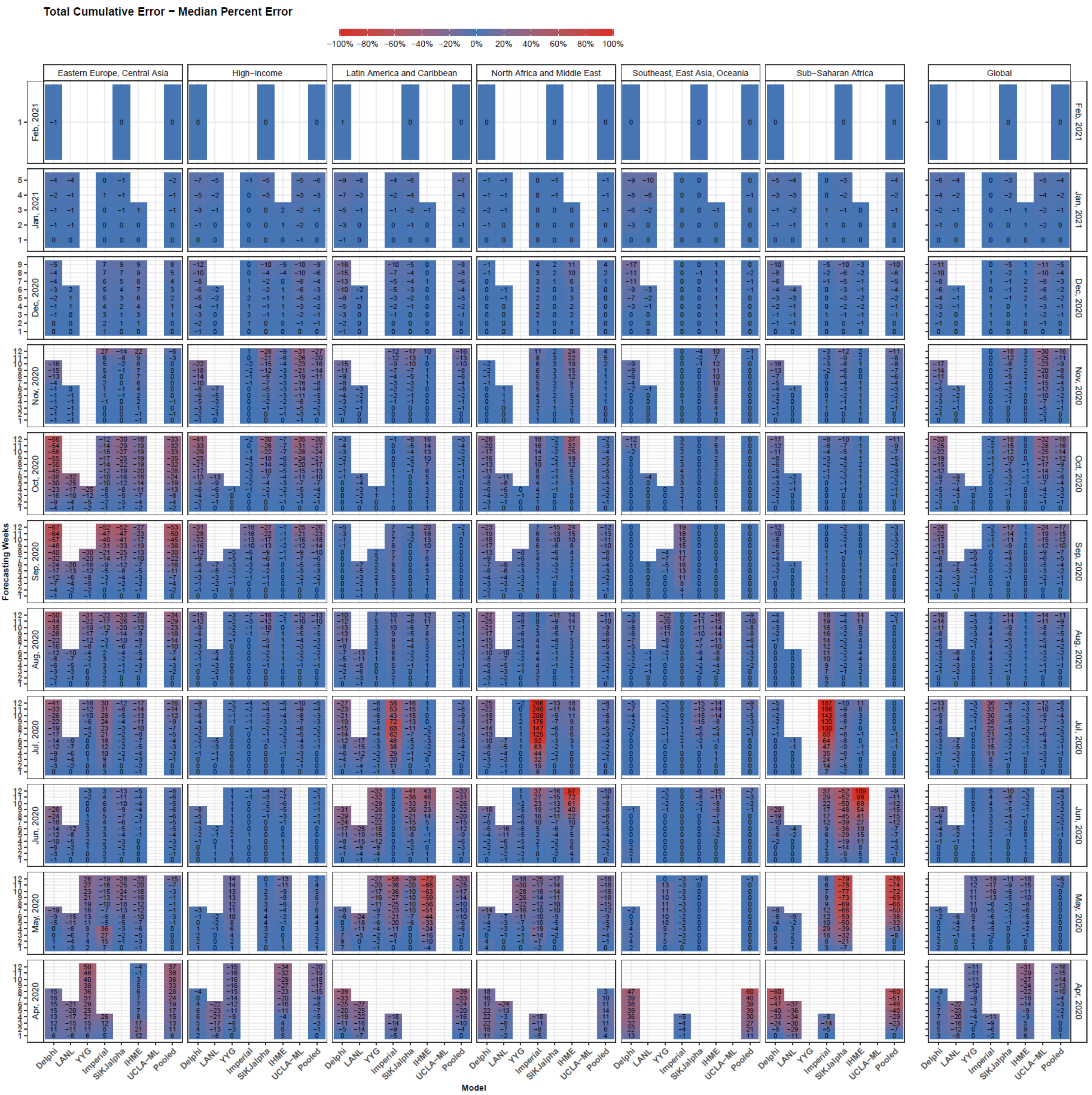

Supplementary Figure 2. Cumulative Mortality – Median Percent Error – Month of Estimation

Median percent error values, a measure of bias, were calculated across all observed errors at weekly intervals, for each model, by month of estimation, weeks of forecasting, and super regional grouping used in the Global Burden of Disease Study. Values that represent fewer than five locations are masked due to small sample size. Models were included in the global average when they included at least five locations in each region. Pooled summary statistics reflect values calculated across all errors from all models, in order to comment on aggregate trends by time or geography.

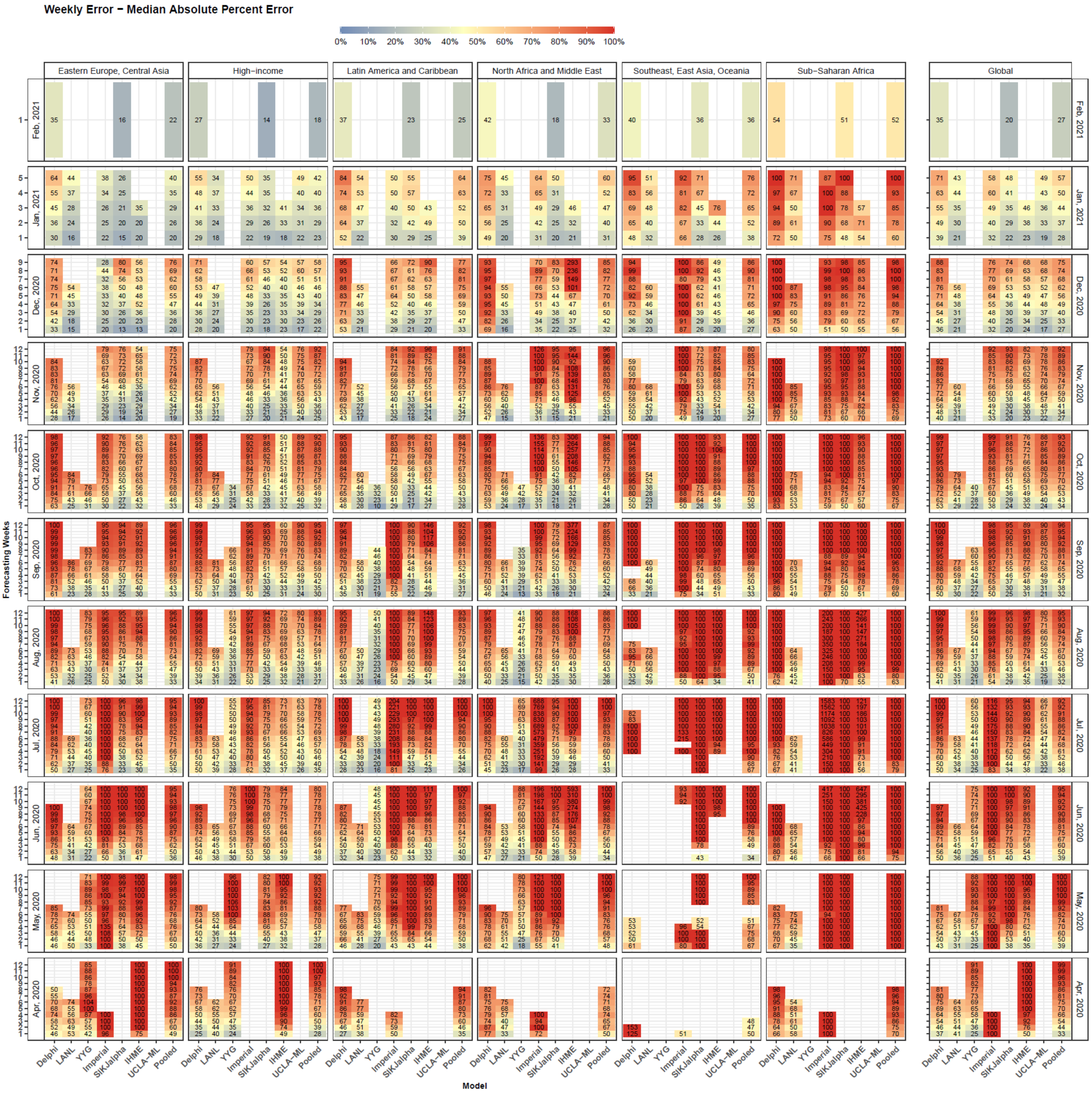

Supplementary Figure 3. Weekly Mortality – Median Absolute Percent Error – Month of Estimation Median absolute percent error values were calculated for weekly mortality rates across all observed errors at weekly intervals, for each model, by month of estimation, weeks of forecasting, and super regional grouping used in the Global Burden of Disease Study. Values that represent fewer than 5 locations are masked due to small sample size. Models were included in the global average when they included at least five locations in each region. Pooled summary statistics reflect values calculated across all errors from all models, in order to comment on aggregate trends by time or geography.

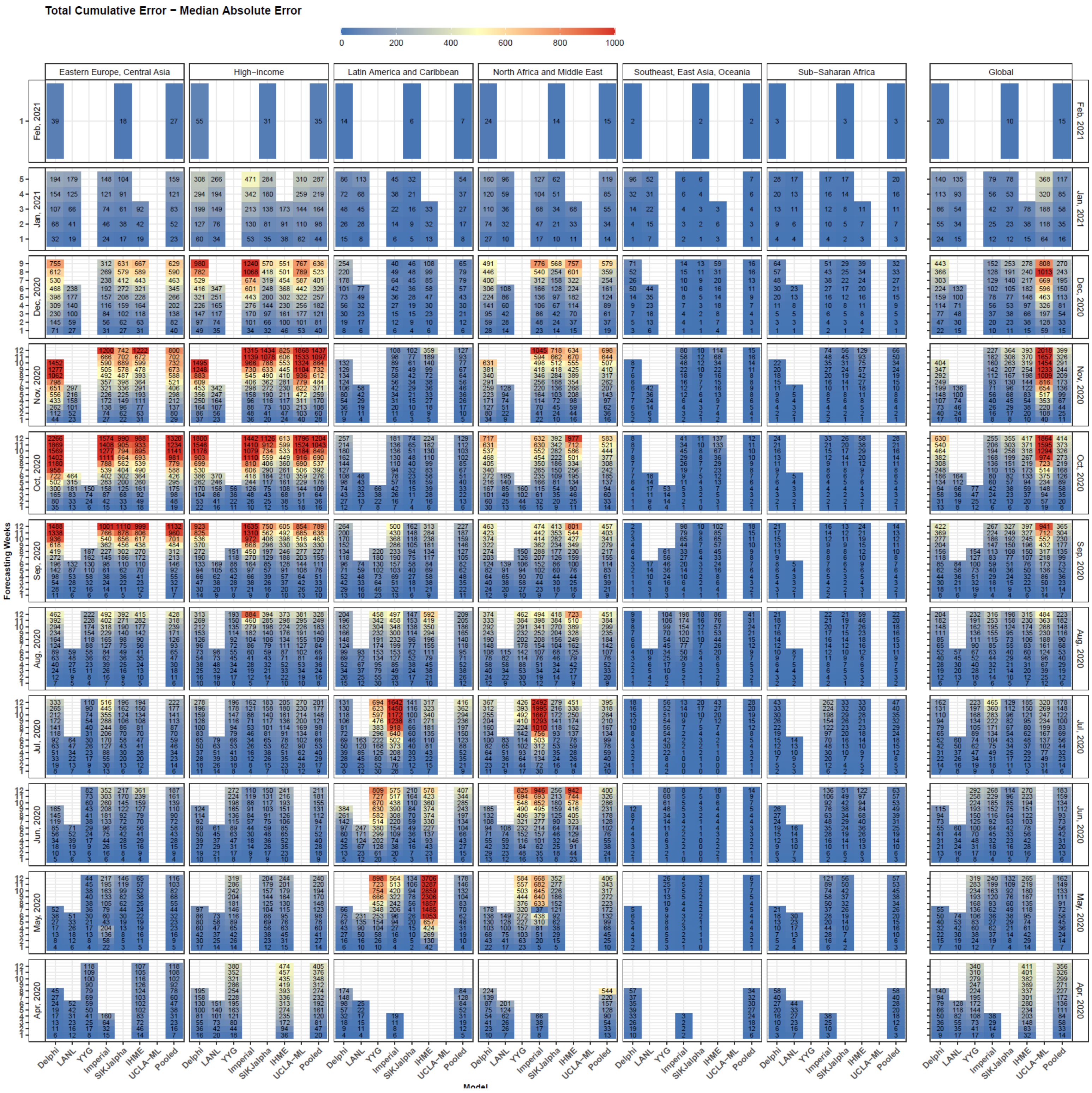

Supplementary Figure 4. Cumulative Mortality – Median Absolute Error – Month of Estimation Median absolute error values were calculated for cumulative mortality rates across all observed errors at weekly intervals, for each model, by month of estimation, weeks of forecasting, and super regional grouping used in the Global Burden of Disease Study. Values that represent fewer than 5 locations are masked due to small sample size. Models were included in the global average when they included at least five locations in each region. Pooled summary statistics reflect values calculated across all errors from all models, in order to comment on aggregate trends by time or geography.

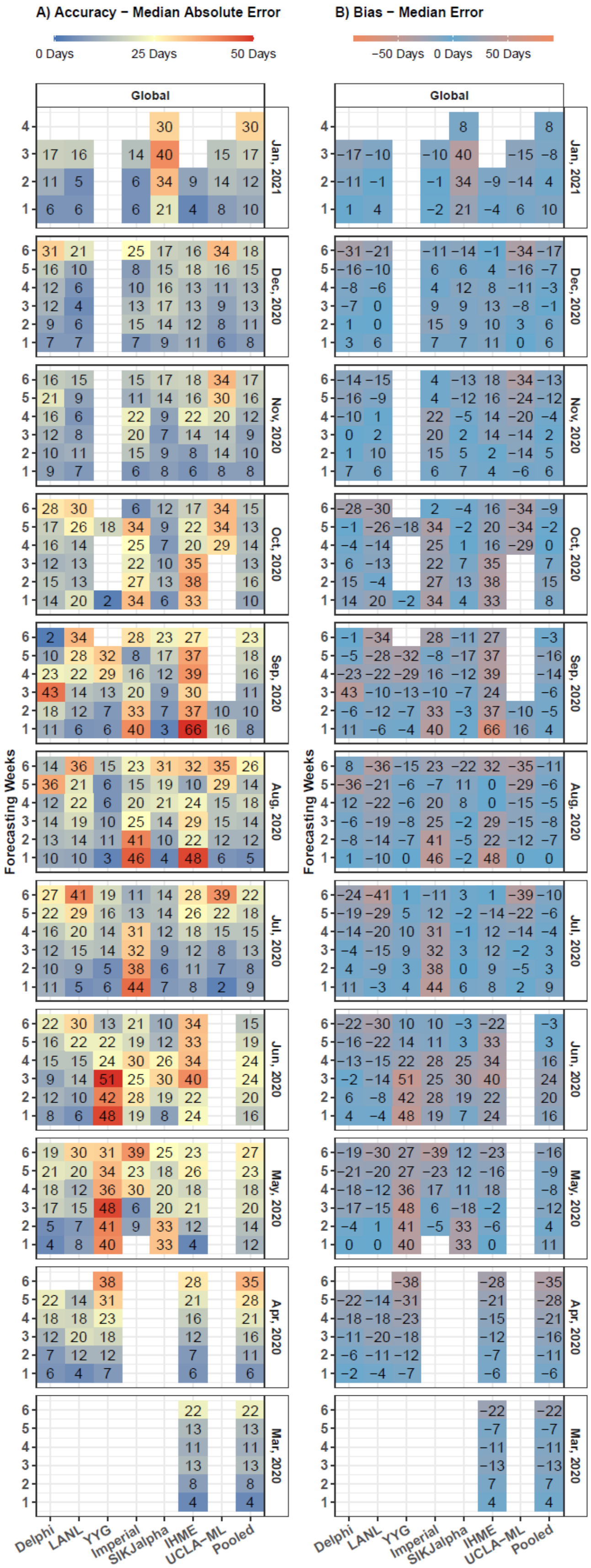

Supplementary Figure 5. Accuracy and Bias in Peak Timing by Month of Estimation

Median error in days are shown by model, weeks of forecasting, and estimation month. Errors only reflect models released at least seven days before the observed peak in daily mortality. One week of forecasting refers to errors occurring from seven to 13 days in advance of the observed peak, while two weeks refers to those occurring from 14 to 20 days prior, and so on, up to six weeks, which refers to 42-48 days prior.

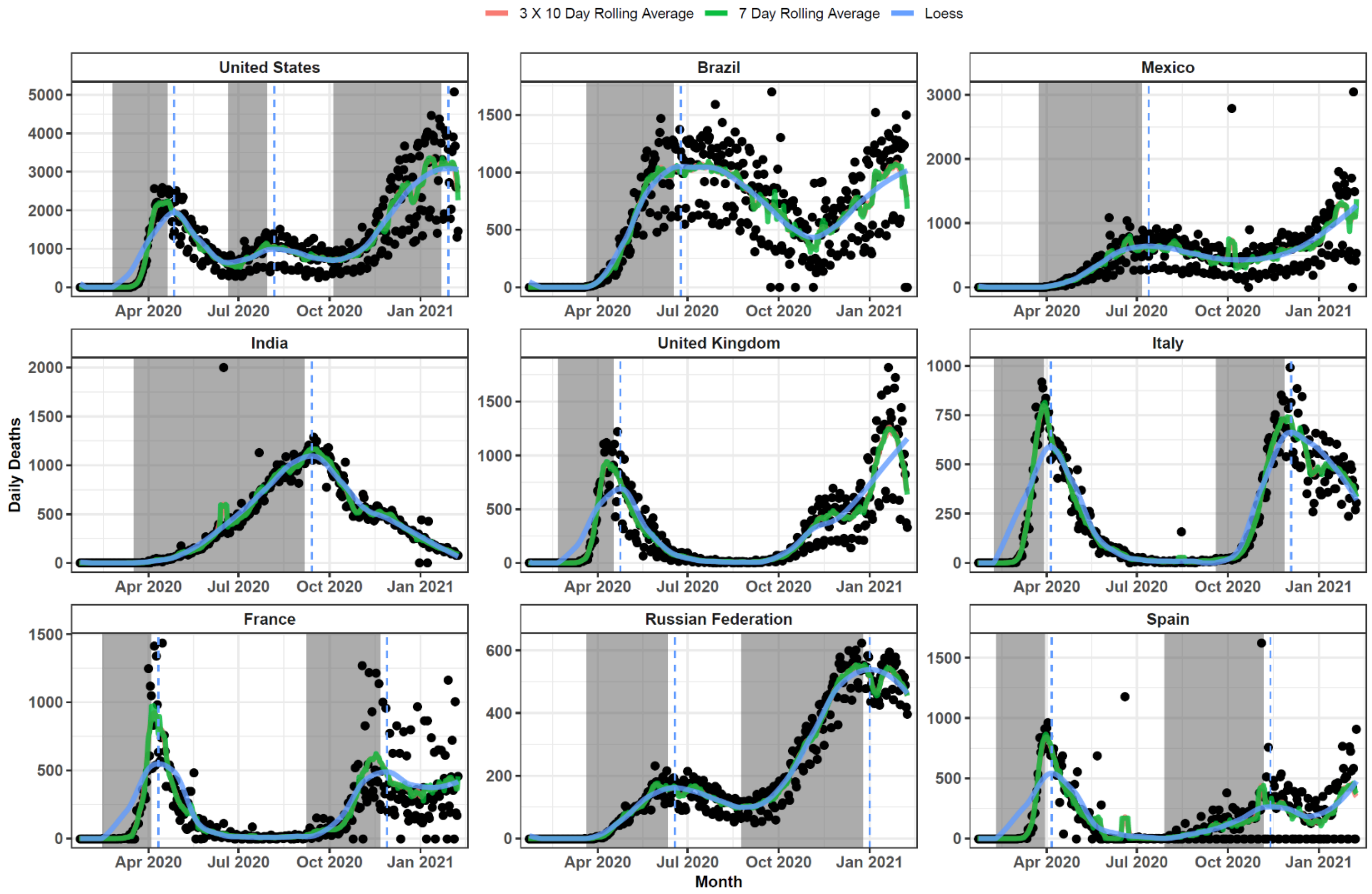

Supplementary Figure 6. Smoothing Method – Example for nine Countries

Daily deaths are shown for nine locations, as well as three methods used to smooth them prior to peak date calculation. Calculated peaks from the selected method (loess) are shown with dashed vertical lines. Smoothing method is shown by color.

United States

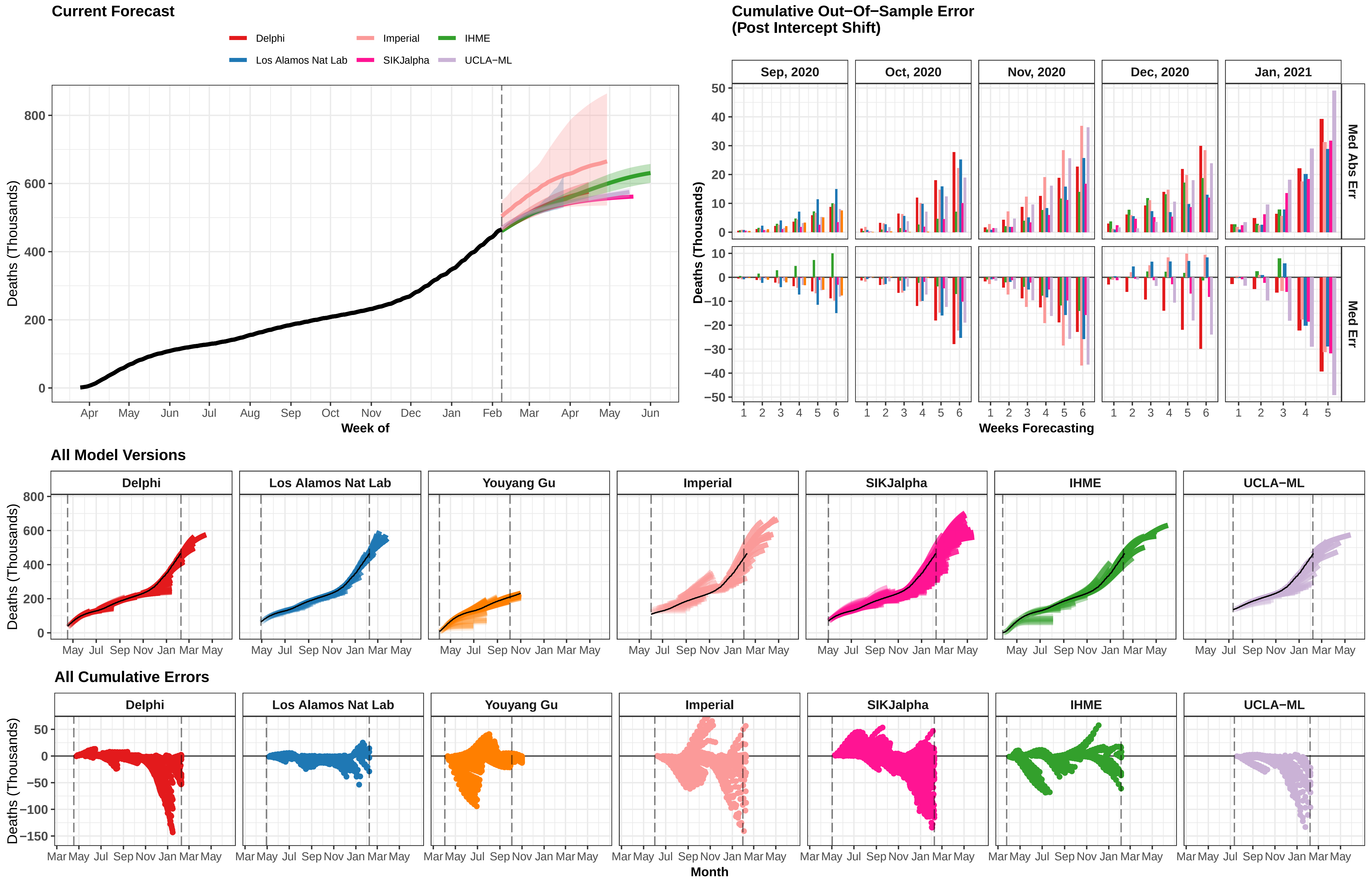

Brazil

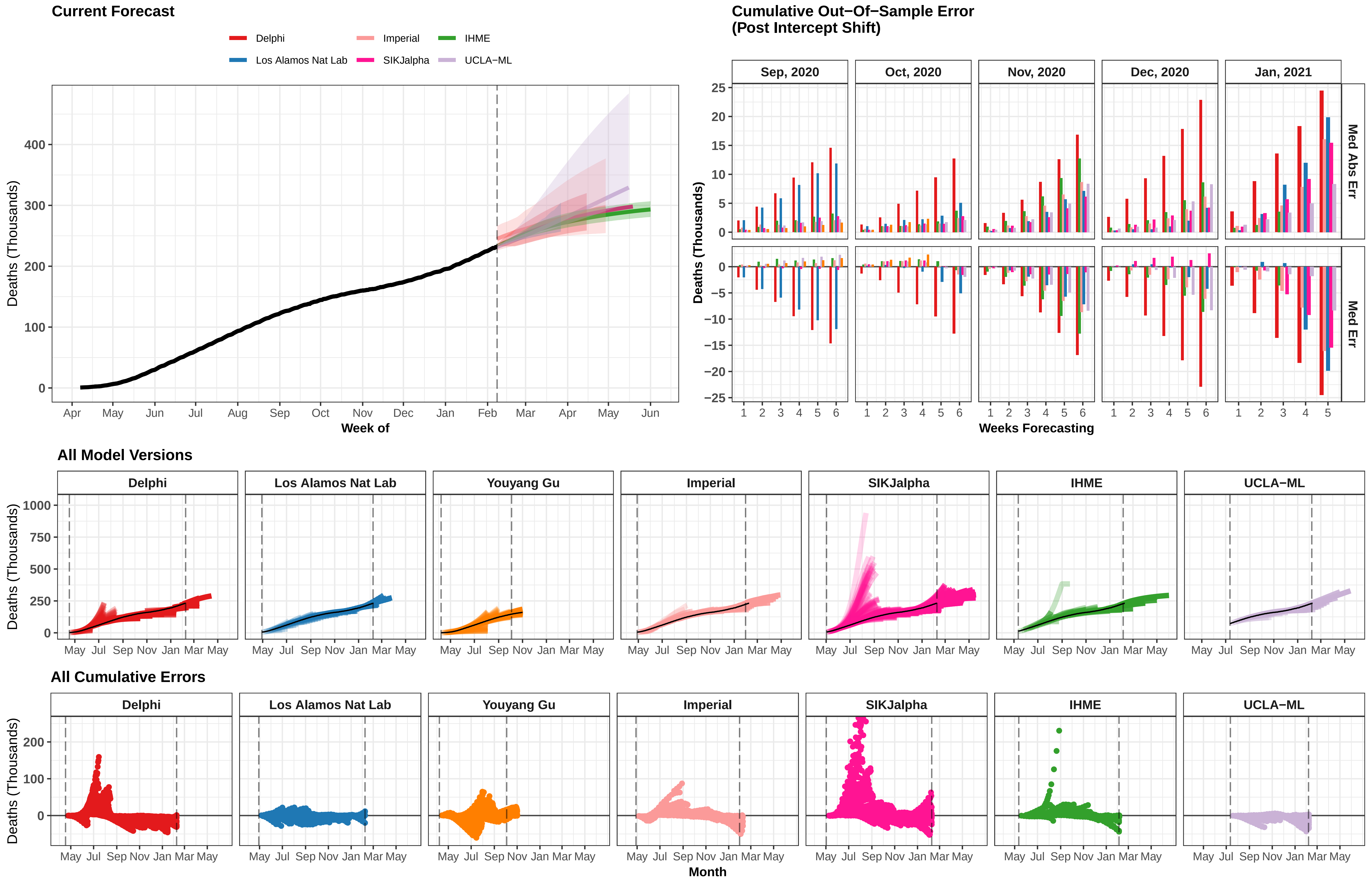
Current Forecast
Delphi
Imperial
IHME
Los Alamos Nat Lab
SIKJalpha
UCLA−ML
Deaths (Thousands)
0
100
200
300
400
Apr
May
Jun
Jul
Aug
Sep
Oct
Nov
Dec
Jan
Feb
Mar
Apr
May
Jun
Week of
Cumulative Out−Of−Sample Error
(Post Intercept Shift)
Sep, 2020
Oct, 2020
Nov, 2020
Dec, 2020
Jan, 2021
Med Abs Err
Med Err
Deaths (Thousands)
25
20
15
10
5
0
−5
−10
−15
−20
−25
Weeks Forecasting
All Model Versions
Delphi
Los Alamos Nat Lab
Youyang Gu
Imperial
SIKJalpha
IHME
UCLA−ML
Deaths (Thousands)
1000
750
500
250
0
May
Jul
Sep
Nov
Jan
Mar
May
All Cumulative Errors
Delphi
Los Alamos Nat Lab
Youyang Gu
Imperial
SIKJalpha
IHME
UCLA−ML
Deaths (Thousands)
200
100
0
Month

Mexico

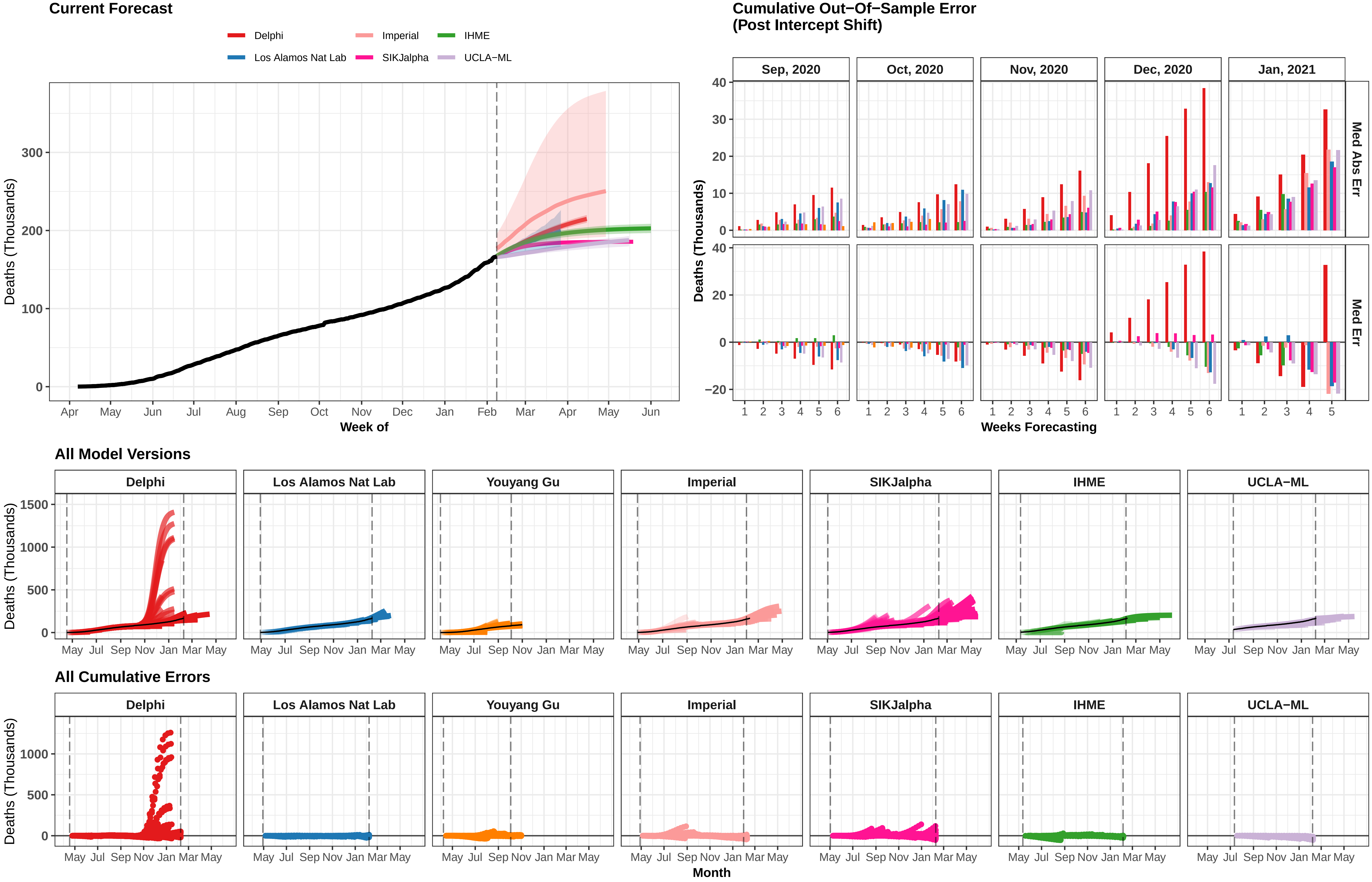
Current Forecast
Delphi
Imperial
IHME
Los Alamos Nat Lab
SIKJalpha
UCLA−ML
Deaths (Thousands)
Week of
Apr May Jun Jul Aug Sep Oct Nov Dec Jan Feb Mar Apr May Jun
Cumulative Out−Of−Sample Error
(Post Intercept Shift)
Sep, 2020
Oct, 2020
Nov, 2020
Dec, 2020
Jan, 2021
Med Abs Err
Med Err
Weeks Forecasting
All Model Versions
Delphi
Los Alamos Nat Lab
Youyang Gu
Imperial
SIKJalpha
IHME
UCLA−ML
All Cumulative Errors
Month
May Jul Sep Nov Jan Mar May

India

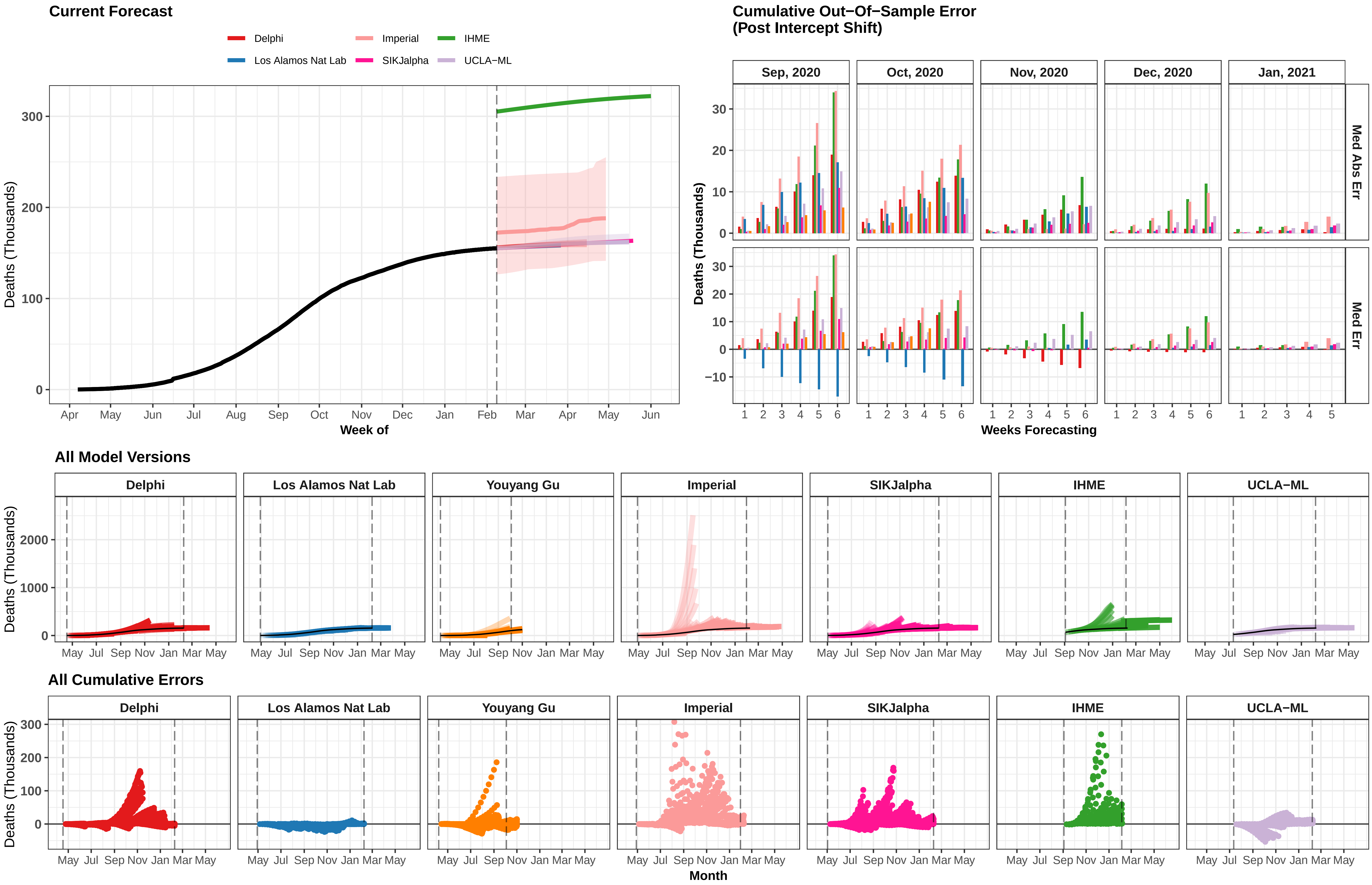
Current Forecast
Delphi
Imperial
IHME
Los Alamos Nat Lab
SIKJalpha
UCLA−ML
Deaths (Thousands)
Week of
Cumulative Out−Of−Sample Error
(Post Intercept Shift)
Sep, 2020
Oct, 2020
Nov, 2020
Dec, 2020
Jan, 2021
Med Abs Err
Med Err
Weeks Forecasting
All Model Versions
Delphi
Los Alamos Nat Lab
Youyang Gu
Imperial
SIKJalpha
IHME
UCLA−ML
All Cumulative Errors
Month

United Kingdom

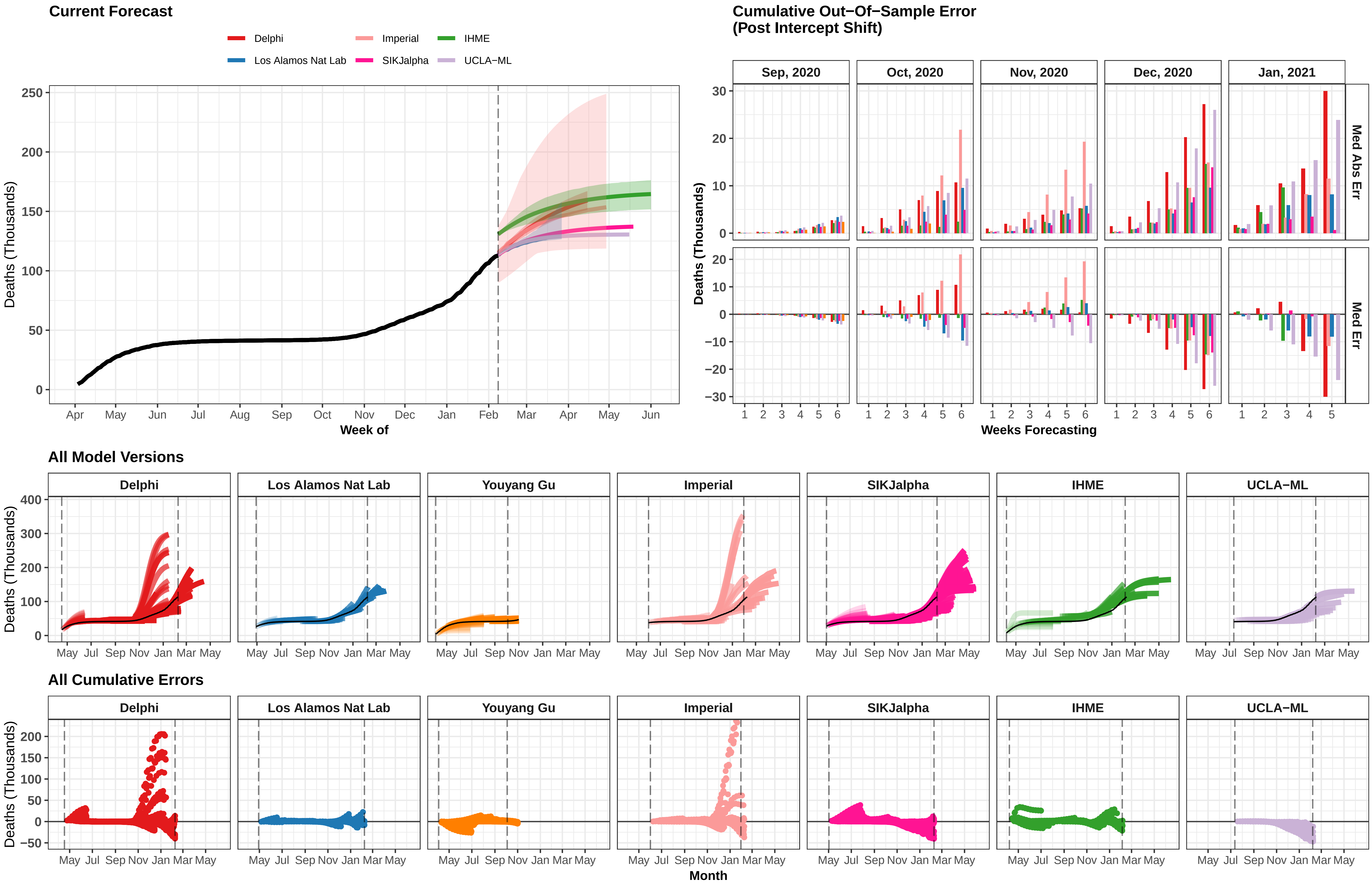

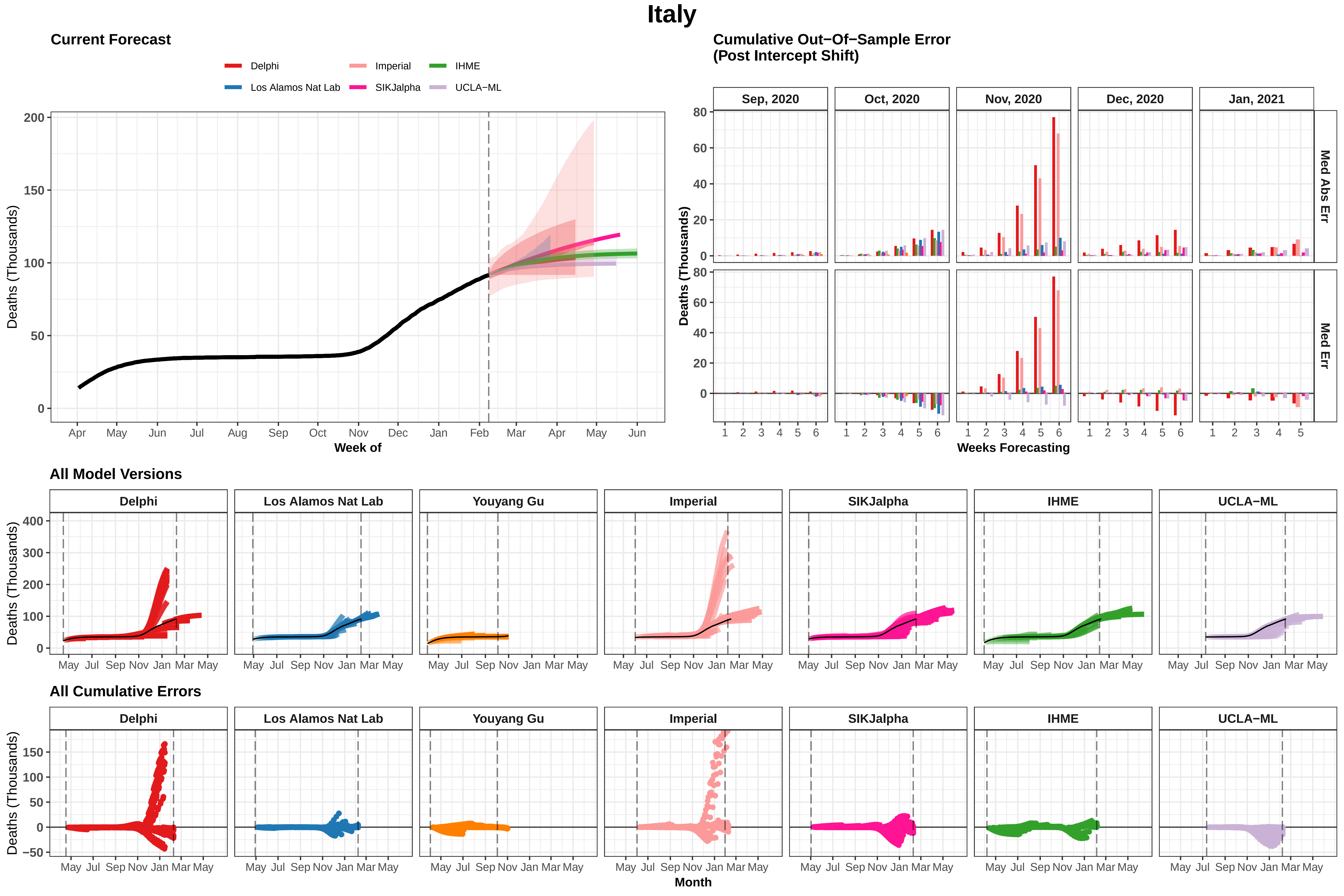
Italy
Current Forecast
Delphi
Imperial
IHME
Los Alamos Nat Lab
SIKJalpha
UCLA−ML
Deaths (Thousands)
Week of
Apr May Jun Jul Aug Sep Oct Nov Dec Jan Feb Mar Apr May Jun
Cumulative Out−Of−Sample Error
(Post Intercept Shift)
Sep, 2020
Oct, 2020
Nov, 2020
Dec, 2020
Jan, 2021
Med Abs Err
Med Err
Weeks Forecasting
All Model Versions
Delphi
Los Alamos Nat Lab
Youyang Gu
Imperial
SIKJalpha
IHME
UCLA−ML
All Cumulative Errors
Month

France

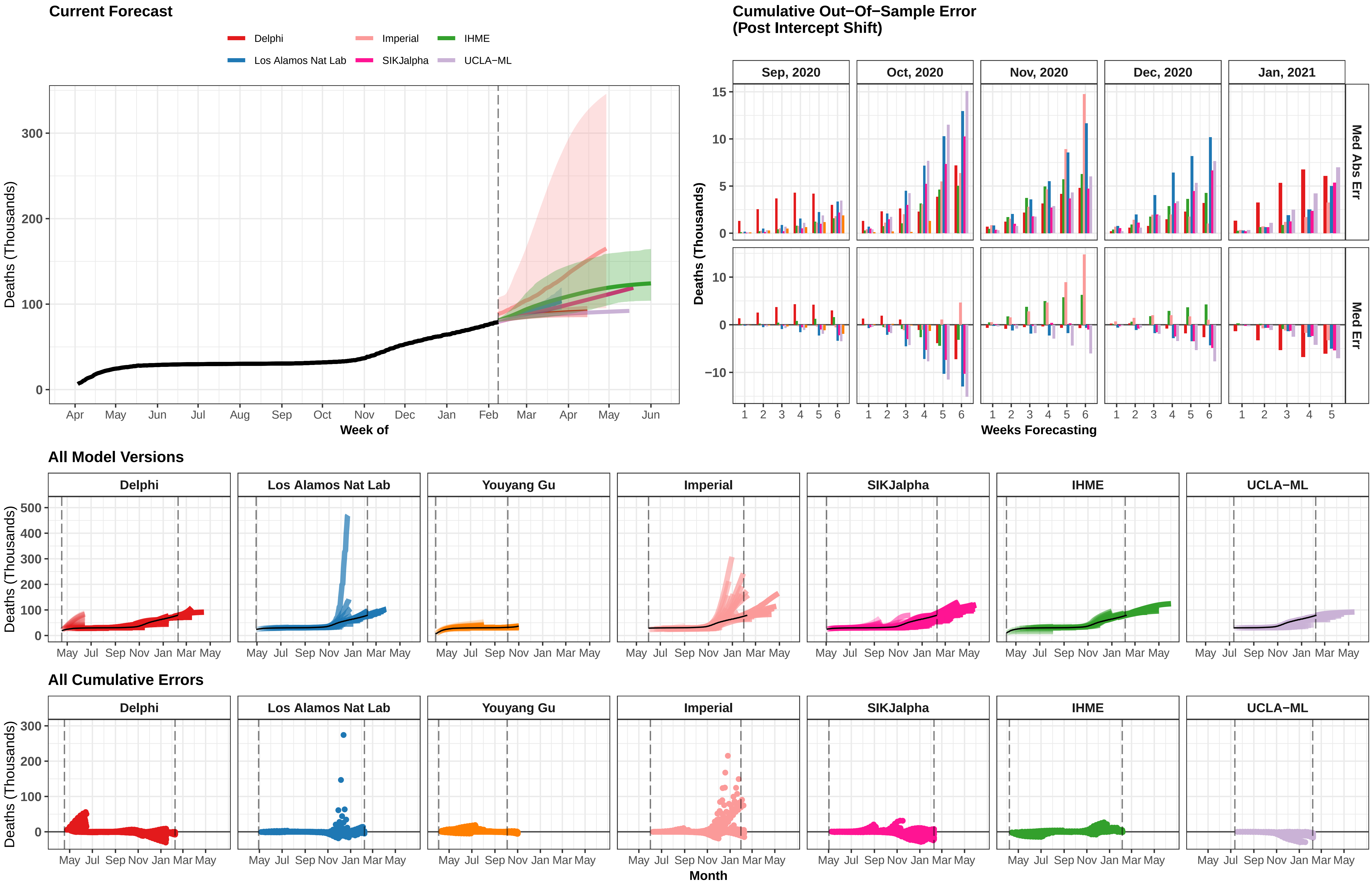
Current Forecast
Delphi
Imperial
IHME
Los Alamos Nat Lab
SIKJalpha
UCLA−ML
Deaths (Thousands)
Week of
Apr May Jun Jul Aug Sep Oct Nov Dec Jan Feb Mar Apr May Jun
Cumulative Out−Of−Sample Error
(Post Intercept Shift)
Sep, 2020
Oct, 2020
Nov, 2020
Dec, 2020
Jan, 2021
Med Abs Err
Med Err
Weeks Forecasting
All Model Versions
Delphi
Los Alamos Nat Lab
Youyang Gu
Imperial
SIKJalpha
IHME
UCLA−ML
All Cumulative Errors
Month

Russian Federation

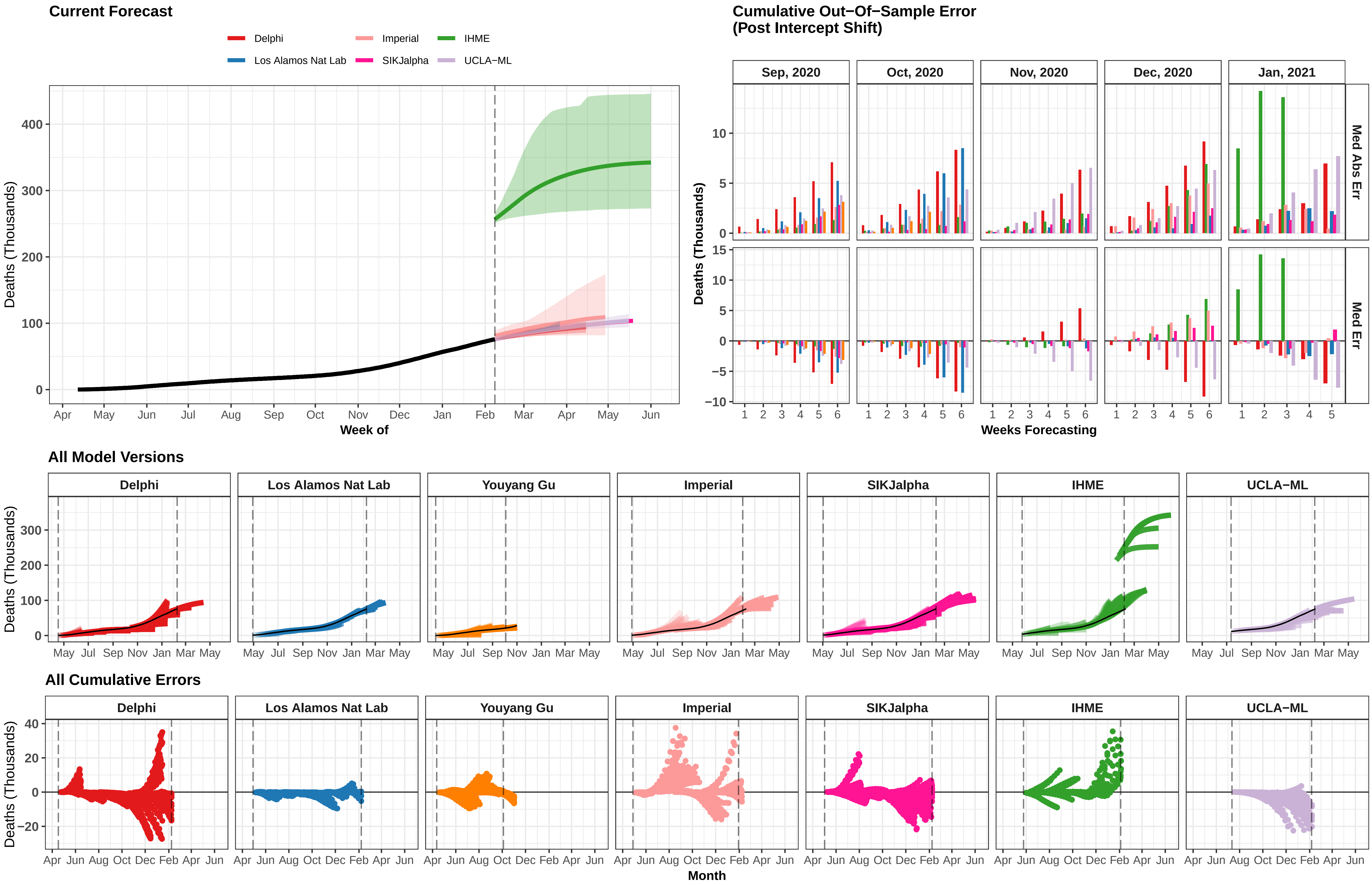

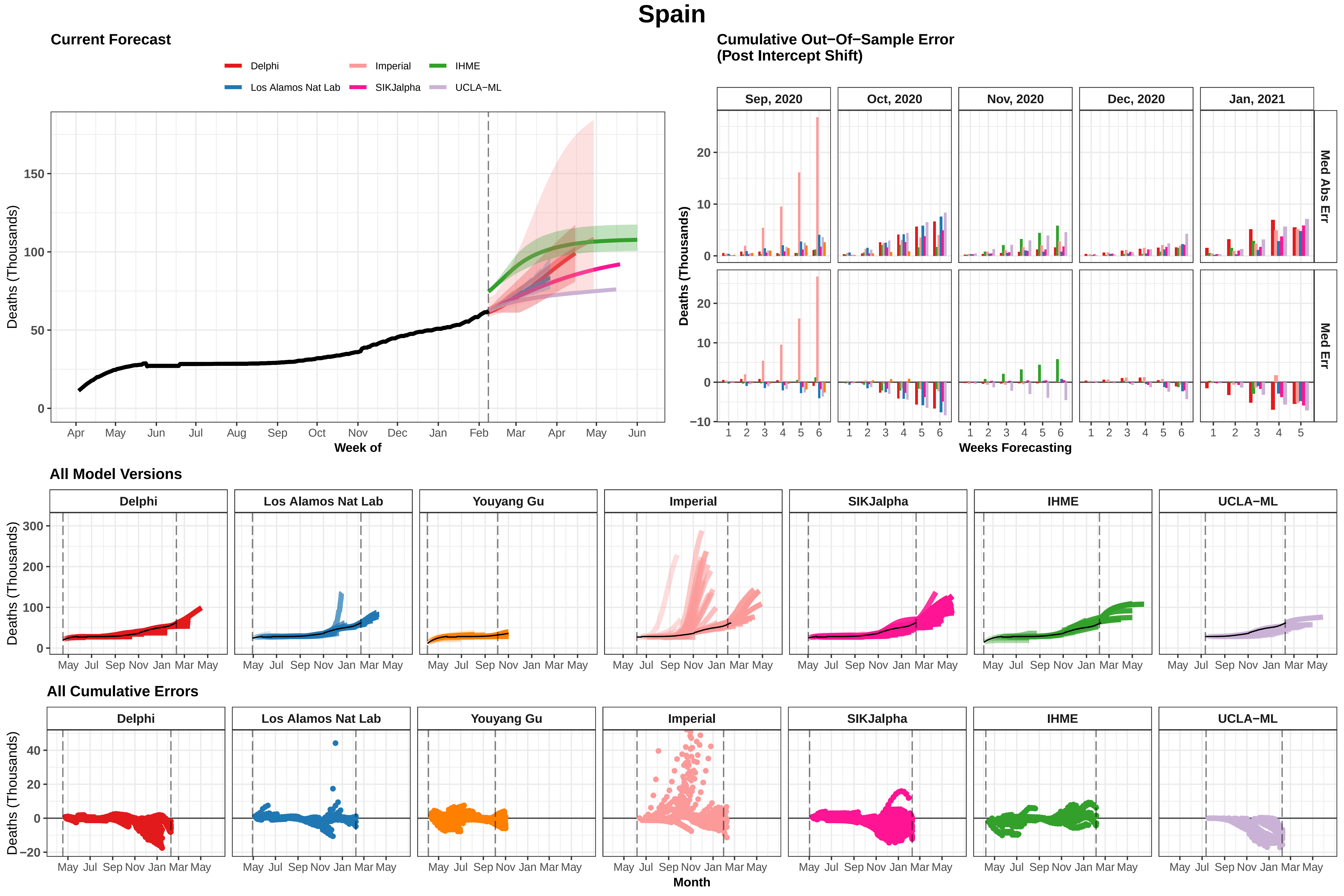
Spain
Current Forecast
Delphi
Imperial
IHME
Los Alamos Nat Lab
SIKJalpha
UCLA−ML
Deaths (Thousands)
Week of
Apr May Jun Jul Aug Sep Oct Nov Dec Jan Feb Mar Apr May Jun
Cumulative Out−Of−Sample Error
(Post Intercept Shift)
Sep, 2020
Oct, 2020
Nov, 2020
Dec, 2020
Jan, 2021
Med Abs Err
Med Err
Weeks Forecasting
All Model Versions
Delphi
Los Alamos Nat Lab
Youyang Gu
Imperial
SIKJalpha
IHME
UCLA−ML
All Cumulative Errors
Month

Germany

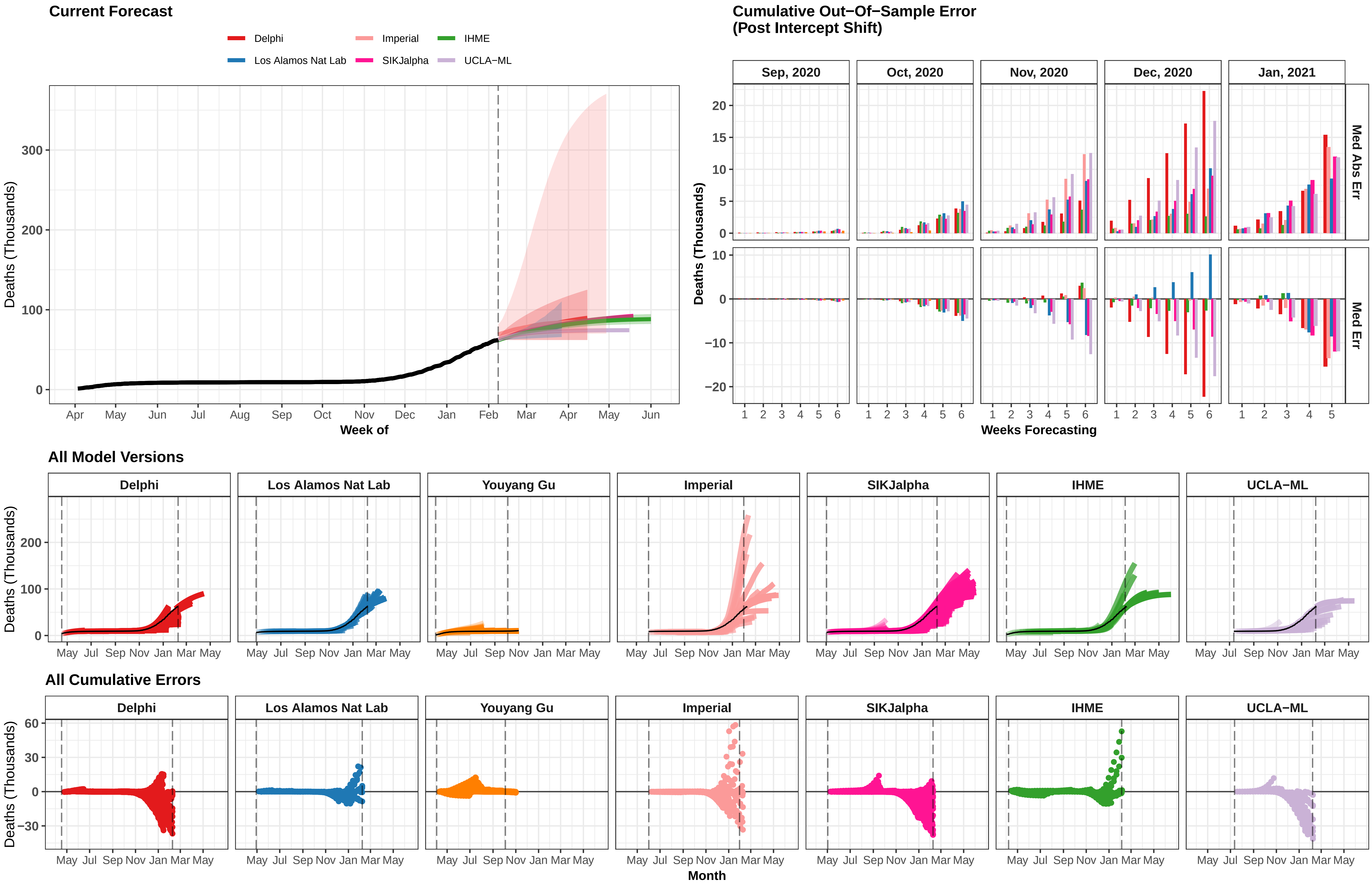

Current Forecast
Delphi
Imperial
IHME
Los Alamos Nat Lab
SIKJalpha
UCLA−ML
Deaths (Thousands)
Week of
Apr May Jun Jul Aug Sep Oct Nov Dec Jan Feb Mar Apr May Jun
Cumulative Out−Of−Sample Error
(Post Intercept Shift)
Sep, 2020
Oct, 2020
Nov, 2020
Dec, 2020
Jan, 2021
Med Abs Err
Med Err
Weeks Forecasting
All Model Versions
Delphi
Los Alamos Nat Lab
Youyang Gu
Imperial
SIKJalpha
IHME
UCLA−ML
May Jul Sep Nov Jan Mar May
All Cumulative Errors
Month

Iran

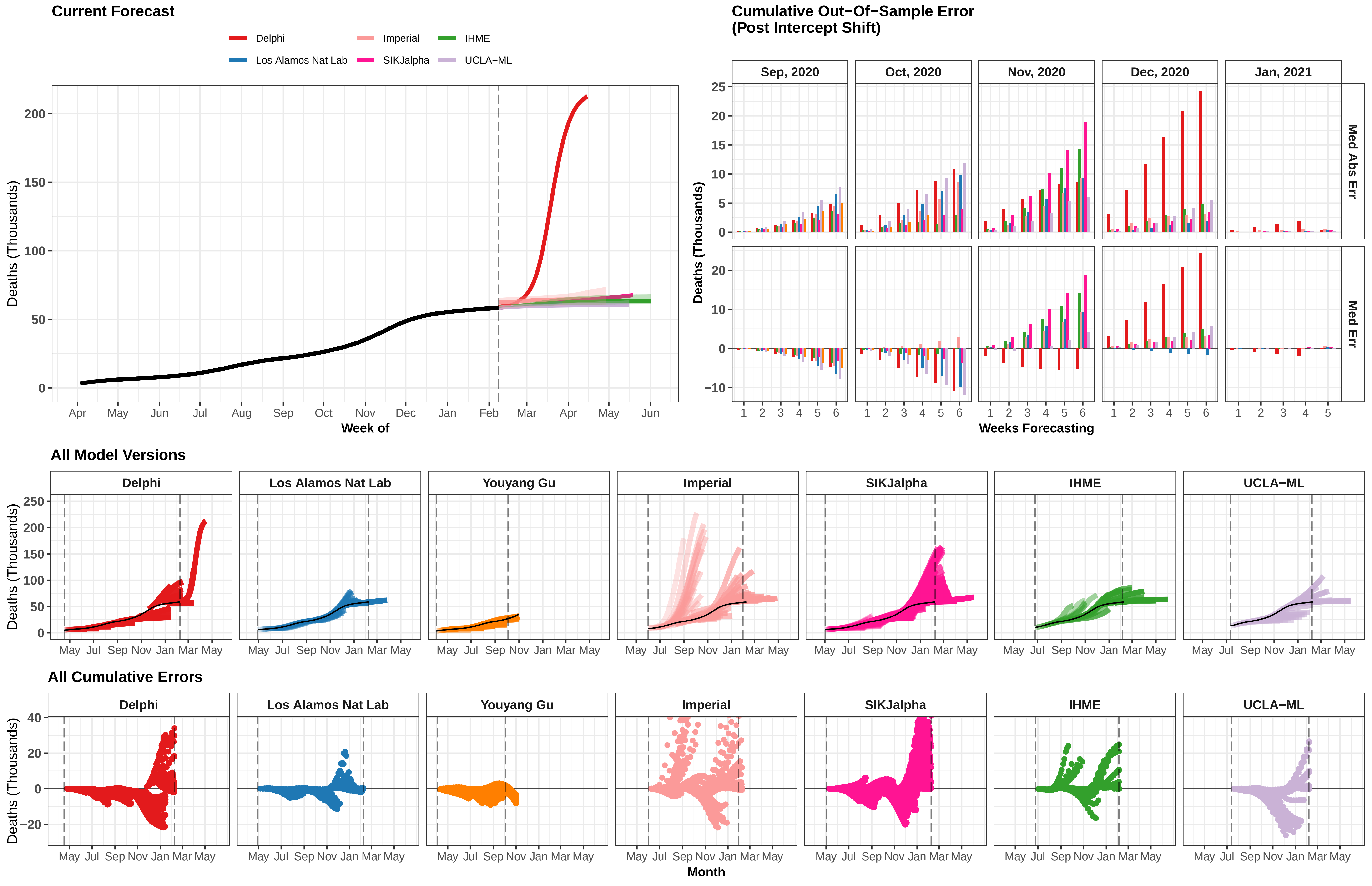

Current Forecast
Delphi
Imperial
IHME
Los Alamos Nat Lab
SIKJalpha
UCLA−ML
Deaths (Thousands)
Week of
Apr May Jun Jul Aug Sep Oct Nov Dec Jan Feb Mar Apr May Jun
Cumulative Out−Of−Sample Error
(Post Intercept Shift)
Sep, 2020
Oct, 2020
Nov, 2020
Dec, 2020
Jan, 2021
Med Abs Err
Med Err
Weeks Forecasting
All Model Versions
Delphi
Los Alamos Nat Lab
Youyang Gu
Imperial
SIKJalpha
IHME
UCLA−ML
All Cumulative Errors
Month
May Jul Sep Nov Jan Mar May

Colombia

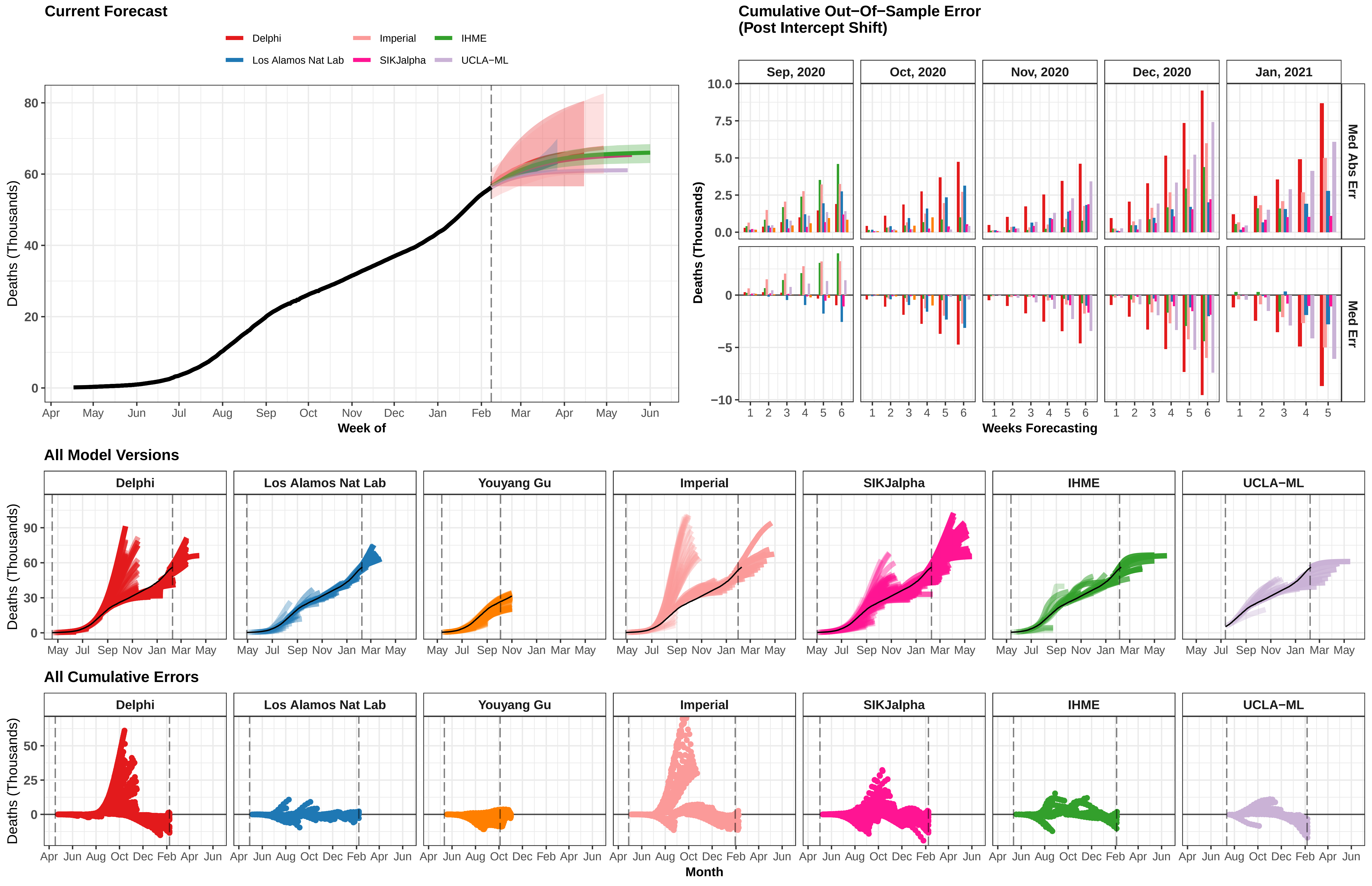
Current Forecast
Delphi
Imperial
IHME
Los Alamos Nat Lab
SIKJalpha
UCLA−ML
Deaths (Thousands)
Week of
Cumulative Out−Of−Sample Error
(Post Intercept Shift)
Sep, 2020
Oct, 2020
Nov, 2020
Dec, 2020
Jan, 2021
Med Abs Err
Med Err
Weeks Forecasting
All Model Versions
Delphi
Los Alamos Nat Lab
Youyang Gu
Imperial
SIKJalpha
IHME
UCLA−ML
All Cumulative Errors
Month

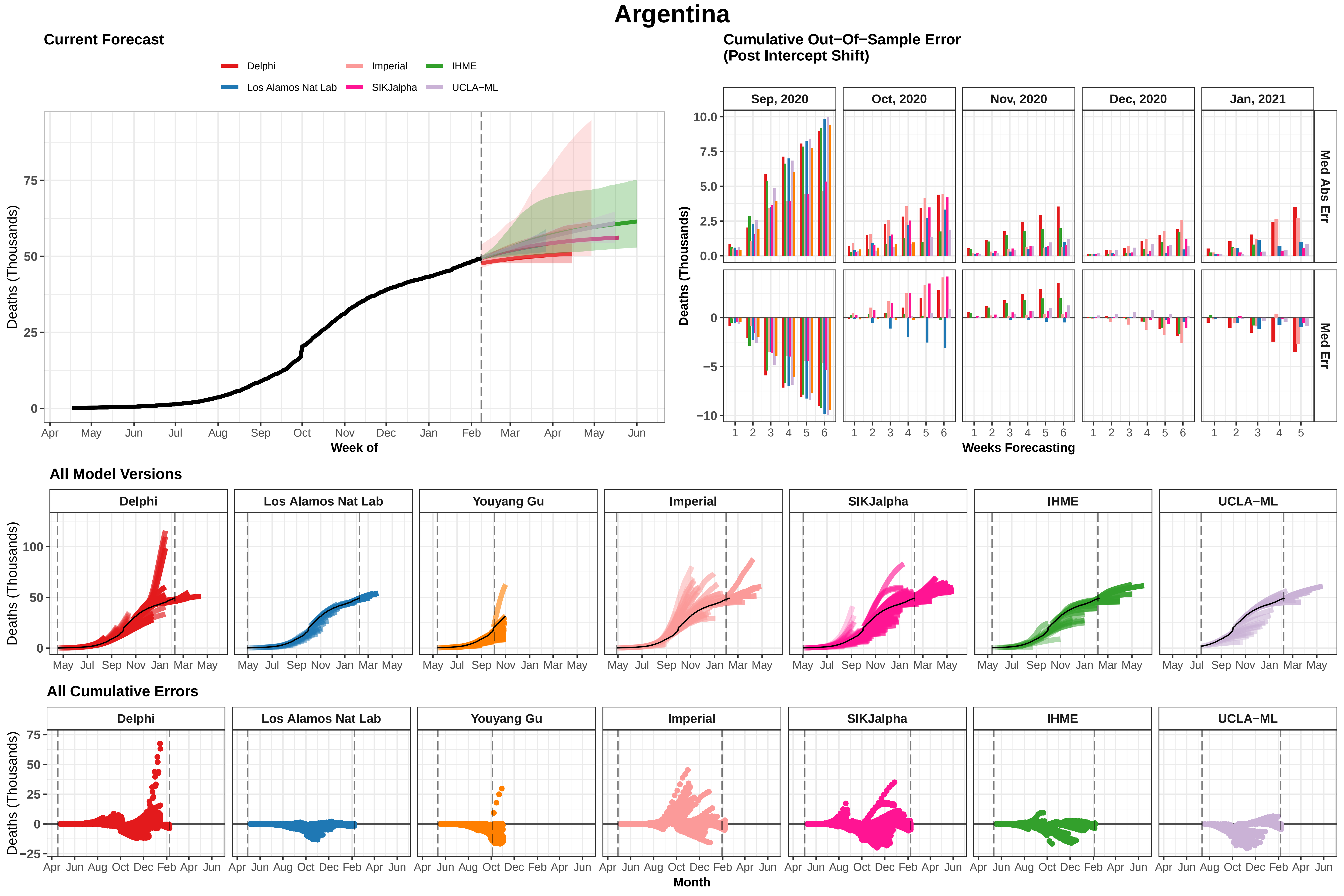

Argentina
Current Forecast
Delphi
Imperial
IHME
Los Alamos Nat Lab
SIKJalpha
UCLA−ML
Deaths (Thousands)
Week of
Cumulative Out−Of−Sample Error
(Post Intercept Shift)
Sep, 2020
Oct, 2020
Nov, 2020
Dec, 2020
Jan, 2021
Med Abs Err
Med Err
Weeks Forecasting
All Model Versions
Delphi
Los Alamos Nat Lab
Youyang Gu
Imperial
SIKJalpha
IHME
UCLA−ML
All Cumulative Errors
Month

South Africa

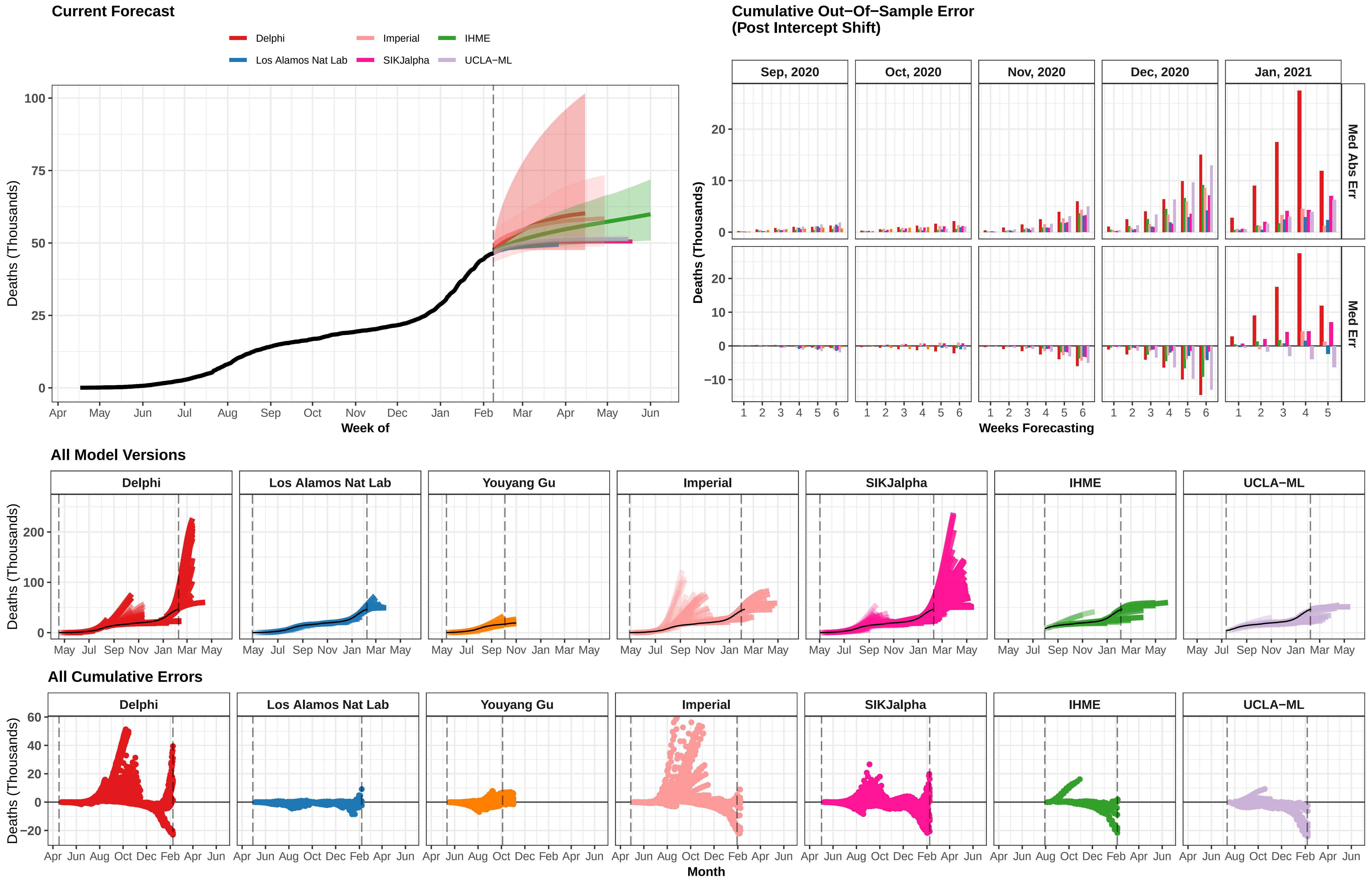

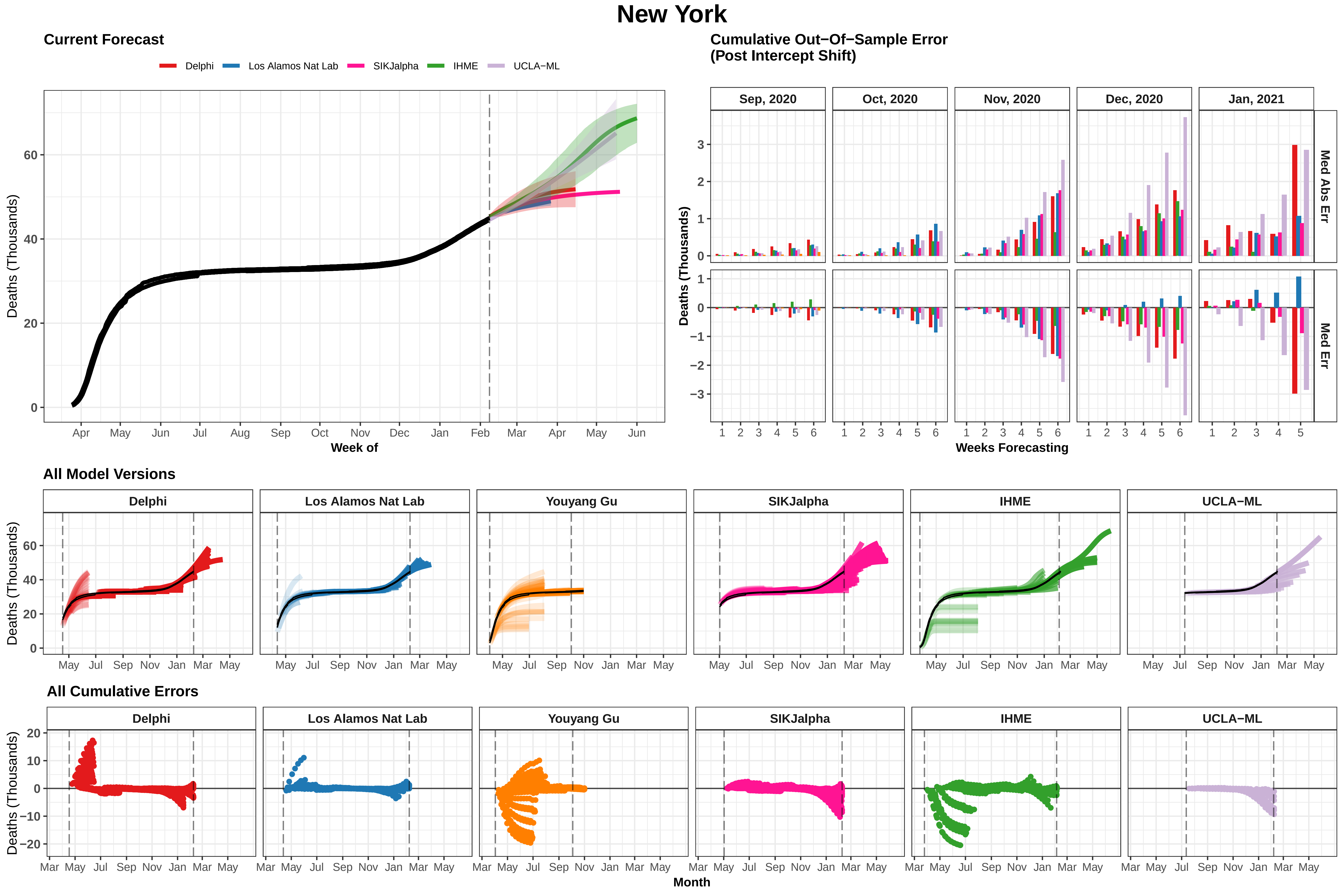
New York
Current Forecast
Delphi
Los Alamos Nat Lab
SIKJalpha
IHME
UCLA−ML
Deaths (Thousands)
Week of
Apr May Jun Jul Aug Sep Oct Nov Dec Jan Feb Mar Apr May Jun
Cumulative Out−Of−Sample Error
(Post Intercept Shift)
Sep, 2020
Oct, 2020
Nov, 2020
Dec, 2020
Jan, 2021
Med Abs Err
Med Err
Weeks Forecasting
All Model Versions
Delphi
Los Alamos Nat Lab
Youyang Gu
SIKJalpha
IHME
UCLA−ML
May Jul Sep Nov Jan Mar May
All Cumulative Errors
Mar May Jul Sep Nov Jan Mar May
Month

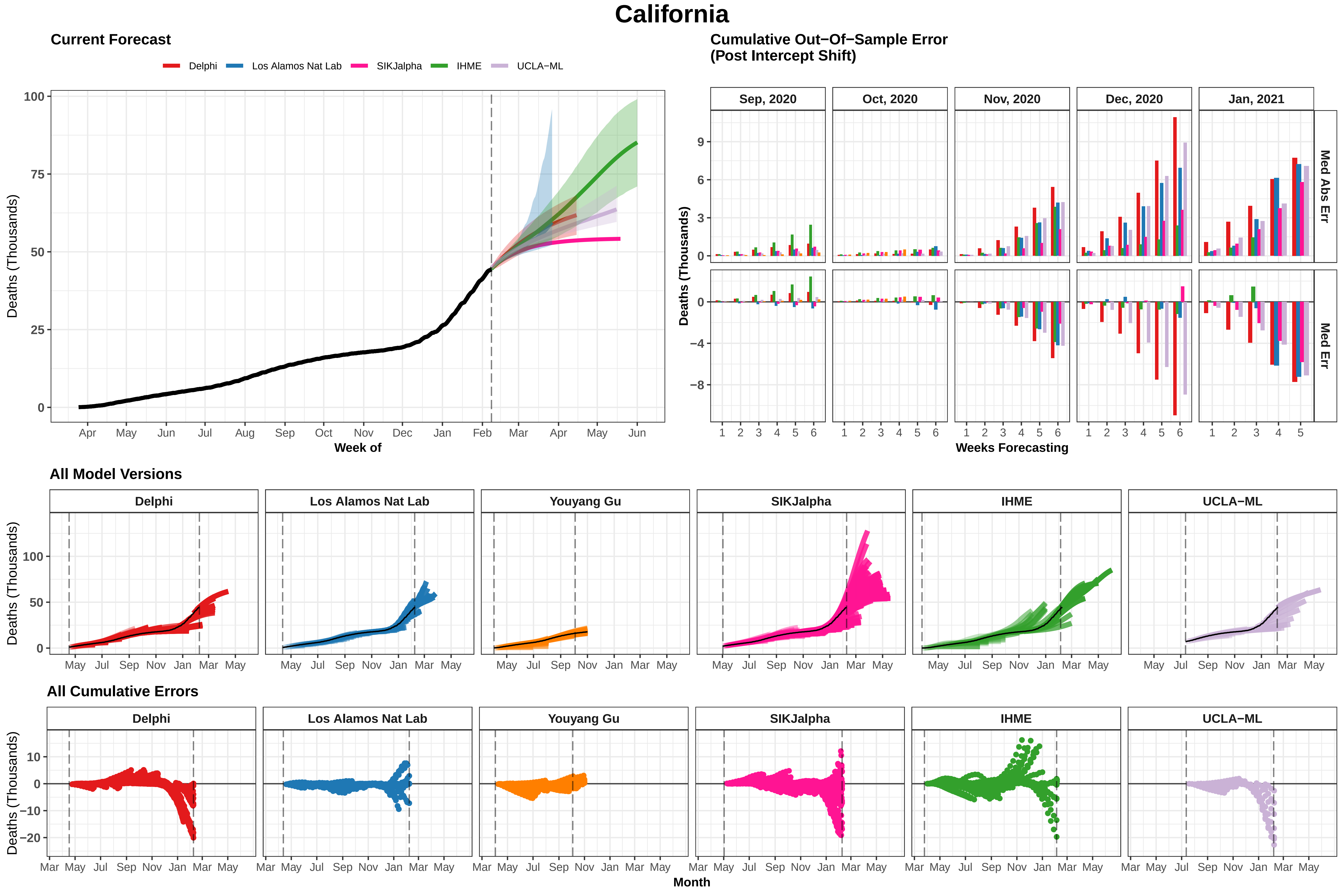
California
Current Forecast
Delphi
Los Alamos Nat Lab
SIKJalpha
IHME
UCLA−ML
Deaths (Thousands)
Week of
Apr May Jun Jul Aug Sep Oct Nov Dec Jan Feb Mar Apr May Jun
Cumulative Out−Of−Sample Error
(Post Intercept Shift)
Sep, 2020
Oct, 2020
Nov, 2020
Dec, 2020
Jan, 2021
Med Abs Err
Med Err
Weeks Forecasting
All Model Versions
Delphi
Los Alamos Nat Lab
Youyang Gu
SIKJalpha
IHME
UCLA−ML
All Cumulative Errors
Month
Mar May Jul Sep Nov Jan Mar May

Texas

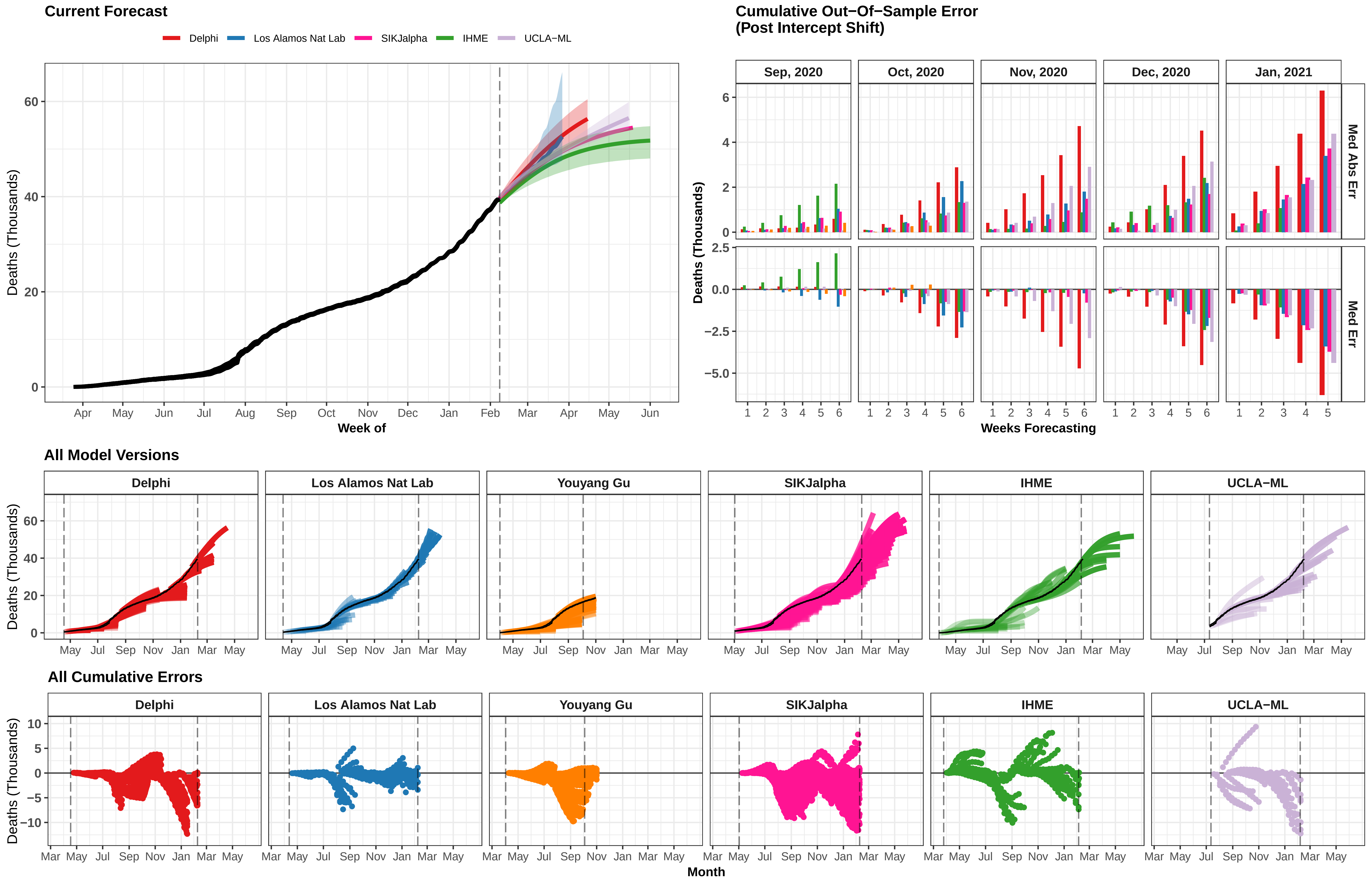

Current Forecast
Delphi
Los Alamos Nat Lab
SIKJalpha
IHME
UCLA−ML
Deaths (Thousands)
Week of
Apr May Jun Jul Aug Sep Oct Nov Dec Jan Feb Mar Apr May Jun
Cumulative Out−Of−Sample Error
(Post Intercept Shift)
Sep, 2020
Oct, 2020
Nov, 2020
Dec, 2020
Jan, 2021
Med Abs Err
Med Err
Weeks Forecasting
All Model Versions
Delphi
Los Alamos Nat Lab
Youyang Gu
SIKJalpha
IHME
UCLA−ML
All Cumulative Errors
Month

Poland

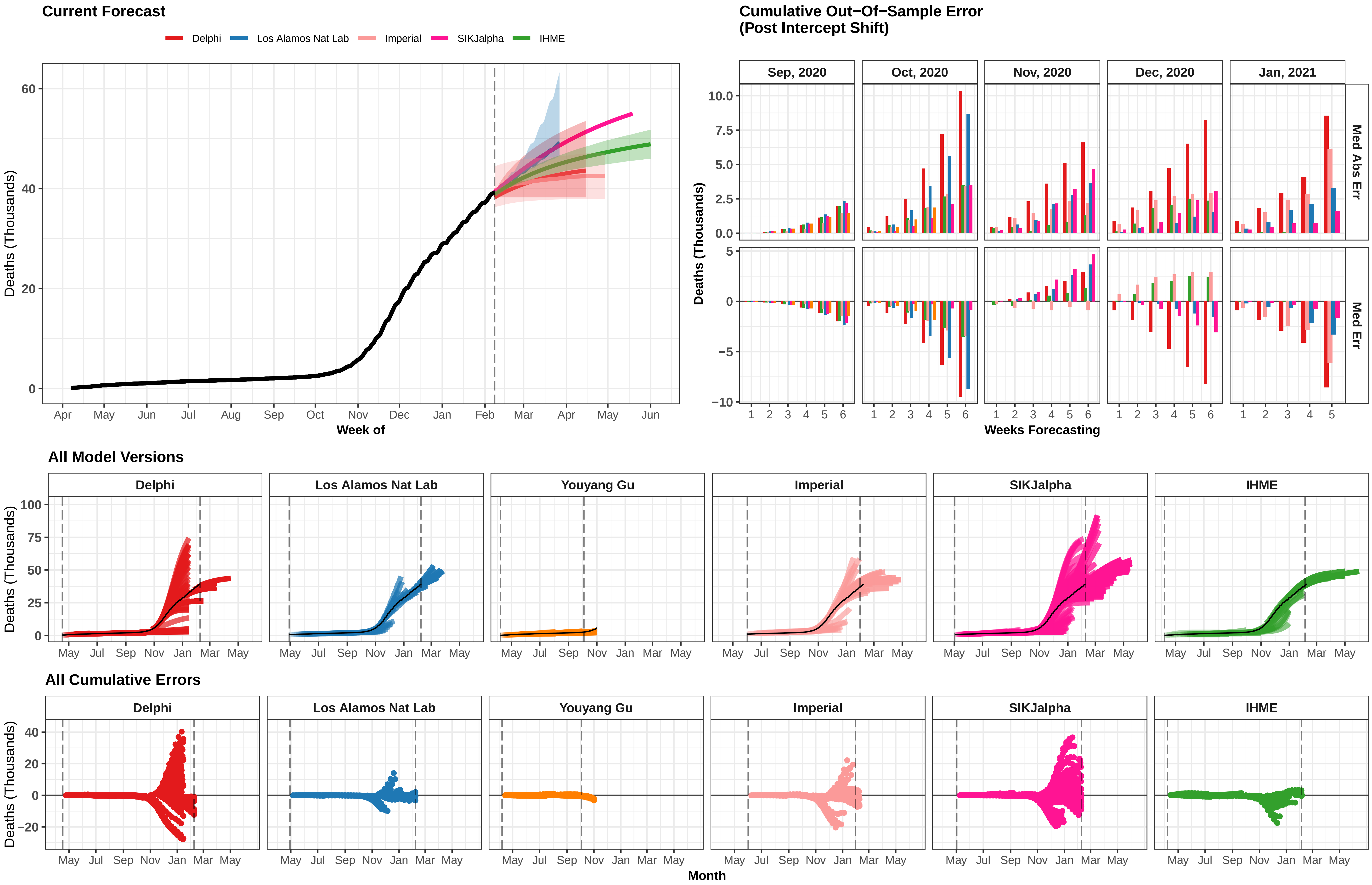
Current Forecast
Delphi
Los Alamos Nat Lab
Imperial
SIKJalpha
IHME
Deaths (Thousands)
0
20
40
60
Apr
May
Jun
Jul
Aug
Sep
Oct
Nov
Dec
Jan
Feb
Mar
Apr
May
Jun
Week of
Cumulative Out−Of−Sample Error
(Post Intercept Shift)
Sep, 2020
Oct, 2020
Nov, 2020
Dec, 2020
Jan, 2021
Med Abs Err
Med Err
Deaths (Thousands)
10.0
7.5
5.0
2.5
0.0
5
0
−5
−10
1
2
3
4
5
6
Weeks Forecasting
All Model Versions
Delphi
Los Alamos Nat Lab
Youyang Gu
Imperial
SIKJalpha
IHME
Deaths (Thousands)
100
75
50
25
0
May
Jul
Sep
Nov
Jan
Mar
May
All Cumulative Errors
Delphi
Los Alamos Nat Lab
Youyang Gu
Imperial
SIKJalpha
IHME
Deaths (Thousands)
40
20
0
−20
May
Jul
Sep
Nov
Jan
Mar
May
Month

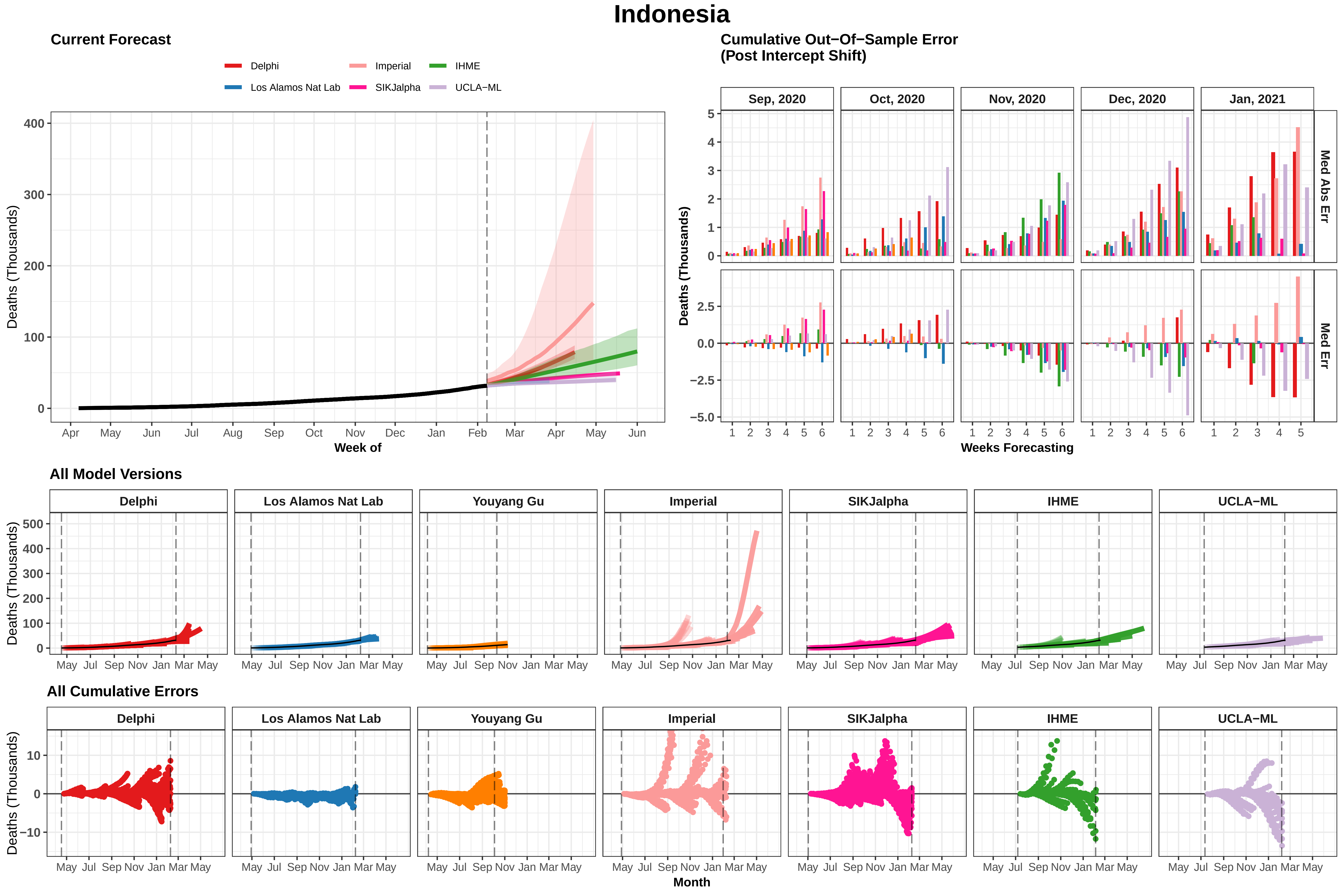

Indonesia
Current Forecast
Delphi
Imperial
IHME
Los Alamos Nat Lab
SIKJalpha
UCLA−ML
Deaths (Thousands)
0
100
200
300
400
Apr May Jun Jul Aug Sep Oct Nov Dec Jan Feb Mar Apr May Jun
Week of
Cumulative Out−Of−Sample Error
(Post Intercept Shift)
Sep, 2020
Oct, 2020
Nov, 2020
Dec, 2020
Jan, 2021
Med Abs Err
Med Err
Deaths (Thousands)
Weeks Forecasting
All Model Versions
Delphi
Los Alamos Nat Lab
Youyang Gu
Imperial
SIKJalpha
IHME
UCLA−ML
Deaths (Thousands)
May Jul Sep Nov Jan Mar May
All Cumulative Errors
Deaths (Thousands)
Month

Florida

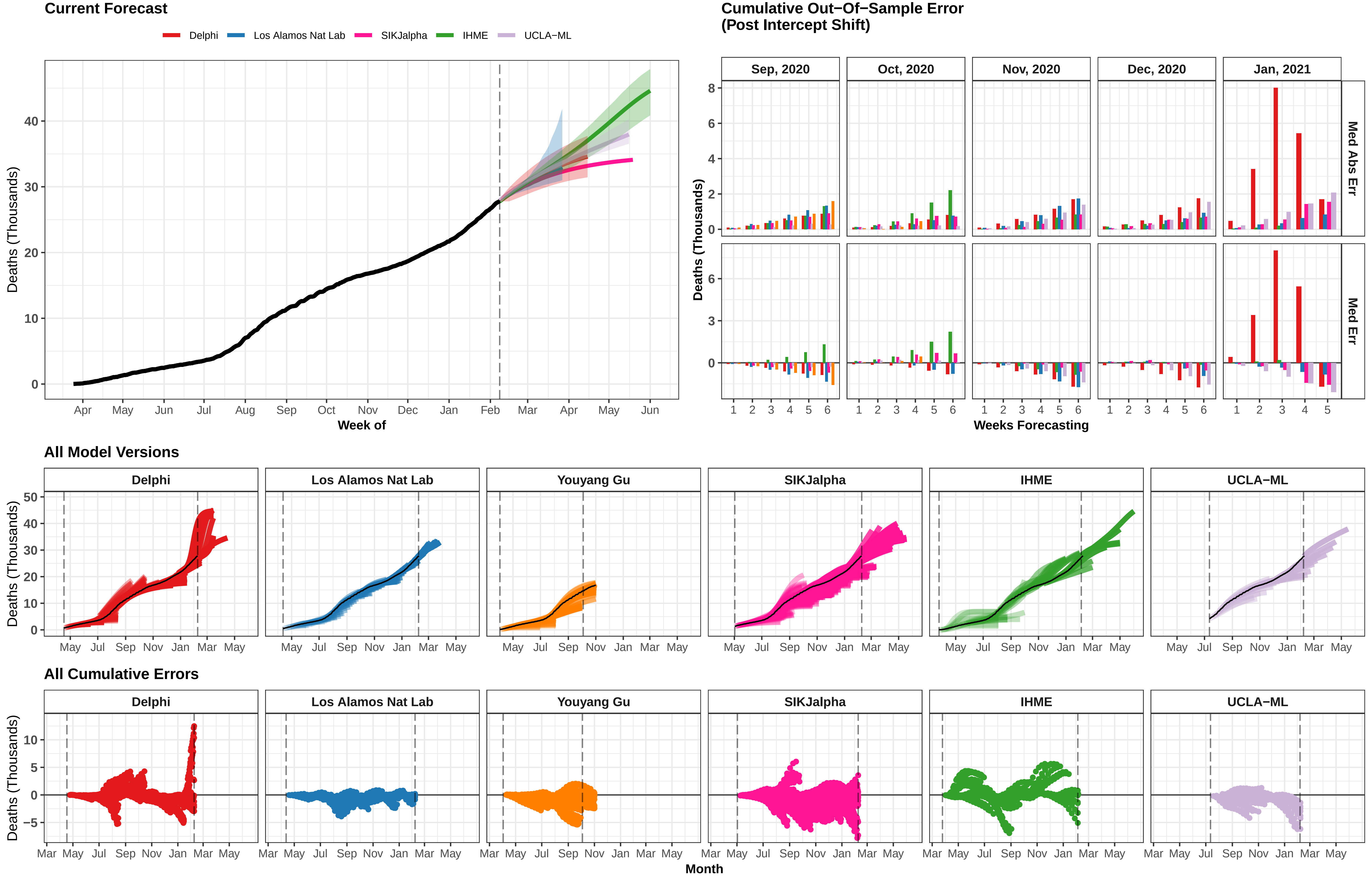
Current Forecast
Delphi
Los Alamos Nat Lab
SIKJalpha
IHME
UCLA−ML
Deaths (Thousands)
Week of
Apr May Jun Jul Aug Sep Oct Nov Dec Jan Feb Mar Apr May Jun
Cumulative Out−Of−Sample Error
(Post Intercept Shift)
Sep, 2020
Oct, 2020
Nov, 2020
Dec, 2020
Jan, 2021
Med Abs Err
Med Err
Weeks Forecasting
All Model Versions
Delphi
Los Alamos Nat Lab
Youyang Gu
SIKJalpha
IHME
UCLA−ML
All Cumulative Errors
Month
Mar May Jul Sep Nov Jan Mar May

Turkey

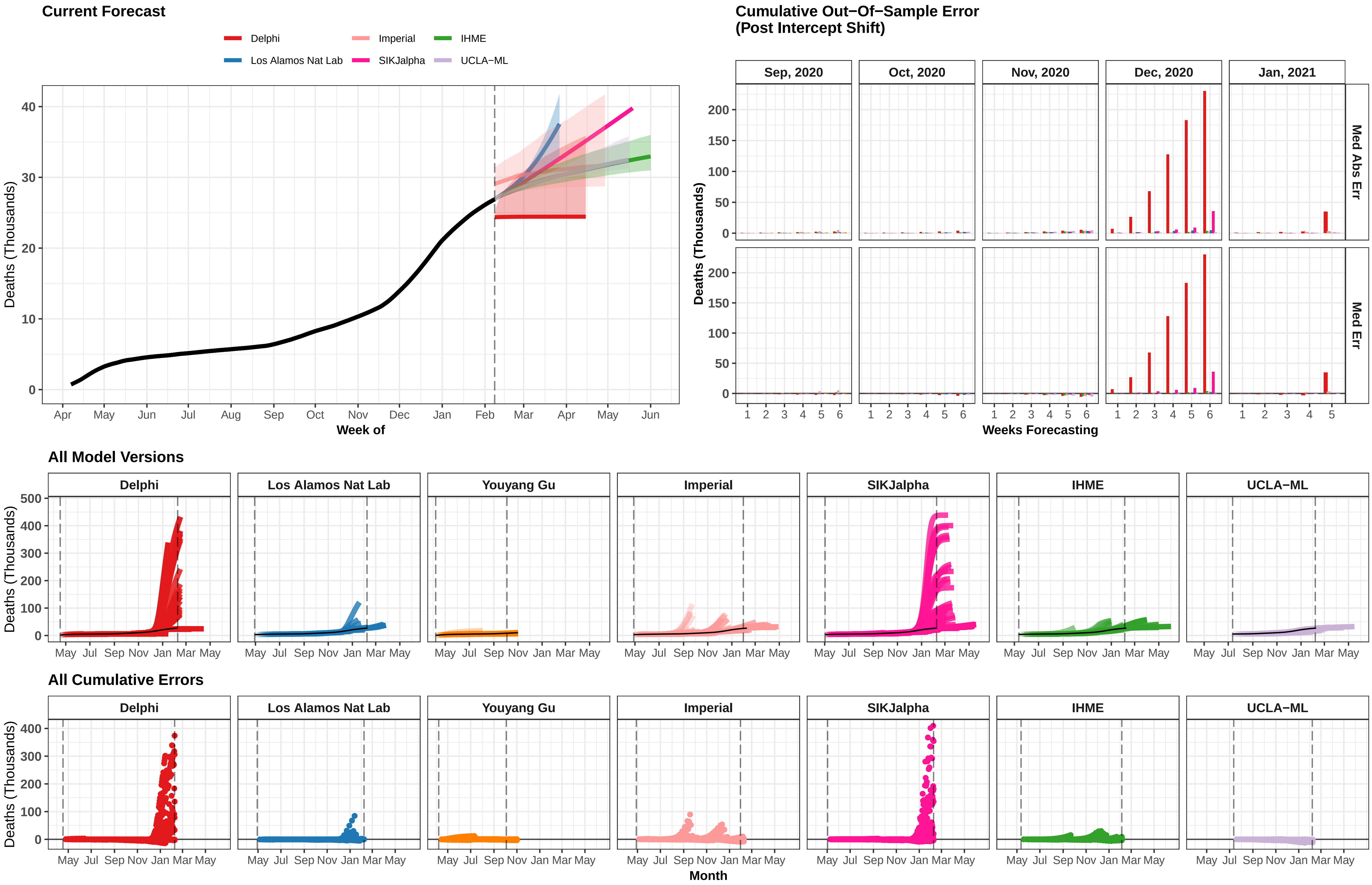
Current Forecast
Delphi
Imperial
IHME
Los Alamos Nat Lab
SIKJalpha
UCLA−ML
Deaths (Thousands)
Week of
Apr May Jun Jul Aug Sep Oct Nov Dec Jan Feb Mar Apr May Jun
Cumulative Out−Of−Sample Error
(Post Intercept Shift)
Sep, 2020
Oct, 2020
Nov, 2020
Dec, 2020
Jan, 2021
Med Abs Err
Med Err
Weeks Forecasting
All Model Versions
Delphi
Los Alamos Nat Lab
Youyang Gu
Imperial
SIKJalpha
IHME
UCLA−ML
May Jul Sep Nov Jan Mar May
All Cumulative Errors
Month

Ukraine

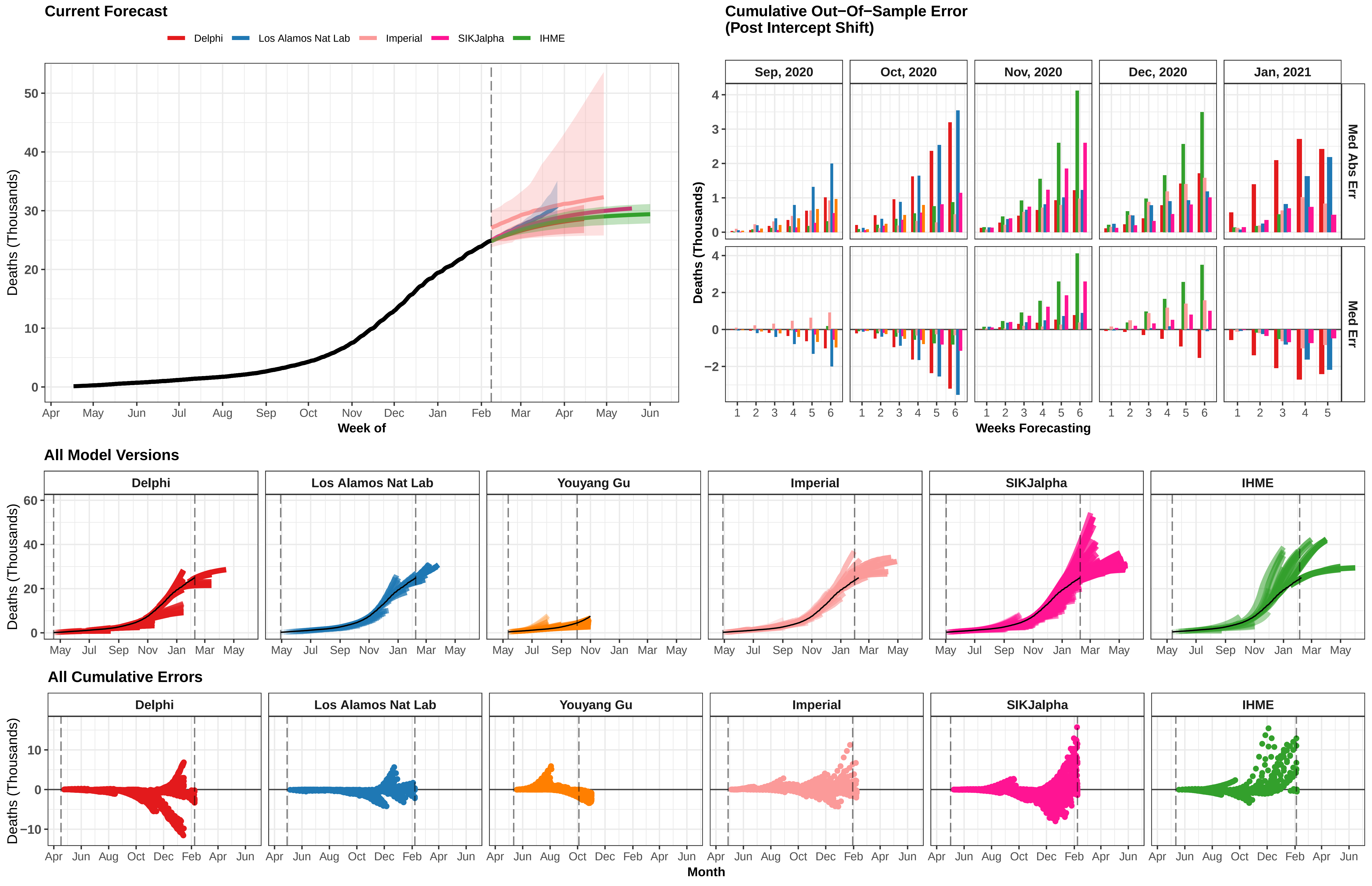

Current Forecast
Delphi
Los Alamos Nat Lab
Imperial
SIKJalpha
IHME
Deaths (Thousands)
Week of
Apr May Jun Jul Aug Sep Oct Nov Dec Jan Feb Mar Apr May Jun
Cumulative Out−Of−Sample Error
(Post Intercept Shift)
Sep, 2020
Oct, 2020
Nov, 2020
Dec, 2020
Jan, 2021
Med Abs Err
Med Err
Weeks Forecasting
All Model Versions
Delphi
Los Alamos Nat Lab
Youyang Gu
Imperial
SIKJalpha
IHME
All Cumulative Errors
Month

Pennsylvania

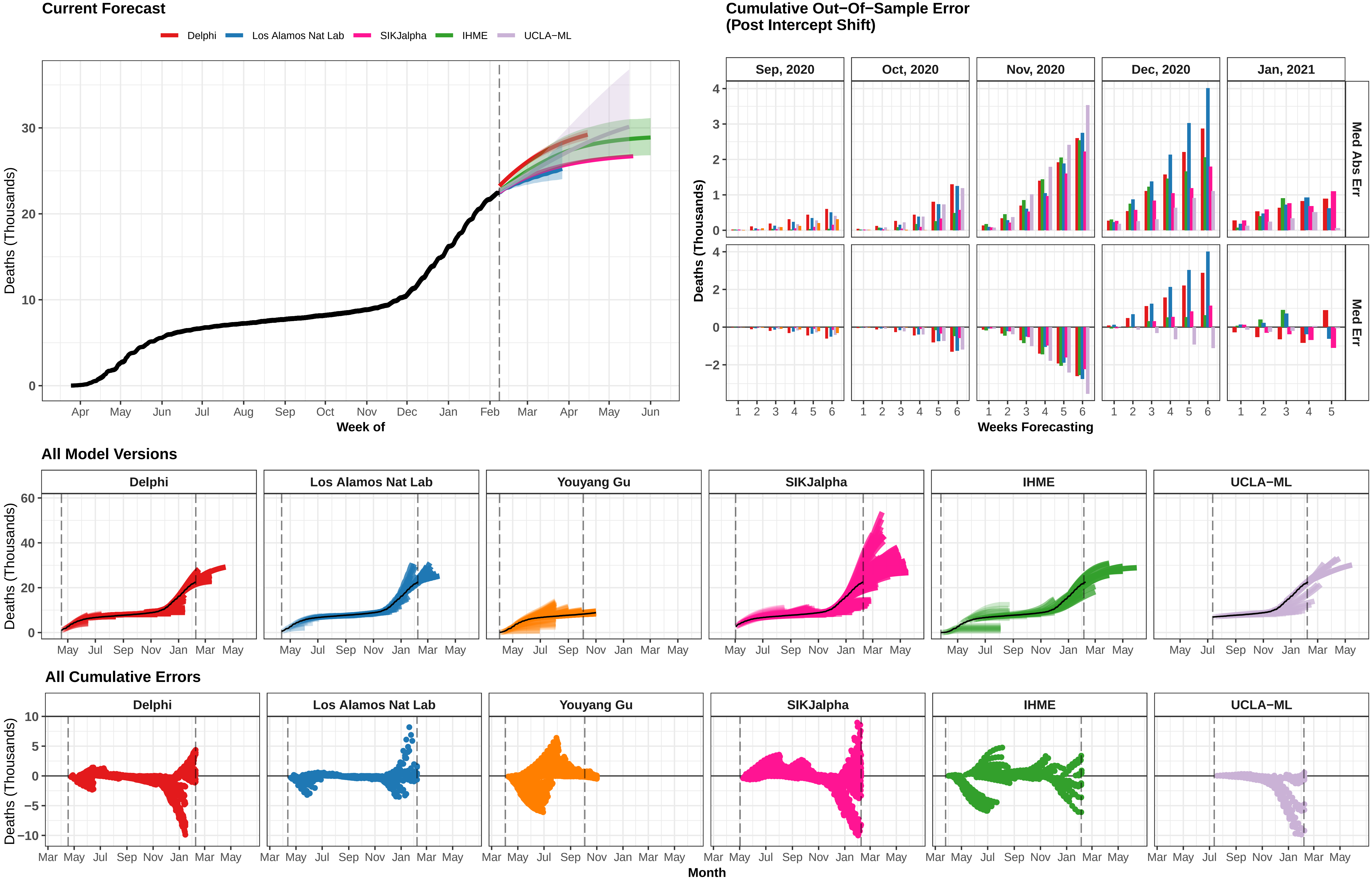

New Jersey

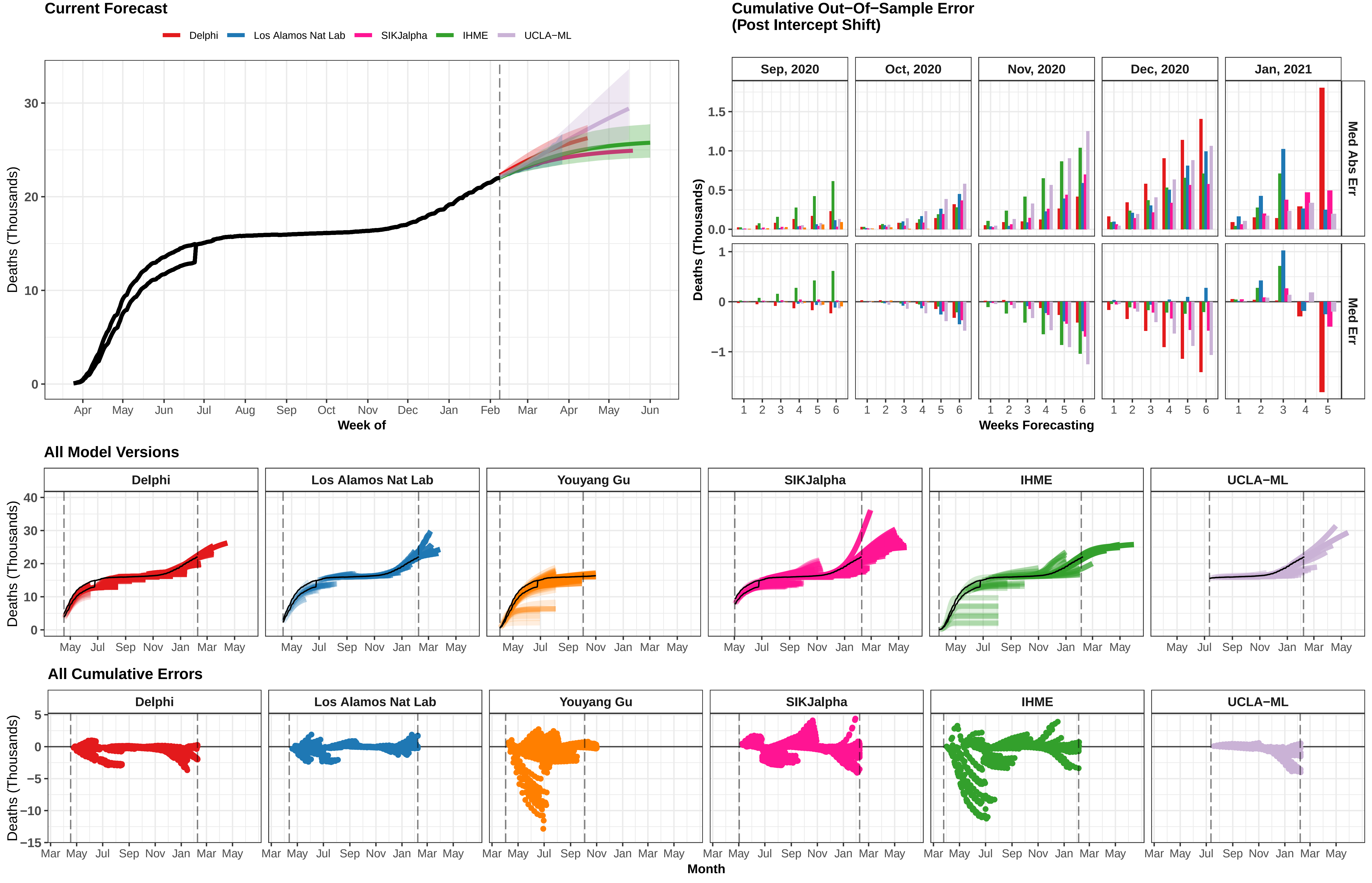

Current Forecast
Delphi
Los Alamos Nat Lab
SIKJalpha
IHME
UCLA−ML
Deaths (Thousands)
Week of
Apr May Jun Jul Aug Sep Oct Nov Dec Jan Feb Mar Apr May Jun
Cumulative Out−Of−Sample Error
(Post Intercept Shift)
Sep, 2020
Oct, 2020
Nov, 2020
Dec, 2020
Jan, 2021
Med Abs Err
Med Err
Weeks Forecasting
All Model Versions
Delphi
Los Alamos Nat Lab
Youyang Gu
SIKJalpha
IHME
UCLA−ML
Deaths (Thousands)
All Cumulative Errors
Delphi
Los Alamos Nat Lab
Youyang Gu
SIKJalpha
IHME
UCLA−ML
Deaths (Thousands)
Month

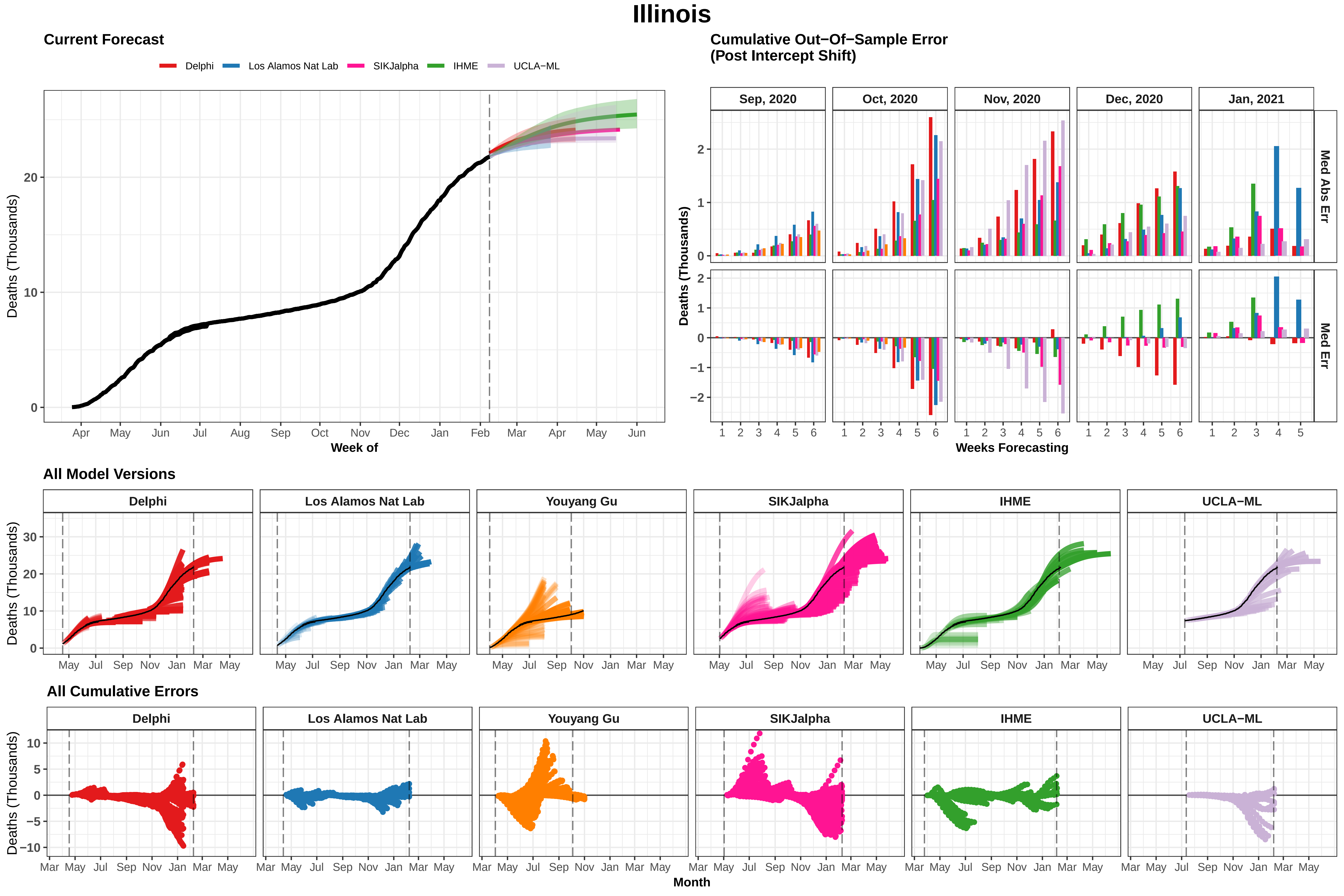

Illinois
Current Forecast
Delphi
Los Alamos Nat Lab
SIKJalpha
IHME
UCLA−ML
Deaths (Thousands)
Week of
Apr May Jun Jul Aug Sep Oct Nov Dec Jan Feb Mar Apr May Jun
Cumulative Out−Of−Sample Error (Post Intercept Shift)
Sep, 2020
Oct, 2020
Nov, 2020
Dec, 2020
Jan, 2021
Med Abs Err
Med Err
Weeks Forecasting
All Model Versions
Delphi
Los Alamos Nat Lab
Youyang Gu
SIKJalpha
IHME
UCLA−ML
All Cumulative Errors
Month

Belgium

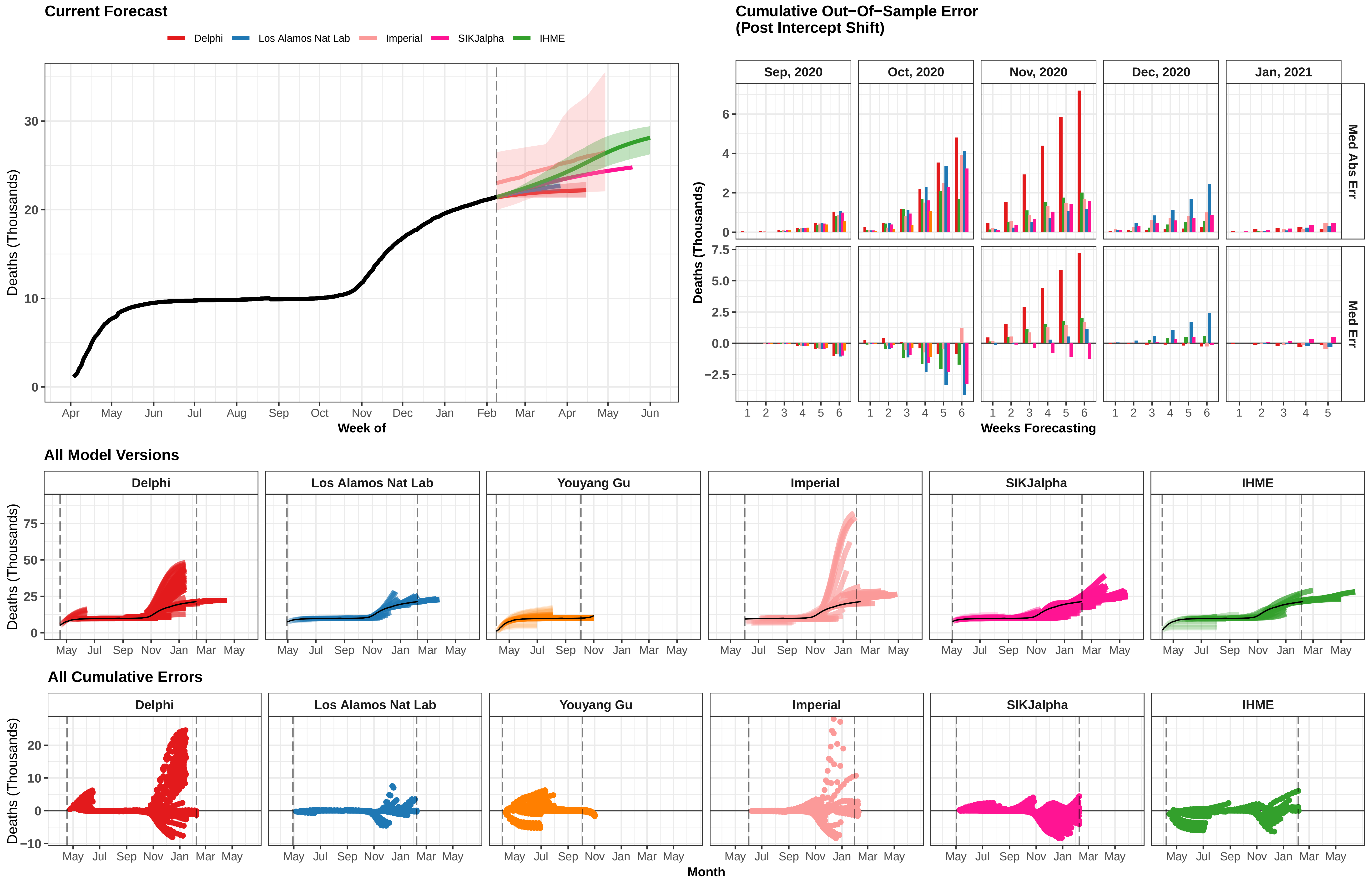
Current Forecast
Delphi
Los Alamos Nat Lab
Imperial
SIKJalpha
IHME
Deaths (Thousands)
Week of
Apr May Jun Jul Aug Sep Oct Nov Dec Jan Feb Mar Apr May Jun
Cumulative Out−Of−Sample Error
(Post Intercept Shift)
Sep, 2020
Oct, 2020
Nov, 2020
Dec, 2020
Jan, 2021
Med Abs Err
Med Err
Weeks Forecasting
All Model Versions
Delphi
Los Alamos Nat Lab
Youyang Gu
Imperial
SIKJalpha
IHME
Month
All Cumulative Errors

Canada

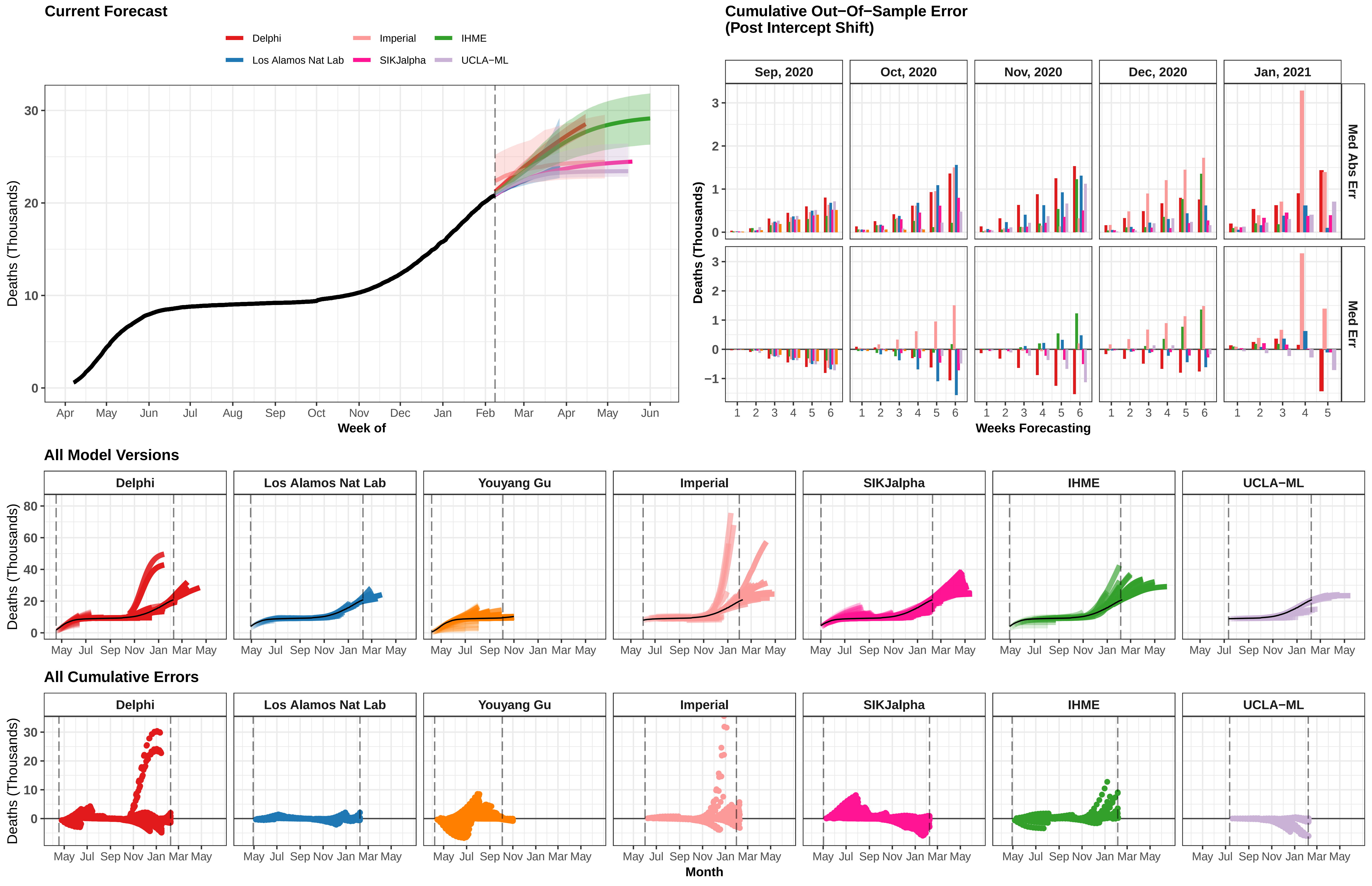
Current Forecast
Delphi
Imperial
IHME
Los Alamos Nat Lab
SIKJalpha
UCLA−ML
Deaths (Thousands)
Week of
Apr May Jun Jul Aug Sep Oct Nov Dec Jan Feb Mar Apr May Jun
Cumulative Out−Of−Sample Error
(Post Intercept Shift)
Sep, 2020
Oct, 2020
Nov, 2020
Dec, 2020
Jan, 2021
Med Abs Err
Med Err
Weeks Forecasting
All Model Versions
Delphi
Los Alamos Nat Lab
Youyang Gu
Imperial
SIKJalpha
IHME
UCLA−ML
All Cumulative Errors
Month
May Jul Sep Nov Jan Mar May

Chile

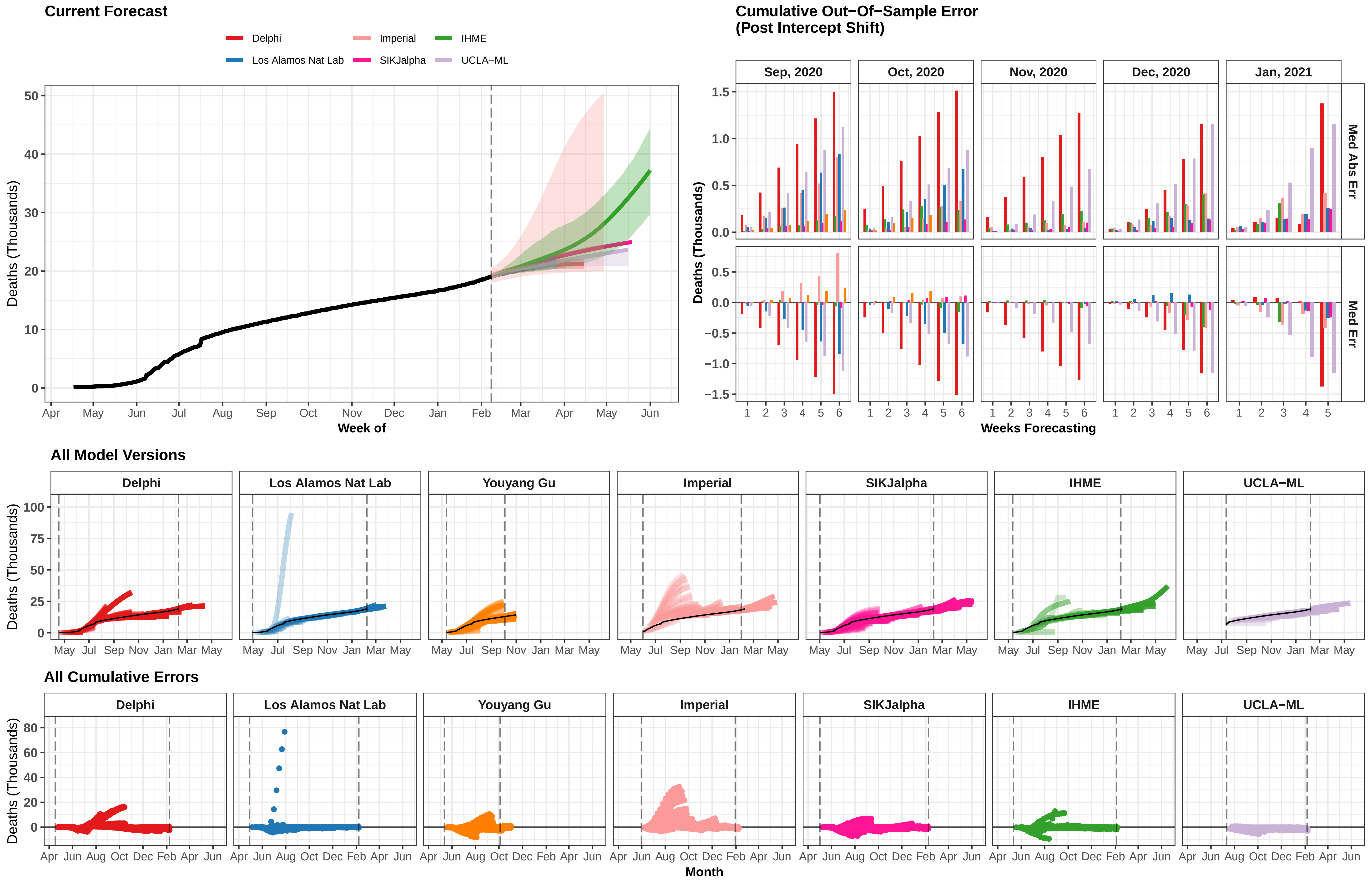

Current Forecast
Delphi
Imperial
IHME
Los Alamos Nat Lab
SIKJalpha
UCLA−ML
Deaths (Thousands)
Week of
Apr May Jun Jul Aug Sep Oct Nov Dec Jan Feb Mar Apr May Jun
Cumulative Out−Of−Sample Error
(Post Intercept Shift)
Sep, 2020
Oct, 2020
Nov, 2020
Dec, 2020
Jan, 2021
Med Abs Err
Med Err
Weeks Forecasting
All Model Versions
Delphi
Los Alamos Nat Lab
Youyang Gu
Imperial
SIKJalpha
IHME
UCLA−ML
All Cumulative Errors
Month

Romania

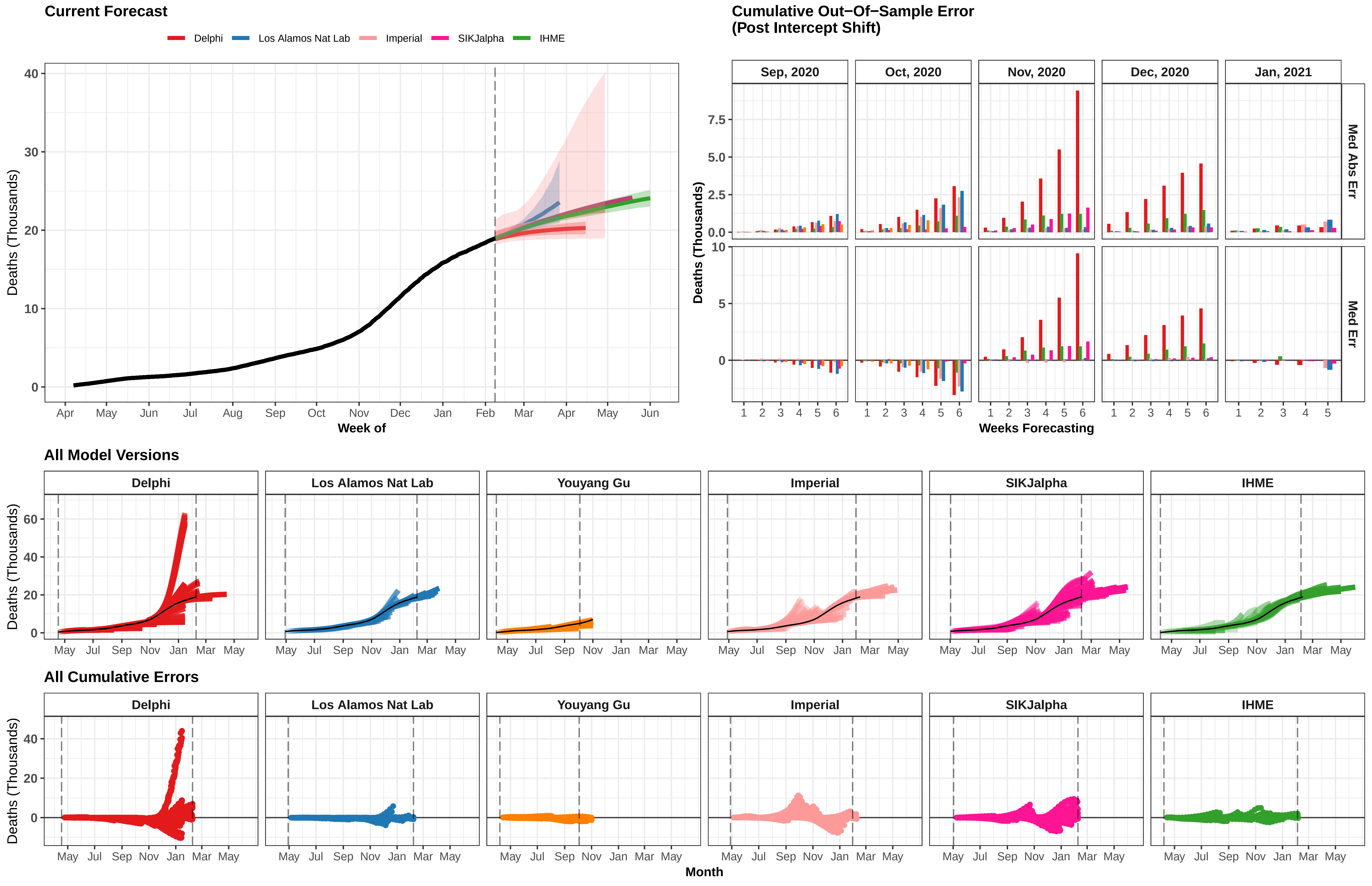

Current Forecast
Delphi
Los Alamos Nat Lab
Imperial
SIKJalpha
IHME
Deaths (Thousands)
Week of
Apr May Jun Jul Aug Sep Oct Nov Dec Jan Feb Mar Apr May Jun
Cumulative Out−Of−Sample Error
(Post Intercept Shift)
Sep, 2020
Oct, 2020
Nov, 2020
Dec, 2020
Jan, 2021
Med Abs Err
Med Err
Weeks Forecasting
All Model Versions
Delphi
Los Alamos Nat Lab
Youyang Gu
Imperial
SIKJalpha
IHME
All Cumulative Errors
Month
May Jul Sep Nov Jan Mar May

Czech Republic

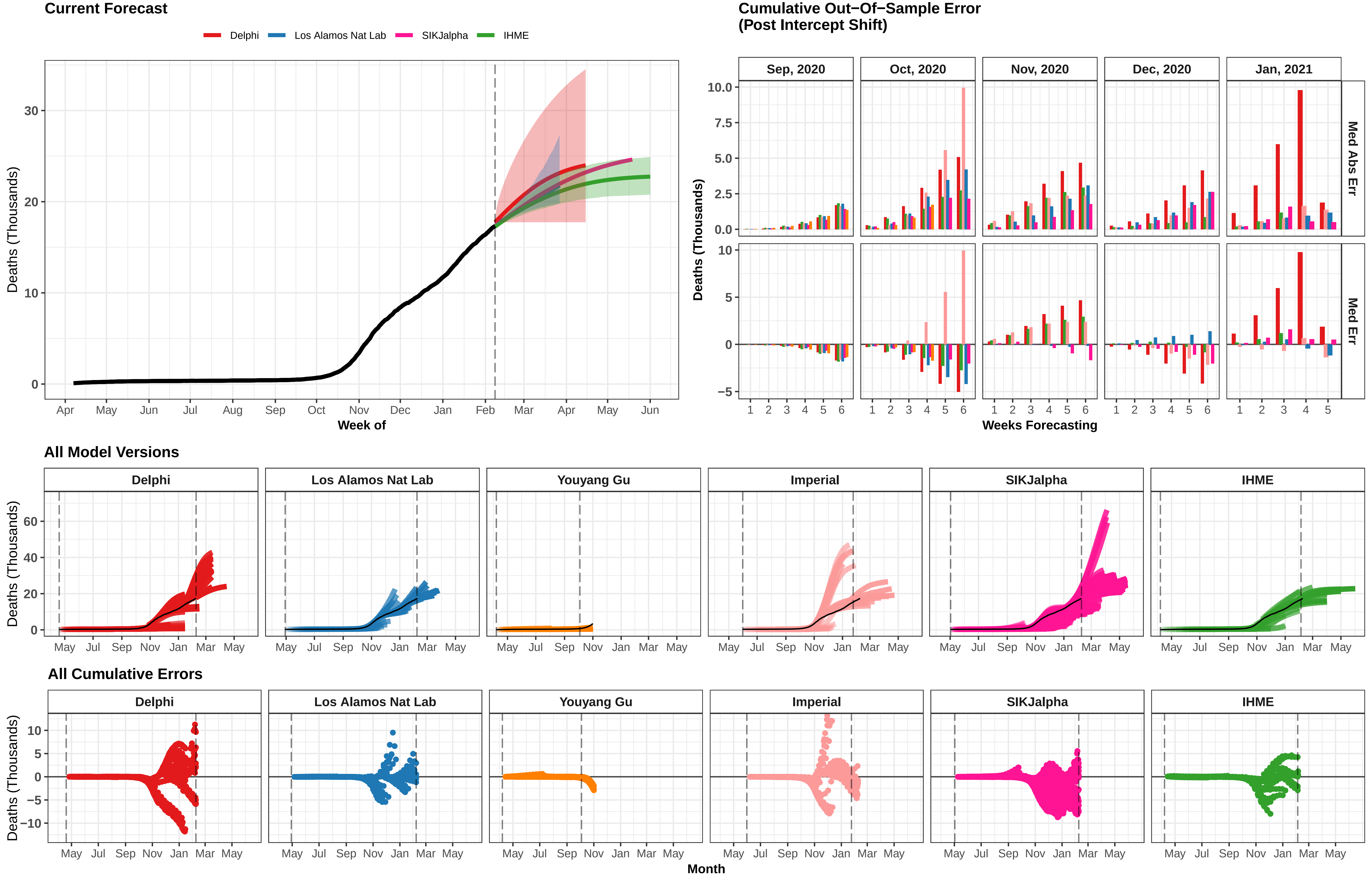

Michigan

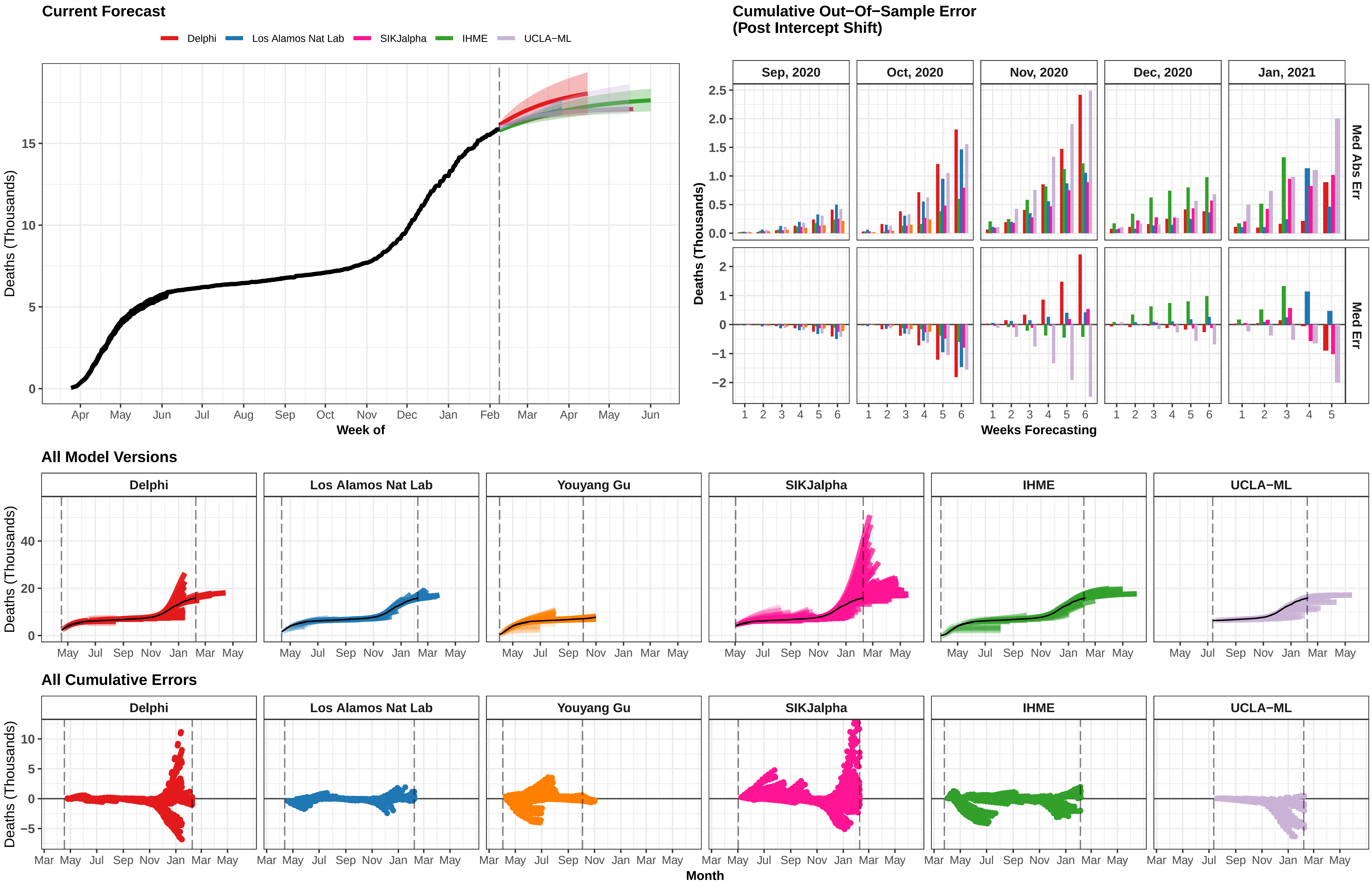
Current Forecast
Delphi
Los Alamos Nat Lab
SIKJalpha
IHME
UCLA−ML
Deaths (Thousands)
Week of
Cumulative Out−Of−Sample Error
(Post Intercept Shift)
Sep, 2020
Oct, 2020
Nov, 2020
Dec, 2020
Jan, 2021
Med Abs Err
Med Err
Weeks Forecasting
All Model Versions
Youyang Gu
All Cumulative Errors
Month

Georgia

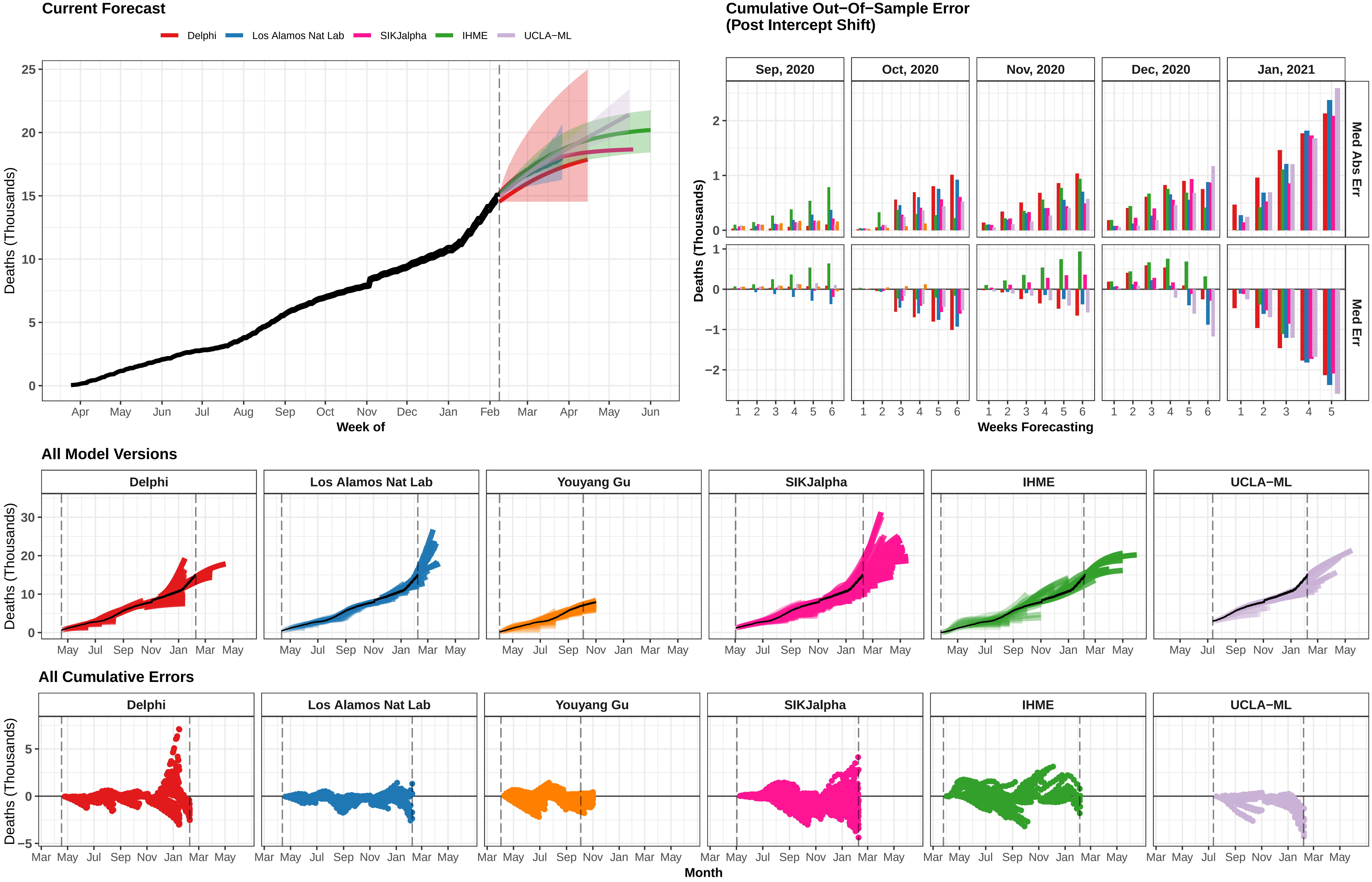
Current Forecast
Delphi
Los Alamos Nat Lab
SIKJalpha
IHME
UCLA−ML
Deaths (Thousands)
Week of
Apr May Jun Jul Aug Sep Oct Nov Dec Jan Feb Mar Apr May Jun
Cumulative Out−Of−Sample Error
(Post Intercept Shift)
Sep, 2020
Oct, 2020
Nov, 2020
Dec, 2020
Jan, 2021
Med Abs Err
Med Err
Weeks Forecasting
All Model Versions
Delphi
Los Alamos Nat Lab
Youyang Gu
SIKJalpha
IHME
UCLA−ML
All Cumulative Errors
Month

Massachusetts

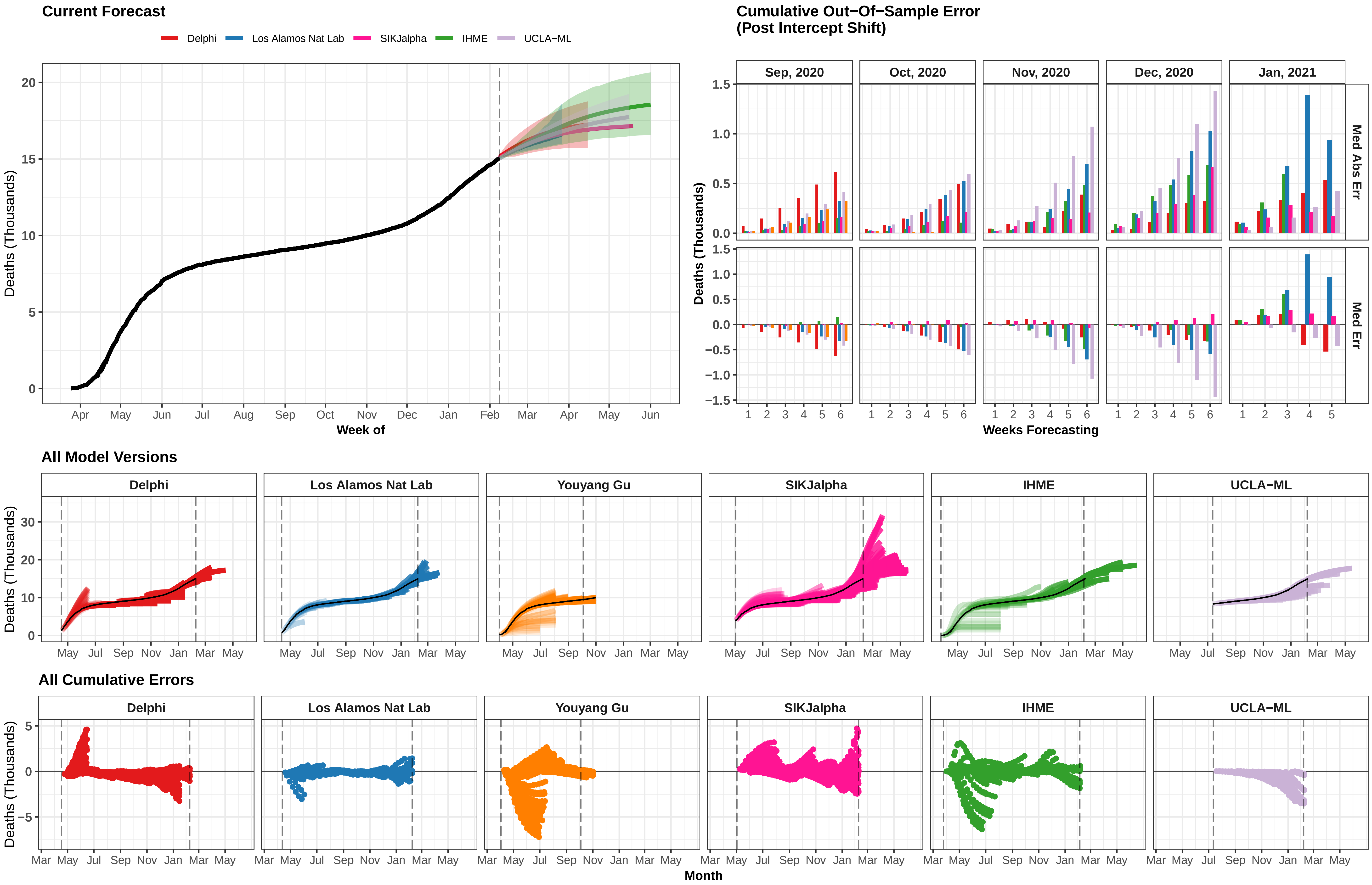

Netherlands

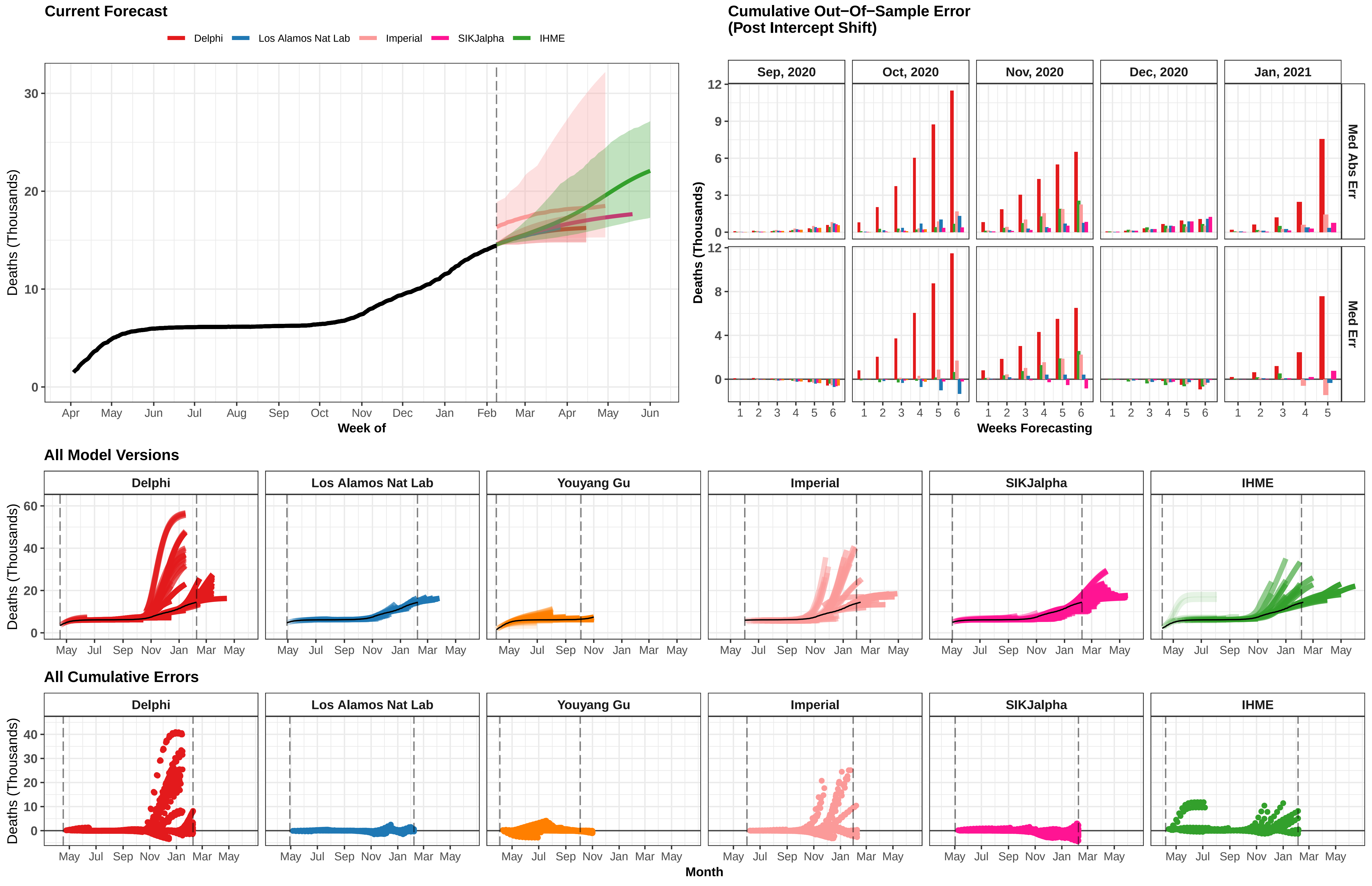

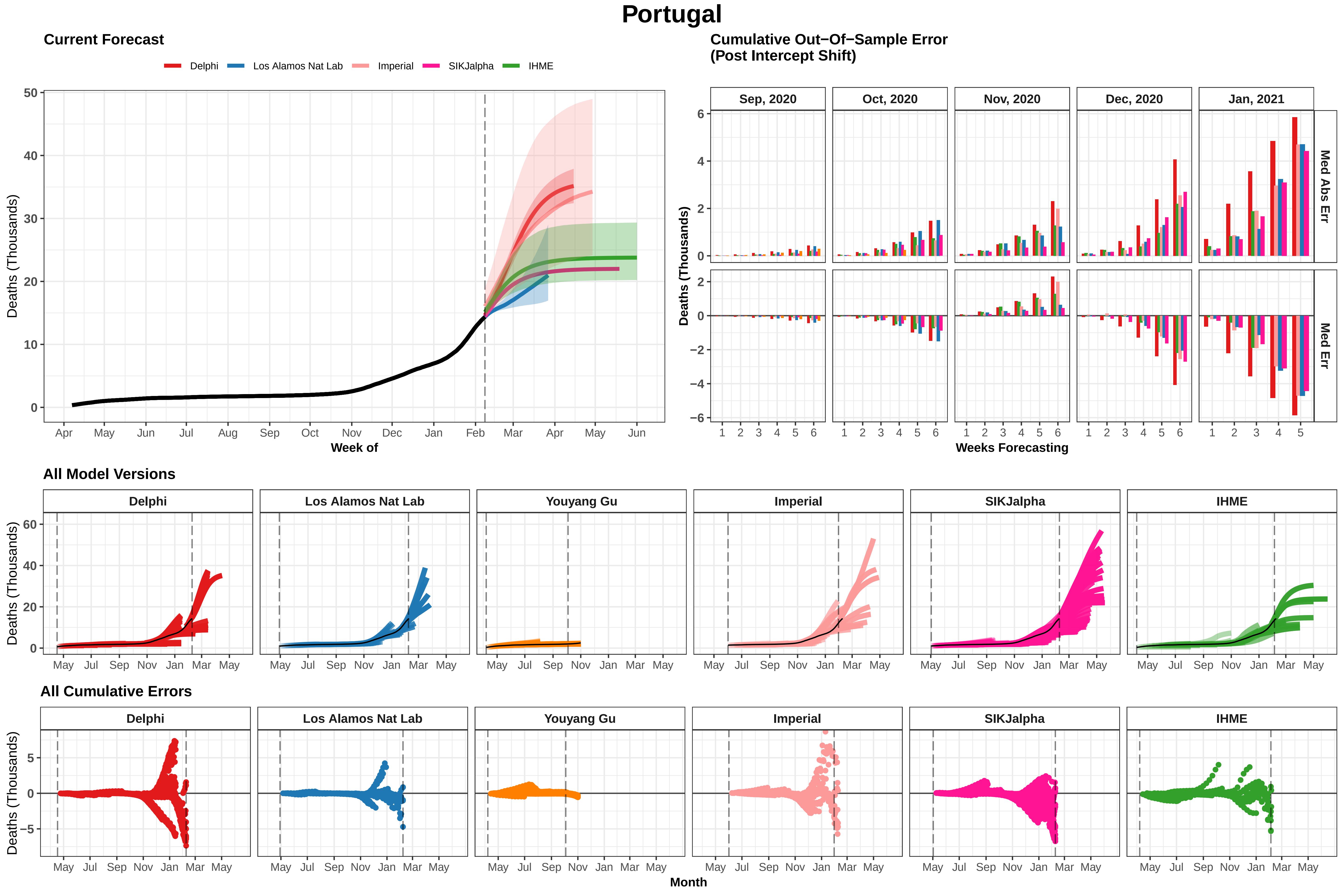
Portugal
Current Forecast
Delphi
Los Alamos Nat Lab
Imperial
SIKJalpha
IHME
Deaths (Thousands)
Week of
Apr May Jun Jul Aug Sep Oct Nov Dec Jan Feb Mar Apr May Jun
Cumulative Out−Of−Sample Error
(Post Intercept Shift)
Sep, 2020
Oct, 2020
Nov, 2020
Dec, 2020
Jan, 2021
Med Abs Err
Med Err
Weeks Forecasting
All Model Versions
Youyang Gu
All Cumulative Errors
Month

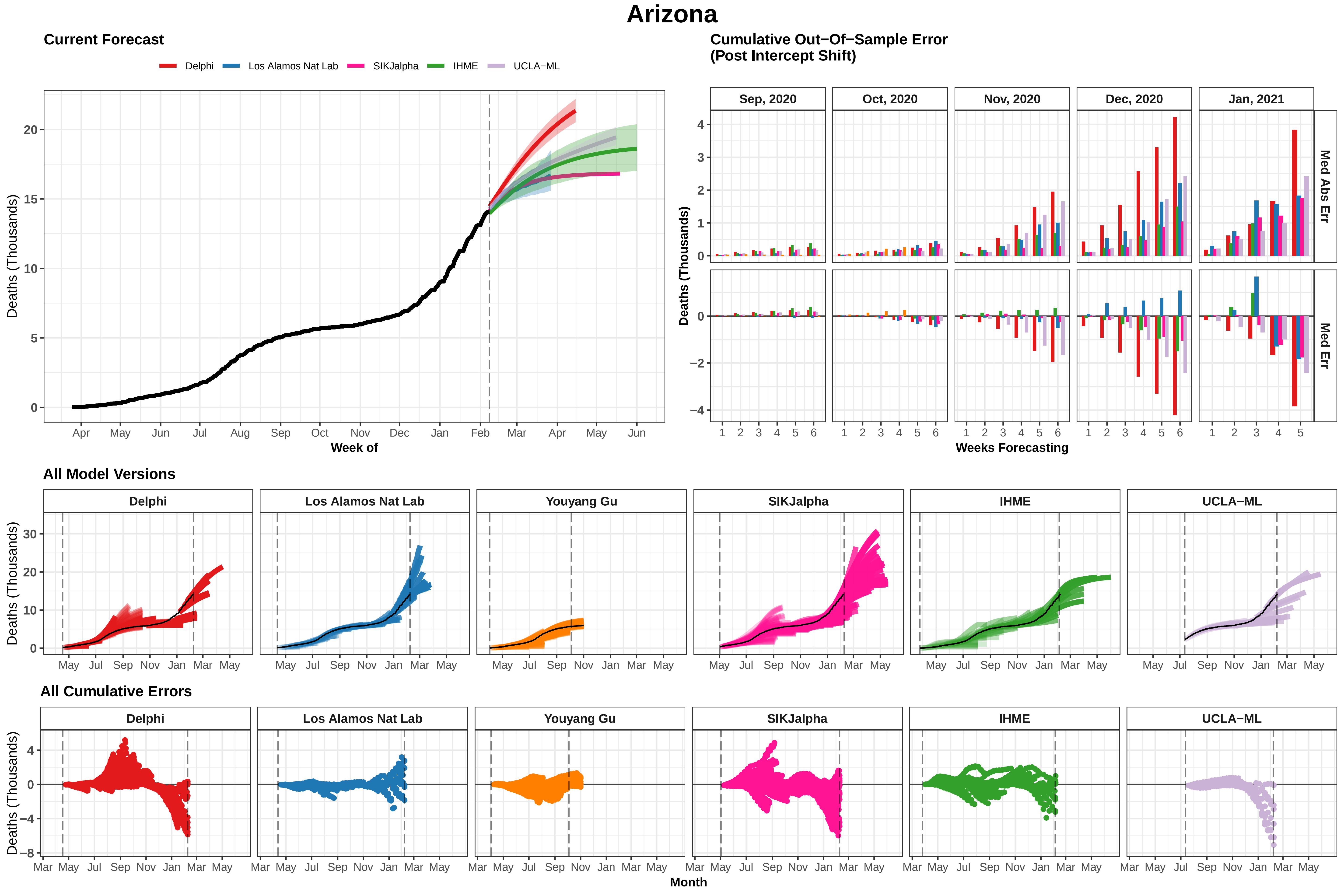
Arizona
Current Forecast
Delphi
Los Alamos Nat Lab
SIKJalpha
IHME
UCLA−ML
Deaths (Thousands)
Week of
Apr May Jun Jul Aug Sep Oct Nov Dec Jan Feb Mar Apr May Jun
Cumulative Out−Of−Sample Error
(Post Intercept Shift)
Sep, 2020
Oct, 2020
Nov, 2020
Dec, 2020
Jan, 2021
Med Abs Err
Med Err
Weeks Forecasting
All Model Versions
Delphi
Los Alamos Nat Lab
Youyang Gu
SIKJalpha
IHME
UCLA−ML
All Cumulative Errors
Month

Hungary

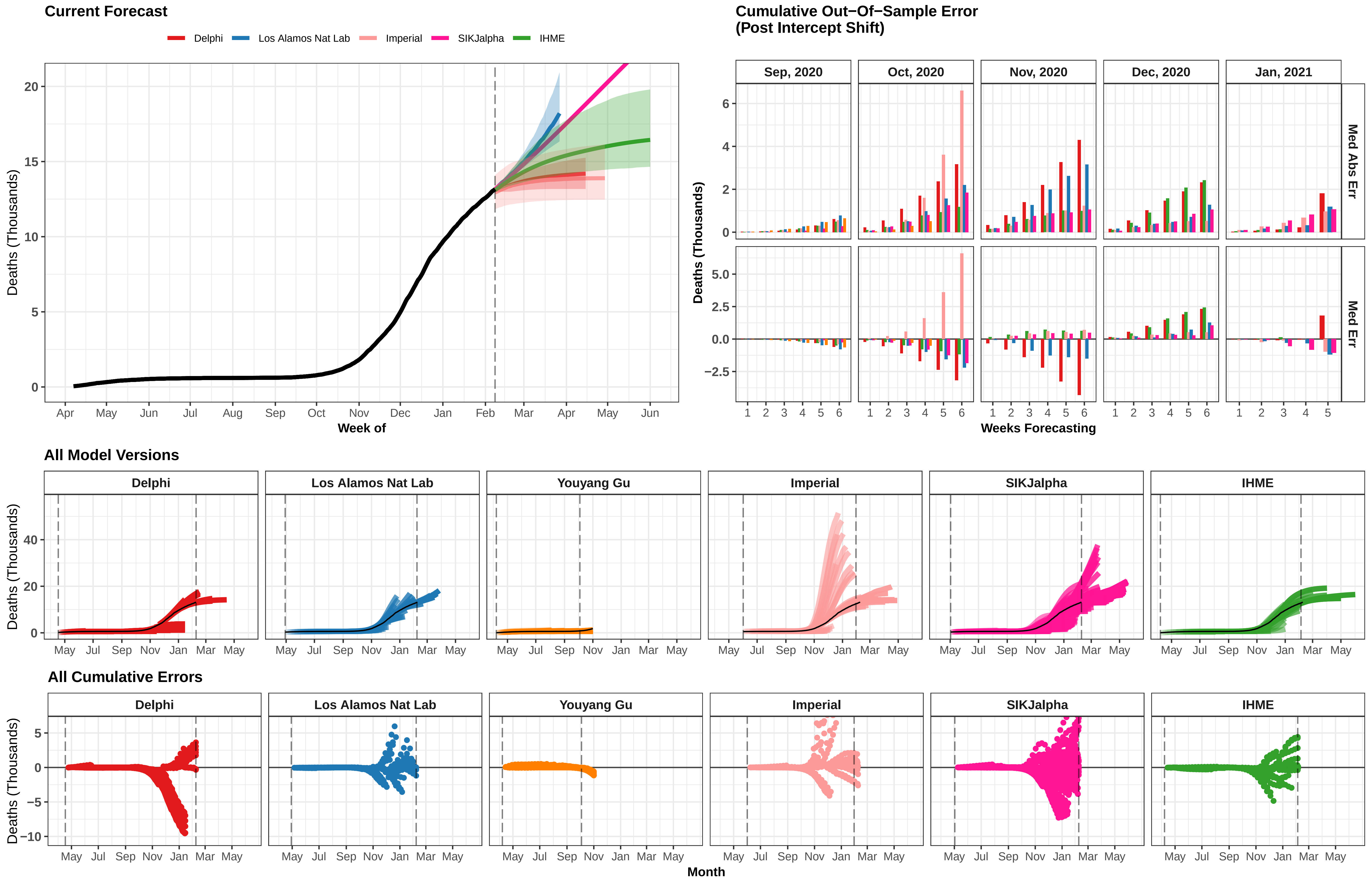
Current Forecast
Delphi
Los Alamos Nat Lab
Imperial
SIKJalpha
IHME
Deaths (Thousands)
Week of
Apr May Jun Jul Aug Sep Oct Nov Dec Jan Feb Mar Apr May Jun
Cumulative Out−Of−Sample Error
(Post Intercept Shift)
Sep, 2020
Oct, 2020
Nov, 2020
Dec, 2020
Jan, 2021
Med Abs Err
Med Err
Weeks Forecasting
All Model Versions
Delphi
Los Alamos Nat Lab
Youyang Gu
Imperial
SIKJalpha
IHME
All Cumulative Errors
Month

Iraq

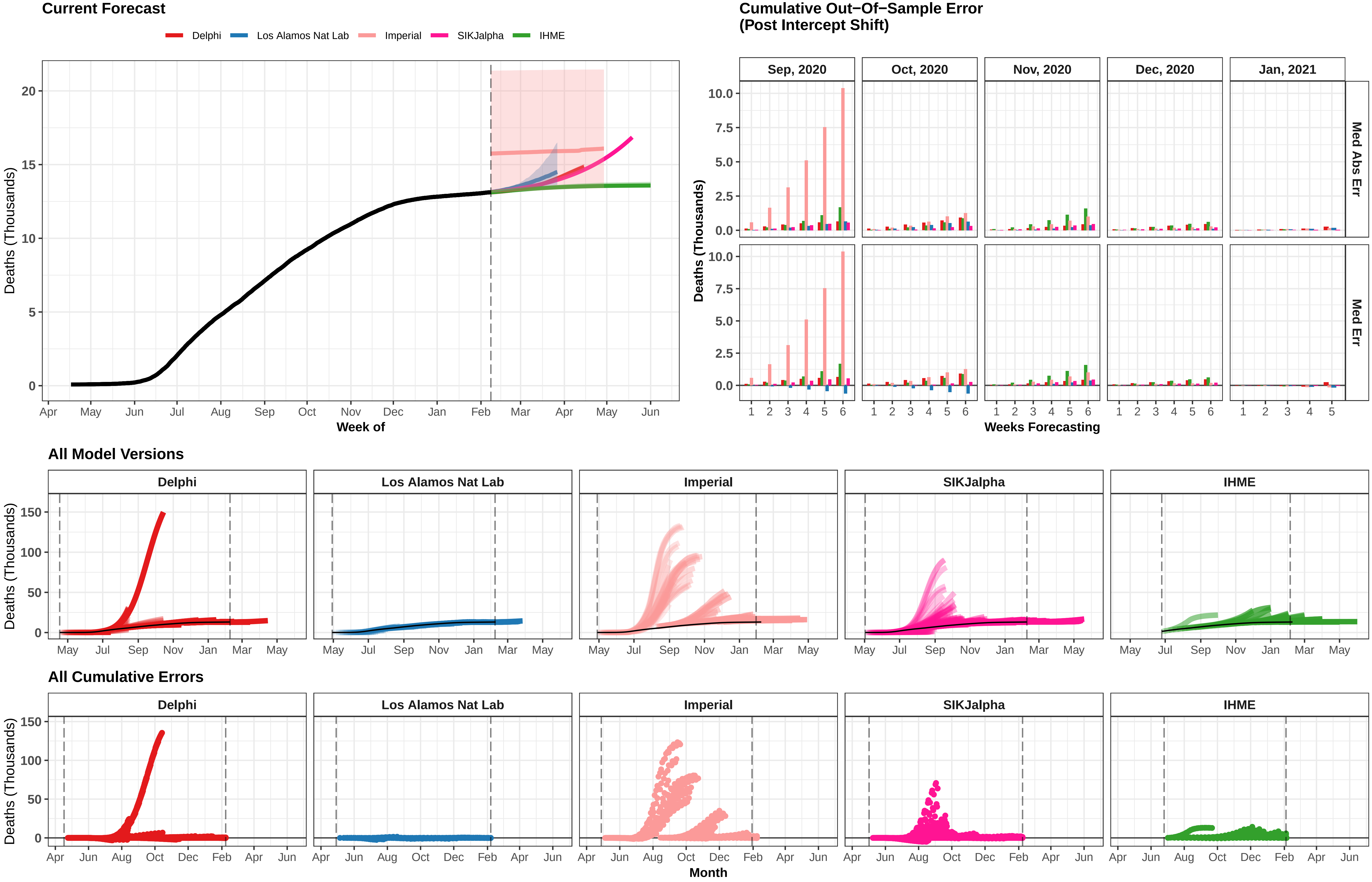

Current Forecast
Delphi
Los Alamos Nat Lab
Imperial
SIKJalpha
IHME
Deaths (Thousands)
Week of
Cumulative Out−Of−Sample Error
(Post Intercept Shift)
Sep, 2020
Oct, 2020
Nov, 2020
Dec, 2020
Jan, 2021
Med Abs Err
Med Err
Weeks Forecasting
All Model Versions
All Cumulative Errors
Month

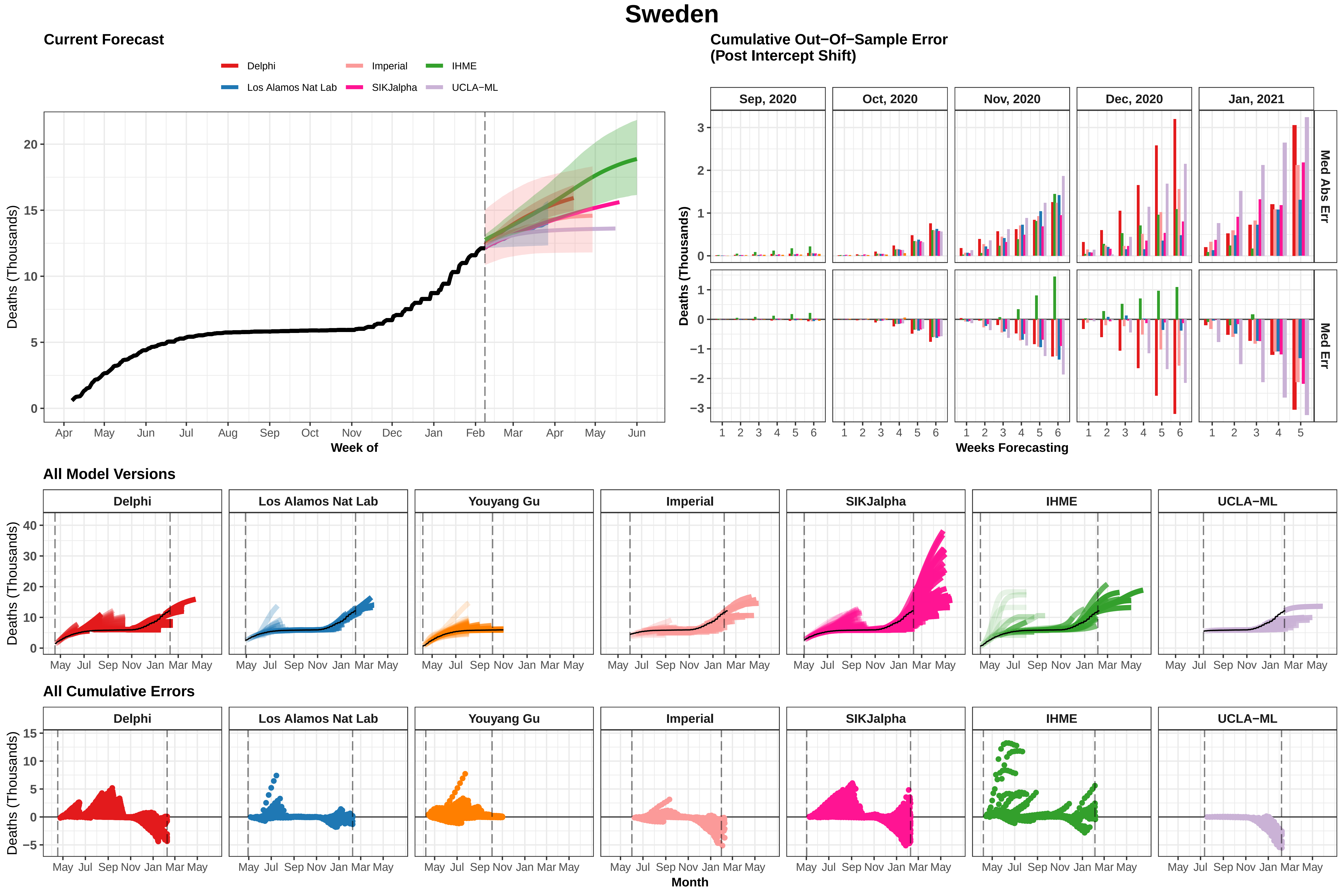
Sweden
Current Forecast
Delphi
Imperial
IHME
Los Alamos Nat Lab
SIKJalpha
UCLA−ML
Deaths (Thousands)
Week of
Apr May Jun Jul Aug Sep Oct Nov Dec Jan Feb Mar Apr May Jun
Cumulative Out−Of−Sample Error
(Post Intercept Shift)
Sep, 2020
Oct, 2020
Nov, 2020
Dec, 2020
Jan, 2021
Med Abs Err
Med Err
Weeks Forecasting
All Model Versions
Delphi
Los Alamos Nat Lab
Youyang Gu
Imperial
SIKJalpha
IHME
UCLA−ML
May Jul Sep Nov Jan Mar May
All Cumulative Errors
Month

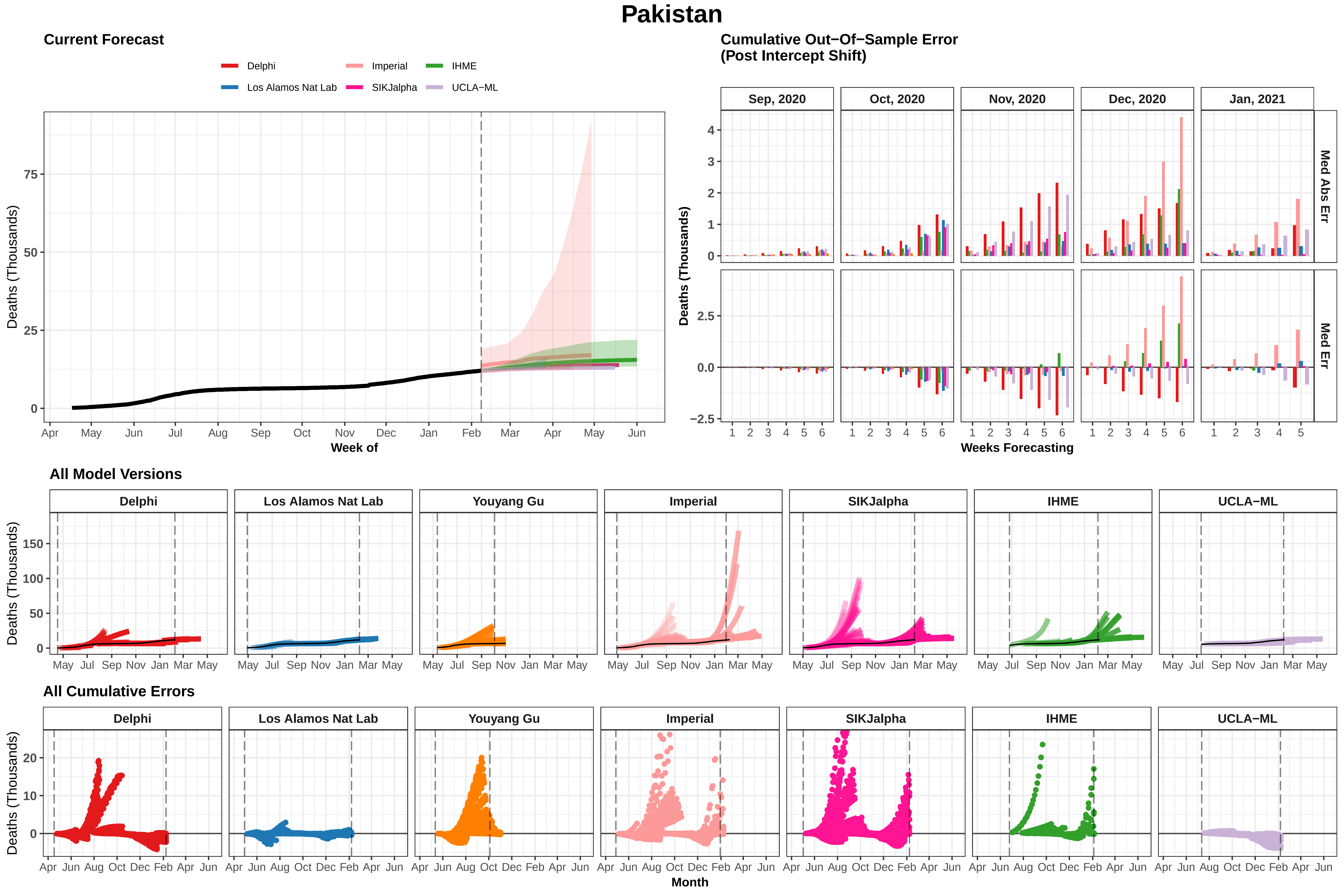
Pakistan
Current Forecast
Delphi
Imperial
IHME
Los Alamos Nat Lab
SIKJalpha
UCLA−ML
Deaths (Thousands)
Week of
Apr May Jun Jul Aug Sep Oct Nov Dec Jan Feb Mar Apr May Jun
Cumulative Out−Of−Sample Error
(Post Intercept Shift)
Sep, 2020
Oct, 2020
Nov, 2020
Dec, 2020
Jan, 2021
Med Abs Err
Med Err
Weeks Forecasting
All Model Versions
Delphi
Los Alamos Nat Lab
Youyang Gu
Imperial
SIKJalpha
IHME
UCLA−ML
May Jul Sep Nov Jan Mar May
All Cumulative Errors
Month
Apr Jun Aug Oct Dec Feb Apr Jun

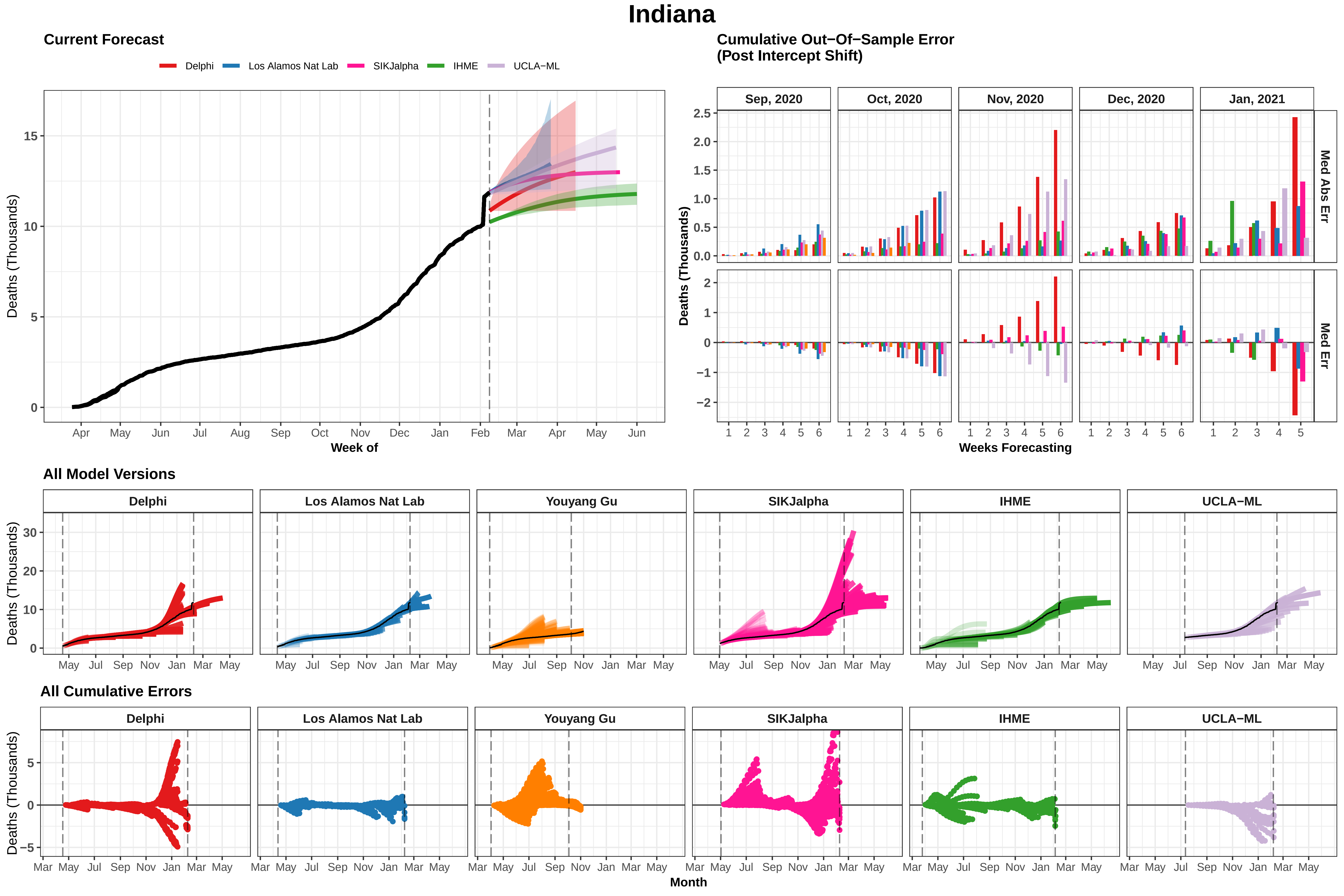

Indiana
Current Forecast
Delphi
Los Alamos Nat Lab
SIKJalpha
IHME
UCLA−ML
Deaths (Thousands)
Week of
Apr May Jun Jul Aug Sep Oct Nov Dec Jan Feb Mar Apr May Jun
Cumulative Out−Of−Sample Error
(Post Intercept Shift)
Sep, 2020
Oct, 2020
Nov, 2020
Dec, 2020
Jan, 2021
Med Abs Err
Med Err
Weeks Forecasting
All Model Versions
Delphi
Los Alamos Nat Lab
Youyang Gu
SIKJalpha
IHME
UCLA−ML
All Cumulative Errors
Month

Ohio

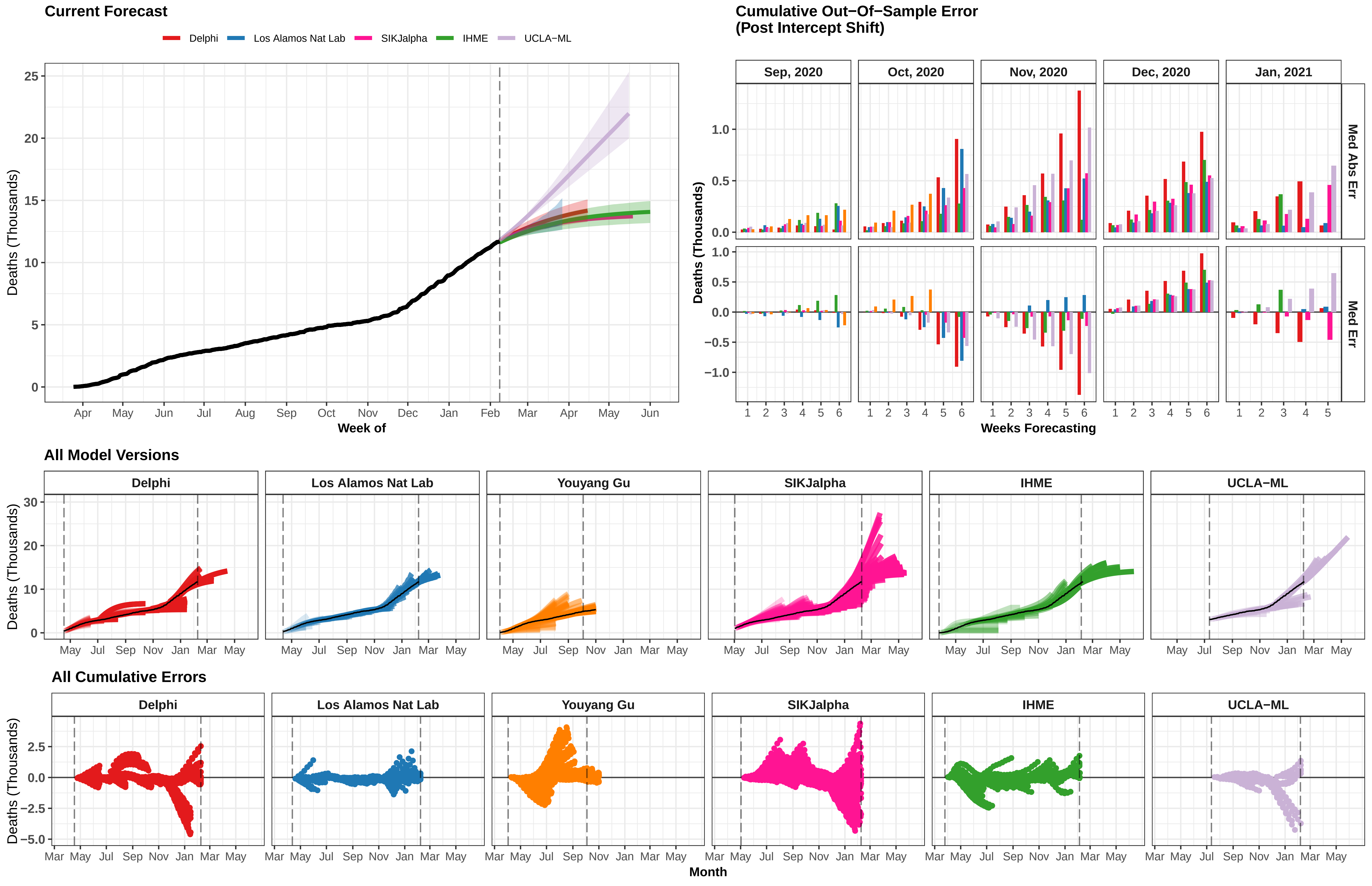
Current Forecast
Delphi
Los Alamos Nat Lab
SIKJalpha
IHME
UCLA−ML
Deaths (Thousands)
Week of
Apr May Jun Jul Aug Sep Oct Nov Dec Jan Feb Mar Apr May Jun
Cumulative Out−Of−Sample Error
(Post Intercept Shift)
Sep, 2020
Oct, 2020
Nov, 2020
Dec, 2020
Jan, 2021
Med Abs Err
Med Err
Weeks Forecasting
All Model Versions
Delphi
Los Alamos Nat Lab
Youyang Gu
SIKJalpha
IHME
UCLA−ML
All Cumulative Errors
Month

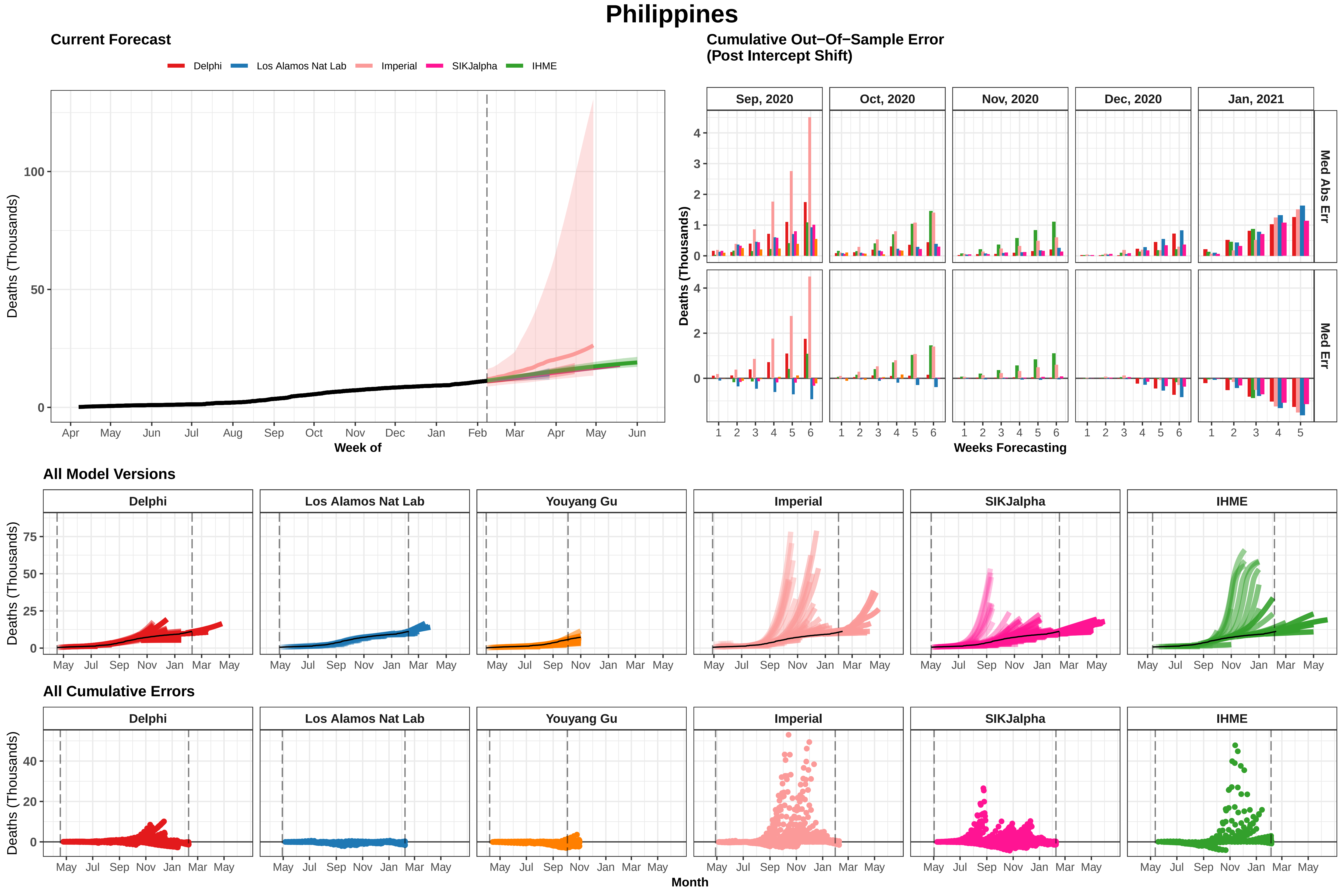
Philippines
Current Forecast
Delphi
Los Alamos Nat Lab
Imperial
SIKJalpha
IHME
Deaths (Thousands)
Week of
Apr May Jun Jul Aug Sep Oct Nov Dec Jan Feb Mar Apr May Jun
Cumulative Out−Of−Sample Error
(Post Intercept Shift)
Sep, 2020
Oct, 2020
Nov, 2020
Dec, 2020
Jan, 2021
Med Abs Err
Med Err
Weeks Forecasting
All Model Versions
Delphi
Los Alamos Nat Lab
Youyang Gu
Imperial
SIKJalpha
IHME
All Cumulative Errors
Month

Bolivia

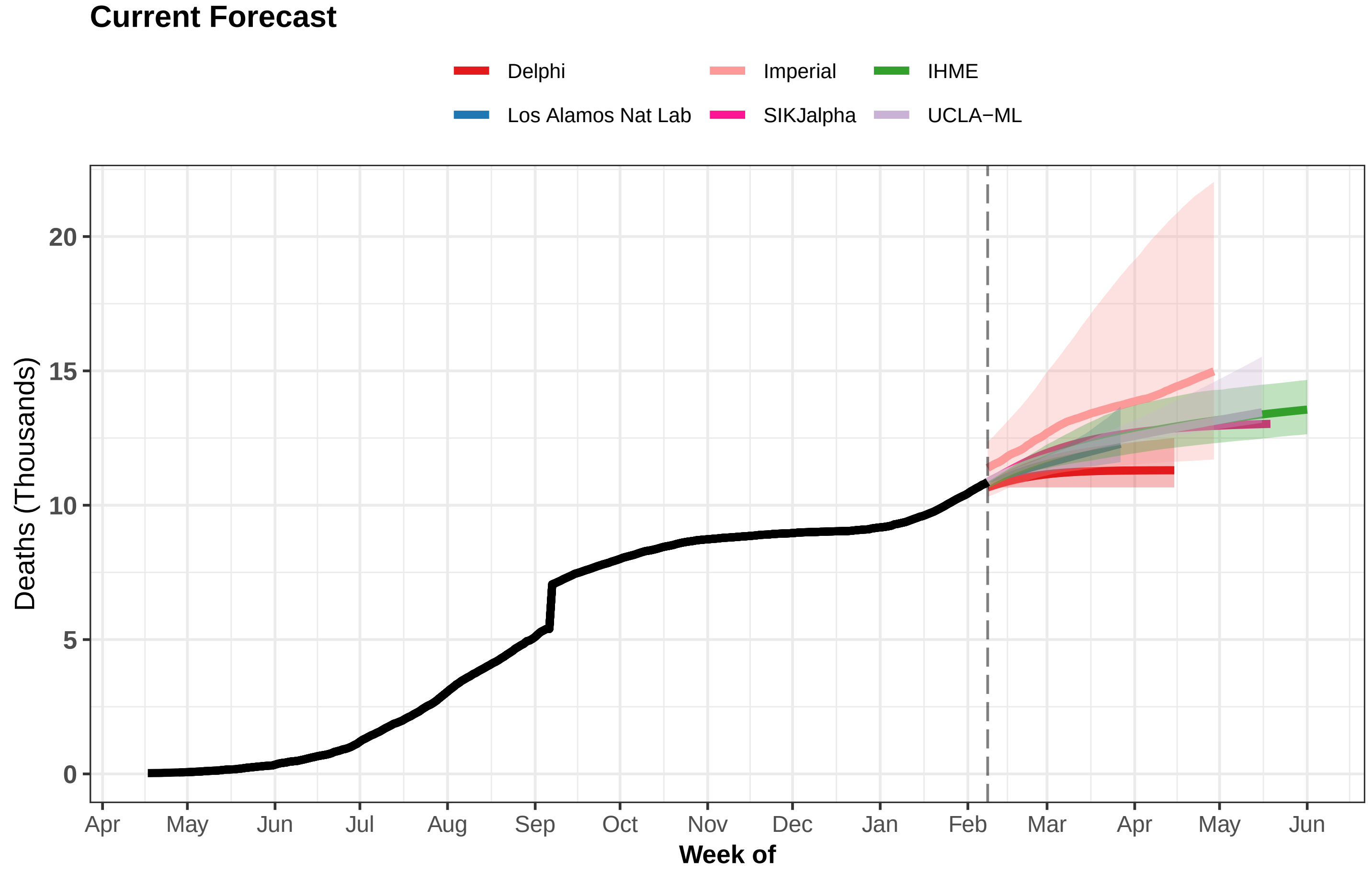

Current Forecast
Delphi
Los Alamos Nat Lab
Imperial
SIKJalpha
IHME
UCLA−ML
Deaths (Thousands)
Week of
Apr May Jun Jul Aug Sep Oct Nov Dec Jan Feb Mar Apr May Jun

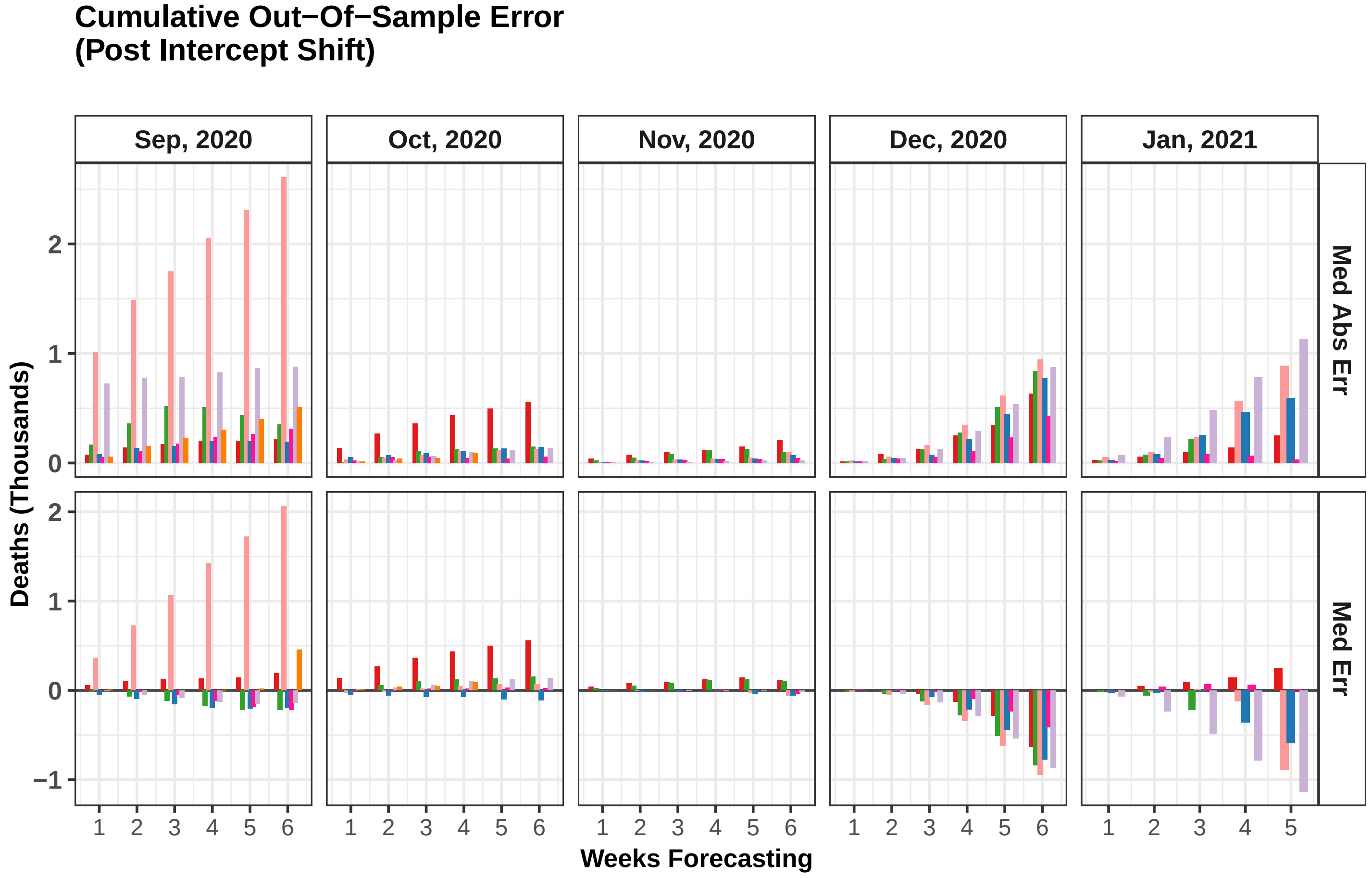

Cumulative Out−Of−Sample Error
(Post Intercept Shift)
Sep, 2020
Oct, 2020
Nov, 2020
Dec, 2020
Jan, 2021
Med Abs Err
Med Err
Deaths (Thousands)
Weeks Forecasting

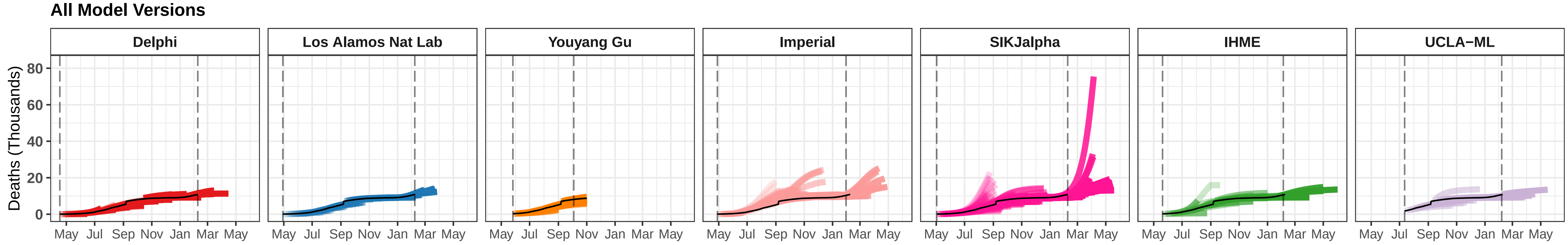

All Model Versions
Delphi
Los Alamos Nat Lab
Youyang Gu
Imperial
SIKJalpha
IHME
UCLA−ML
Deaths (Thousands)
May Jul Sep Nov Jan Mar May

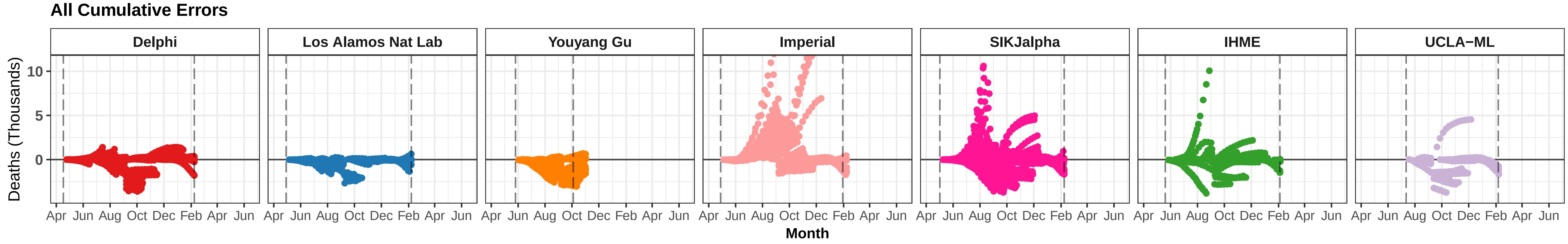

All Cumulative Errors
Delphi
Los Alamos Nat Lab
Youyang Gu
Imperial
SIKJalpha
IHME
UCLA−ML
Deaths (Thousands)
Apr Jun Aug Oct Dec Feb Apr Jun
Month

Tennessee

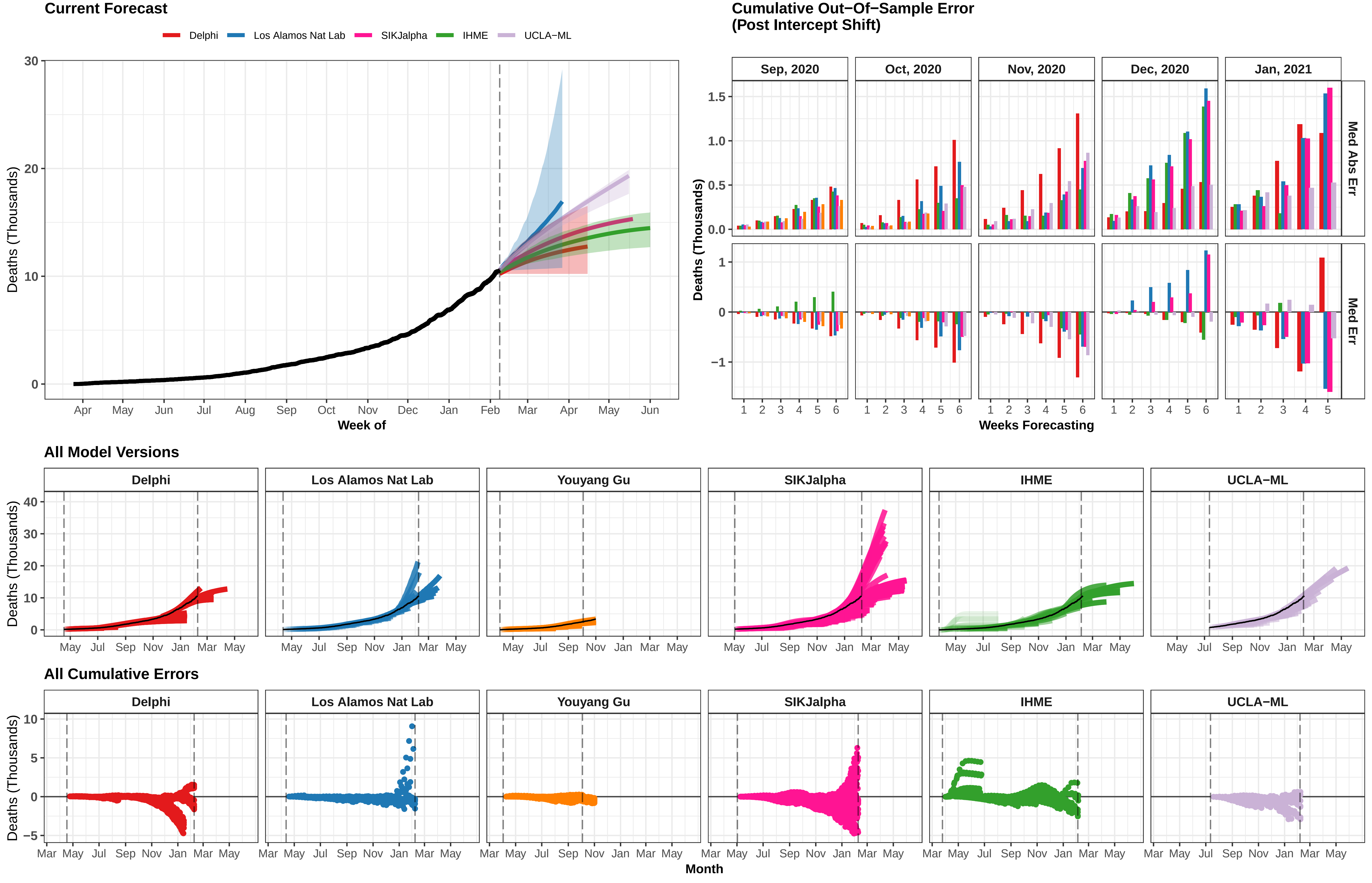
Current Forecast
Delphi
Los Alamos Nat Lab
SIKJalpha
IHME
UCLA−ML
Deaths (Thousands)
Week of
Apr May Jun Jul Aug Sep Oct Nov Dec Jan Feb Mar Apr May Jun
Cumulative Out−Of−Sample Error
(Post Intercept Shift)
Sep, 2020
Oct, 2020
Nov, 2020
Dec, 2020
Jan, 2021
Med Abs Err
Med Err
Weeks Forecasting
All Model Versions
Delphi
Los Alamos Nat Lab
Youyang Gu
SIKJalpha
IHME
UCLA−ML
Month
All Cumulative Errors

North Carolina

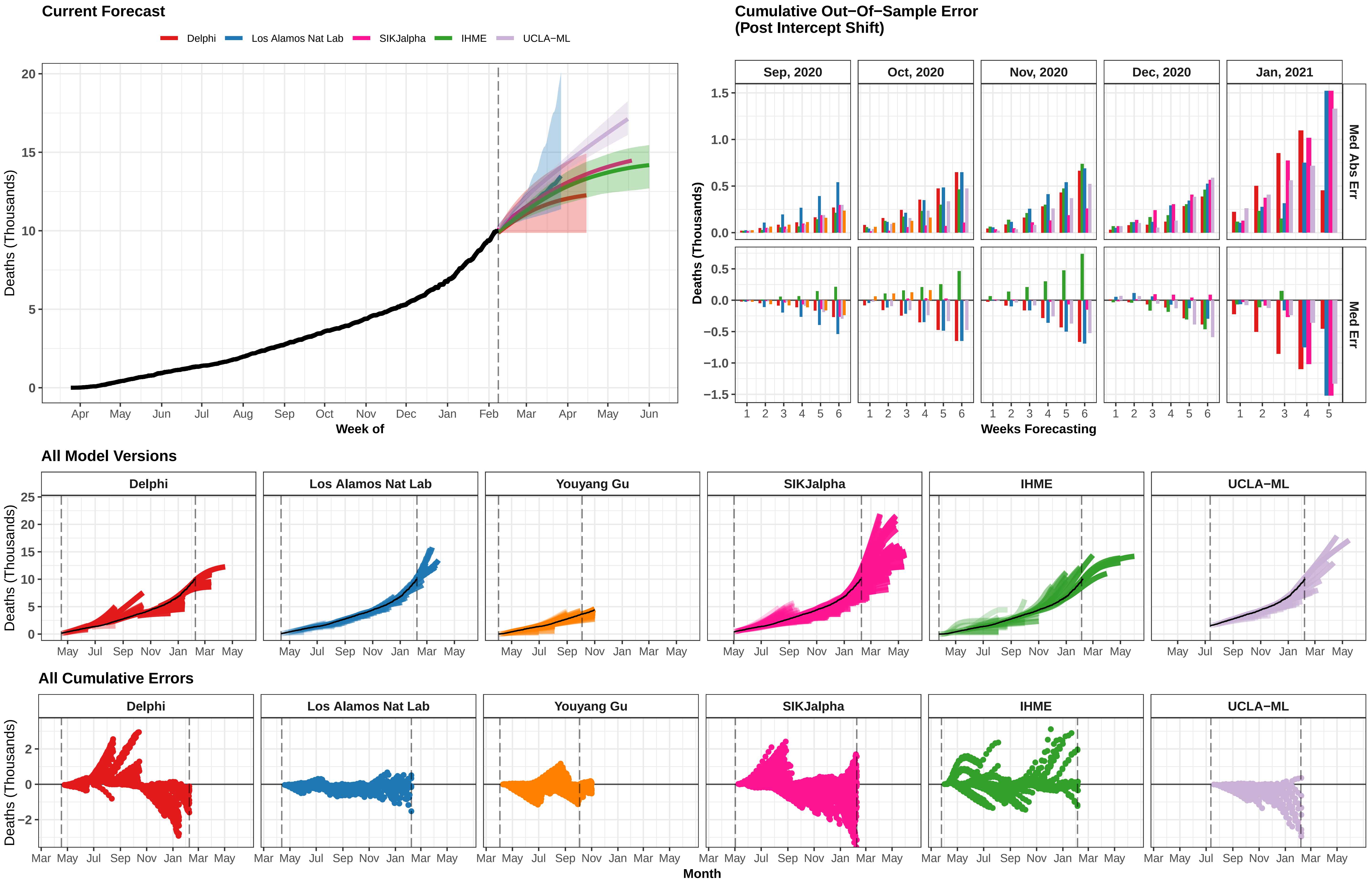

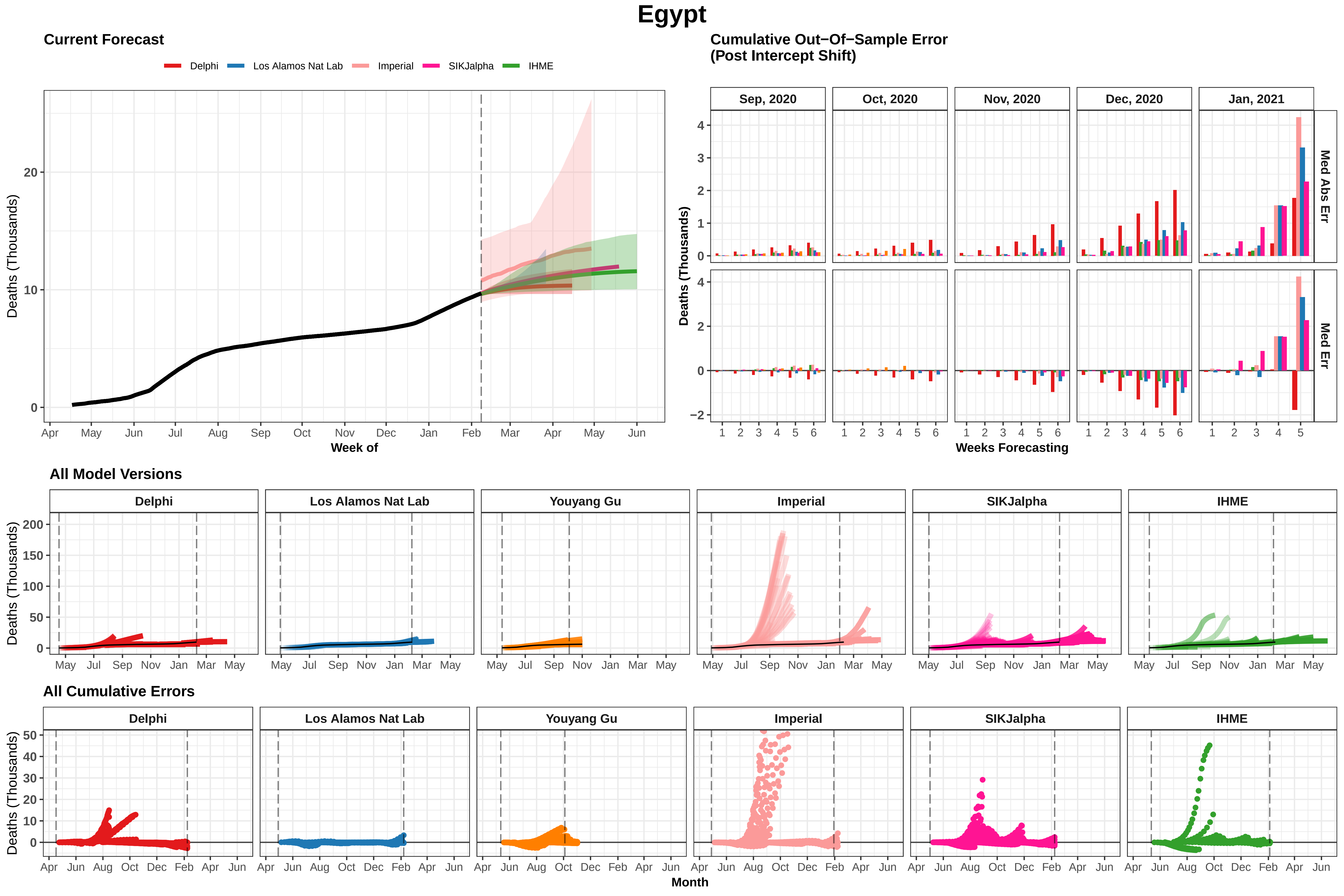

Egypt
Current Forecast
Delphi
Los Alamos Nat Lab
Imperial
SIKJalpha
IHME
Deaths (Thousands)
Week of
Cumulative Out−Of−Sample Error
(Post Intercept Shift)
Sep, 2020
Oct, 2020
Nov, 2020
Dec, 2020
Jan, 2021
Med Abs Err
Med Err
Weeks Forecasting
All Model Versions
Delphi
Los Alamos Nat Lab
Youyang Gu
Imperial
SIKJalpha
IHME
All Cumulative Errors
Month

Switzerland

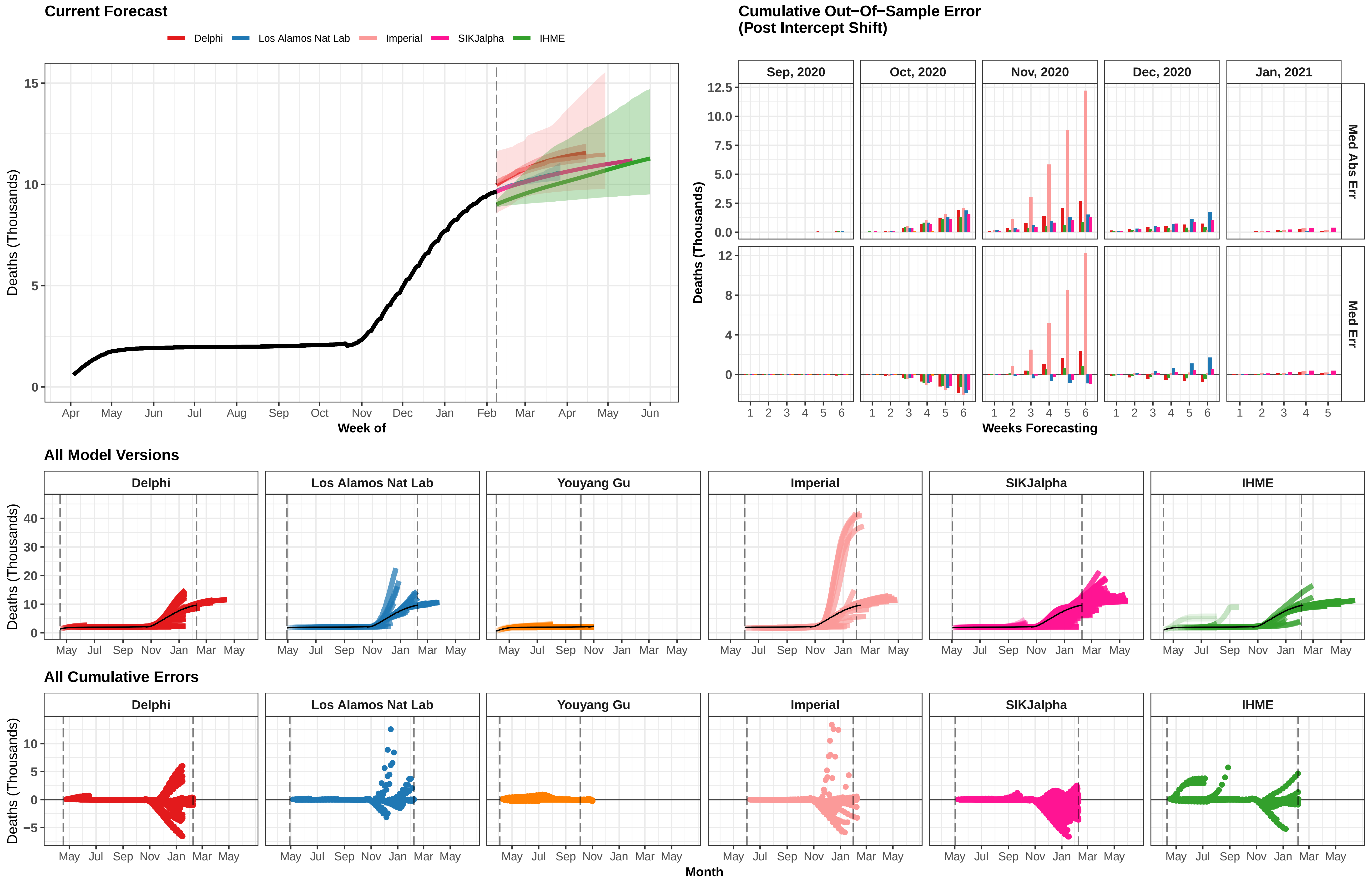

Bulgaria

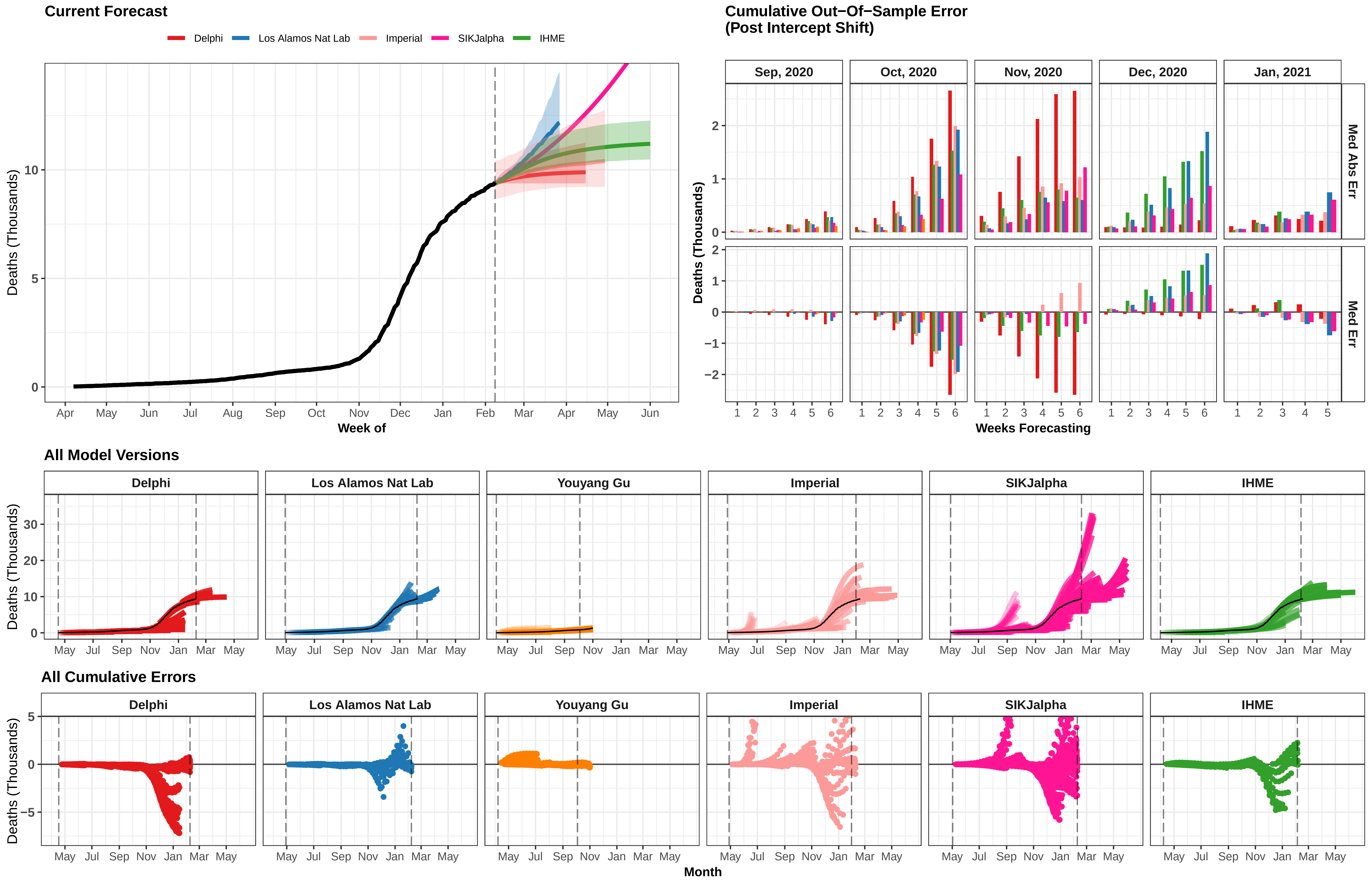

Current Forecast
Delphi
Los Alamos Nat Lab
Imperial
SIKJalpha
IHME
Deaths (Thousands)
Week of
Apr May Jun Jul Aug Sep Oct Nov Dec Jan Feb Mar Apr May Jun
Cumulative Out−Of−Sample Error
(Post Intercept Shift)
Sep, 2020
Oct, 2020
Nov, 2020
Dec, 2020
Jan, 2021
Med Abs Err
Med Err
Weeks Forecasting
All Model Versions
Delphi
Los Alamos Nat Lab
Youyang Gu
Imperial
SIKJalpha
IHME
All Cumulative Errors
Month

Louisiana

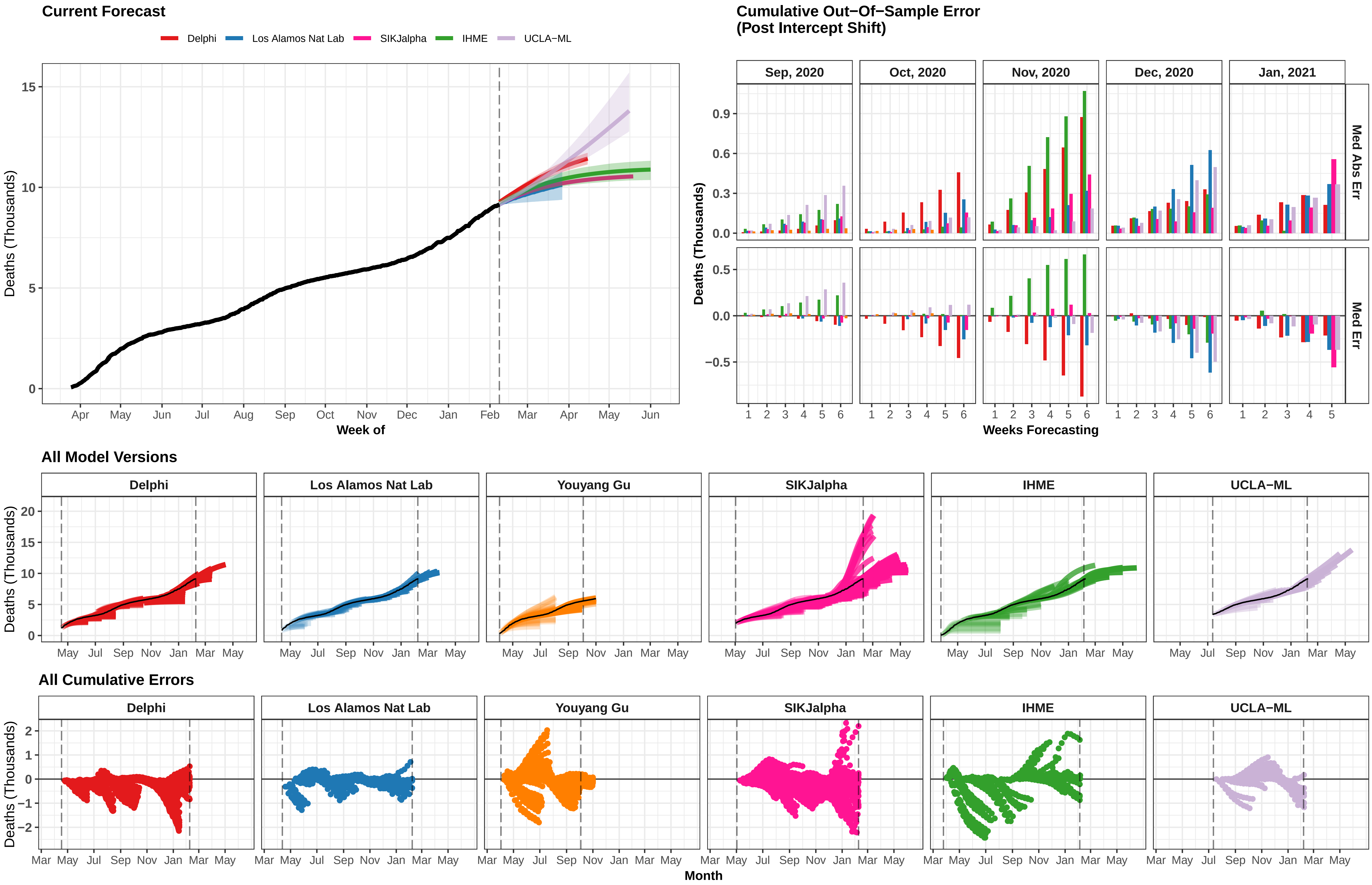
Current Forecast
Delphi
Los Alamos Nat Lab
SIKJalpha
IHME
UCLA−ML
Deaths (Thousands)
Week of
Apr May Jun Jul Aug Sep Oct Nov Dec Jan Feb Mar Apr May Jun
Cumulative Out−Of−Sample Error
(Post Intercept Shift)
Sep, 2020
Oct, 2020
Nov, 2020
Dec, 2020
Jan, 2021
Med Abs Err
Med Err
Weeks Forecasting
All Model Versions
Delphi
Los Alamos Nat Lab
Youyang Gu
SIKJalpha
IHME
UCLA−ML
All Cumulative Errors
Month

Alabama

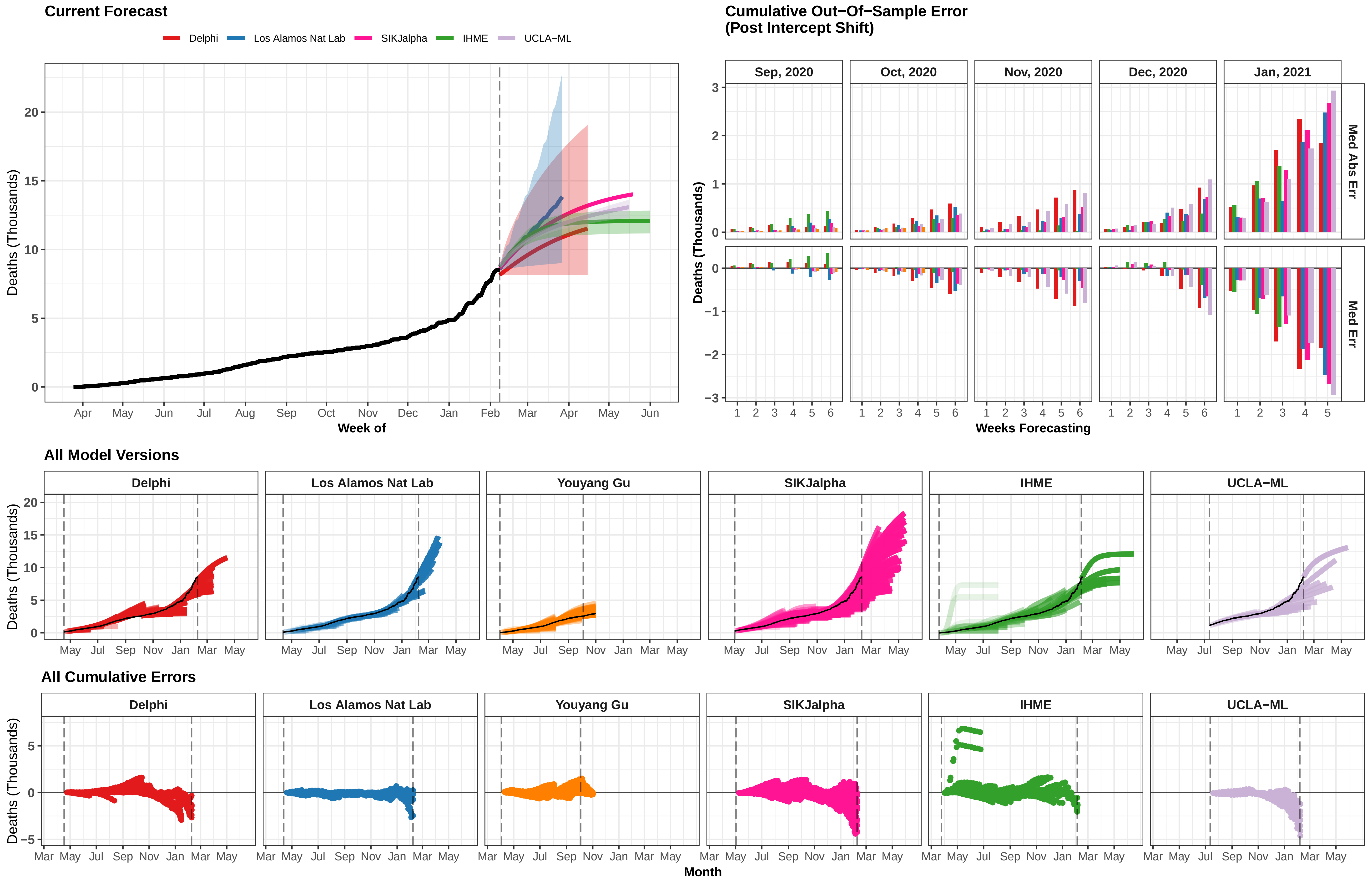

Current Forecast
Delphi
Los Alamos Nat Lab
SIKJalpha
IHME
UCLA−ML
Deaths (Thousands)
Week of
Apr
May
Jun
Jul
Aug
Sep
Oct
Nov
Dec
Jan
Feb
Mar
Cumulative Out−Of−Sample Error
(Post Intercept Shift)
Sep, 2020
Oct, 2020
Nov, 2020
Dec, 2020
Jan, 2021
Med Abs Err
Med Err
Weeks Forecasting
All Model Versions
Youyang Gu
All Cumulative Errors
Month

Morocco

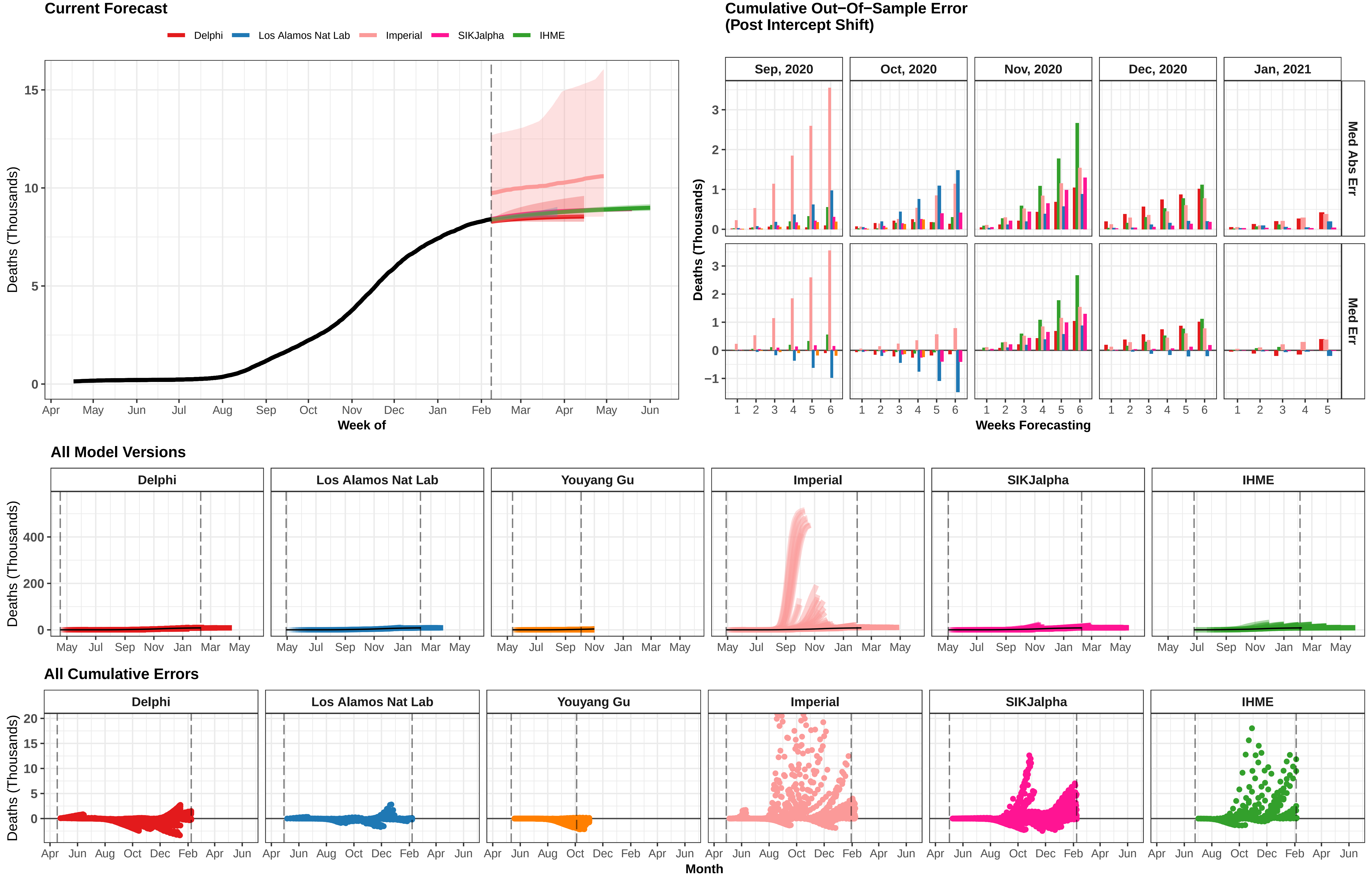
Current Forecast
Delphi
Los Alamos Nat Lab
Imperial
SIKJalpha
IHME
Deaths (Thousands)
Week of
Apr May Jun Jul Aug Sep Oct Nov Dec Jan Feb Mar Apr May Jun
Cumulative Out−Of−Sample Error
(Post Intercept Shift)
Sep, 2020
Oct, 2020
Nov, 2020
Dec, 2020
Jan, 2021
Med Abs Err
Med Err
Weeks Forecasting
All Model Versions
Delphi
Los Alamos Nat Lab
Youyang Gu
Imperial
SIKJalpha
IHME
May Jul Sep Nov Jan Mar May
All Cumulative Errors
Month
Apr Jun Aug Oct Dec Feb Apr Jun

Bangladesh

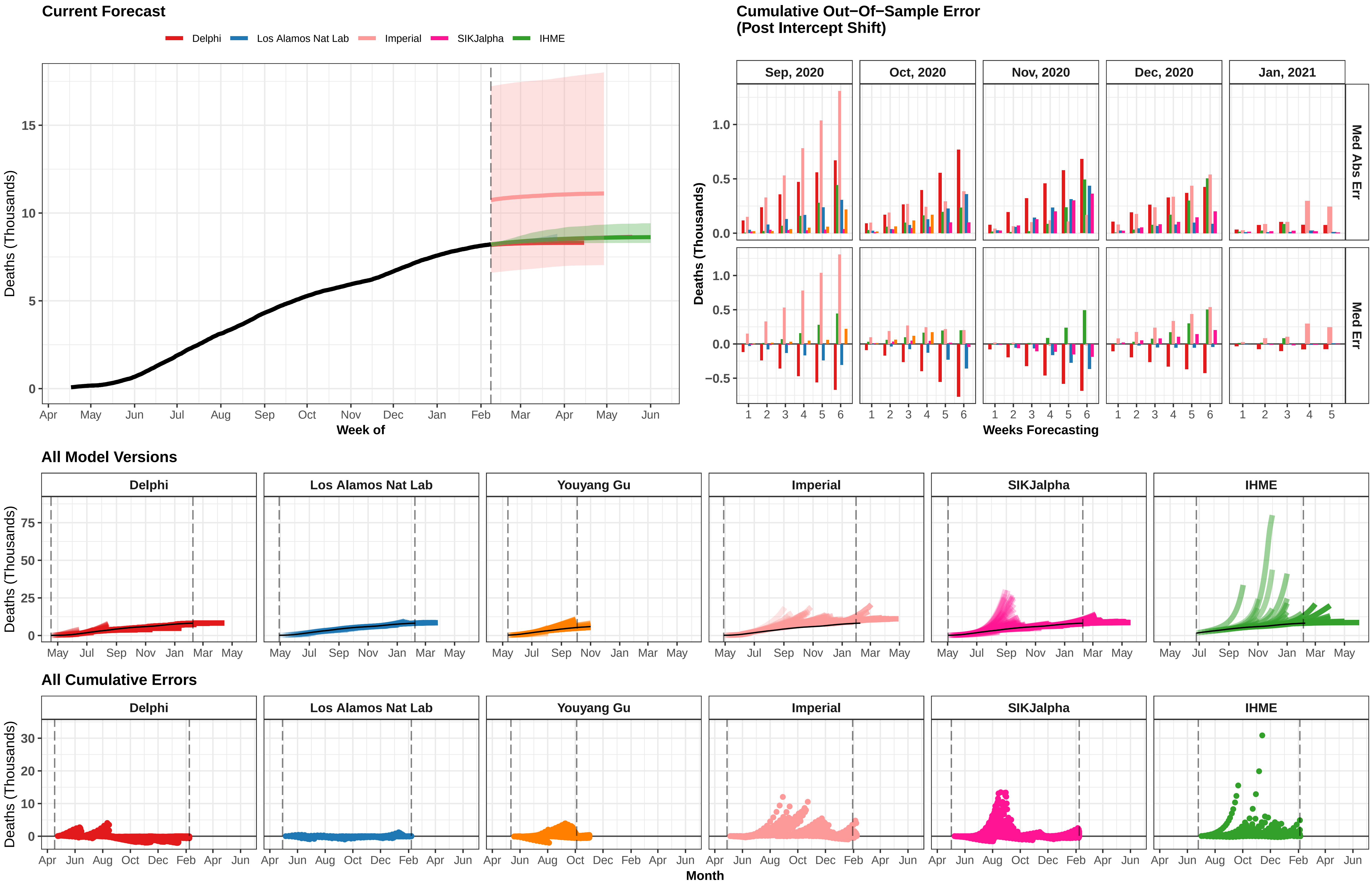

Current Forecast
Delphi
Los Alamos Nat Lab
Imperial
SIKJalpha
IHME
Deaths (Thousands)
Week of
Apr May Jun Jul Aug Sep Oct Nov Dec Jan Feb Mar Apr May Jun
Cumulative Out−Of−Sample Error (Post Intercept Shift)
Sep, 2020
Oct, 2020
Nov, 2020
Dec, 2020
Jan, 2021
Med Abs Err
Med Err
Weeks Forecasting
All Model Versions
Delphi
Los Alamos Nat Lab
Youyang Gu
Imperial
SIKJalpha
IHME
All Cumulative Errors
Month

Austria

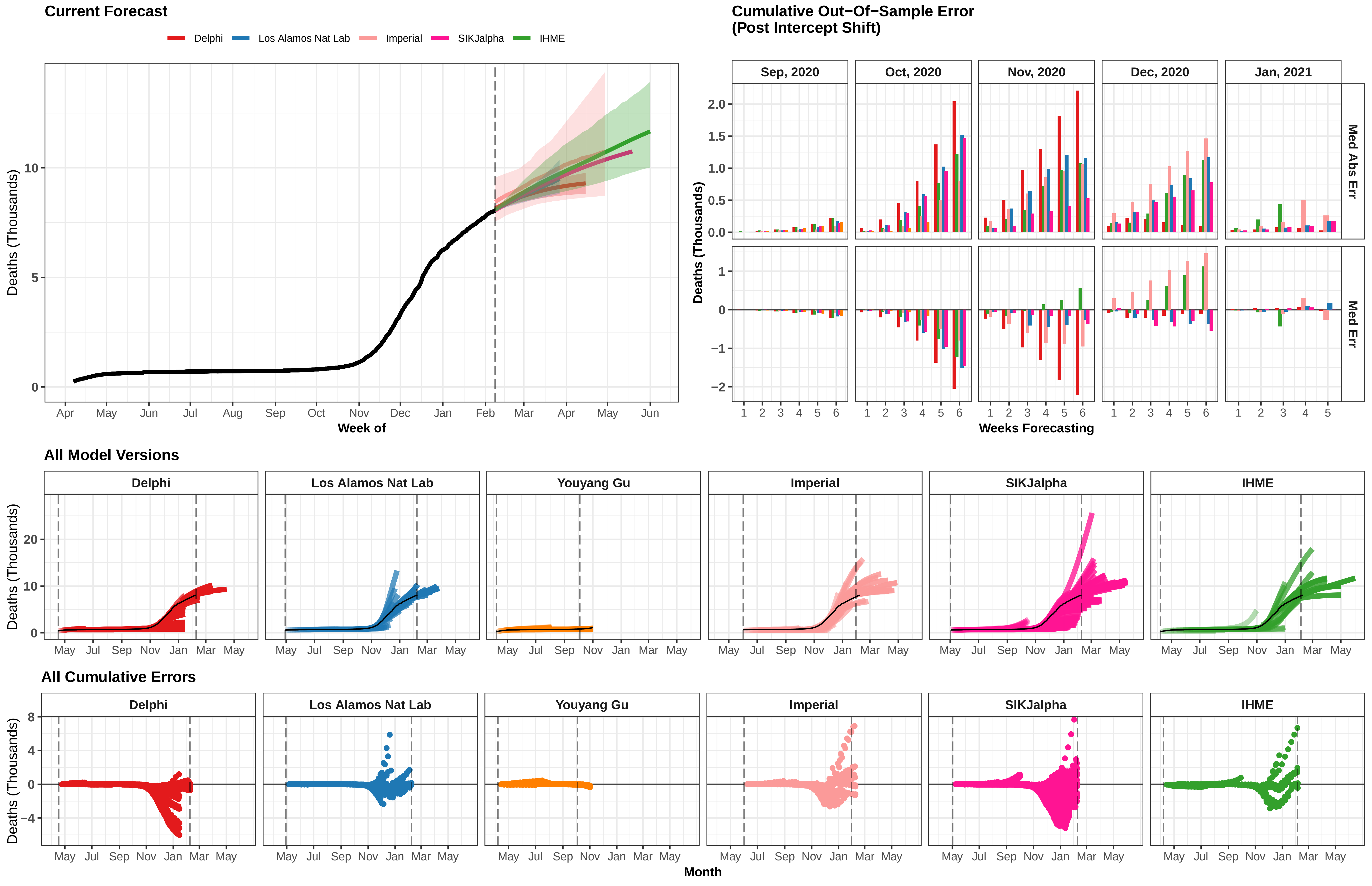
Current Forecast
Delphi
Los Alamos Nat Lab
Imperial
SIKJalpha
IHME
Deaths (Thousands)
Week of
Apr
May
Jun
Jul
Aug
Sep
Oct
Nov
Dec
Jan
Feb
Mar
Cumulative Out−Of−Sample Error
(Post Intercept Shift)
Sep, 2020
Oct, 2020
Nov, 2020
Dec, 2020
Jan, 2021
Med Abs Err
Med Err
Weeks Forecasting
All Model Versions
Youyang Gu
All Cumulative Errors
Month

South Carolina

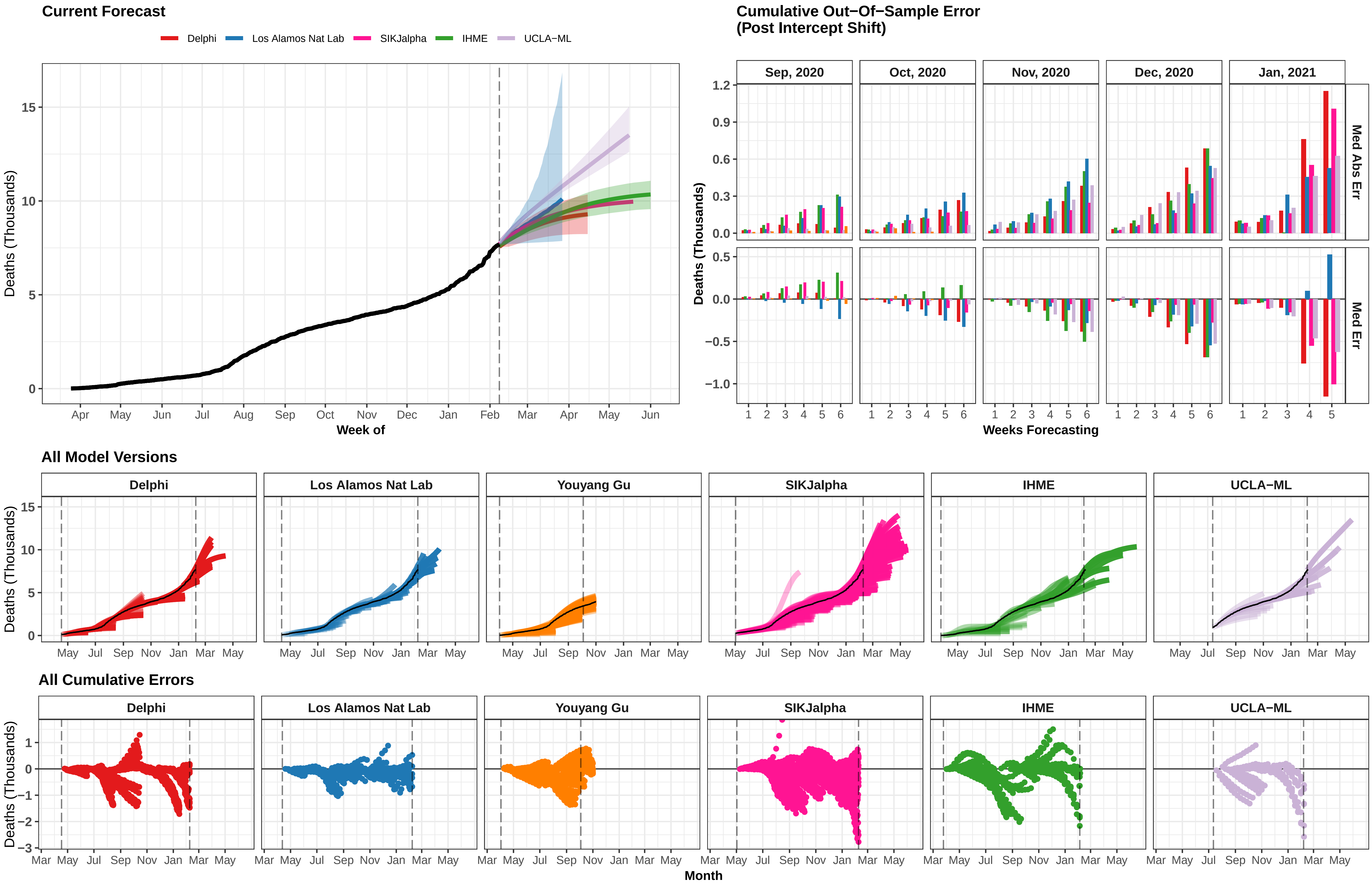

Missouri

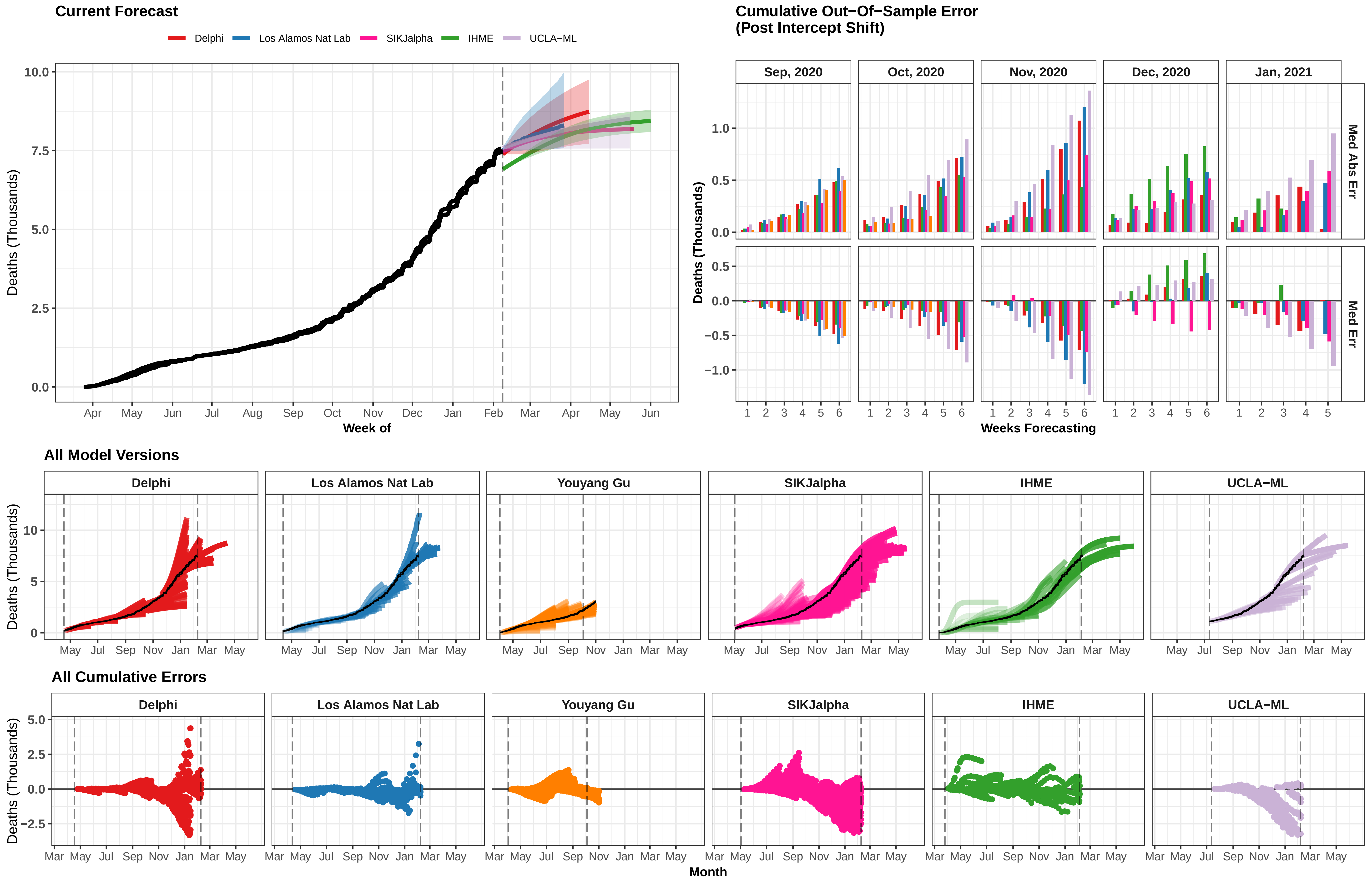
Current Forecast
Delphi
Los Alamos Nat Lab
SIKJalpha
IHME
UCLA−ML
Deaths (Thousands)
Week of
Cumulative Out−Of−Sample Error
(Post Intercept Shift)
Sep, 2020
Oct, 2020
Nov, 2020
Dec, 2020
Jan, 2021
Med Abs Err
Med Err
Weeks Forecasting
All Model Versions
Youyang Gu
All Cumulative Errors
Month

Maryland

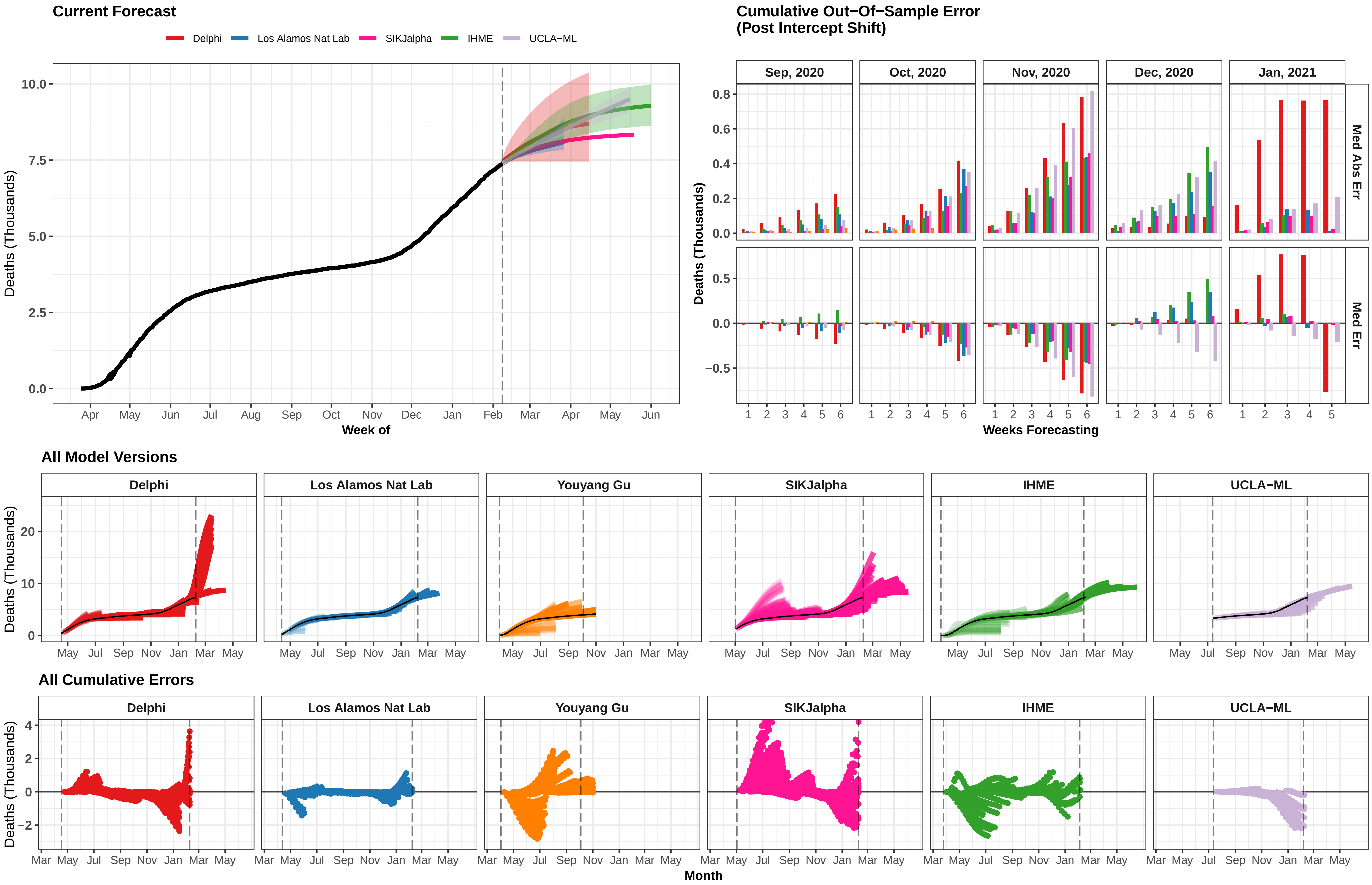

Current Forecast
Delphi
Los Alamos Nat Lab
SIKJalpha
IHME
UCLA−ML
Deaths (Thousands)
Week of
Cumulative Out−Of−Sample Error
(Post Intercept Shift)
Sep, 2020
Oct, 2020
Nov, 2020
Dec, 2020
Jan, 2021
Med Abs Err
Med Err
Weeks Forecasting
All Model Versions
Delphi
Los Alamos Nat Lab
Youyang Gu
SIKJalpha
IHME
UCLA−ML
All Cumulative Errors
Month

Connecticut

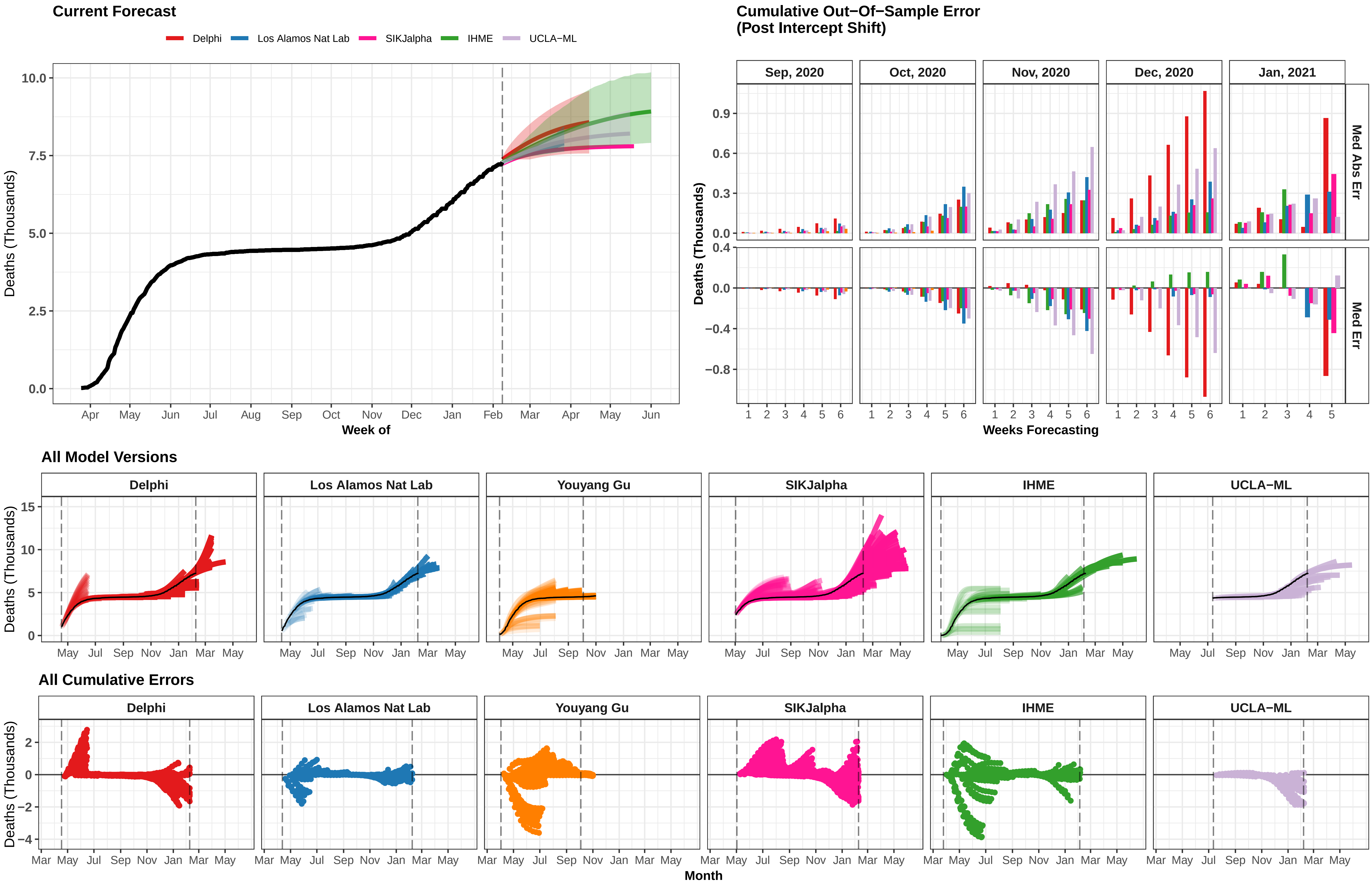

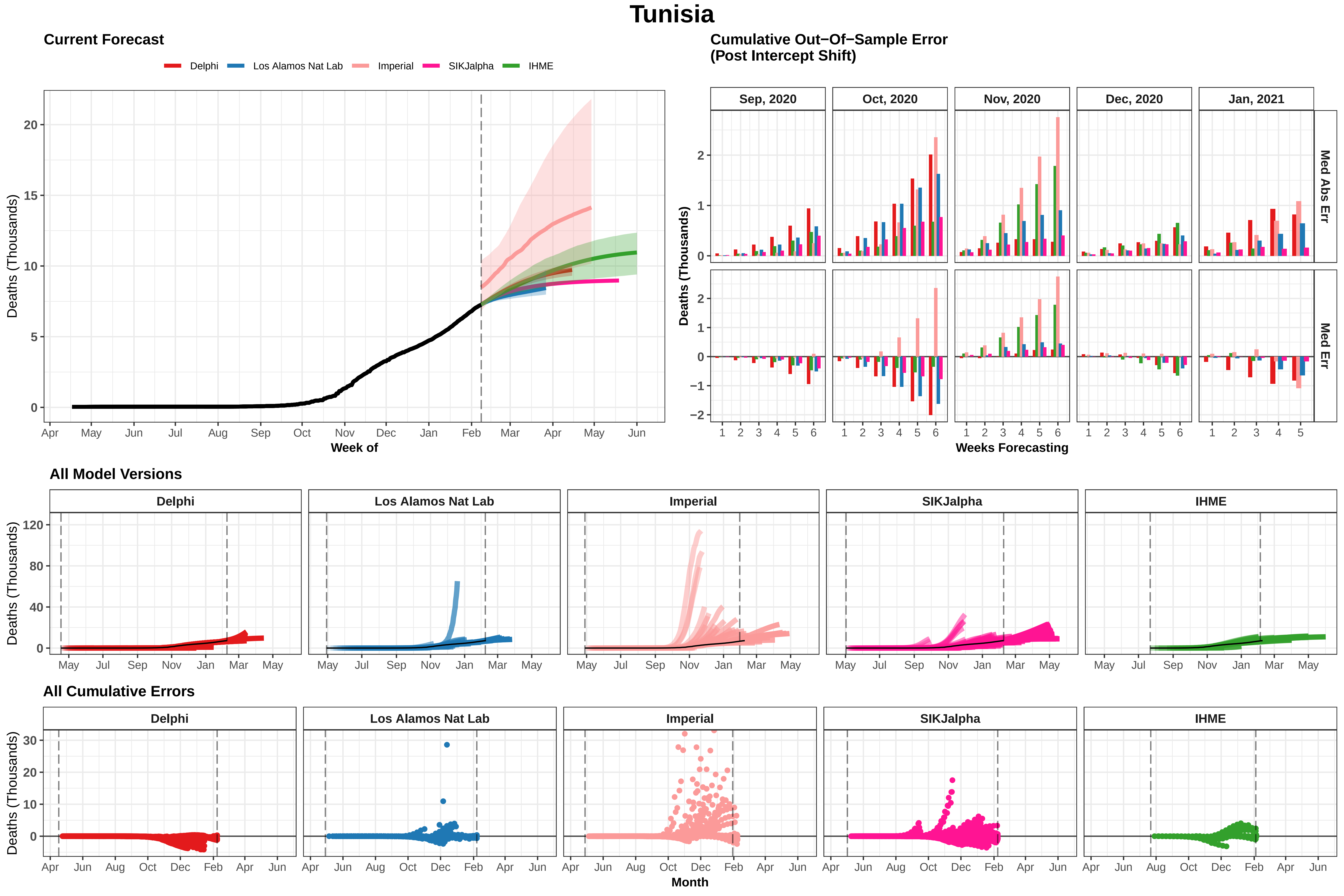

Tunisia
Current Forecast
Delphi
Los Alamos Nat Lab
Imperial
SIKJalpha
IHME
Deaths (Thousands)
Week of
Cumulative Out−Of−Sample Error
(Post Intercept Shift)
Sep, 2020
Oct, 2020
Nov, 2020
Dec, 2020
Jan, 2021
Med Abs Err
Med Err
Weeks Forecasting
All Model Versions
All Cumulative Errors
Month

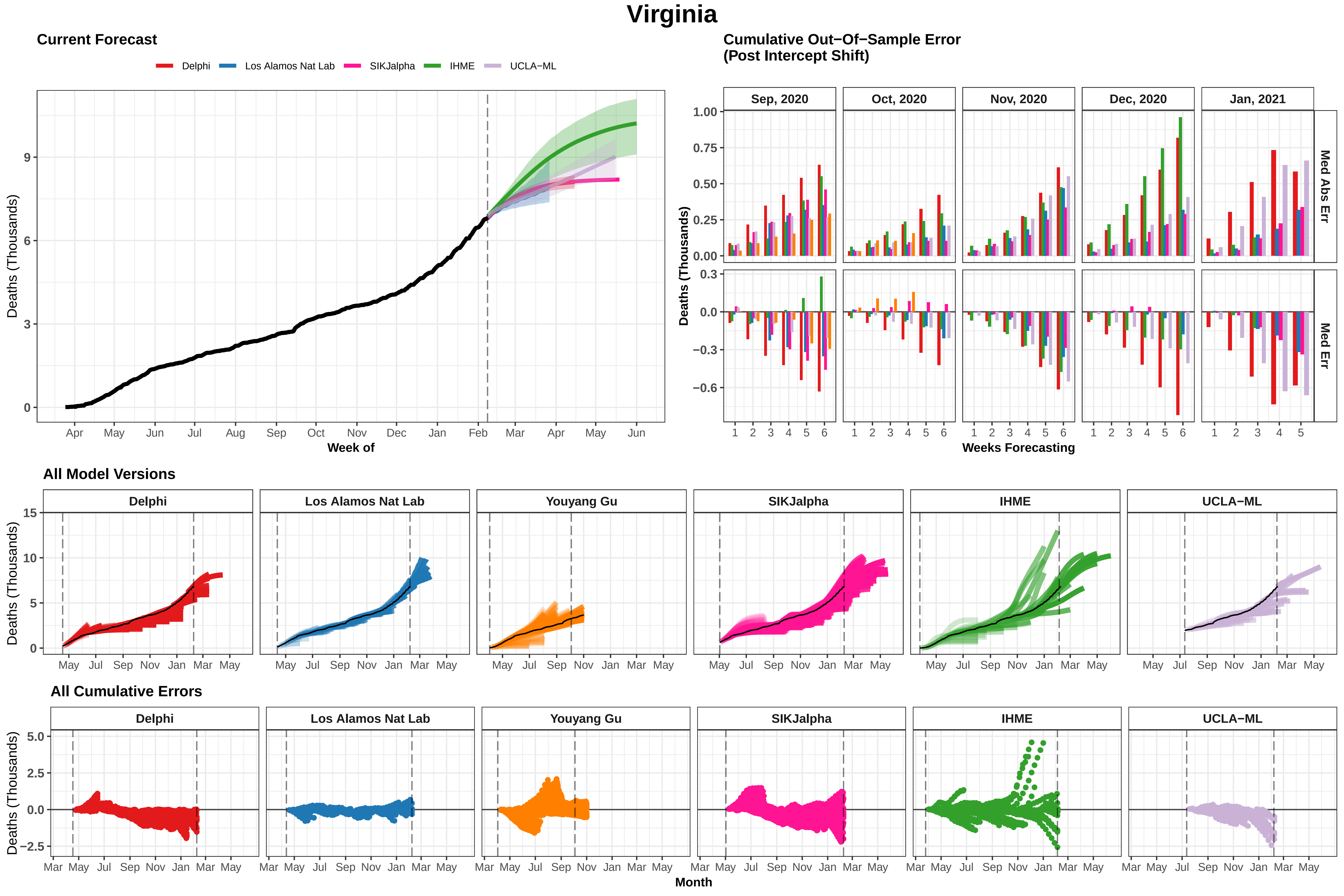

Virginia
Current Forecast
Delphi
Los Alamos Nat Lab
SIKJalpha
IHME
UCLA−ML
Deaths (Thousands)
Week of
Cumulative Out−Of−Sample Error
(Post Intercept Shift)
Sep, 2020
Oct, 2020
Nov, 2020
Dec, 2020
Jan, 2021
Med Abs Err
Med Err
Weeks Forecasting
All Model Versions
Delphi
Los Alamos Nat Lab
Youyang Gu
SIKJalpha
IHME
UCLA−ML
All Cumulative Errors
Month

Wisconsin

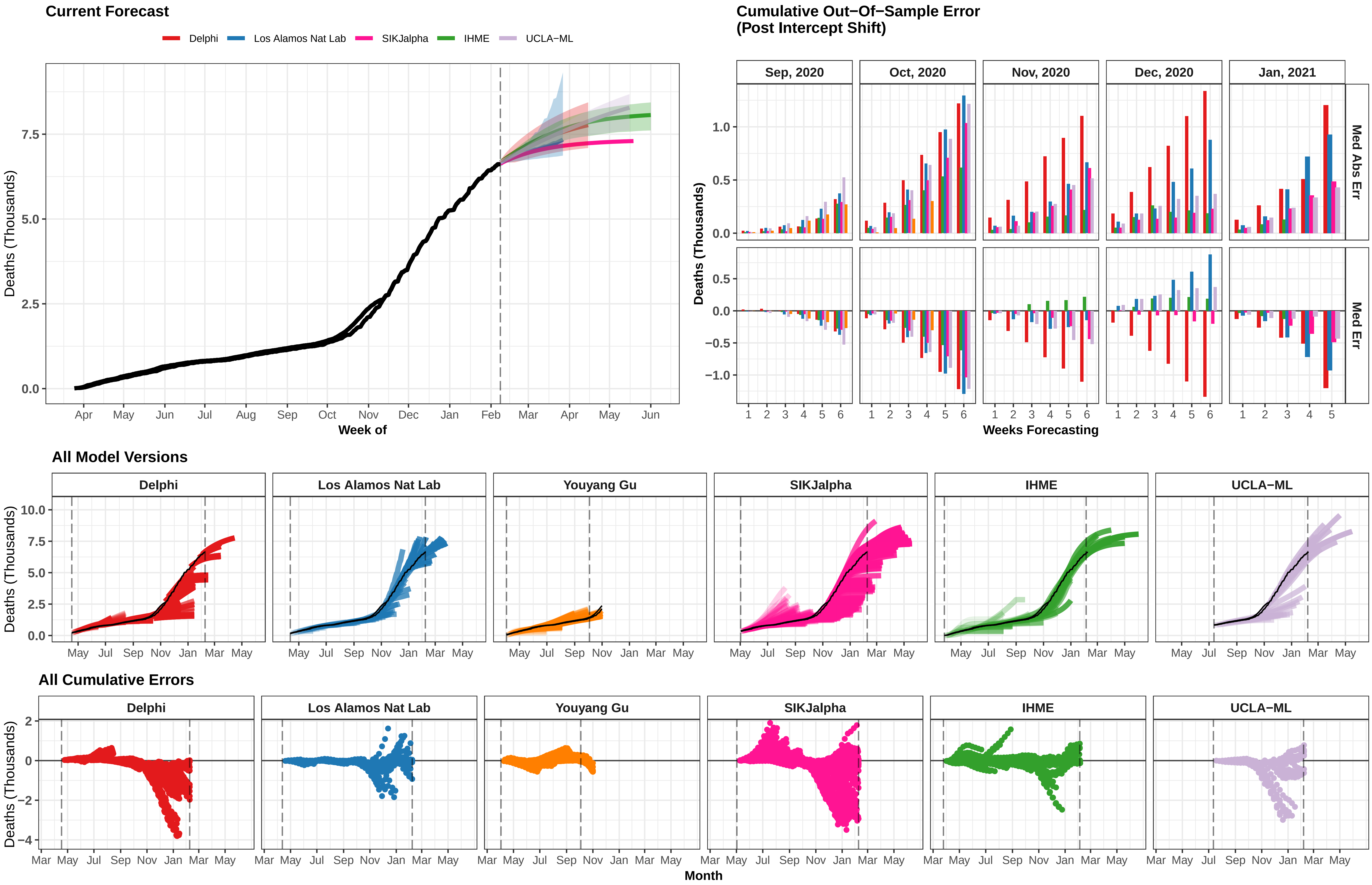
Current Forecast
Delphi
Los Alamos Nat Lab
SIKJalpha
IHME
UCLA−ML
Deaths (Thousands)
Week of
Apr
May
Jun
Jul
Aug
Sep
Oct
Nov
Dec
Jan
Feb
Mar
Cumulative Out−Of−Sample Error
(Post Intercept Shift)
Sep, 2020
Oct, 2020
Nov, 2020
Dec, 2020
Jan, 2021
Med Abs Err
Med Err
Weeks Forecasting
All Model Versions
Delphi
Los Alamos Nat Lab
Youyang Gu
SIKJalpha
IHME
UCLA−ML
All Cumulative Errors
Month

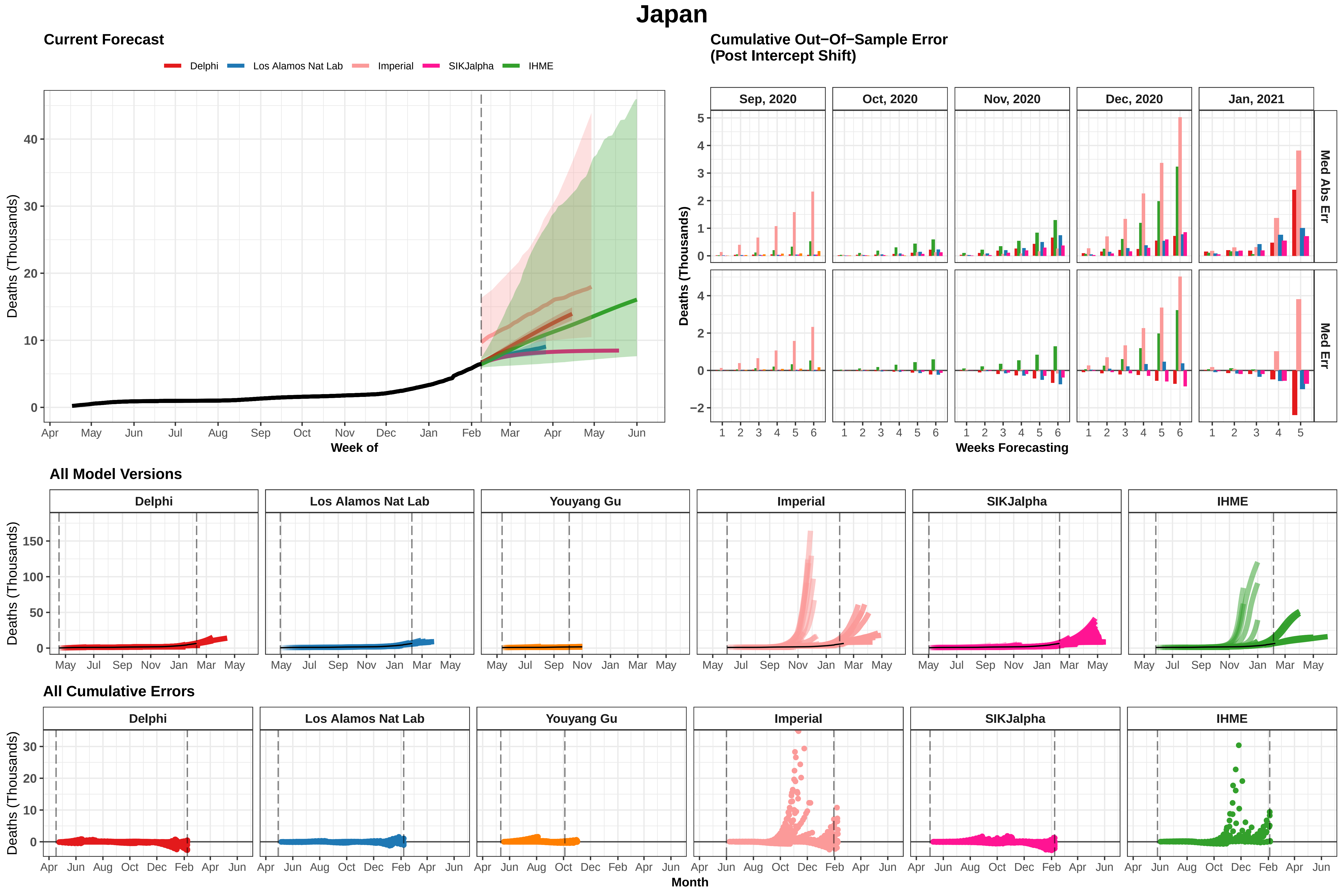

Japan
Current Forecast
Delphi
Los Alamos Nat Lab
Imperial
SIKJalpha
IHME
Deaths (Thousands)
Week of
Cumulative Out−Of−Sample Error
(Post Intercept Shift)
Sep, 2020
Oct, 2020
Nov, 2020
Dec, 2020
Jan, 2021
Med Abs Err
Med Err
Weeks Forecasting
All Model Versions
Delphi
Los Alamos Nat Lab
Youyang Gu
Imperial
SIKJalpha
IHME
All Cumulative Errors
Month

Saudi Arabia

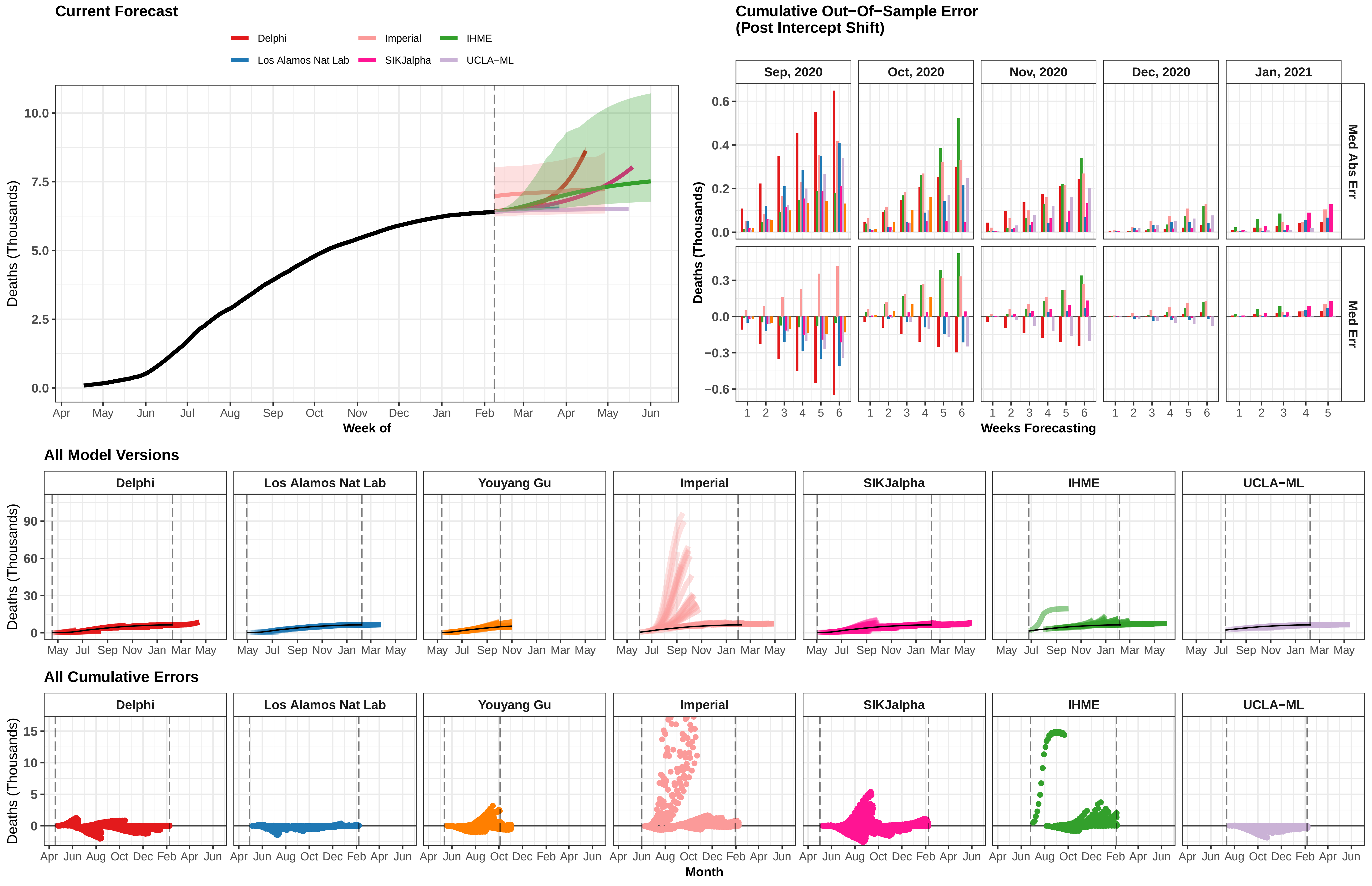

Minnesota

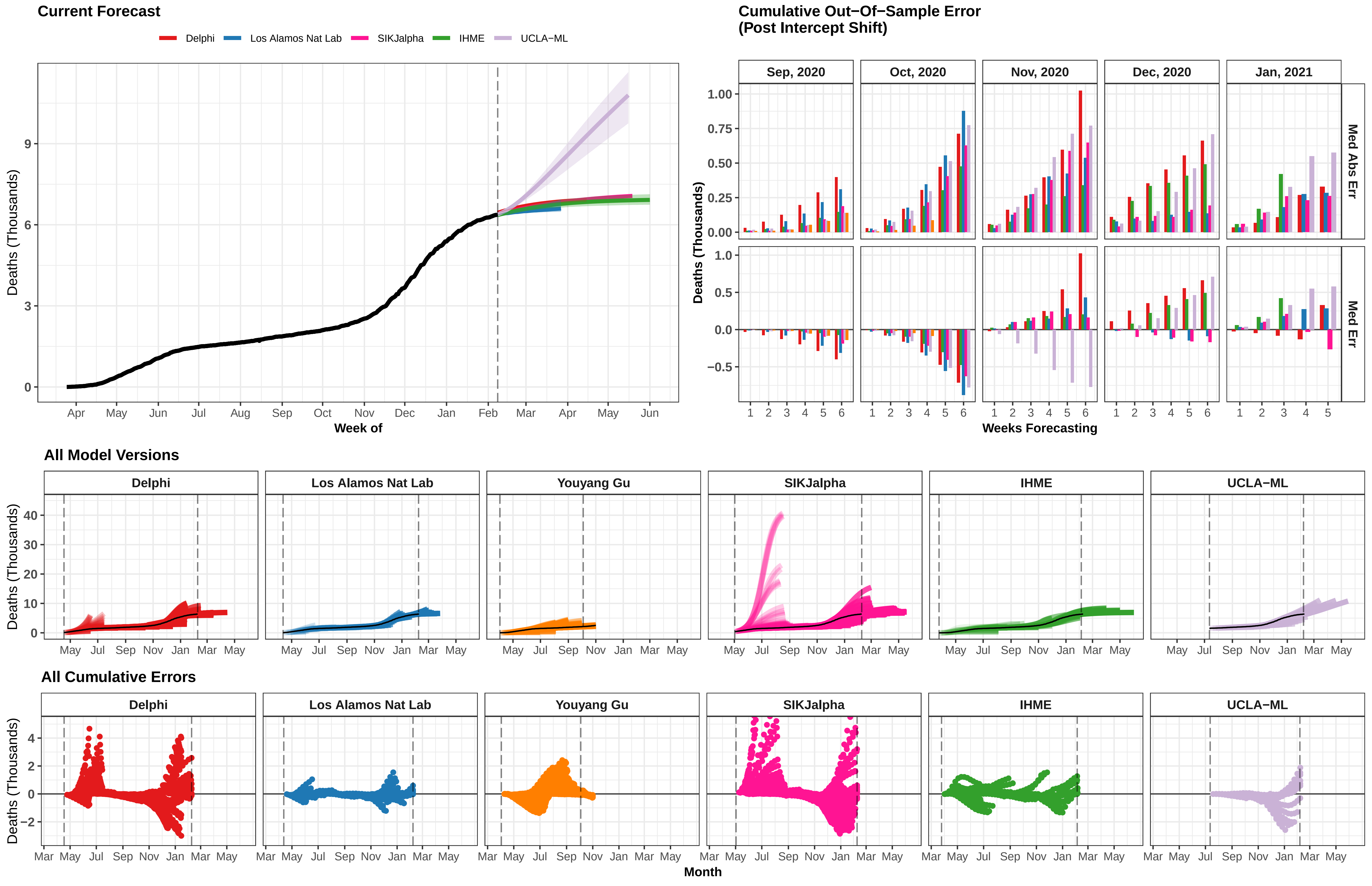

Current Forecast
Delphi
Los Alamos Nat Lab
SIKJalpha
IHME
UCLA−ML
Deaths (Thousands)
Week of
Apr May Jun Jul Aug Sep Oct Nov Dec Jan Feb Mar Apr May Jun
Cumulative Out−Of−Sample Error
(Post Intercept Shift)
Sep, 2020
Oct, 2020
Nov, 2020
Dec, 2020
Jan, 2021
Med Abs Err
Med Err
Weeks Forecasting
All Model Versions
Delphi
Los Alamos Nat Lab
Youyang Gu
SIKJalpha
IHME
UCLA−ML
All Cumulative Errors
Month

Mississippi

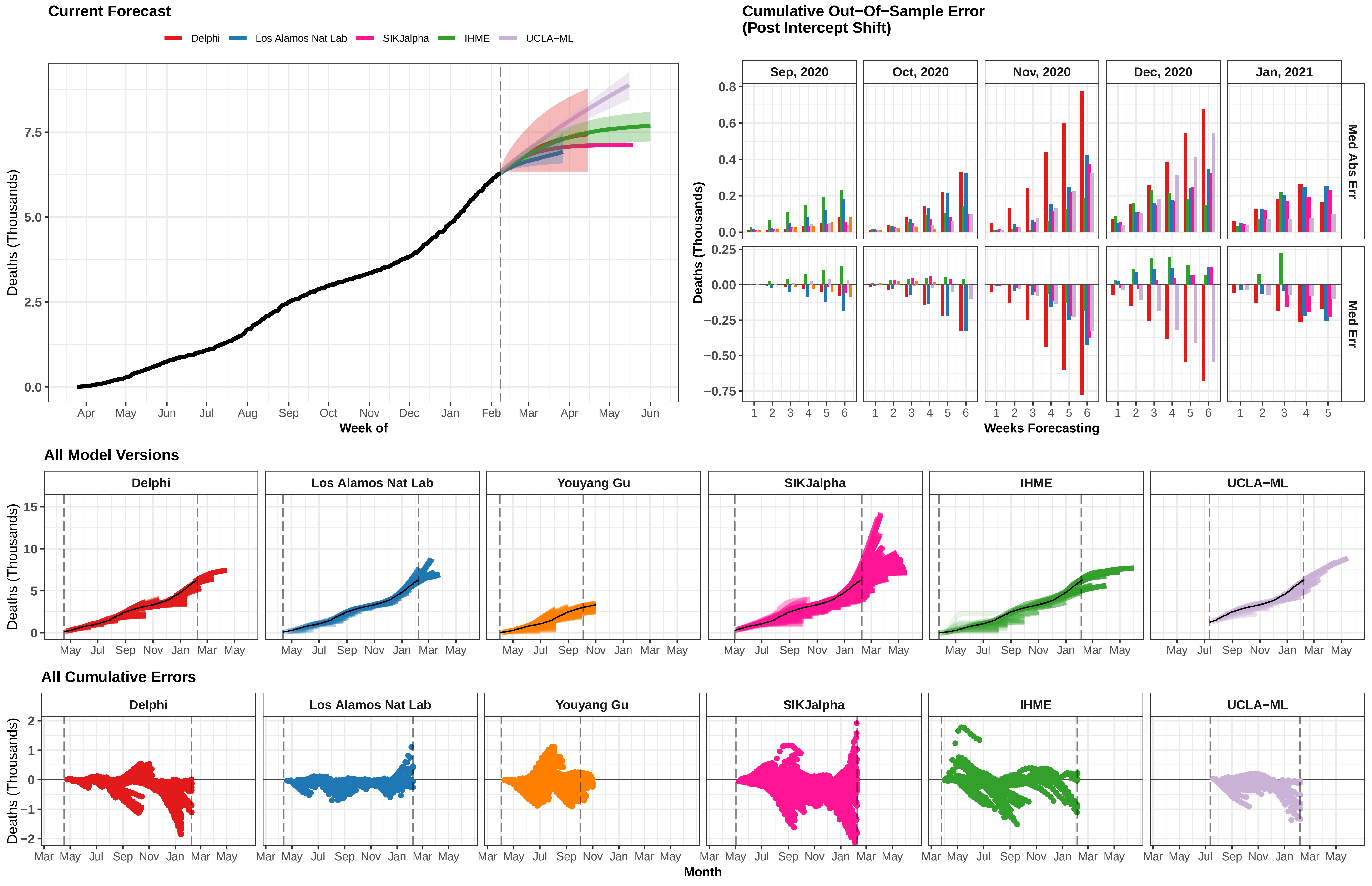

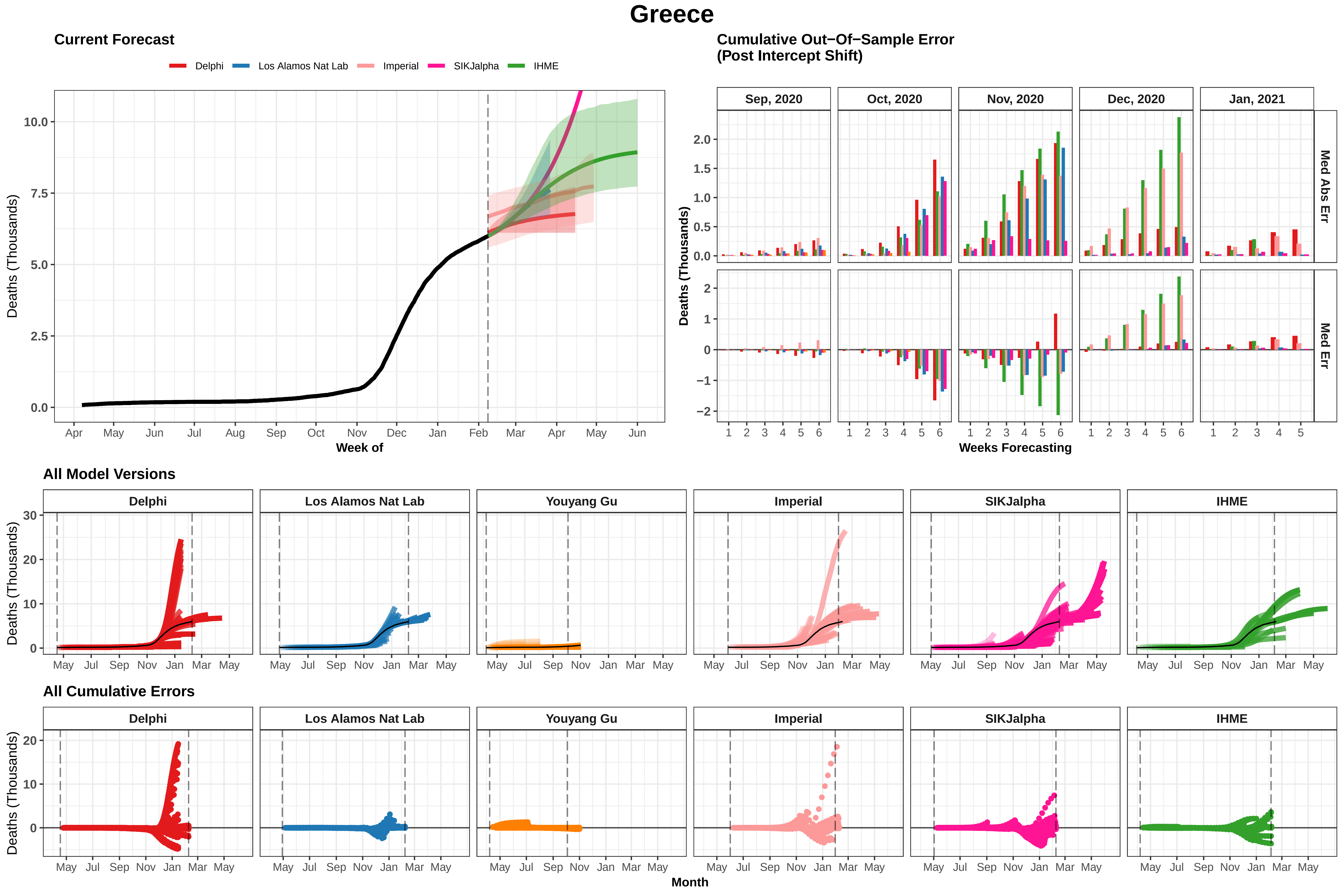
Greece
Current Forecast
Delphi
Los Alamos Nat Lab
Imperial
SIKJalpha
IHME
Deaths (Thousands)
Week of
Cumulative Out−Of−Sample Error
(Post Intercept Shift)
Sep, 2020
Oct, 2020
Nov, 2020
Dec, 2020
Jan, 2021
Med Abs Err
Med Err
Weeks Forecasting
All Model Versions
Youyang Gu
All Cumulative Errors
Month

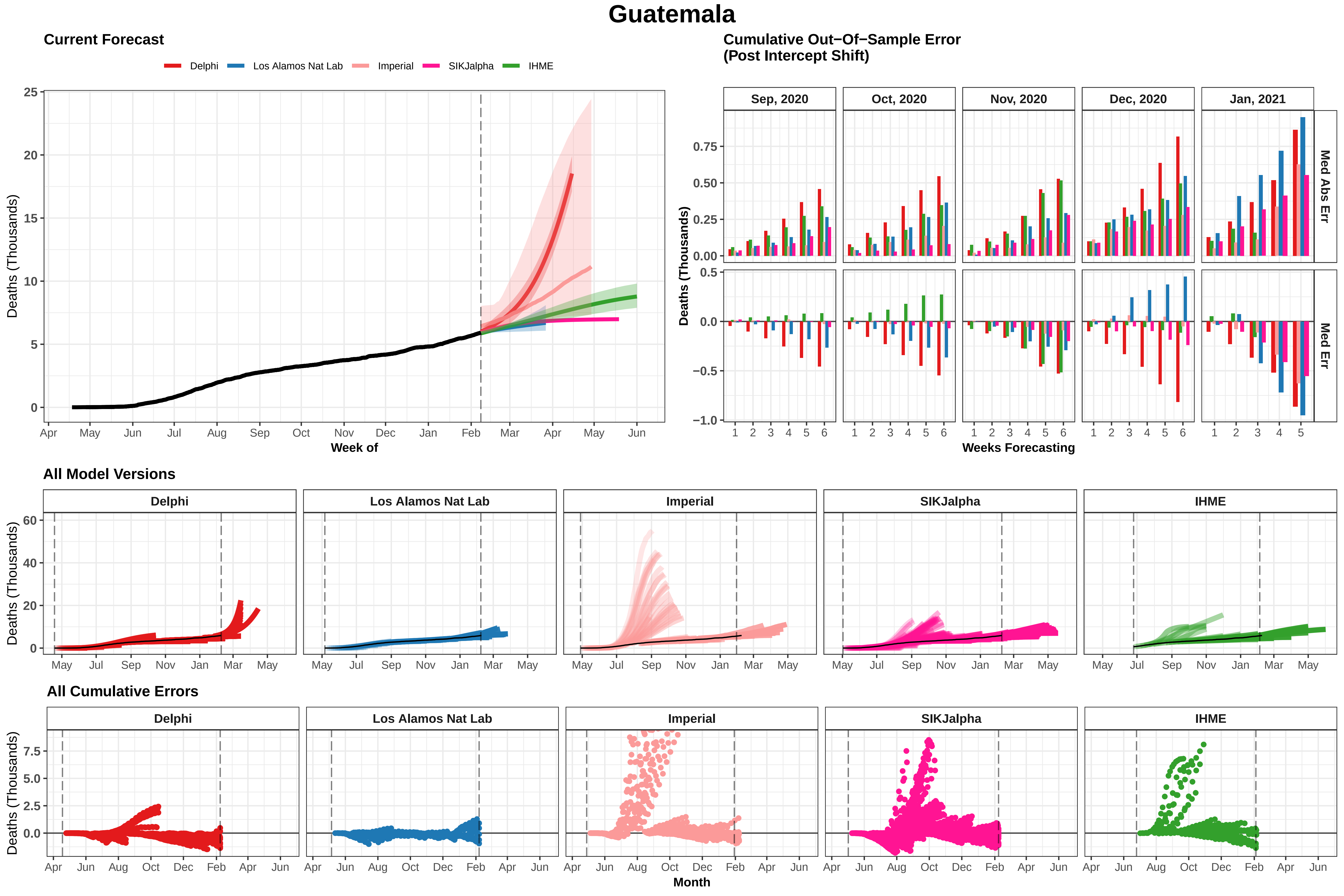
Guatemala
Current Forecast
Delphi
Los Alamos Nat Lab
Imperial
SIKJalpha
IHME
Deaths (Thousands)
Week of
Cumulative Out−Of−Sample Error
(Post Intercept Shift)
Sep, 2020
Oct, 2020
Nov, 2020
Dec, 2020
Jan, 2021
Med Abs Err
Med Err
Weeks Forecasting
All Model Versions
All Cumulative Errors
Month

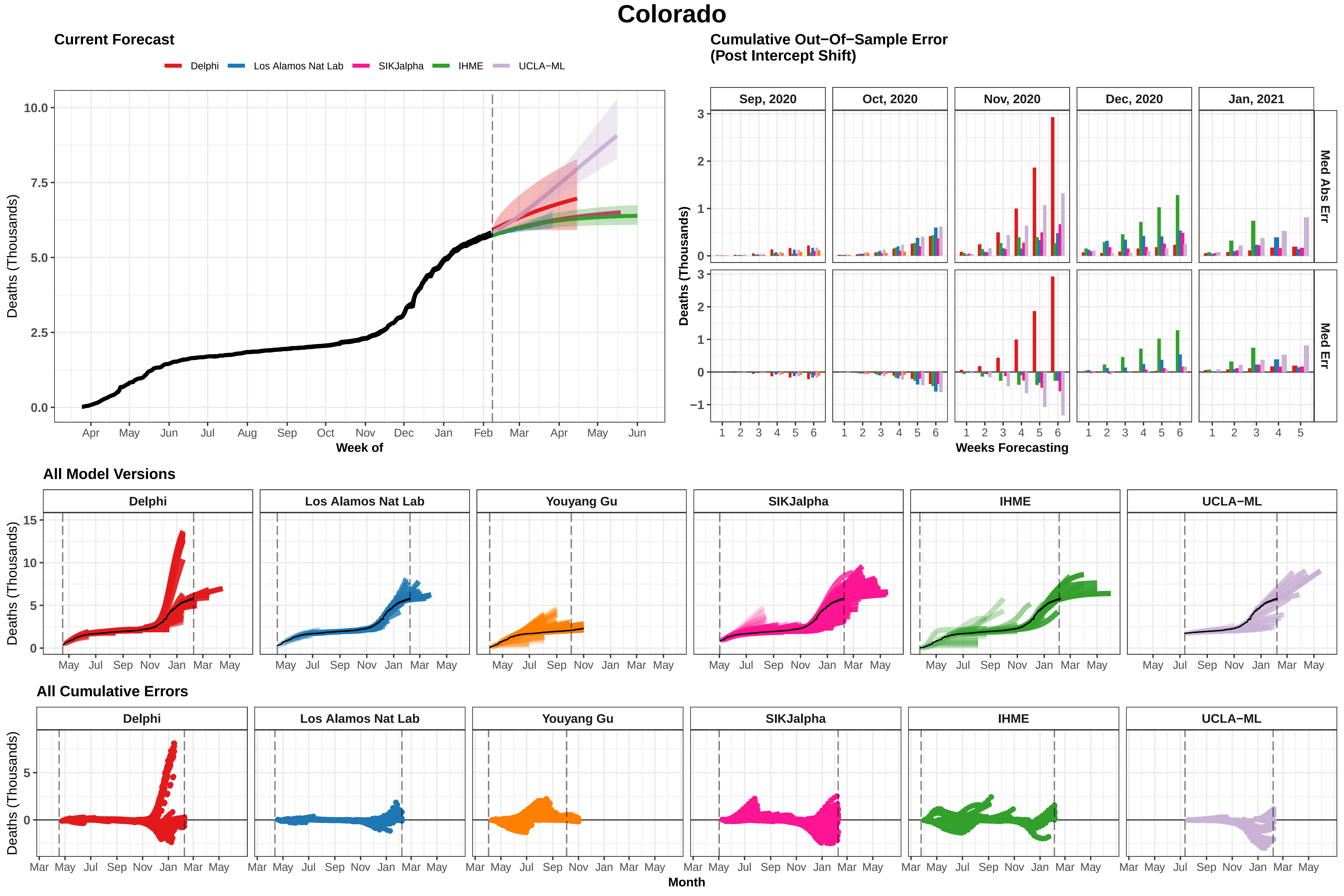

Colorado
Current Forecast
Delphi
Los Alamos Nat Lab
SIKJalpha
IHME
UCLA−ML
Deaths (Thousands)
Week of
Cumulative Out−Of−Sample Error
(Post Intercept Shift)
Sep, 2020
Oct, 2020
Nov, 2020
Dec, 2020
Jan, 2021
Med Abs Err
Med Err
Weeks Forecasting
All Model Versions
Delphi
Los Alamos Nat Lab
Youyang Gu
SIKJalpha
IHME
UCLA−ML
All Cumulative Errors
Month

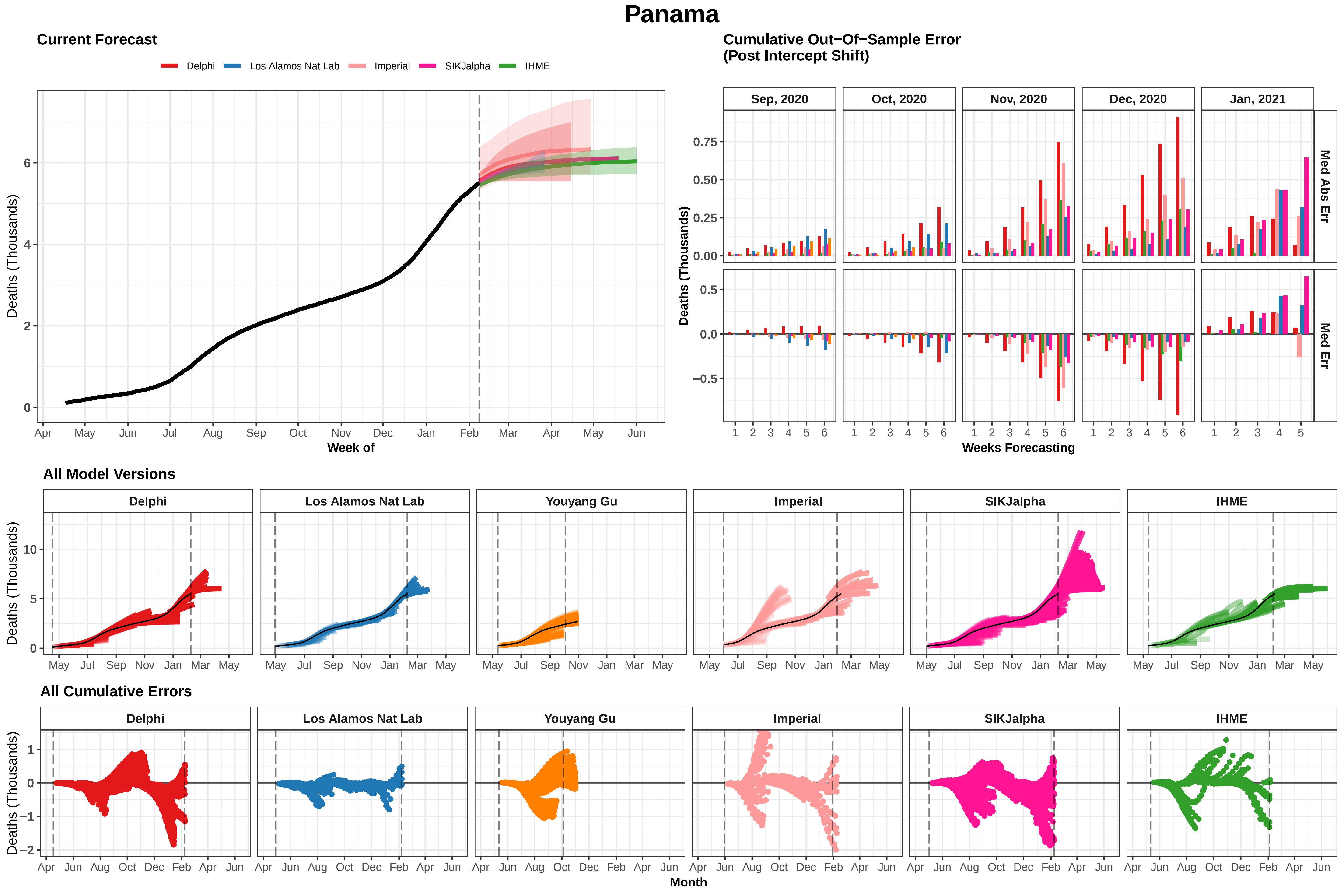
Panama
Current Forecast
Delphi
Los Alamos Nat Lab
Imperial
SIKJalpha
IHME
Deaths (Thousands)
Week of
Cumulative Out−Of−Sample Error
(Post Intercept Shift)
Sep, 2020
Oct, 2020
Nov, 2020
Dec, 2020
Jan, 2021
Med Abs Err
Med Err
Weeks Forecasting
All Model Versions
Youyang Gu
All Cumulative Errors
Month

Slovakia

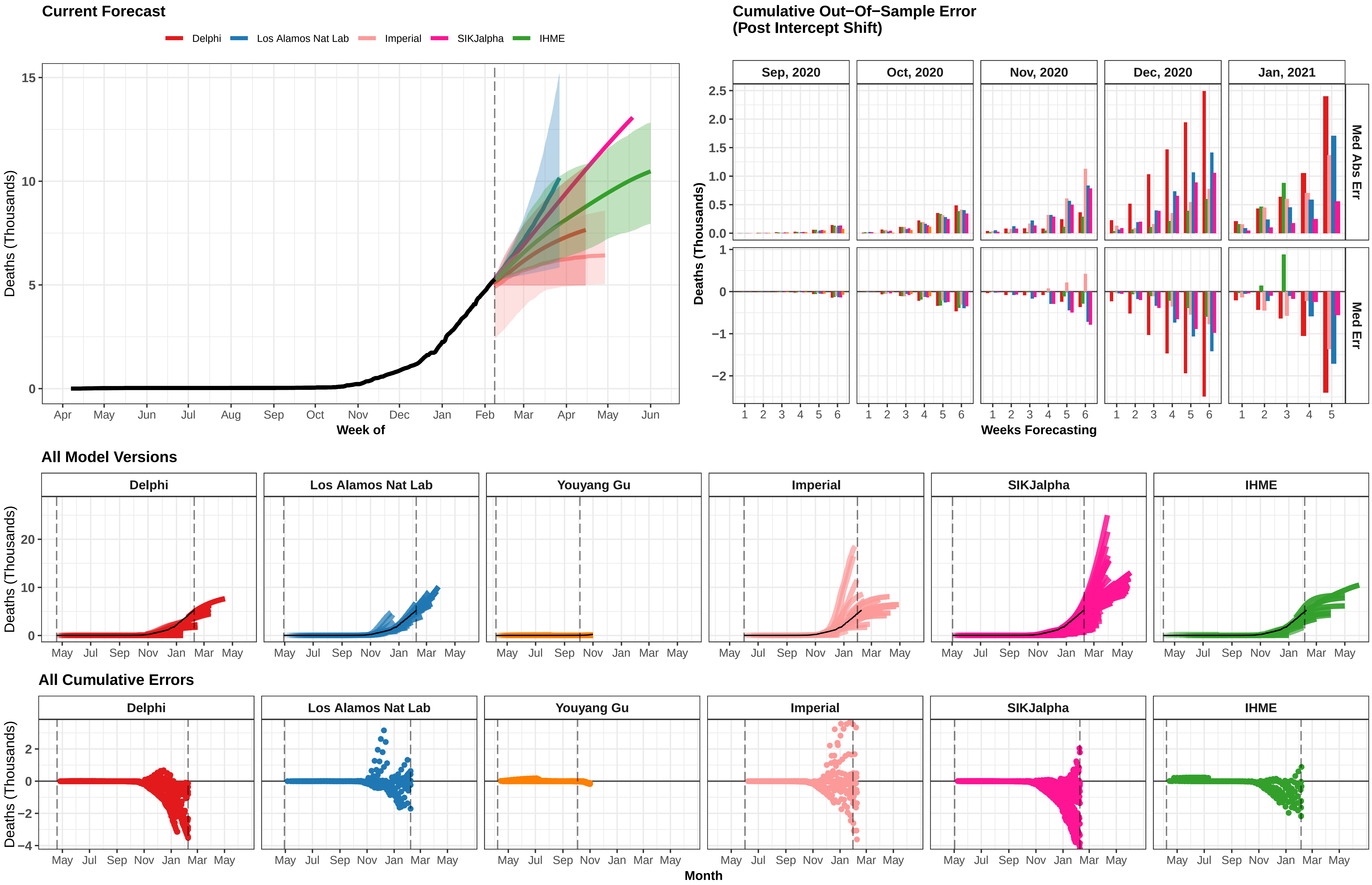
Current Forecast
Delphi
Los Alamos Nat Lab
Imperial
SIKJalpha
IHME
Deaths (Thousands)
15
10
5
0
Apr May Jun Jul Aug Sep Oct Nov Dec Jan Feb Mar Apr May Jun
Week of
Cumulative Out−Of−Sample Error
(Post Intercept Shift)
Sep, 2020
Oct, 2020
Nov, 2020
Dec, 2020
Jan, 2021
Med Abs Err
Med Err
2.5
2.0
1.5
1.0
0.5
0.0
1
0
−1
−2
1 2 3 4 5 6
1 2 3 4 5
Weeks Forecasting
All Model Versions
Delphi
Los Alamos Nat Lab
Youyang Gu
Imperial
SIKJalpha
IHME
20
10
0
May Jul Sep Nov Jan Mar May
All Cumulative Errors
2
0
−2
−4
Month

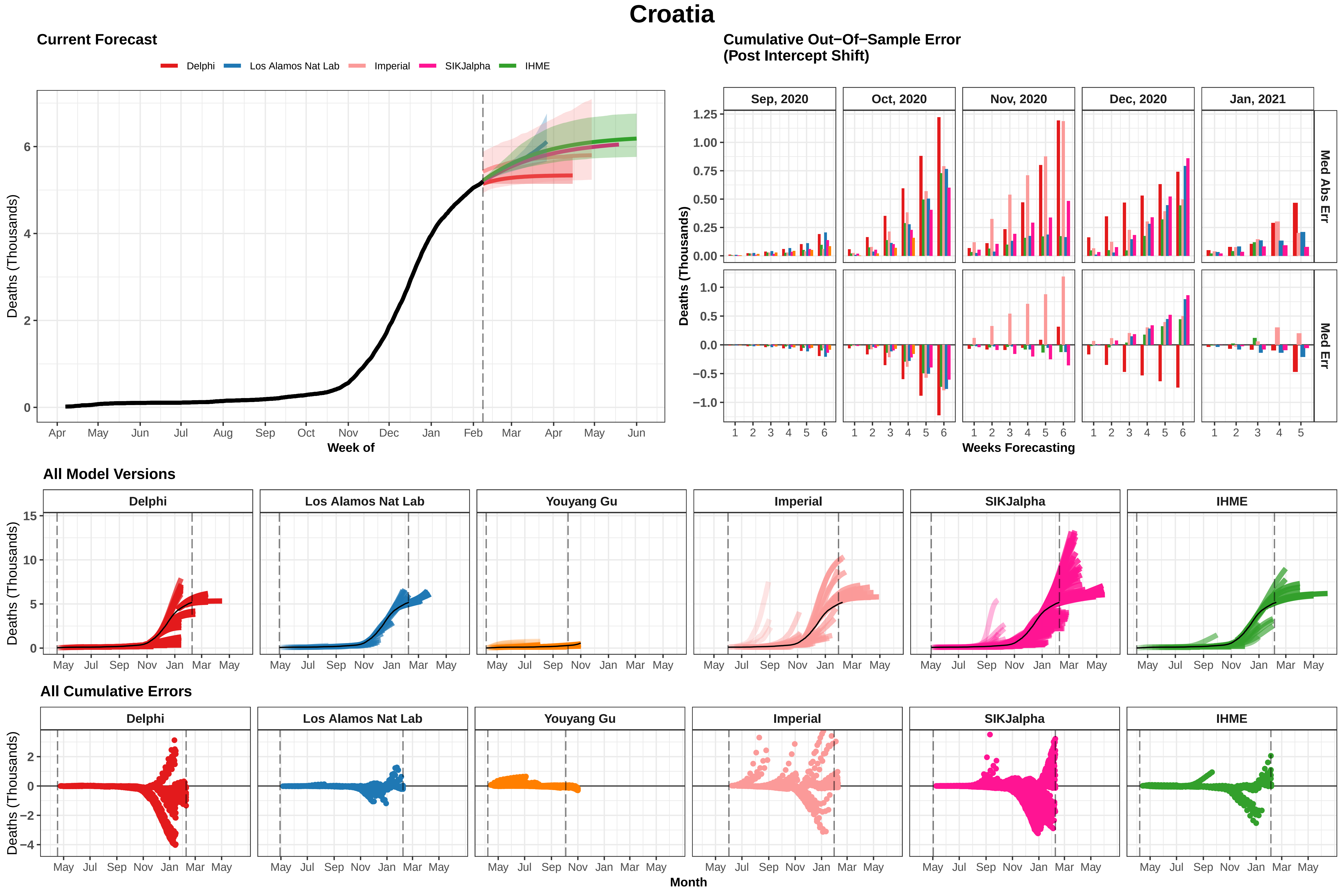

Croatia
Current Forecast
Delphi
Los Alamos Nat Lab
Imperial
SIKJalpha
IHME
Deaths (Thousands)
Week of
Cumulative Out−Of−Sample Error
(Post Intercept Shift)
Sep, 2020
Oct, 2020
Nov, 2020
Dec, 2020
Jan, 2021
Med Abs Err
Med Err
Weeks Forecasting
All Model Versions
Delphi
Los Alamos Nat Lab
Youyang Gu
Imperial
SIKJalpha
IHME
All Cumulative Errors
Month

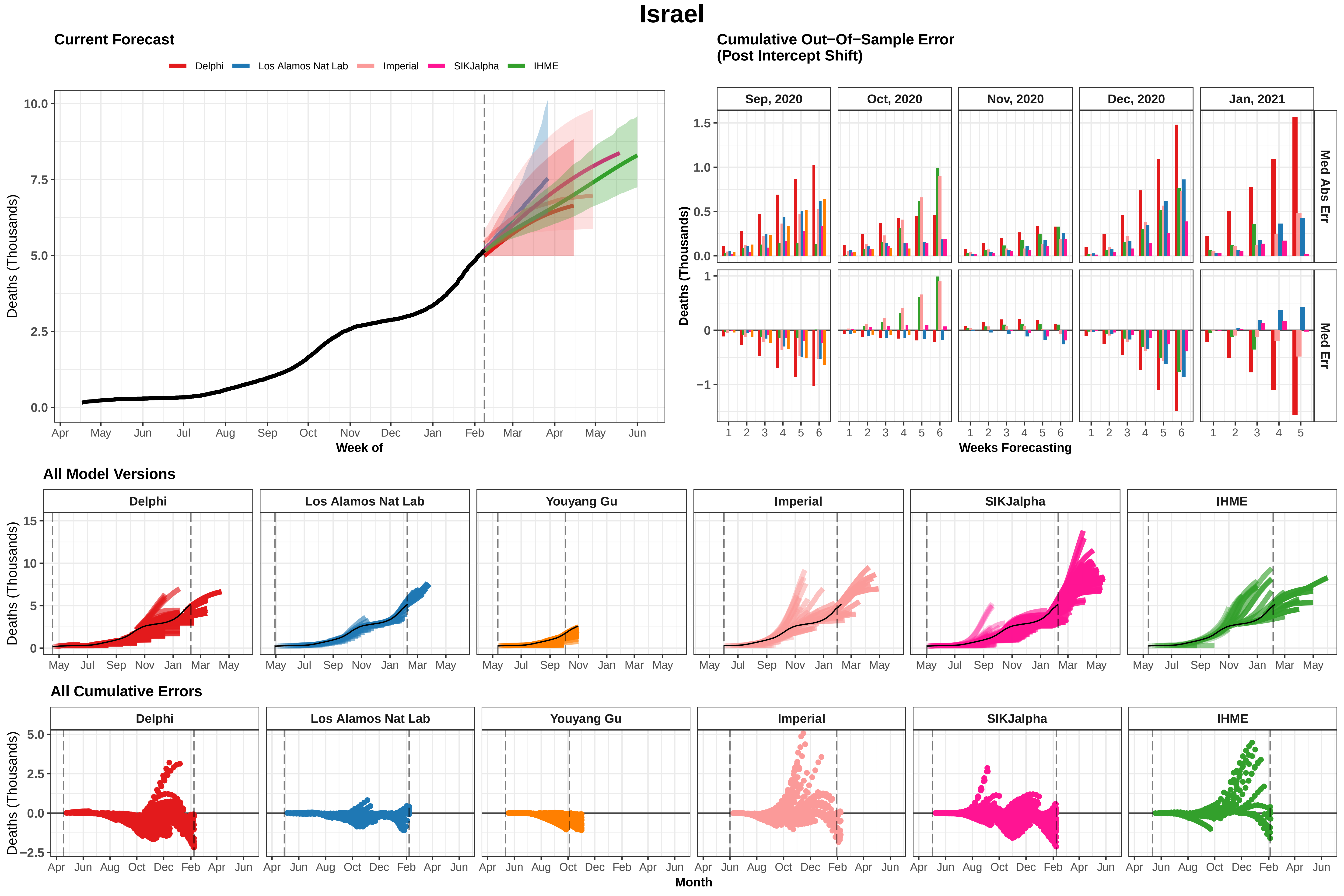
Israel
Current Forecast
Delphi
Los Alamos Nat Lab
Imperial
SIKJalpha
IHME
Deaths (Thousands)
Week of
Cumulative Out−Of−Sample Error
(Post Intercept Shift)
Sep, 2020
Oct, 2020
Nov, 2020
Dec, 2020
Jan, 2021
Med Abs Err
Med Err
Weeks Forecasting
All Model Versions
Delphi
Los Alamos Nat Lab
Youyang Gu
Imperial
SIKJalpha
IHME
All Cumulative Errors
Month

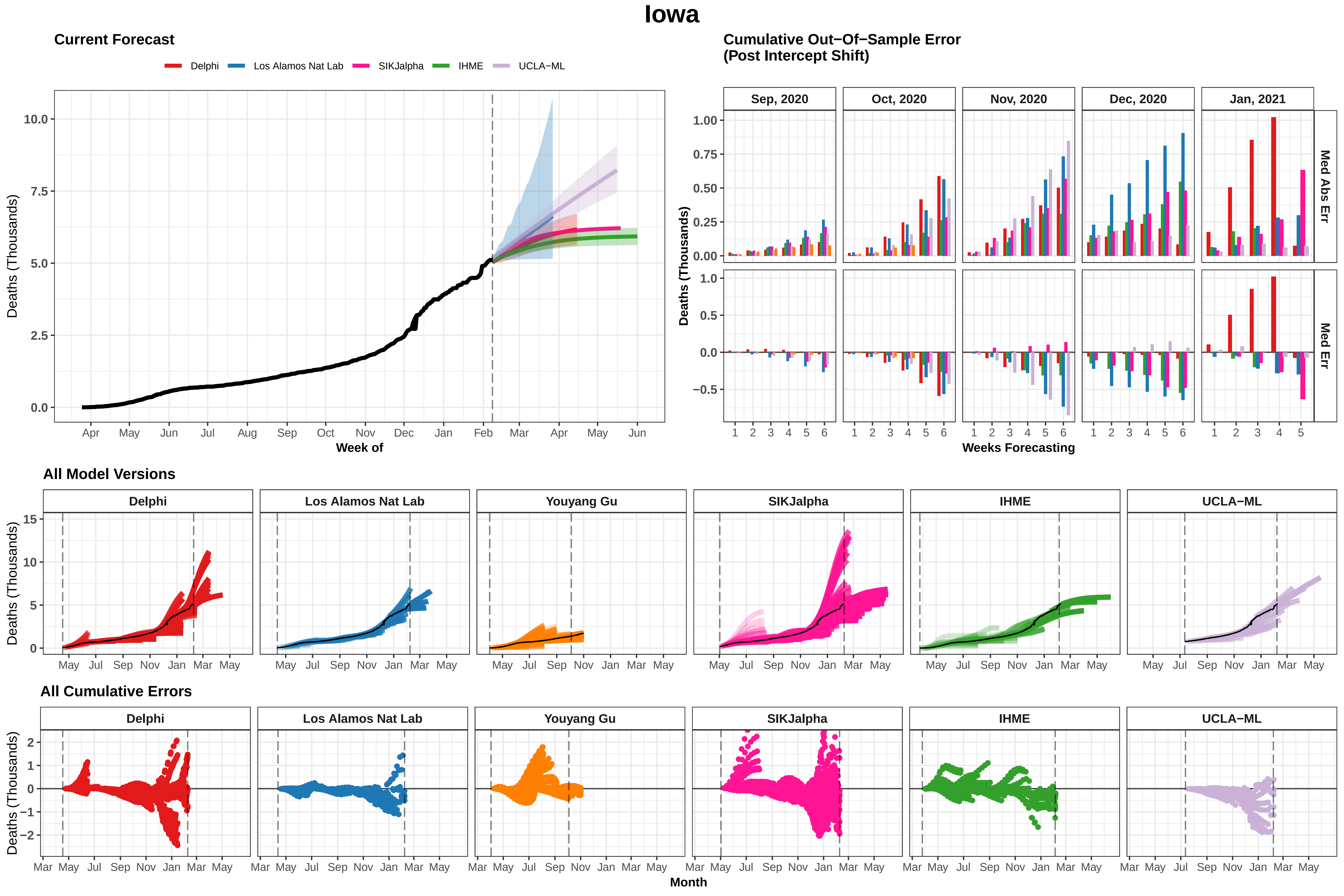
Iowa
Current Forecast
Delphi
Los Alamos Nat Lab
SIKJalpha
IHME
UCLA−ML
Deaths (Thousands)
Week of
Apr May Jun Jul Aug Sep Oct Nov Dec Jan Feb Mar Apr May Jun
Cumulative Out−Of−Sample Error
(Post Intercept Shift)
Sep, 2020
Oct, 2020
Nov, 2020
Dec, 2020
Jan, 2021
Med Abs Err
Med Err
Weeks Forecasting
All Model Versions
Delphi
Los Alamos Nat Lab
Youyang Gu
SIKJalpha
IHME
UCLA−ML
All Cumulative Errors
Month

Arkansas

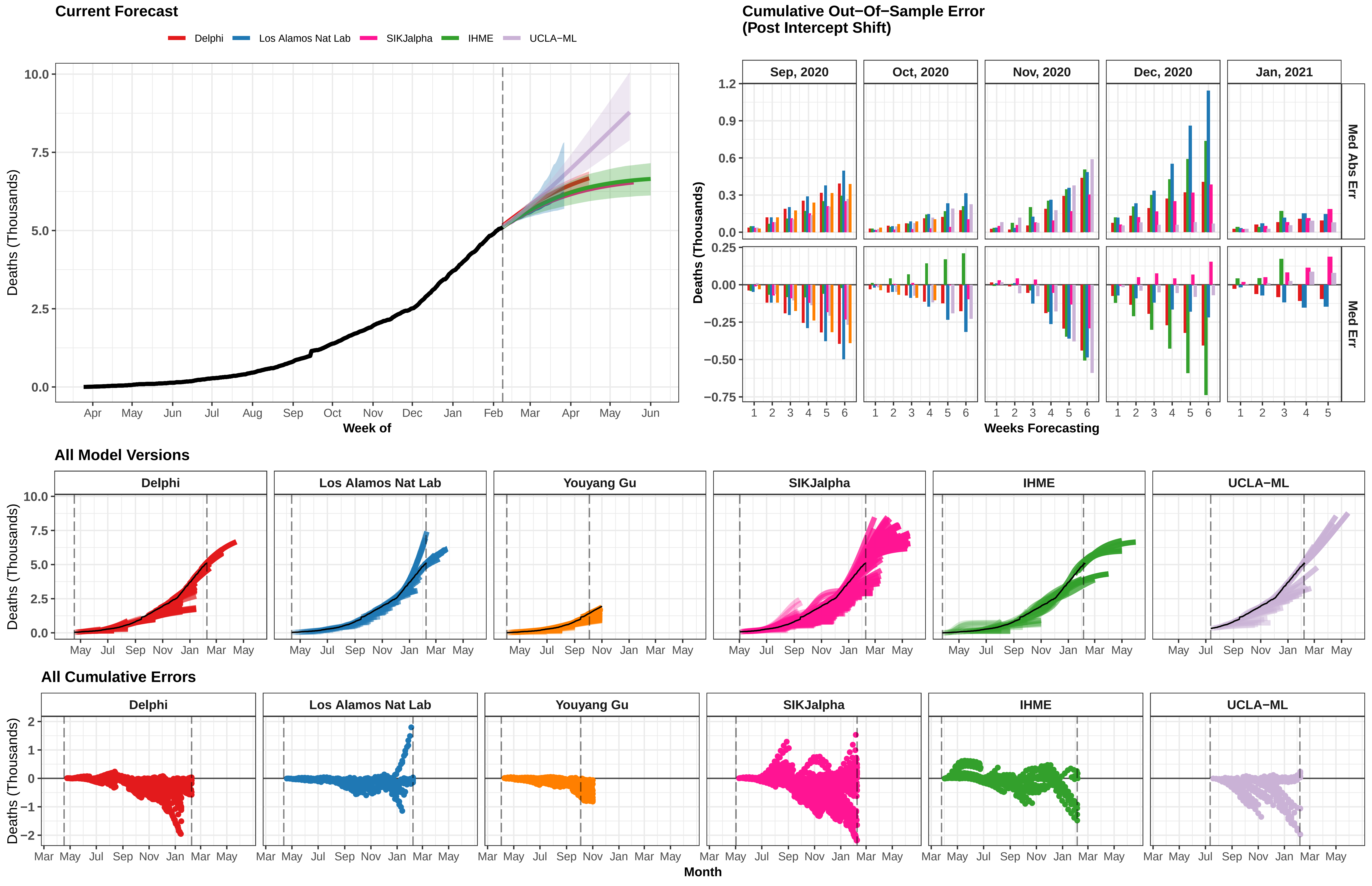
Current Forecast
Delphi
Los Alamos Nat Lab
SIKJalpha
IHME
UCLA−ML
Deaths (Thousands)
Week of
Cumulative Out−Of−Sample Error
(Post Intercept Shift)
Sep, 2020
Oct, 2020
Nov, 2020
Dec, 2020
Jan, 2021
Med Abs Err
Med Err
Weeks Forecasting
All Model Versions
Youyang Gu
All Cumulative Errors
Month

China

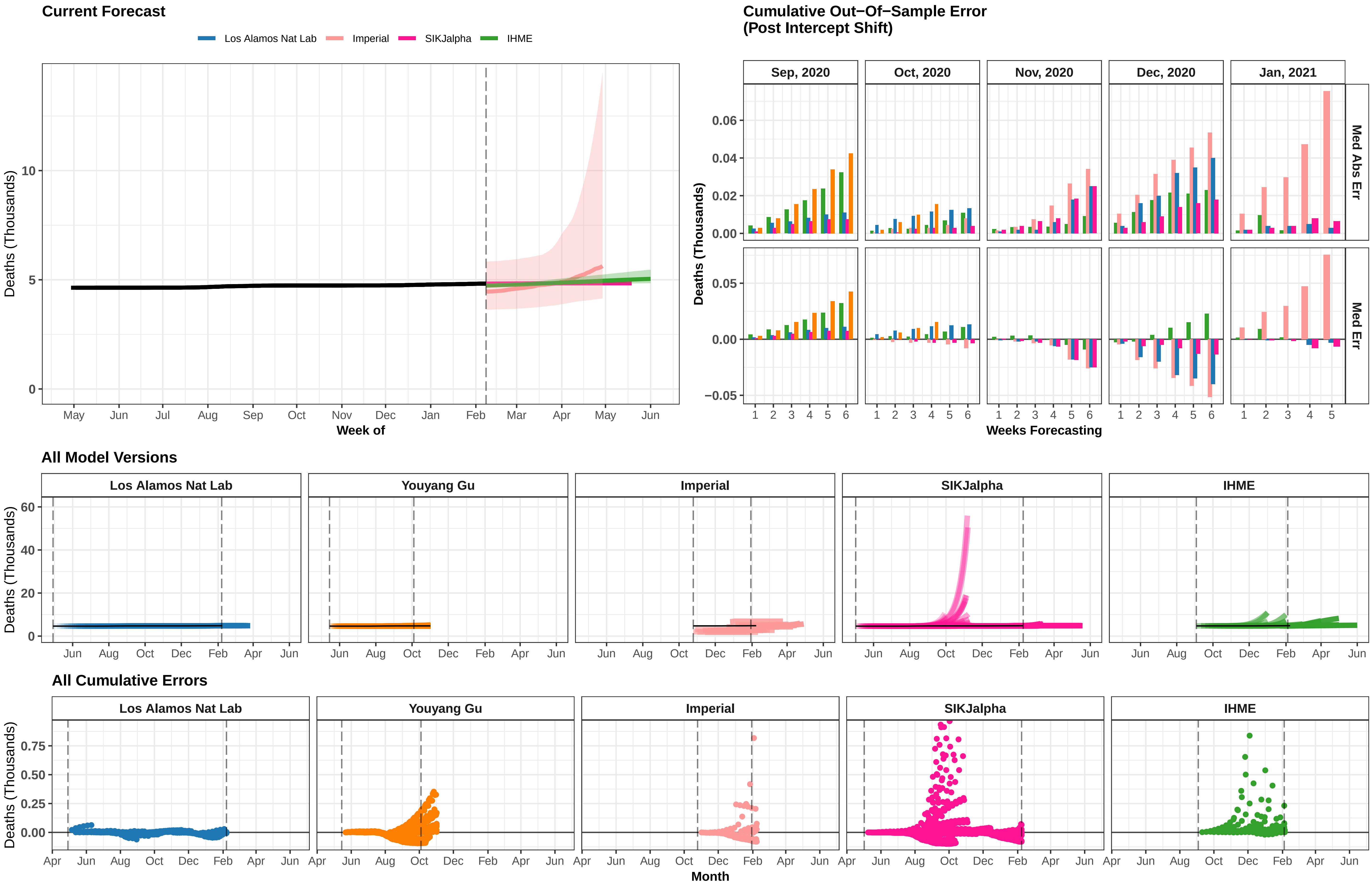

Current Forecast
Los Alamos Nat Lab
Imperial
SIKJalpha
IHME
Deaths (Thousands)
0
5
10
May
Jun
Jul
Aug
Sep
Oct
Nov
Dec
Jan
Feb
Mar
Apr
May
Jun
Week of
Cumulative Out−Of−Sample Error
(Post Intercept Shift)
Sep, 2020
Oct, 2020
Nov, 2020
Dec, 2020
Jan, 2021
Med Abs Err
Med Err
0.00
0.02
0.04
0.06
−0.05
0.00
0.05
1 2 3 4 5 6
1 2 3 4 5
Weeks Forecasting
All Model Versions
Los Alamos Nat Lab
Youyang Gu
Imperial
SIKJalpha
IHME
0
20
40
60
Jun Aug Oct Dec Feb Apr Jun
All Cumulative Errors
0.00
0.25
0.50
0.75
Apr Jun Aug Oct Dec Feb Apr Jun
Month

Bosnia and Herzegovina

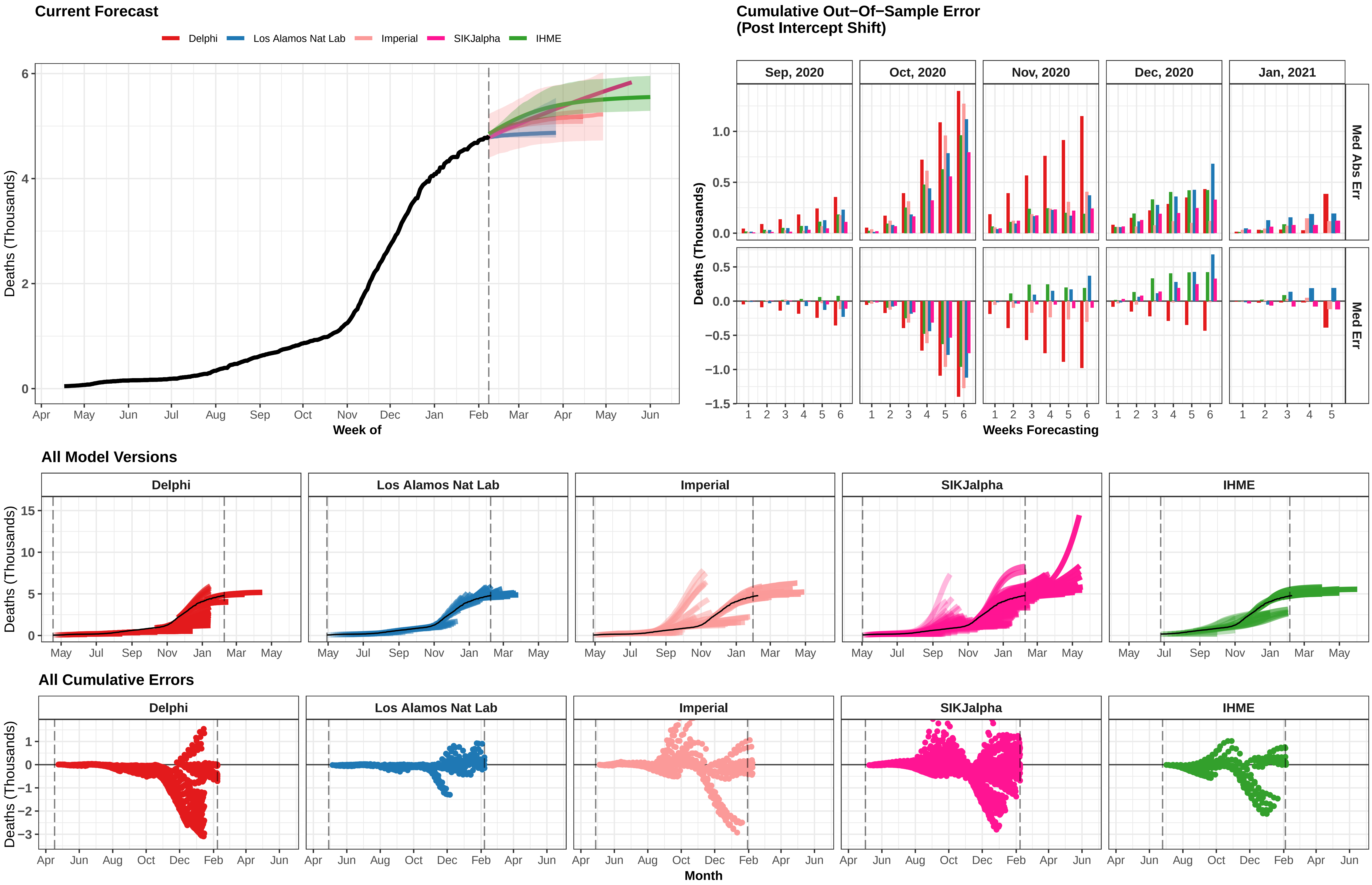

Nevada

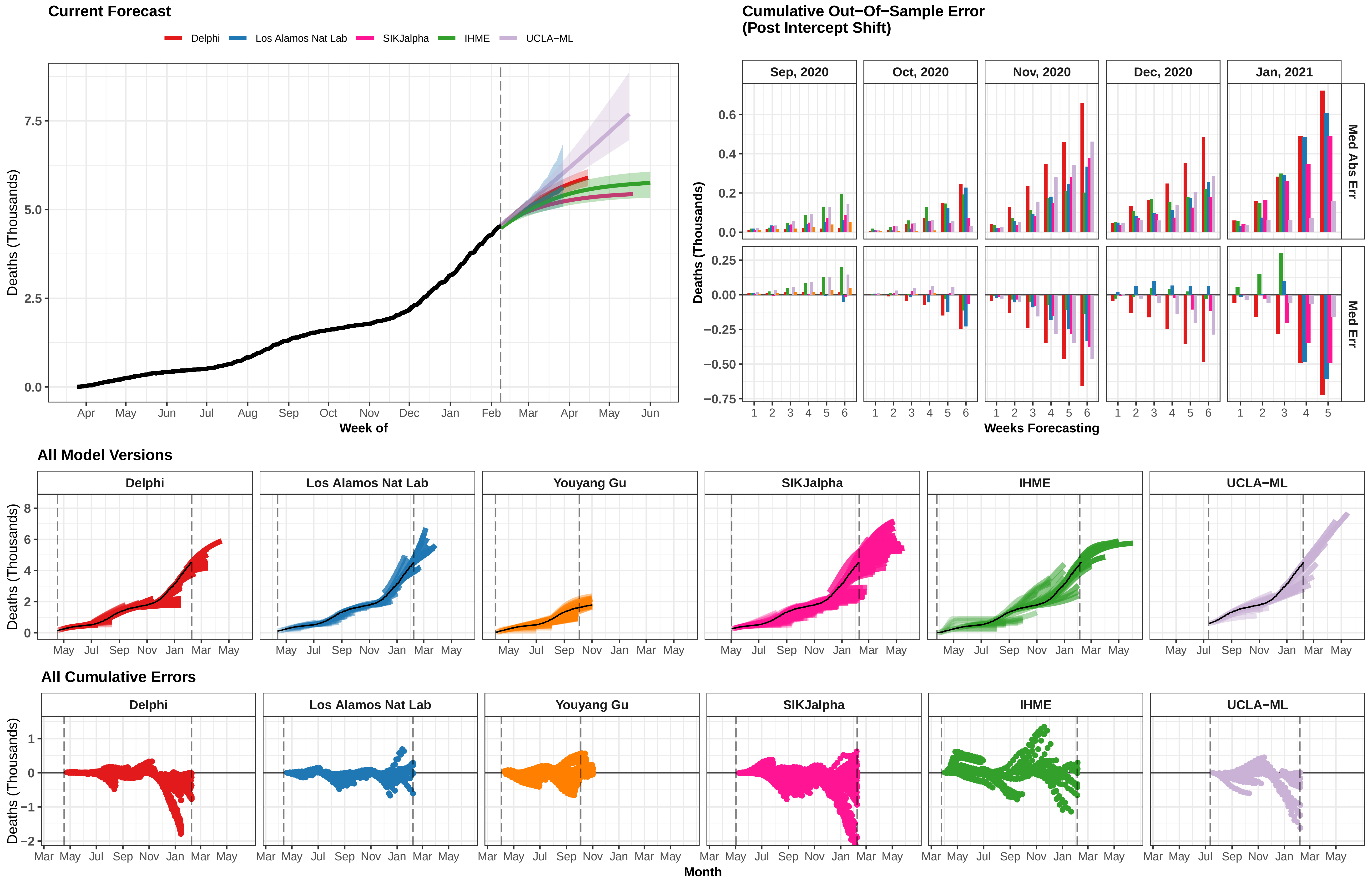

Current Forecast
Delphi
Los Alamos Nat Lab
SIKJalpha
IHME
UCLA−ML
Deaths (Thousands)
Week of
Apr May Jun Jul Aug Sep Oct Nov Dec Jan Feb Mar Apr May Jun
Cumulative Out−Of−Sample Error (Post Intercept Shift)
Sep, 2020
Oct, 2020
Nov, 2020
Dec, 2020
Jan, 2021
Med Abs Err
Med Err
Weeks Forecasting
All Model Versions
Delphi
Los Alamos Nat Lab
Youyang Gu
SIKJalpha
IHME
UCLA−ML
All Cumulative Errors
Month

Washington

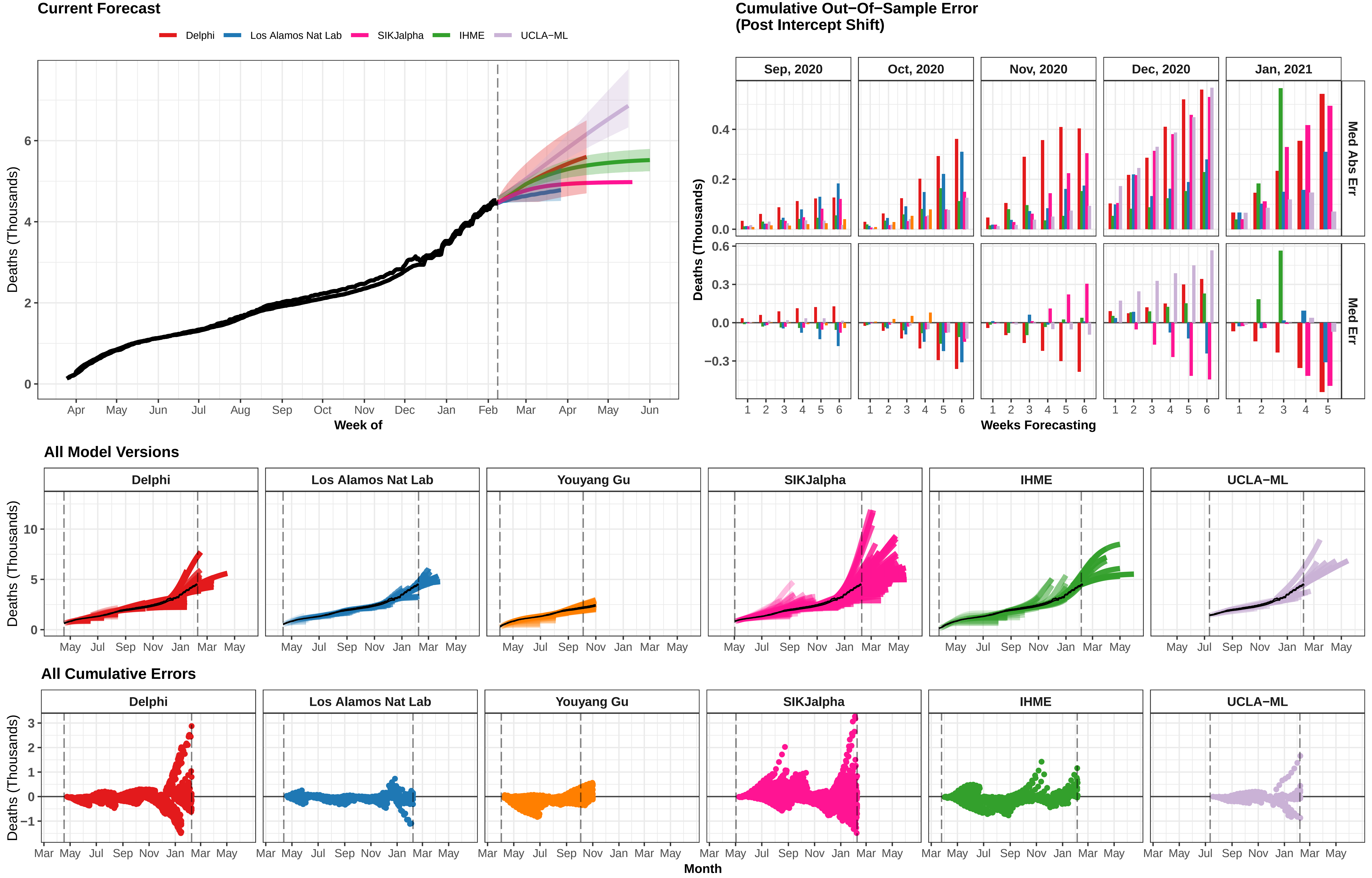
Current Forecast
Delphi
Los Alamos Nat Lab
SIKJalpha
IHME
UCLA−ML
Deaths (Thousands)
Week of
Apr May Jun Jul Aug Sep Oct Nov Dec Jan Feb Mar Apr May Jun
Cumulative Out−Of−Sample Error
(Post Intercept Shift)
Sep, 2020
Oct, 2020
Nov, 2020
Dec, 2020
Jan, 2021
Med Abs Err
Med Err
Weeks Forecasting
All Model Versions
Delphi
Los Alamos Nat Lab
Youyang Gu
SIKJalpha
IHME
UCLA−ML
All Cumulative Errors
Month

Jordan

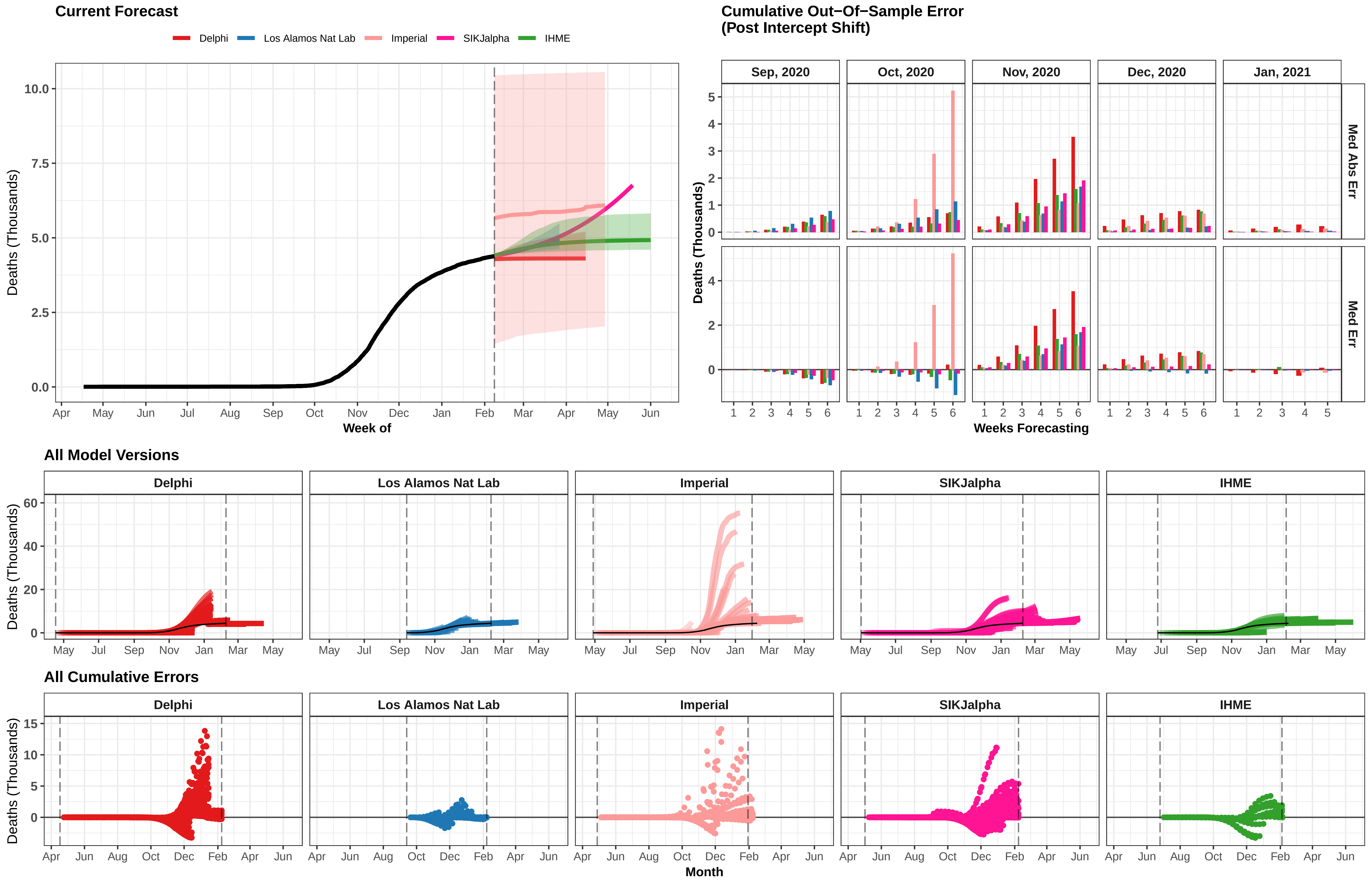

Current Forecast
Delphi
Los Alamos Nat Lab
Imperial
SIKJalpha
IHME
Deaths (Thousands)
Week of
Cumulative Out−Of−Sample Error
(Post Intercept Shift)
Sep, 2020
Oct, 2020
Nov, 2020
Dec, 2020
Jan, 2021
Med Abs Err
Med Err
Weeks Forecasting
All Model Versions
All Cumulative Errors
Month

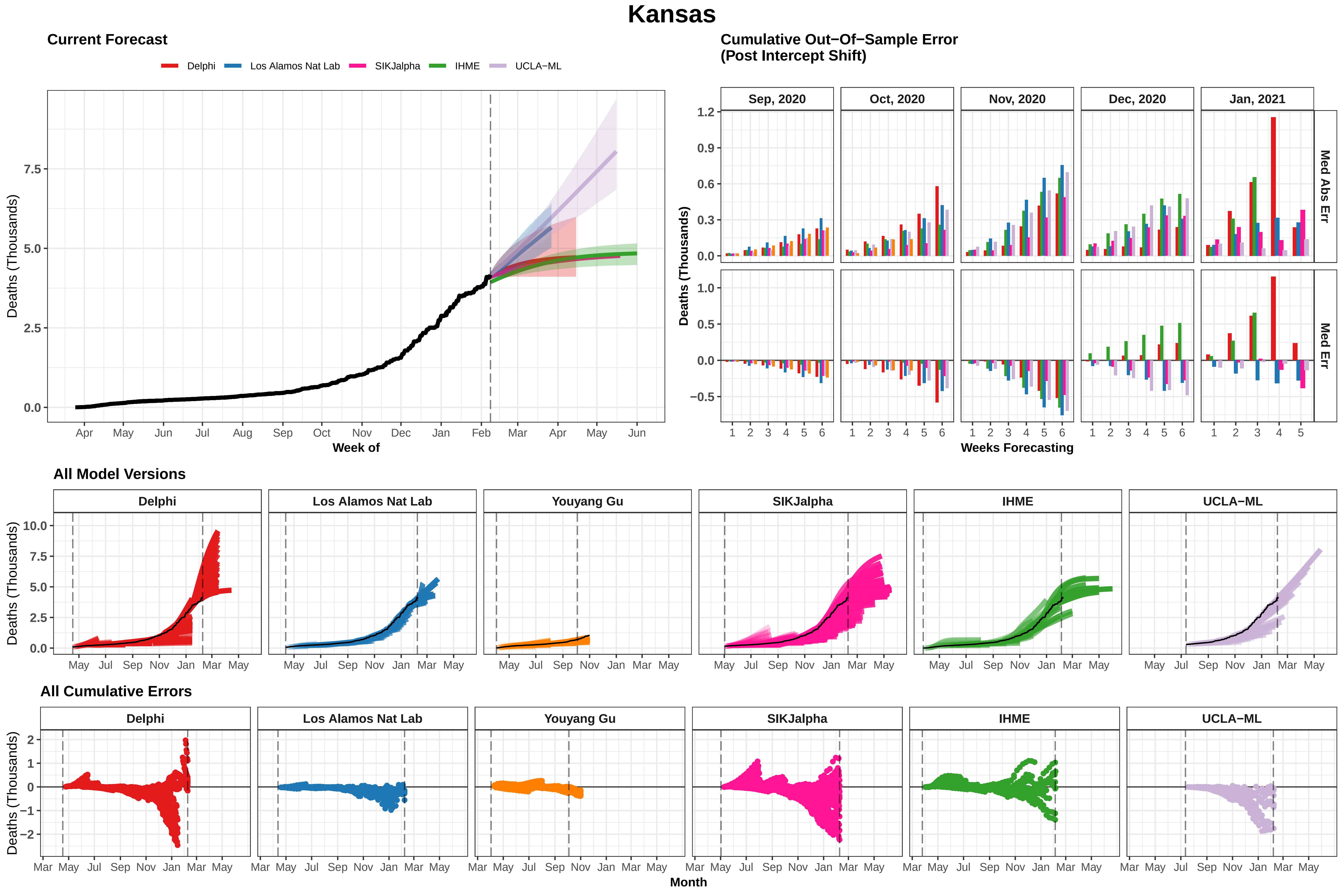

Kansas
Current Forecast
Delphi
Los Alamos Nat Lab
SIKJalpha
IHME
UCLA−ML
Deaths (Thousands)
Week of
Cumulative Out−Of−Sample Error
(Post Intercept Shift)
Sep, 2020
Oct, 2020
Nov, 2020
Dec, 2020
Jan, 2021
Med Abs Err
Med Err
Weeks Forecasting
All Model Versions
Delphi
Los Alamos Nat Lab
Youyang Gu
SIKJalpha
IHME
UCLA−ML
All Cumulative Errors
Month

Serbia

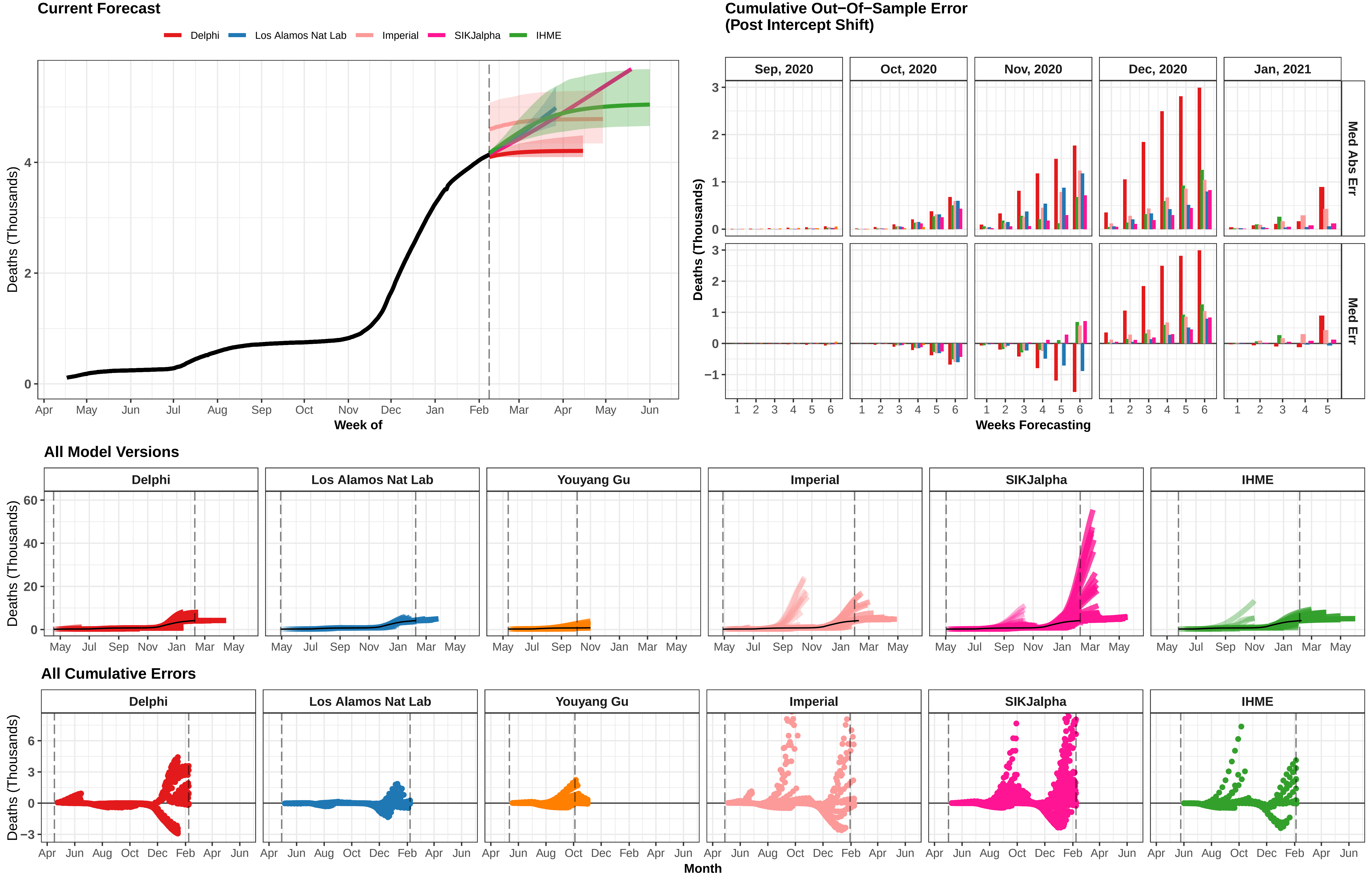

Current Forecast
Delphi
Los Alamos Nat Lab
Imperial
SIKJalpha
IHME
Deaths (Thousands)
Week of
Cumulative Out−Of−Sample Error
(Post Intercept Shift)
Sep, 2020
Oct, 2020
Nov, 2020
Dec, 2020
Jan, 2021
Med Abs Err
Med Err
Weeks Forecasting
All Model Versions
Delphi
Los Alamos Nat Lab
Youyang Gu
Imperial
SIKJalpha
IHME
All Cumulative Errors
Month

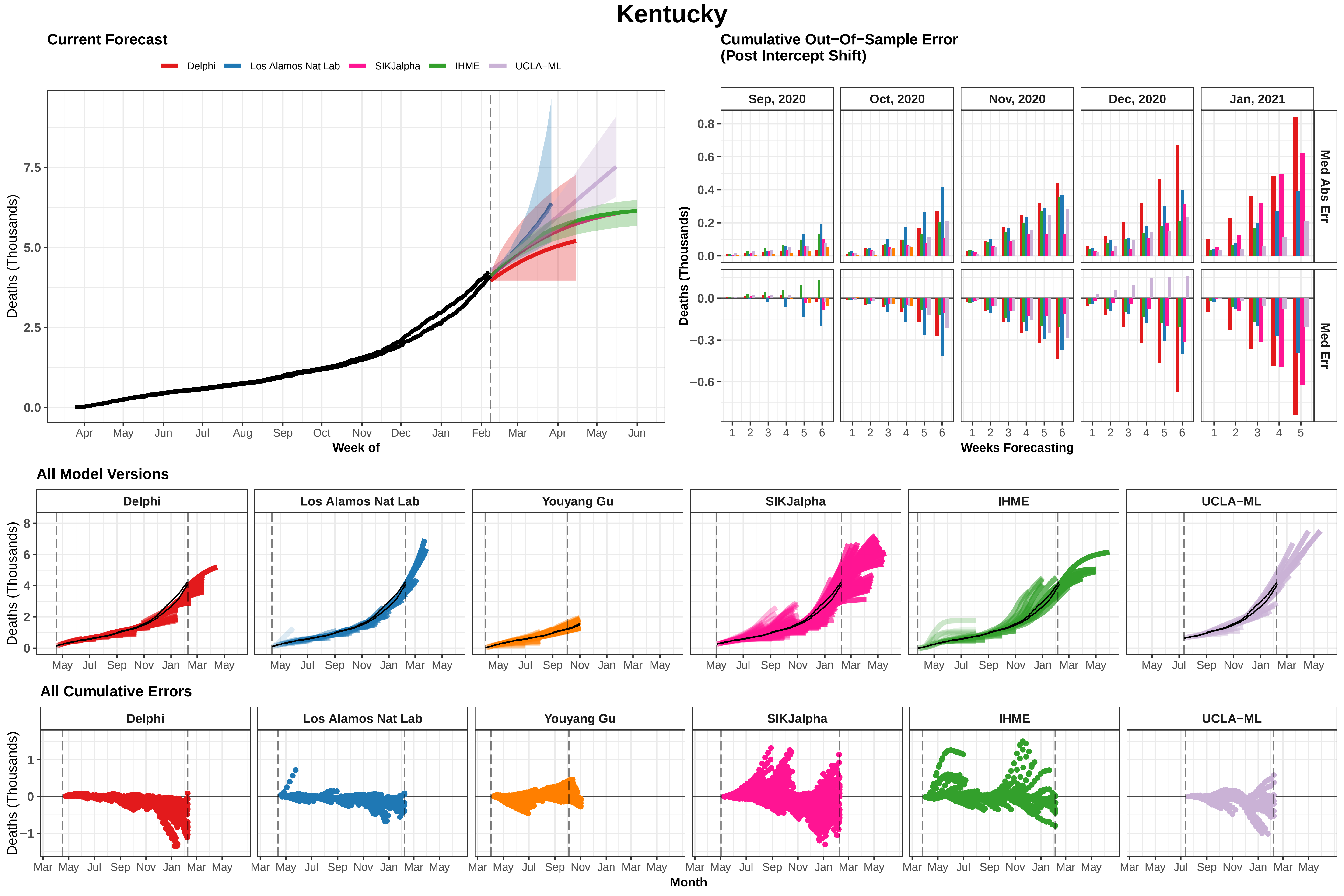

Kentucky
Current Forecast
Delphi
Los Alamos Nat Lab
SIKJalpha
IHME
UCLA−ML
Deaths (Thousands)
Week of
Apr May Jun Jul Aug Sep Oct Nov Dec Jan Feb Mar Apr May Jun
Cumulative Out−Of−Sample Error (Post Intercept Shift)
Sep, 2020
Oct, 2020
Nov, 2020
Dec, 2020
Jan, 2021
Med Abs Err
Med Err
Weeks Forecasting
All Model Versions
Delphi
Los Alamos Nat Lab
Youyang Gu
SIKJalpha
IHME
UCLA−ML
All Cumulative Errors
Month

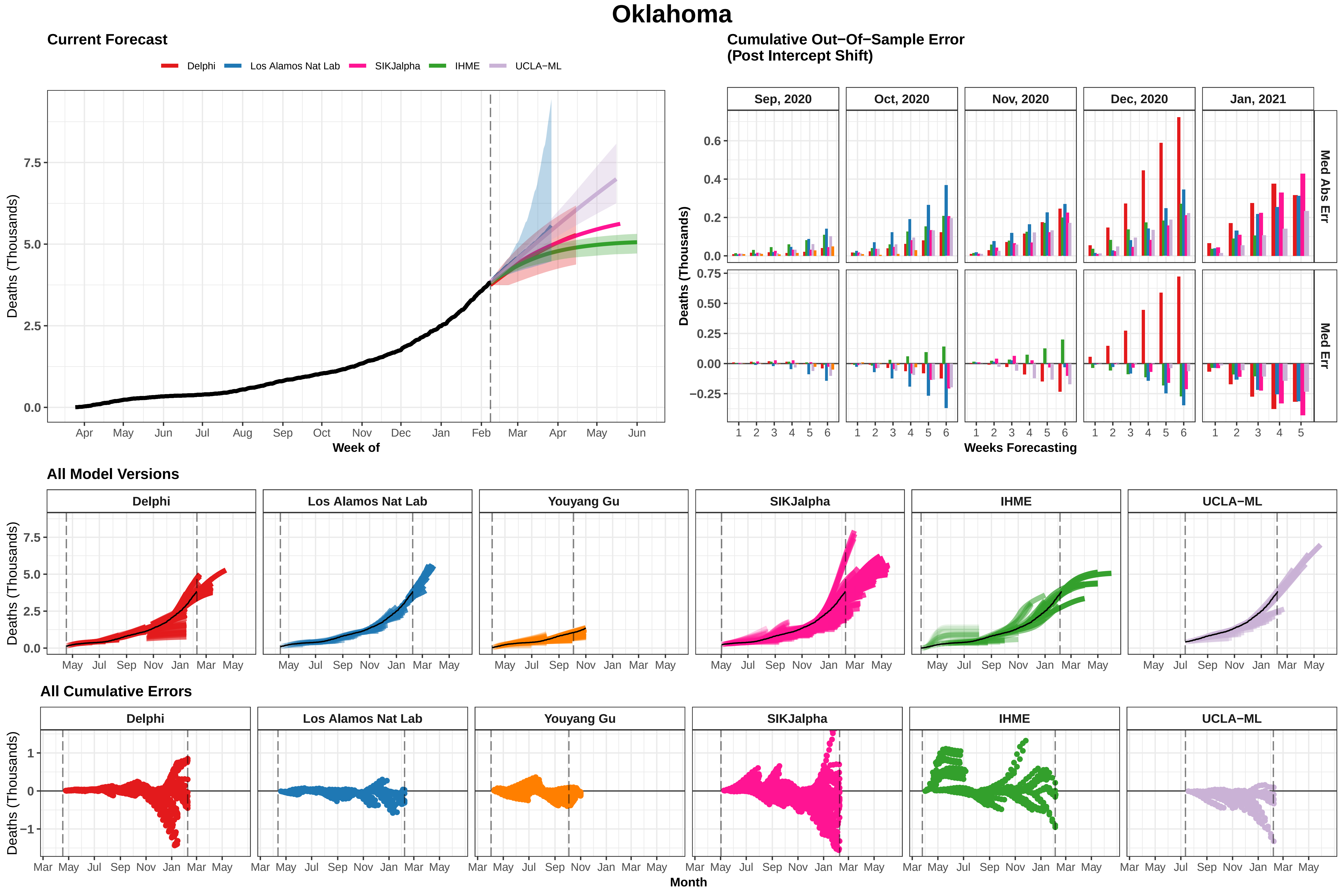

Oklahoma
Current Forecast
Delphi
Los Alamos Nat Lab
SIKJalpha
IHME
UCLA−ML
Deaths (Thousands)
Week of
Cumulative Out−Of−Sample Error
(Post Intercept Shift)
Sep, 2020
Oct, 2020
Nov, 2020
Dec, 2020
Jan, 2021
Med Abs Err
Med Err
Weeks Forecasting
All Model Versions
Delphi
Los Alamos Nat Lab
Youyang Gu
SIKJalpha
IHME
UCLA−ML
All Cumulative Errors
Month

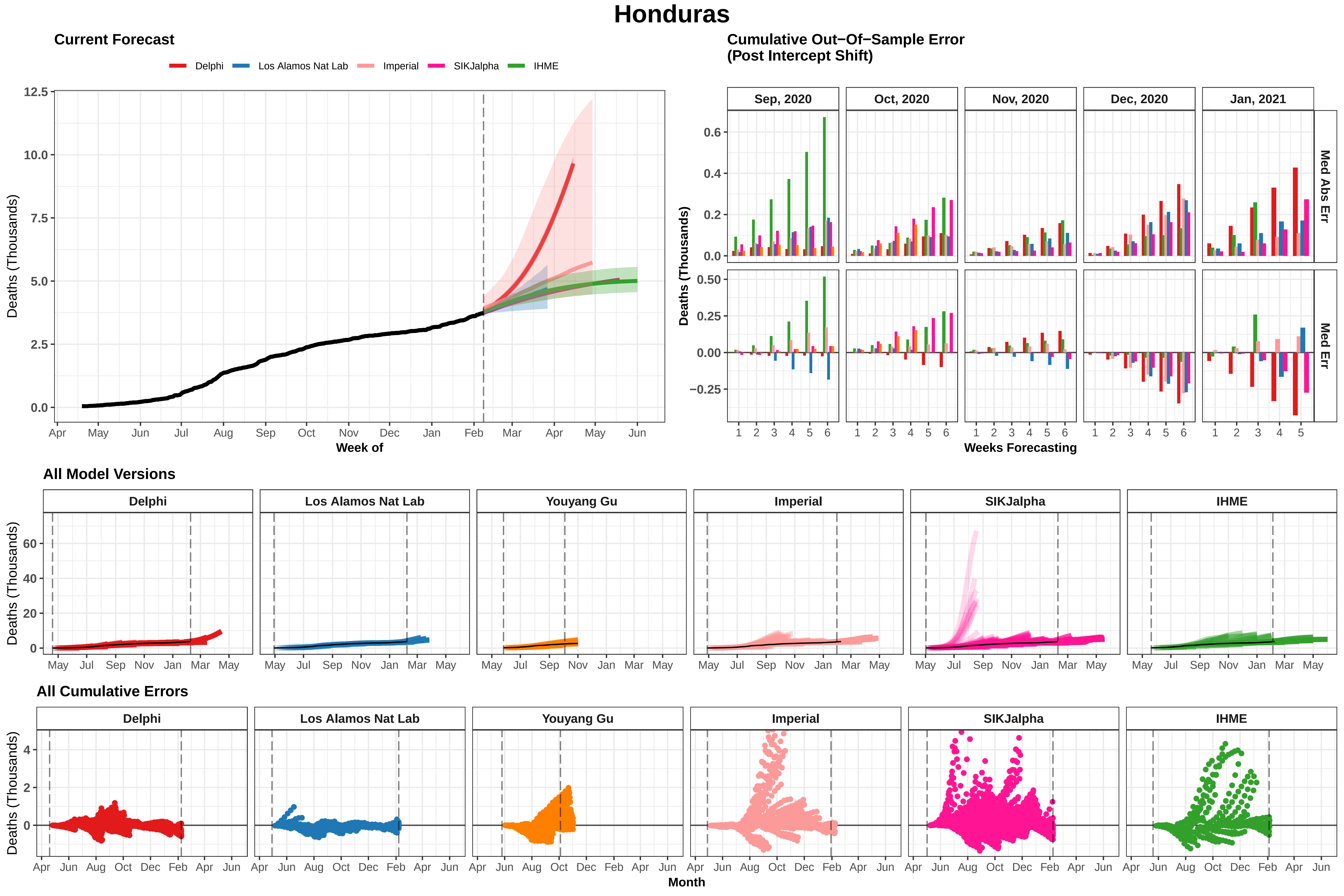

Honduras
Current Forecast
Delphi
Los Alamos Nat Lab
Imperial
SIKJalpha
IHME
Deaths (Thousands)
Week of
Cumulative Out−Of−Sample Error
(Post Intercept Shift)
Sep, 2020
Oct, 2020
Nov, 2020
Dec, 2020
Jan, 2021
Med Abs Err
Med Err
Weeks Forecasting
All Model Versions
Delphi
Los Alamos Nat Lab
Youyang Gu
Imperial
SIKJalpha
IHME
All Cumulative Errors
Month

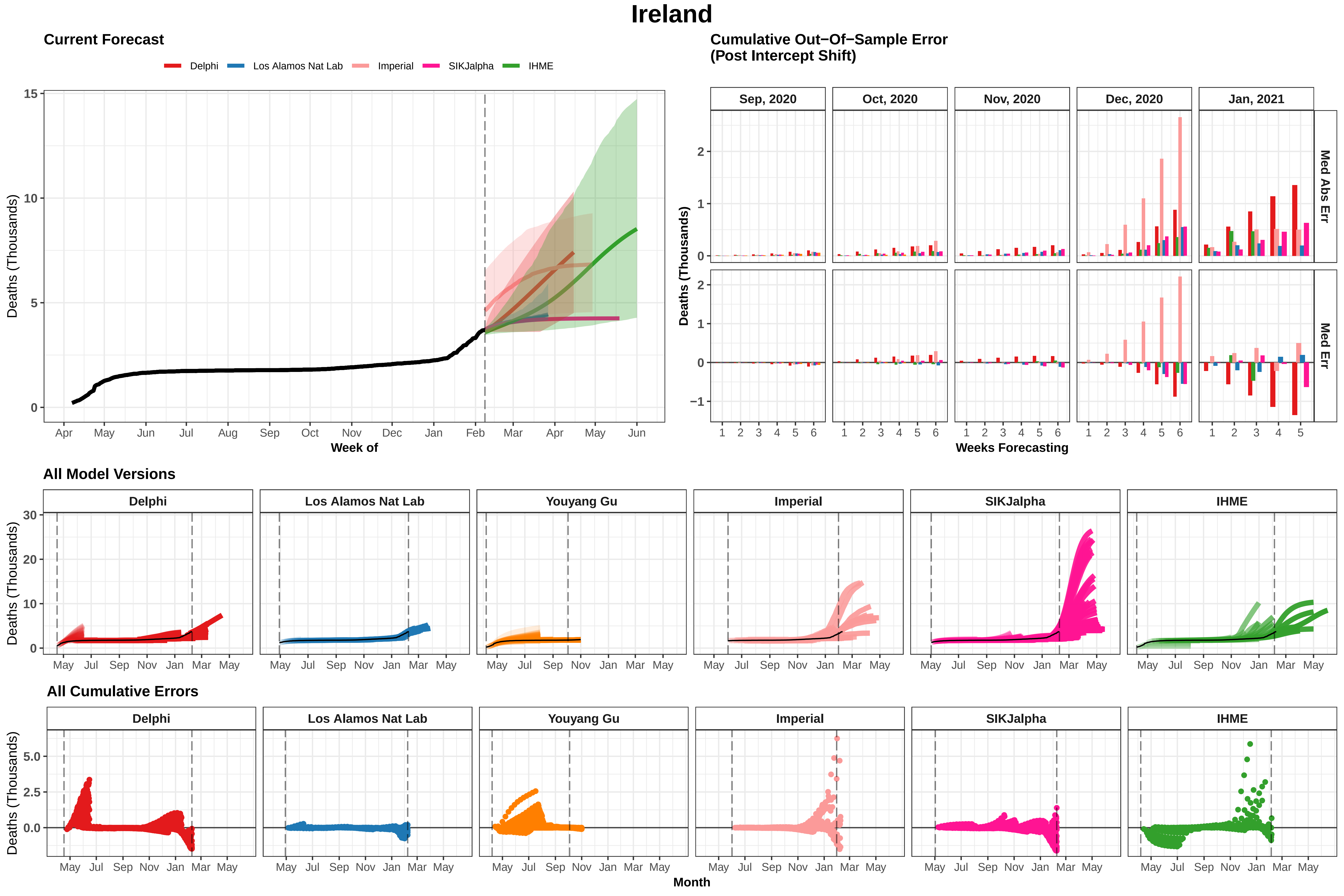

Ireland
Current Forecast
Delphi
Los Alamos Nat Lab
Imperial
SIKJalpha
IHME
Deaths (Thousands)
Week of
Cumulative Out−Of−Sample Error
(Post Intercept Shift)
Sep, 2020
Oct, 2020
Nov, 2020
Dec, 2020
Jan, 2021
Med Abs Err
Med Err
Weeks Forecasting
All Model Versions
Delphi
Los Alamos Nat Lab
Youyang Gu
Imperial
SIKJalpha
IHME
All Cumulative Errors
Month

Lebanon

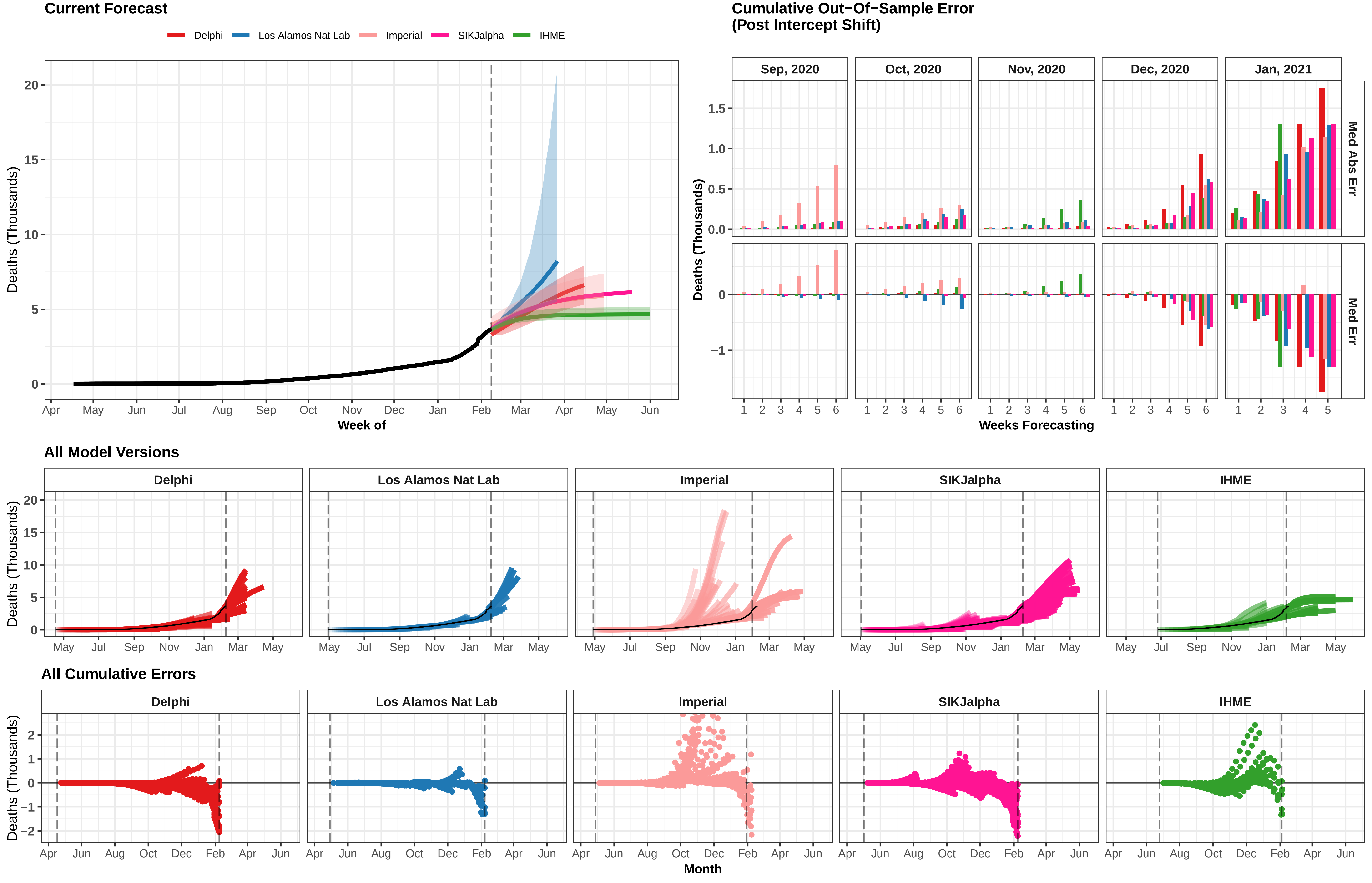

Current Forecast
Delphi
Los Alamos Nat Lab
Imperial
SIKJalpha
IHME
Deaths (Thousands)
Week of
Apr
May
Jun
Jul
Aug
Sep
Oct
Nov
Dec
Jan
Feb
Mar
Cumulative Out−Of−Sample Error
(Post Intercept Shift)
Sep, 2020
Oct, 2020
Nov, 2020
Dec, 2020
Jan, 2021
Med Abs Err
Med Err
Weeks Forecasting
All Model Versions
All Cumulative Errors
Month

Slovenia

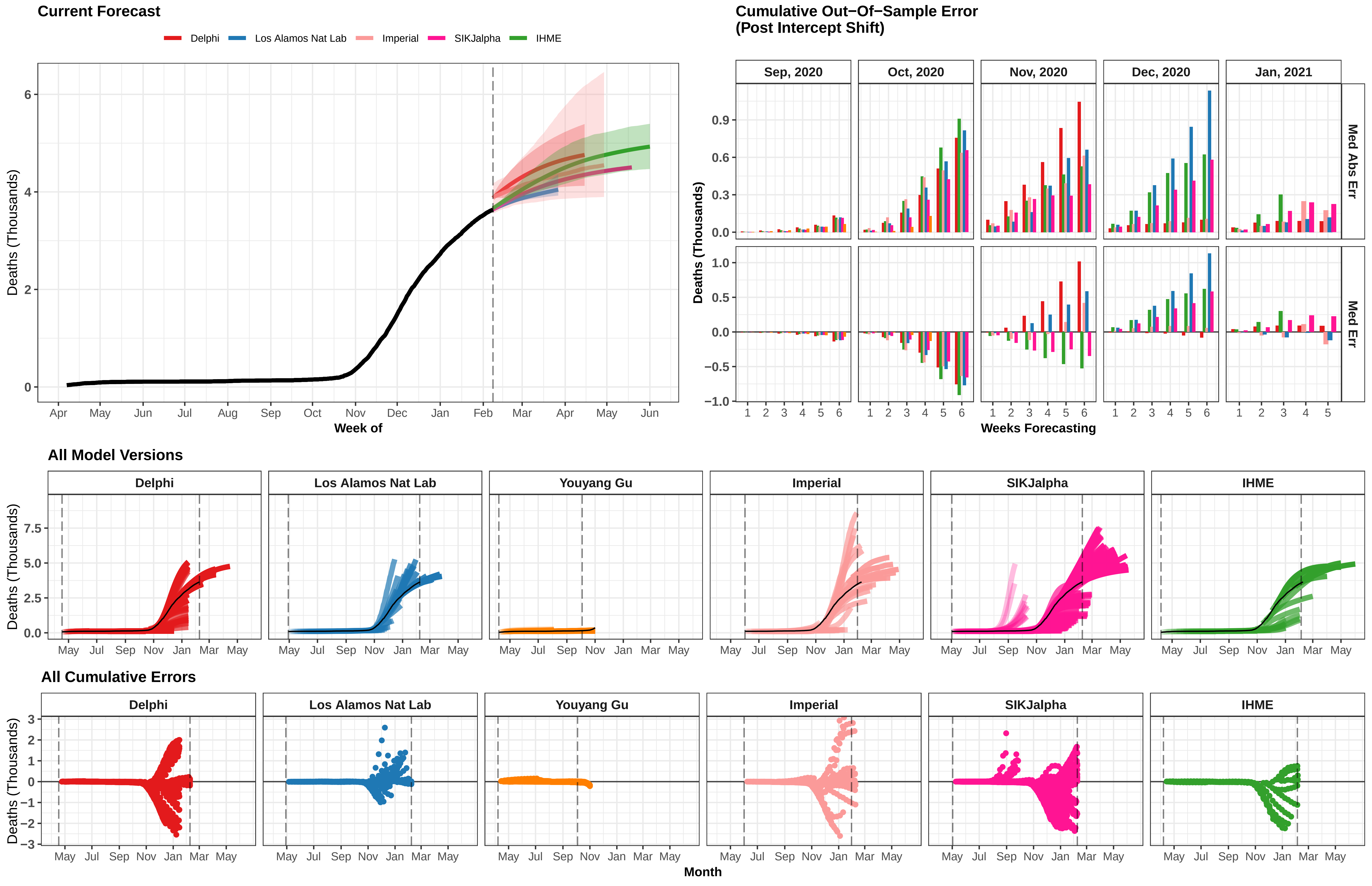

Current Forecast
Delphi
Los Alamos Nat Lab
Imperial
SIKJalpha
IHME
Deaths (Thousands)
Week of
Apr May Jun Jul Aug Sep Oct Nov Dec Jan Feb Mar Apr May Jun
Cumulative Out−Of−Sample Error
(Post Intercept Shift)
Sep, 2020
Oct, 2020
Nov, 2020
Dec, 2020
Jan, 2021
Med Abs Err
Med Err
Weeks Forecasting
All Model Versions
Delphi
Los Alamos Nat Lab
Youyang Gu
Imperial
SIKJalpha
IHME
All Cumulative Errors
Month
May Jul Sep Nov Jan Mar May

Moldova

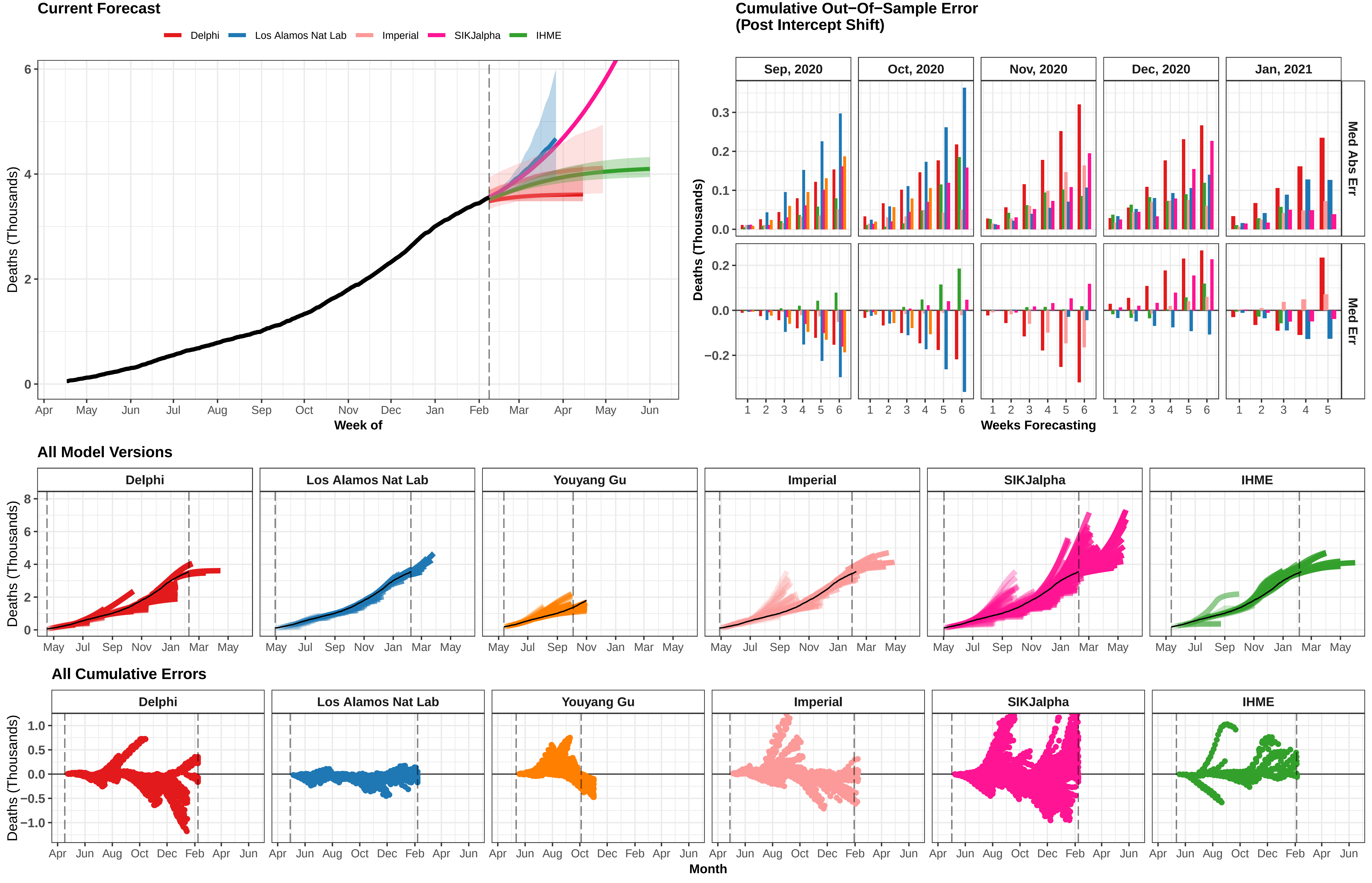

Current Forecast
Delphi
Los Alamos Nat Lab
Imperial
SIKJalpha
IHME
Deaths (Thousands)
Week of
Apr
May
Jun
Jul
Aug
Sep
Oct
Nov
Dec
Jan
Feb
Mar
Cumulative Out−Of−Sample Error
(Post Intercept Shift)
Sep, 2020
Oct, 2020
Nov, 2020
Dec, 2020
Jan, 2021
Med Abs Err
Med Err
Weeks Forecasting
All Model Versions
Youyang Gu
All Cumulative Errors
Month

New Mexico

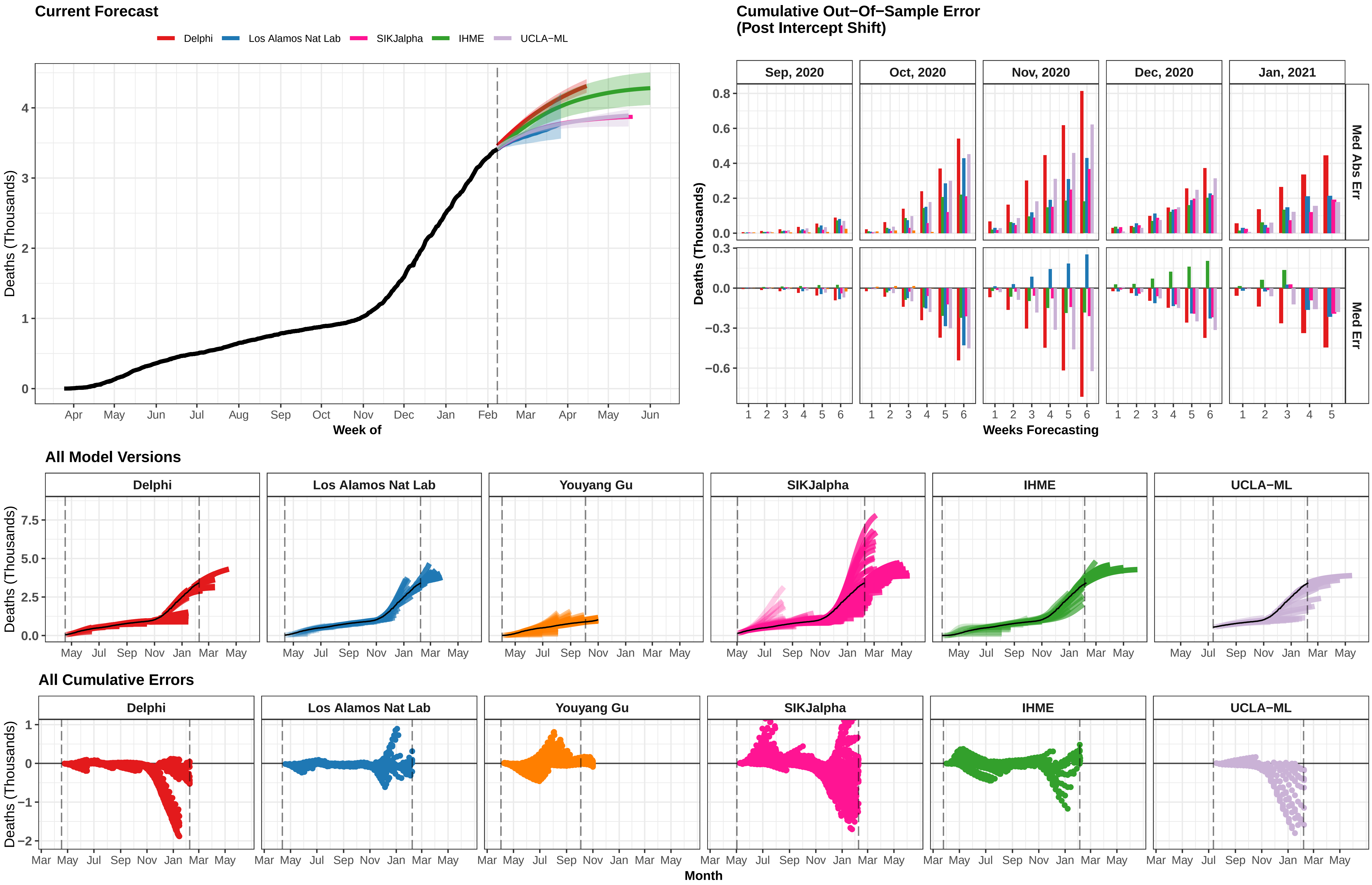

Current Forecast
Delphi
Los Alamos Nat Lab
SIKJalpha
IHME
UCLA−ML
Deaths (Thousands)
Week of
Apr May Jun Jul Aug Sep Oct Nov Dec Jan Feb Mar Apr May Jun
Cumulative Out−Of−Sample Error
(Post Intercept Shift)
Sep, 2020
Oct, 2020
Nov, 2020
Dec, 2020
Jan, 2021
Med Abs Err
Med Err
Weeks Forecasting
All Model Versions
Delphi
Los Alamos Nat Lab
Youyang Gu
SIKJalpha
IHME
UCLA−ML
All Cumulative Errors
Month

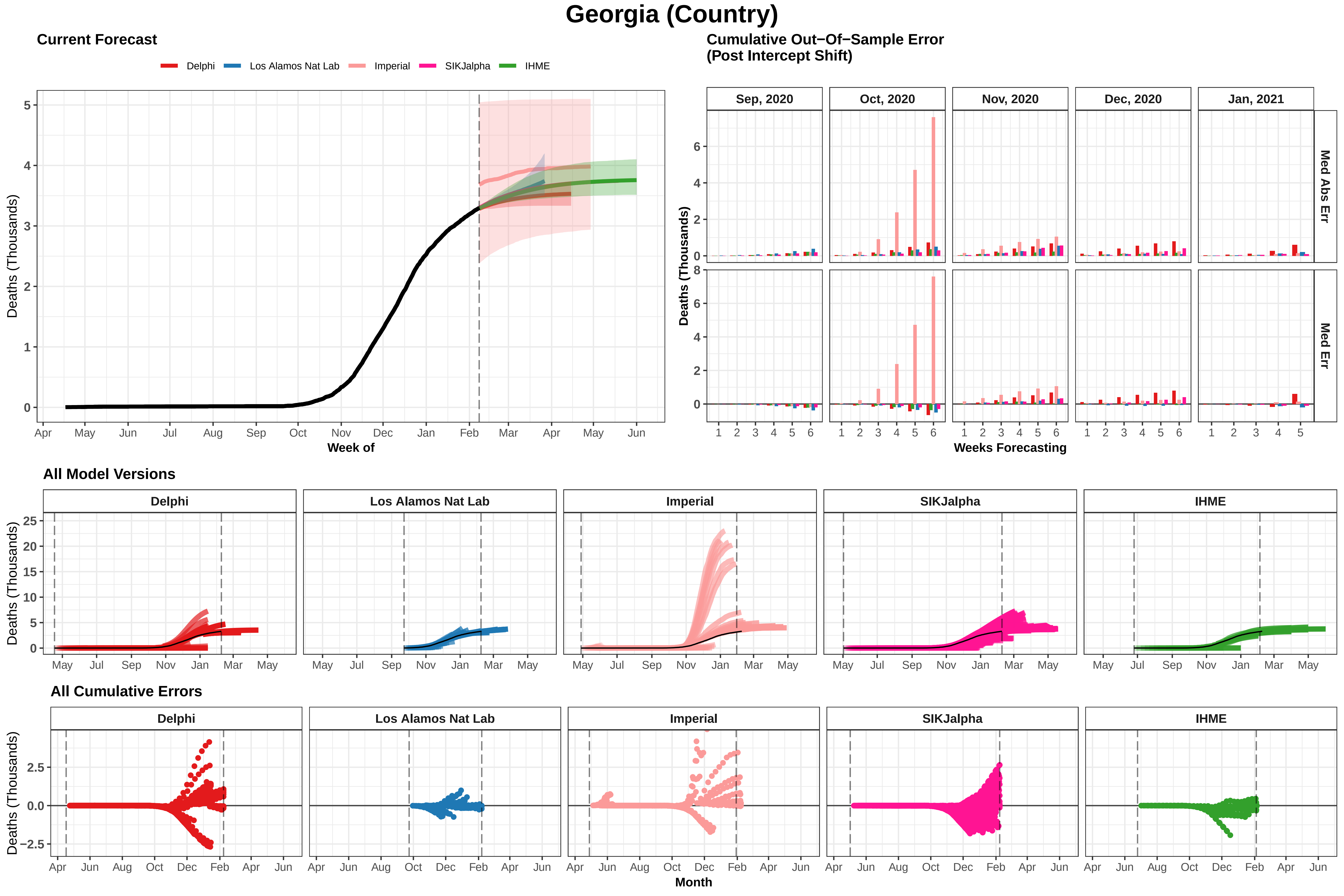
Georgia (Country)
Current Forecast
Delphi
Los Alamos Nat Lab
Imperial
SIKJalpha
IHME
Deaths (Thousands)
Week of
Cumulative Out−Of−Sample Error
(Post Intercept Shift)
Sep, 2020
Oct, 2020
Nov, 2020
Dec, 2020
Jan, 2021
Med Abs Err
Med Err
Weeks Forecasting
All Model Versions
All Cumulative Errors
Month

Myanmar

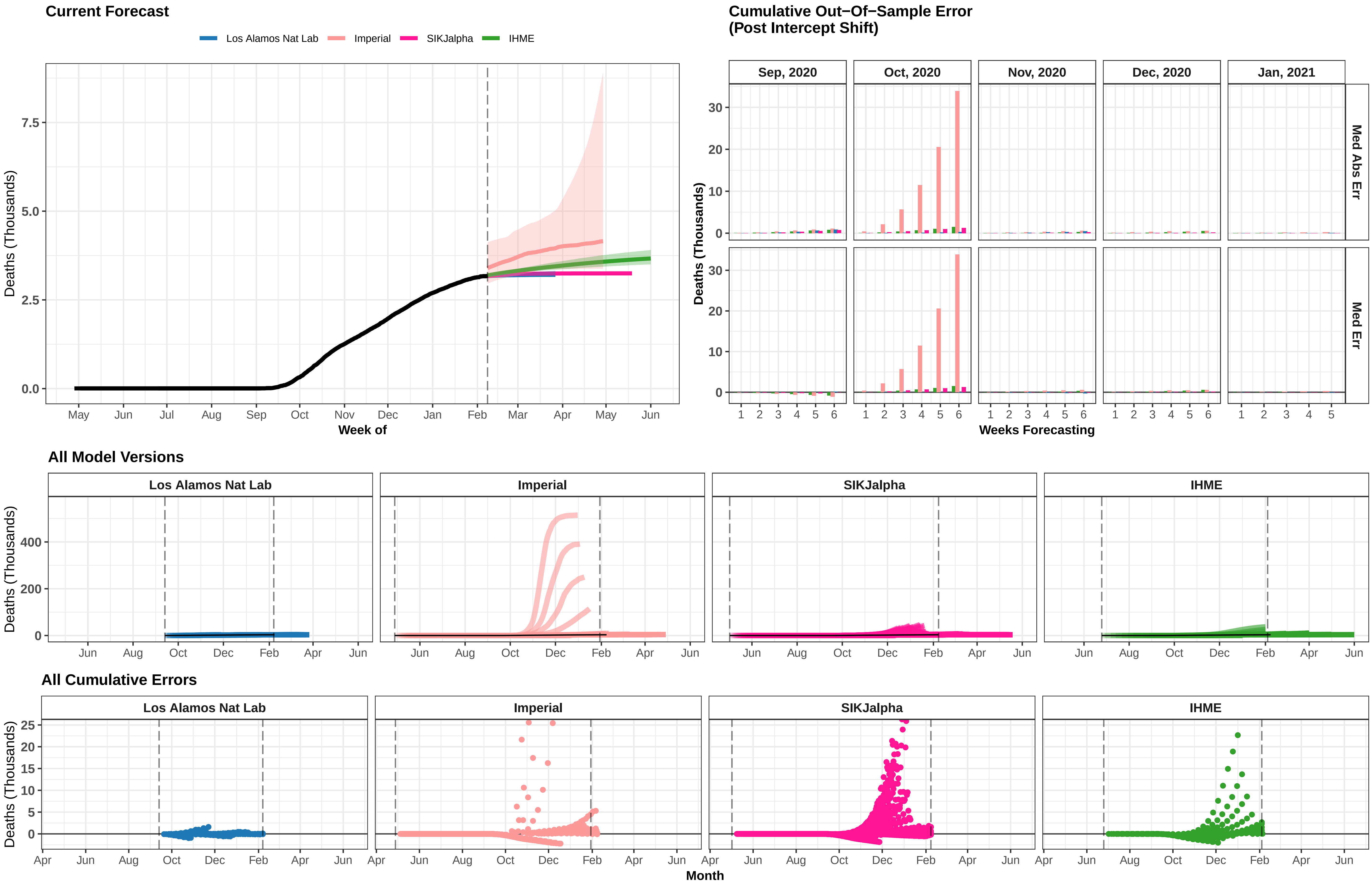

Current Forecast
Los Alamos Nat Lab
Imperial
SIKJalpha
IHME
Deaths (Thousands)
Week of
Cumulative Out−Of−Sample Error
(Post Intercept Shift)
Sep, 2020
Oct, 2020
Nov, 2020
Dec, 2020
Jan, 2021
Med Abs Err
Med Err
Weeks Forecasting
All Model Versions
All Cumulative Errors
Month

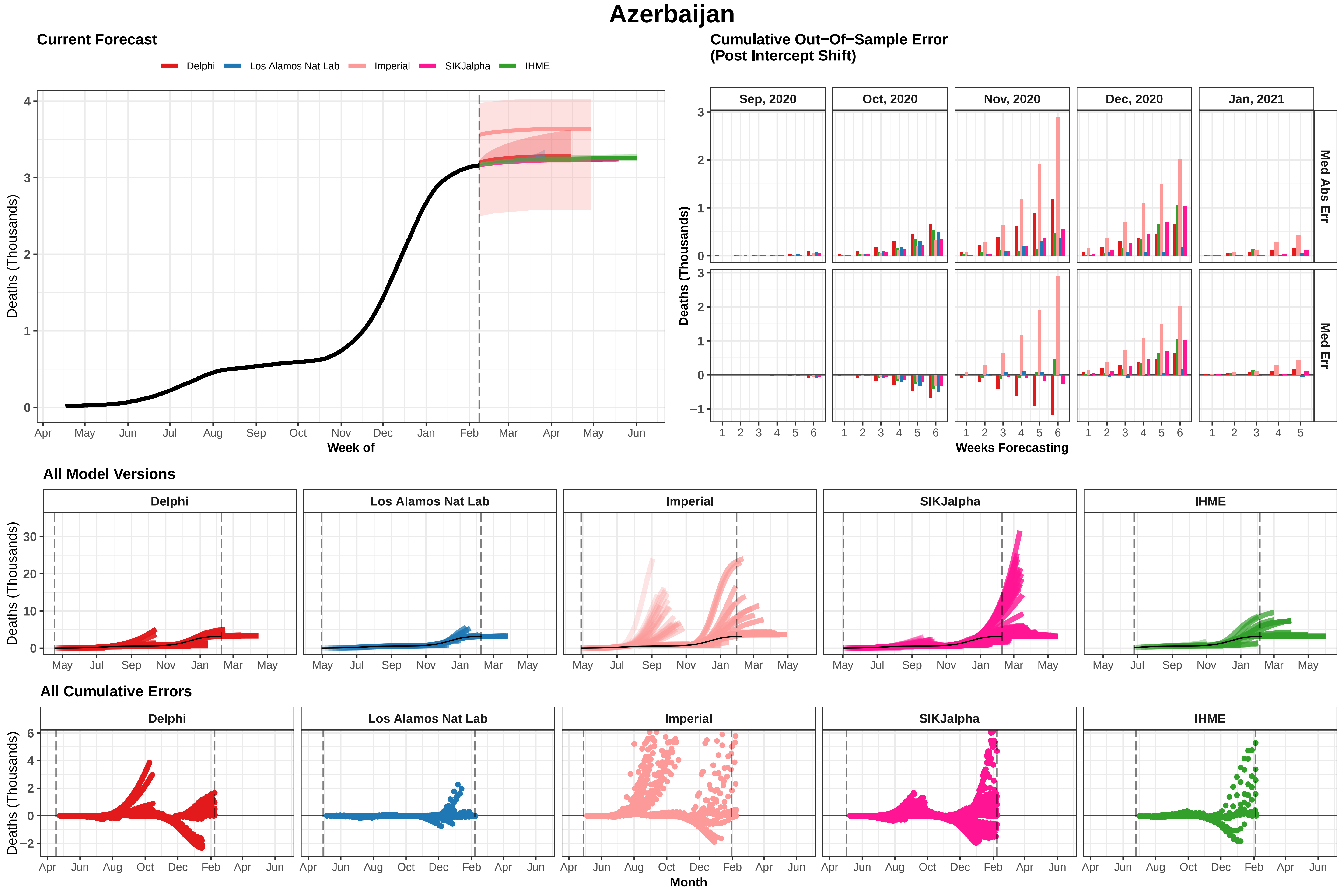
Azerbaijan
Current Forecast
Delphi
Los Alamos Nat Lab
Imperial
SIKJalpha
IHME
Deaths (Thousands)
Week of
Apr May Jun Jul Aug Sep Oct Nov Dec Jan Feb Mar Apr May Jun
Cumulative Out−Of−Sample Error
(Post Intercept Shift)
Sep, 2020
Oct, 2020
Nov, 2020
Dec, 2020
Jan, 2021
Med Abs Err
Med Err
Weeks Forecasting
All Model Versions
Delphi
Los Alamos Nat Lab
Imperial
SIKJalpha
IHME
May Jul Sep Nov Jan Mar May
All Cumulative Errors
Delphi
Los Alamos Nat Lab
Imperial
SIKJalpha
IHME
Apr Jun Aug Oct Dec Feb Apr Jun
Month

Kazakhstan

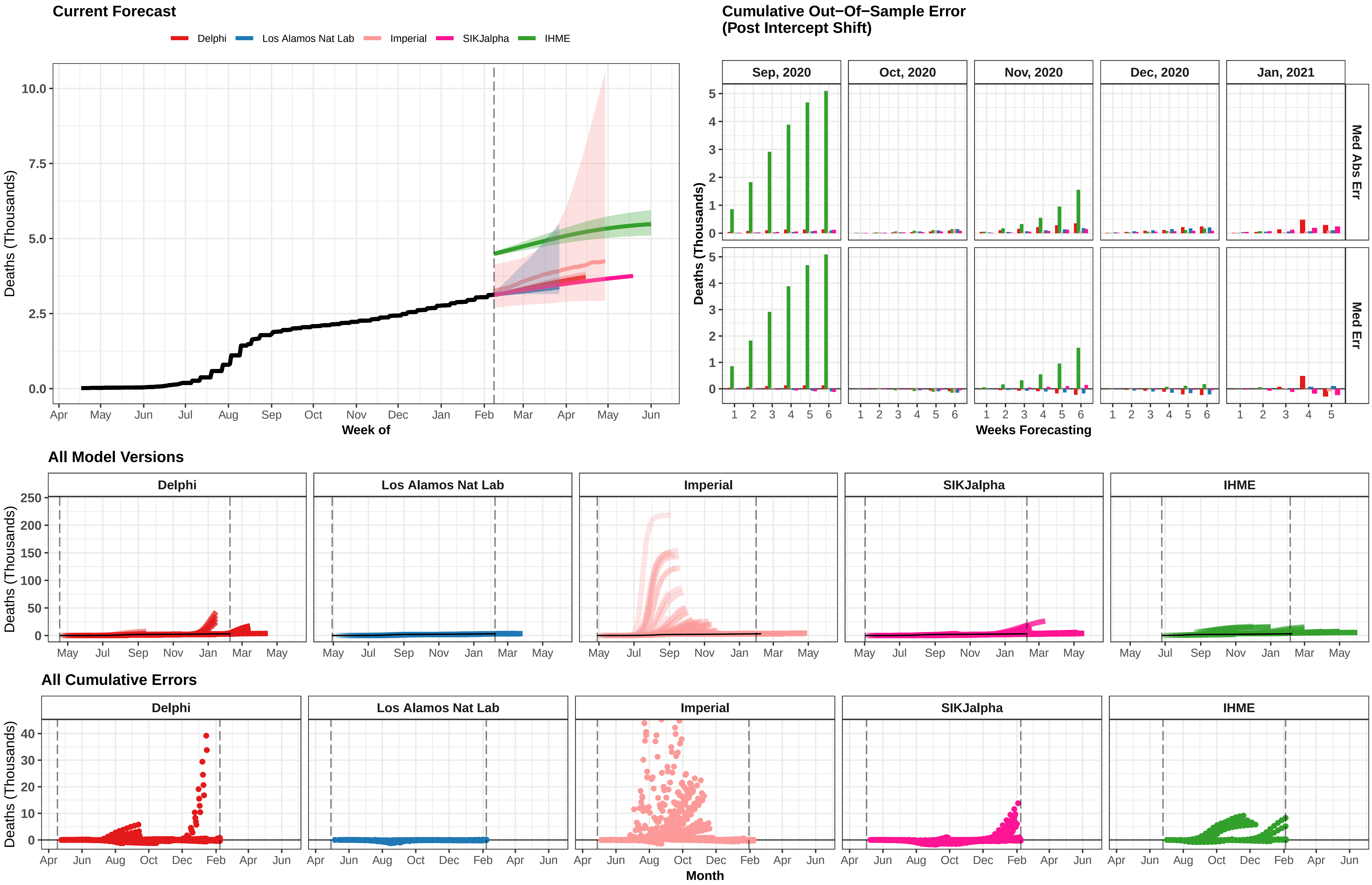

Current Forecast
Delphi
Los Alamos Nat Lab
Imperial
SIKJalpha
IHME
Deaths (Thousands)
Week of
Cumulative Out−Of−Sample Error
(Post Intercept Shift)
Sep, 2020
Oct, 2020
Nov, 2020
Dec, 2020
Jan, 2021
Med Abs Err
Med Err
Weeks Forecasting
All Model Versions
All Cumulative Errors
Month

Armenia

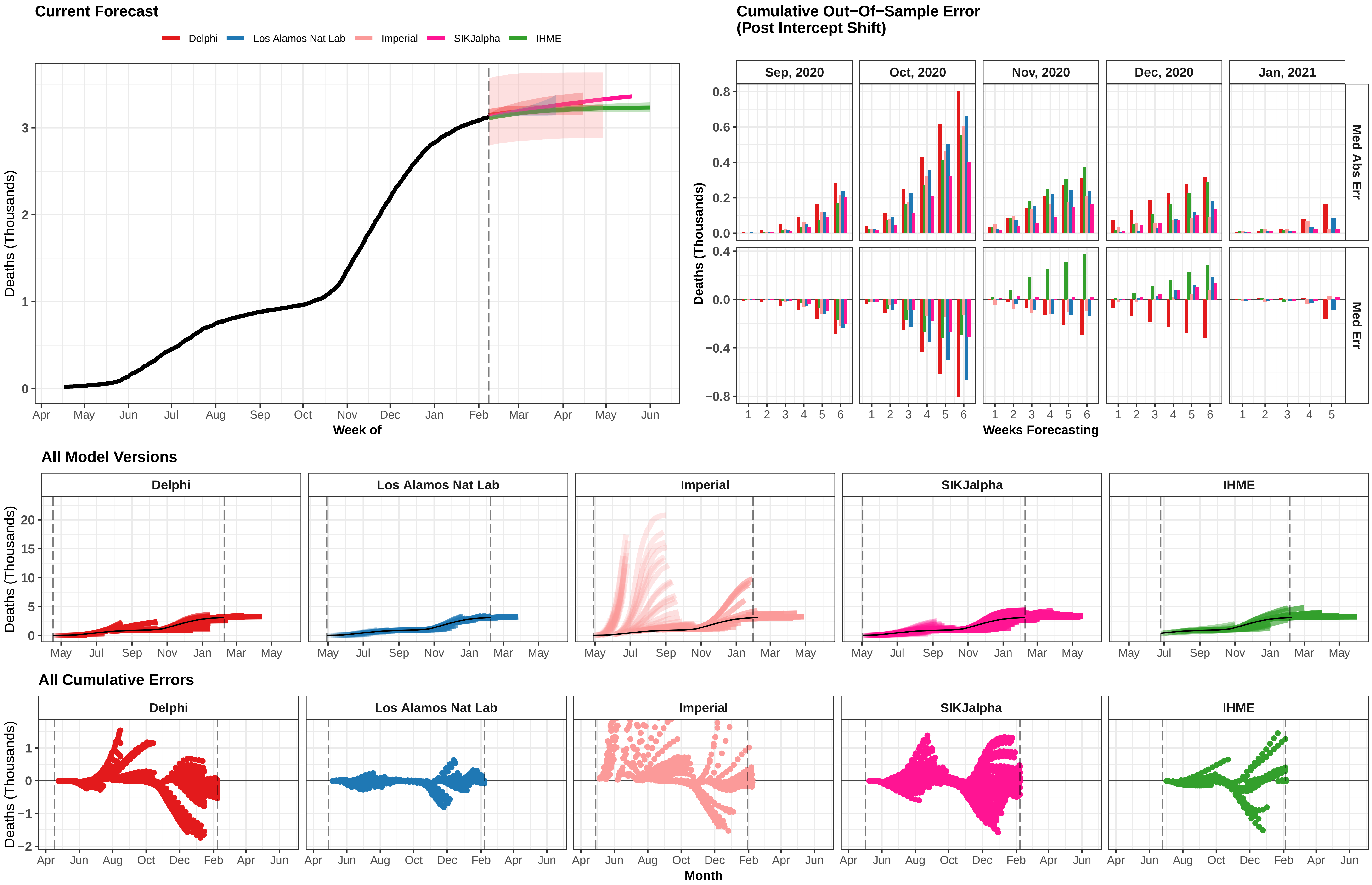

Current Forecast
Delphi
Los Alamos Nat Lab
Imperial
SIKJalpha
IHME
Deaths (Thousands)
Week of
Apr
May
Jun
Jul
Aug
Sep
Oct
Nov
Dec
Jan
Feb
Mar
Cumulative Out−Of−Sample Error
(Post Intercept Shift)
Sep, 2020
Oct, 2020
Nov, 2020
Dec, 2020
Jan, 2021
Med Abs Err
Med Err
Weeks Forecasting
All Model Versions
All Cumulative Errors
Month

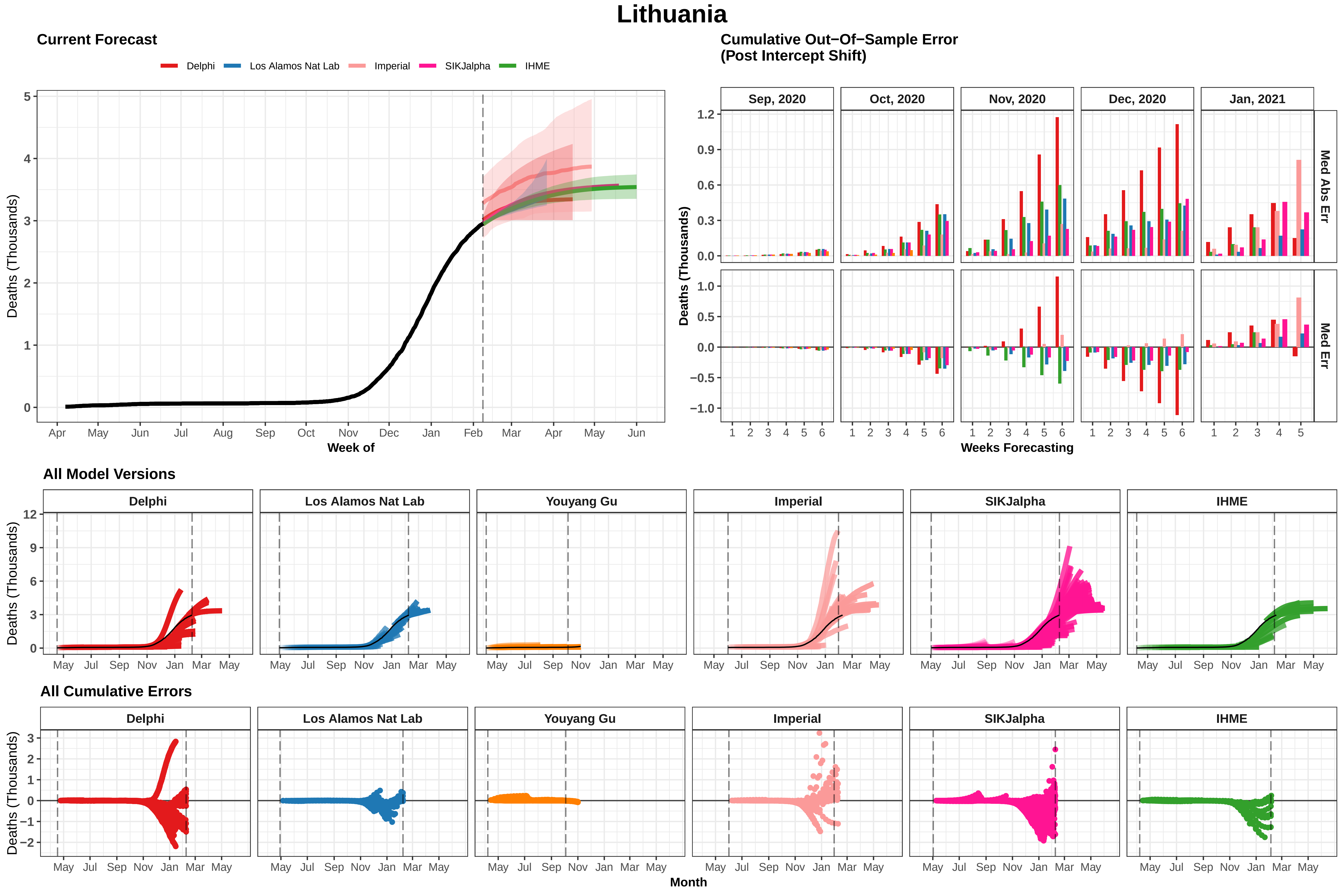
Lithuania
Current Forecast
Delphi
Los Alamos Nat Lab
Imperial
SIKJalpha
IHME
Deaths (Thousands)
Week of
Apr May Jun Jul Aug Sep Oct Nov Dec Jan Feb Mar Apr May Jun
Cumulative Out−Of−Sample Error
(Post Intercept Shift)
Sep, 2020
Oct, 2020
Nov, 2020
Dec, 2020
Jan, 2021
Med Abs Err
Med Err
Weeks Forecasting
All Model Versions
Delphi
Los Alamos Nat Lab
Youyang Gu
Imperial
SIKJalpha
IHME
All Cumulative Errors
Month

Macedonia

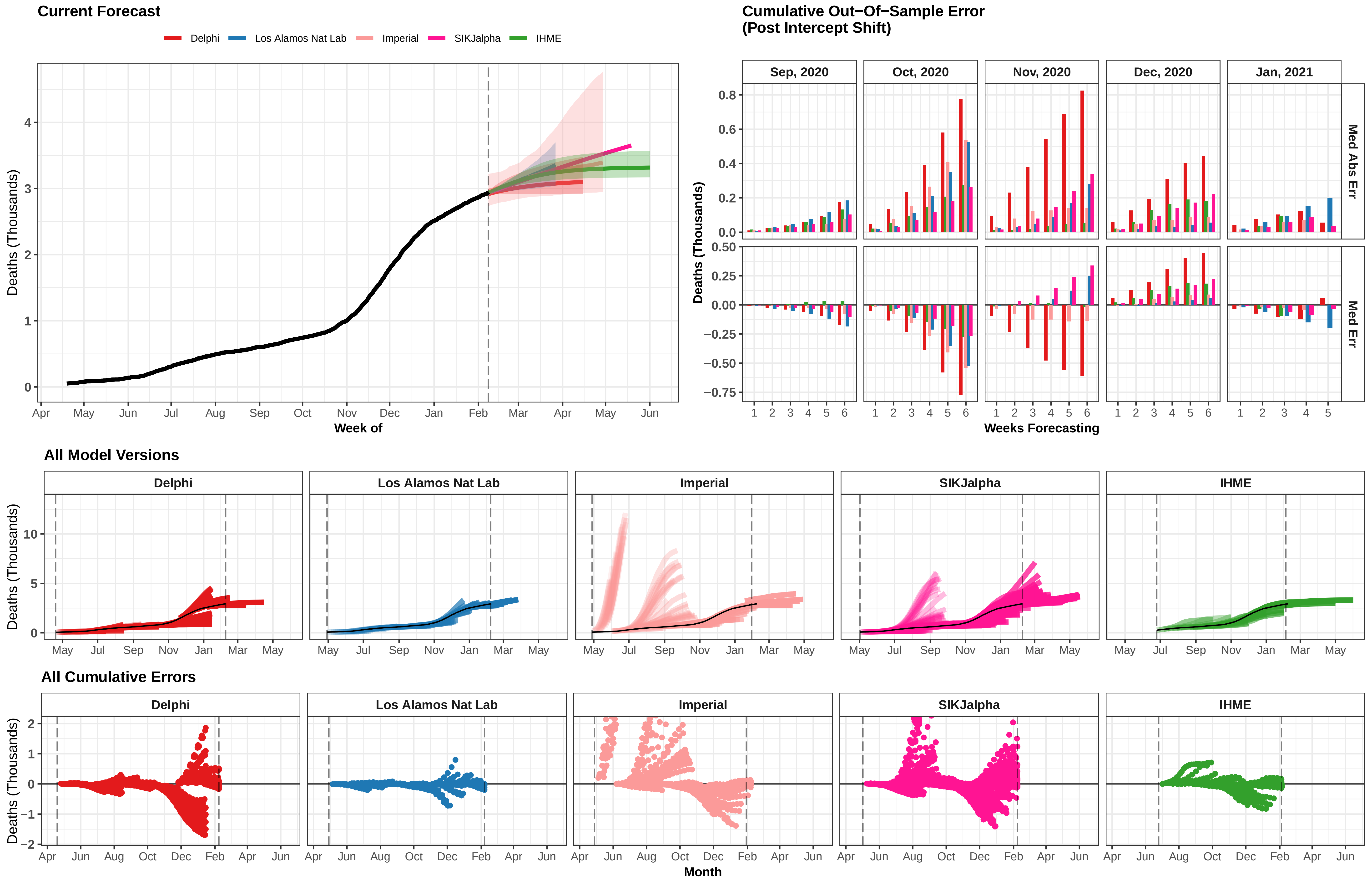

Current Forecast
Delphi
Los Alamos Nat Lab
Imperial
SIKJalpha
IHME
Deaths (Thousands)
Week of
Apr May Jun Jul Aug Sep Oct Nov Dec Jan Feb Mar Apr May Jun
Cumulative Out−Of−Sample Error
(Post Intercept Shift)
Sep, 2020
Oct, 2020
Nov, 2020
Dec, 2020
Jan, 2021
Med Abs Err
Med Err
Weeks Forecasting
All Model Versions
All Cumulative Errors
Month

Algeria

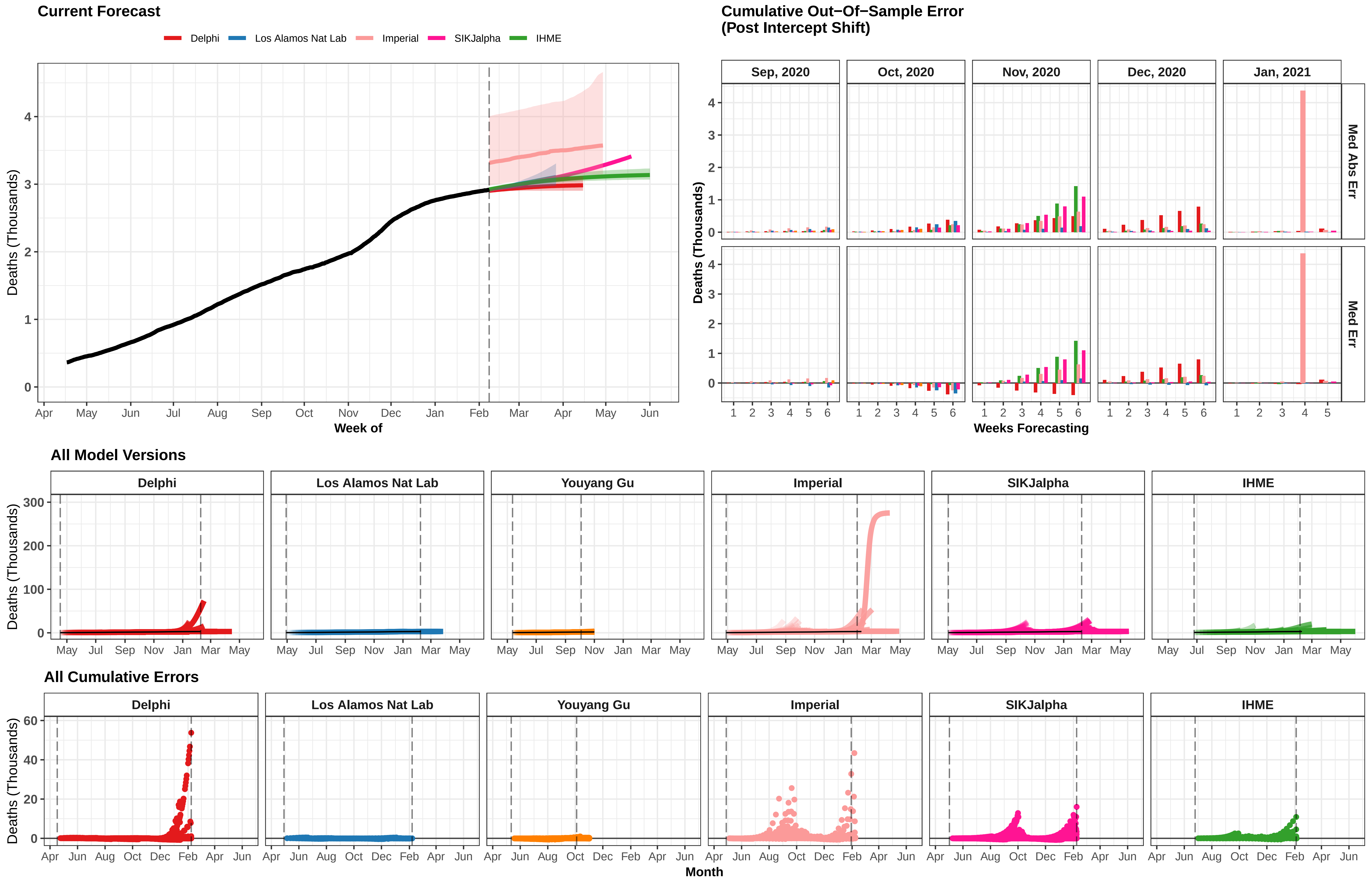

Current Forecast
Delphi
Los Alamos Nat Lab
Imperial
SIKJalpha
IHME
Deaths (Thousands)
Week of
Apr May Jun Jul Aug Sep Oct Nov Dec Jan Feb Mar Apr May Jun
Cumulative Out−Of−Sample Error
(Post Intercept Shift)
Sep, 2020
Oct, 2020
Nov, 2020
Dec, 2020
Jan, 2021
Med Abs Err
Med Err
Weeks Forecasting
All Model Versions
Delphi
Los Alamos Nat Lab
Youyang Gu
Imperial
SIKJalpha
IHME
All Cumulative Errors
Month

Paraguay

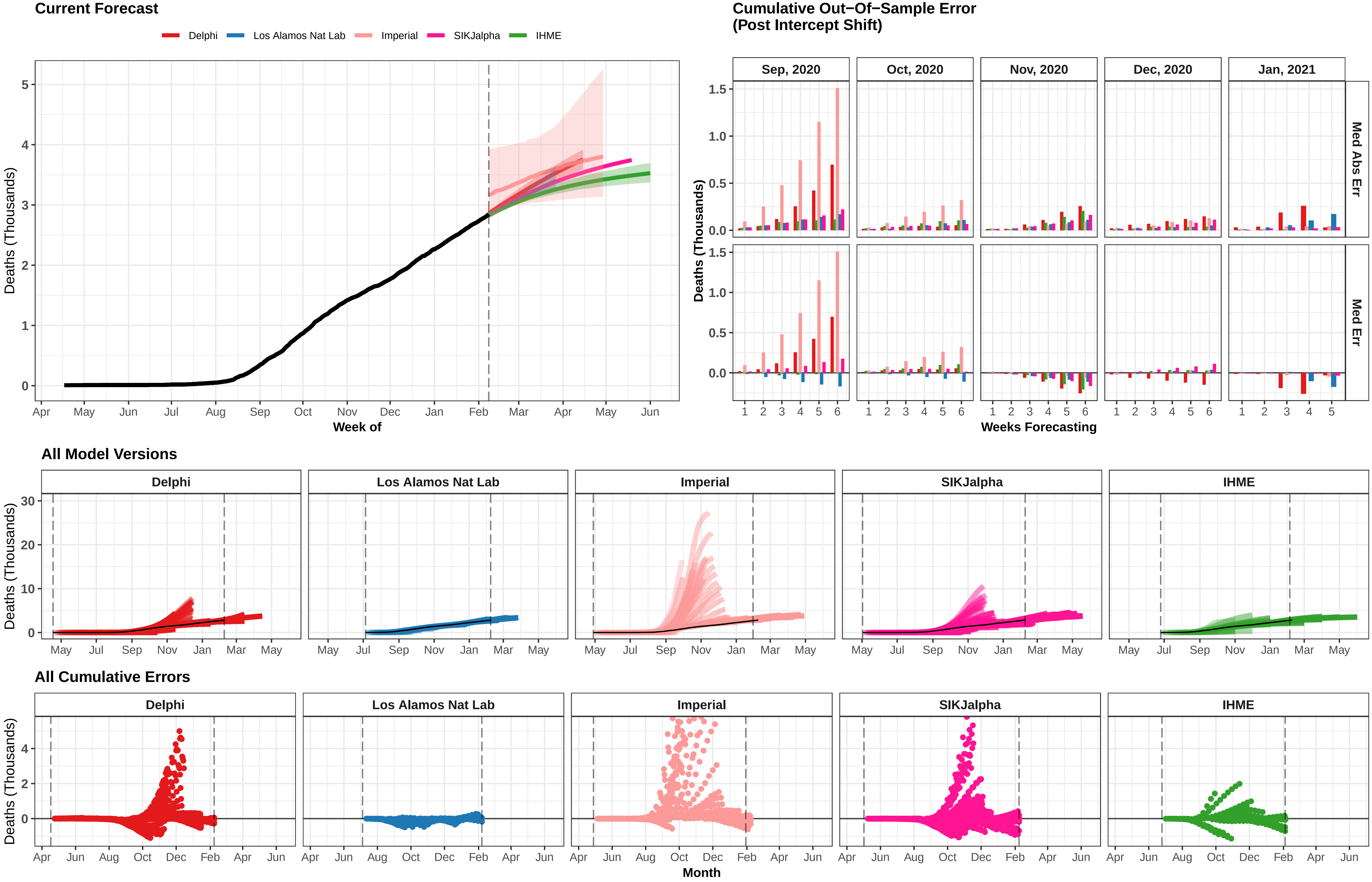

Current Forecast
Delphi
Los Alamos Nat Lab
Imperial
SIKJalpha
IHME
Deaths (Thousands)
Week of
Apr
May
Jun
Jul
Aug
Sep
Oct
Nov
Dec
Jan
Feb
Mar
Cumulative Out−Of−Sample Error
(Post Intercept Shift)
Sep, 2020
Oct, 2020
Nov, 2020
Dec, 2020
Jan, 2021
Med Abs Err
Med Err
Weeks Forecasting
All Model Versions
All Cumulative Errors
Month

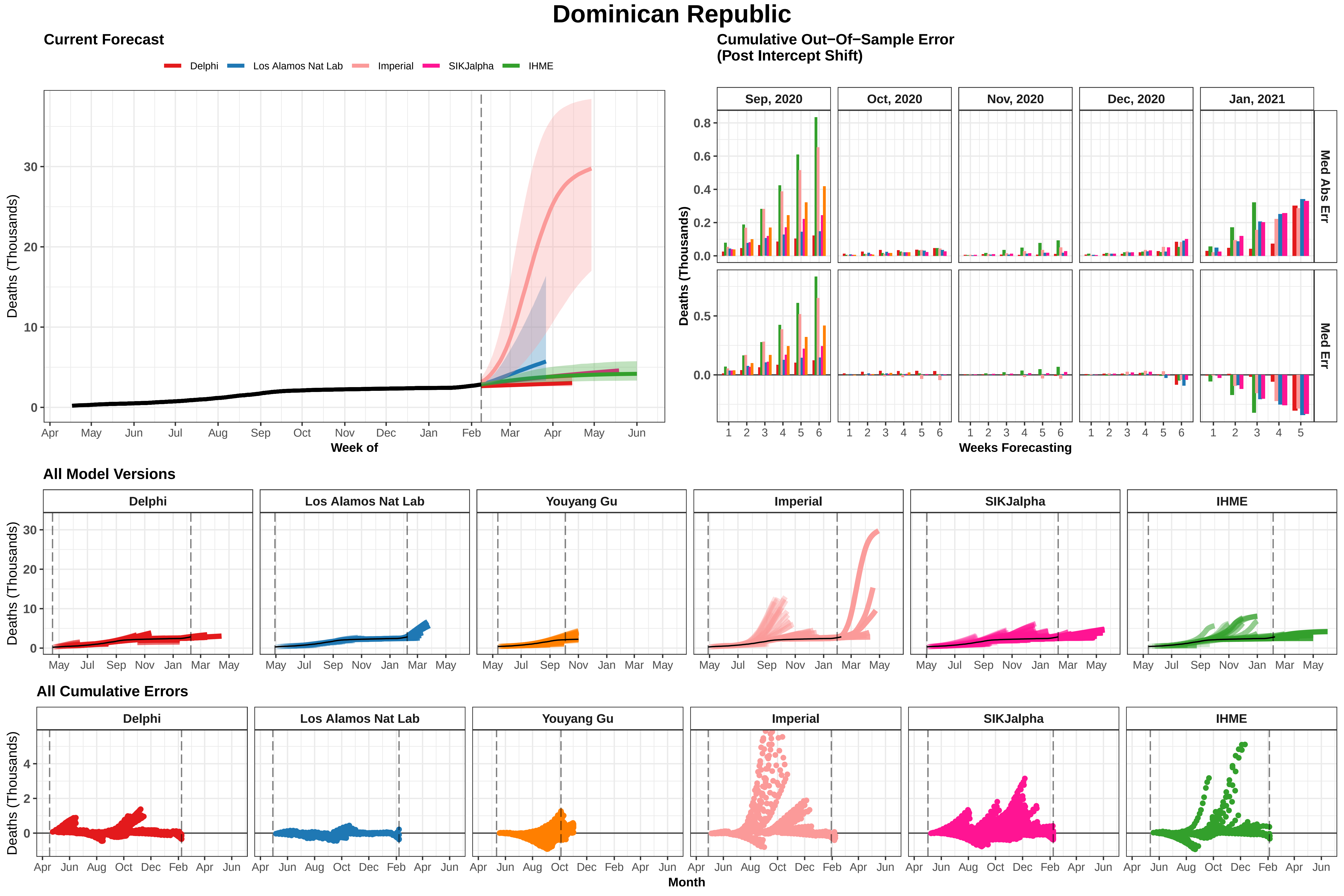

Dominican Republic
Current Forecast
Delphi
Los Alamos Nat Lab
Imperial
SIKJalpha
IHME
Deaths (Thousands)
Week of
Cumulative Out−Of−Sample Error
(Post Intercept Shift)
Sep, 2020
Oct, 2020
Nov, 2020
Dec, 2020
Jan, 2021
Med Abs Err
Med Err
Weeks Forecasting
All Model Versions
Delphi
Los Alamos Nat Lab
Youyang Gu
Imperial
SIKJalpha
IHME
All Cumulative Errors
Month

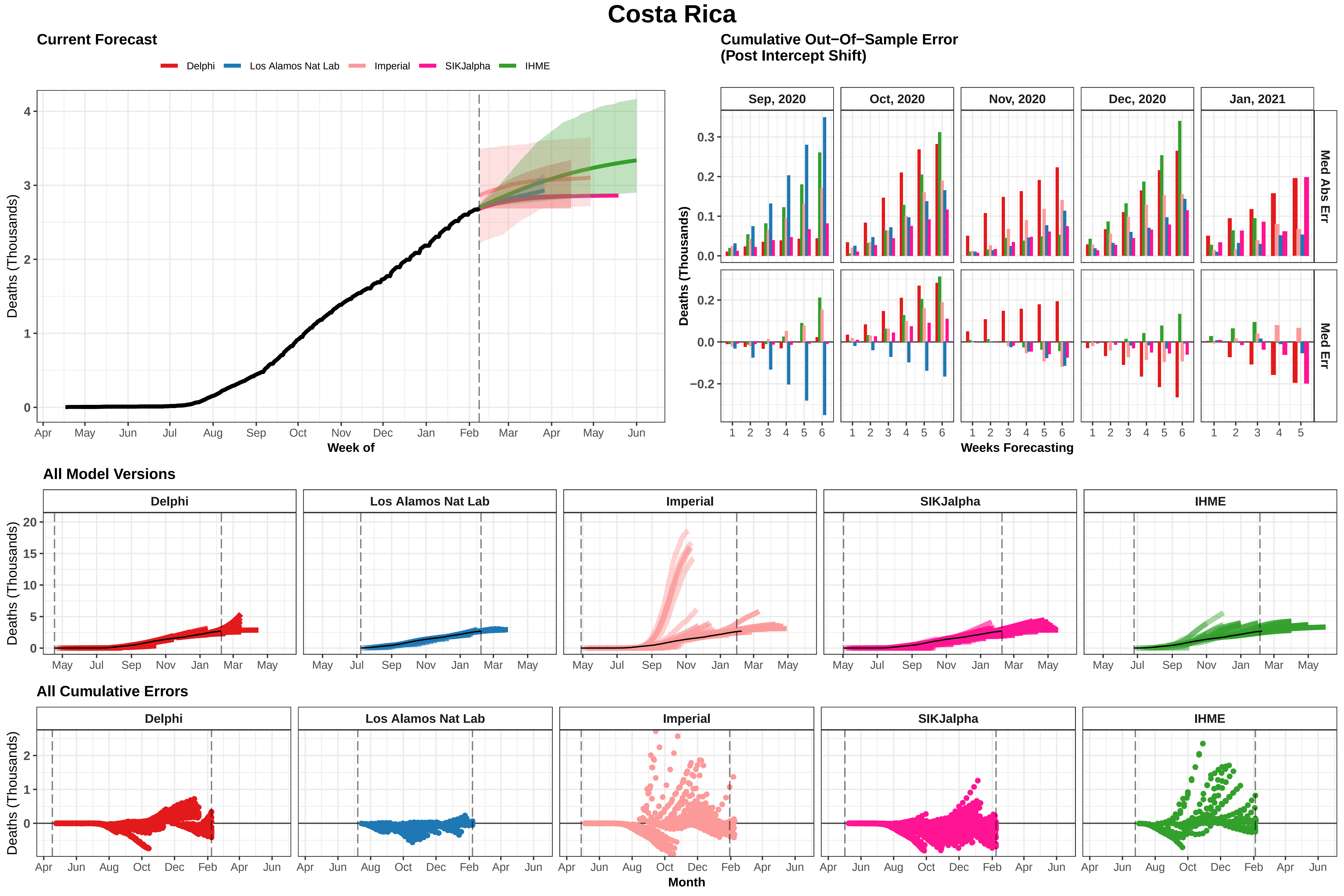

Costa Rica
Current Forecast
Delphi
Los Alamos Nat Lab
Imperial
SIKJalpha
IHME
Deaths (Thousands)
Week of
Apr May Jun Jul Aug Sep Oct Nov Dec Jan Feb Mar Apr May Jun
Cumulative Out−Of−Sample Error
(Post Intercept Shift)
Sep, 2020
Oct, 2020
Nov, 2020
Dec, 2020
Jan, 2021
Med Abs Err
Med Err
Weeks Forecasting
All Model Versions
All Cumulative Errors
Month

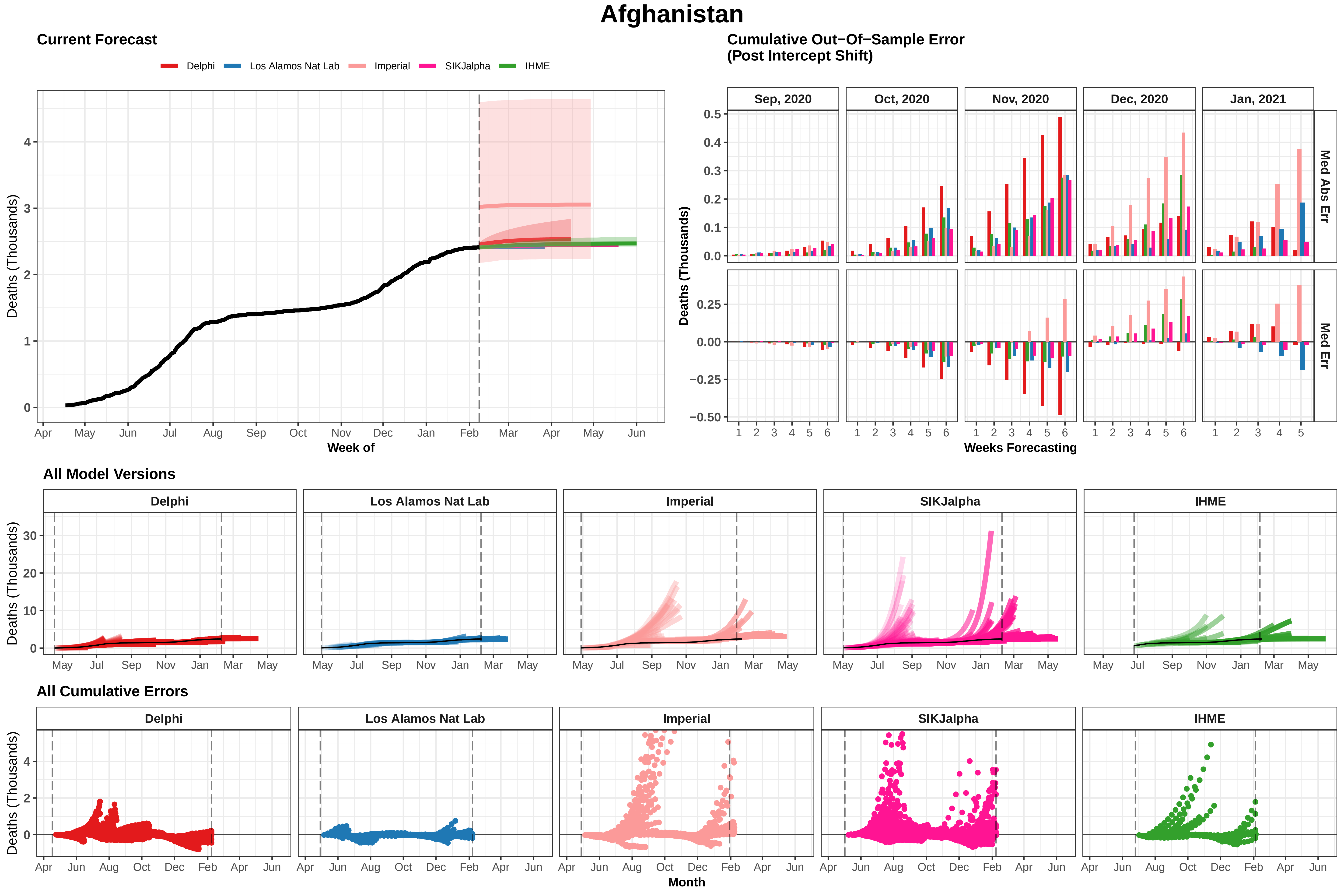

Afghanistan
Current Forecast
Delphi
Los Alamos Nat Lab
Imperial
SIKJalpha
IHME
Deaths (Thousands)
Week of
Apr May Jun Jul Aug Sep Oct Nov Dec Jan Feb Mar Apr May Jun
Cumulative Out−Of−Sample Error
(Post Intercept Shift)
Sep, 2020
Oct, 2020
Nov, 2020
Dec, 2020
Jan, 2021
Med Abs Err
Med Err
Weeks Forecasting
All Model Versions
All Cumulative Errors
Month

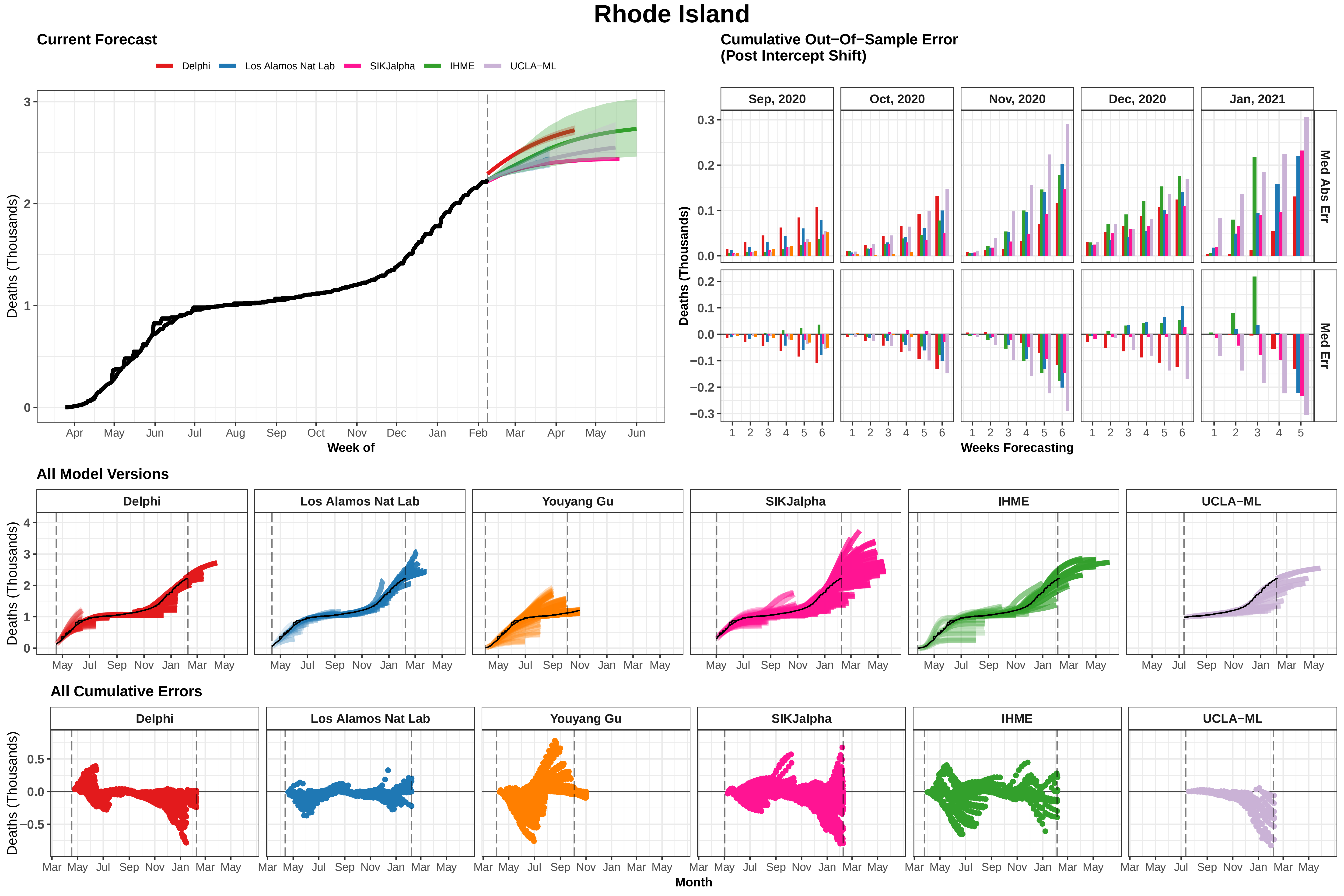
Rhode Island
Current Forecast
Delphi
Los Alamos Nat Lab
SIKJalpha
IHME
UCLA−ML
Deaths (Thousands)
Week of
Apr May Jun Jul Aug Sep Oct Nov Dec Jan Feb Mar Apr May Jun
Cumulative Out−Of−Sample Error
(Post Intercept Shift)
Sep, 2020
Oct, 2020
Nov, 2020
Dec, 2020
Jan, 2021
Med Abs Err
Med Err
Weeks Forecasting
All Model Versions
Delphi
Los Alamos Nat Lab
Youyang Gu
SIKJalpha
IHME
UCLA−ML
All Cumulative Errors
Month
Mar May Jul Sep Nov Jan Mar May

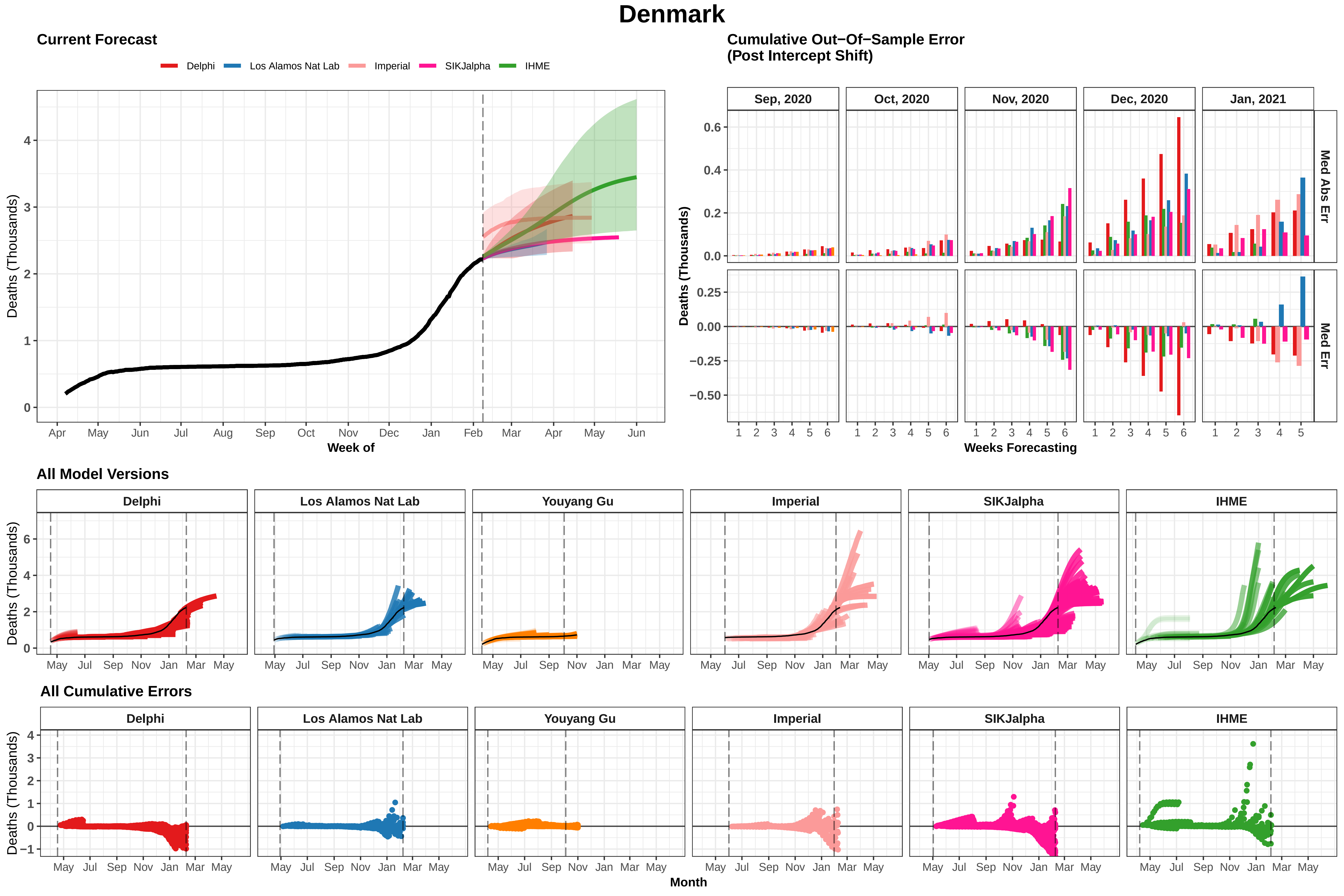

Denmark
Current Forecast
Delphi
Los Alamos Nat Lab
Imperial
SIKJalpha
IHME
Deaths (Thousands)
Week of
Apr May Jun Jul Aug Sep Oct Nov Dec Jan Feb Mar Apr May Jun
Cumulative Out−Of−Sample Error
(Post Intercept Shift)
Sep, 2020
Oct, 2020
Nov, 2020
Dec, 2020
Jan, 2021
Med Abs Err
Med Err
Weeks Forecasting
All Model Versions
Delphi
Los Alamos Nat Lab
Youyang Gu
Imperial
SIKJalpha
IHME
All Cumulative Errors
Month

Ethiopia

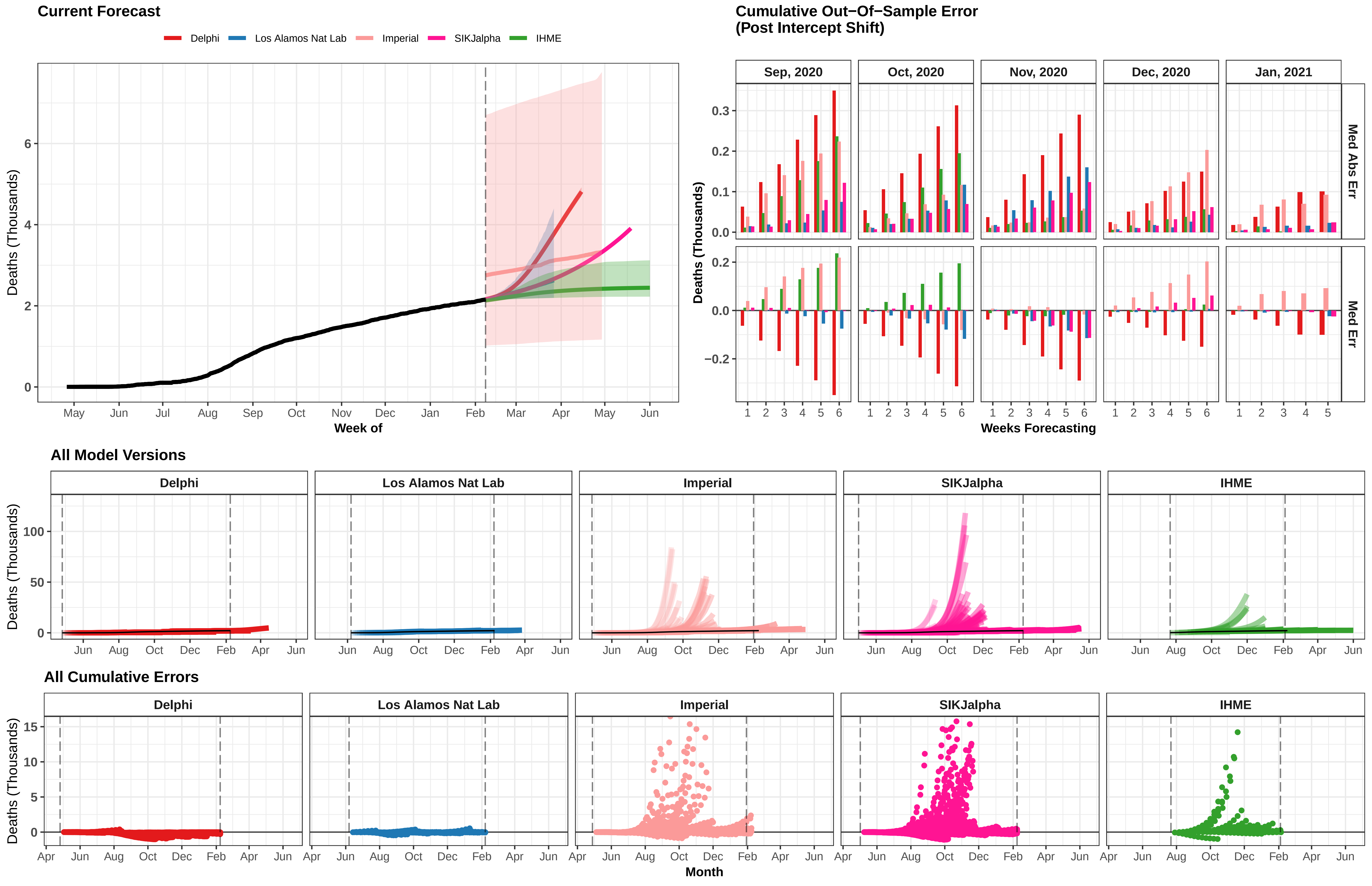

Current Forecast
Delphi
Los Alamos Nat Lab
Imperial
SIKJalpha
IHME
Deaths (Thousands)
Week of
May Jun Jul Aug Sep Oct Nov Dec Jan Feb Mar Apr May Jun
Cumulative Out−Of−Sample Error
(Post Intercept Shift)
Sep, 2020
Oct, 2020
Nov, 2020
Dec, 2020
Jan, 2021
Med Abs Err
Med Err
Weeks Forecasting
All Model Versions
All Cumulative Errors
Month

West Virginia

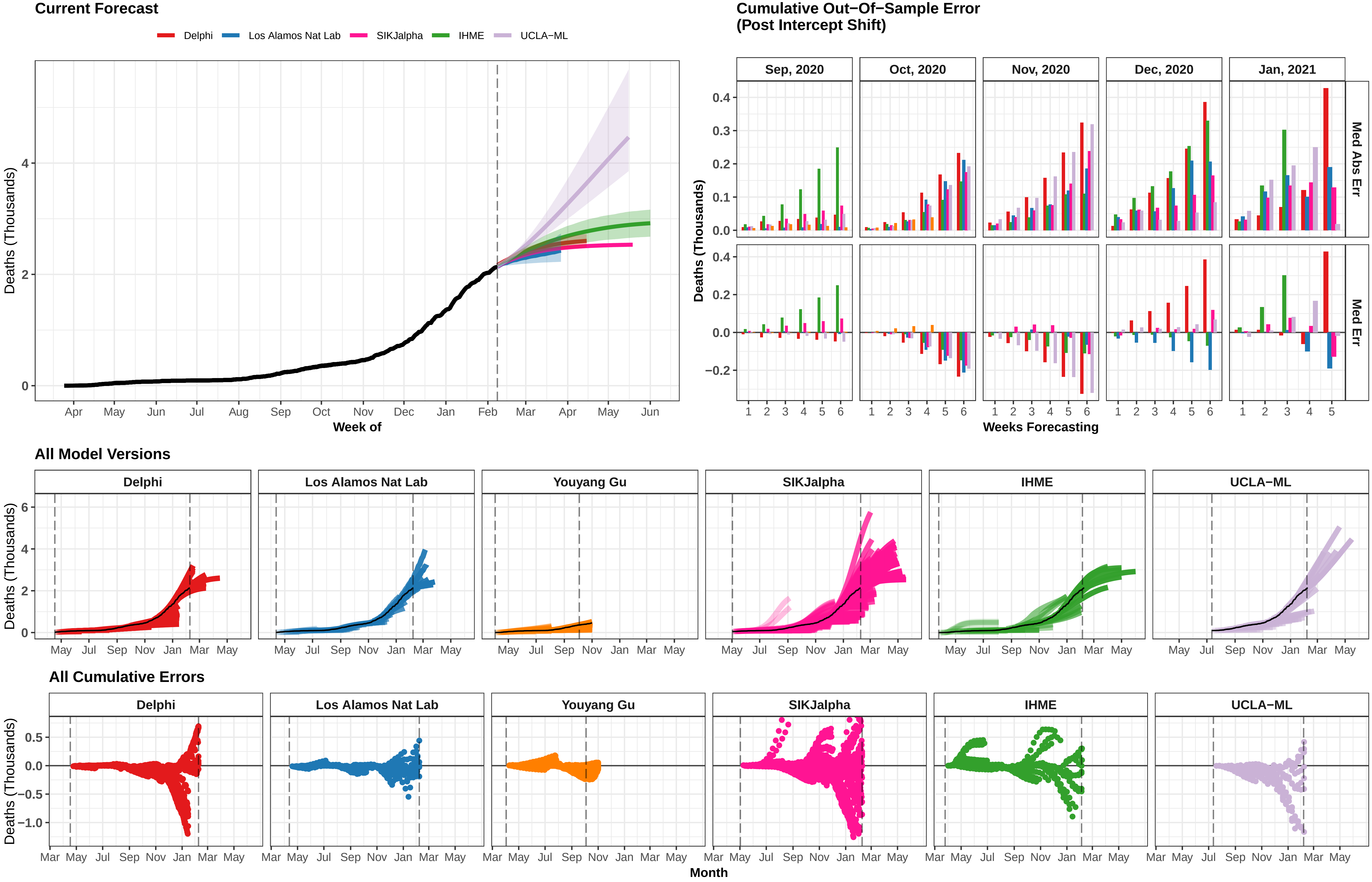

Nepal

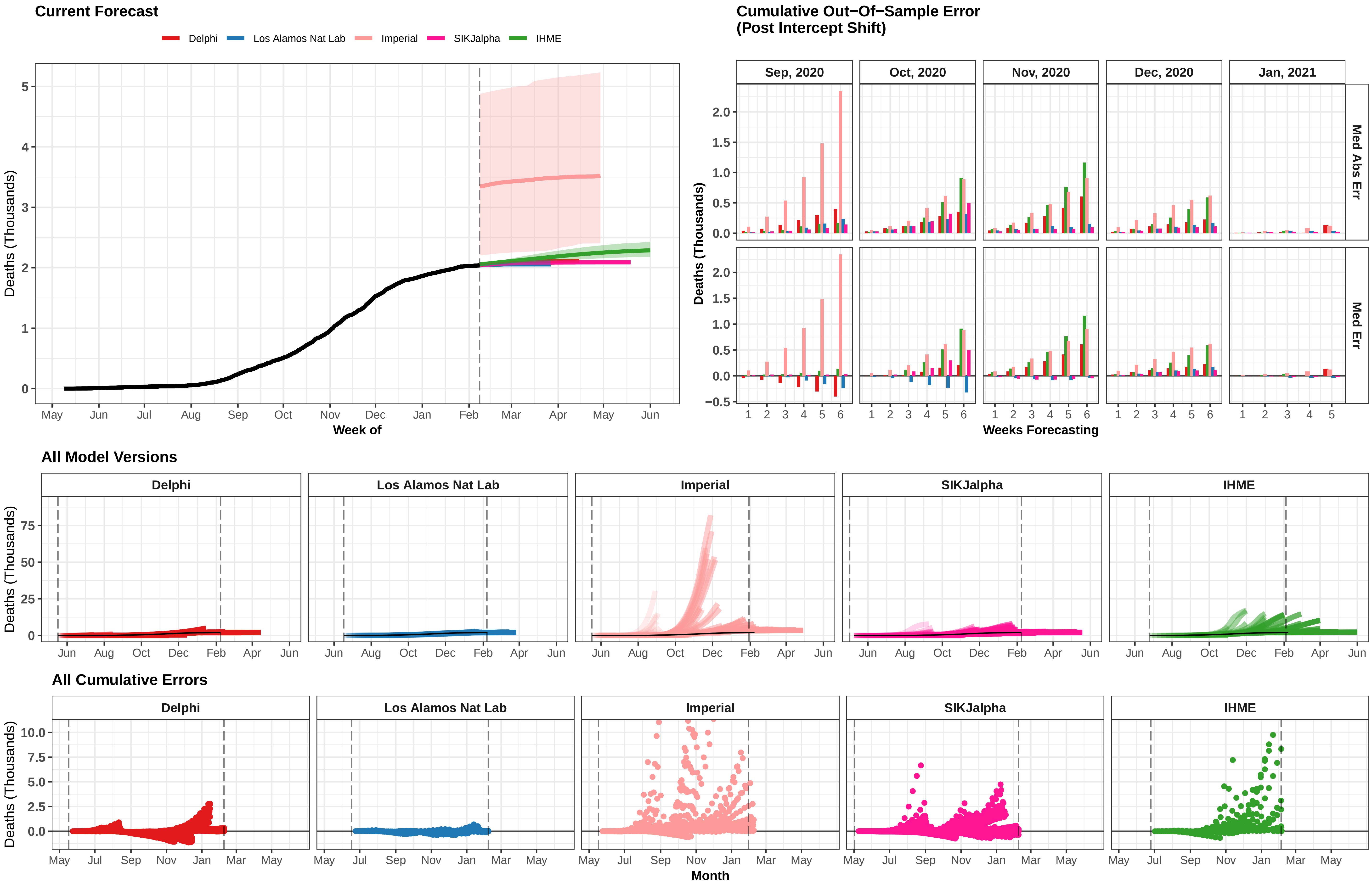
Current Forecast
Delphi
Los Alamos Nat Lab
Imperial
SIKJalpha
IHME
Deaths (Thousands)
Week of
Cumulative Out−Of−Sample Error
(Post Intercept Shift)
Sep, 2020
Oct, 2020
Nov, 2020
Dec, 2020
Jan, 2021
Med Abs Err
Med Err
Weeks Forecasting
All Model Versions
All Cumulative Errors
Month

Oregon

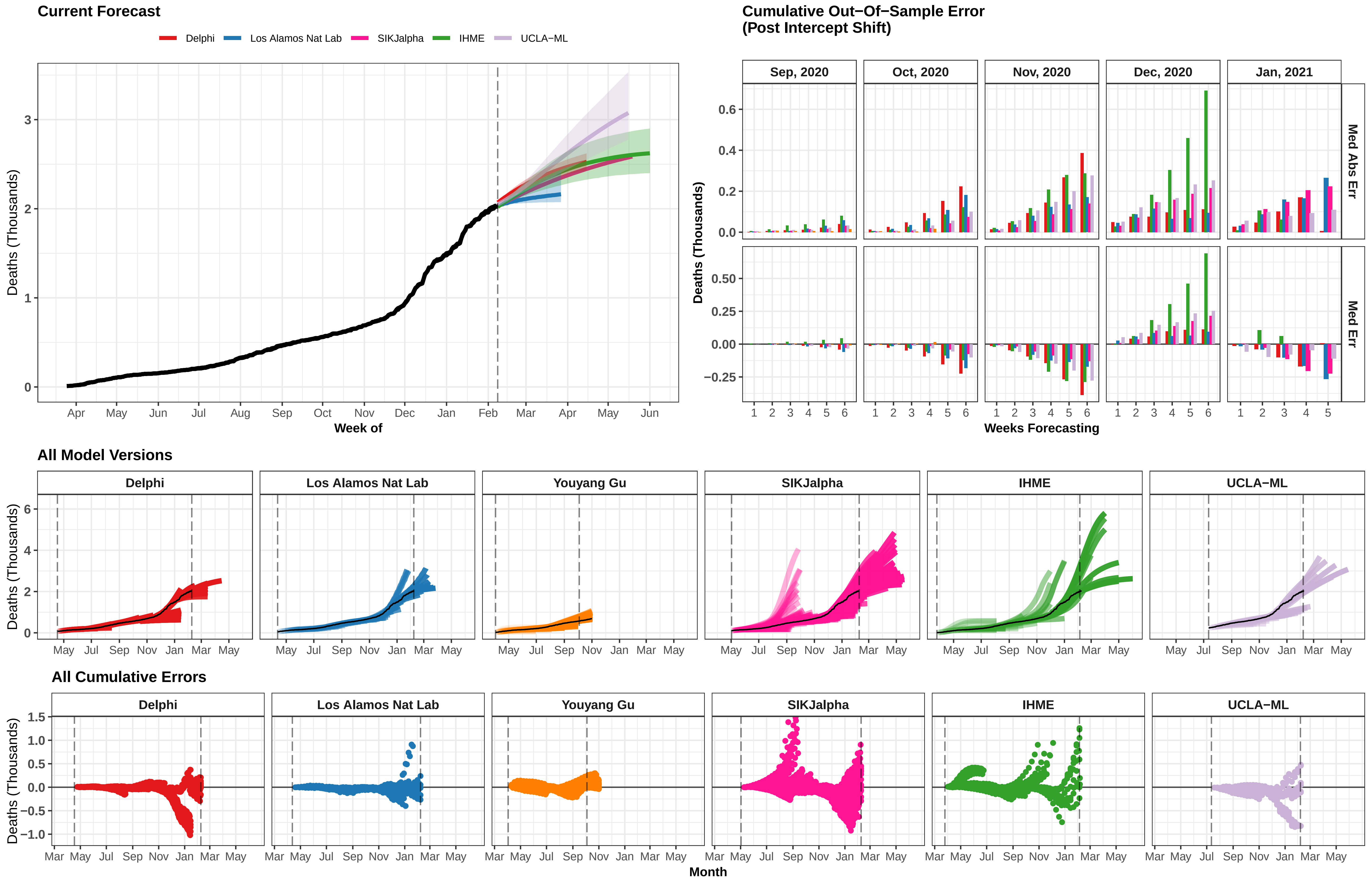
Current Forecast
Delphi
Los Alamos Nat Lab
SIKJalpha
IHME
UCLA−ML
Deaths (Thousands)
Week of
Apr May Jun Jul Aug Sep Oct Nov Dec Jan Feb Mar Apr May Jun
Cumulative Out−Of−Sample Error
(Post Intercept Shift)
Sep, 2020
Oct, 2020
Nov, 2020
Dec, 2020
Jan, 2021
Med Abs Err
Med Err
Weeks Forecasting
All Model Versions
Youyang Gu
All Cumulative Errors
Month

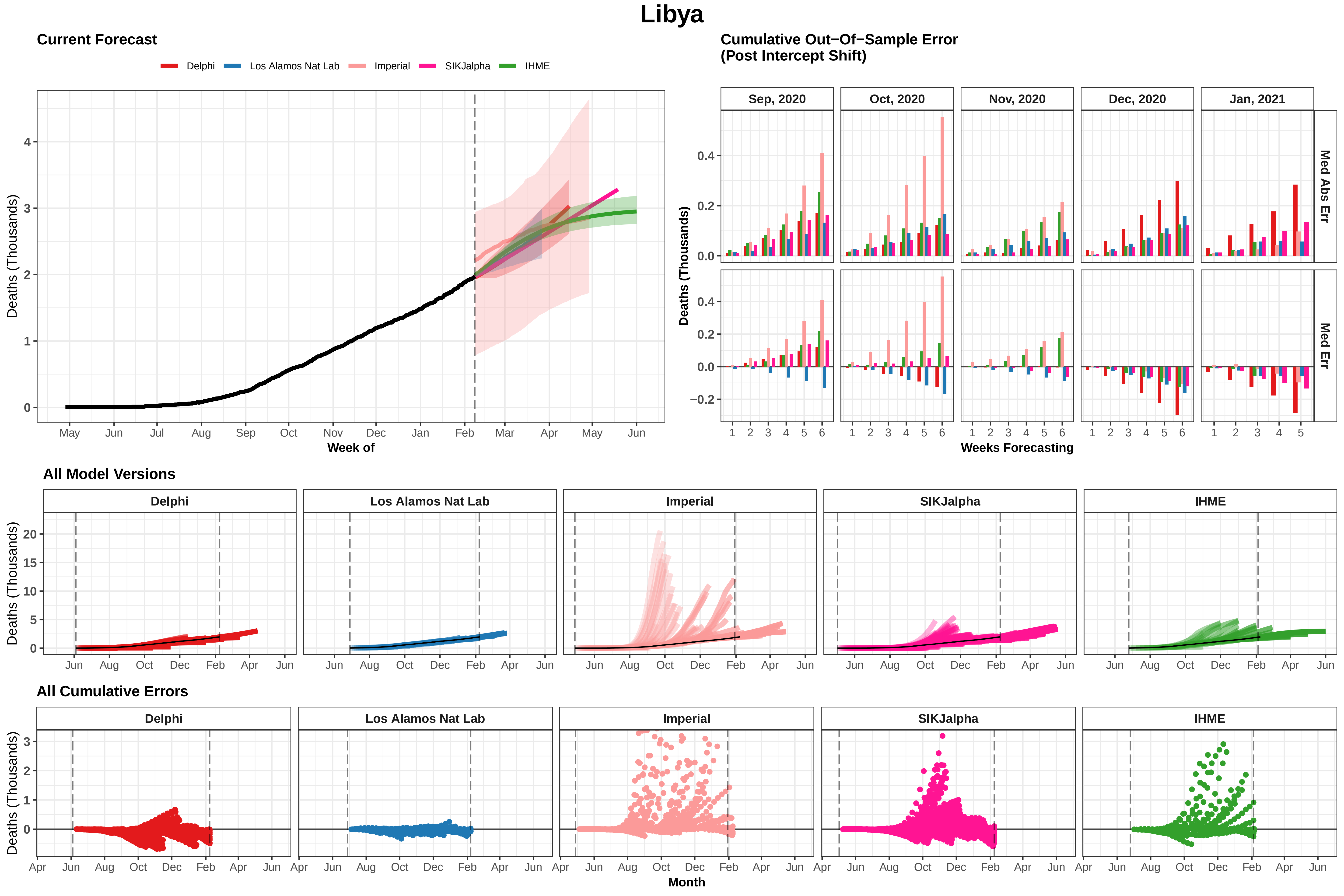

Libya
Current Forecast
Delphi
Los Alamos Nat Lab
Imperial
SIKJalpha
IHME
Deaths (Thousands)
Week of
Cumulative Out−Of−Sample Error
(Post Intercept Shift)
Sep, 2020
Oct, 2020
Nov, 2020
Dec, 2020
Jan, 2021
Med Abs Err
Med Err
Weeks Forecasting
All Model Versions
All Cumulative Errors
Month

Nebraska

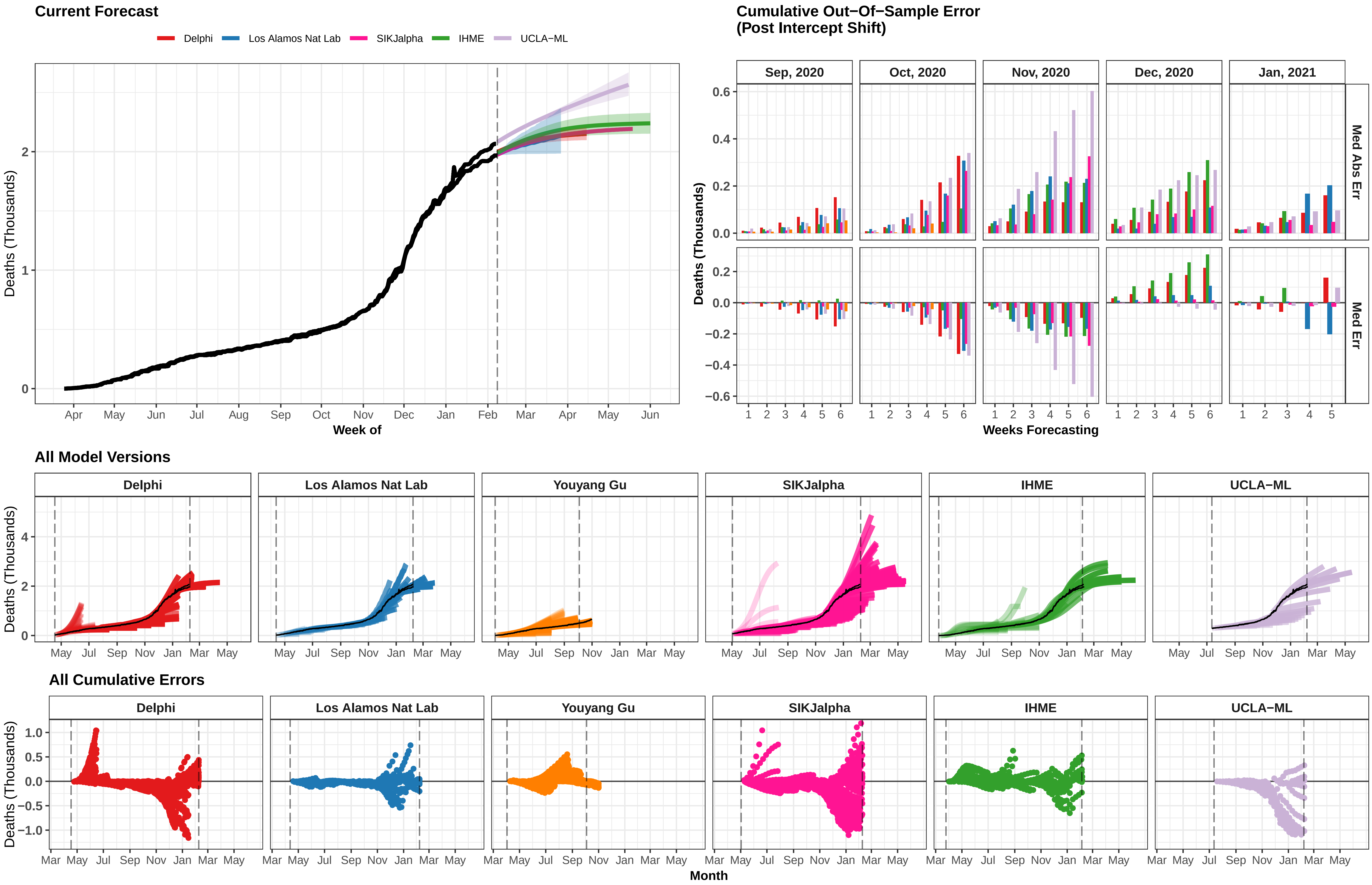

Current Forecast
Delphi
Los Alamos Nat Lab
SIKJalpha
IHME
UCLA−ML
Deaths (Thousands)
Week of
Apr May Jun Jul Aug Sep Oct Nov Dec Jan Feb Mar Apr May Jun
Cumulative Out−Of−Sample Error
(Post Intercept Shift)
Sep, 2020
Oct, 2020
Nov, 2020
Dec, 2020
Jan, 2021
Med Abs Err
Med Err
Weeks Forecasting
All Model Versions
Delphi
Los Alamos Nat Lab
Youyang Gu
SIKJalpha
IHME
UCLA−ML
All Cumulative Errors
Month

Puerto Rico

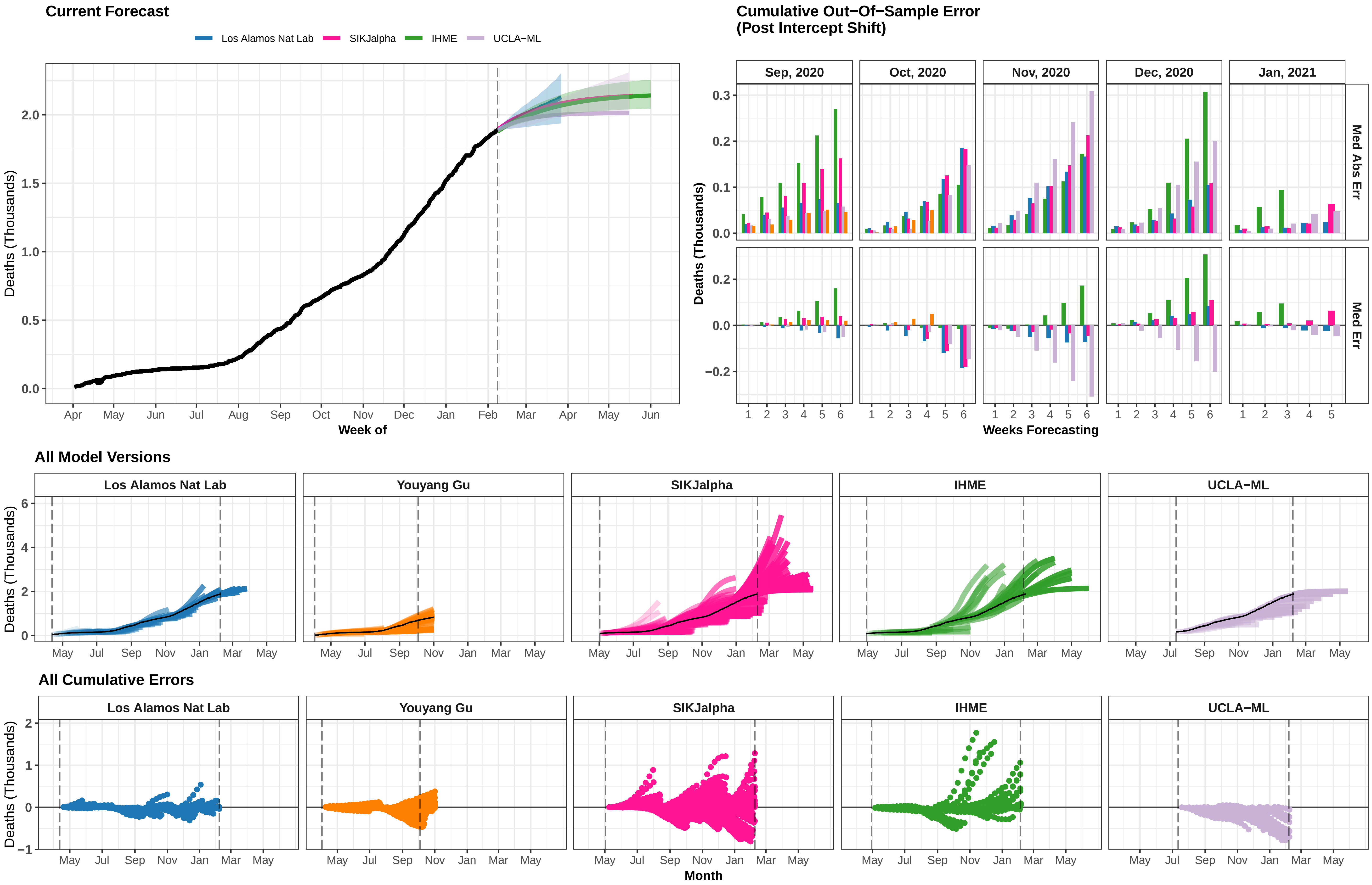

Current Forecast
Los Alamos Nat Lab
SIKJalpha
IHME
UCLA−ML
Deaths (Thousands)
Week of
Apr May Jun Jul Aug Sep Oct Nov Dec Jan Feb Mar Apr May Jun
Cumulative Out−Of−Sample Error
(Post Intercept Shift)
Sep, 2020
Oct, 2020
Nov, 2020
Dec, 2020
Jan, 2021
Med Abs Err
Med Err
Weeks Forecasting
All Model Versions
Los Alamos Nat Lab
Youyang Gu
SIKJalpha
IHME
UCLA−ML
All Cumulative Errors
Month

Sudan

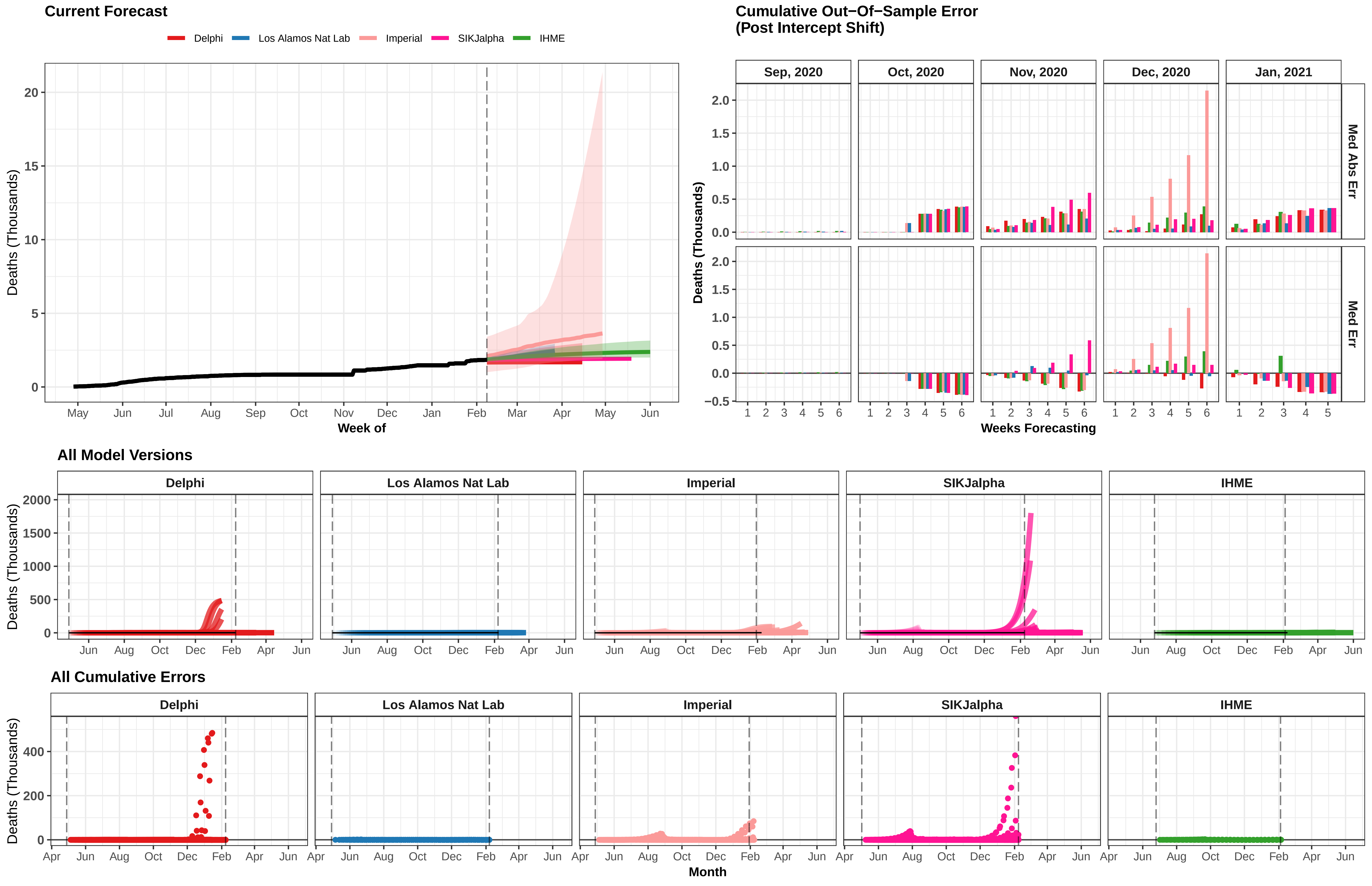

Current Forecast
Delphi
Los Alamos Nat Lab
Imperial
SIKJalpha
IHME
Deaths (Thousands)
Week of
Cumulative Out−Of−Sample Error
(Post Intercept Shift)
Sep, 2020
Oct, 2020
Nov, 2020
Dec, 2020
Jan, 2021
Med Abs Err
Med Err
Weeks Forecasting
All Model Versions
All Cumulative Errors
Month

South Dakota

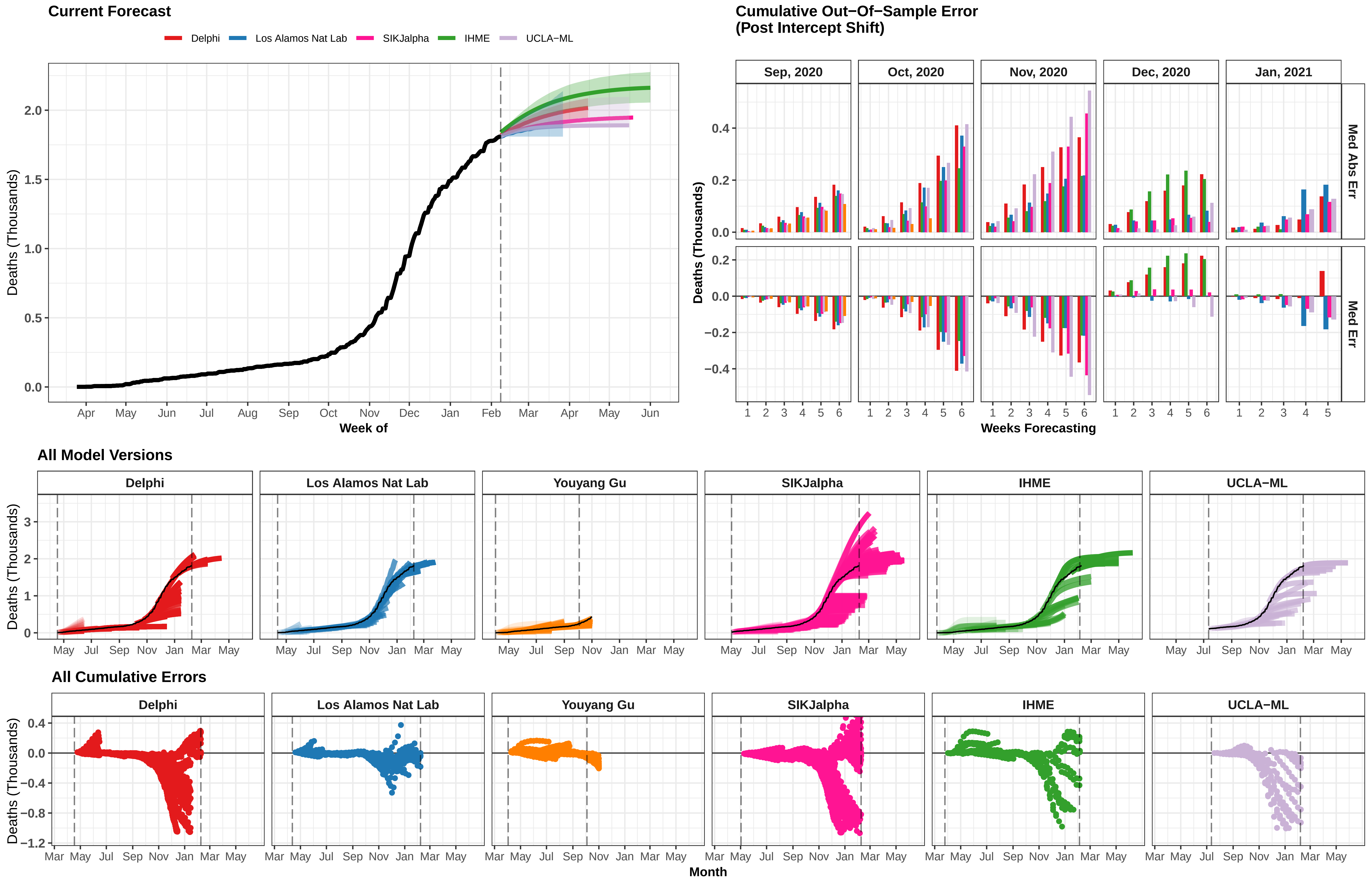

Belarus

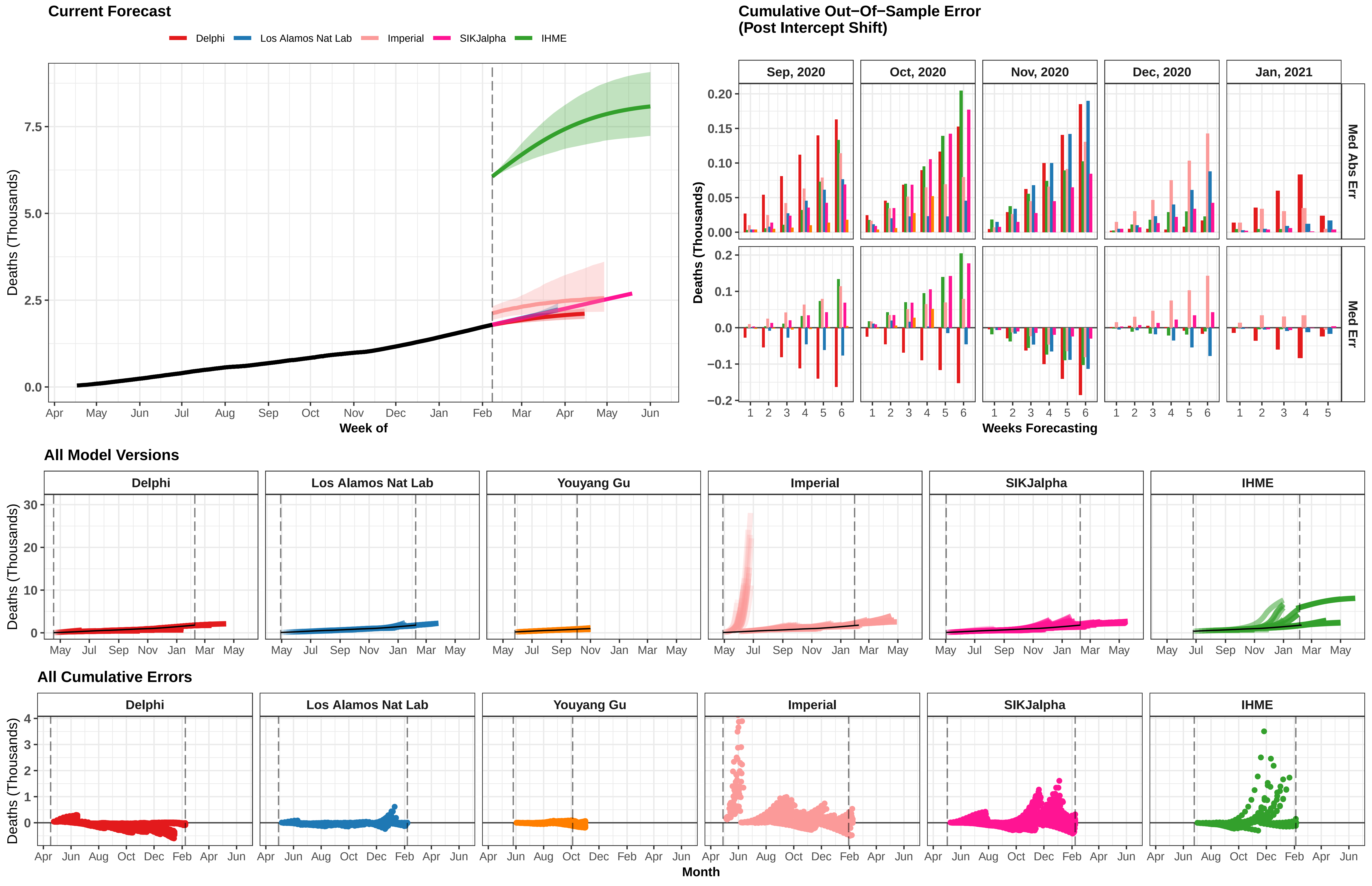

Current Forecast
Delphi
Los Alamos Nat Lab
Imperial
SIKJalpha
IHME
Deaths (Thousands)
Week of
Cumulative Out−Of−Sample Error
(Post Intercept Shift)
Sep, 2020
Oct, 2020
Nov, 2020
Dec, 2020
Jan, 2021
Med Abs Err
Med Err
Weeks Forecasting
All Model Versions
Youyang Gu
All Cumulative Errors
Month

Kenya

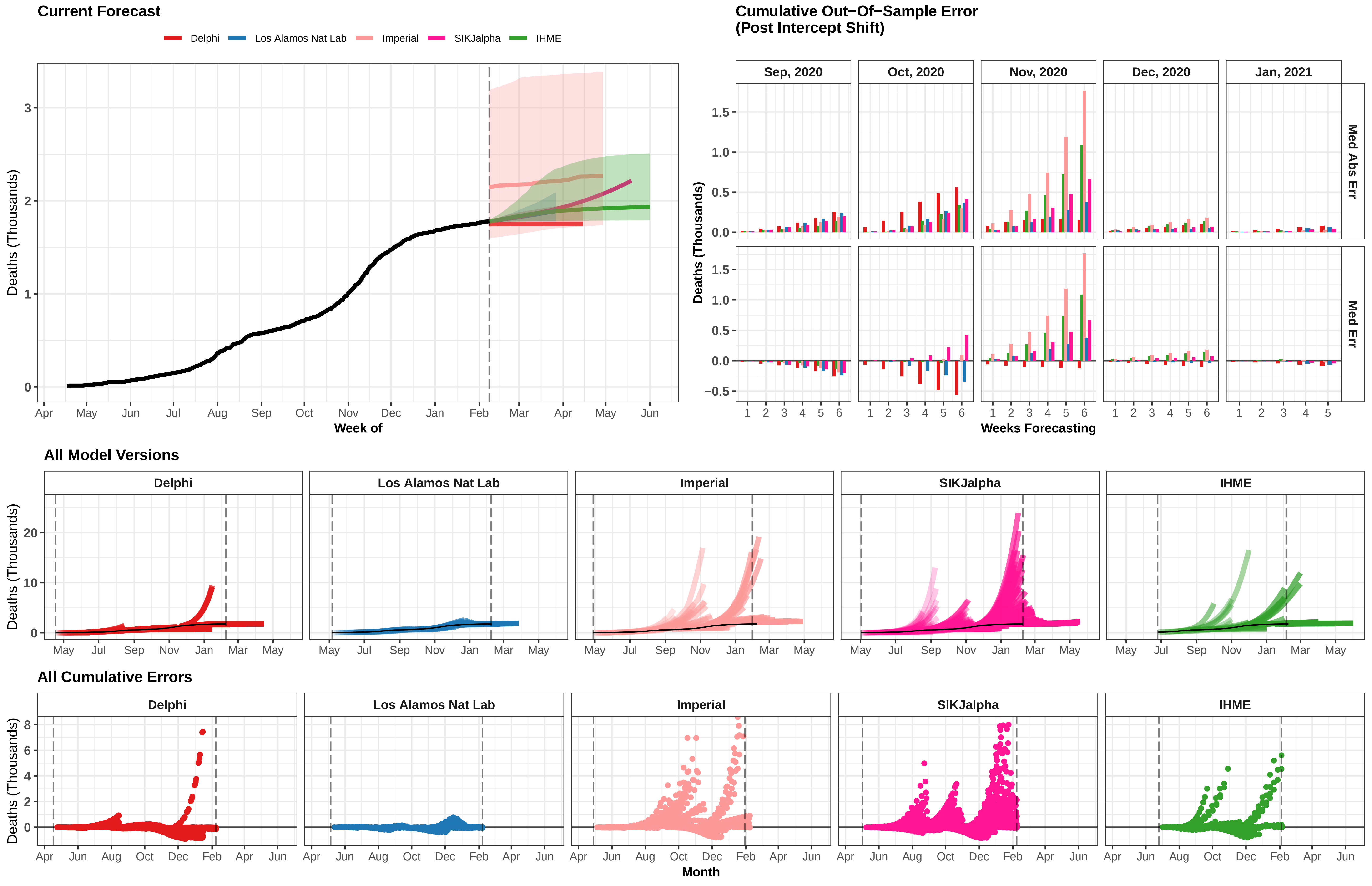

Current Forecast
Delphi
Los Alamos Nat Lab
Imperial
SIKJalpha
IHME
Deaths (Thousands)
Week of
Cumulative Out−Of−Sample Error
(Post Intercept Shift)
Sep, 2020
Oct, 2020
Nov, 2020
Dec, 2020
Jan, 2021
Med Abs Err
Med Err
Weeks Forecasting
All Model Versions
All Cumulative Errors
Month

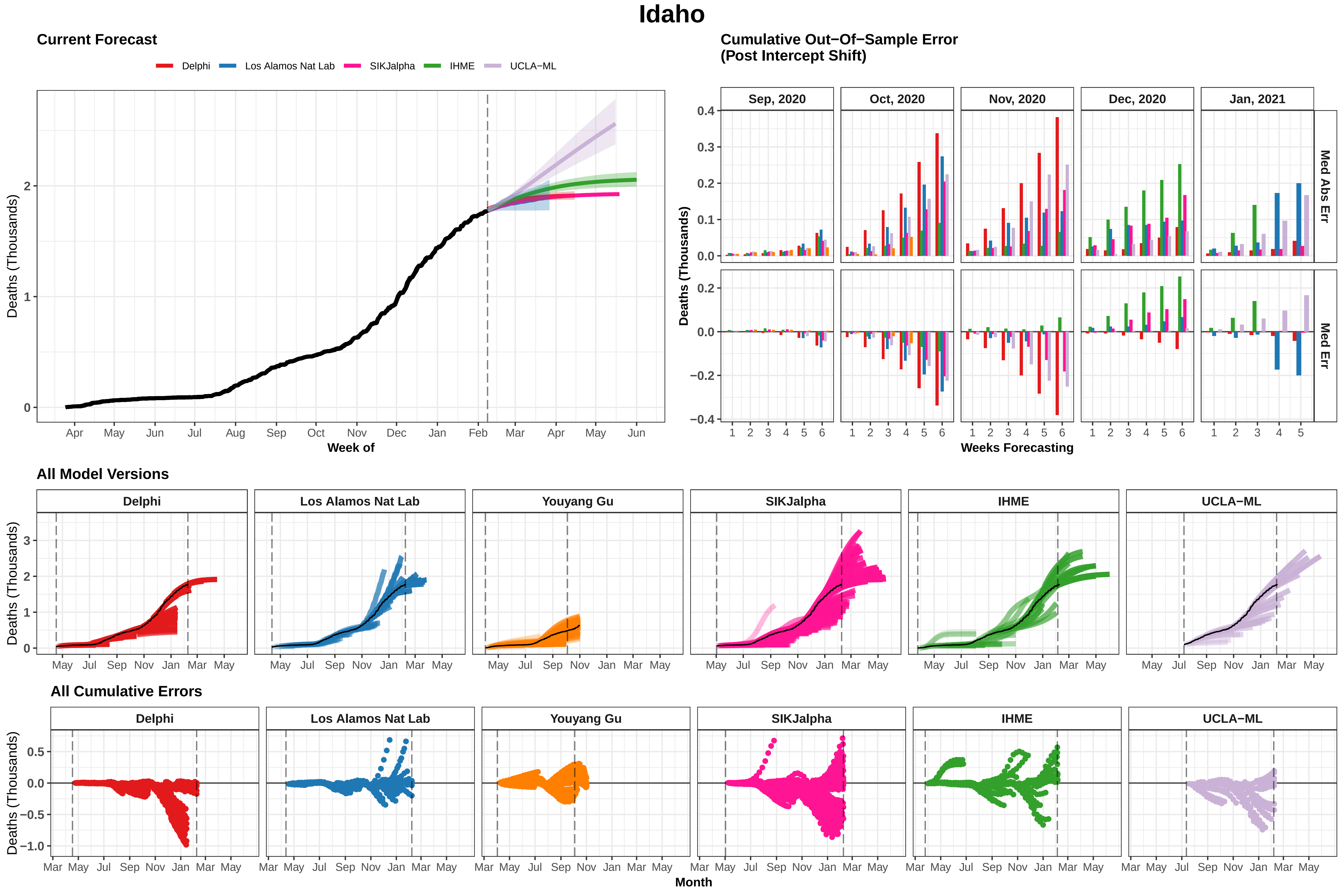

Idaho
Current Forecast
Delphi
Los Alamos Nat Lab
SIKJalpha
IHME
UCLA−ML
Deaths (Thousands)
Week of
Apr May Jun Jul Aug Sep Oct Nov Dec Jan Feb Mar Apr May Jun
Cumulative Out−Of−Sample Error
(Post Intercept Shift)
Sep, 2020
Oct, 2020
Nov, 2020
Dec, 2020
Jan, 2021
Med Abs Err
Med Err
Weeks Forecasting
All Model Versions
Delphi
Los Alamos Nat Lab
Youyang Gu
SIKJalpha
IHME
UCLA−ML
All Cumulative Errors
Month

Utah

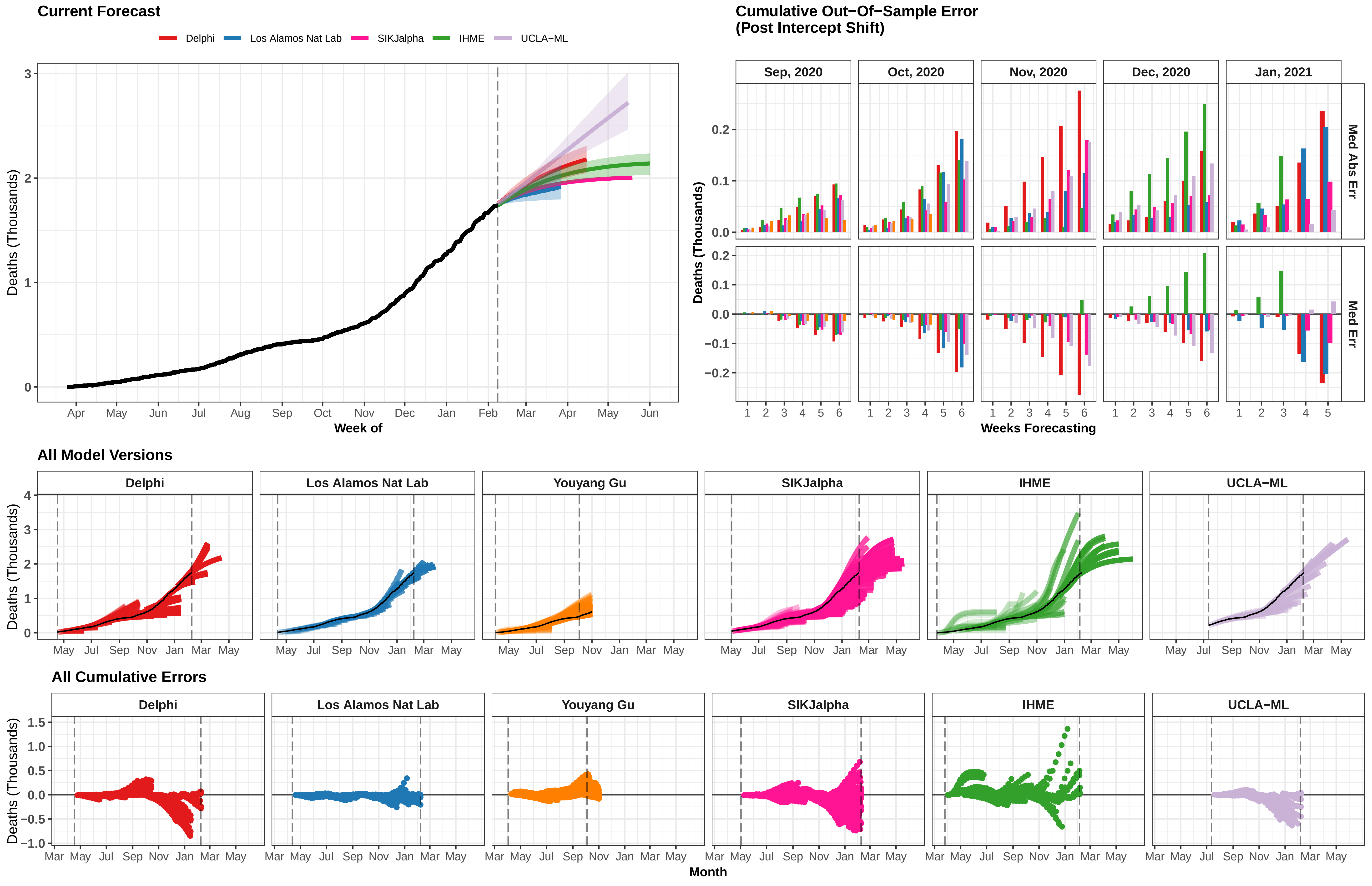

Current Forecast
Delphi
Los Alamos Nat Lab
SIKJalpha
IHME
UCLA−ML
Deaths (Thousands)
Week of
Apr May Jun Jul Aug Sep Oct Nov Dec Jan Feb Mar Apr May Jun
Cumulative Out−Of−Sample Error
(Post Intercept Shift)
Sep, 2020
Oct, 2020
Nov, 2020
Dec, 2020
Jan, 2021
Med Abs Err
Med Err
Weeks Forecasting
All Model Versions
Delphi
Los Alamos Nat Lab
Youyang Gu
SIKJalpha
IHME
UCLA−ML
All Cumulative Errors
Month

El Salvador

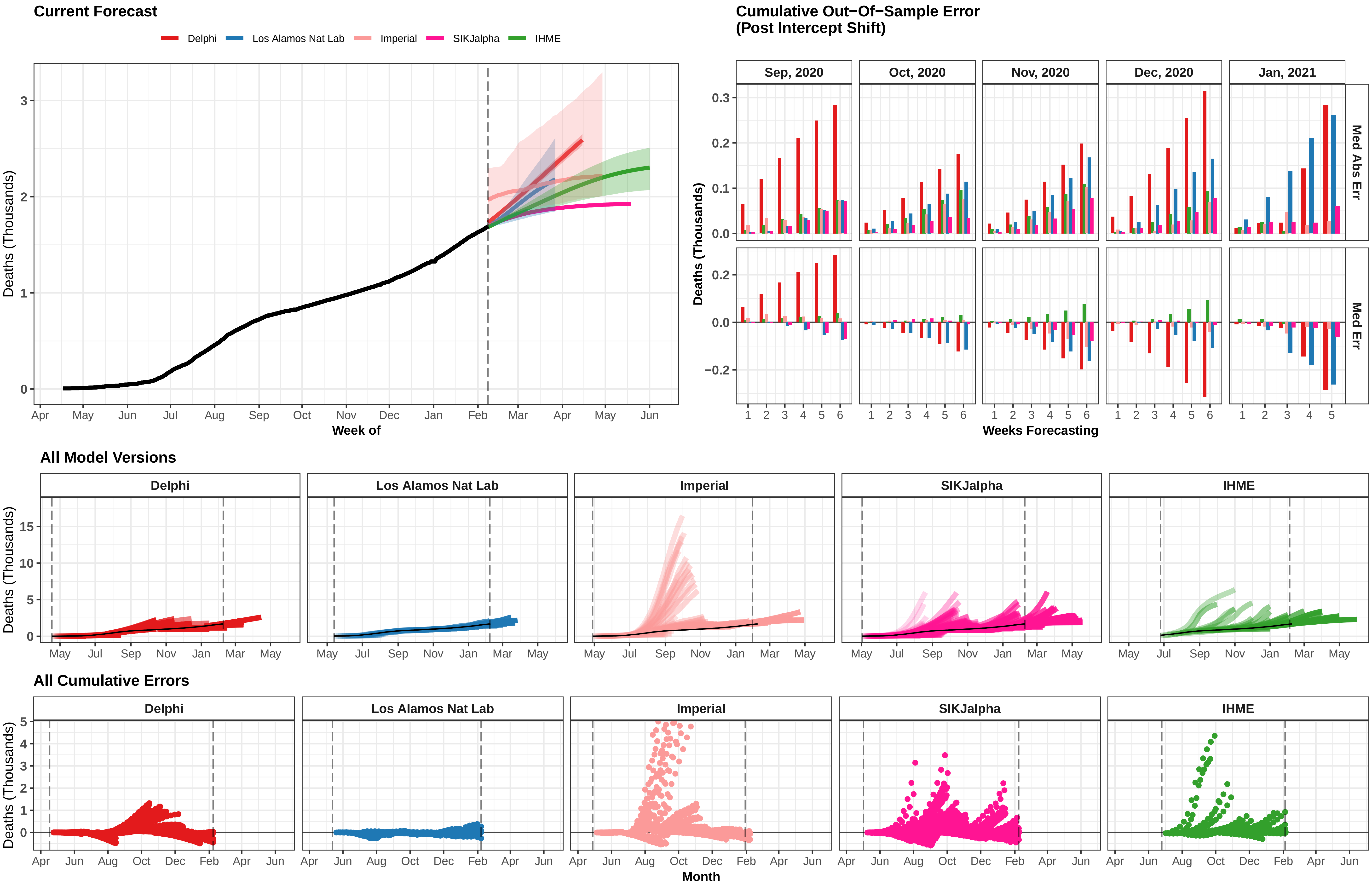
Current Forecast
Delphi
Los Alamos Nat Lab
Imperial
SIKJalpha
IHME
Deaths (Thousands)
Week of
Apr May Jun Jul Aug Sep Oct Nov Dec Jan Feb Mar Apr May Jun
Cumulative Out−Of−Sample Error
(Post Intercept Shift)
Sep, 2020
Oct, 2020
Nov, 2020
Dec, 2020
Jan, 2021
Med Abs Err
Med Err
Weeks Forecasting
All Model Versions
All Cumulative Errors
Month

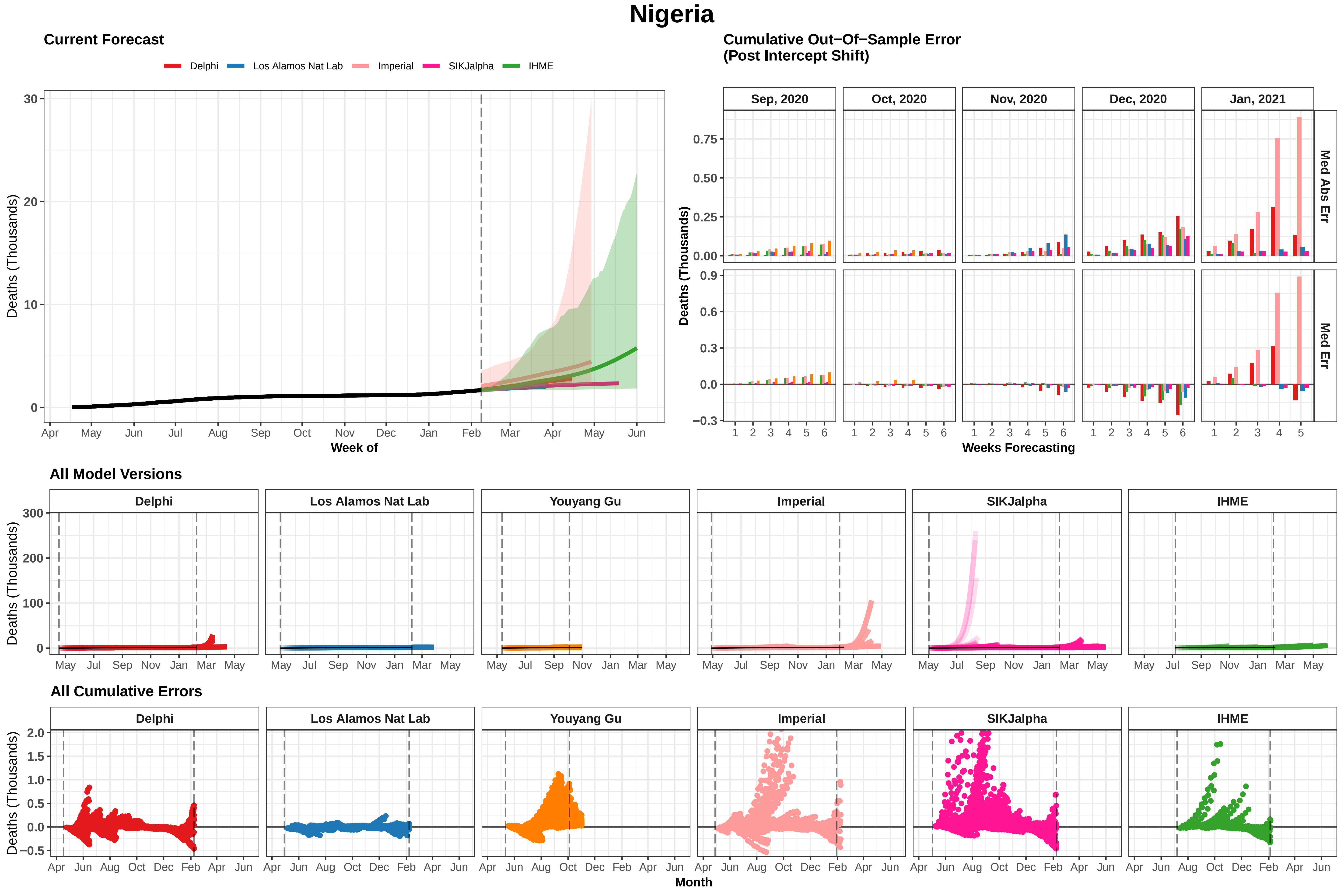
Nigeria
Current Forecast
Delphi
Los Alamos Nat Lab
Imperial
SIKJalpha
IHME
Deaths (Thousands)
Week of
Apr May Jun Jul Aug Sep Oct Nov Dec Jan Feb Mar Apr May Jun
Cumulative Out−Of−Sample Error
(Post Intercept Shift)
Sep, 2020
Oct, 2020
Nov, 2020
Dec, 2020
Jan, 2021
Med Abs Err
Med Err
Weeks Forecasting
All Model Versions
Delphi
Los Alamos Nat Lab
Youyang Gu
Imperial
SIKJalpha
IHME
All Cumulative Errors
Month

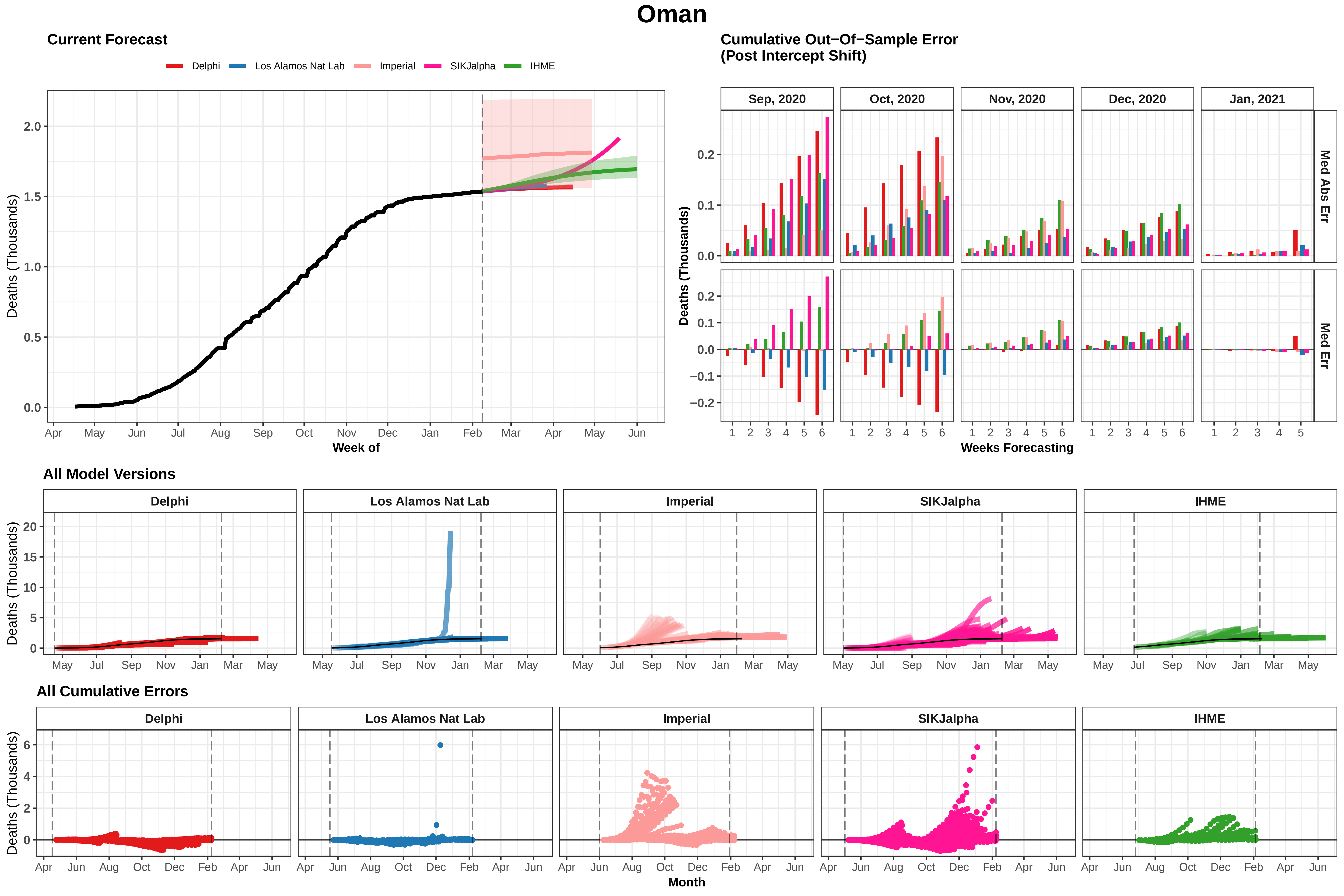
Oman
Current Forecast
Delphi
Los Alamos Nat Lab
Imperial
SIKJalpha
IHME
Deaths (Thousands)
Week of
Apr May Jun Jul Aug Sep Oct Nov Dec Jan Feb Mar Apr May Jun
Cumulative Out−Of−Sample Error
(Post Intercept Shift)
Sep, 2020
Oct, 2020
Nov, 2020
Dec, 2020
Jan, 2021
Med Abs Err
Med Err
Weeks Forecasting
All Model Versions
All Cumulative Errors
Month

South Korea

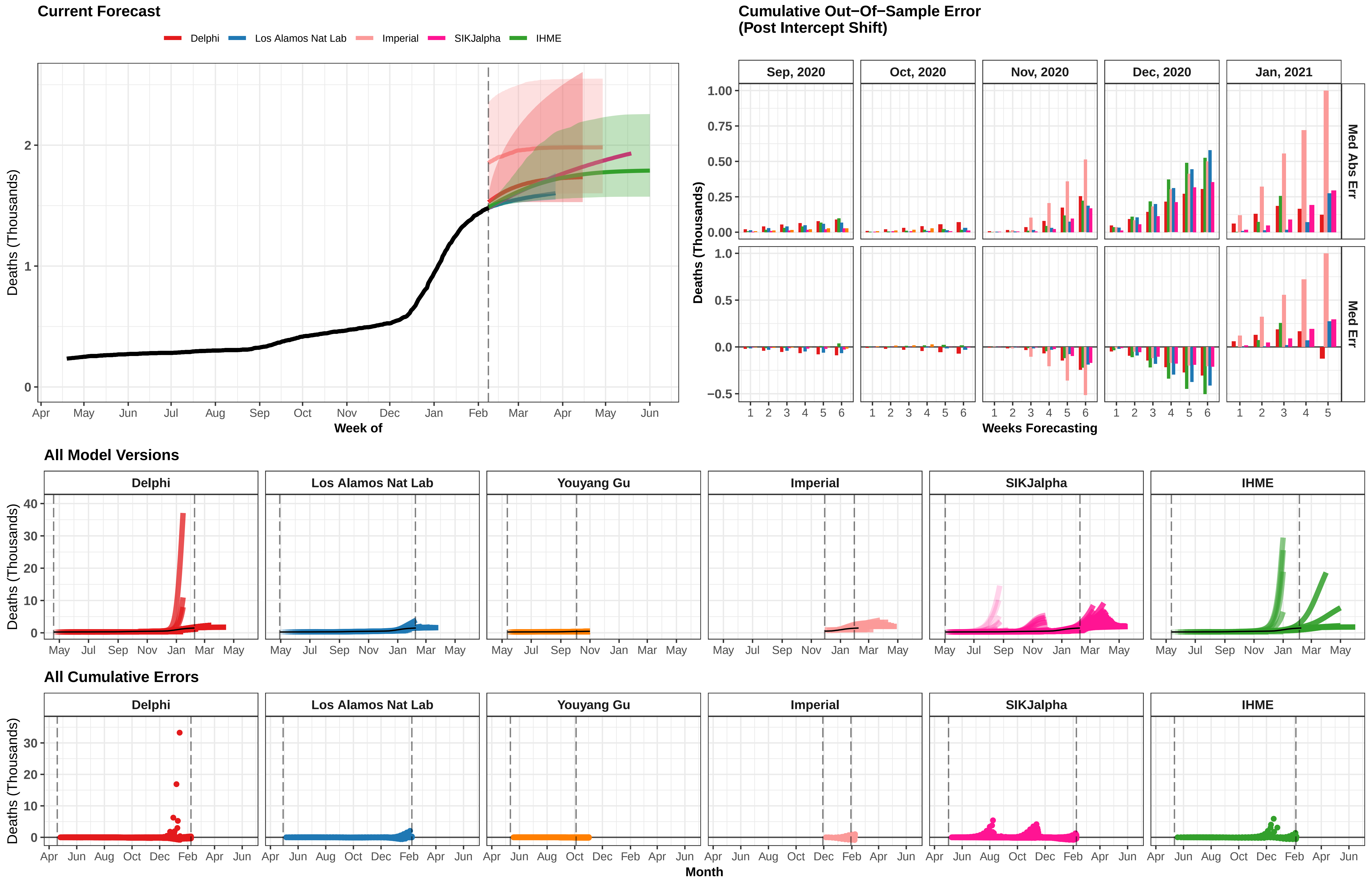
South Korea
Current Forecast
Delphi
Los Alamos Nat Lab
Imperial
SIKJalpha
IHME
Deaths (Thousands)
Week of
Apr May Jun Jul Aug Sep Oct Nov Dec Jan Feb Mar Apr May Jun
Cumulative Out−Of−Sample Error
(Post Intercept Shift)
Sep, 2020
Oct, 2020
Nov, 2020
Dec, 2020
Jan, 2021
Med Abs Err
Med Err
Weeks Forecasting
All Model Versions
Delphi
Los Alamos Nat Lab
Youyang Gu
Imperial
SIKJalpha
IHME
All Cumulative Errors
Month

Albania

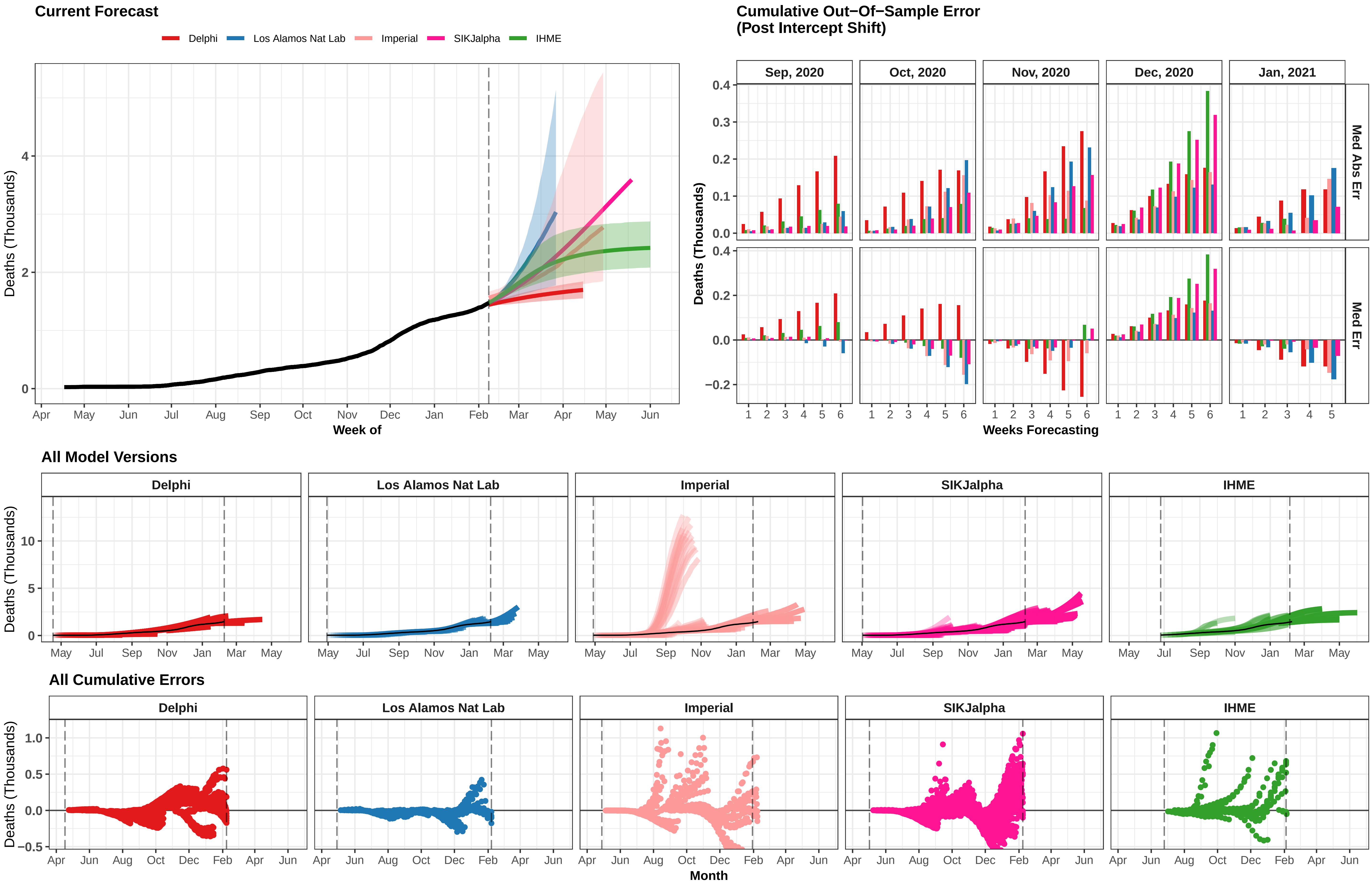
Current Forecast
Delphi
Los Alamos Nat Lab
Imperial
SIKJalpha
IHME
Deaths (Thousands)
Week of
Cumulative Out−Of−Sample Error
(Post Intercept Shift)
Sep, 2020
Oct, 2020
Nov, 2020
Dec, 2020
Jan, 2021
Med Abs Err
Med Err
Weeks Forecasting
All Model Versions
All Cumulative Errors
Month

North Dakota

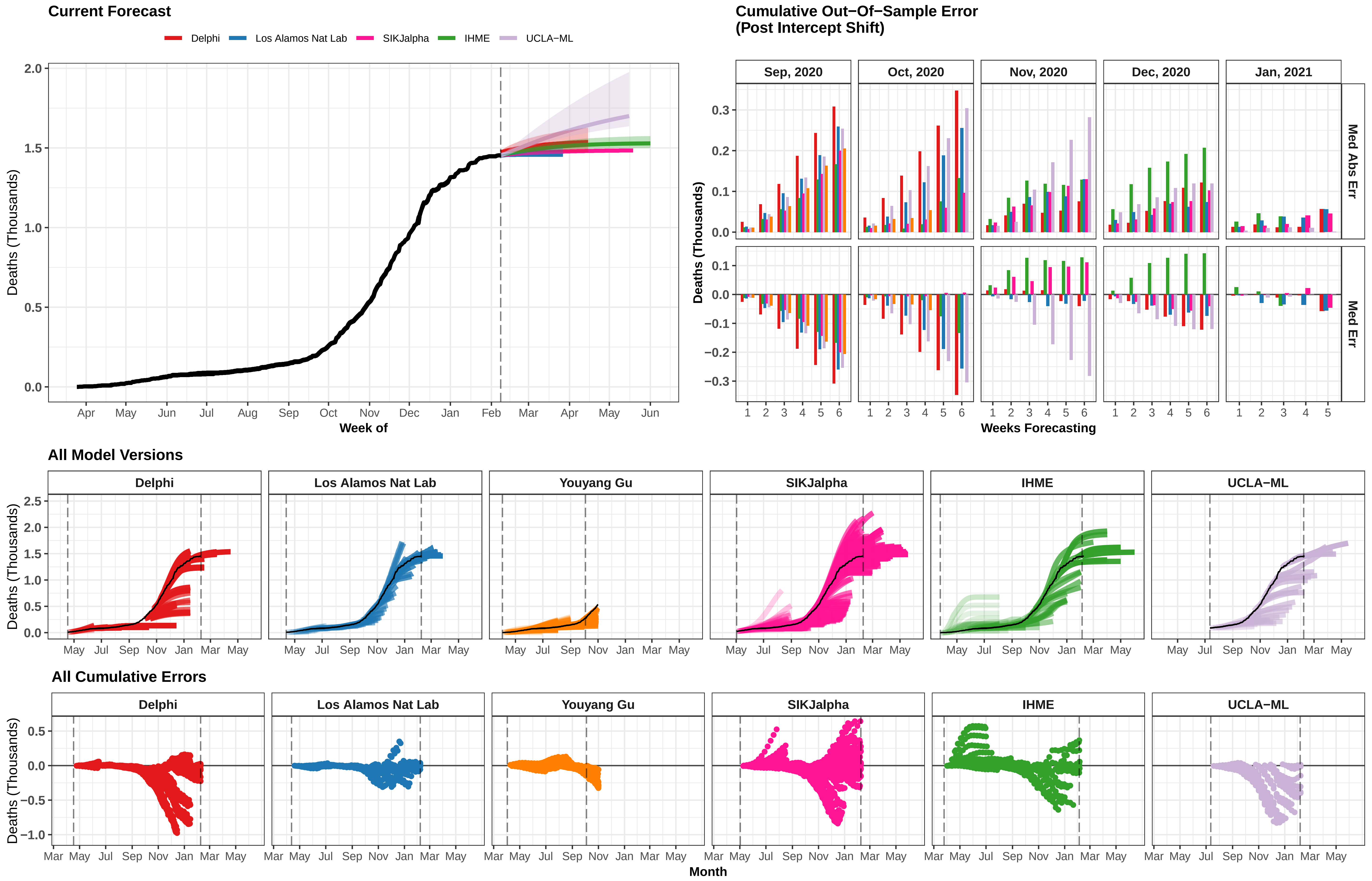

Kyrgyzstan

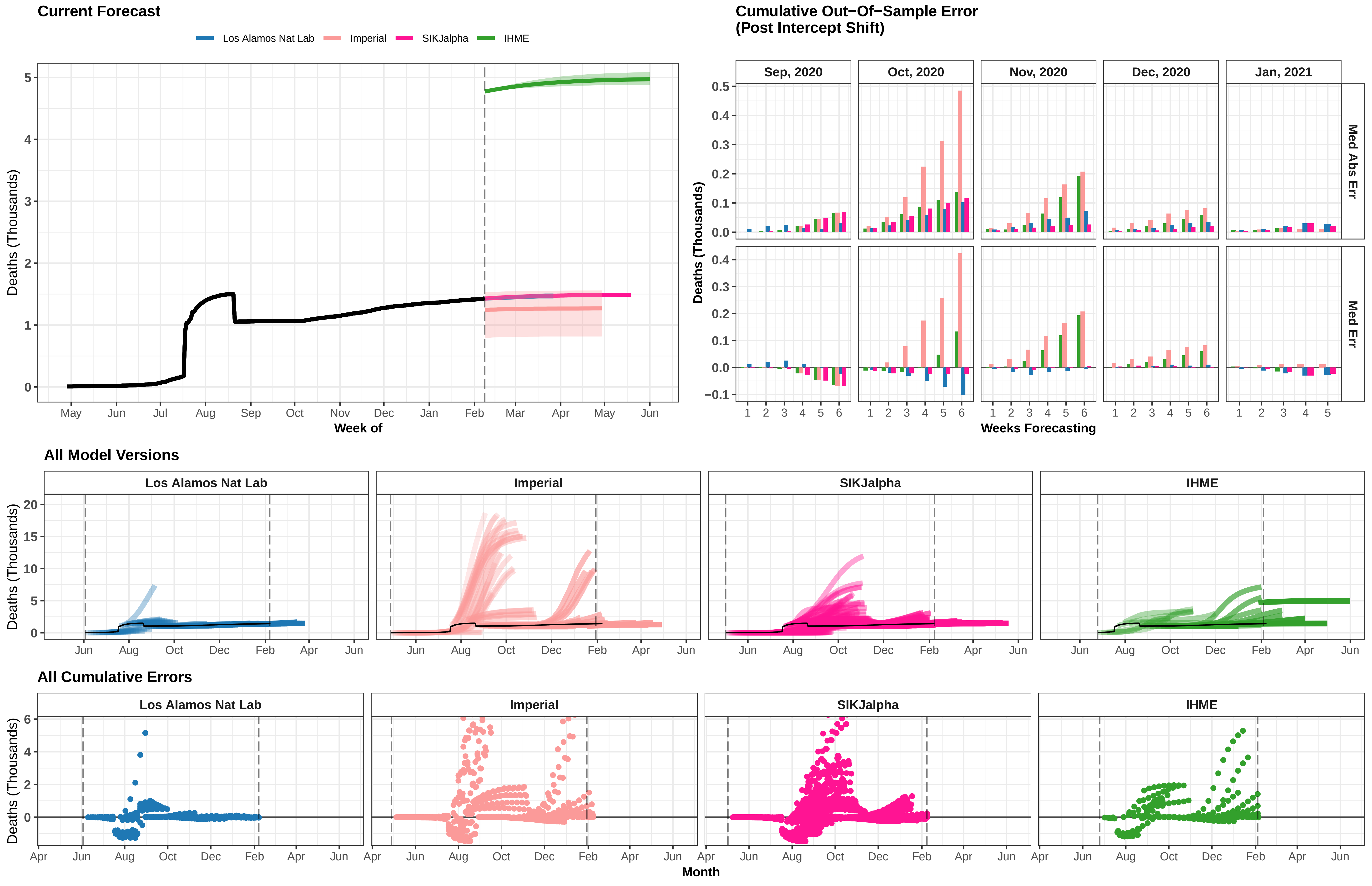
Current Forecast
Los Alamos Nat Lab
Imperial
SIKJalpha
IHME
Deaths (Thousands)
Week of
May
Jun
Jul
Aug
Sep
Oct
Nov
Dec
Jan
Feb
Mar
Apr
Cumulative Out−Of−Sample Error
(Post Intercept Shift)
Sep, 2020
Oct, 2020
Nov, 2020
Dec, 2020
Jan, 2021
Med Abs Err
Med Err
Weeks Forecasting
All Model Versions
All Cumulative Errors
Month

Latvia

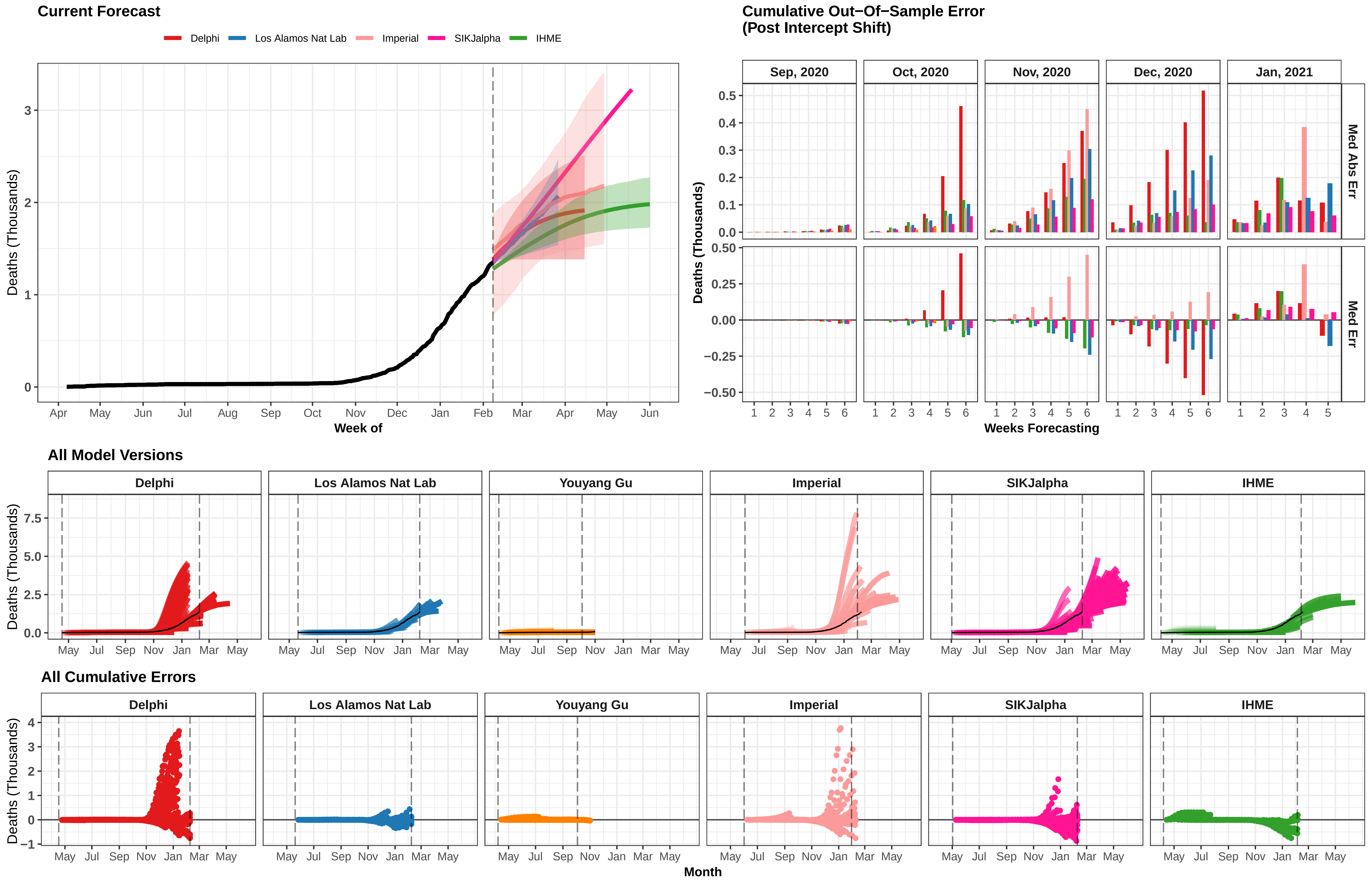
Current Forecast
Delphi
Los Alamos Nat Lab
Imperial
SIKJalpha
IHME
Deaths (Thousands)
Week of
Apr May Jun Jul Aug Sep Oct Nov Dec Jan Feb Mar Apr May Jun
Cumulative Out−Of−Sample Error
(Post Intercept Shift)
Sep, 2020
Oct, 2020
Nov, 2020
Dec, 2020
Jan, 2021
Med Abs Err
Med Err
Weeks Forecasting
All Model Versions
Delphi
Los Alamos Nat Lab
Youyang Gu
Imperial
SIKJalpha
IHME
All Cumulative Errors
Month

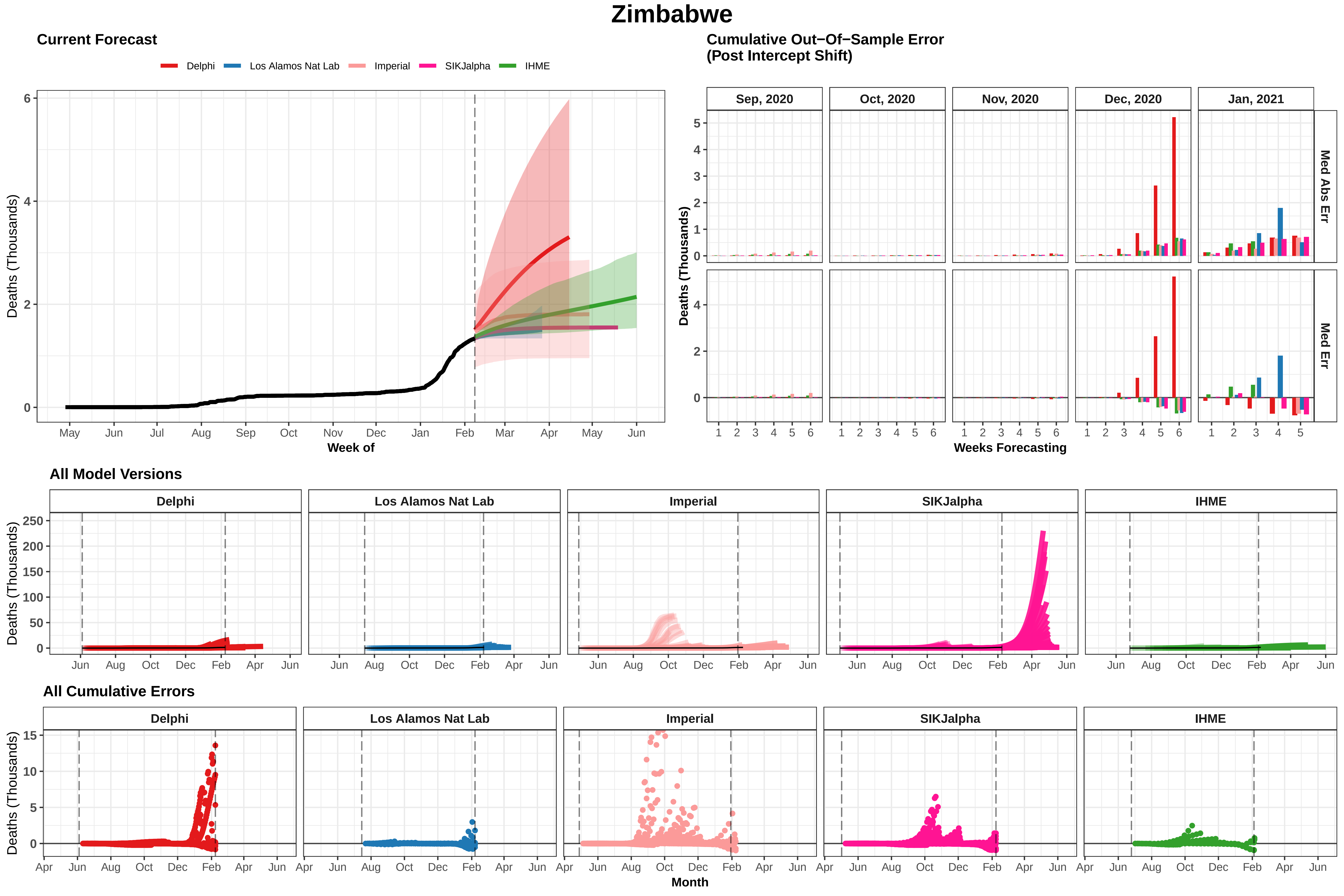
Zimbabwe
Current Forecast
Delphi
Los Alamos Nat Lab
Imperial
SIKJalpha
IHME
Deaths (Thousands)
Week of
May Jun Jul Aug Sep Oct Nov Dec Jan Feb Mar Apr May Jun
Cumulative Out−Of−Sample Error
(Post Intercept Shift)
Sep, 2020
Oct, 2020
Nov, 2020
Dec, 2020
Jan, 2021
Med Abs Err
Med Err
Weeks Forecasting
All Model Versions
All Cumulative Errors
Month

Montana

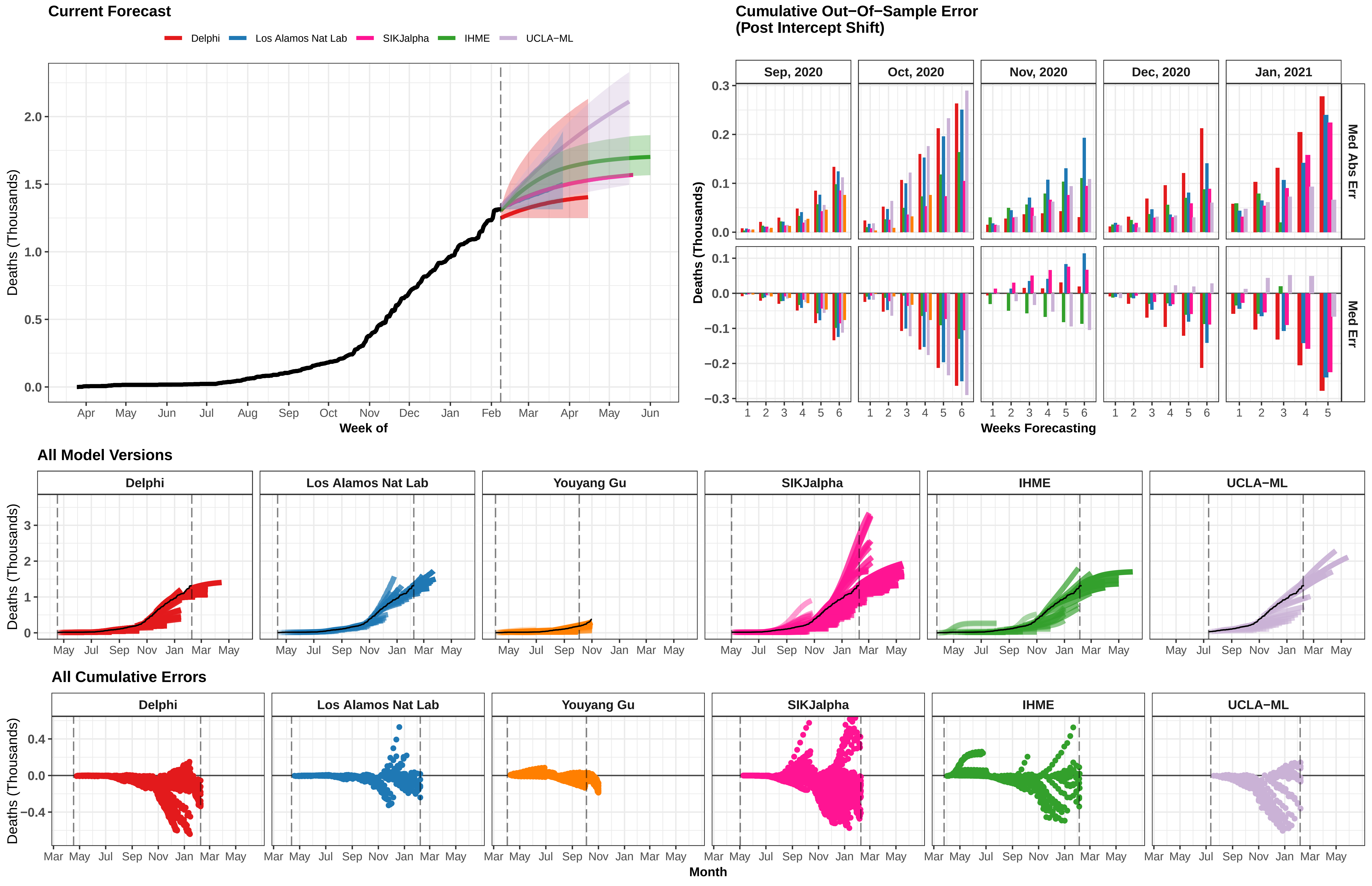

Current Forecast
Delphi
Los Alamos Nat Lab
SIKJalpha
IHME
UCLA−ML
Deaths (Thousands)
Week of
Cumulative Out−Of−Sample Error
(Post Intercept Shift)
Sep, 2020
Oct, 2020
Nov, 2020
Dec, 2020
Jan, 2021
Med Abs Err
Med Err
Weeks Forecasting
All Model Versions
Delphi
Los Alamos Nat Lab
Youyang Gu
SIKJalpha
IHME
UCLA−ML
All Cumulative Errors
Month

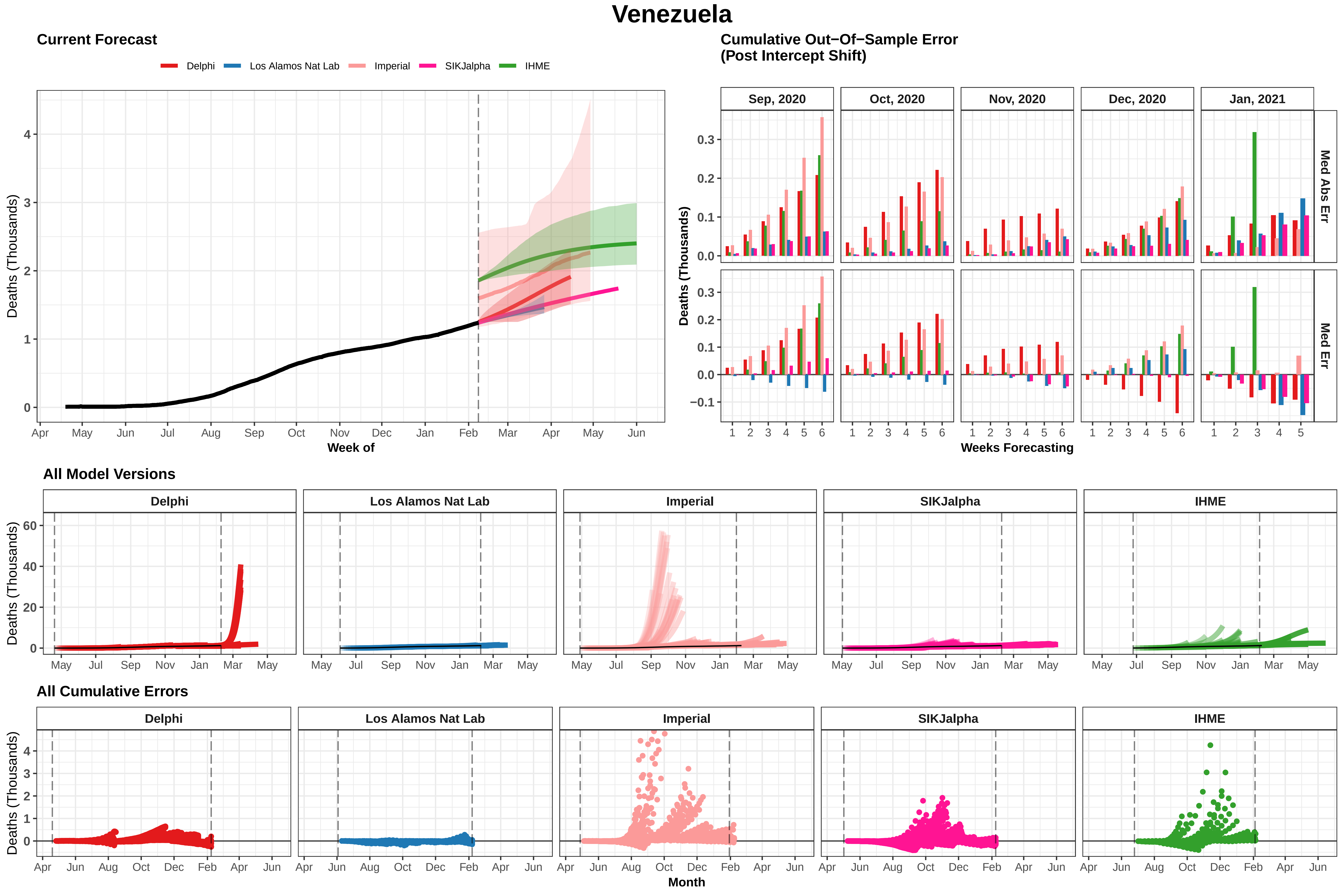
Venezuela
Current Forecast
Delphi
Los Alamos Nat Lab
Imperial
SIKJalpha
IHME
Deaths (Thousands)
Week of
Cumulative Out−Of−Sample Error
(Post Intercept Shift)
Sep, 2020
Oct, 2020
Nov, 2020
Dec, 2020
Jan, 2021
Med Abs Err
Med Err
Weeks Forecasting
All Model Versions
All Cumulative Errors
Month

Delaware

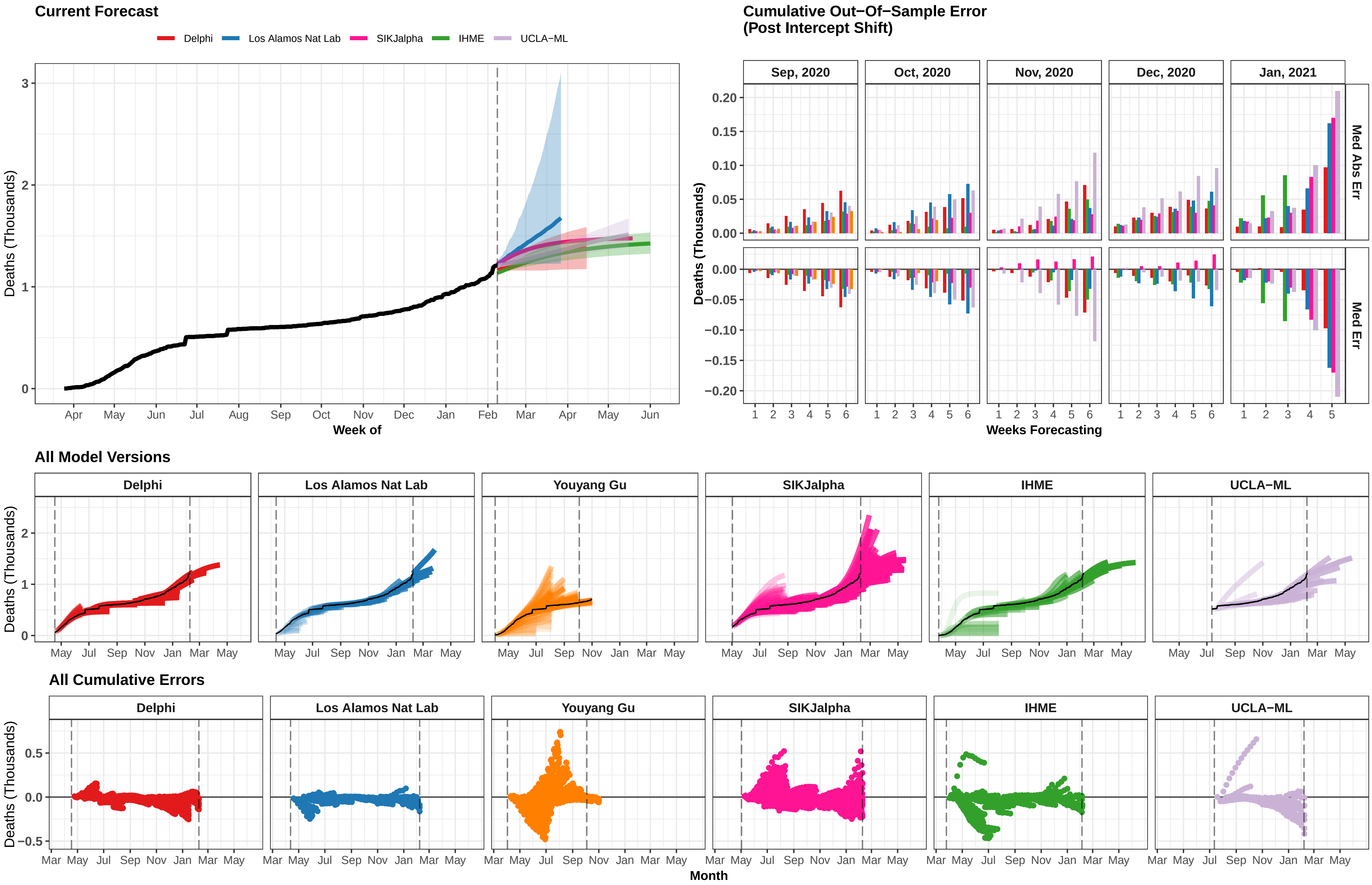

Current Forecast
Delphi
Los Alamos Nat Lab
SIKJalpha
IHME
UCLA−ML
Deaths (Thousands)
Week of
Apr May Jun Jul Aug Sep Oct Nov Dec Jan Feb Mar Apr May Jun
Cumulative Out−Of−Sample Error
(Post Intercept Shift)
Sep, 2020
Oct, 2020
Nov, 2020
Dec, 2020
Jan, 2021
Med Abs Err
Med Err
Weeks Forecasting
All Model Versions
Delphi
Los Alamos Nat Lab
Youyang Gu
SIKJalpha
IHME
UCLA−ML
All Cumulative Errors
Month

New Hampshire

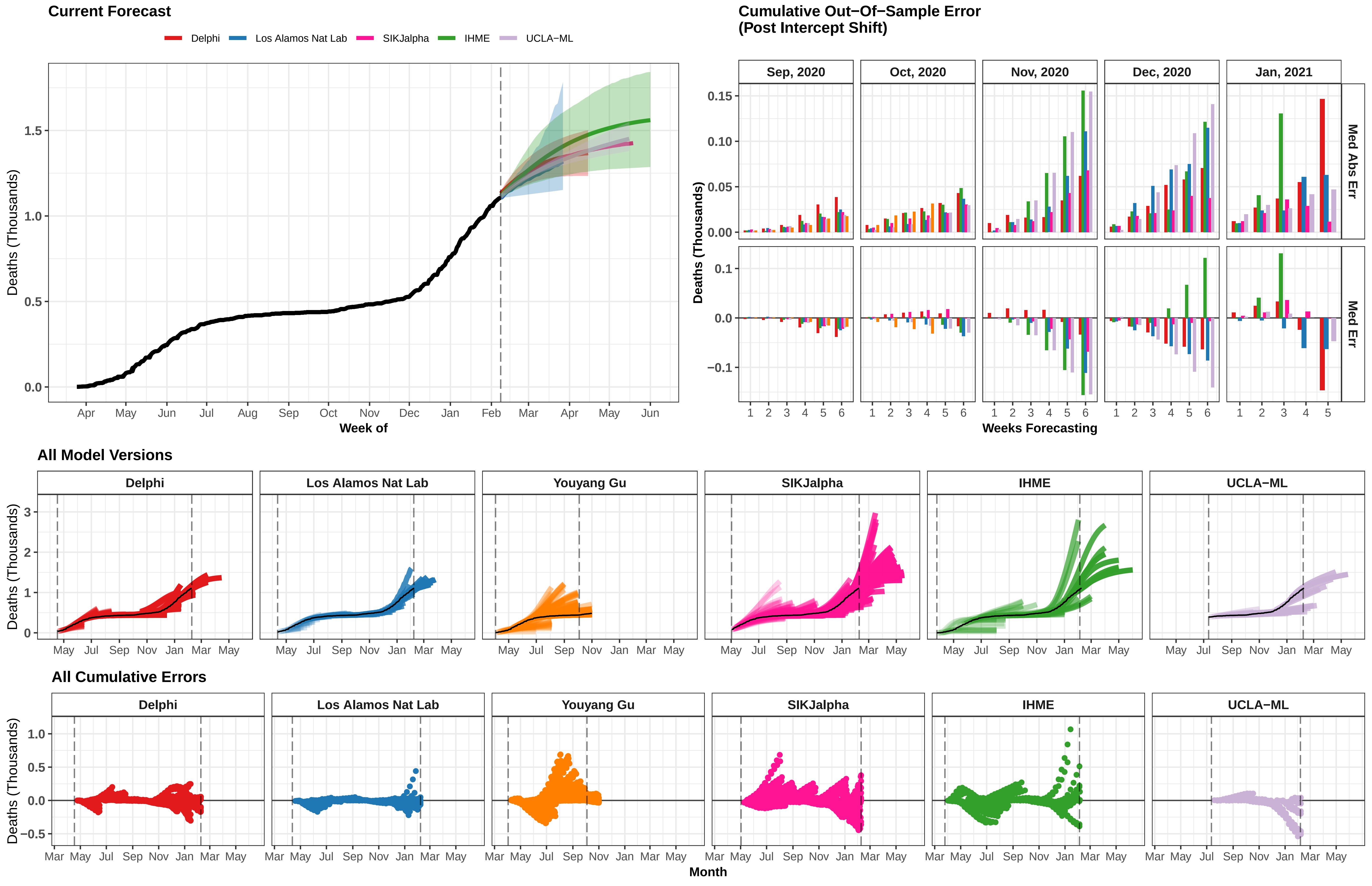

Kuwait

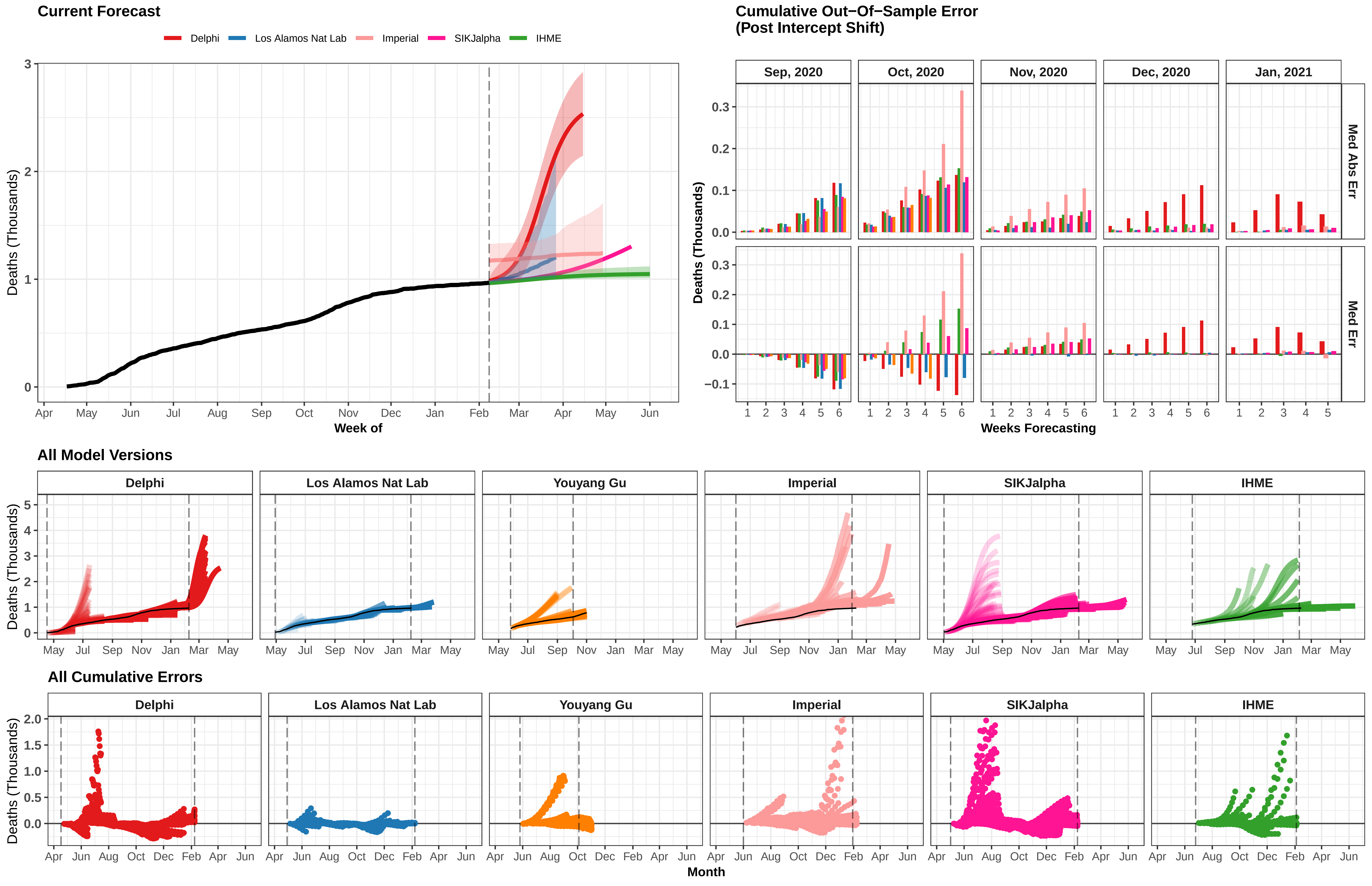

Current Forecast
Delphi
Los Alamos Nat Lab
Imperial
SIKJalpha
IHME
Deaths (Thousands)
0
1
2
3
Apr May Jun Jul Aug Sep Oct Nov Dec Jan Feb Mar Apr May Jun
Week of
Cumulative Out−Of−Sample Error
(Post Intercept Shift)
Sep, 2020
Oct, 2020
Nov, 2020
Dec, 2020
Jan, 2021
Med Abs Err
Med Err
Deaths (Thousands)
0.0
0.1
0.2
0.3
−0.1
1 2 3 4 5 6
1 2 3 4 5
Weeks Forecasting
All Model Versions
Delphi
Los Alamos Nat Lab
Youyang Gu
Imperial
SIKJalpha
IHME
Deaths (Thousands)
0 1 2 3 4 5
May Jul Sep Nov Jan Mar May
All Cumulative Errors
Deaths (Thousands)
0.0 0.5 1.0 1.5 2.0
Apr Jun Aug Oct Dec Feb Apr Jun
Month

Syria

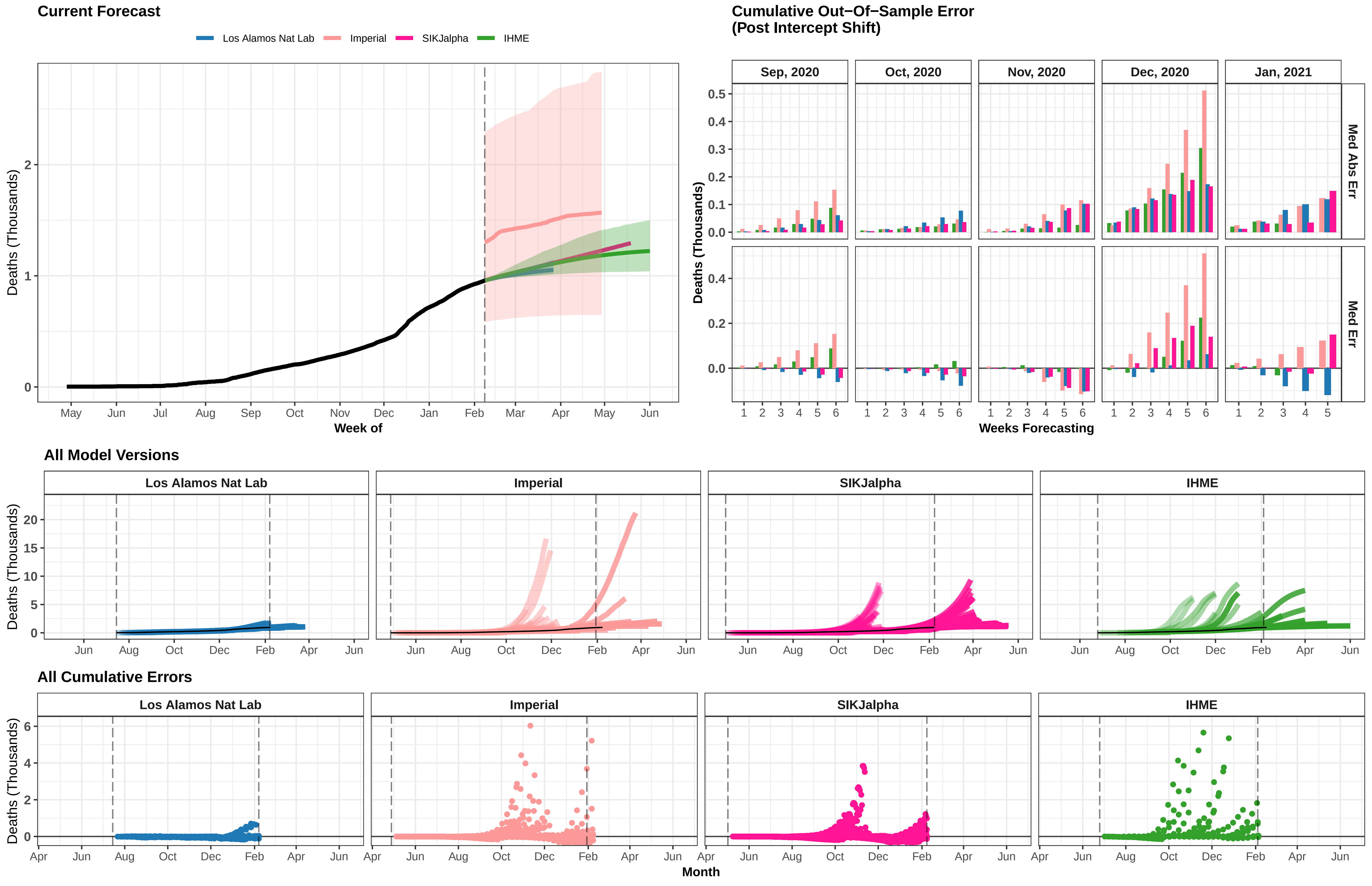
Current Forecast
Los Alamos Nat Lab
Imperial
SIKJalpha
IHME
Deaths (Thousands)
Week of
May
Jun
Jul
Aug
Sep
Oct
Nov
Dec
Jan
Feb
Mar
Apr
Cumulative Out−Of−Sample Error
(Post Intercept Shift)
Sep, 2020
Oct, 2020
Nov, 2020
Dec, 2020
Jan, 2021
Med Abs Err
Med Err
Weeks Forecasting
All Model Versions
All Cumulative Errors
Month

District of Columbia

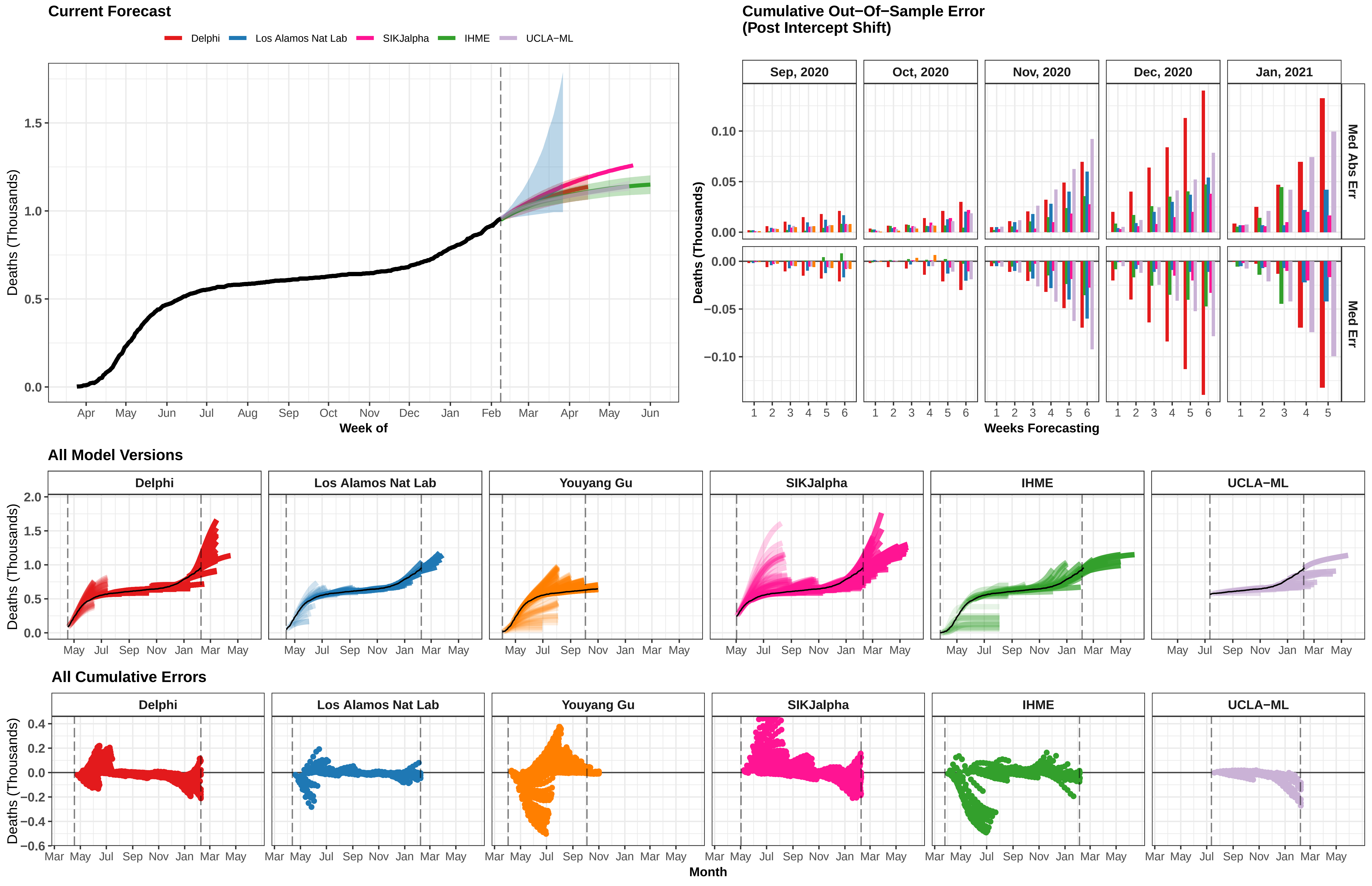

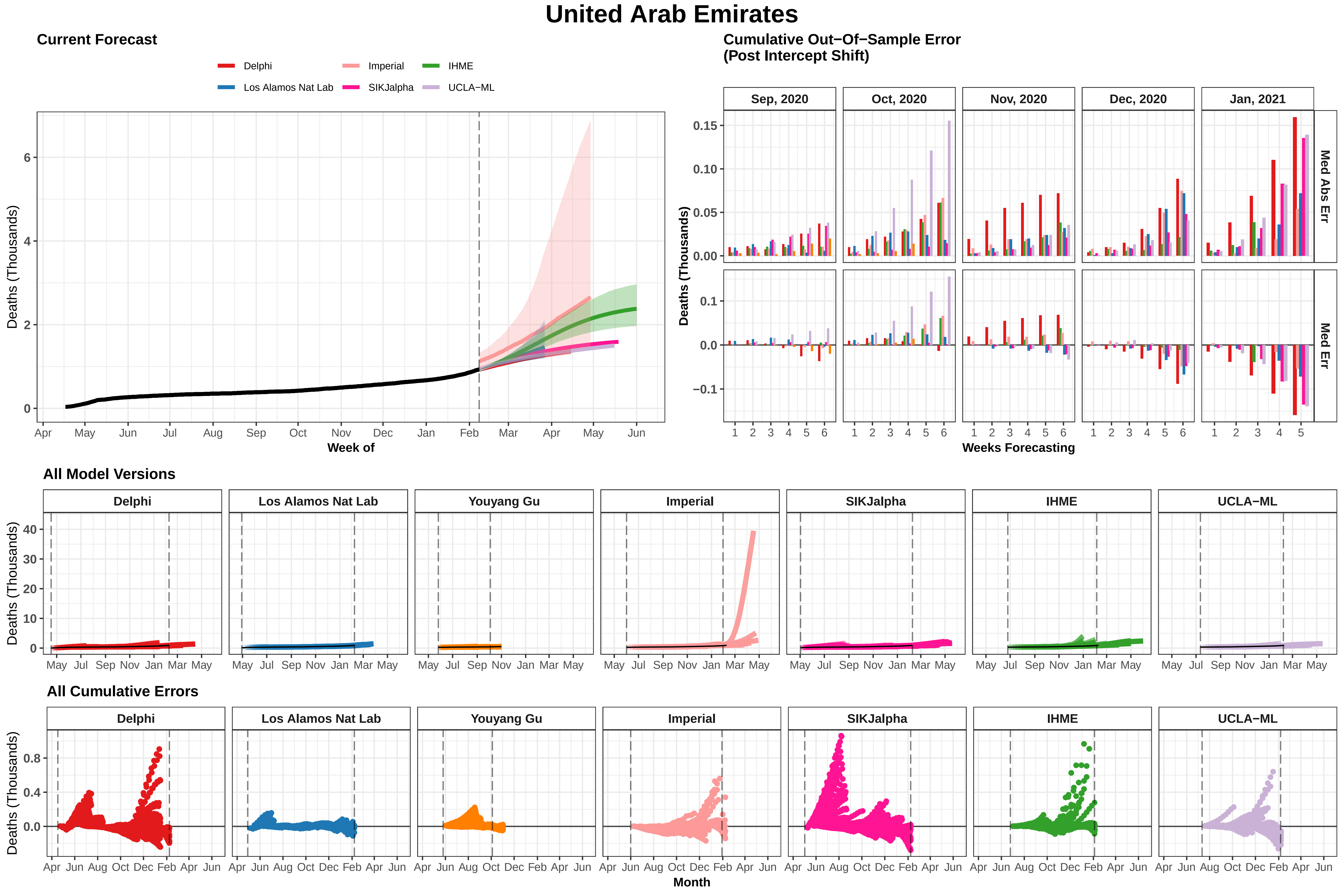
United Arab Emirates
Current Forecast
Delphi
Los Alamos Nat Lab
Imperial
SIKJalpha
IHME
UCLA−ML
Deaths (Thousands)
Week of
Cumulative Out−Of−Sample Error
(Post Intercept Shift)
Sep, 2020
Oct, 2020
Nov, 2020
Dec, 2020
Jan, 2021
Med Abs Err
Med Err
Weeks Forecasting
All Model Versions
Delphi
Los Alamos Nat Lab
Youyang Gu
Imperial
SIKJalpha
IHME
UCLA−ML
All Cumulative Errors
Month

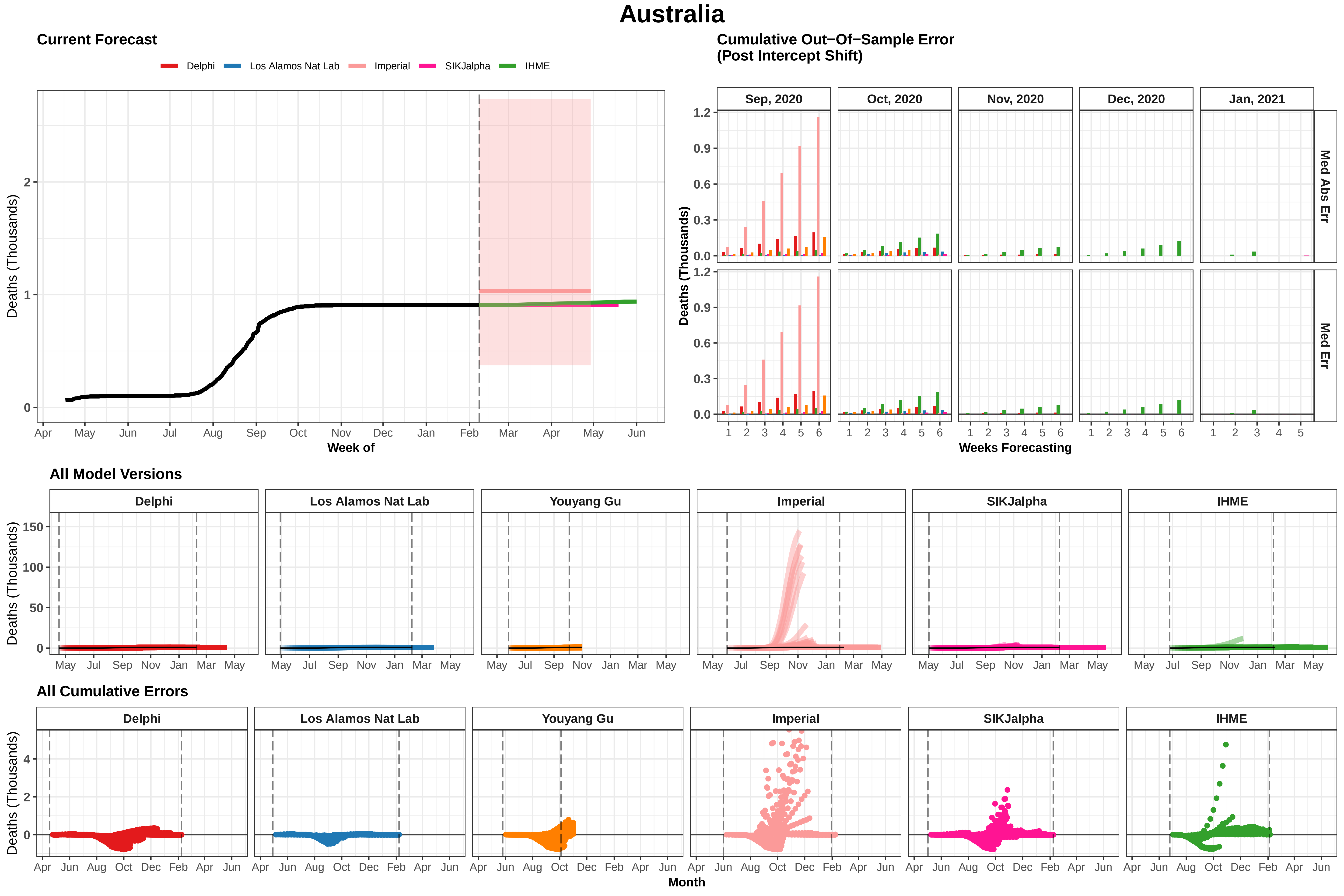

Australia
Current Forecast
Delphi
Los Alamos Nat Lab
Imperial
SIKJalpha
IHME
Deaths (Thousands)
Week of
Apr May Jun Jul Aug Sep Oct Nov Dec Jan Feb Mar Apr May Jun
Cumulative Out−Of−Sample Error
(Post Intercept Shift)
Sep, 2020
Oct, 2020
Nov, 2020
Dec, 2020
Jan, 2021
Med Abs Err
Med Err
Weeks Forecasting
All Model Versions
Delphi
Los Alamos Nat Lab
Youyang Gu
Imperial
SIKJalpha
IHME
All Cumulative Errors
Month

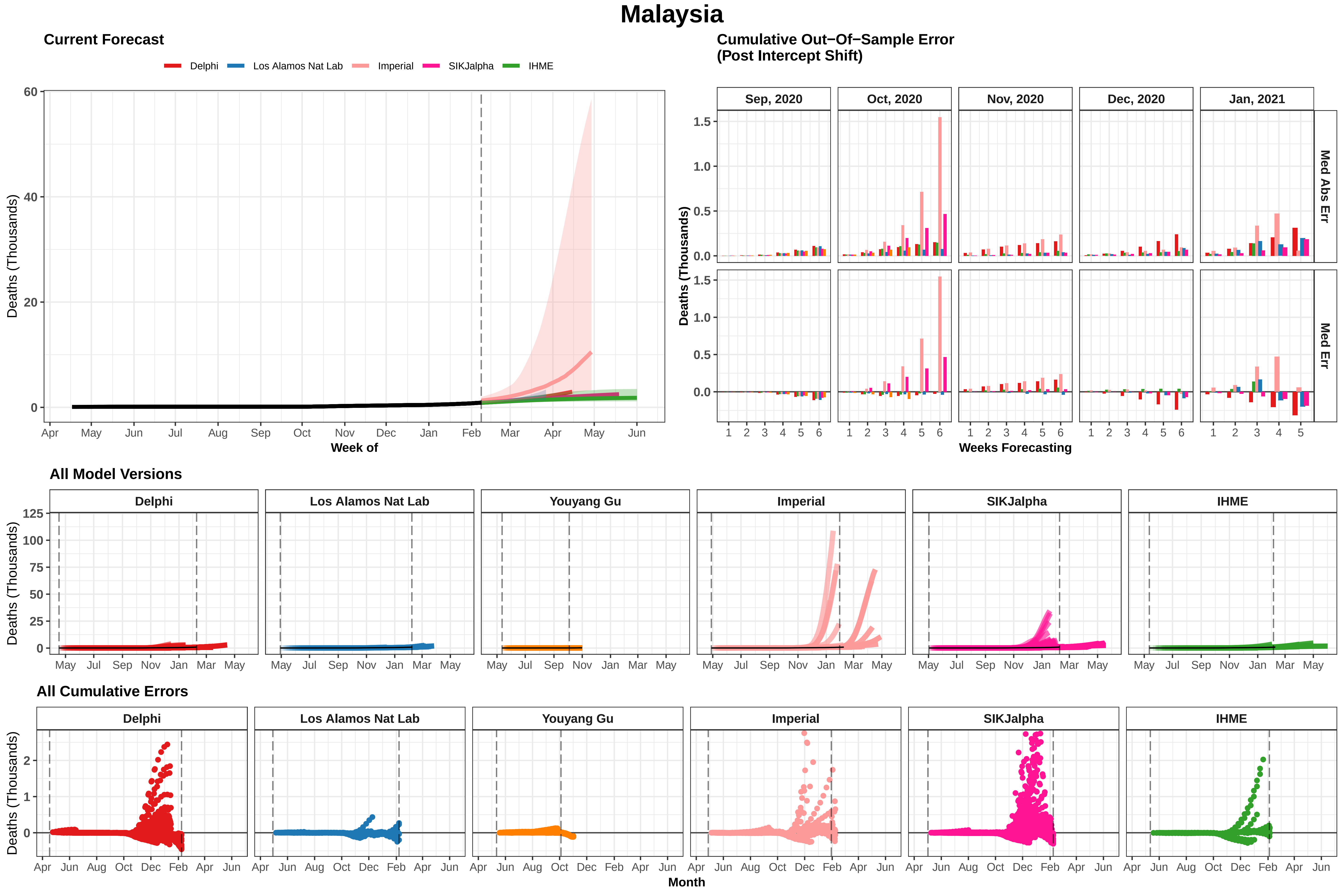
Malaysia
Current Forecast
Delphi
Los Alamos Nat Lab
Imperial
SIKJalpha
IHME
Deaths (Thousands)
Week of
Cumulative Out−Of−Sample Error
(Post Intercept Shift)
Sep, 2020
Oct, 2020
Nov, 2020
Dec, 2020
Jan, 2021
Med Abs Err
Med Err
Weeks Forecasting
All Model Versions
Youyang Gu
All Cumulative Errors
Month

Malawi

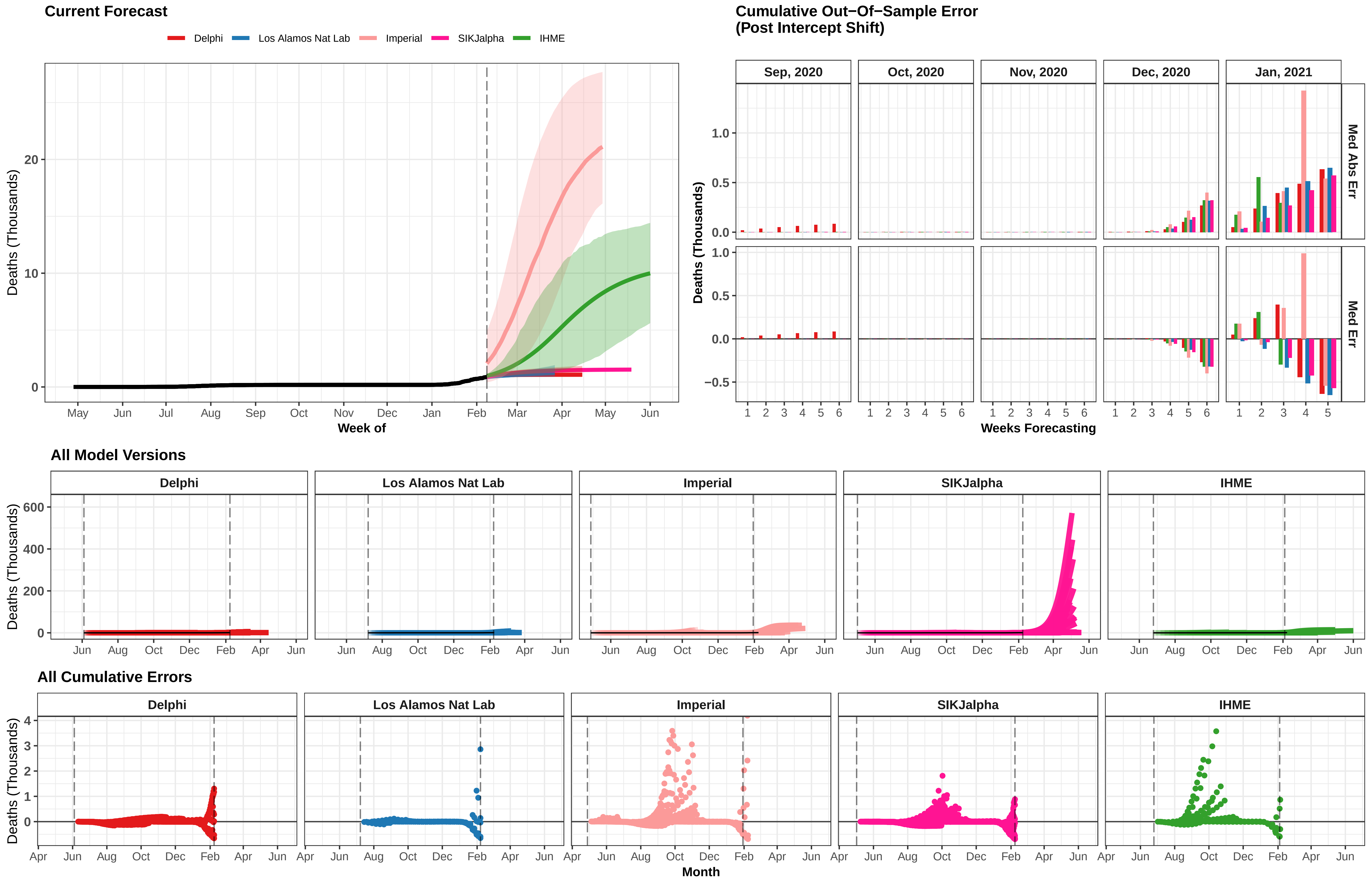
Current Forecast
Delphi
Los Alamos Nat Lab
Imperial
SIKJalpha
IHME
Deaths (Thousands)
0
10
20
May
Jun
Jul
Aug
Sep
Oct
Nov
Dec
Jan
Feb
Mar
Apr
May
Jun
Week of
Cumulative Out−Of−Sample Error
(Post Intercept Shift)
Sep, 2020
Oct, 2020
Nov, 2020
Dec, 2020
Jan, 2021
Med Abs Err
Med Err
Deaths (Thousands)
1.0
0.5
0.0
−0.5
1
2
3
4
5
6
Weeks Forecasting
All Model Versions
Delphi
Los Alamos Nat Lab
Imperial
SIKJalpha
IHME
Deaths (Thousands)
0
200
400
600
Jun
Aug
Oct
Dec
Feb
Apr
Jun
All Cumulative Errors
Delphi
Los Alamos Nat Lab
Imperial
SIKJalpha
IHME
Deaths (Thousands)
0
1
2
3
4
Apr
Jun
Aug
Oct
Dec
Feb
Apr
Jun
Month

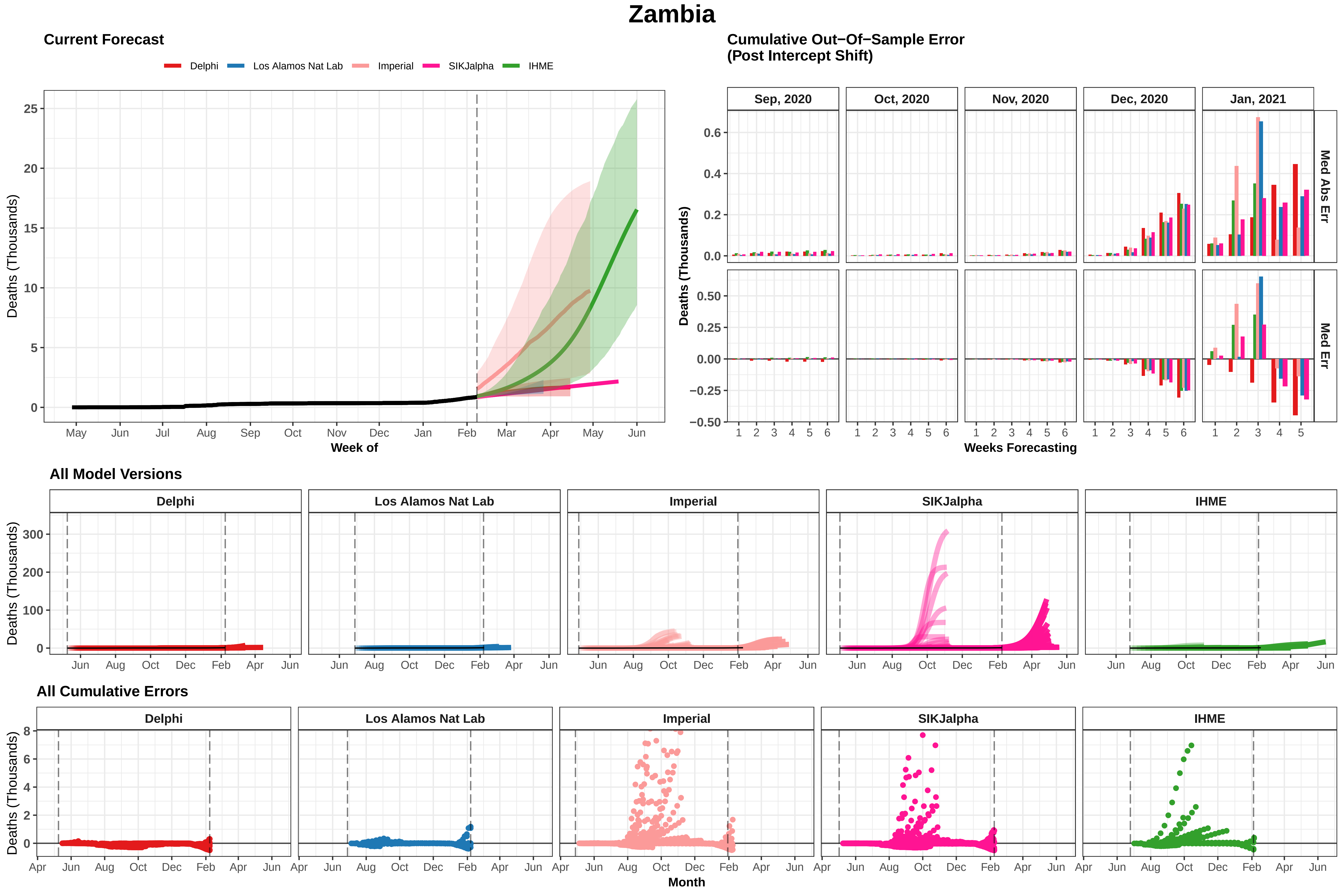
Zambia
Current Forecast
Delphi
Los Alamos Nat Lab
Imperial
SIKJalpha
IHME
Deaths (Thousands)
Week of
Cumulative Out−Of−Sample Error
(Post Intercept Shift)
Sep, 2020
Oct, 2020
Nov, 2020
Dec, 2020
Jan, 2021
Med Abs Err
Med Err
Weeks Forecasting
All Model Versions
All Cumulative Errors
Month

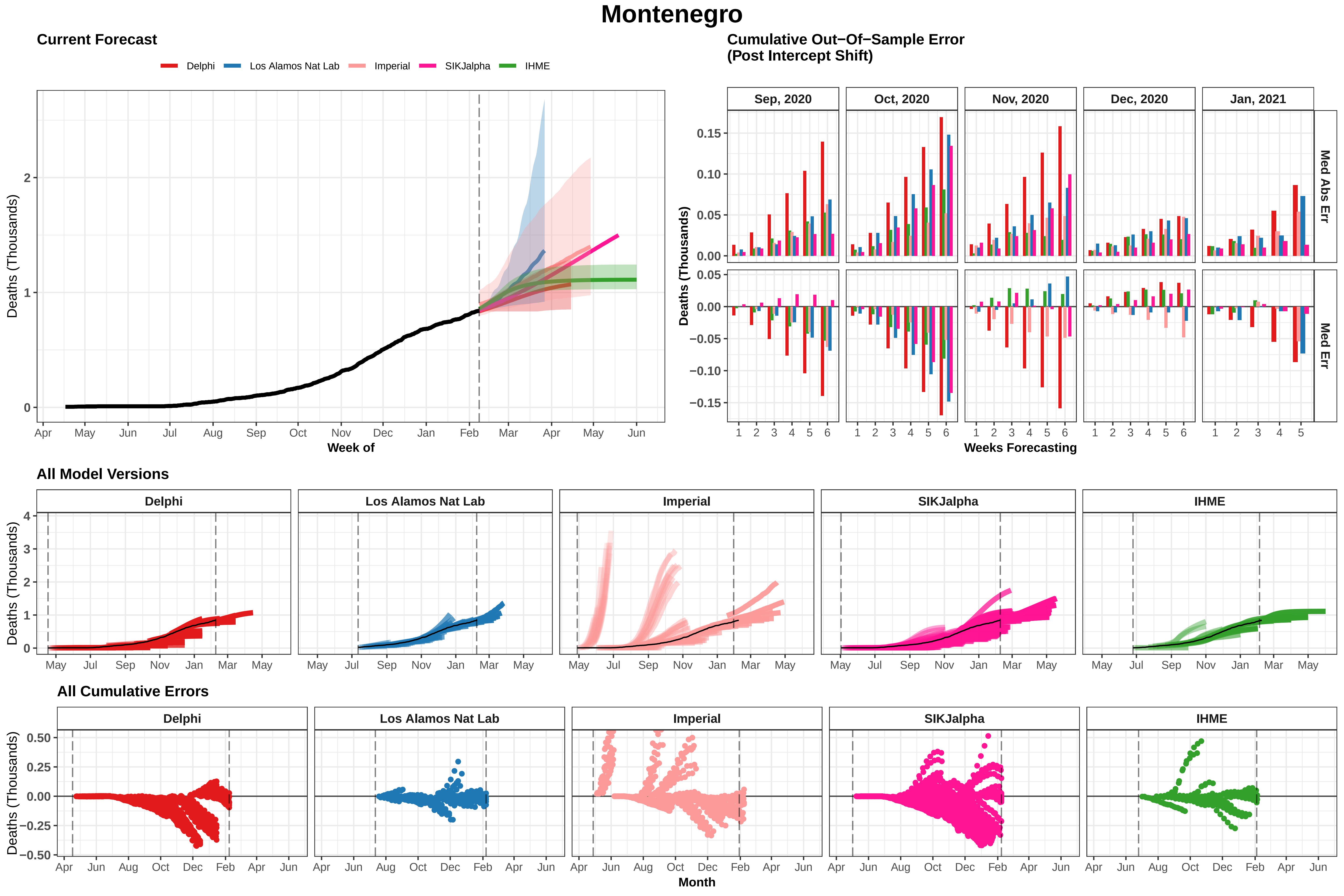

Montenegro
Current Forecast
Delphi
Los Alamos Nat Lab
Imperial
SIKJalpha
IHME
Deaths (Thousands)
Week of
Cumulative Out−Of−Sample Error
(Post Intercept Shift)
Sep, 2020
Oct, 2020
Nov, 2020
Dec, 2020
Jan, 2021
Med Abs Err
Med Err
Weeks Forecasting
All Model Versions
All Cumulative Errors
Month

Senegal

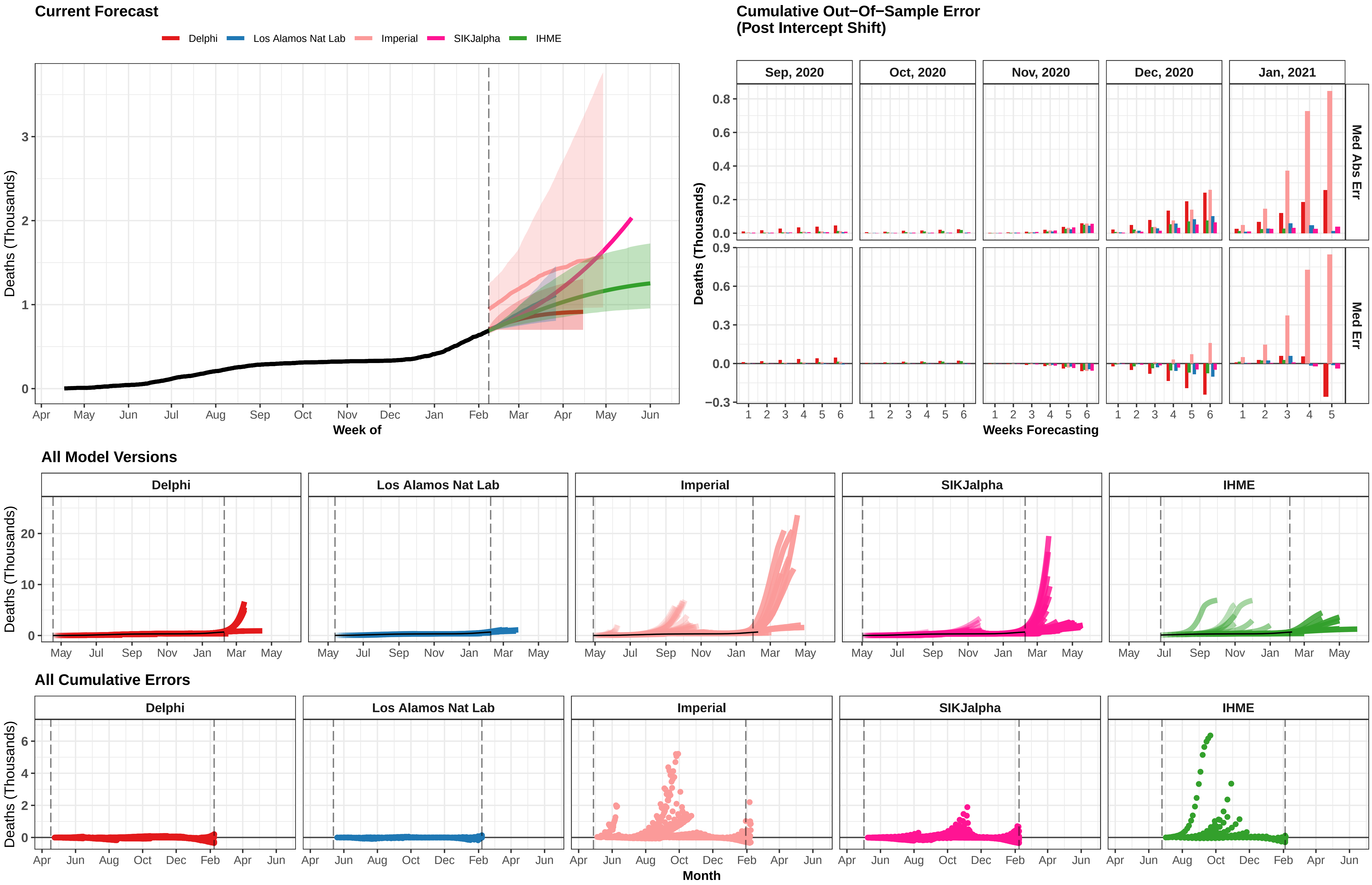
Current Forecast
Delphi
Los Alamos Nat Lab
Imperial
SIKJalpha
IHME
Deaths (Thousands)
Week of
Apr
May
Jun
Jul
Aug
Sep
Oct
Nov
Dec
Jan
Feb
Mar
Cumulative Out−Of−Sample Error
(Post Intercept Shift)
Sep, 2020
Oct, 2020
Nov, 2020
Dec, 2020
Jan, 2021
Med Abs Err
Med Err
Weeks Forecasting
All Model Versions
All Cumulative Errors
Month

Finland

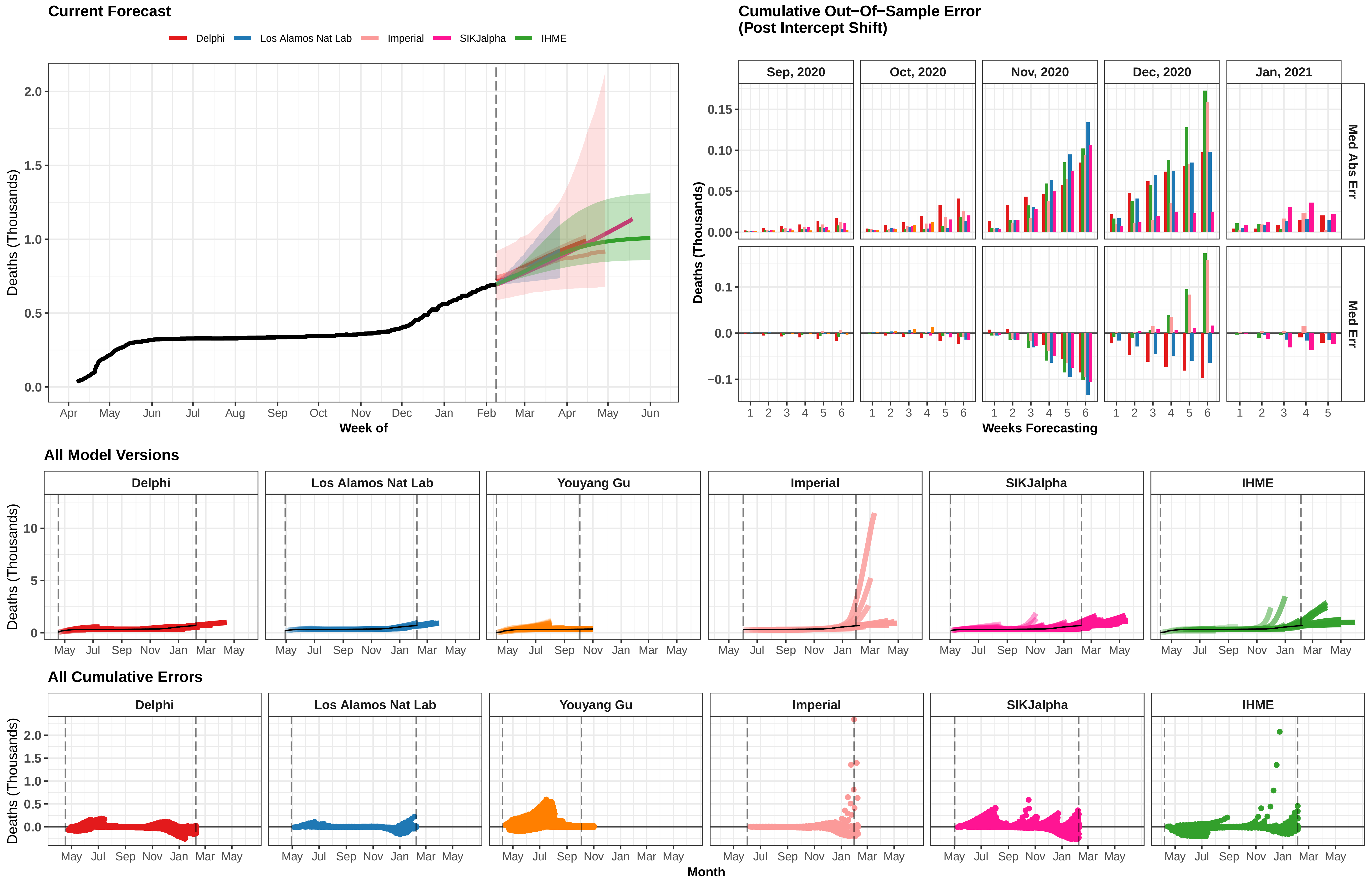

Current Forecast
Delphi
Los Alamos Nat Lab
Imperial
SIKJalpha
IHME
Deaths (Thousands)
Week of
Apr May Jun Jul Aug Sep Oct Nov Dec Jan Feb Mar Apr May Jun
Cumulative Out−Of−Sample Error
(Post Intercept Shift)
Sep, 2020
Oct, 2020
Nov, 2020
Dec, 2020
Jan, 2021
Med Abs Err
Med Err
Weeks Forecasting
All Model Versions
Delphi
Los Alamos Nat Lab
Youyang Gu
Imperial
SIKJalpha
IHME
All Cumulative Errors
Month

Democratic Republic of the Congo

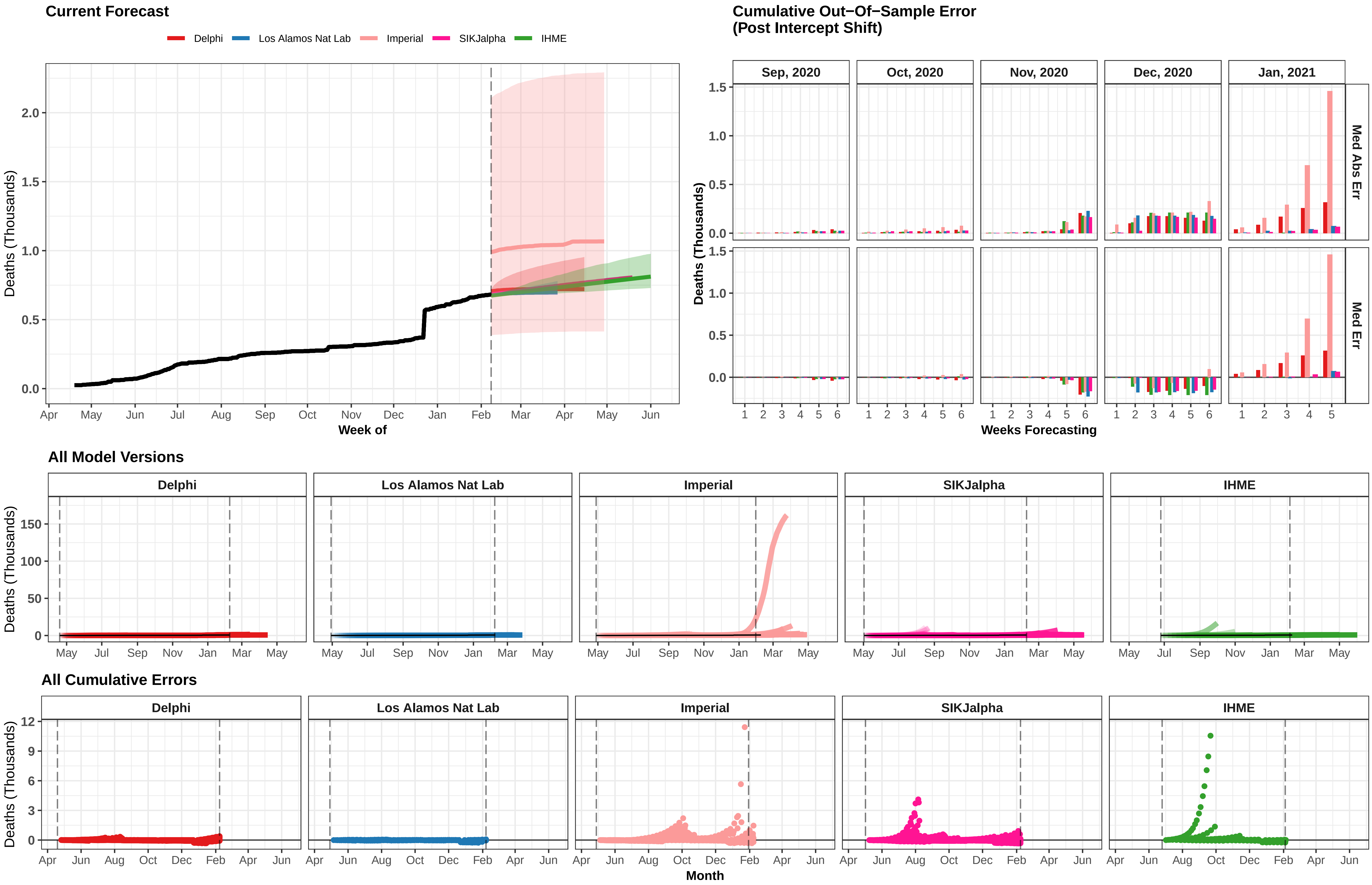

Maine

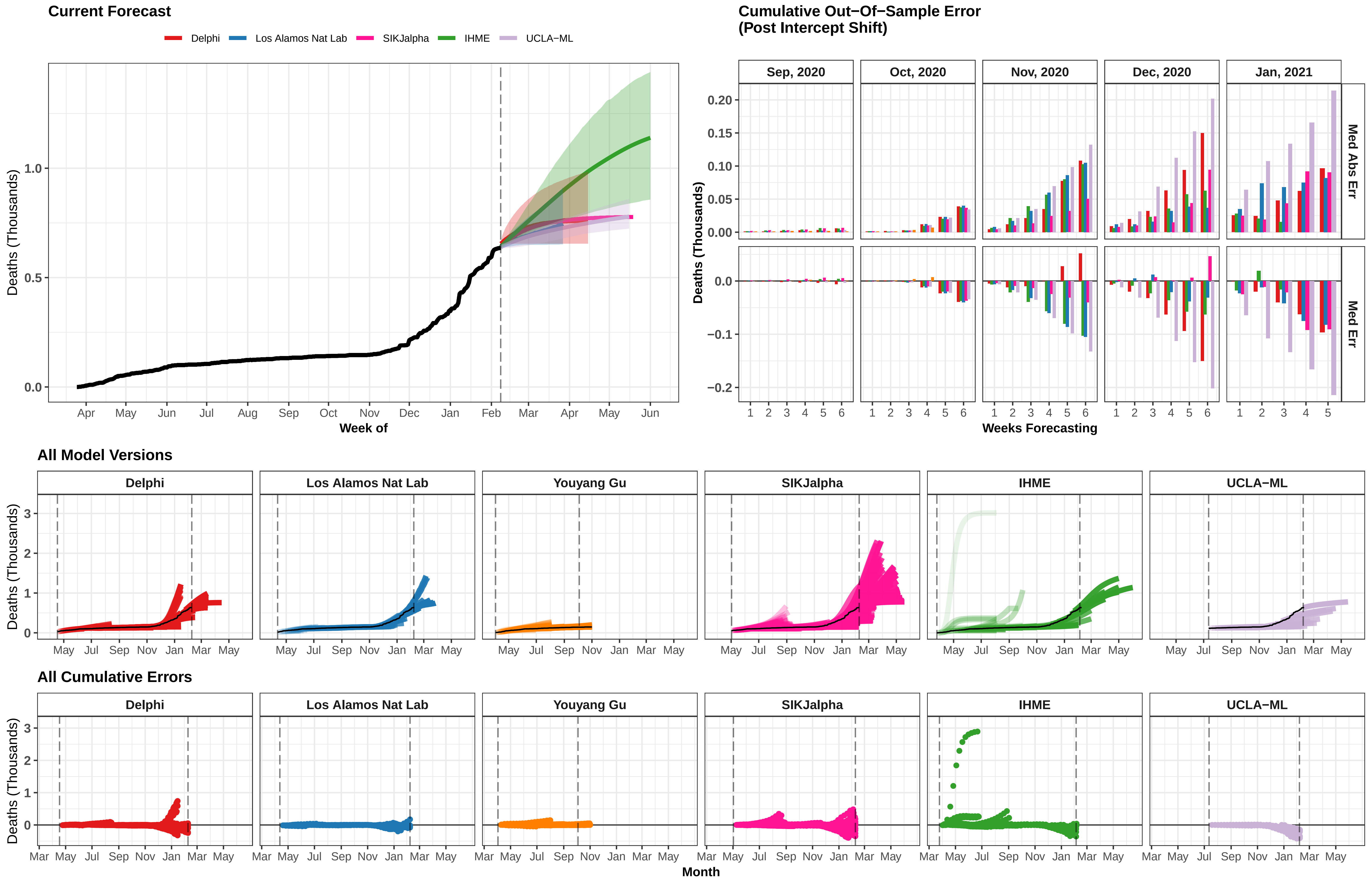
Current Forecast
Delphi
Los Alamos Nat Lab
SIKJalpha
IHME
UCLA−ML
Deaths (Thousands)
Week of
Apr May Jun Jul Aug Sep Oct Nov Dec Jan Feb Mar Apr May Jun
Cumulative Out−Of−Sample Error
(Post Intercept Shift)
Sep, 2020
Oct, 2020
Nov, 2020
Dec, 2020
Jan, 2021
Med Abs Err
Med Err
Weeks Forecasting
All Model Versions
Delphi
Los Alamos Nat Lab
Youyang Gu
SIKJalpha
IHME
UCLA−ML
All Cumulative Errors
Month

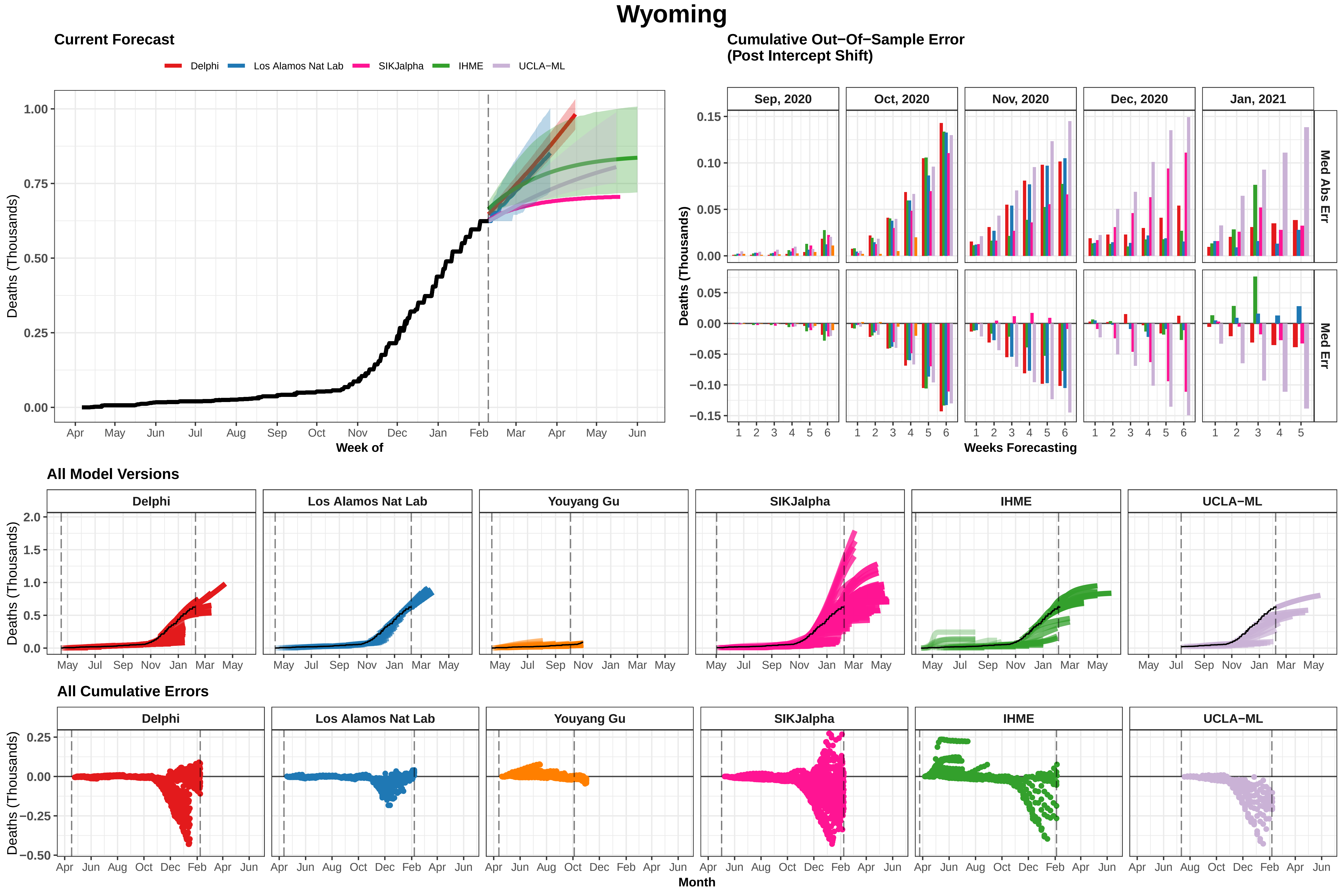
Wyoming
Current Forecast
Delphi
Los Alamos Nat Lab
SIKJalpha
IHME
UCLA−ML
Deaths (Thousands)
Week of
Cumulative Out−Of−Sample Error
(Post Intercept Shift)
Sep, 2020
Oct, 2020
Nov, 2020
Dec, 2020
Jan, 2021
Med Abs Err
Med Err
Weeks Forecasting
All Model Versions
Delphi
Los Alamos Nat Lab
Youyang Gu
SIKJalpha
IHME
UCLA−ML
All Cumulative Errors
Month

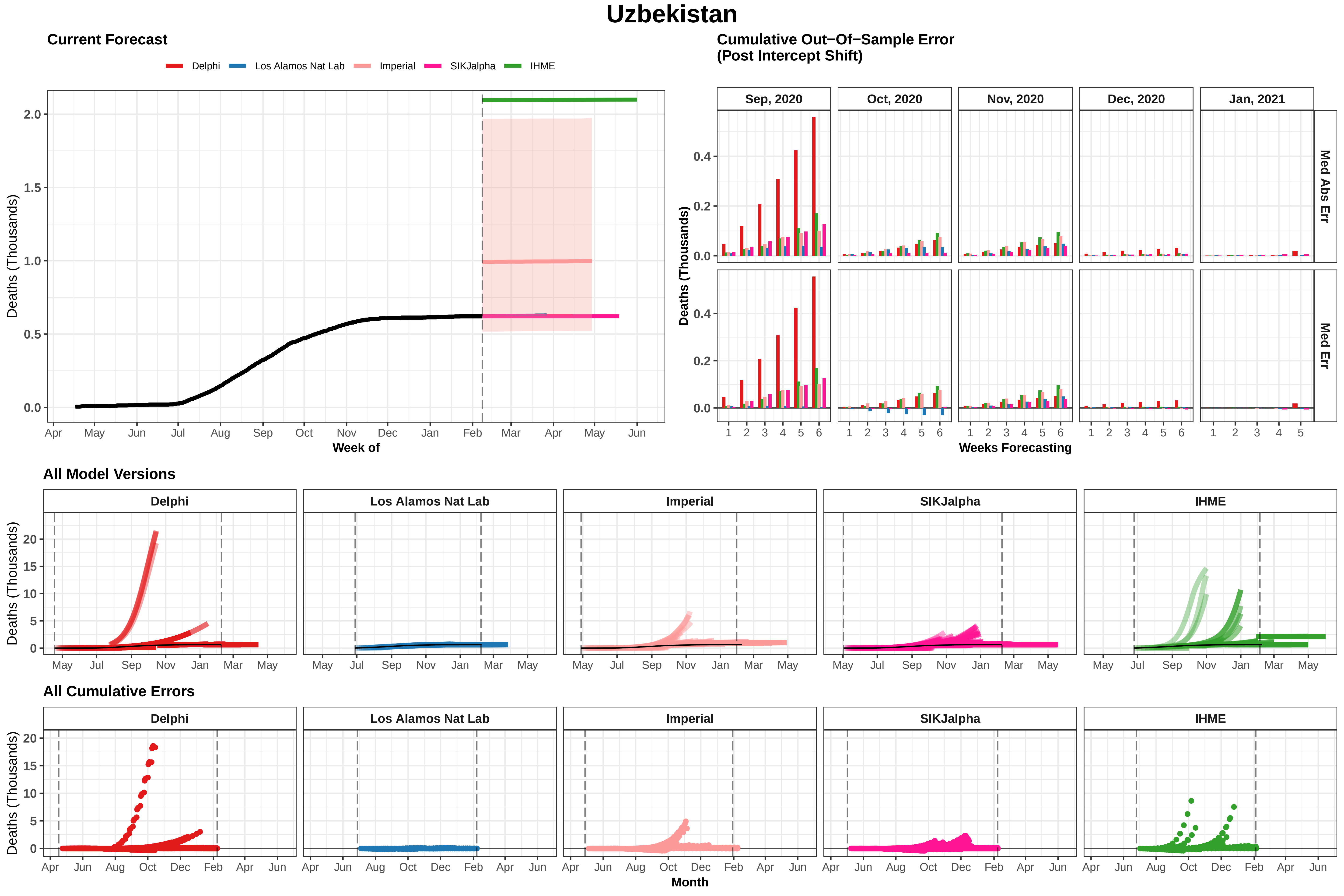
Uzbekistan
Current Forecast
Delphi
Los Alamos Nat Lab
Imperial
SIKJalpha
IHME
Deaths (Thousands)
0.0
0.5
1.0
1.5
2.0
Apr
May
Jun
Jul
Aug
Sep
Oct
Nov
Dec
Jan
Feb
Mar
Apr
May
Jun
Week of
Cumulative Out−Of−Sample Error
(Post Intercept Shift)
Sep, 2020
Oct, 2020
Nov, 2020
Dec, 2020
Jan, 2021
Med Abs Err
Med Err
0.0
0.2
0.4
1
2
3
4
5
6
Weeks Forecasting
All Model Versions
Delphi
Los Alamos Nat Lab
Imperial
SIKJalpha
IHME
0
5
10
15
20
May
Jul
Sep
Nov
Jan
Mar
May
All Cumulative Errors
Apr
Jun
Aug
Oct
Dec
Feb
Month

Yemen

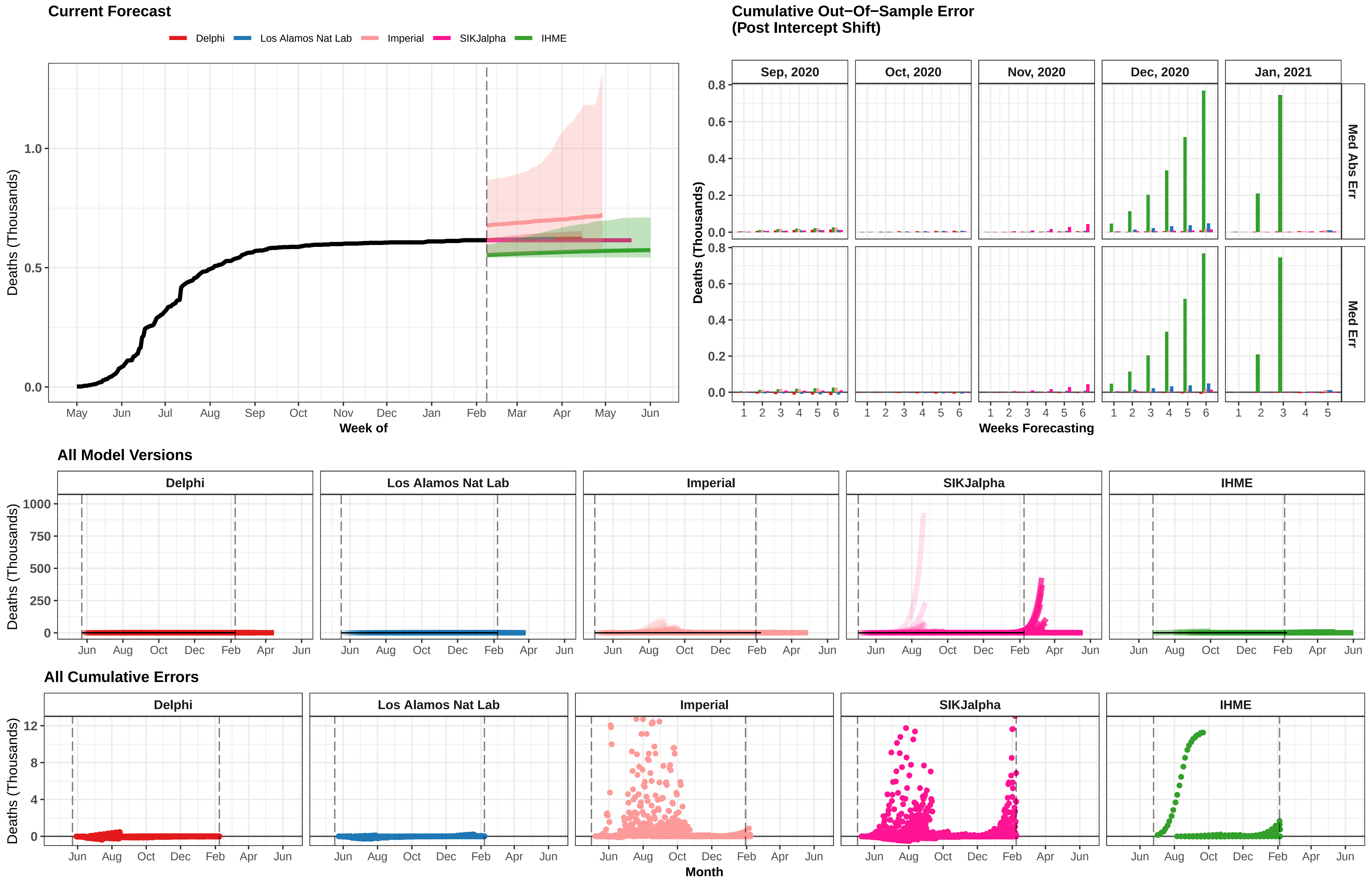

Current Forecast
Delphi
Los Alamos Nat Lab
Imperial
SIKJalpha
IHME
Deaths (Thousands)
Week of
Cumulative Out−Of−Sample Error
(Post Intercept Shift)
Sep, 2020
Oct, 2020
Nov, 2020
Dec, 2020
Jan, 2021
Med Abs Err
Med Err
Weeks Forecasting
All Model Versions
All Cumulative Errors
Month

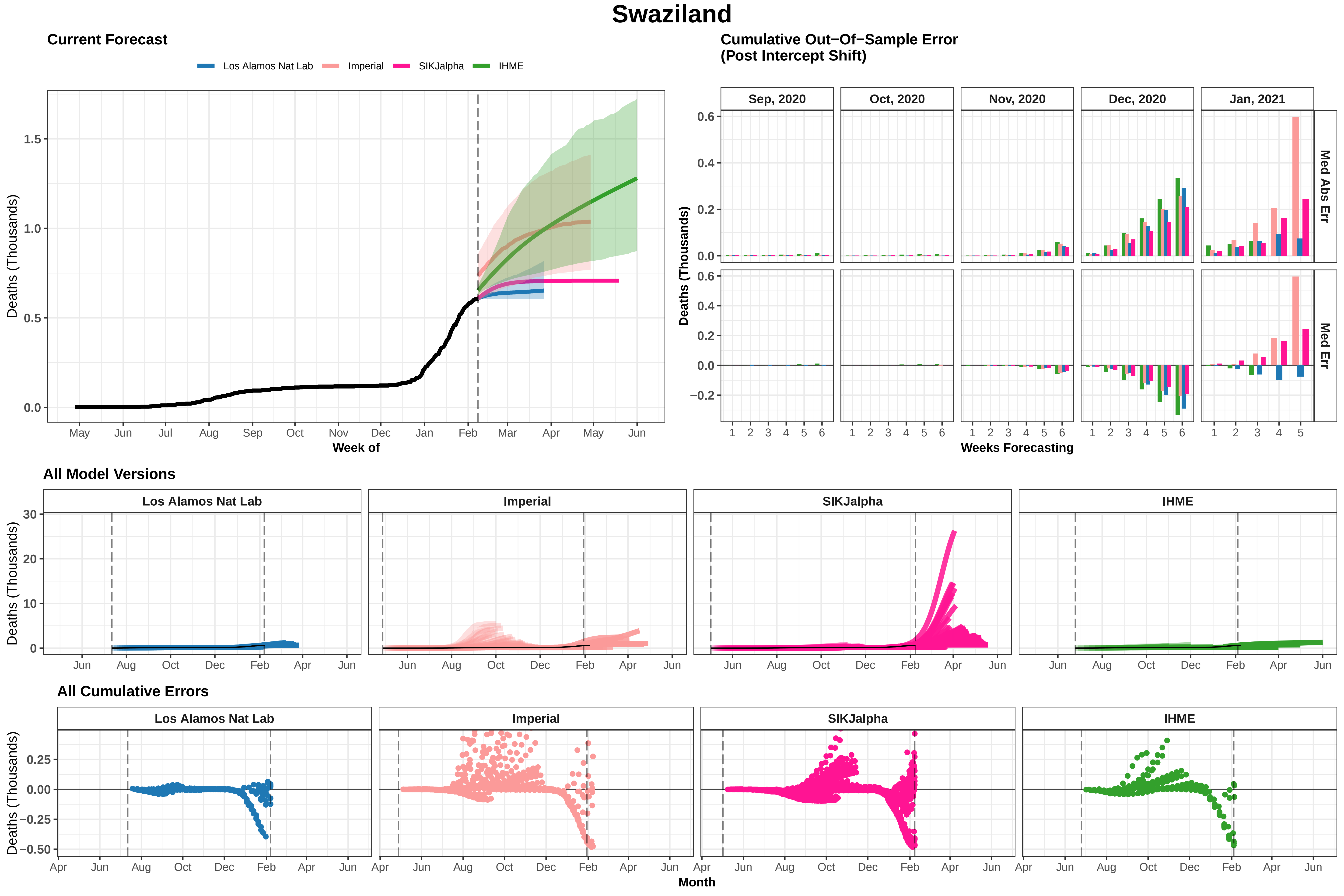

Swaziland
Current Forecast
Los Alamos Nat Lab
Imperial
SIKJalpha
IHME
Deaths (Thousands)
Week of
May
Jun
Jul
Aug
Sep
Oct
Nov
Dec
Jan
Feb
Mar
Apr
Cumulative Out−Of−Sample Error
(Post Intercept Shift)
Sep, 2020
Oct, 2020
Nov, 2020
Dec, 2020
Jan, 2021
Med Abs Err
Med Err
Weeks Forecasting
All Model Versions
All Cumulative Errors
Month

Luxembourg

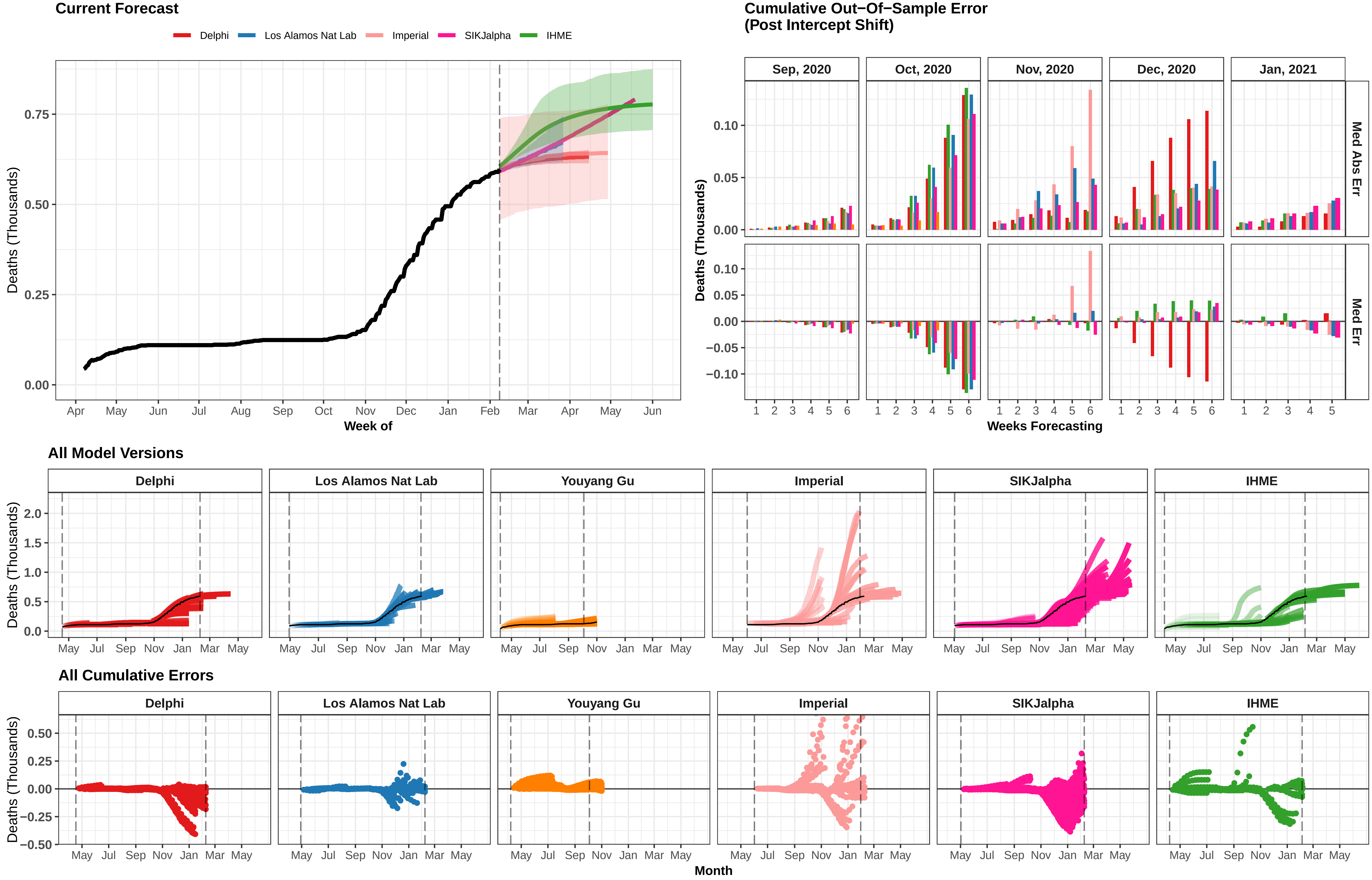

Current Forecast
Delphi
Los Alamos Nat Lab
Imperial
SIKJalpha
IHME
Deaths (Thousands)
0.00
0.25
0.50
0.75
Apr
May
Jun
Jul
Aug
Sep
Oct
Nov
Dec
Jan
Feb
Mar
Apr
May
Jun
Week of
Cumulative Out−Of−Sample Error
(Post Intercept Shift)
Sep, 2020
Oct, 2020
Nov, 2020
Dec, 2020
Jan, 2021
Med Abs Err
Med Err
Deaths (Thousands)
0.00
0.05
0.10
−0.05
−0.10
Weeks Forecasting
All Model Versions
Delphi
Los Alamos Nat Lab
Youyang Gu
Imperial
SIKJalpha
IHME
Deaths (Thousands)
0.0
0.5
1.0
1.5
2.0
May
Jul
Sep
Nov
Jan
Mar
May
All Cumulative Errors
Deaths (Thousands)
−0.50
−0.25
0.00
0.25
0.50
Month

Norway

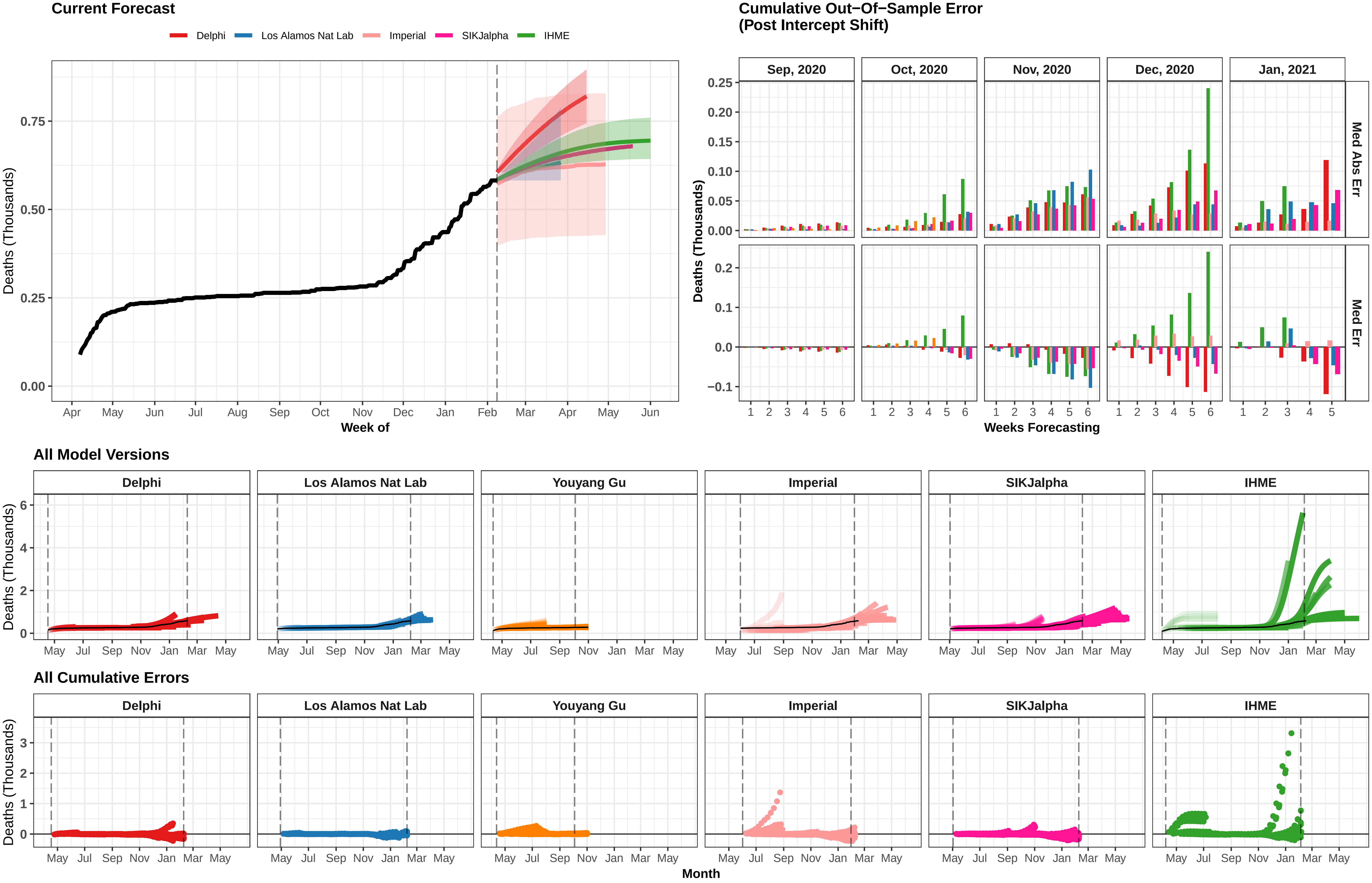
Current Forecast
Delphi
Los Alamos Nat Lab
Imperial
SIKJalpha
IHME
Deaths (Thousands)
Week of
Apr May Jun Jul Aug Sep Oct Nov Dec Jan Feb Mar Apr May Jun
Cumulative Out−Of−Sample Error
(Post Intercept Shift)
Sep, 2020
Oct, 2020
Nov, 2020
Dec, 2020
Jan, 2021
Med Abs Err
Med Err
Weeks Forecasting
All Model Versions
Delphi
Los Alamos Nat Lab
Youyang Gu
Imperial
SIKJalpha
IHME
All Cumulative Errors
Month

Uruguay

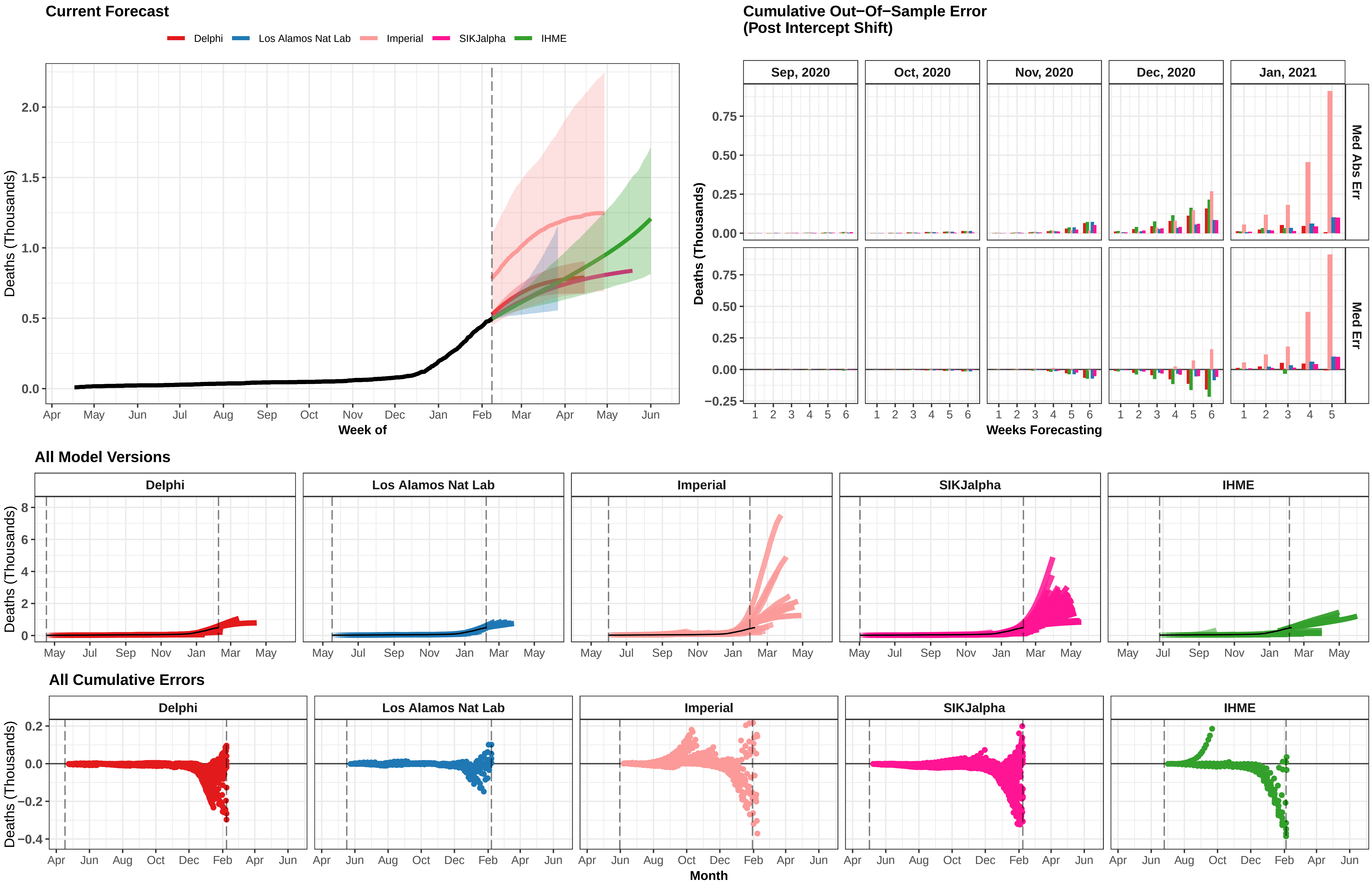

Current Forecast
Delphi
Los Alamos Nat Lab
Imperial
SIKJalpha
IHME
Deaths (Thousands)
Week of
Cumulative Out−Of−Sample Error
(Post Intercept Shift)
Sep, 2020
Oct, 2020
Nov, 2020
Dec, 2020
Jan, 2021
Med Abs Err
Med Err
Weeks Forecasting
All Model Versions
All Cumulative Errors
Month

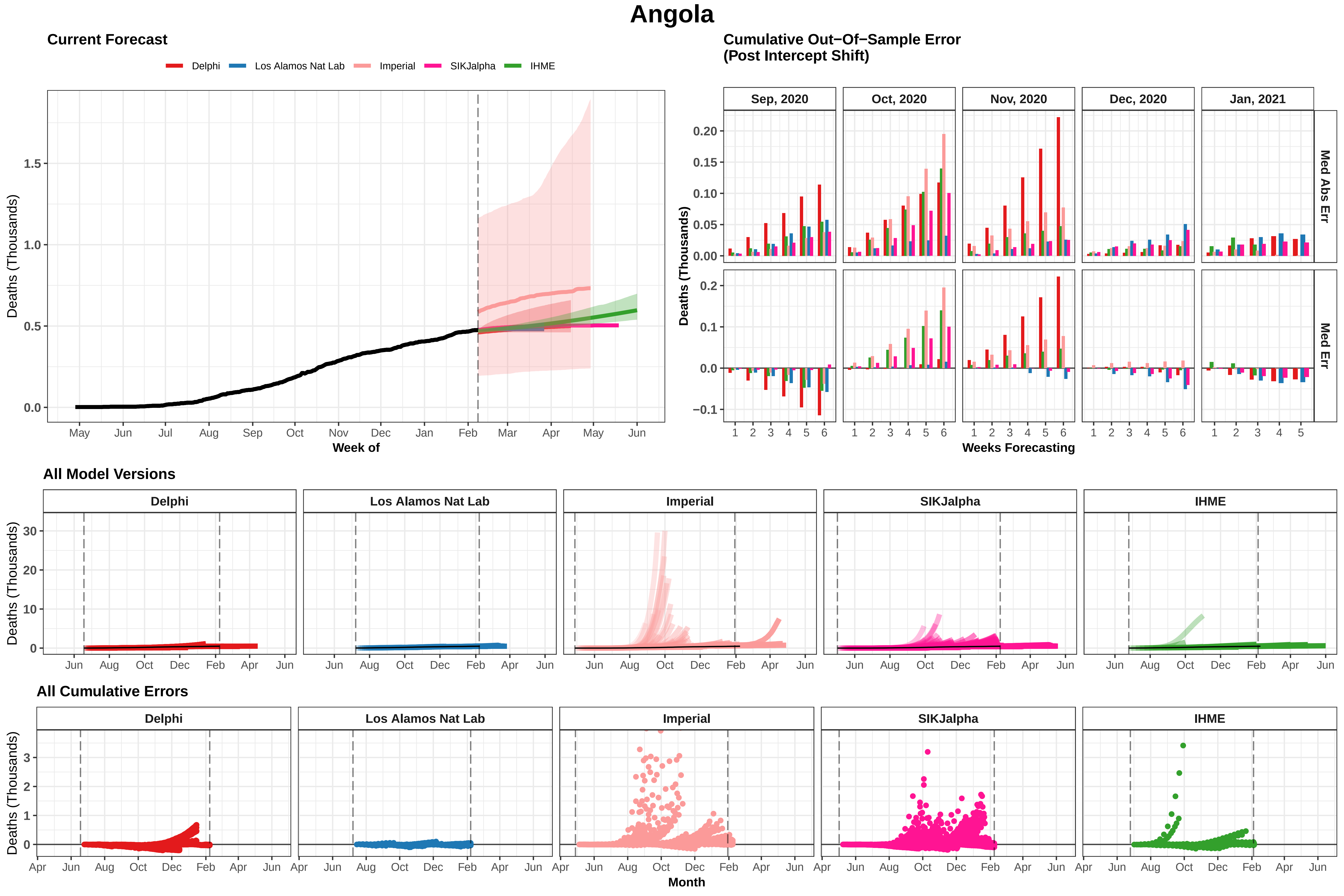

Angola
Current Forecast
Delphi
Los Alamos Nat Lab
Imperial
SIKJalpha
IHME
Deaths (Thousands)
Week of
Cumulative Out−Of−Sample Error
(Post Intercept Shift)
Sep, 2020
Oct, 2020
Nov, 2020
Dec, 2020
Jan, 2021
Med Abs Err
Med Err
Weeks Forecasting
All Model Versions
All Cumulative Errors
Month

Cameroon

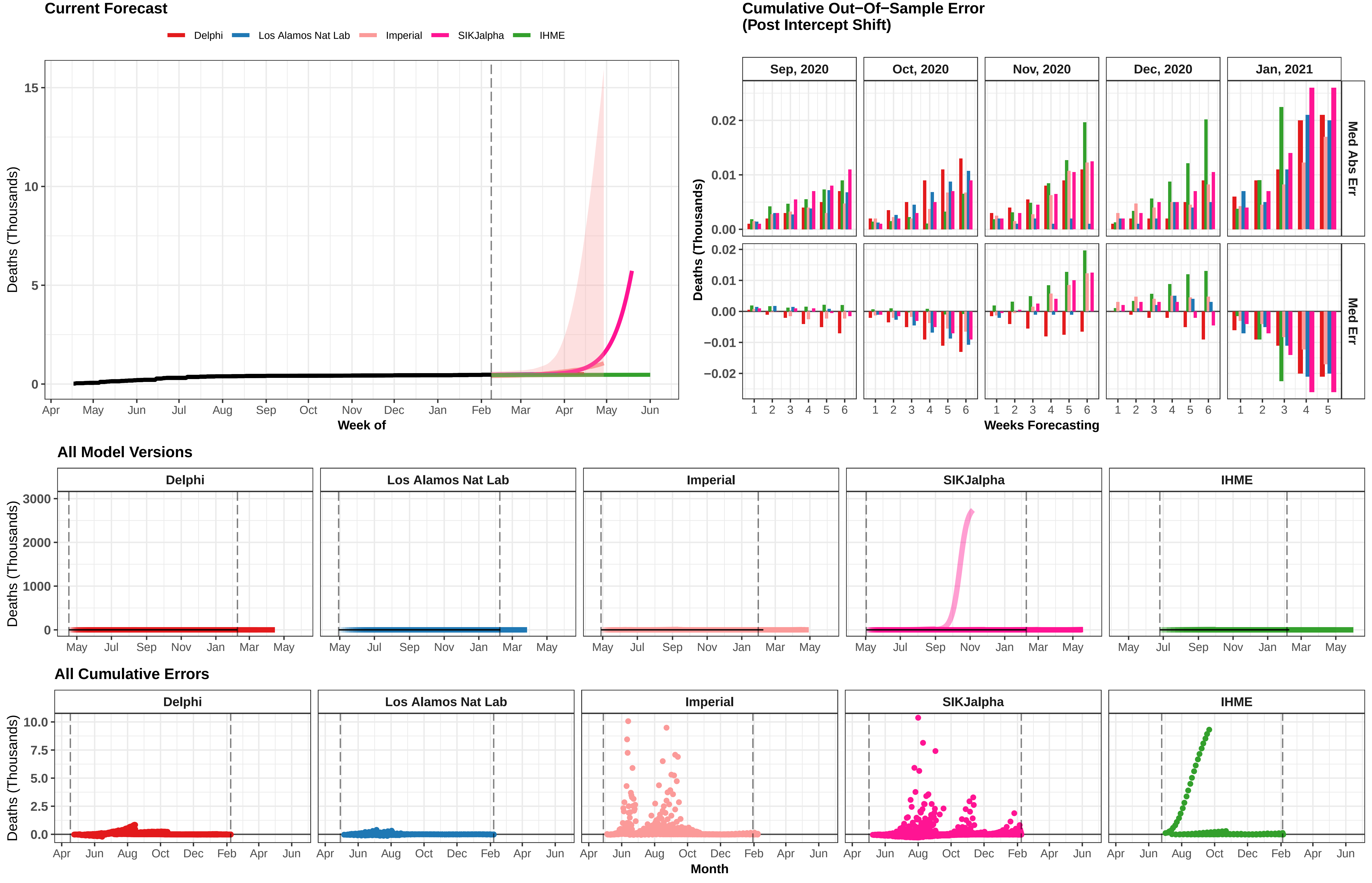

Current Forecast
Delphi
Los Alamos Nat Lab
Imperial
SIKJalpha
IHME
Deaths (Thousands)
Week of
Cumulative Out−Of−Sample Error
(Post Intercept Shift)
Sep, 2020
Oct, 2020
Nov, 2020
Dec, 2020
Jan, 2021
Med Abs Err
Med Err
Weeks Forecasting
All Model Versions
All Cumulative Errors
Month

Ghana

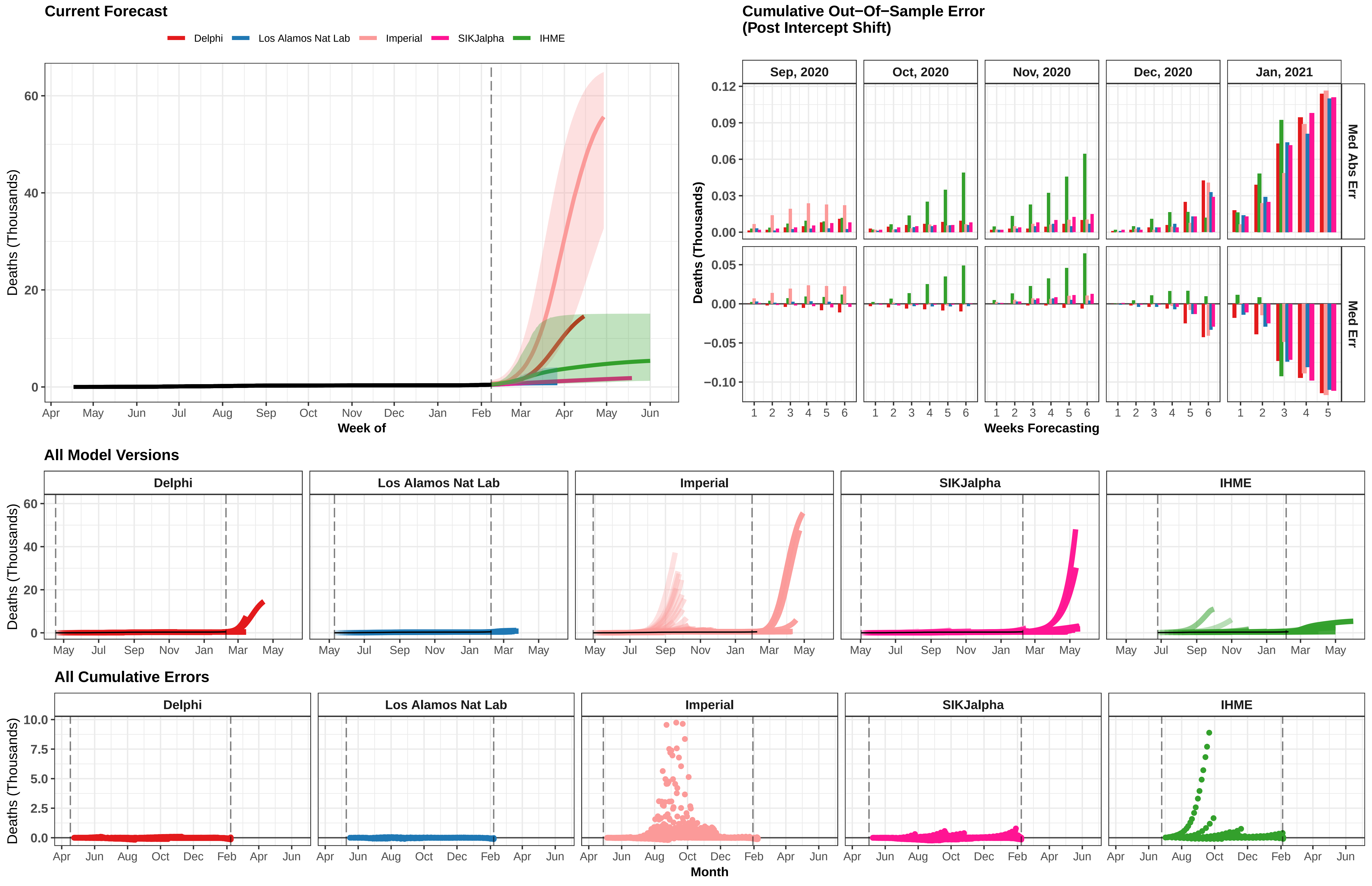

Current Forecast
Delphi
Los Alamos Nat Lab
Imperial
SIKJalpha
IHME
Deaths (Thousands)
Week of
Apr May Jun Jul Aug Sep Oct Nov Dec Jan Feb Mar Apr May Jun
Cumulative Out−Of−Sample Error
(Post Intercept Shift)
Sep, 2020
Oct, 2020
Nov, 2020
Dec, 2020
Jan, 2021
Med Abs Err
Med Err
Weeks Forecasting
All Model Versions
All Cumulative Errors
Month

Estonia

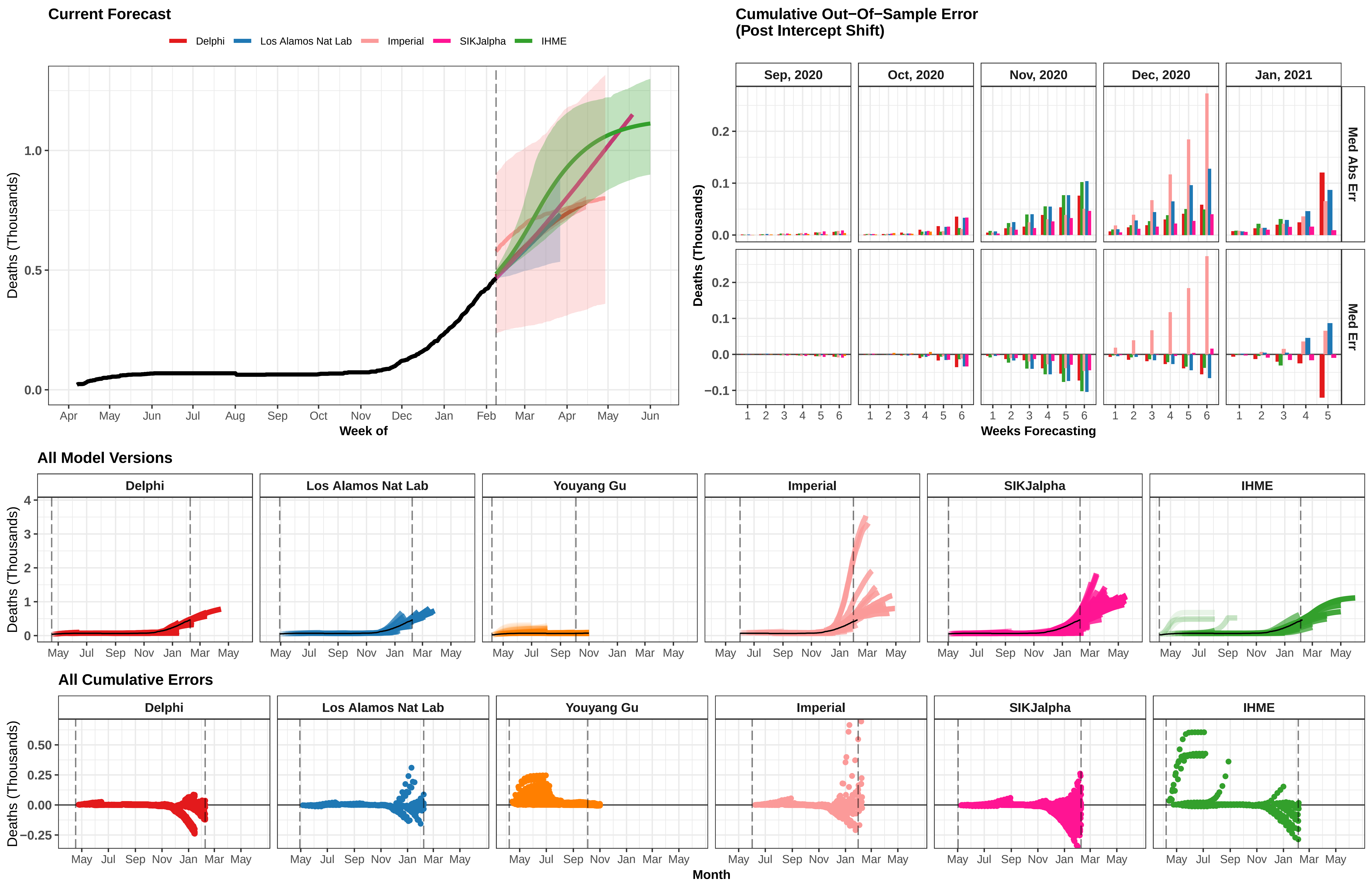

Current Forecast
Delphi
Los Alamos Nat Lab
Imperial
SIKJalpha
IHME
Deaths (Thousands)
Week of
Apr May Jun Jul Aug Sep Oct Nov Dec Jan Feb Mar Apr May Jun
Cumulative Out−Of−Sample Error
(Post Intercept Shift)
Sep, 2020
Oct, 2020
Nov, 2020
Dec, 2020
Jan, 2021
Med Abs Err
Med Err
Weeks Forecasting
All Model Versions
Delphi
Los Alamos Nat Lab
Youyang Gu
Imperial
SIKJalpha
IHME
All Cumulative Errors
Month

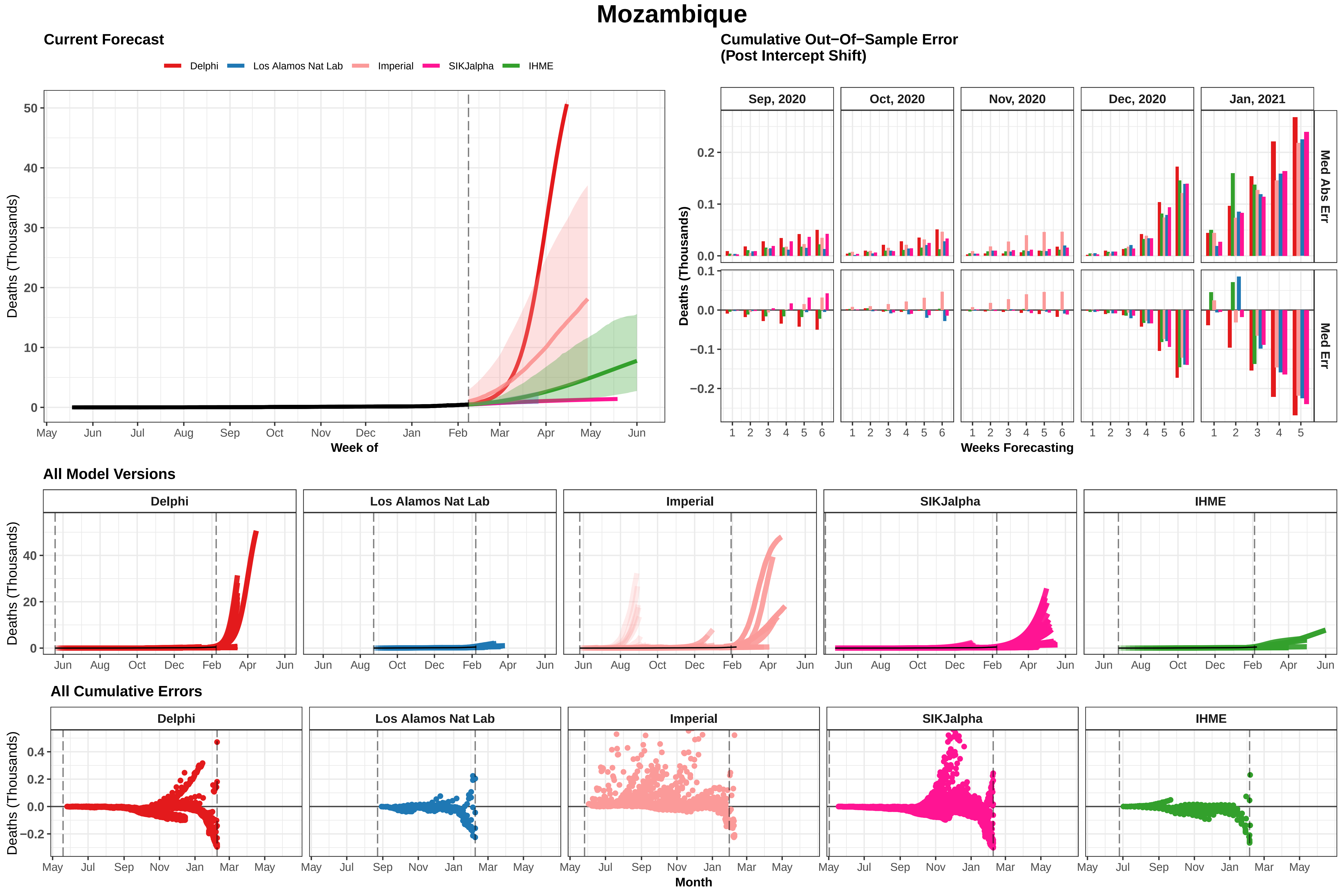

Mozambique
Current Forecast
Delphi
Los Alamos Nat Lab
Imperial
SIKJalpha
IHME
Deaths (Thousands)
Week of
Cumulative Out−Of−Sample Error
(Post Intercept Shift)
Sep, 2020
Oct, 2020
Nov, 2020
Dec, 2020
Jan, 2021
Med Abs Err
Med Err
Weeks Forecasting
All Model Versions
All Cumulative Errors
Month

Mauritania

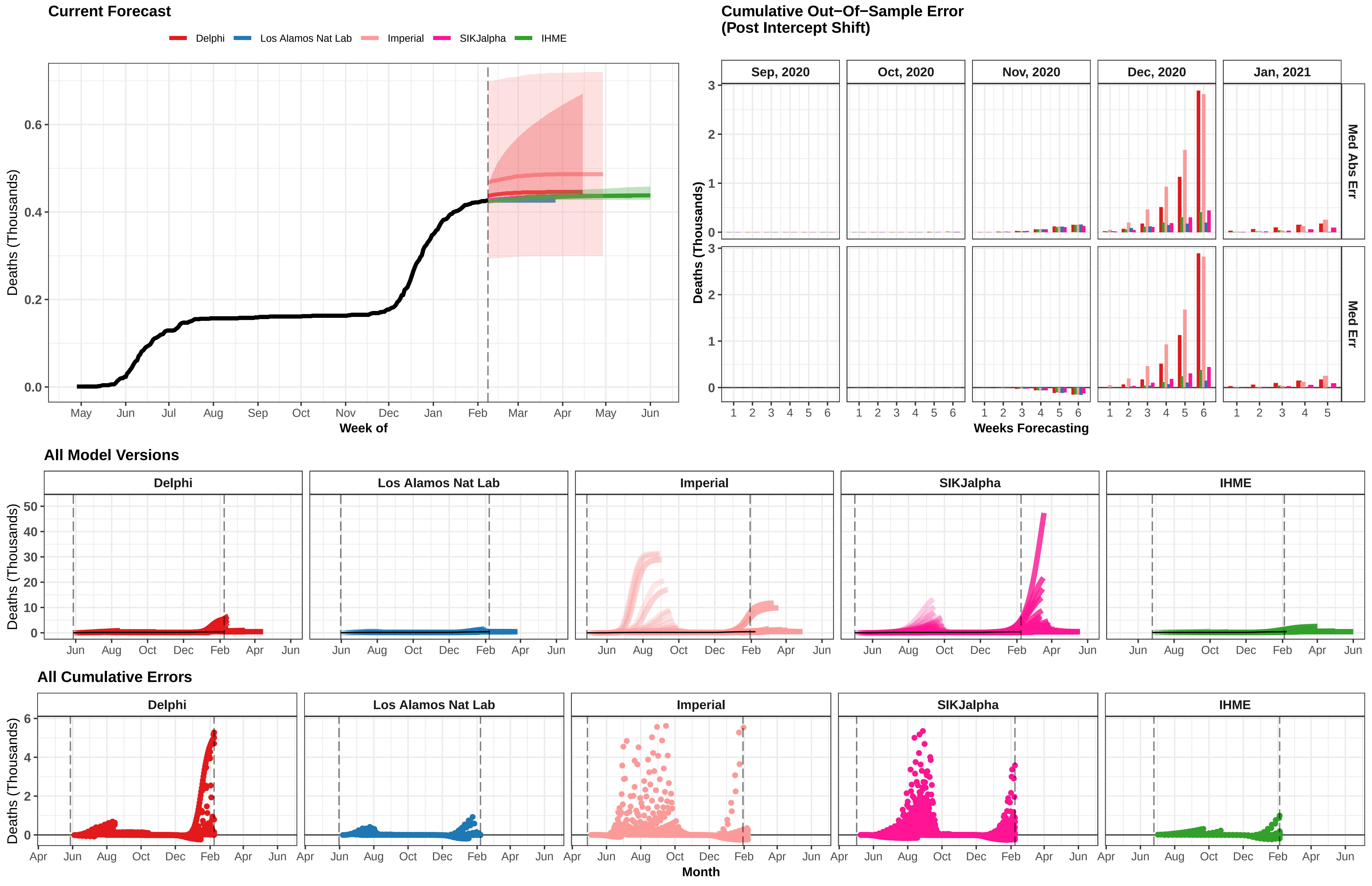
Current Forecast
Delphi
Los Alamos Nat Lab
Imperial
SIKJalpha
IHME
Deaths (Thousands)
0.0
0.2
0.4
0.6
May
Jun
Jul
Aug
Sep
Oct
Nov
Dec
Jan
Feb
Mar
Apr
May
Jun
Week of
Cumulative Out−Of−Sample Error
(Post Intercept Shift)
Sep, 2020
Oct, 2020
Nov, 2020
Dec, 2020
Jan, 2021
Med Abs Err
Med Err
Deaths (Thousands)
Weeks Forecasting
All Model Versions
Delphi
Los Alamos Nat Lab
Imperial
SIKJalpha
IHME
Deaths (Thousands)
0
10
20
30
40
50
All Cumulative Errors
Delphi
Los Alamos Nat Lab
Imperial
SIKJalpha
IHME
Deaths (Thousands)
0
2
4
6
Month

Hawaii

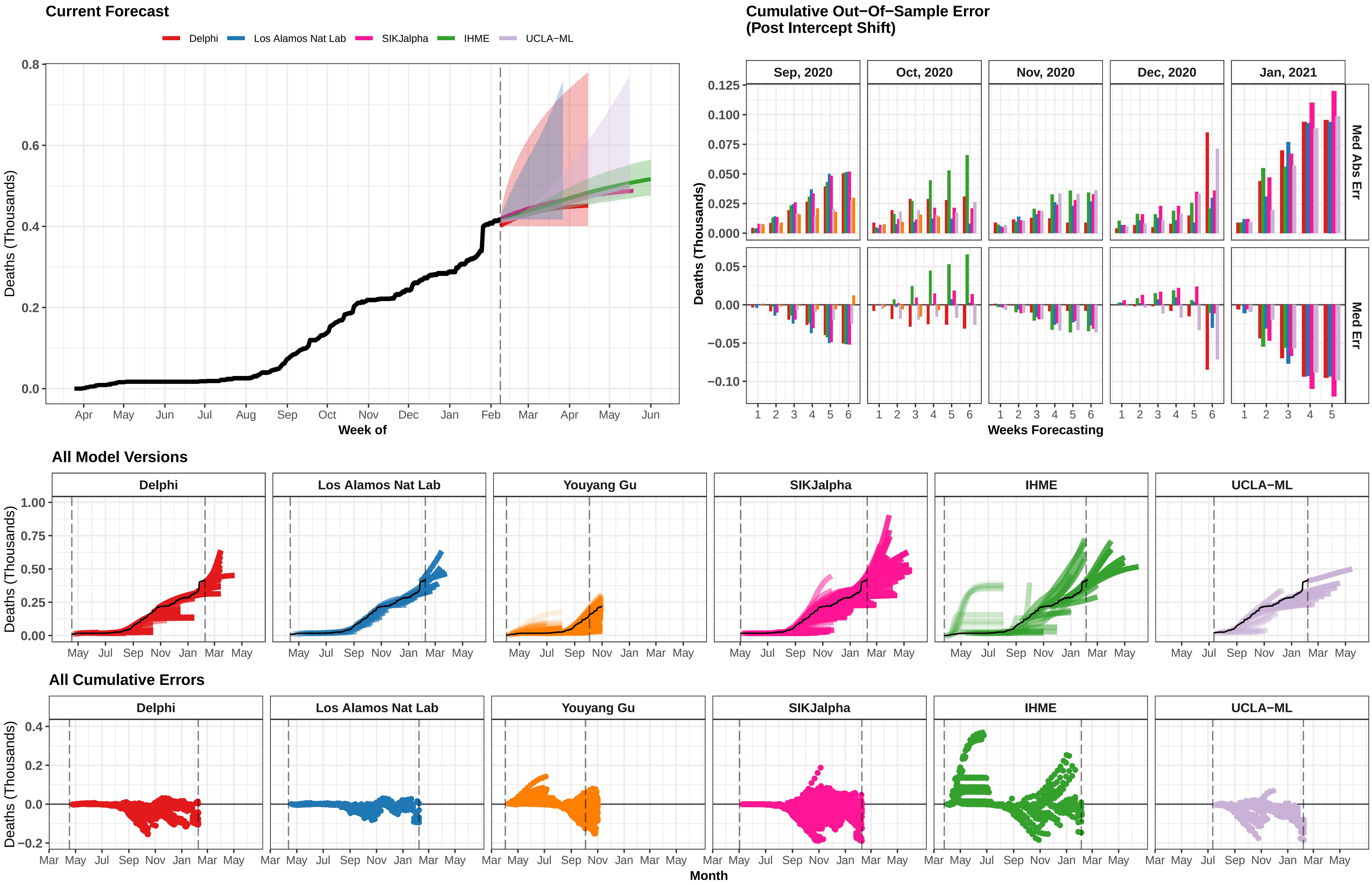
Current Forecast
Delphi
Los Alamos Nat Lab
SIKJalpha
IHME
UCLA−ML
Deaths (Thousands)
0.0
0.2
0.4
0.6
0.8
Apr May Jun Jul Aug Sep Oct Nov Dec Jan Feb Mar Apr May Jun
Week of
Cumulative Out−Of−Sample Error
(Post Intercept Shift)
Sep, 2020
Oct, 2020
Nov, 2020
Dec, 2020
Jan, 2021
Med Abs Err
Med Err
0.000
0.025
0.050
0.075
0.100
0.125
0.05
0.00
−0.05
−0.10
Weeks Forecasting
All Model Versions
Delphi
Los Alamos Nat Lab
Youyang Gu
SIKJalpha
IHME
UCLA−ML
0.00
0.25
0.50
0.75
1.00
May Jul Sep Nov Jan Mar May
All Cumulative Errors
0.4
0.2
0.0
−0.2
Mar May Jul Sep Nov Jan Mar May
Month

Bahrain

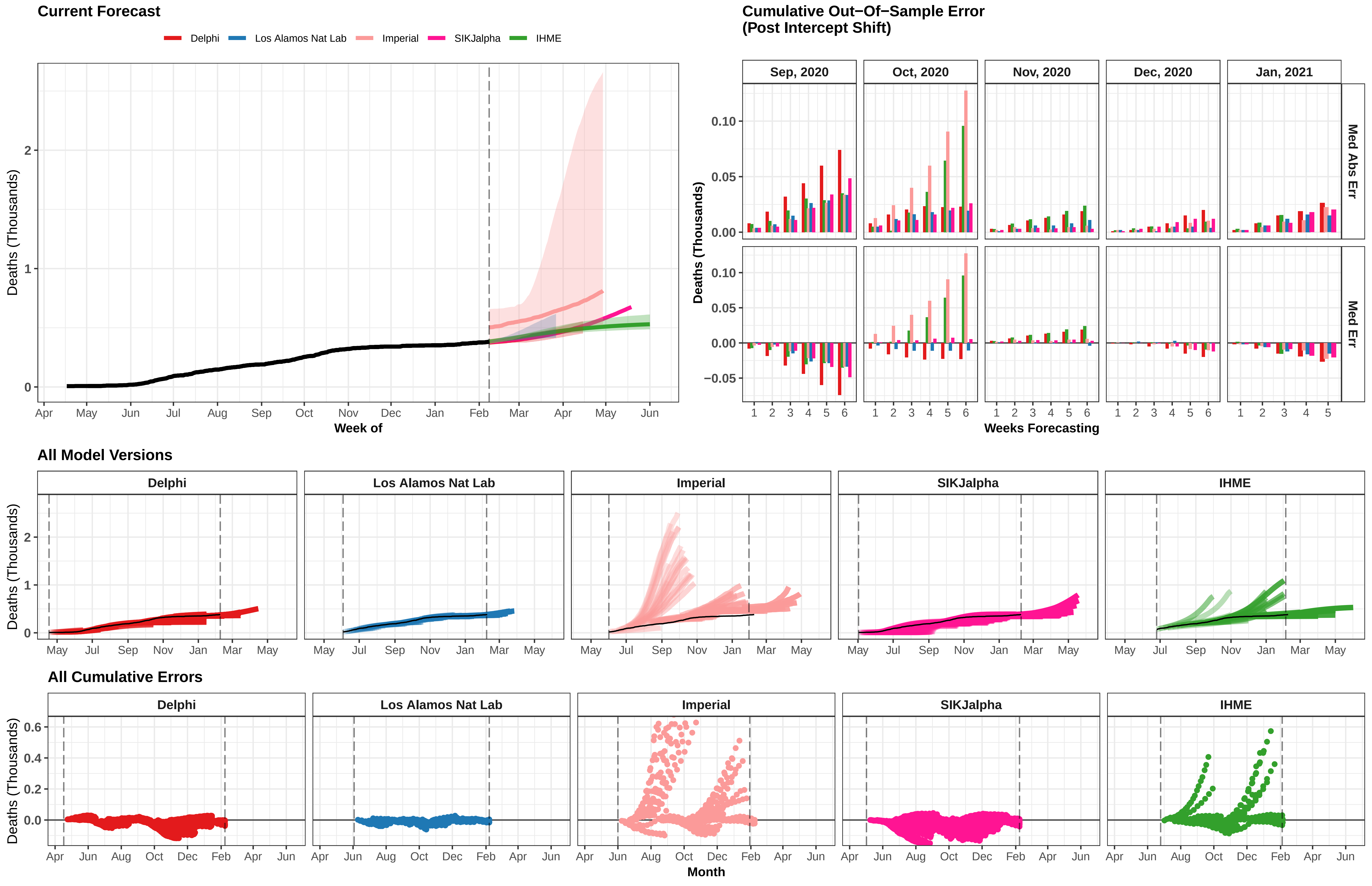

Current Forecast
Delphi
Los Alamos Nat Lab
Imperial
SIKJalpha
IHME
Deaths (Thousands)
Week of
Cumulative Out−Of−Sample Error
(Post Intercept Shift)
Sep, 2020
Oct, 2020
Nov, 2020
Dec, 2020
Jan, 2021
Med Abs Err
Med Err
Weeks Forecasting
All Model Versions
All Cumulative Errors
Month

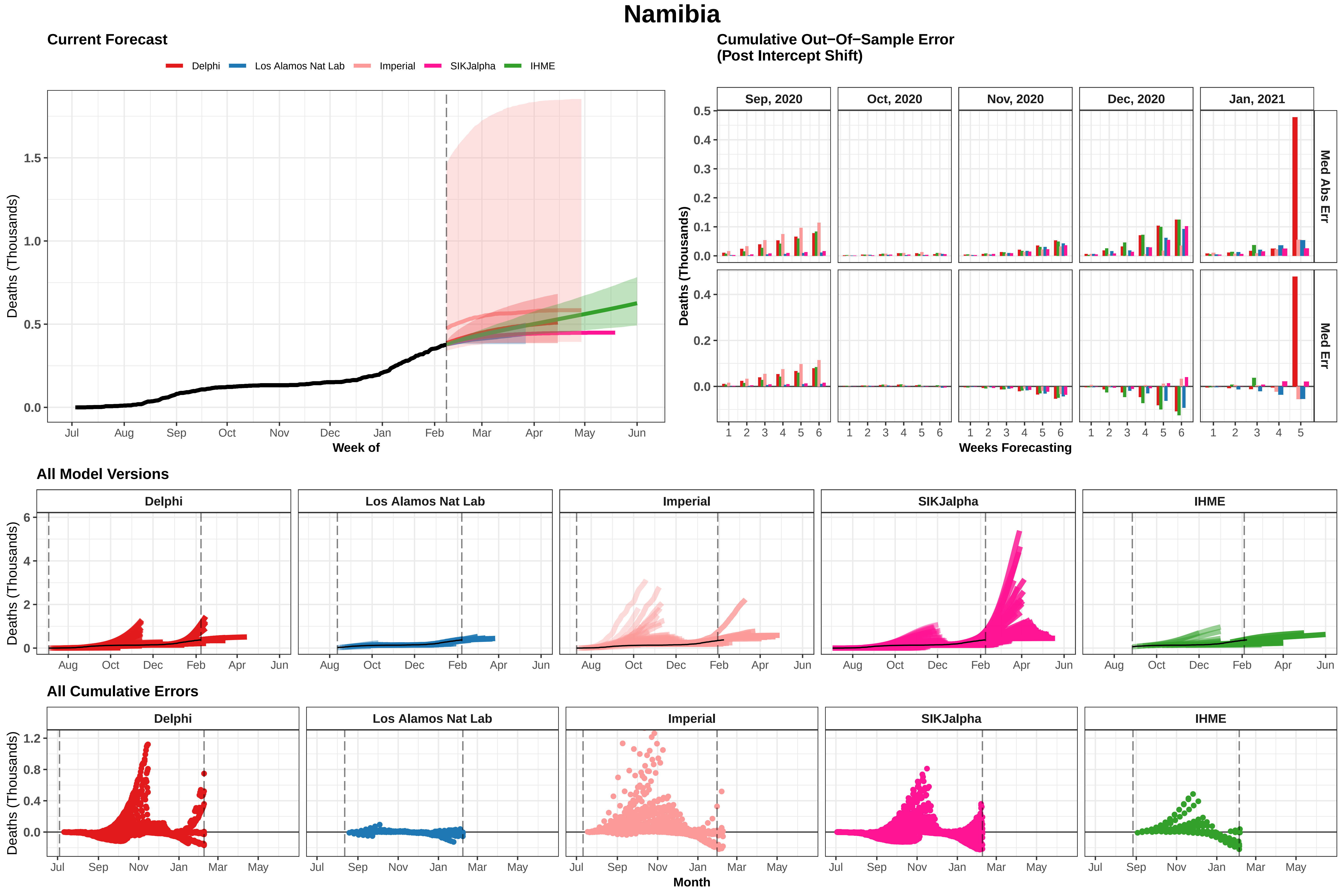

Namibia
Current Forecast
Delphi
Los Alamos Nat Lab
Imperial
SIKJalpha
IHME
Deaths (Thousands)
Week of
Cumulative Out−Of−Sample Error
(Post Intercept Shift)
Sep, 2020
Oct, 2020
Nov, 2020
Dec, 2020
Jan, 2021
Med Abs Err
Med Err
Weeks Forecasting
All Model Versions
All Cumulative Errors
Month

Sri Lanka

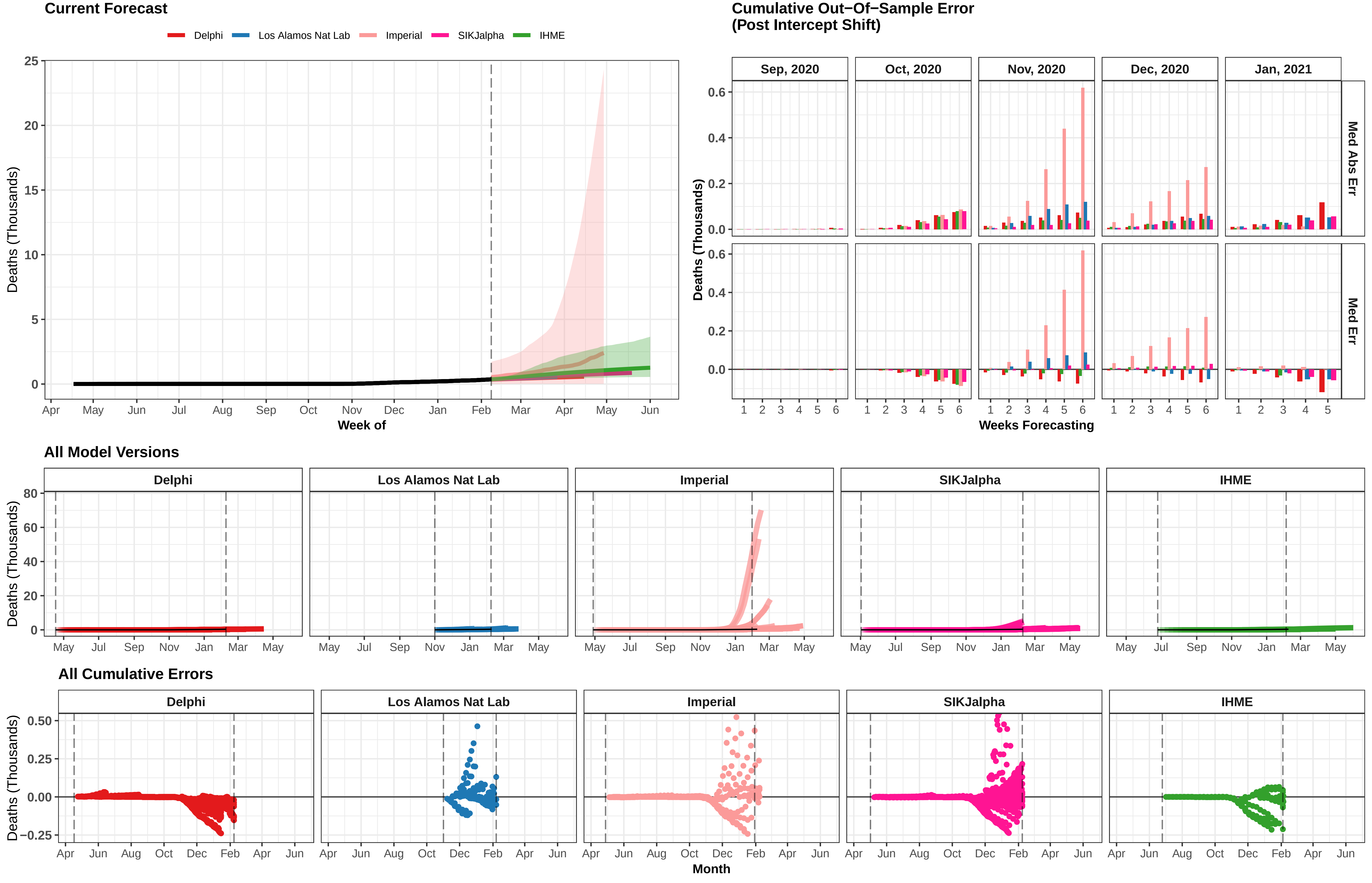
Current Forecast
Delphi
Los Alamos Nat Lab
Imperial
SIKJalpha
IHME
Deaths (Thousands)
Week of
Apr
May
Jun
Jul
Aug
Sep
Oct
Nov
Dec
Jan
Feb
Mar
Cumulative Out−Of−Sample Error
(Post Intercept Shift)
Sep, 2020
Oct, 2020
Nov, 2020
Dec, 2020
Jan, 2021
Med Abs Err
Med Err
Weeks Forecasting
All Model Versions
All Cumulative Errors
Month

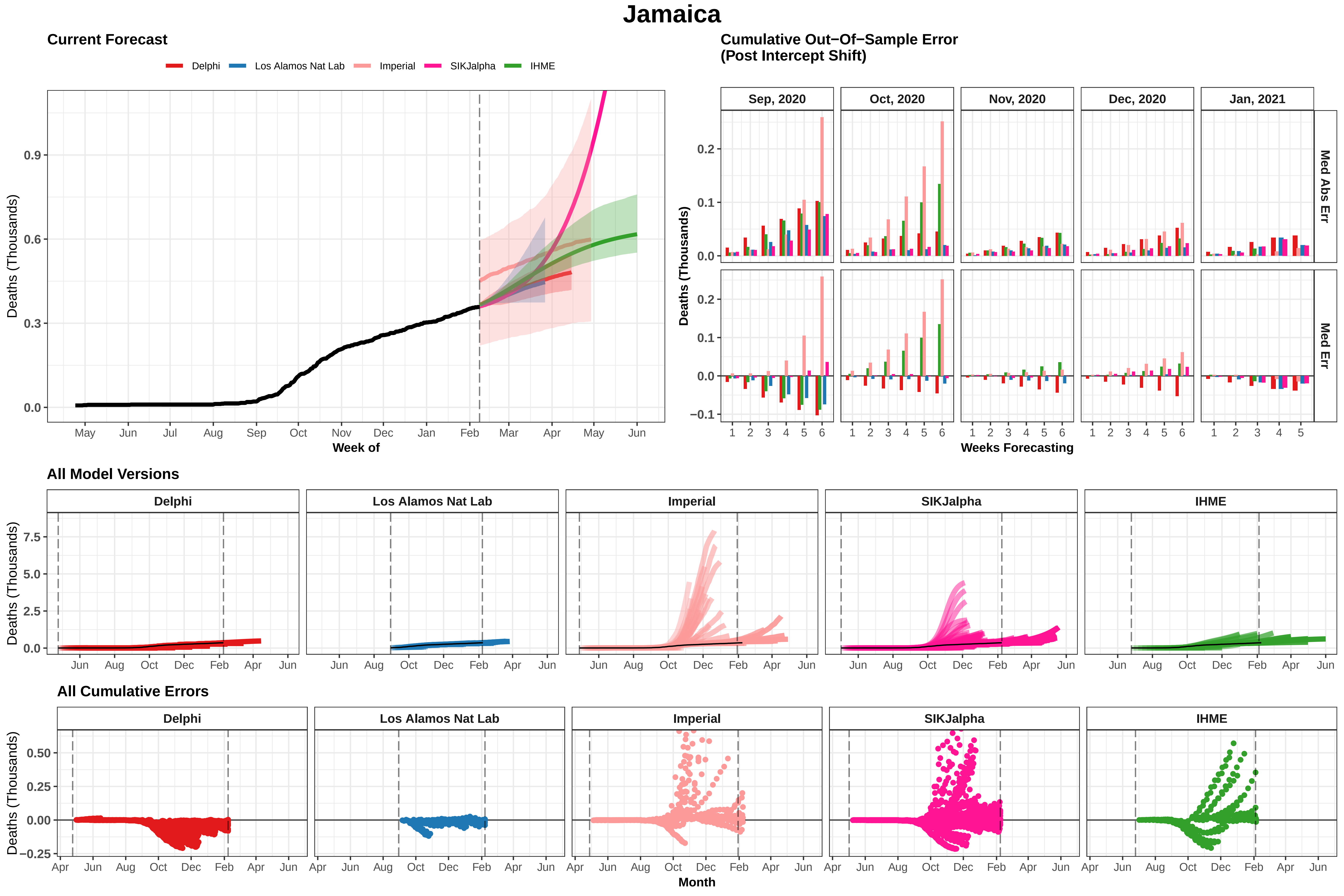
Jamaica
Current Forecast
Delphi
Los Alamos Nat Lab
Imperial
SIKJalpha
IHME
Deaths (Thousands)
Week of
Cumulative Out−Of−Sample Error
(Post Intercept Shift)
Sep, 2020
Oct, 2020
Nov, 2020
Dec, 2020
Jan, 2021
Med Abs Err
Med Err
Weeks Forecasting
All Model Versions
All Cumulative Errors
Month

Mali

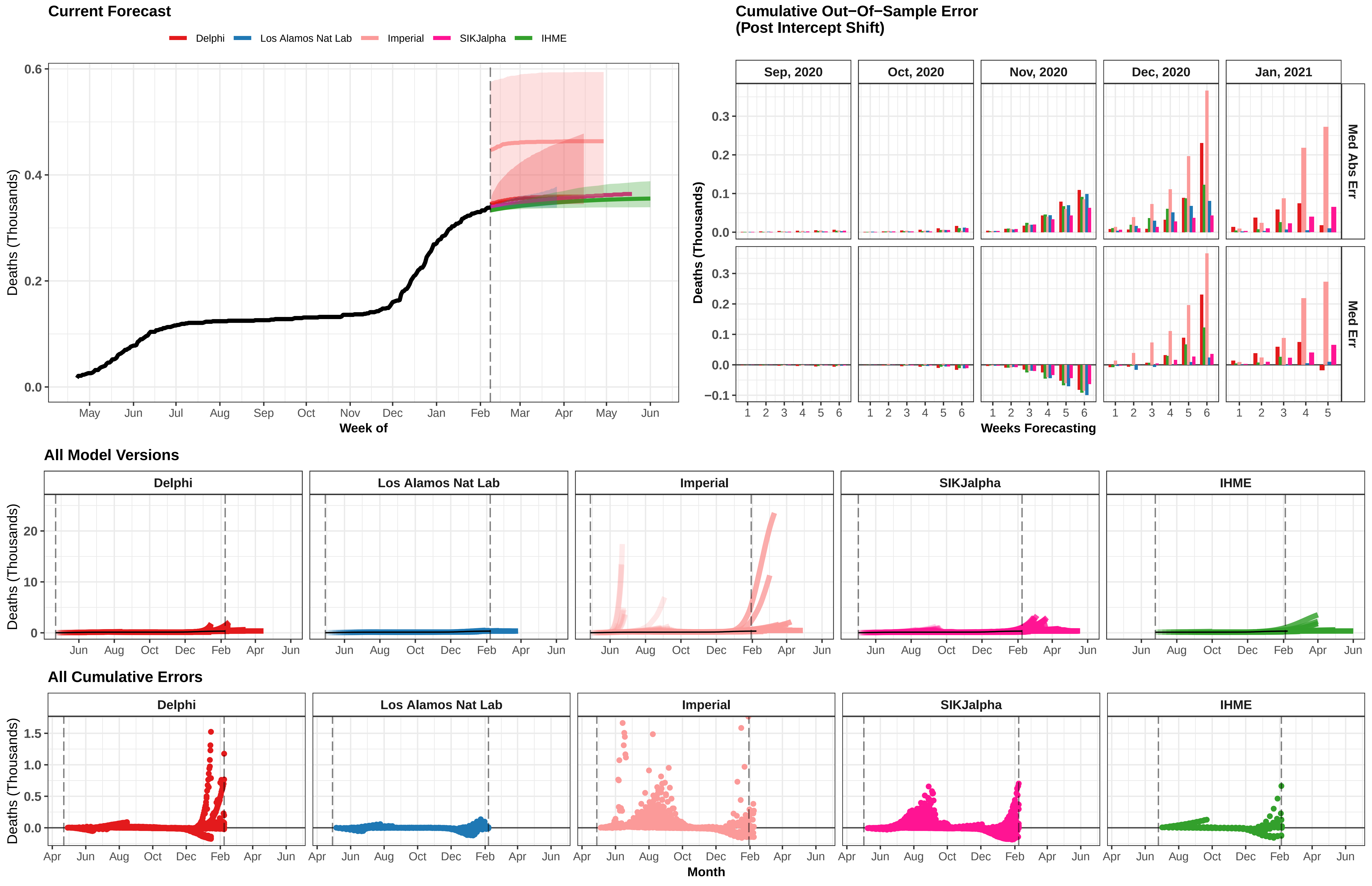

Current Forecast
Delphi
Los Alamos Nat Lab
Imperial
SIKJalpha
IHME
Deaths (Thousands)
Week of
May
Jun
Jul
Aug
Sep
Oct
Nov
Dec
Jan
Feb
Mar
Apr
Cumulative Out−Of−Sample Error
(Post Intercept Shift)
Sep, 2020
Oct, 2020
Nov, 2020
Dec, 2020
Jan, 2021
Med Abs Err
Med Err
Weeks Forecasting
All Model Versions
All Cumulative Errors
Month

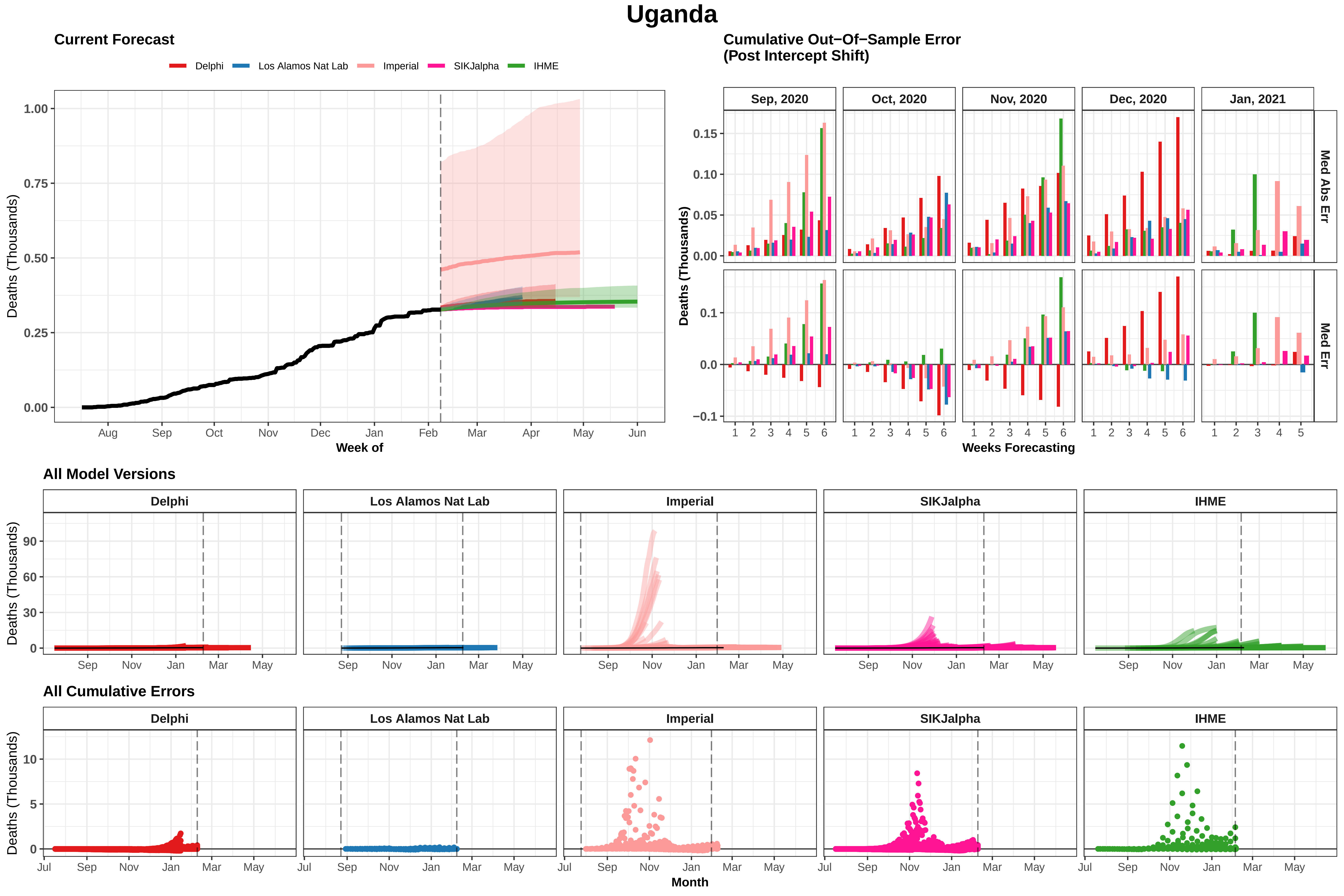

Uganda
Current Forecast
Delphi
Los Alamos Nat Lab
Imperial
SIKJalpha
IHME
Deaths (Thousands)
1.00
0.75
0.50
0.25
0.00
Aug
Sep
Oct
Nov
Dec
Jan
Feb
Mar
Apr
May
Jun
Week of
Cumulative Out−Of−Sample Error
(Post Intercept Shift)
Sep, 2020
Oct, 2020
Nov, 2020
Dec, 2020
Jan, 2021
Med Abs Err
Med Err
0.15
0.10
0.05
0.00
0.1
0.0
−0.1
1
2
3
4
5
6
Weeks Forecasting
All Model Versions
90
60
30
0
All Cumulative Errors
10
5
0
Jul
Month

Belize

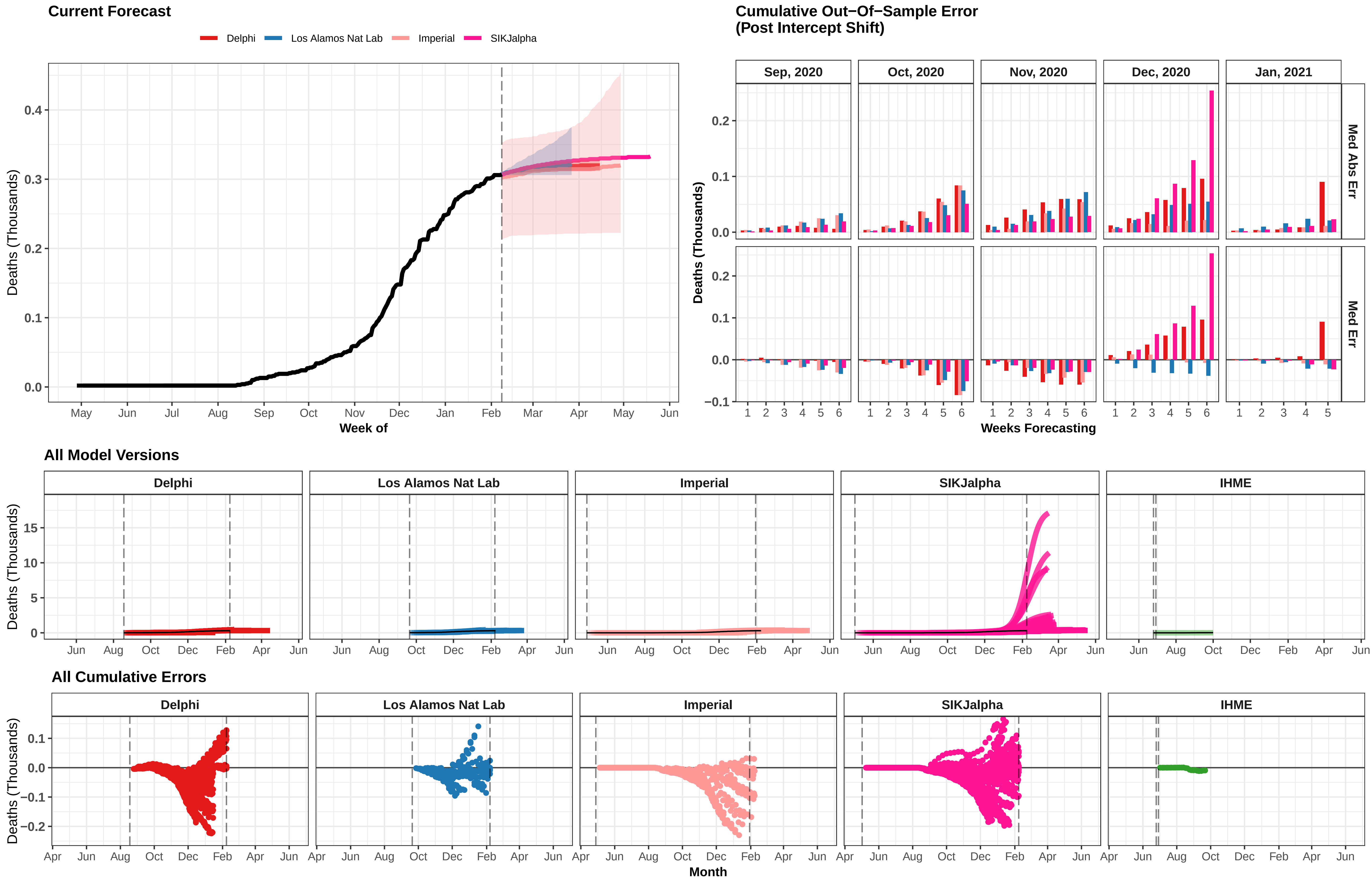
Current Forecast
Delphi
Los Alamos Nat Lab
Imperial
SIKJalpha
Deaths (Thousands)
Week of
Cumulative Out−Of−Sample Error
(Post Intercept Shift)
Sep, 2020
Oct, 2020
Nov, 2020
Dec, 2020
Jan, 2021
Med Abs Err
Med Err
Weeks Forecasting
All Model Versions
IHME
All Cumulative Errors
Month

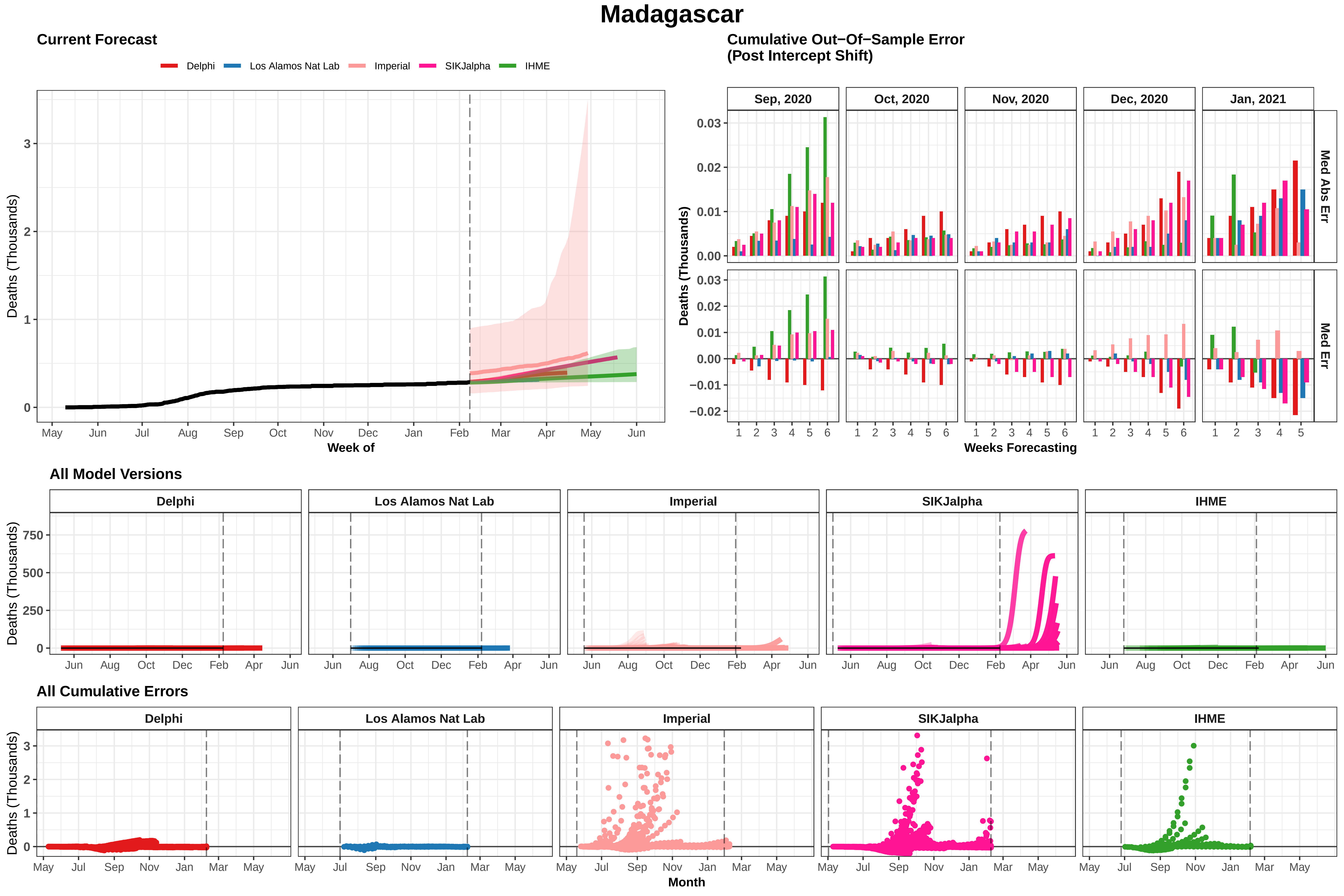

Madagascar
Current Forecast
Delphi
Los Alamos Nat Lab
Imperial
SIKJalpha
IHME
Deaths (Thousands)
Week of
Cumulative Out−Of−Sample Error
(Post Intercept Shift)
Sep, 2020
Oct, 2020
Nov, 2020
Dec, 2020
Jan, 2021
Med Abs Err
Med Err
Weeks Forecasting
All Model Versions
All Cumulative Errors
Month

Malta

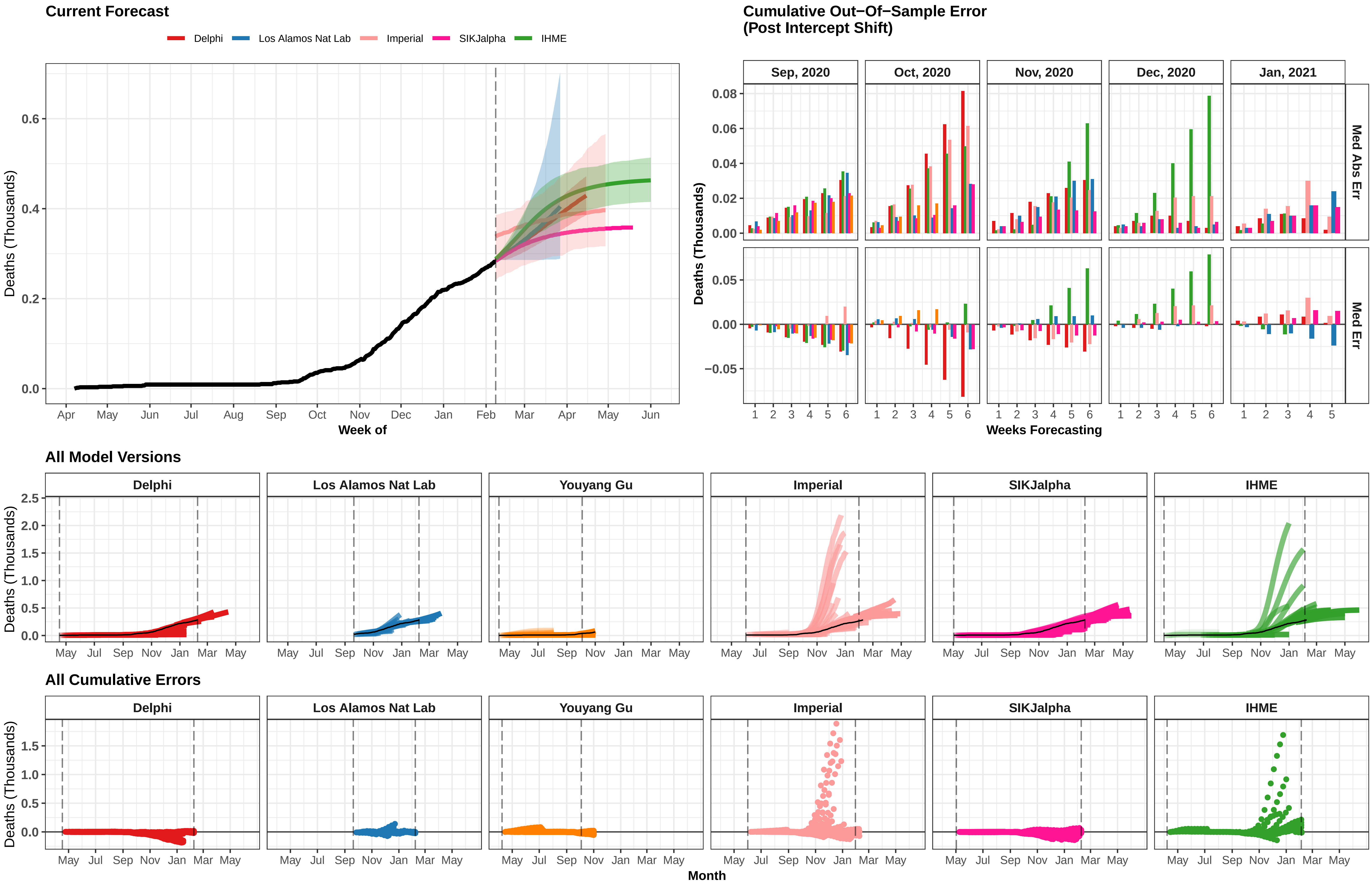

Current Forecast
Delphi
Los Alamos Nat Lab
Imperial
SIKJalpha
IHME
Deaths (Thousands)
0.0
0.2
0.4
0.6
Apr May Jun Jul Aug Sep Oct Nov Dec Jan Feb Mar Apr May Jun
Week of
Cumulative Out−Of−Sample Error
(Post Intercept Shift)
Sep, 2020
Oct, 2020
Nov, 2020
Dec, 2020
Jan, 2021
0.00
0.02
0.04
0.06
0.08
Med Abs Err
−0.05
0.00
0.05
Med Err
Weeks Forecasting
All Model Versions
Delphi
Los Alamos Nat Lab
Youyang Gu
Imperial
SIKJalpha
IHME
0.0
0.5
1.0
1.5
2.0
2.5
May Jul Sep Nov Jan Mar May
All Cumulative Errors
0.0
0.5
1.0
1.5
Month

Alaska

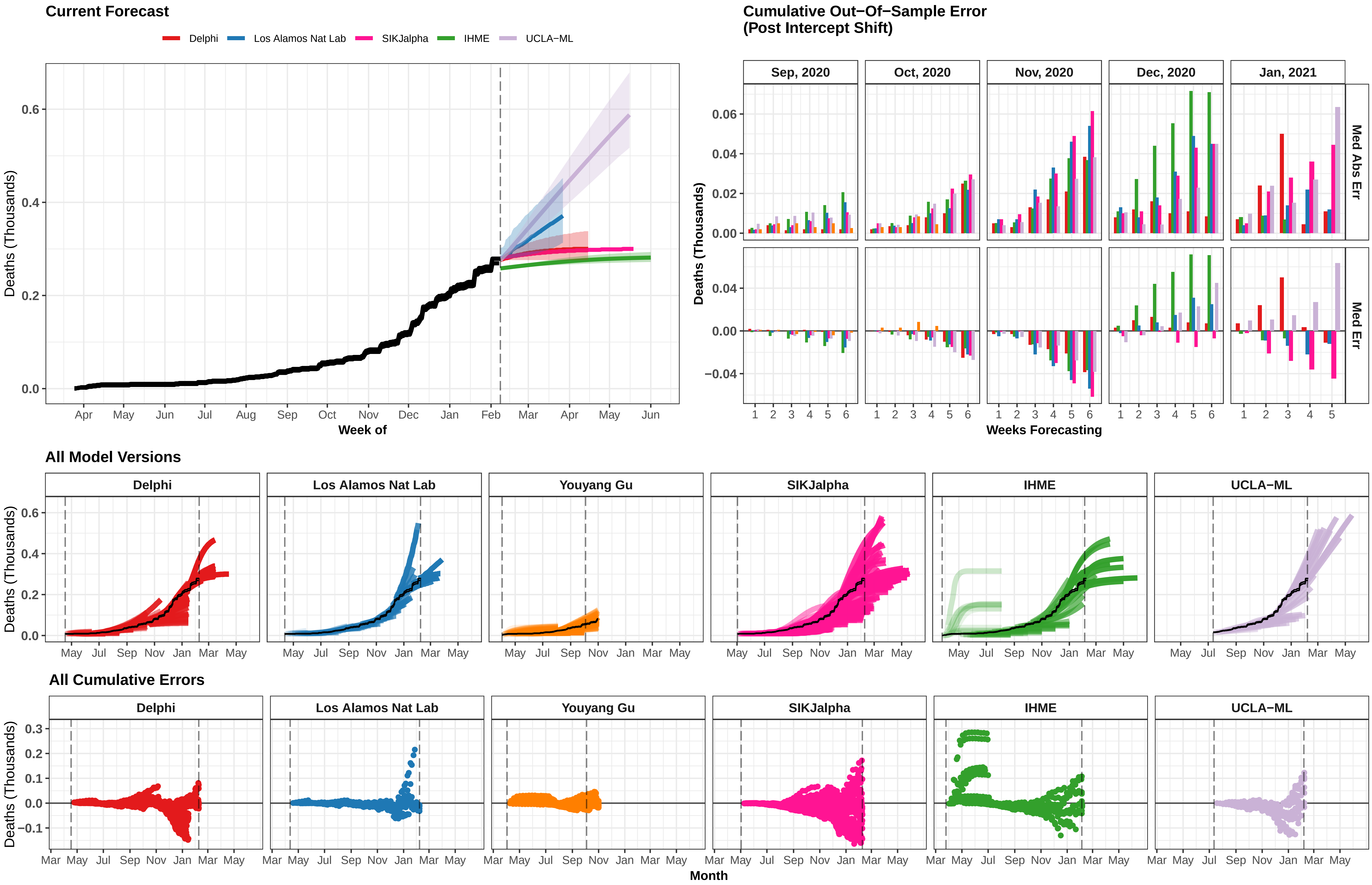
Current Forecast
Delphi
Los Alamos Nat Lab
SIKJalpha
IHME
UCLA−ML
Deaths (Thousands)
Week of
Apr
May
Jun
Jul
Aug
Sep
Oct
Nov
Dec
Jan
Feb
Mar
Cumulative Out−Of−Sample Error
(Post Intercept Shift)
Sep, 2020
Oct, 2020
Nov, 2020
Dec, 2020
Jan, 2021
Med Abs Err
Med Err
Weeks Forecasting
All Model Versions
Delphi
Los Alamos Nat Lab
Youyang Gu
SIKJalpha
IHME
UCLA−ML
All Cumulative Errors
Month

Qatar

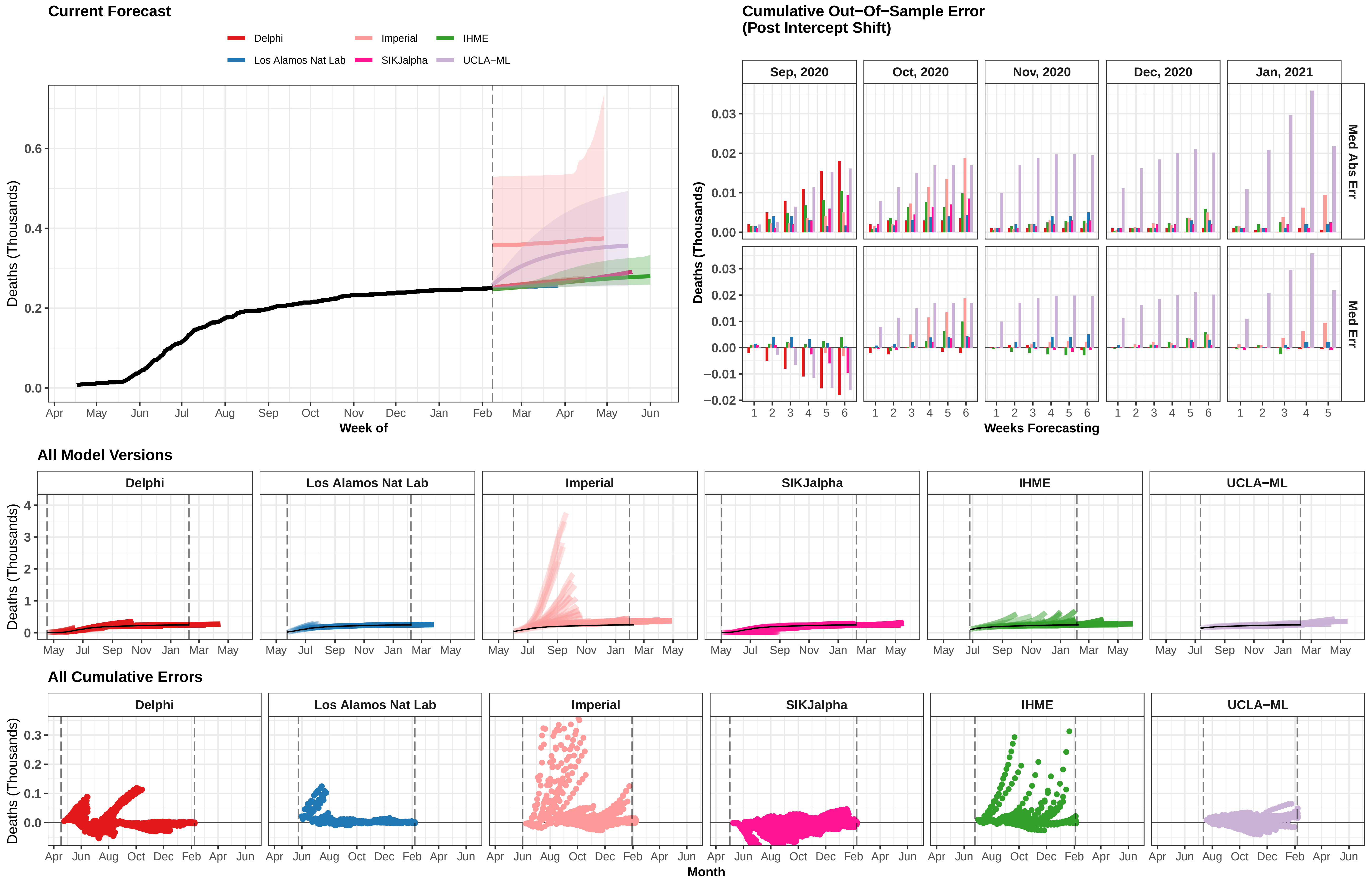

Current Forecast
Delphi
Imperial
IHME
Los Alamos Nat Lab
SIKJalpha
UCLA−ML
Deaths (Thousands)
Week of
Cumulative Out−Of−Sample Error
(Post Intercept Shift)
Sep, 2020
Oct, 2020
Nov, 2020
Dec, 2020
Jan, 2021
Med Abs Err
Med Err
Weeks Forecasting
All Model Versions
All Cumulative Errors
Month

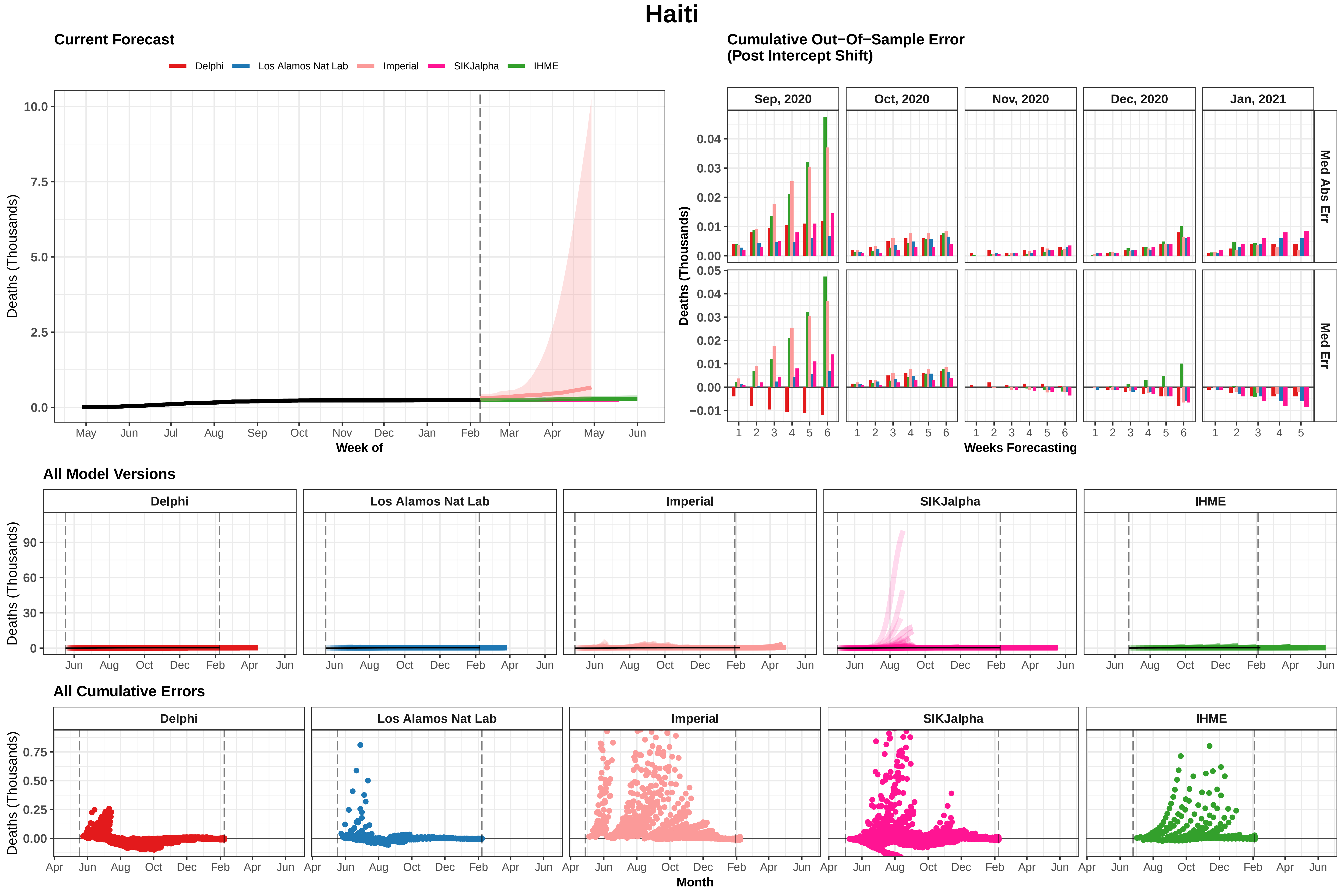
Haiti
Current Forecast
Delphi
Los Alamos Nat Lab
Imperial
SIKJalpha
IHME
Deaths (Thousands)
Week of
Cumulative Out−Of−Sample Error
(Post Intercept Shift)
Sep, 2020
Oct, 2020
Nov, 2020
Dec, 2020
Jan, 2021
Med Abs Err
Med Err
Weeks Forecasting
All Model Versions
All Cumulative Errors
Month

Cuba

Current Forecast

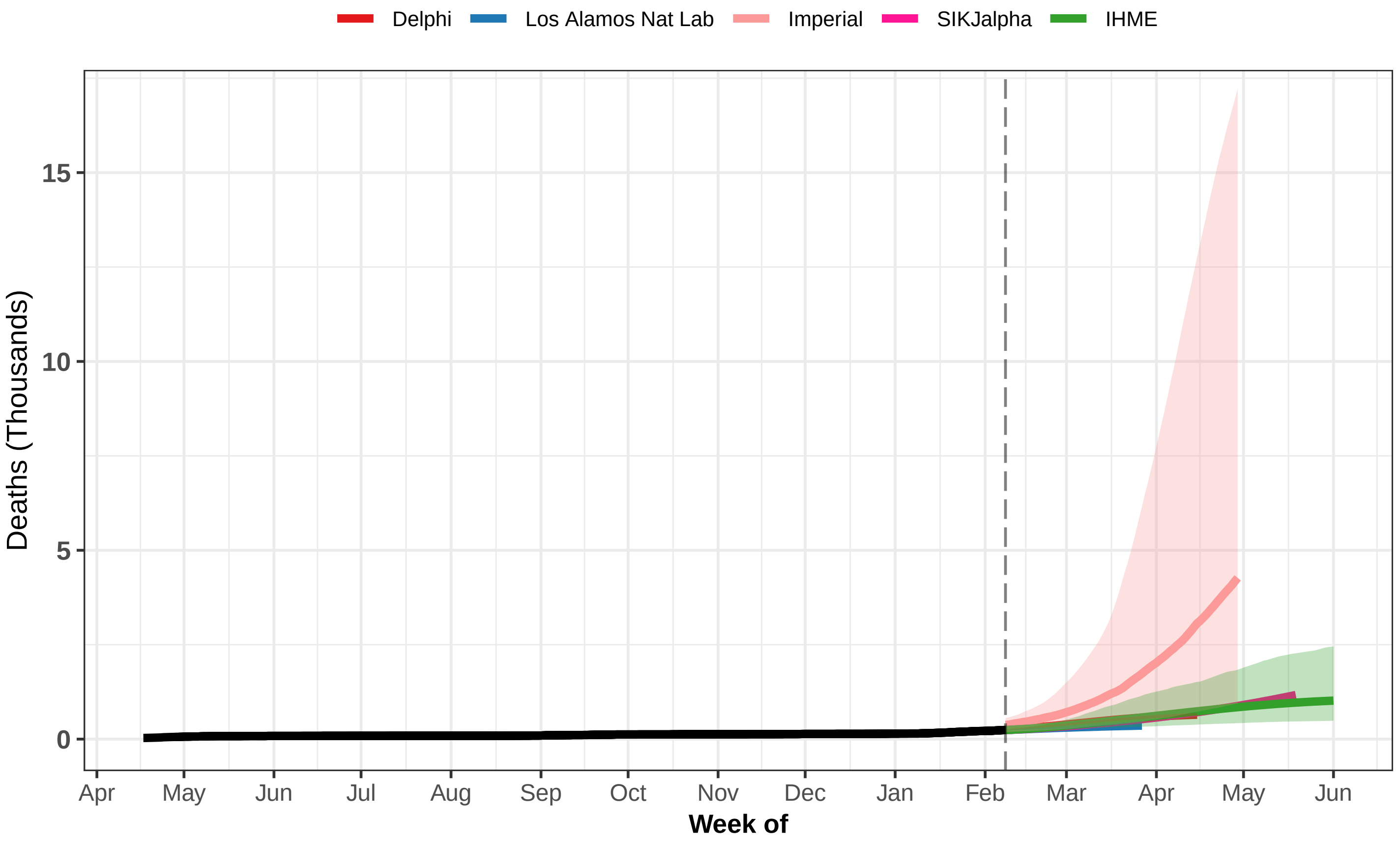

Cumulative Out−Of−Sample Error (Post Intercept Shift)

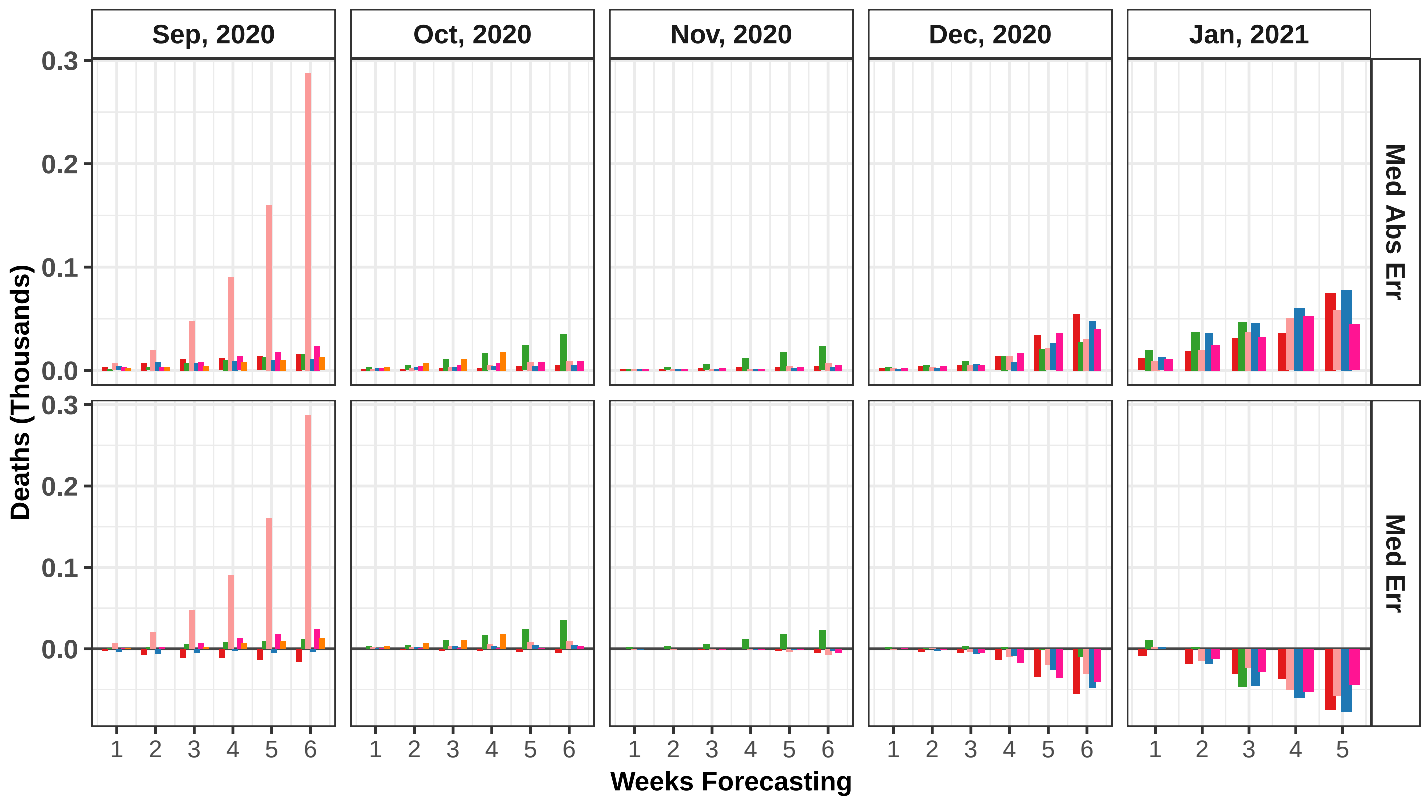

All Model Versions

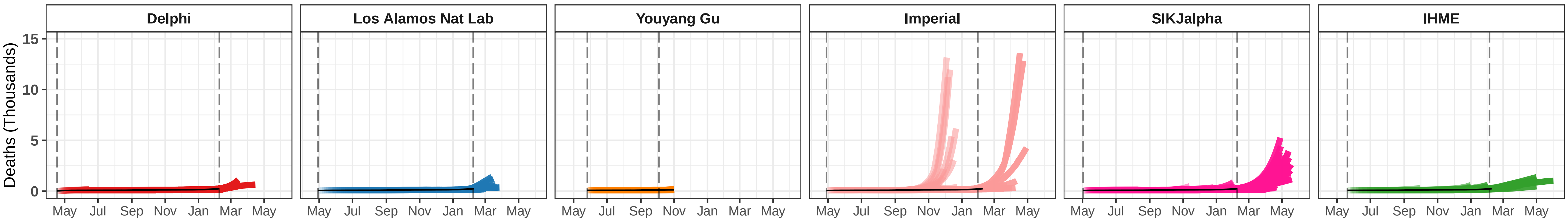

All Cumulative Errors

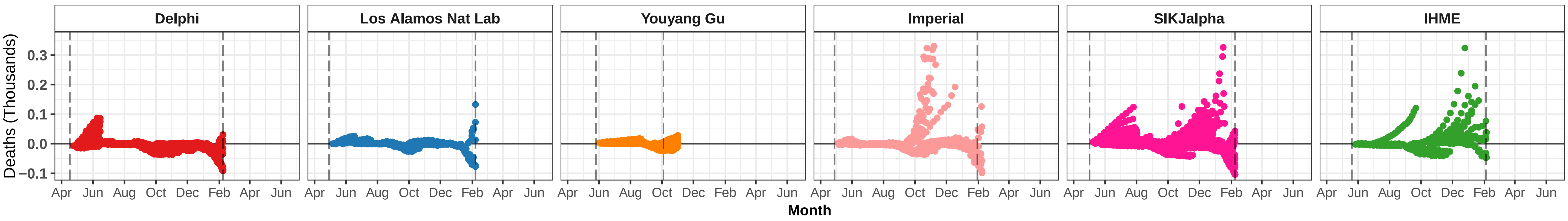

Rwanda

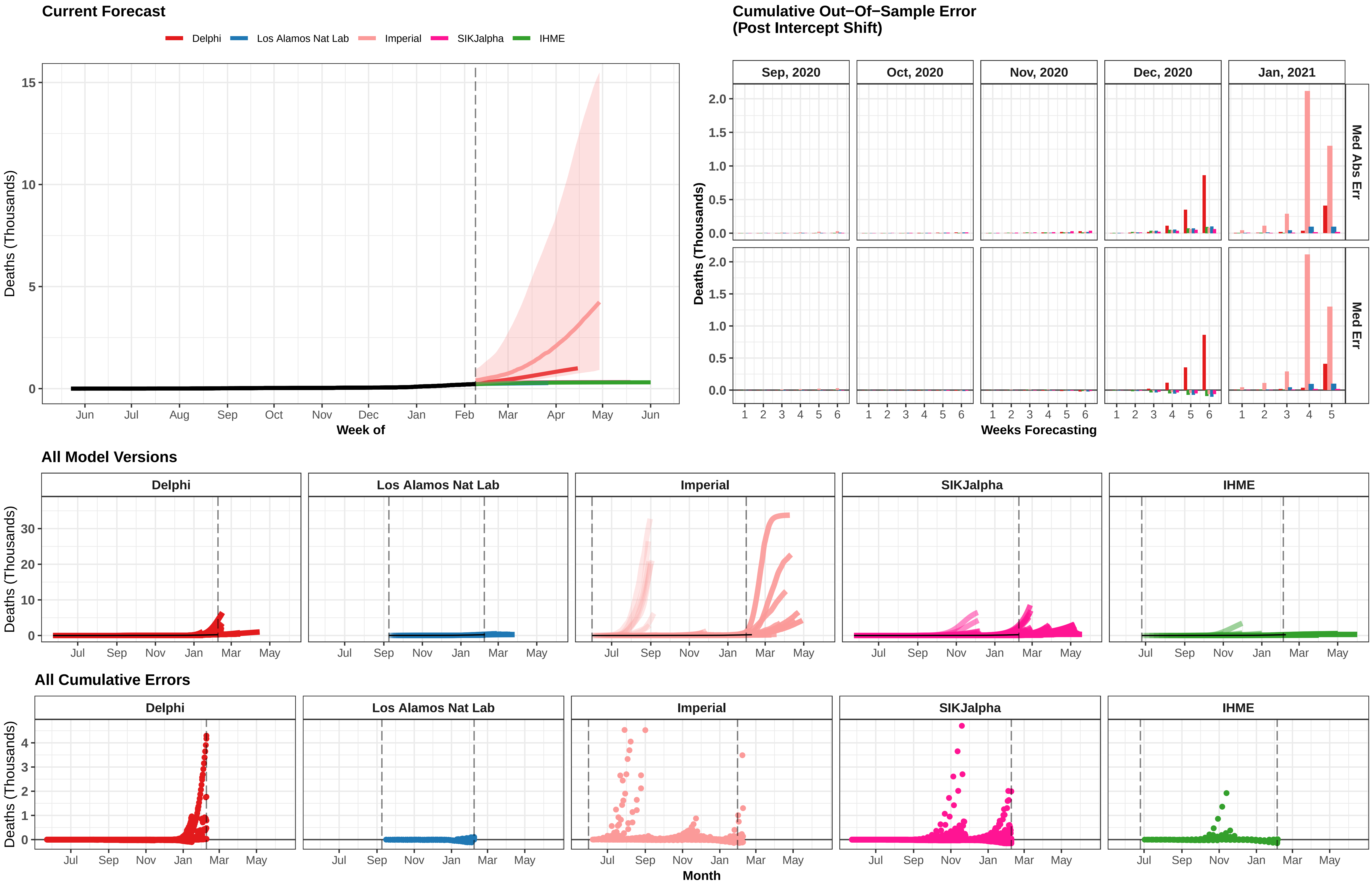

Current Forecast
Delphi
Los Alamos Nat Lab
Imperial
SIKJalpha
IHME
Deaths (Thousands)
Week of
Jun Jul Aug Sep Oct Nov Dec Jan Feb Mar Apr May Jun
Cumulative Out−Of−Sample Error
(Post Intercept Shift)
Sep, 2020
Oct, 2020
Nov, 2020
Dec, 2020
Jan, 2021
Med Abs Err
Med Err
Weeks Forecasting
All Model Versions
All Cumulative Errors
Month

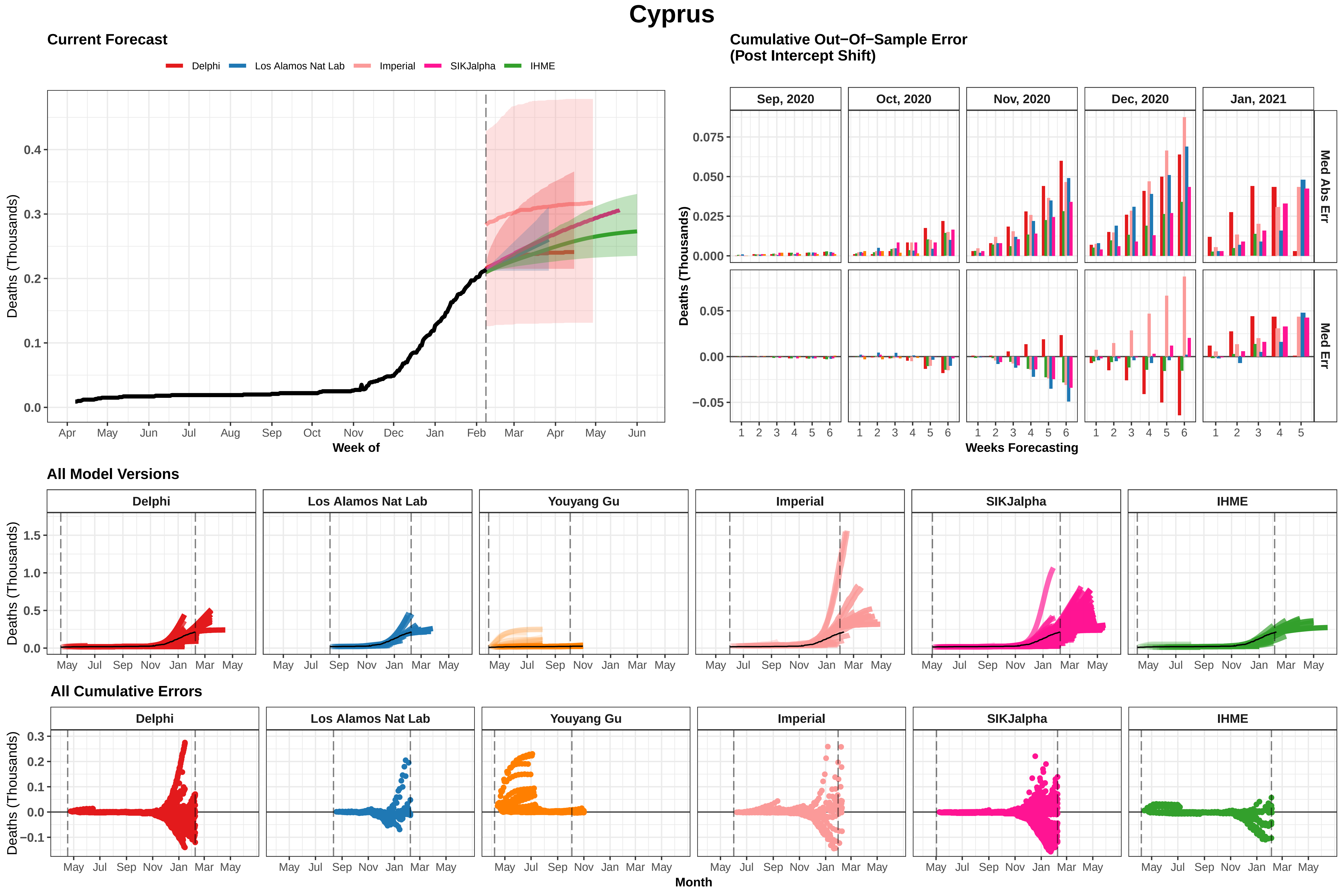

Cyprus
Current Forecast
Delphi
Los Alamos Nat Lab
Imperial
SIKJalpha
IHME
Deaths (Thousands)
Week of
Cumulative Out−Of−Sample Error
(Post Intercept Shift)
Sep, 2020
Oct, 2020
Nov, 2020
Dec, 2020
Jan, 2021
Med Abs Err
Med Err
Weeks Forecasting
All Model Versions
Delphi
Los Alamos Nat Lab
Youyang Gu
Imperial
SIKJalpha
IHME
All Cumulative Errors
Month

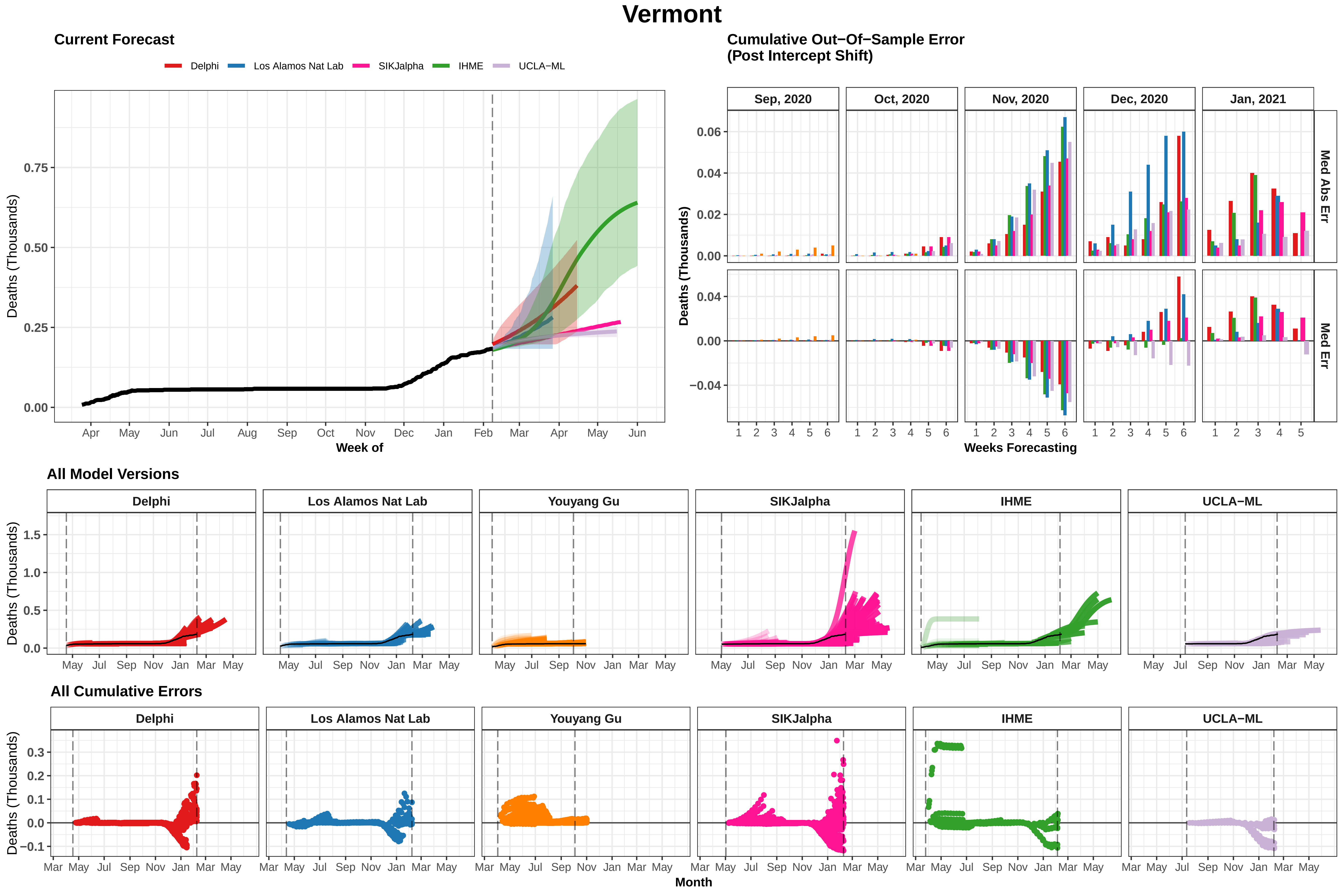
Vermont
Current Forecast
Delphi
Los Alamos Nat Lab
SIKJalpha
IHME
UCLA−ML
Deaths (Thousands)
Week of
Cumulative Out−Of−Sample Error
(Post Intercept Shift)
Sep, 2020
Oct, 2020
Nov, 2020
Dec, 2020
Jan, 2021
Med Abs Err
Med Err
Weeks Forecasting
All Model Versions
Youyang Gu
All Cumulative Errors
Month

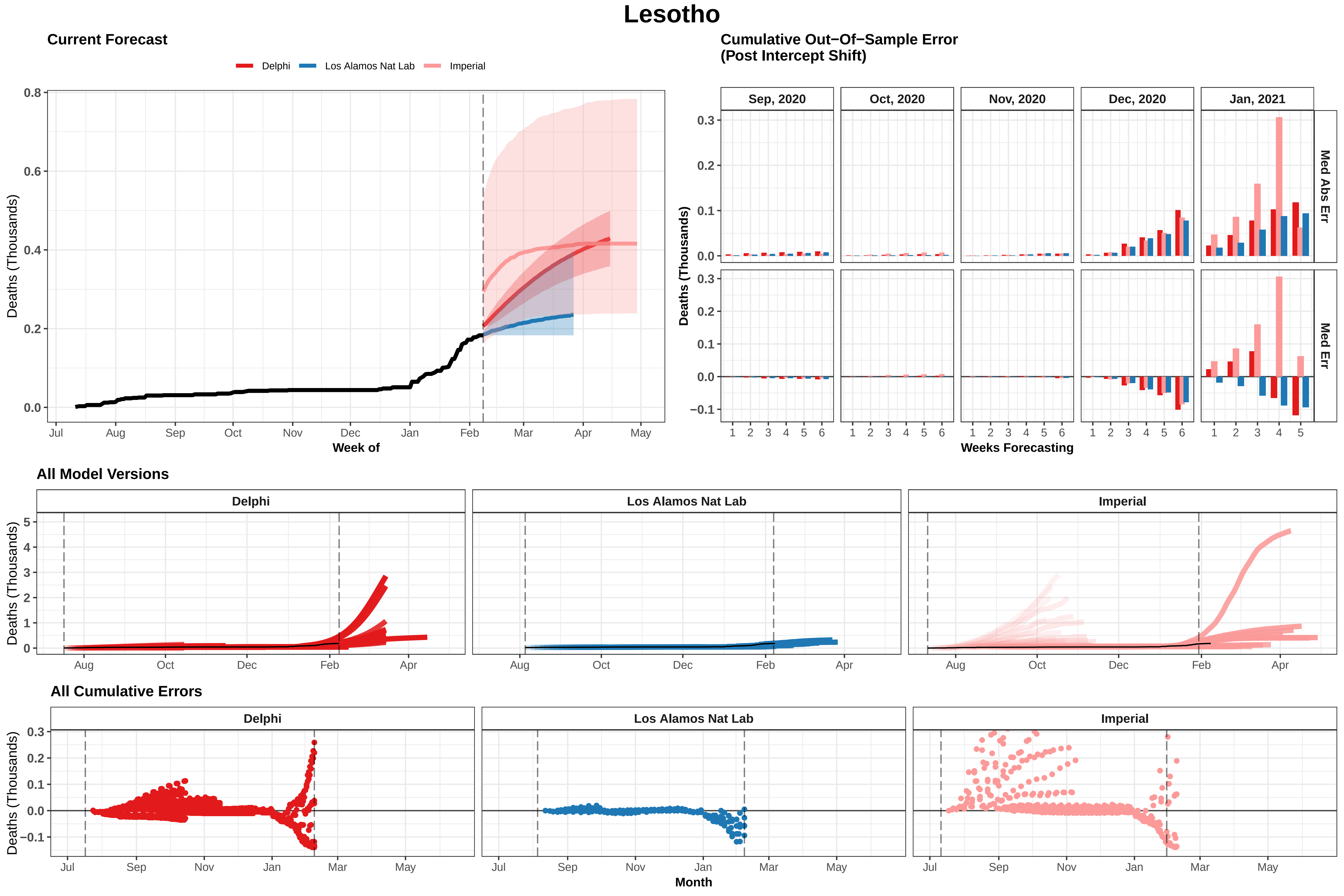
Lesotho
Current Forecast
Delphi
Los Alamos Nat Lab
Imperial
Deaths (Thousands)
Week of
Jul
Aug
Sep
Oct
Nov
Dec
Jan
Feb
Mar
Apr
May
Cumulative Out−Of−Sample Error
(Post Intercept Shift)
Sep, 2020
Oct, 2020
Nov, 2020
Dec, 2020
Jan, 2021
Med Abs Err
Med Err
Weeks Forecasting
All Model Versions
Delphi
Los Alamos Nat Lab
Imperial
All Cumulative Errors
Month

Guyana

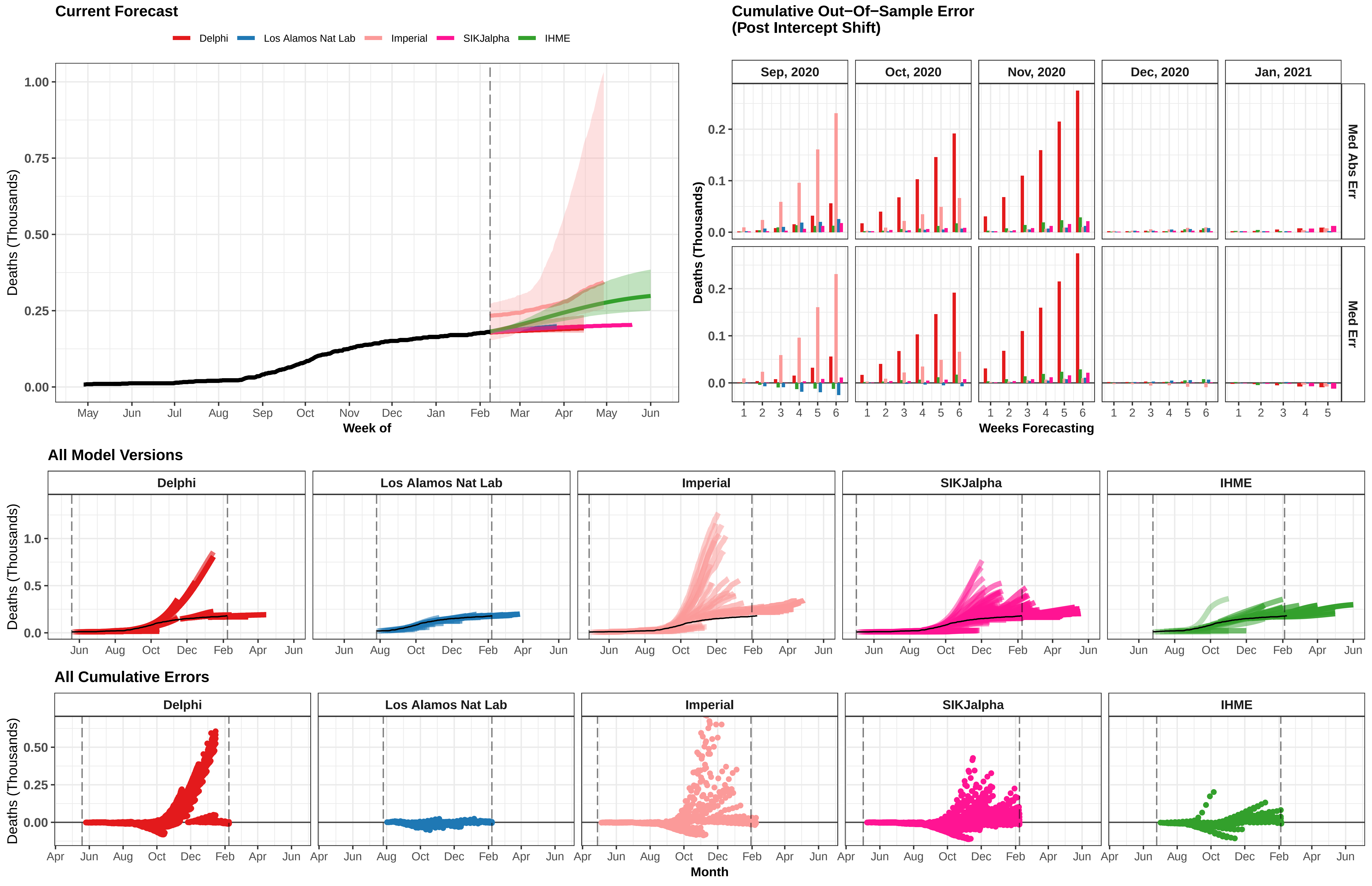

Current Forecast
Delphi
Los Alamos Nat Lab
Imperial
SIKJalpha
IHME
Deaths (Thousands)
Week of
May Jun Jul Aug Sep Oct Nov Dec Jan Feb Mar Apr May Jun
Cumulative Out−Of−Sample Error
(Post Intercept Shift)
Sep, 2020
Oct, 2020
Nov, 2020
Dec, 2020
Jan, 2021
Med Abs Err
Med Err
Weeks Forecasting
All Model Versions
All Cumulative Errors
Month

Botswana

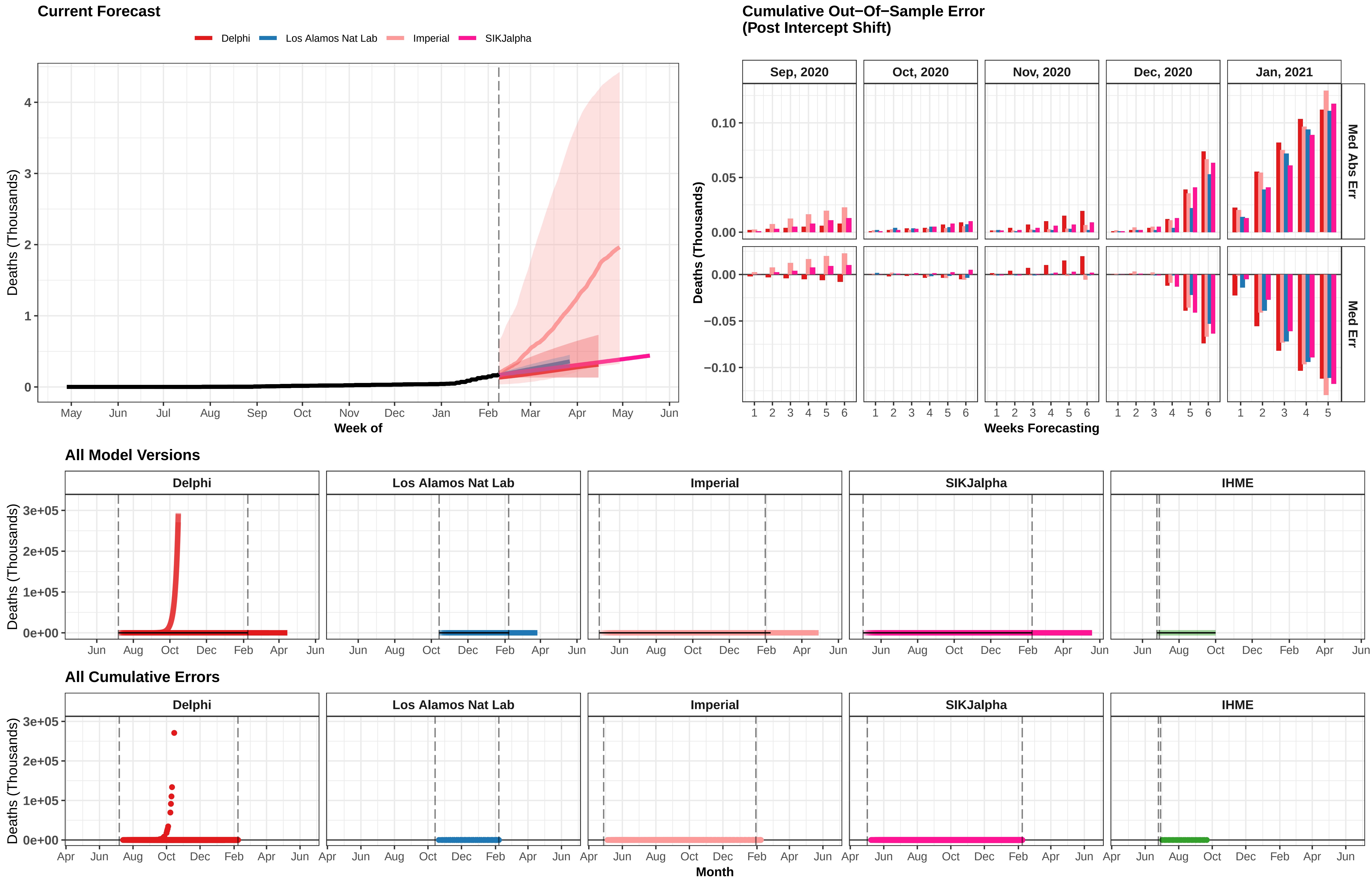

Current Forecast
Delphi
Los Alamos Nat Lab
Imperial
SIKJalpha
Deaths (Thousands)
Week of
May Jun Jul Aug Sep Oct Nov Dec Jan Feb Mar Apr May Jun
Cumulative Out−Of−Sample Error
(Post Intercept Shift)
Sep, 2020
Oct, 2020
Nov, 2020
Dec, 2020
Jan, 2021
Med Abs Err
Med Err
Weeks Forecasting
All Model Versions
Delphi
Los Alamos Nat Lab
Imperial
SIKJalpha
IHME
All Cumulative Errors
Month

Nicaragua

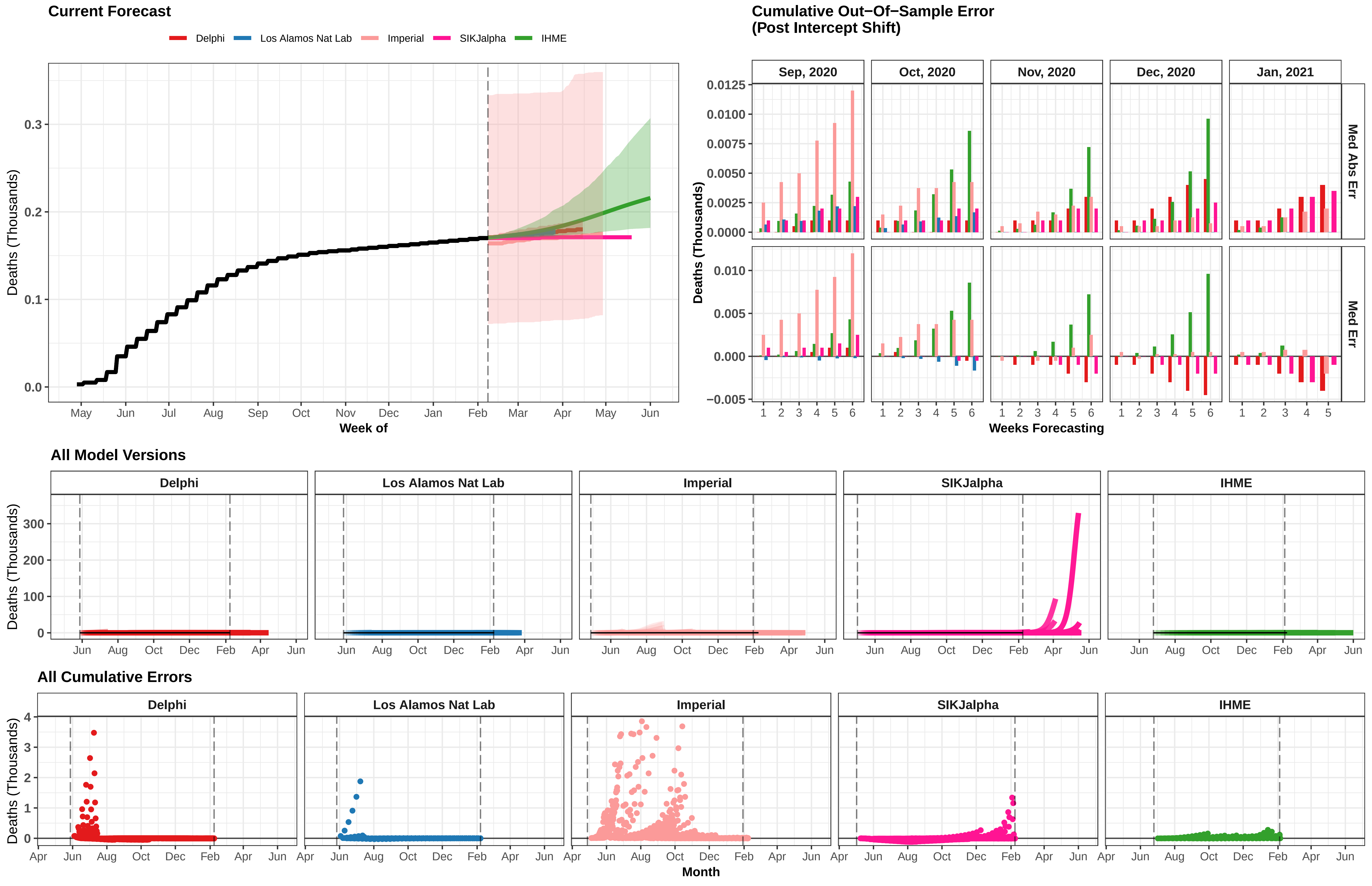
Current Forecast
Delphi
Los Alamos Nat Lab
Imperial
SIKJalpha
IHME
Deaths (Thousands)
Week of
Cumulative Out−Of−Sample Error
(Post Intercept Shift)
Sep, 2020
Oct, 2020
Nov, 2020
Dec, 2020
Jan, 2021
Med Abs Err
Med Err
Weeks Forecasting
All Model Versions
All Cumulative Errors
Month

Niger

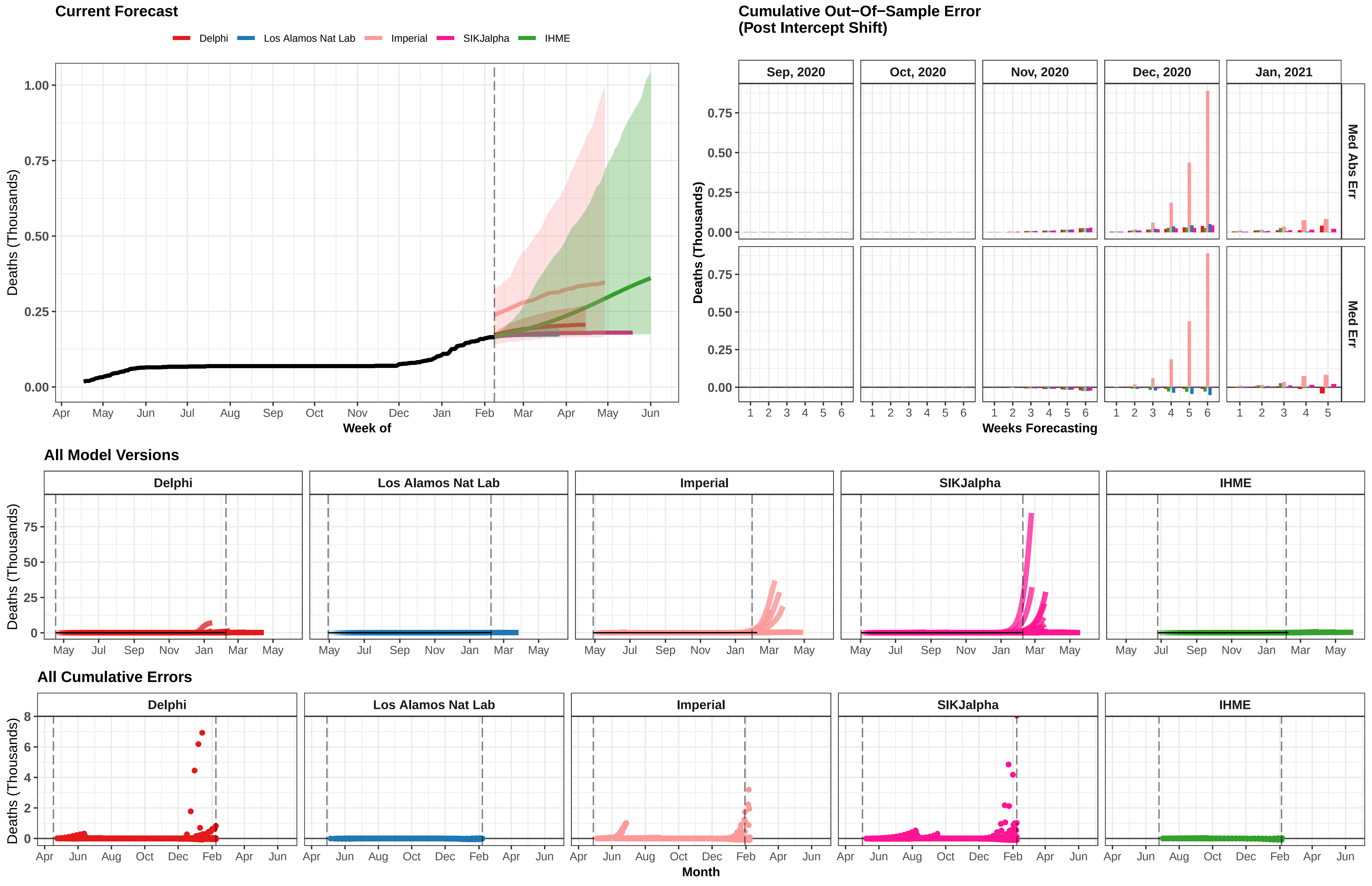

Current Forecast
Delphi
Los Alamos Nat Lab
Imperial
SIKJalpha
IHME
Deaths (Thousands)
Week of
Cumulative Out−Of−Sample Error
(Post Intercept Shift)
Sep, 2020
Oct, 2020
Nov, 2020
Dec, 2020
Jan, 2021
Med Abs Err
Med Err
Weeks Forecasting
All Model Versions
All Cumulative Errors
Month

Cote d'Ivoire

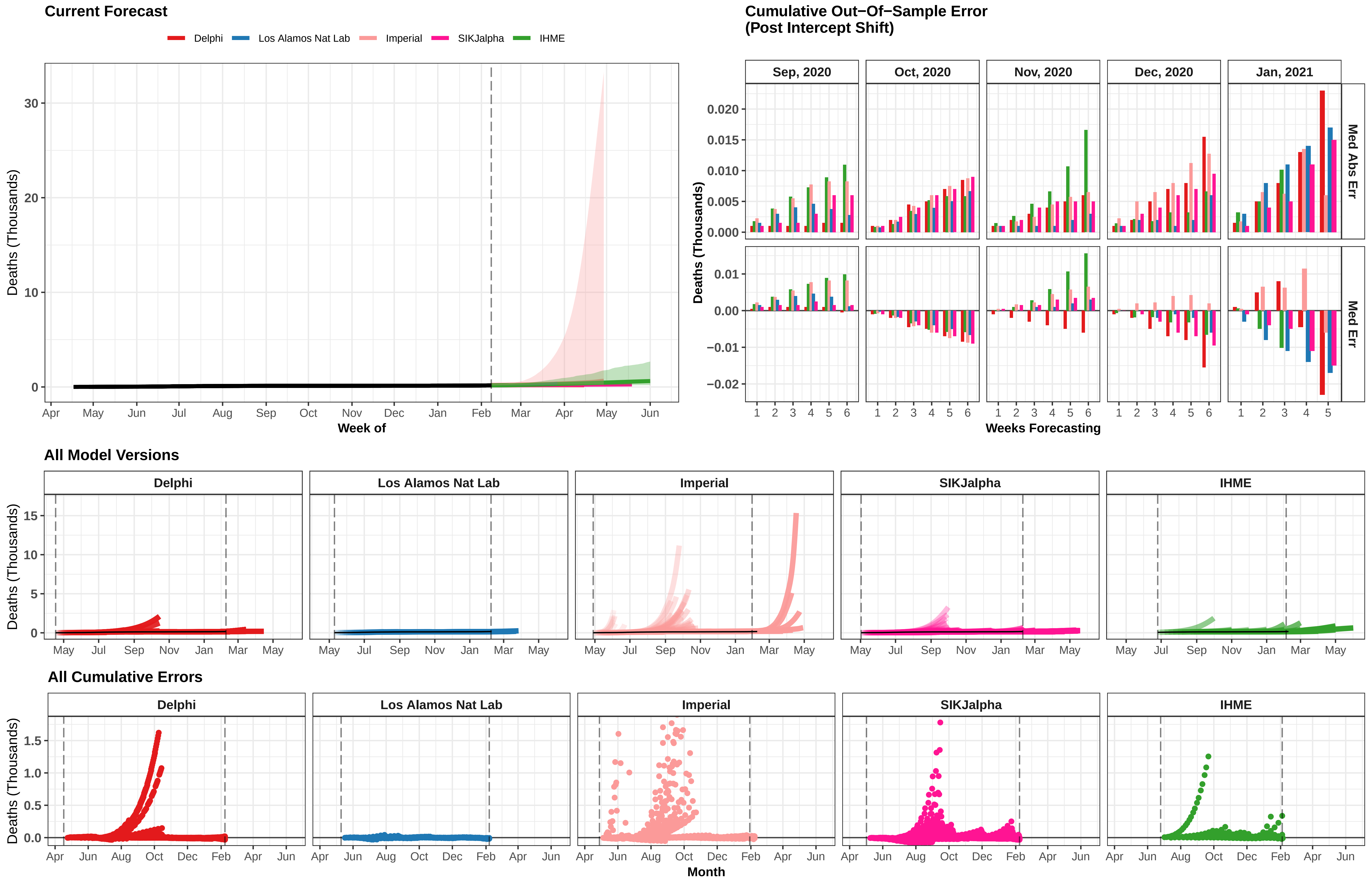

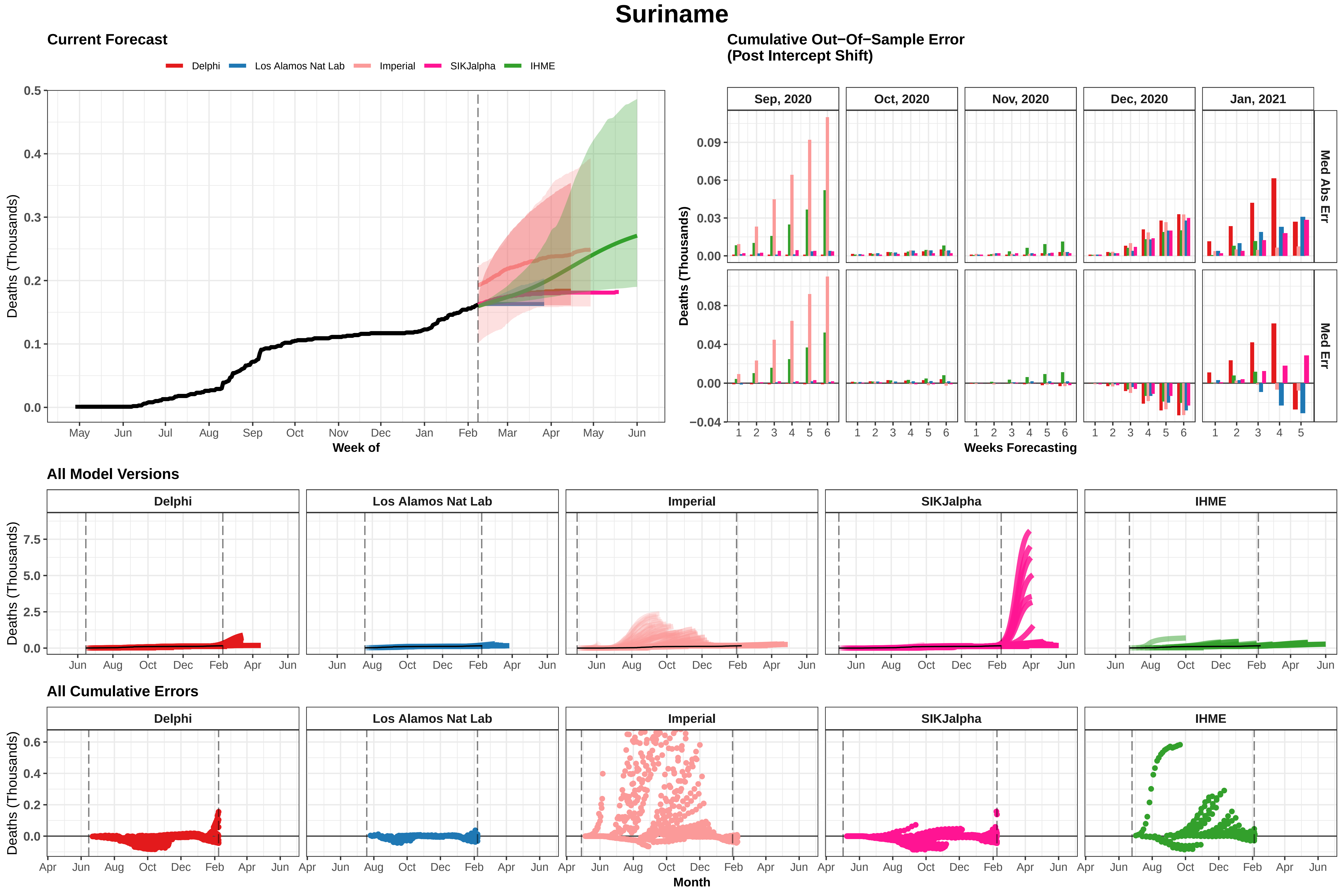

Suriname
Current Forecast
Delphi
Los Alamos Nat Lab
Imperial
SIKJalpha
IHME
Deaths (Thousands)
Week of
Cumulative Out−Of−Sample Error
(Post Intercept Shift)
Sep, 2020
Oct, 2020
Nov, 2020
Dec, 2020
Jan, 2021
Med Abs Err
Med Err
Weeks Forecasting
All Model Versions
All Cumulative Errors
Month

Cape Verde

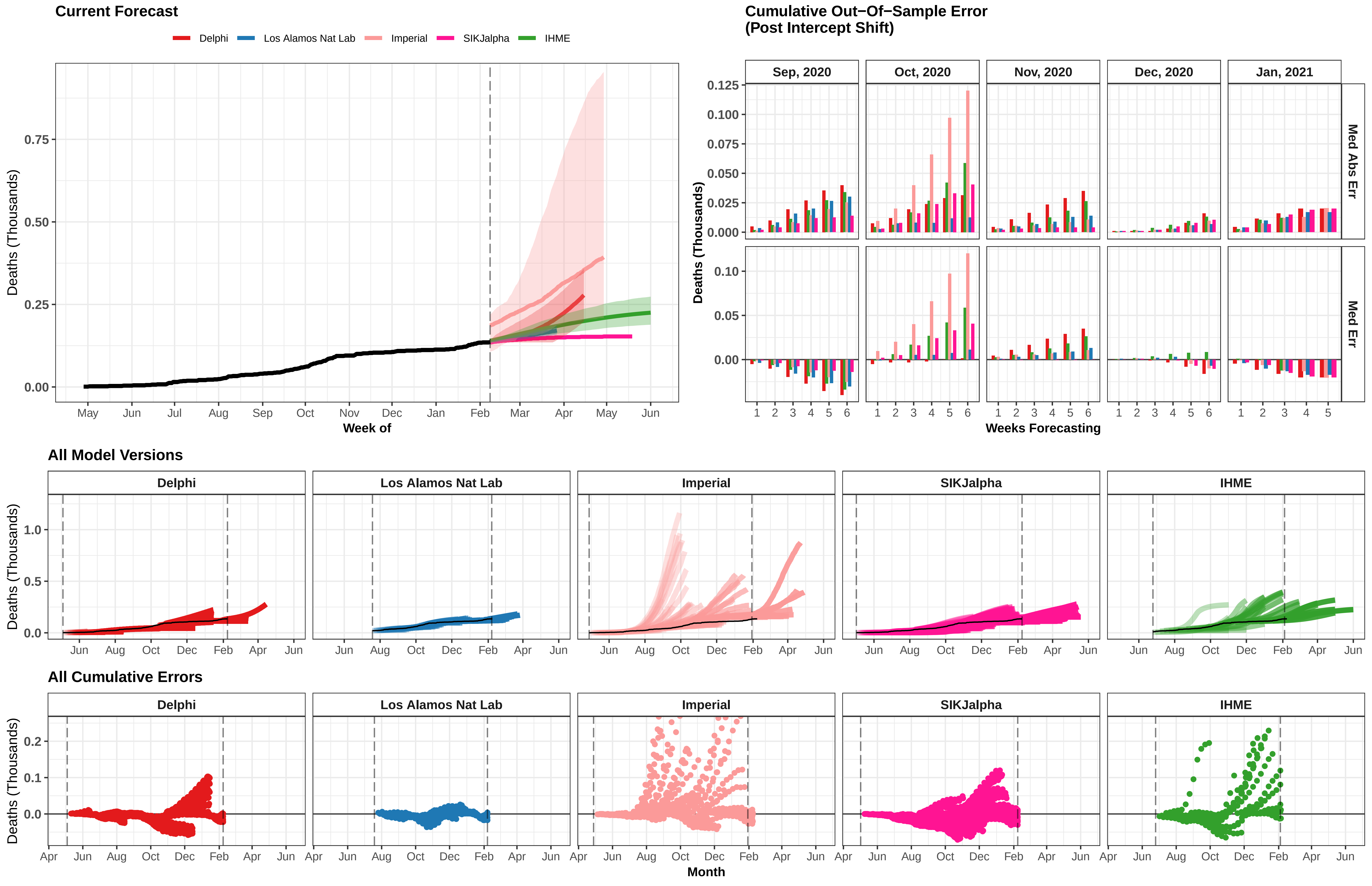

Current Forecast
Delphi
Los Alamos Nat Lab
Imperial
SIKJalpha
IHME
Deaths (Thousands)
Week of
Cumulative Out−Of−Sample Error
(Post Intercept Shift)
Sep, 2020
Oct, 2020
Nov, 2020
Dec, 2020
Jan, 2021
Med Abs Err
Med Err
Weeks Forecasting
All Model Versions
All Cumulative Errors
Month

Trinidad and Tobago

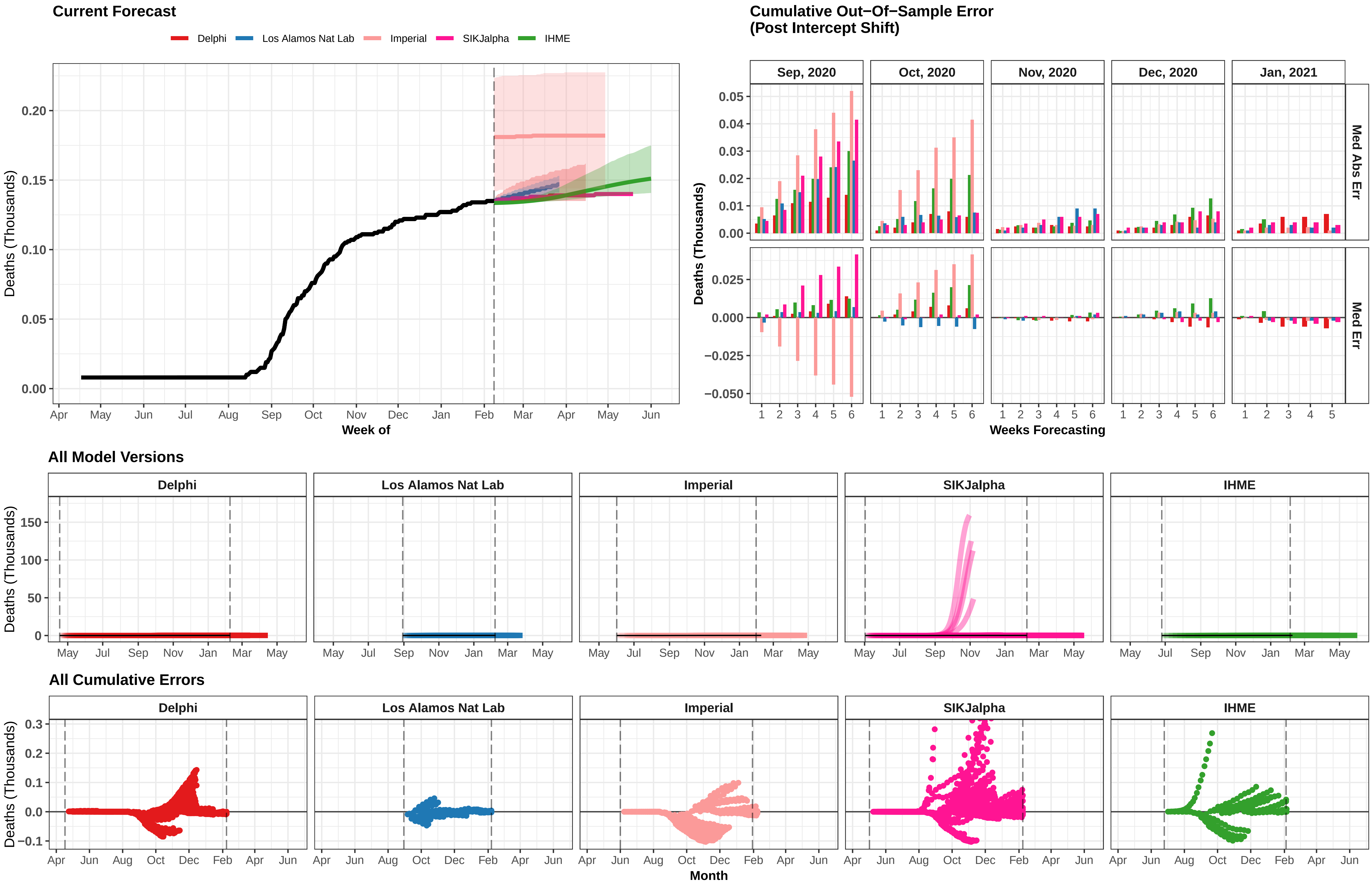

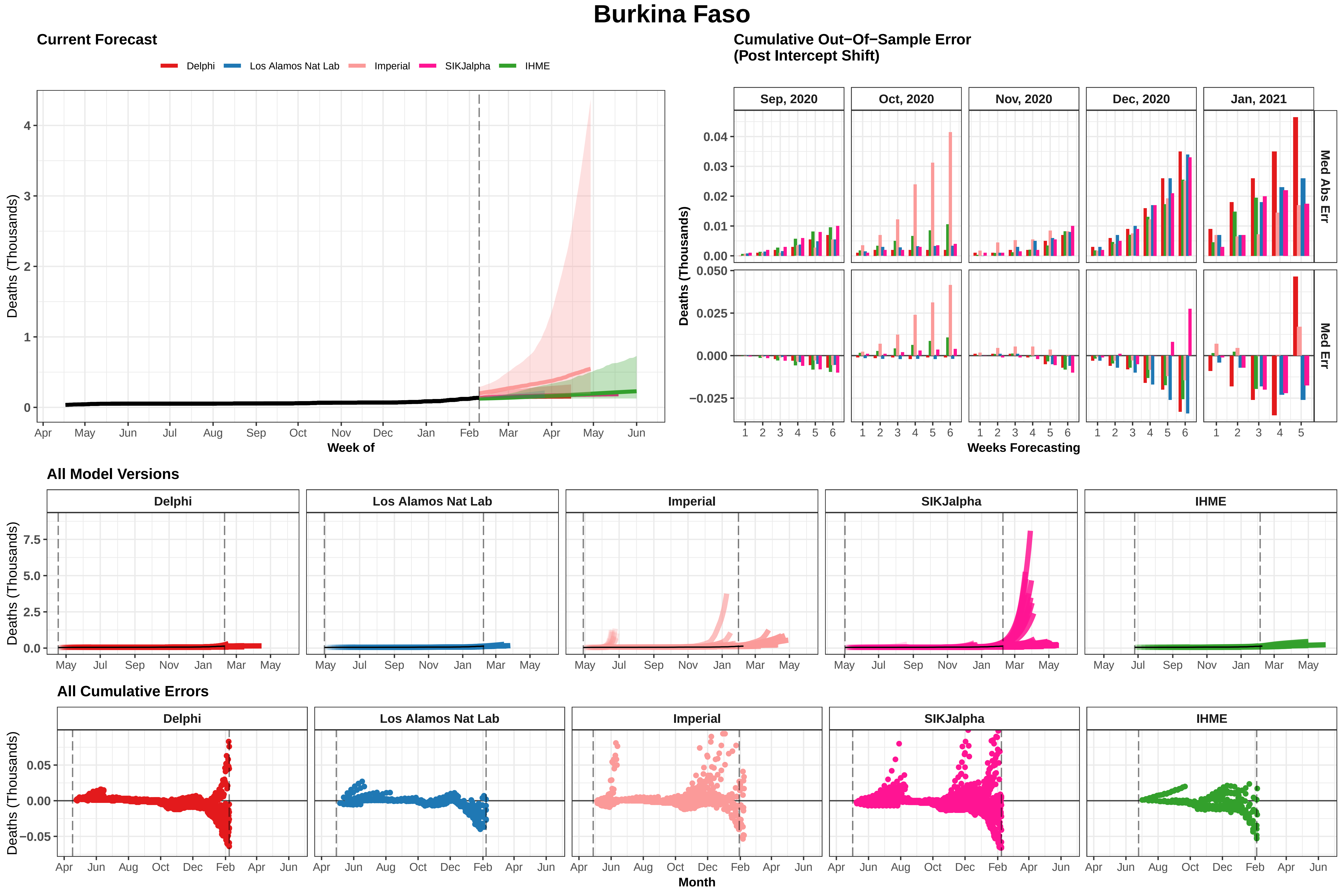

Burkina Faso
Current Forecast
Delphi
Los Alamos Nat Lab
Imperial
SIKJalpha
IHME
Deaths (Thousands)
Week of
Cumulative Out−Of−Sample Error
(Post Intercept Shift)
Sep, 2020
Oct, 2020
Nov, 2020
Dec, 2020
Jan, 2021
Med Abs Err
Med Err
Weeks Forecasting
All Model Versions
All Cumulative Errors
Month

Somalia

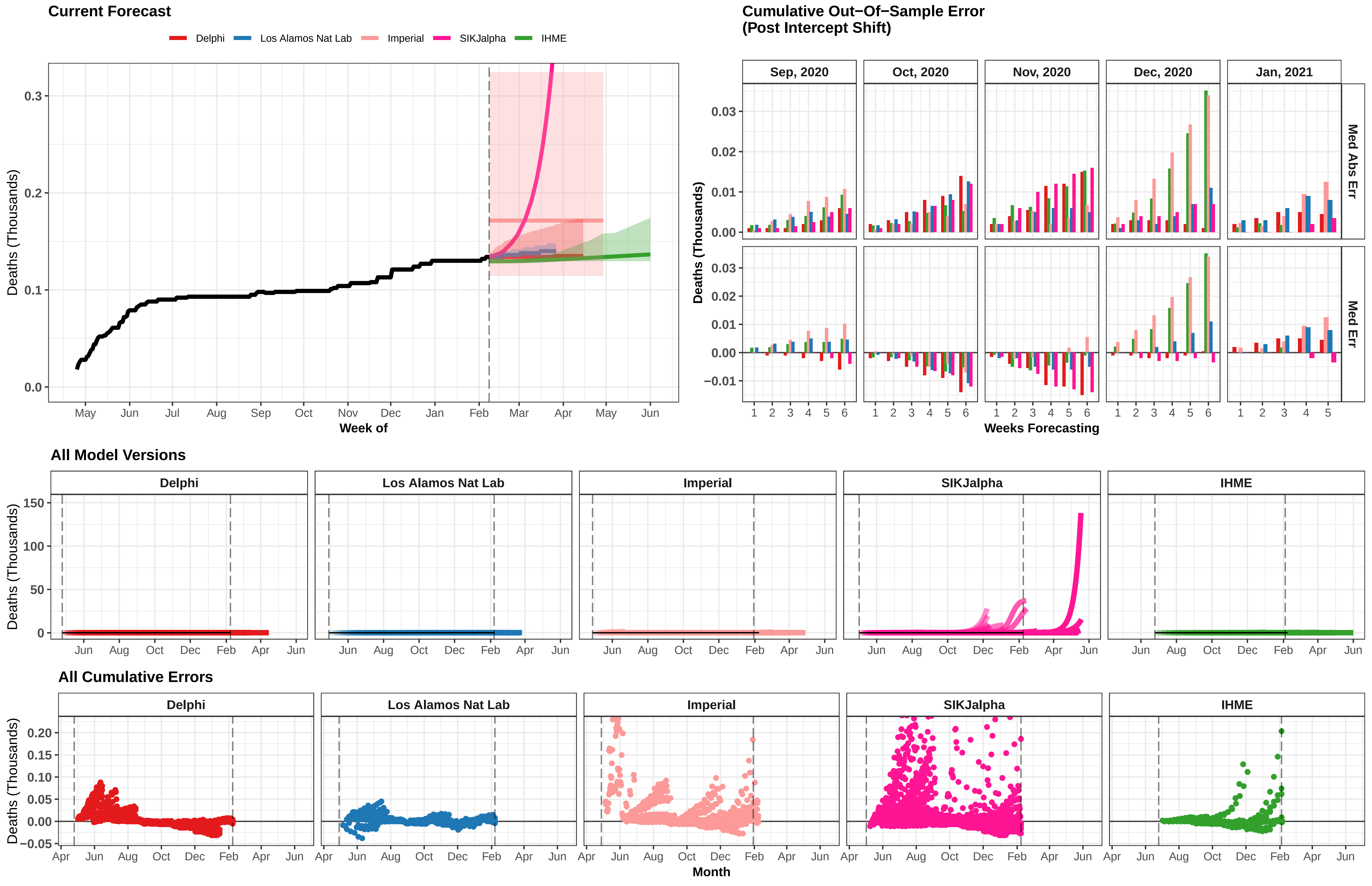

Current Forecast
Delphi
Los Alamos Nat Lab
Imperial
SIKJalpha
IHME
Deaths (Thousands)
Week of
Cumulative Out−Of−Sample Error
(Post Intercept Shift)
Sep, 2020
Oct, 2020
Nov, 2020
Dec, 2020
Jan, 2021
Med Abs Err
Med Err
Weeks Forecasting
All Model Versions
All Cumulative Errors
Month

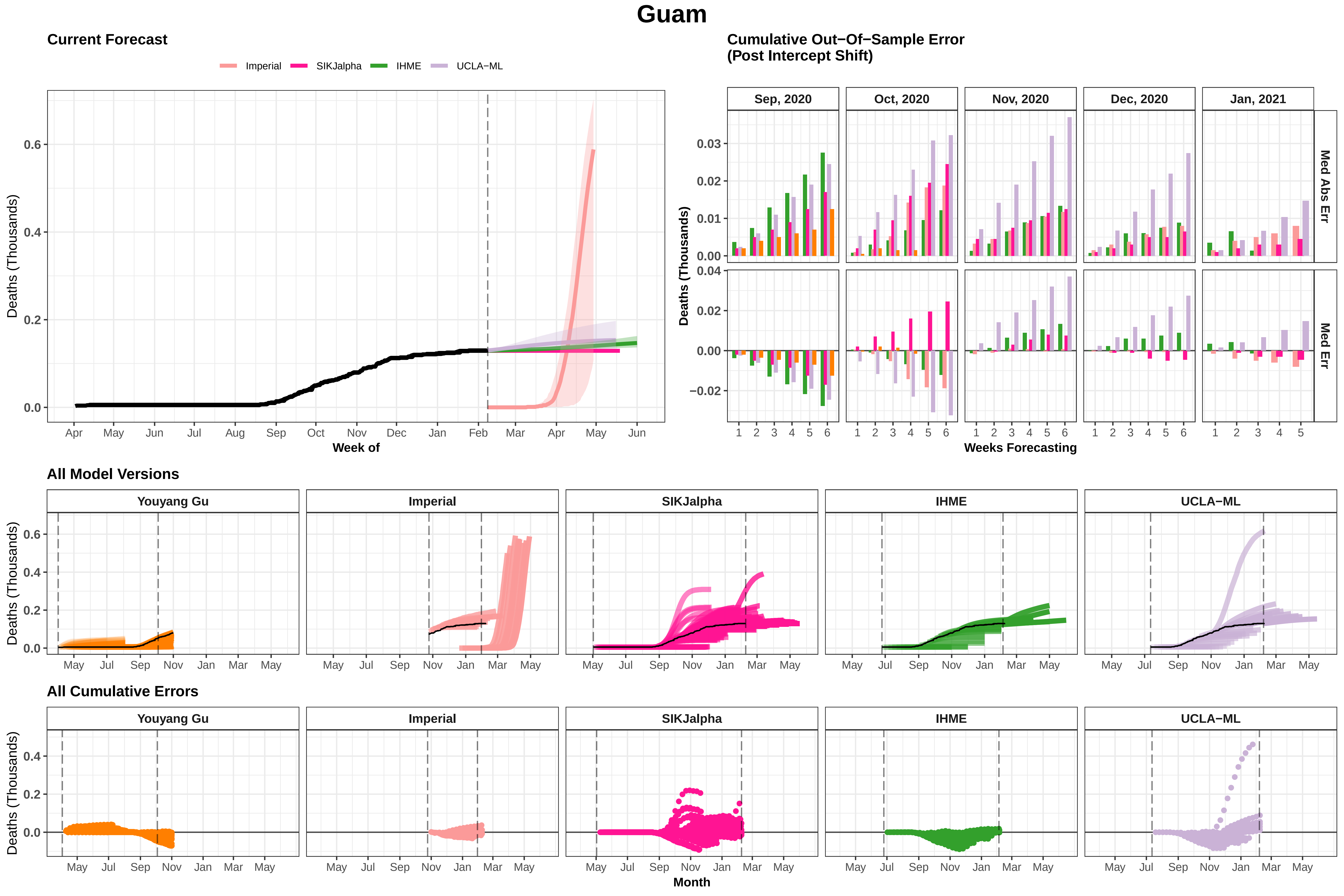
Guam
Current Forecast
Imperial
SIKJalpha
IHME
UCLA−ML
Deaths (Thousands)
0.0
0.2
0.4
0.6
Apr
May
Jun
Jul
Aug
Sep
Oct
Nov
Dec
Jan
Feb
Mar
Apr
May
Jun
Week of
Cumulative Out−Of−Sample Error
(Post Intercept Shift)
Sep, 2020
Oct, 2020
Nov, 2020
Dec, 2020
Jan, 2021
Med Abs Err
Med Err
0.00
0.01
0.02
0.03
0.04
−0.02
Weeks Forecasting
All Model Versions
Youyang Gu
Imperial
SIKJalpha
IHME
UCLA−ML
All Cumulative Errors
Month

Chad

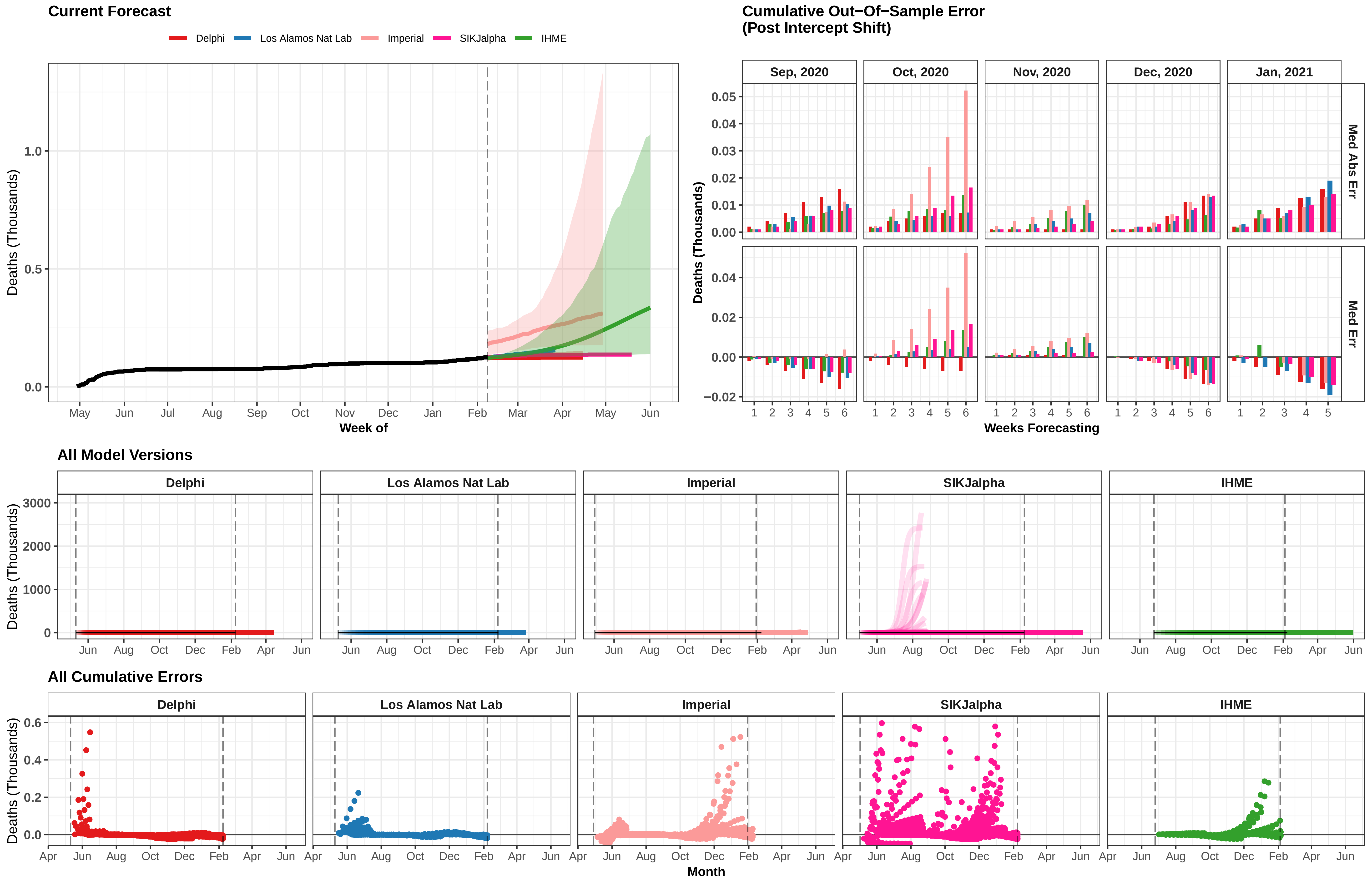

Current Forecast
Delphi
Los Alamos Nat Lab
Imperial
SIKJalpha
IHME
Deaths (Thousands)
0.0
0.5
1.0
May
Jun
Jul
Aug
Sep
Oct
Nov
Dec
Jan
Feb
Mar
Apr
May
Jun
Week of
Cumulative Out−Of−Sample Error
(Post Intercept Shift)
Sep, 2020
Oct, 2020
Nov, 2020
Dec, 2020
Jan, 2021
Med Abs Err
Med Err
Deaths (Thousands)
0.00
0.01
0.02
0.03
0.04
0.05
−0.02
0.00
0.02
0.04
1
2
3
4
5
6
Weeks Forecasting
All Model Versions
Delphi
Los Alamos Nat Lab
Imperial
SIKJalpha
IHME
Deaths (Thousands)
0
1000
2000
3000
Jun
Aug
Oct
Dec
Feb
Apr
Jun
All Cumulative Errors
Delphi
Los Alamos Nat Lab
Imperial
SIKJalpha
IHME
Deaths (Thousands)
0.0
0.2
0.4
0.6
Apr
Jun
Aug
Oct
Dec
Feb
Apr
Jun
Month

Congo

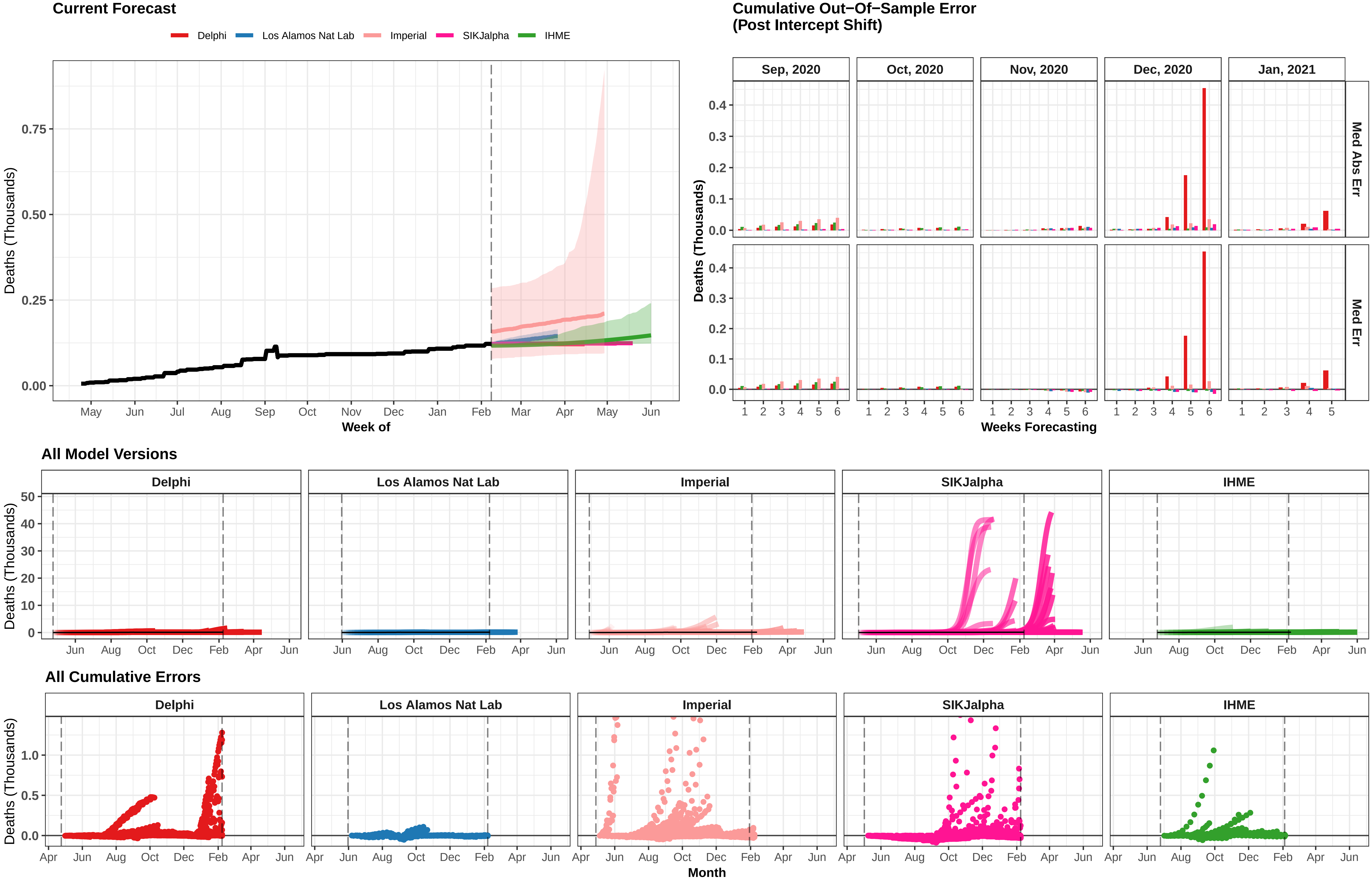

Current Forecast
Delphi
Los Alamos Nat Lab
Imperial
SIKJalpha
IHME
Deaths (Thousands)
Week of
Cumulative Out−Of−Sample Error
(Post Intercept Shift)
Sep, 2020
Oct, 2020
Nov, 2020
Dec, 2020
Jan, 2021
Med Abs Err
Med Err
Weeks Forecasting
All Model Versions
All Cumulative Errors
Month

Comoros

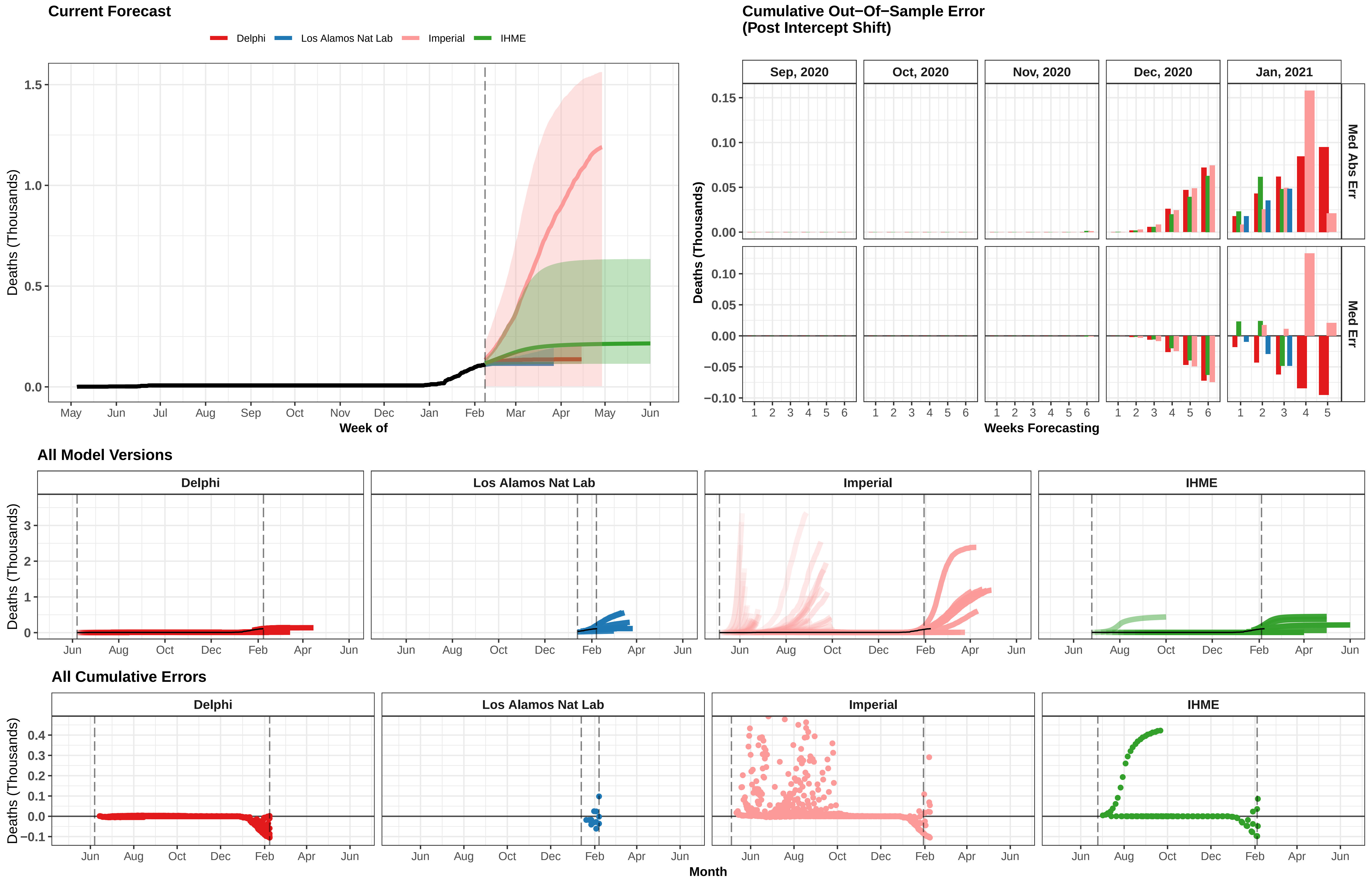
Current Forecast
Delphi
Los Alamos Nat Lab
Imperial
IHME
Deaths (Thousands)
Week of
Cumulative Out−Of−Sample Error
(Post Intercept Shift)
Sep, 2020
Oct, 2020
Nov, 2020
Dec, 2020
Jan, 2021
Med Abs Err
Med Err
Weeks Forecasting
All Model Versions
All Cumulative Errors
Month

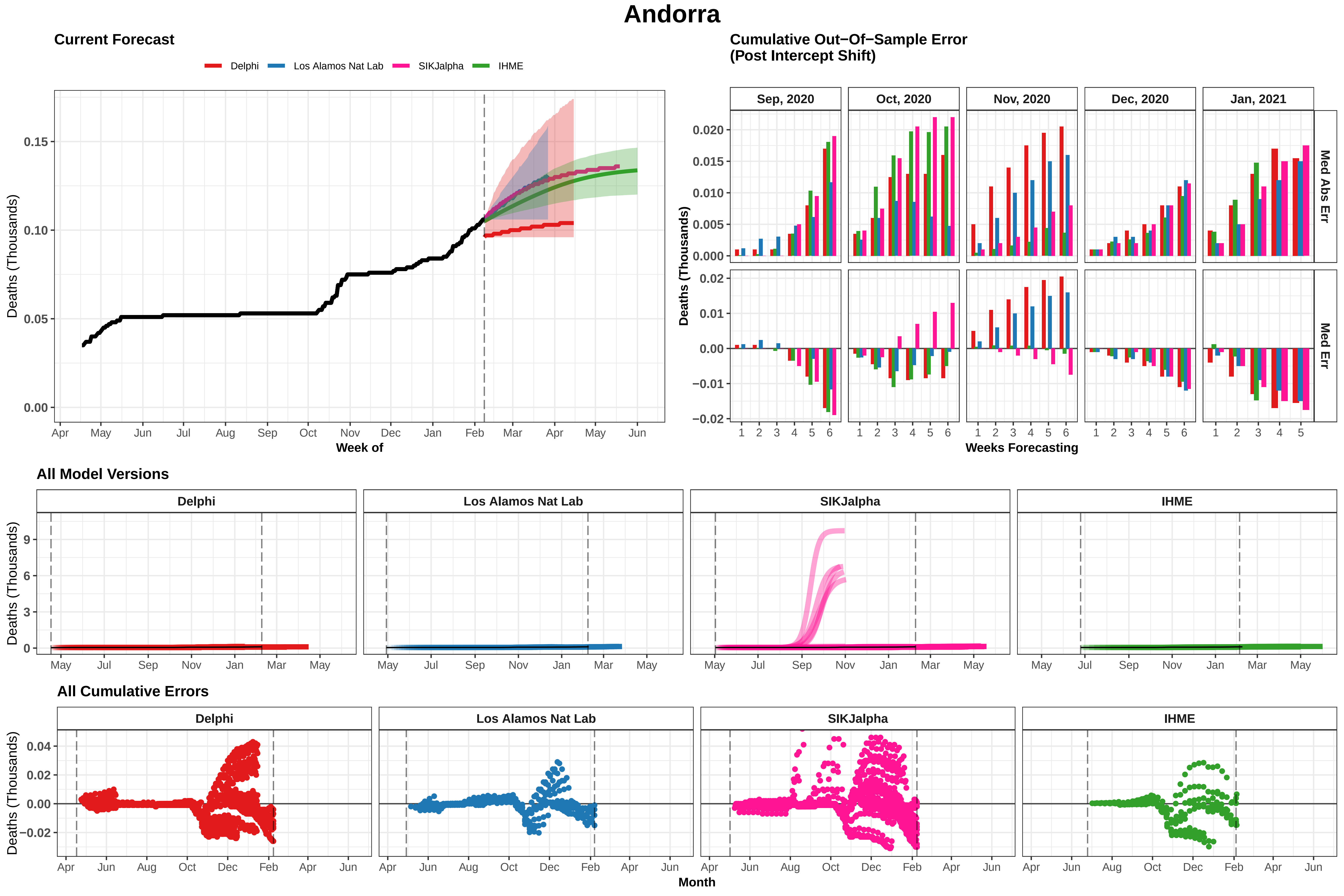

Andorra
Current Forecast
Delphi
Los Alamos Nat Lab
SIKJalpha
IHME
Deaths (Thousands)
Week of
Cumulative Out−Of−Sample Error
(Post Intercept Shift)
Sep, 2020
Oct, 2020
Nov, 2020
Dec, 2020
Jan, 2021
Med Abs Err
Med Err
Weeks Forecasting
All Model Versions
All Cumulative Errors
Month

Tajikistan

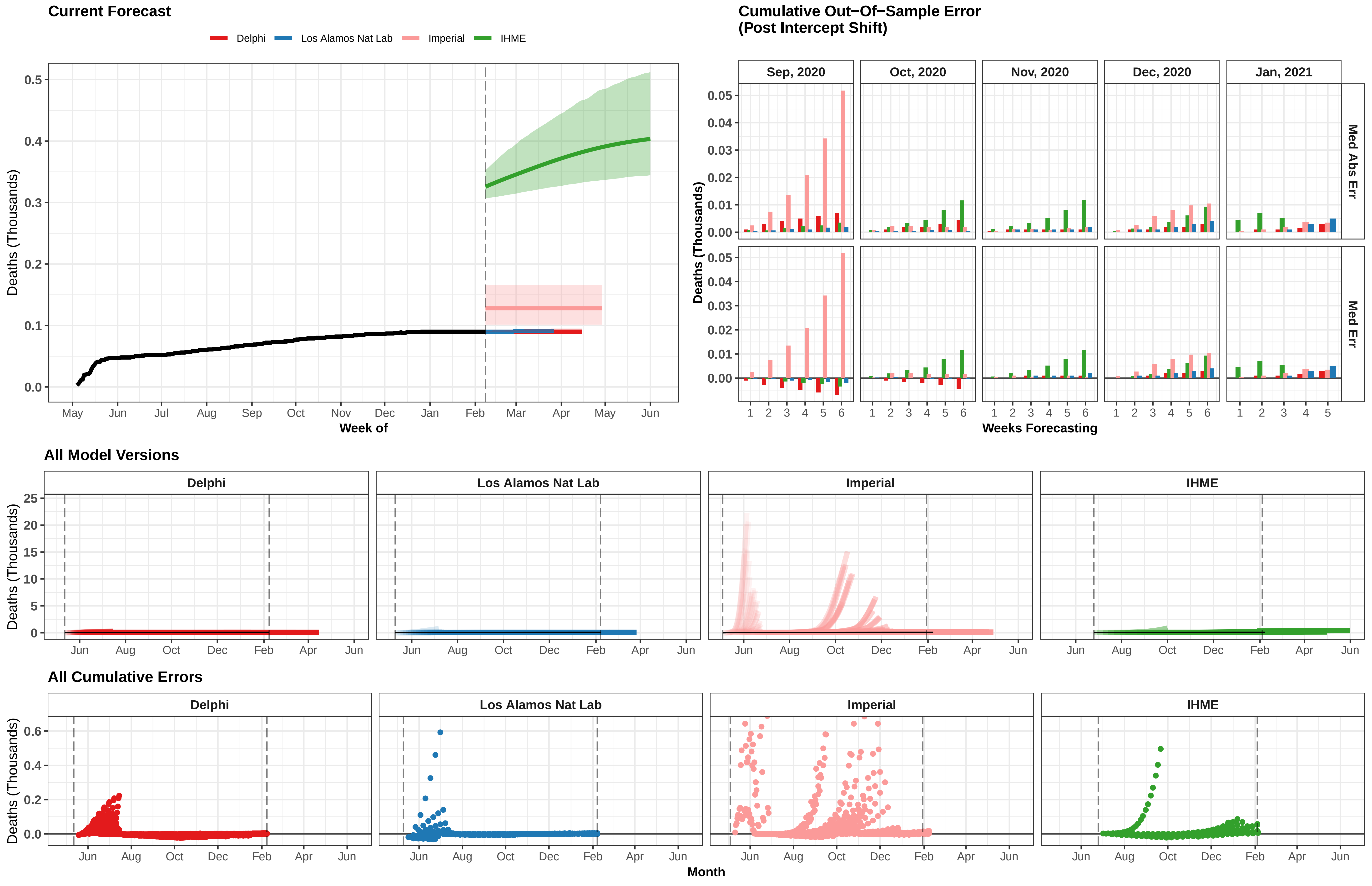
Current Forecast
Delphi
Los Alamos Nat Lab
Imperial
IHME
Deaths (Thousands)
Week of
Cumulative Out−Of−Sample Error
(Post Intercept Shift)
Sep, 2020
Oct, 2020
Nov, 2020
Dec, 2020
Jan, 2021
Med Abs Err
Med Err
Weeks Forecasting
All Model Versions
All Cumulative Errors
Month

Equatorial Guinea

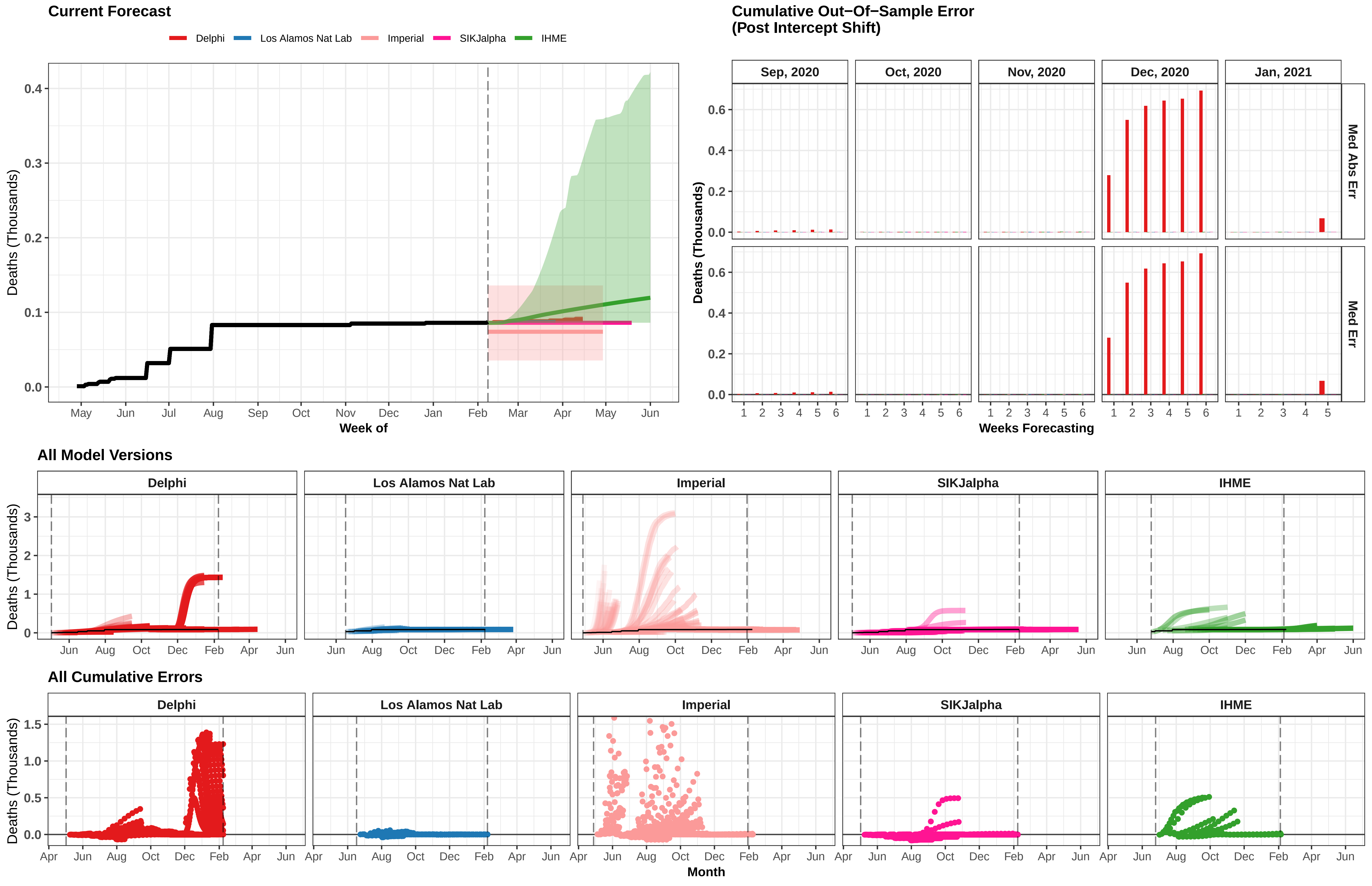

Guinea

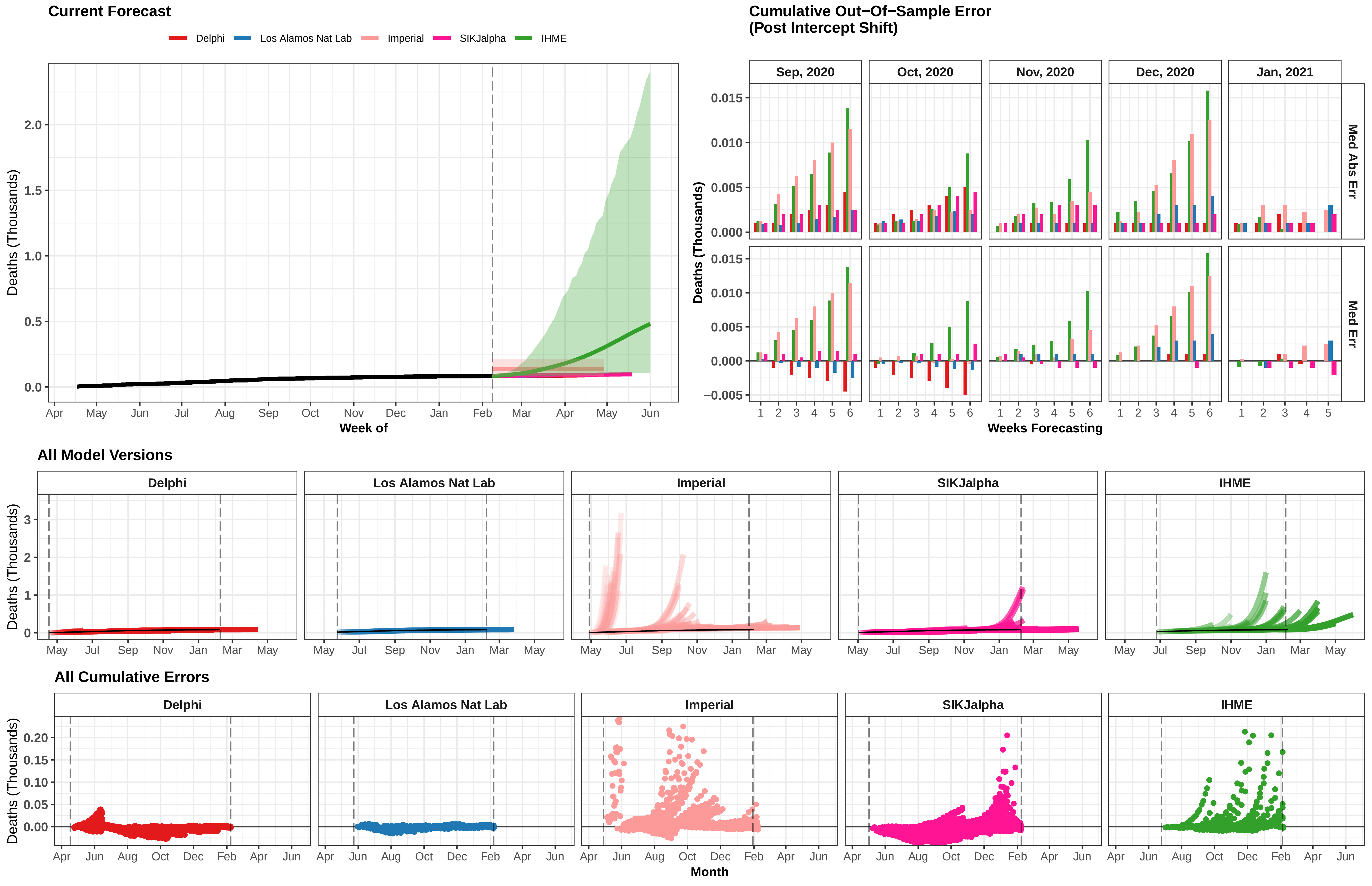

Current Forecast
Delphi
Los Alamos Nat Lab
Imperial
SIKJalpha
IHME
Deaths (Thousands)
Week of
Cumulative Out−Of−Sample Error
(Post Intercept Shift)
Sep, 2020
Oct, 2020
Nov, 2020
Dec, 2020
Jan, 2021
Med Abs Err
Med Err
Weeks Forecasting
All Model Versions
All Cumulative Errors
Month

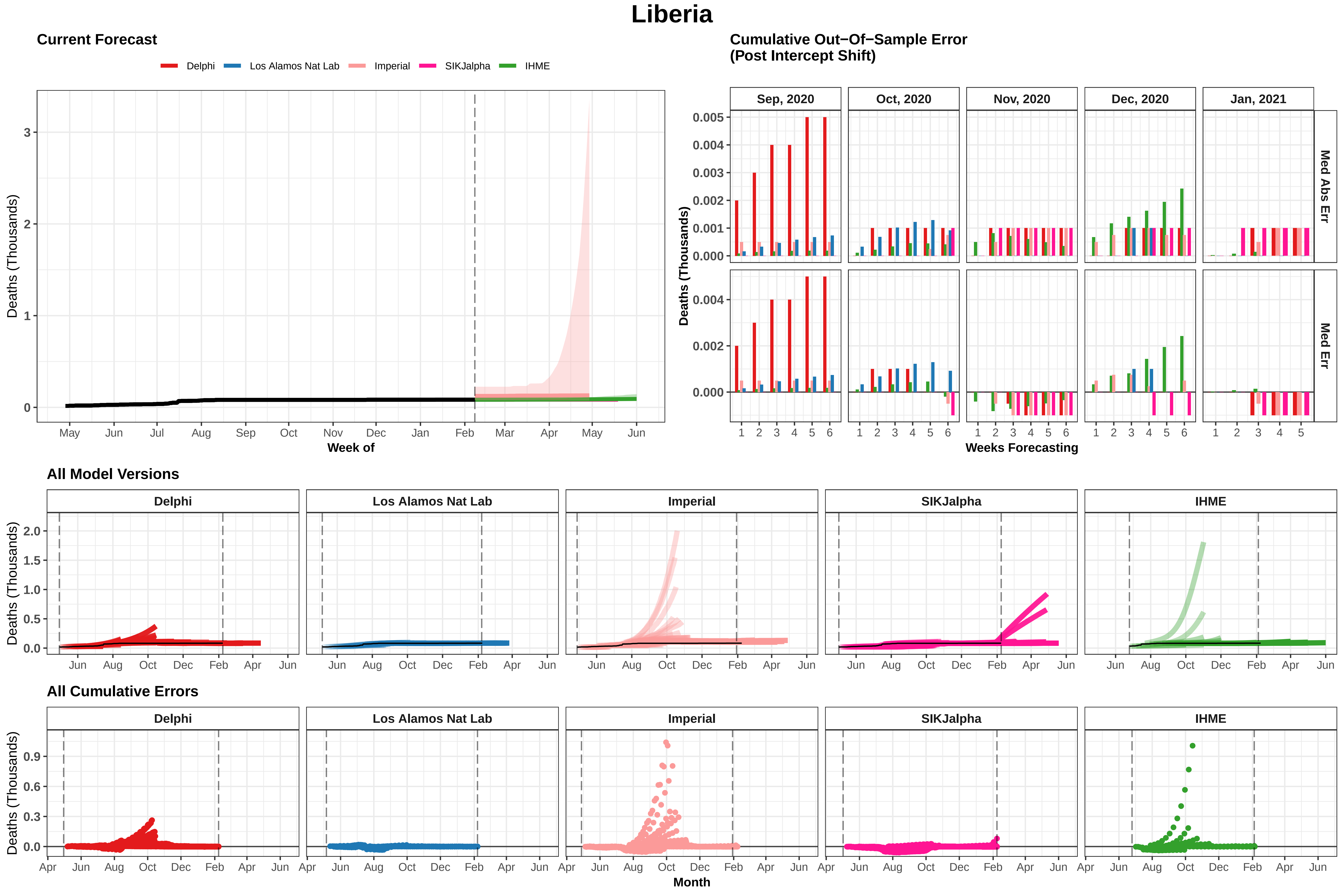
Liberia
Current Forecast
Delphi
Los Alamos Nat Lab
Imperial
SIKJalpha
IHME
Deaths (Thousands)
Week of
May
Jun
Jul
Aug
Sep
Oct
Nov
Dec
Jan
Feb
Mar
Apr
Cumulative Out−Of−Sample Error
(Post Intercept Shift)
Sep, 2020
Oct, 2020
Nov, 2020
Dec, 2020
Jan, 2021
Med Abs Err
Med Err
Weeks Forecasting
All Model Versions
All Cumulative Errors
Month

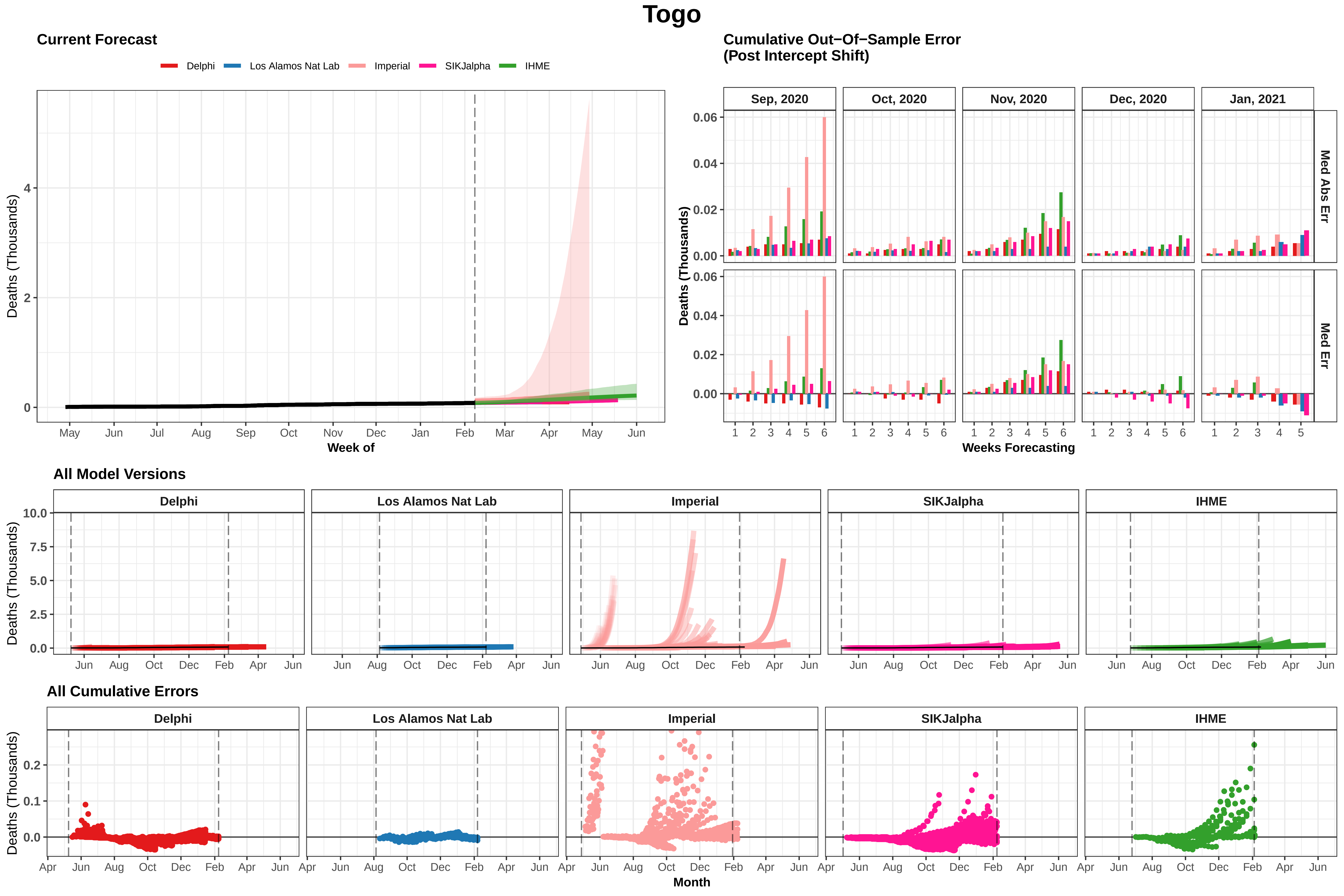

Togo
Current Forecast
Delphi
Los Alamos Nat Lab
Imperial
SIKJalpha
IHME
Deaths (Thousands)
Week of
Cumulative Out−Of−Sample Error
(Post Intercept Shift)
Sep, 2020
Oct, 2020
Nov, 2020
Dec, 2020
Jan, 2021
Med Abs Err
Med Err
Weeks Forecasting
All Model Versions
All Cumulative Errors
Month

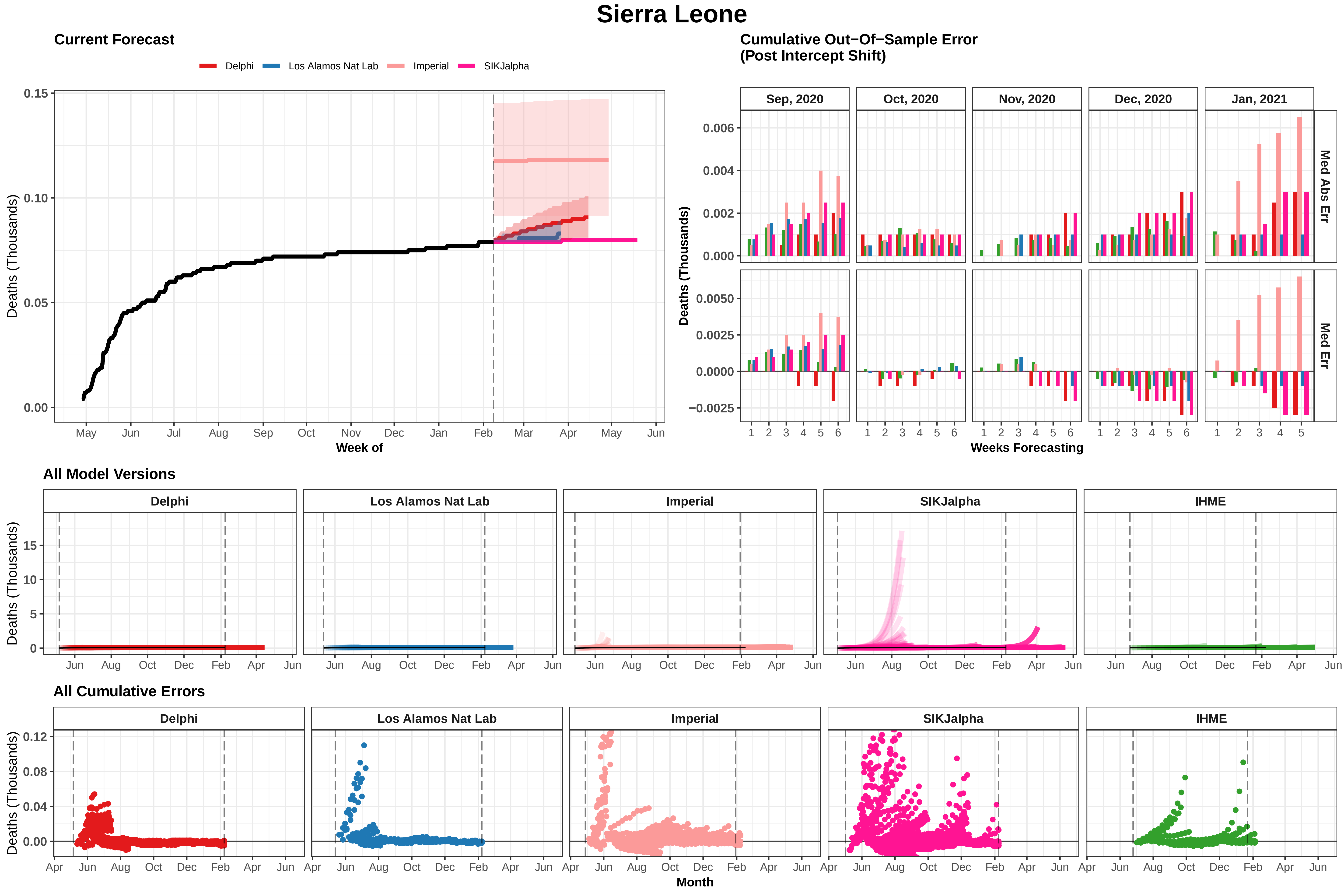
Sierra Leone
Current Forecast
Delphi
Los Alamos Nat Lab
Imperial
SIKJalpha
Deaths (Thousands)
Week of
Cumulative Out−Of−Sample Error
(Post Intercept Shift)
Sep, 2020
Oct, 2020
Nov, 2020
Dec, 2020
Jan, 2021
Med Abs Err
Med Err
Weeks Forecasting
All Model Versions
IHME
All Cumulative Errors
Month

Thailand

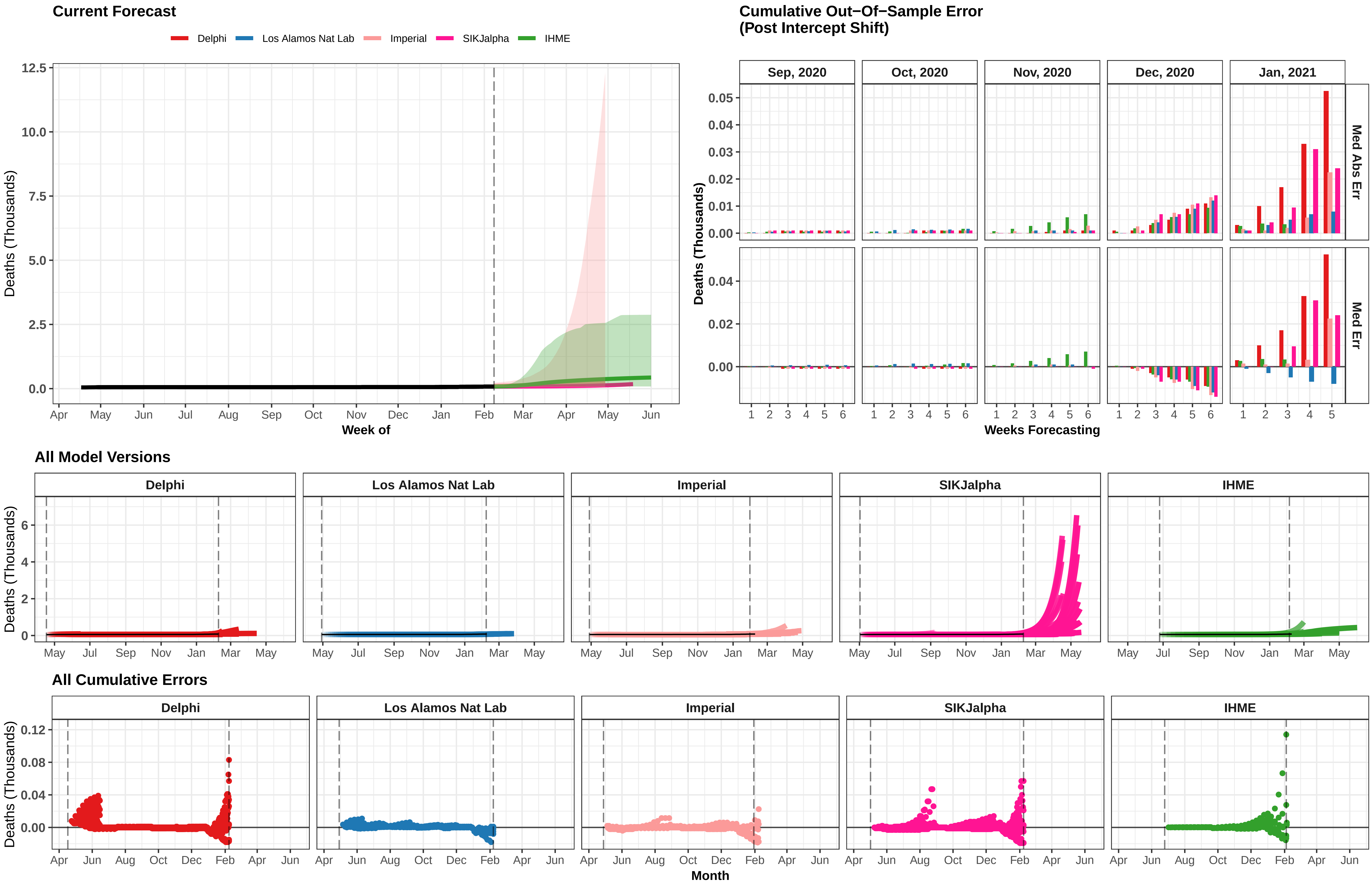
Current Forecast
Delphi
Los Alamos Nat Lab
Imperial
SIKJalpha
IHME
Deaths (Thousands)
Week of
Cumulative Out−Of−Sample Error
(Post Intercept Shift)
Sep, 2020
Oct, 2020
Nov, 2020
Dec, 2020
Jan, 2021
Med Abs Err
Med Err
Weeks Forecasting
All Model Versions
All Cumulative Errors
Month

Gabon

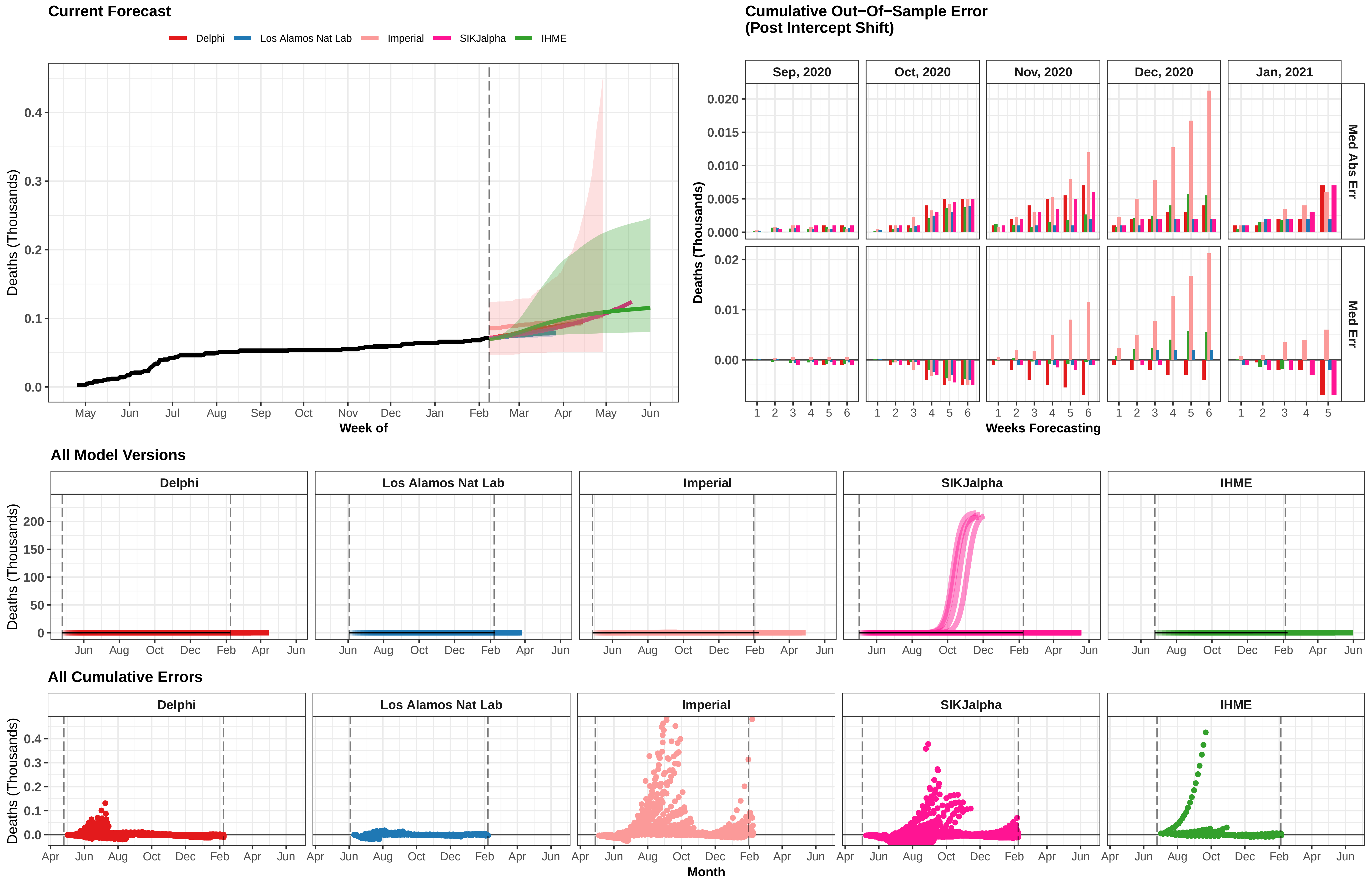

Current Forecast
Delphi
Los Alamos Nat Lab
Imperial
SIKJalpha
IHME
Deaths (Thousands)
Week of
Cumulative Out−Of−Sample Error (Post Intercept Shift)
Sep, 2020
Oct, 2020
Nov, 2020
Dec, 2020
Jan, 2021
Med Abs Err
Med Err
Weeks Forecasting
All Model Versions
All Cumulative Errors
Month

South Sudan

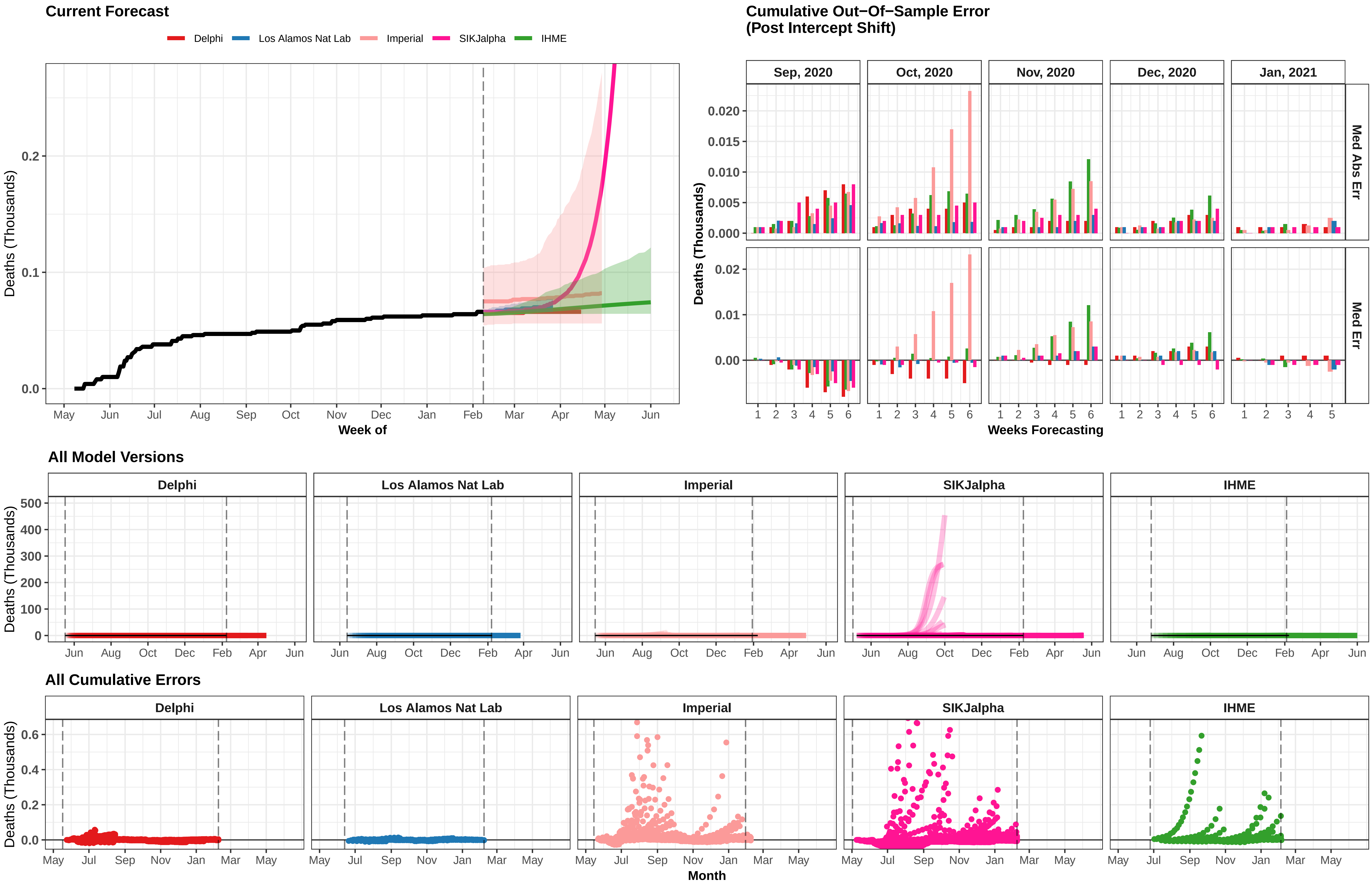

Current Forecast
Delphi
Los Alamos Nat Lab
Imperial
SIKJalpha
IHME
Deaths (Thousands)
0.0
0.1
0.2
May
Jun
Jul
Aug
Sep
Oct
Nov
Dec
Jan
Feb
Mar
Apr
May
Jun
Week of
Cumulative Out−Of−Sample Error
(Post Intercept Shift)
Sep, 2020
Oct, 2020
Nov, 2020
Dec, 2020
Jan, 2021
Med Abs Err
Med Err
0.000
0.005
0.010
0.015
0.020
0.00
0.01
0.02
Weeks Forecasting
All Model Versions
0
100
200
300
400
500
All Cumulative Errors
0.0
0.2
0.4
0.6
Month

Central African Republic

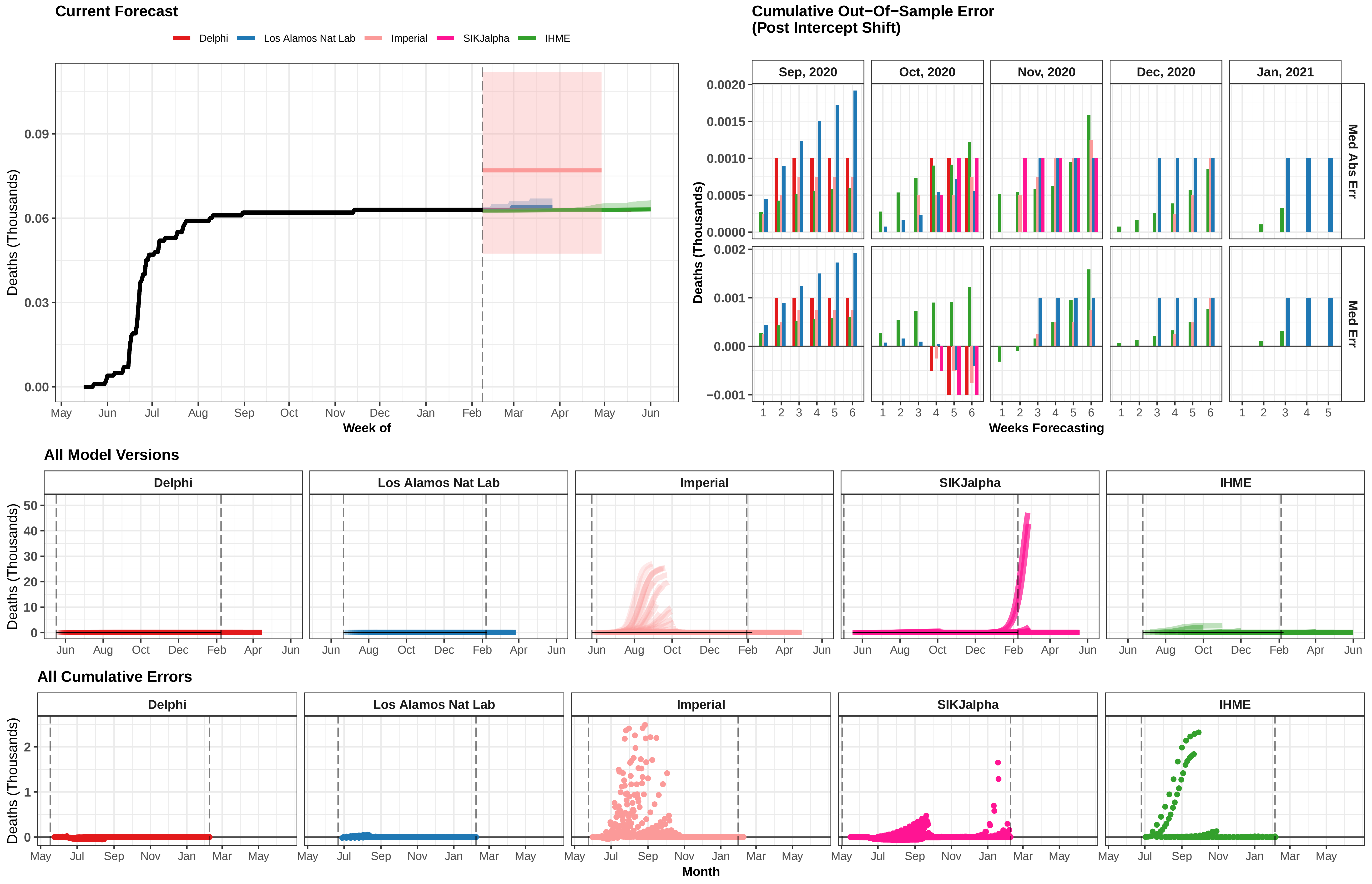

Djibouti

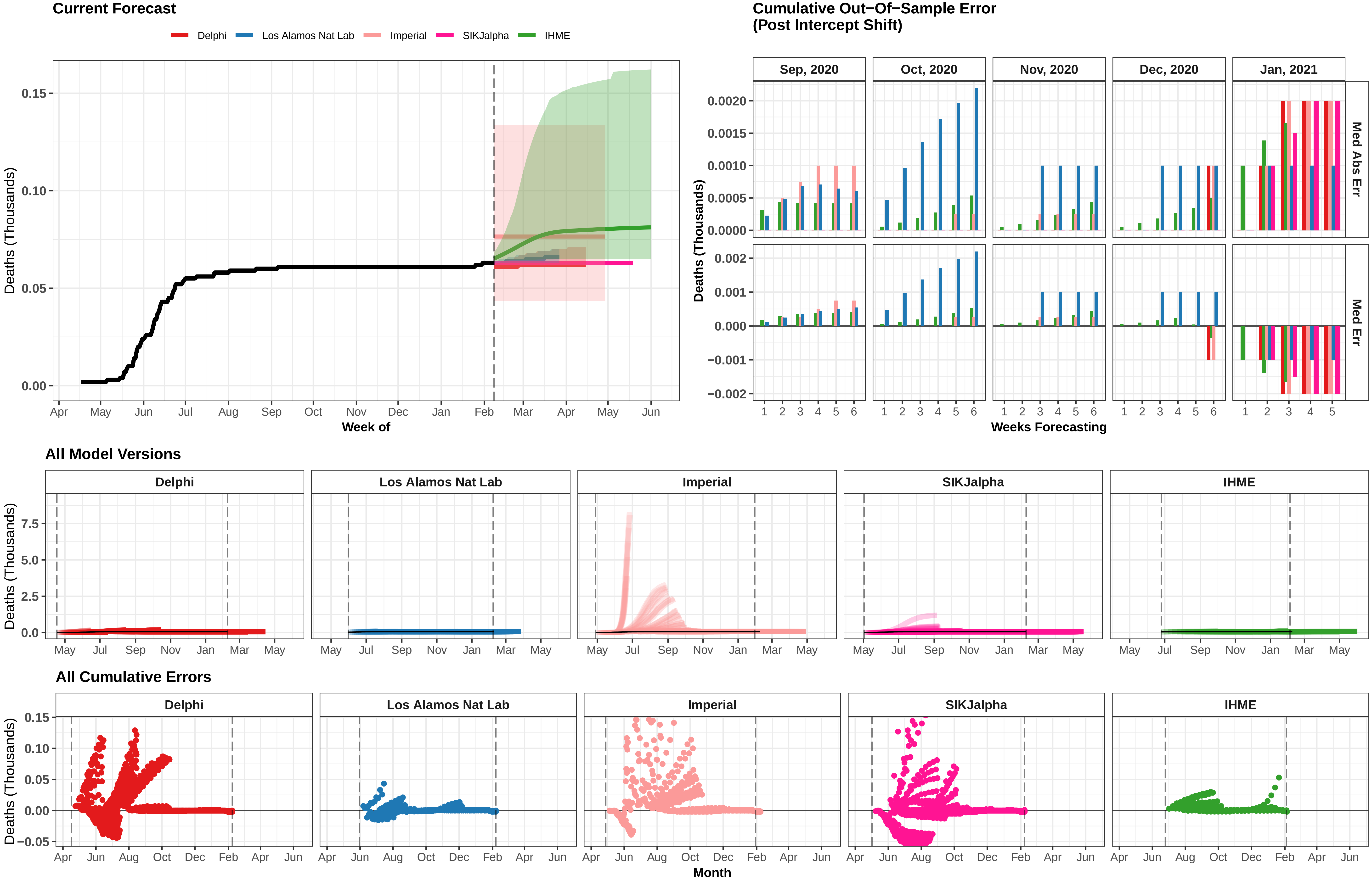
Current Forecast
Delphi
Los Alamos Nat Lab
Imperial
SIKJalpha
IHME
Deaths (Thousands)
0.15
0.10
0.05
0.00
Apr
May
Jun
Jul
Aug
Sep
Oct
Nov
Dec
Jan
Feb
Mar
Apr
May
Jun
Week of
Cumulative Out−Of−Sample Error
(Post Intercept Shift)
Sep, 2020
Oct, 2020
Nov, 2020
Dec, 2020
Jan, 2021
Med Abs Err
Med Err
0.0020
0.0015
0.0010
0.0005
0.0000
0.002
0.001
0.000
−0.001
−0.002
Weeks Forecasting
All Model Versions
7.5
5.0
2.5
0.0
All Cumulative Errors
0.15
0.10
0.05
0.00
−0.05
Month

Benin

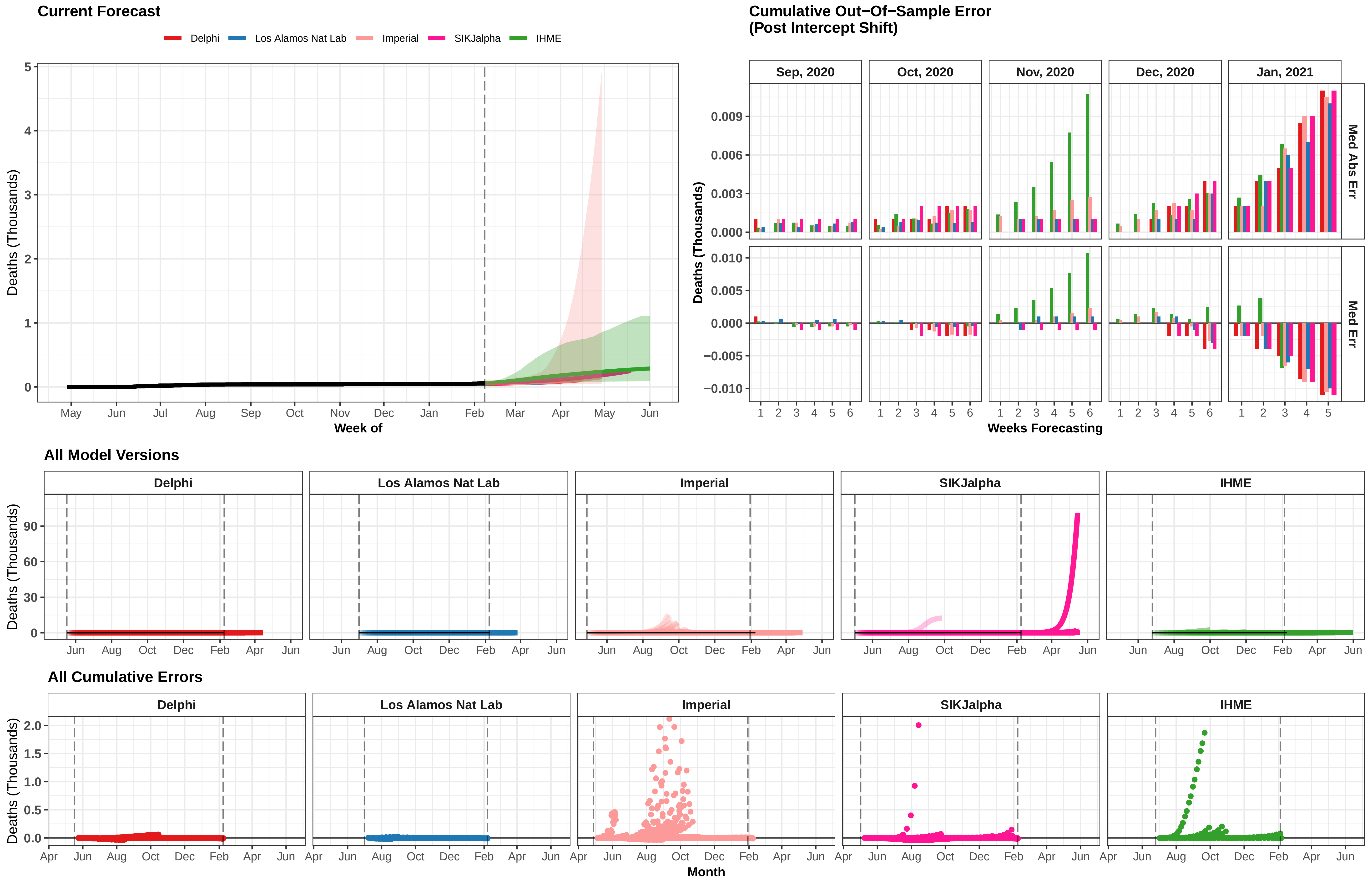

Current Forecast
Delphi
Los Alamos Nat Lab
Imperial
SIKJalpha
IHME
Deaths (Thousands)
Week of
May
Jun
Jul
Aug
Sep
Oct
Nov
Dec
Jan
Feb
Mar
Apr
Cumulative Out−Of−Sample Error
(Post Intercept Shift)
Sep, 2020
Oct, 2020
Nov, 2020
Dec, 2020
Jan, 2021
Med Abs Err
Med Err
Weeks Forecasting
All Model Versions
All Cumulative Errors
Month

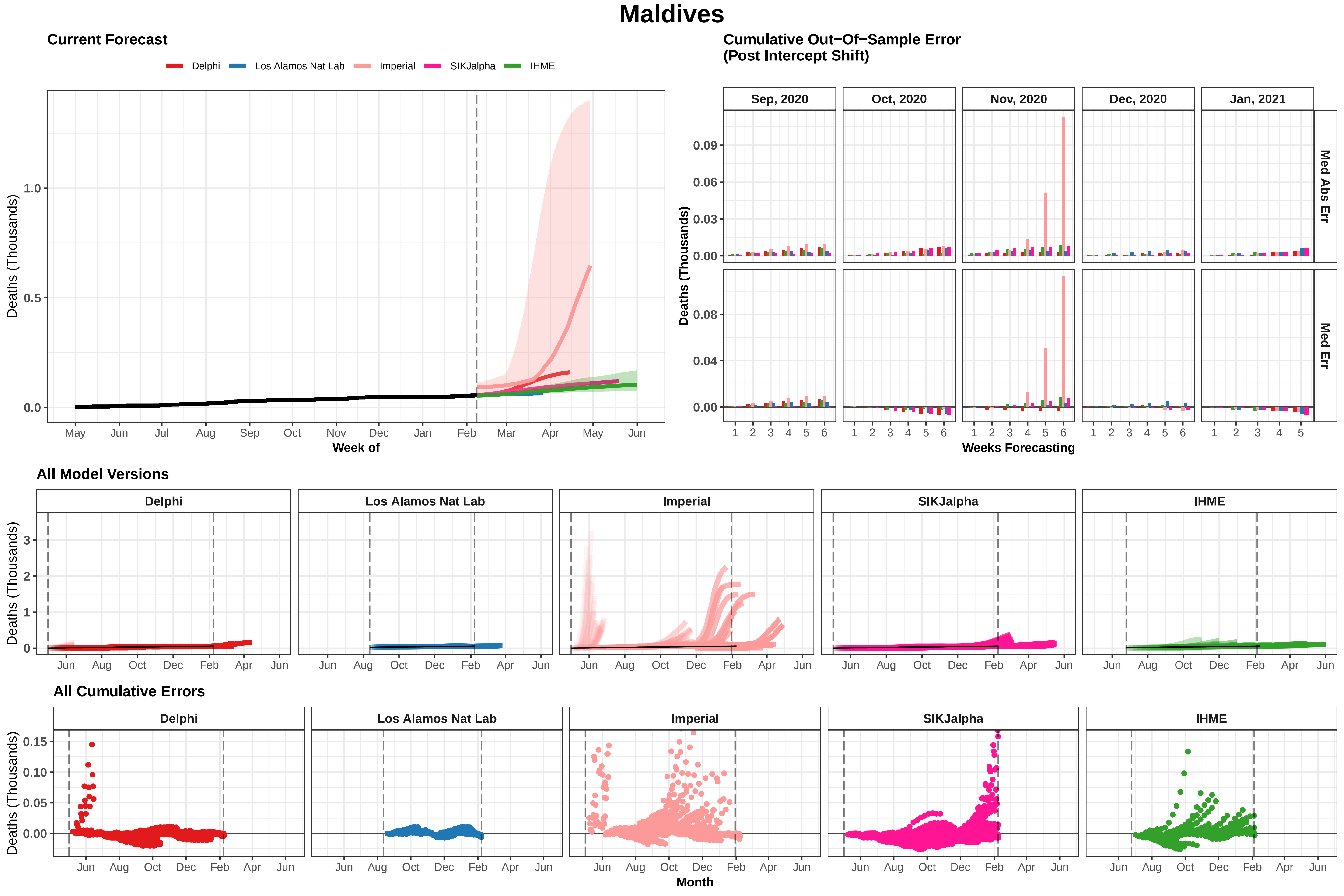

Maldives
Current Forecast
Delphi
Los Alamos Nat Lab
Imperial
SIKJalpha
IHME
Deaths (Thousands)
Week of
Cumulative Out−Of−Sample Error
(Post Intercept Shift)
Sep, 2020
Oct, 2020
Nov, 2020
Dec, 2020
Jan, 2021
Med Abs Err
Med Err
Weeks Forecasting
All Model Versions
All Cumulative Errors
Month

Guinea−Bissau

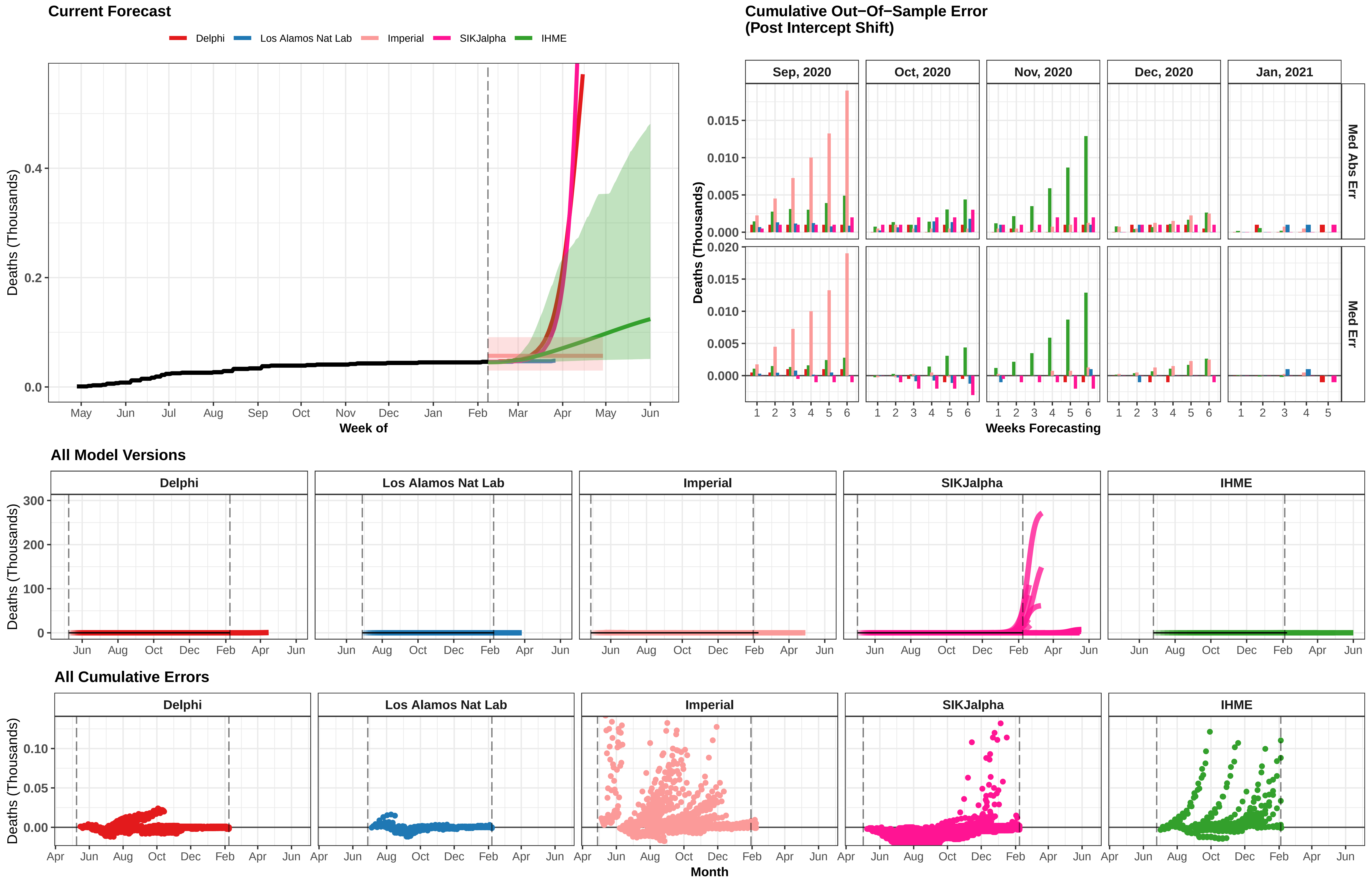

Vietnam

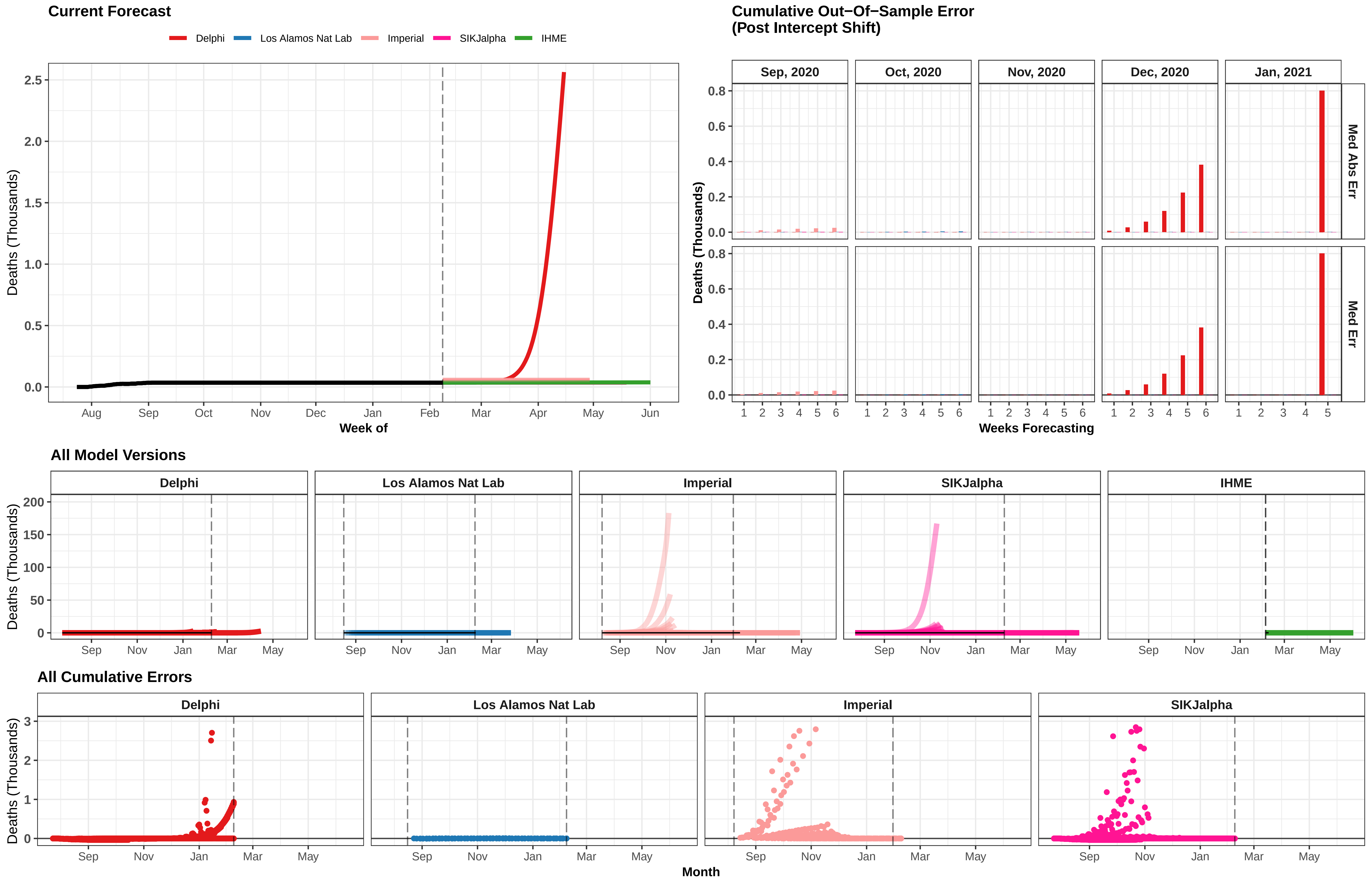

Current Forecast
Delphi
Los Alamos Nat Lab
Imperial
SIKJalpha
IHME
Deaths (Thousands)
Week of
Aug
Sep
Oct
Nov
Dec
Jan
Feb
Mar
Apr
May
Jun
Cumulative Out−Of−Sample Error
(Post Intercept Shift)
Sep, 2020
Oct, 2020
Nov, 2020
Dec, 2020
Jan, 2021
Med Abs Err
Med Err
Weeks Forecasting
All Model Versions
All Cumulative Errors
Month

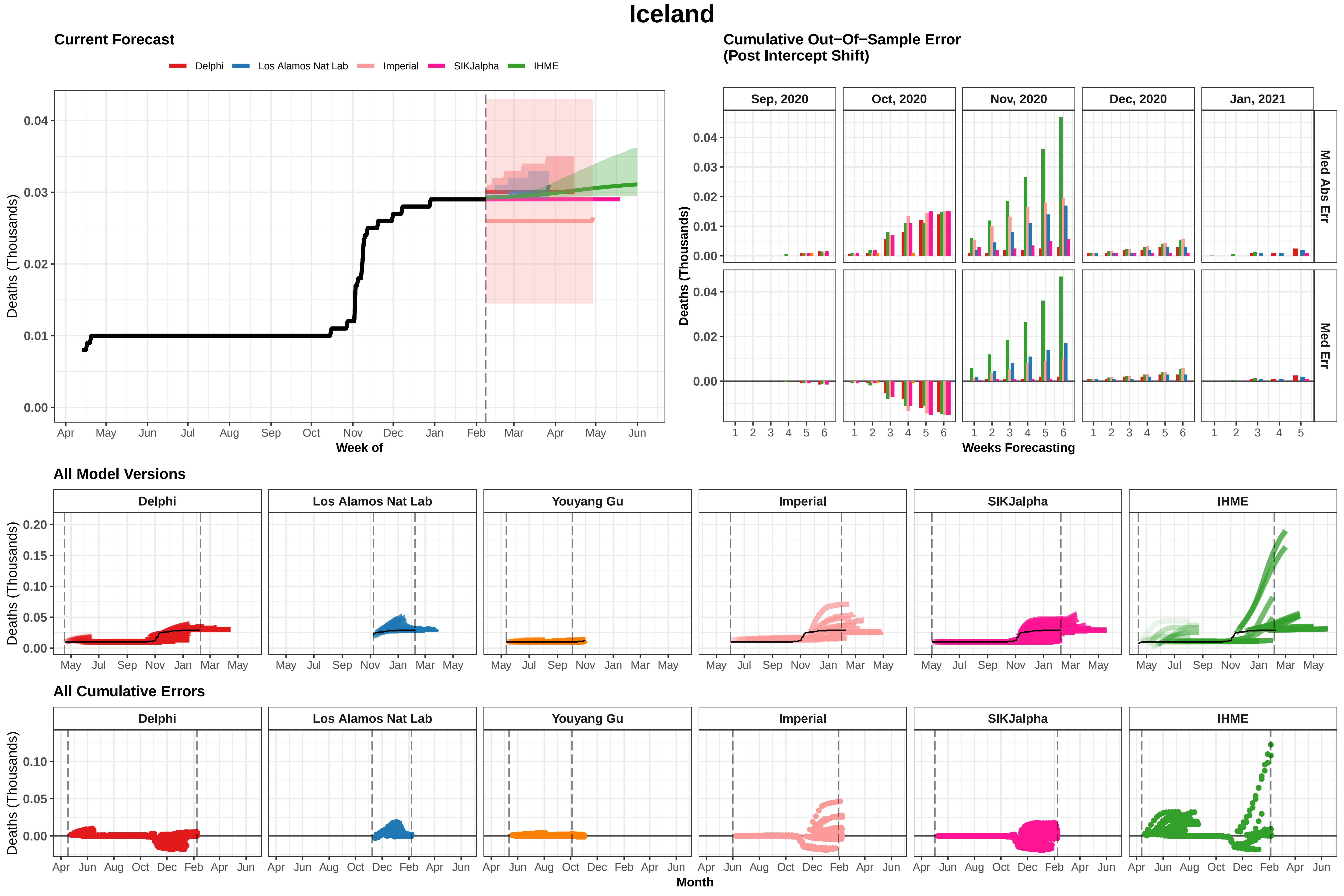
Iceland
Current Forecast
Delphi
Los Alamos Nat Lab
Imperial
SIKJalpha
IHME
Deaths (Thousands)
Week of
Apr May Jun Jul Aug Sep Oct Nov Dec Jan Feb Mar Apr May Jun
Cumulative Out−Of−Sample Error
(Post Intercept Shift)
Sep, 2020
Oct, 2020
Nov, 2020
Dec, 2020
Jan, 2021
Med Abs Err
Med Err
Weeks Forecasting
All Model Versions
Delphi
Los Alamos Nat Lab
Youyang Gu
Imperial
SIKJalpha
IHME
All Cumulative Errors
Month

Singapore

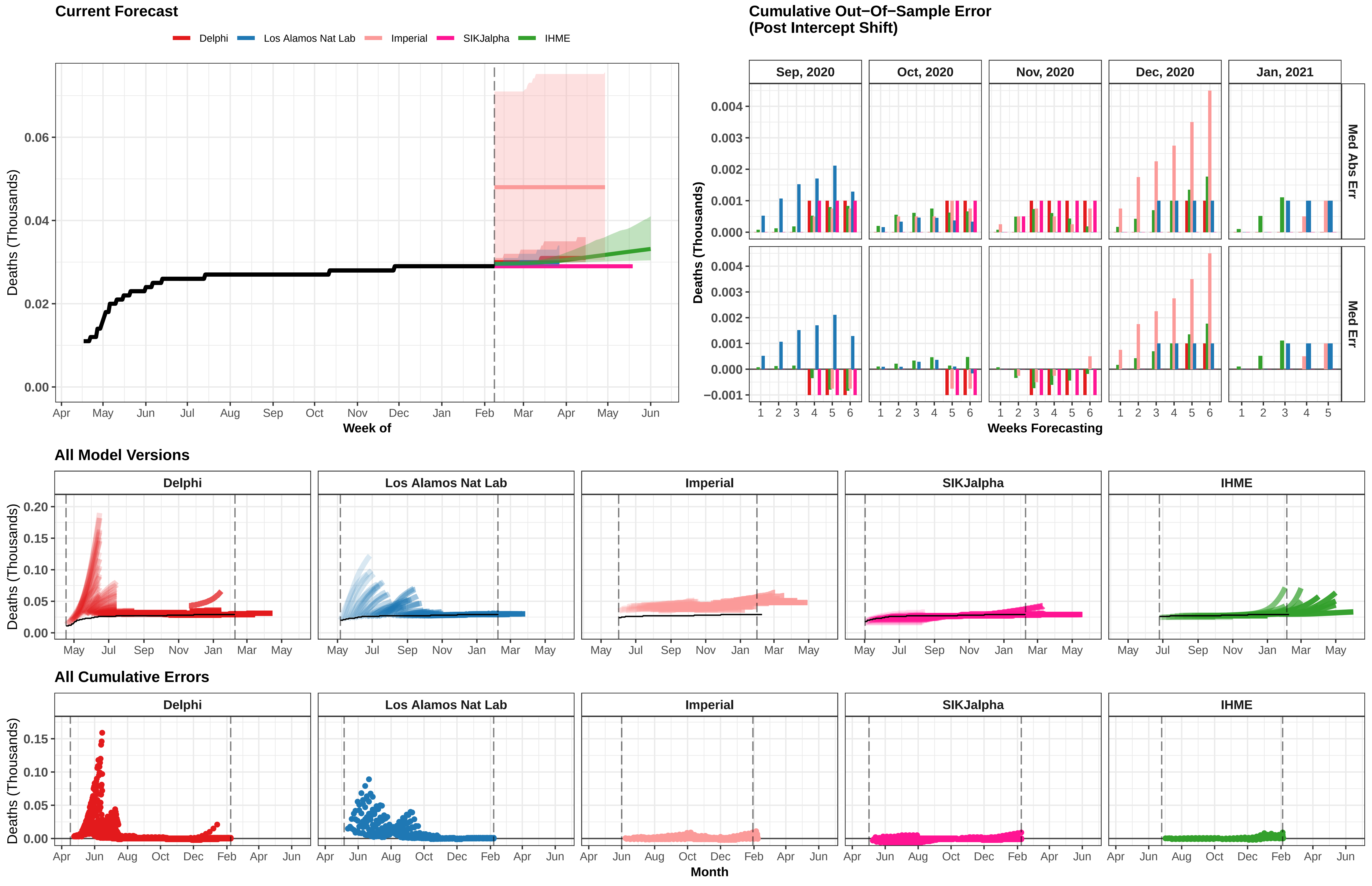

Current Forecast
Delphi
Los Alamos Nat Lab
Imperial
SIKJalpha
IHME
Deaths (Thousands)
Week of
Cumulative Out−Of−Sample Error
(Post Intercept Shift)
Sep, 2020
Oct, 2020
Nov, 2020
Dec, 2020
Jan, 2021
Med Abs Err
Med Err
Weeks Forecasting
All Model Versions
Month
All Cumulative Errors

New Zealand

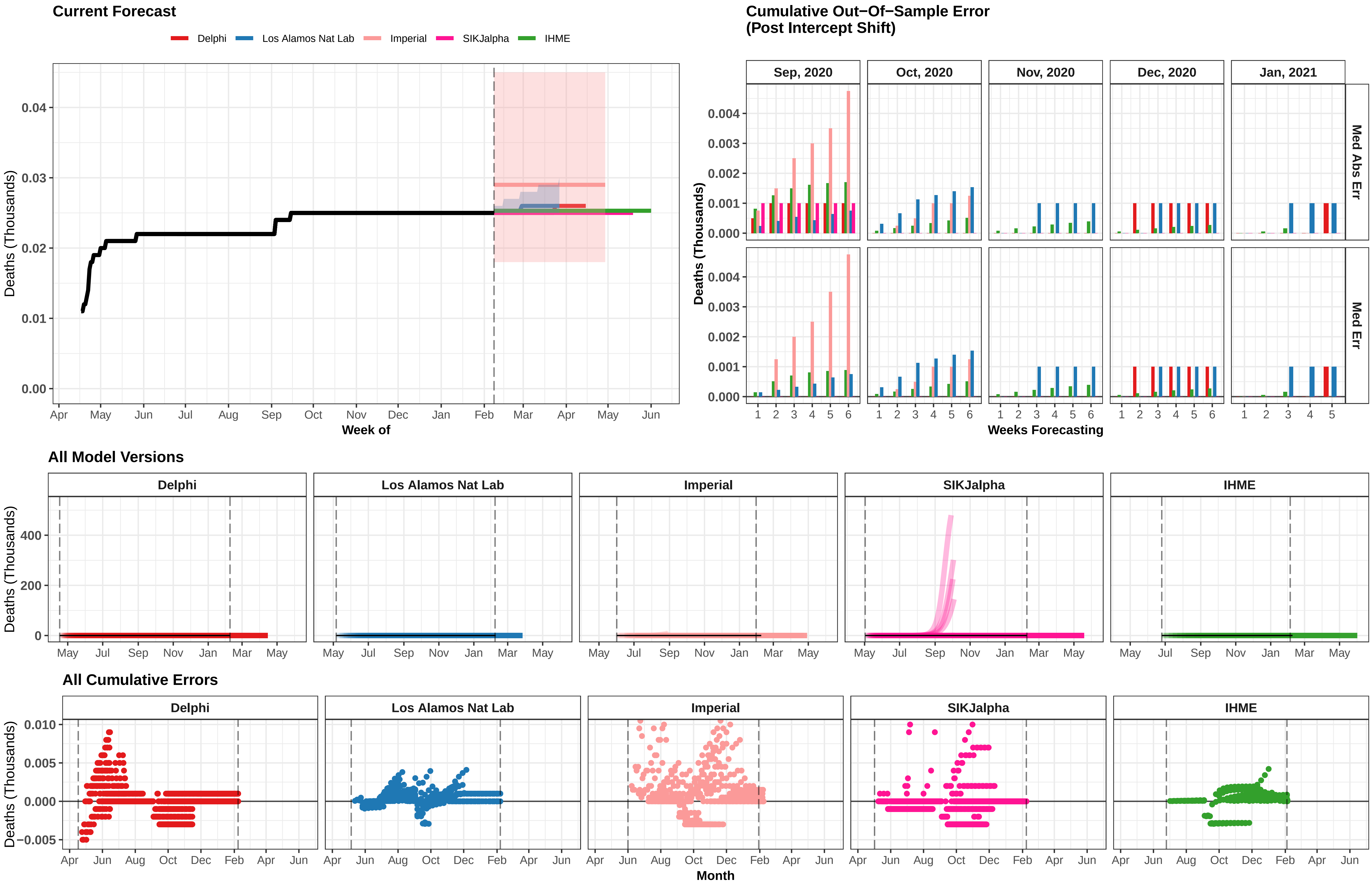

Current Forecast
Delphi
Los Alamos Nat Lab
Imperial
SIKJalpha
IHME
Deaths (Thousands)
Week of
Cumulative Out−Of−Sample Error
(Post Intercept Shift)
Sep, 2020
Oct, 2020
Nov, 2020
Dec, 2020
Jan, 2021
Med Abs Err
Med Err
Weeks Forecasting
All Model Versions
All Cumulative Errors
Month

Virgin Islands, U.S.

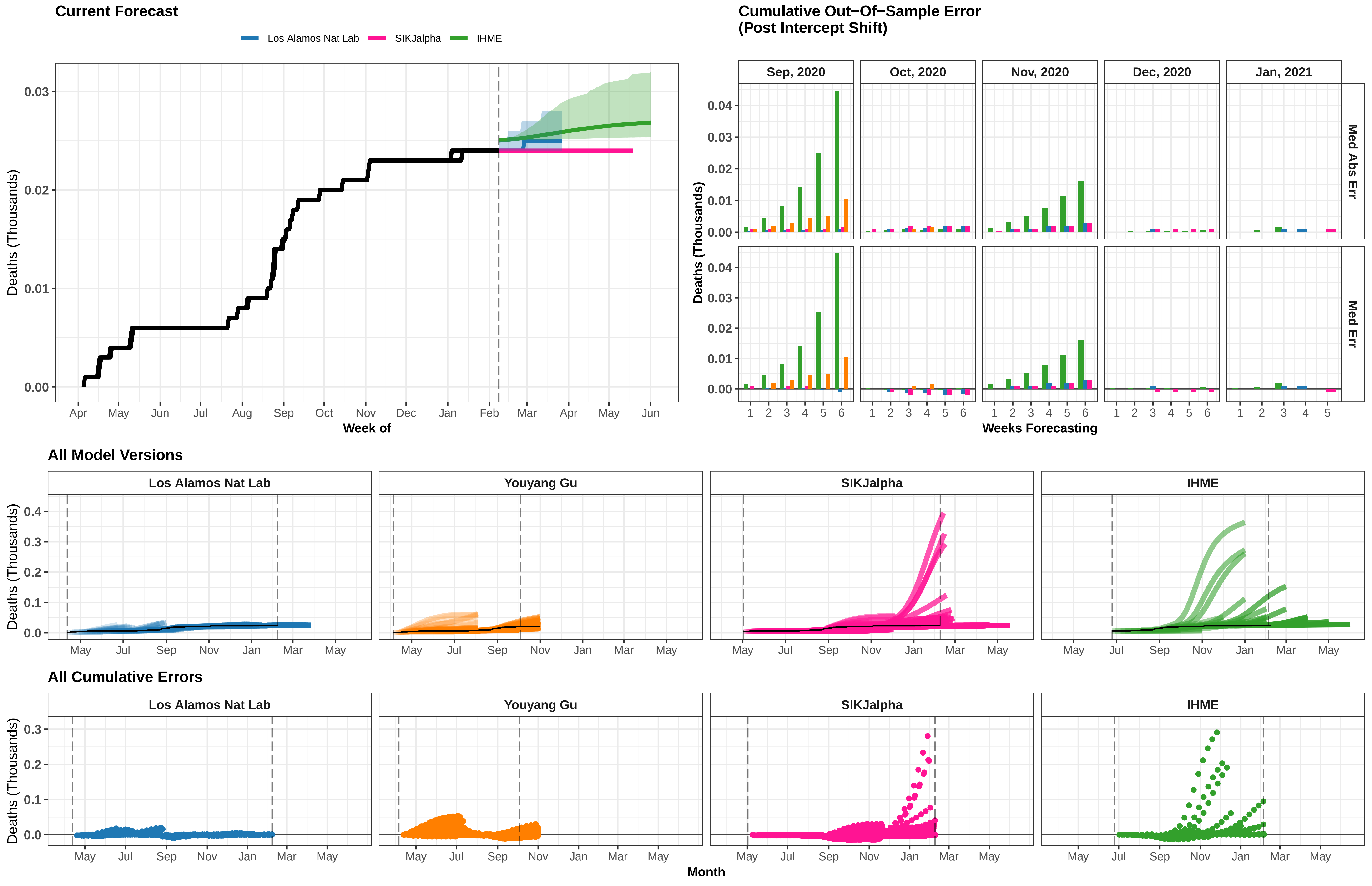

Tanzania

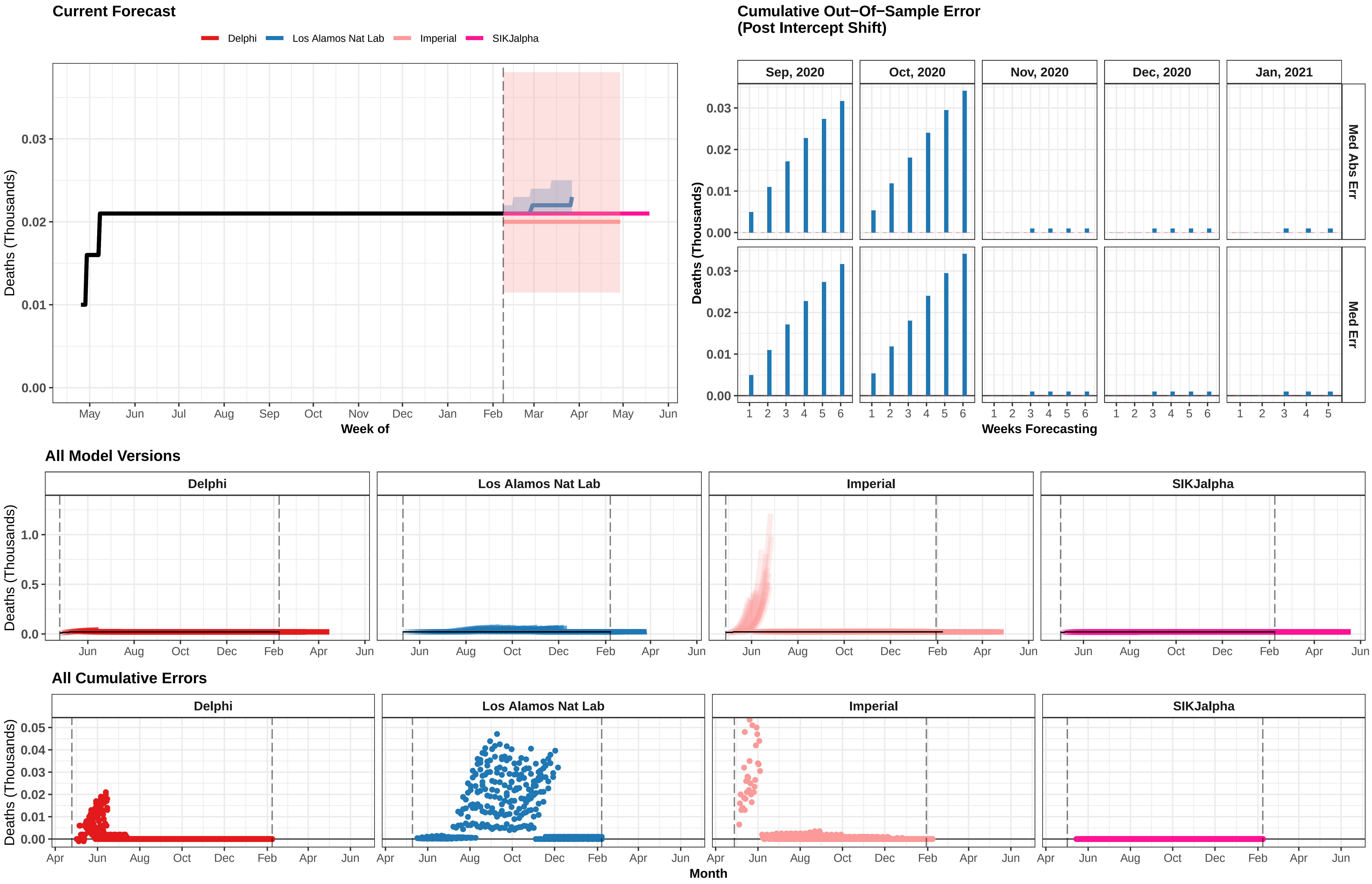

Current Forecast
Delphi
Los Alamos Nat Lab
Imperial
SIKJalpha
Deaths (Thousands)
0.00
0.01
0.02
0.03
May
Jun
Jul
Aug
Sep
Oct
Nov
Dec
Jan
Feb
Mar
Apr
May
Jun
Week of
Cumulative Out−Of−Sample Error
(Post Intercept Shift)
Sep, 2020
Oct, 2020
Nov, 2020
Dec, 2020
Jan, 2021
Med Abs Err
Med Err
Weeks Forecasting
All Model Versions
Delphi
Los Alamos Nat Lab
Imperial
SIKJalpha
0.0
0.5
1.0
All Cumulative Errors
0.00
0.01
0.02
0.03
0.04
0.05
Apr
Jun
Aug
Oct
Dec
Feb
Apr
Jun
Month

Saint Lucia

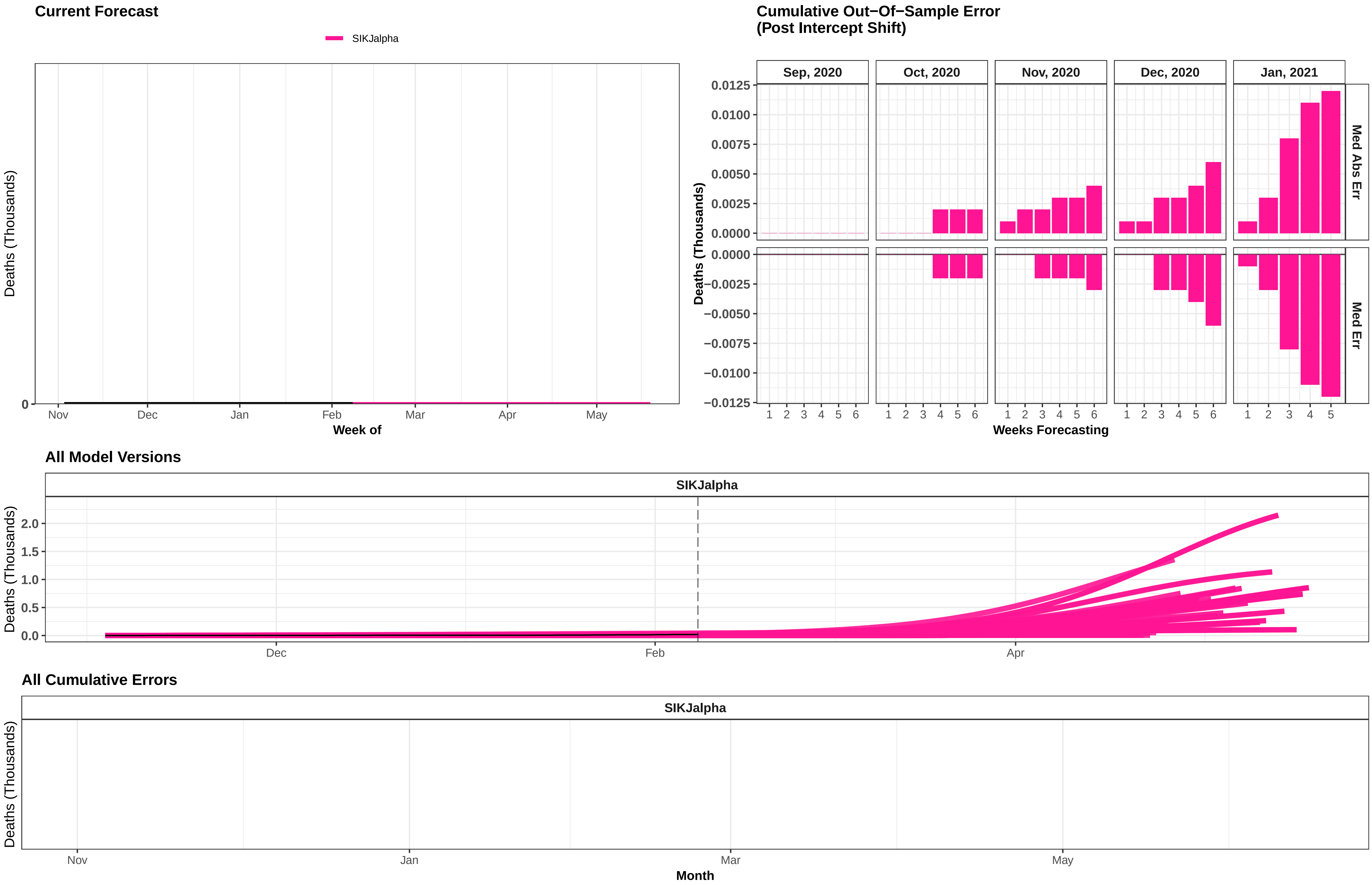
Current Forecast
SIKJalpha
Deaths (Thousands)
0
Nov
Dec
Jan
Feb
Mar
Apr
May
Week of
Cumulative Out−Of−Sample Error
(Post Intercept Shift)
Sep, 2020
Oct, 2020
Nov, 2020
Dec, 2020
Jan, 2021
Med Abs Err
Med Err
0.0125
0.0100
0.0075
0.0050
0.0025
0.0000
−0.0025
−0.0050
−0.0075
−0.0100
−0.0125
Weeks Forecasting
All Model Versions
SIKJalpha
2.0
1.5
1.0
0.5
0.0
Dec
Feb
Apr
All Cumulative Errors
SIKJalpha
Nov
Jan
Mar
May
Month

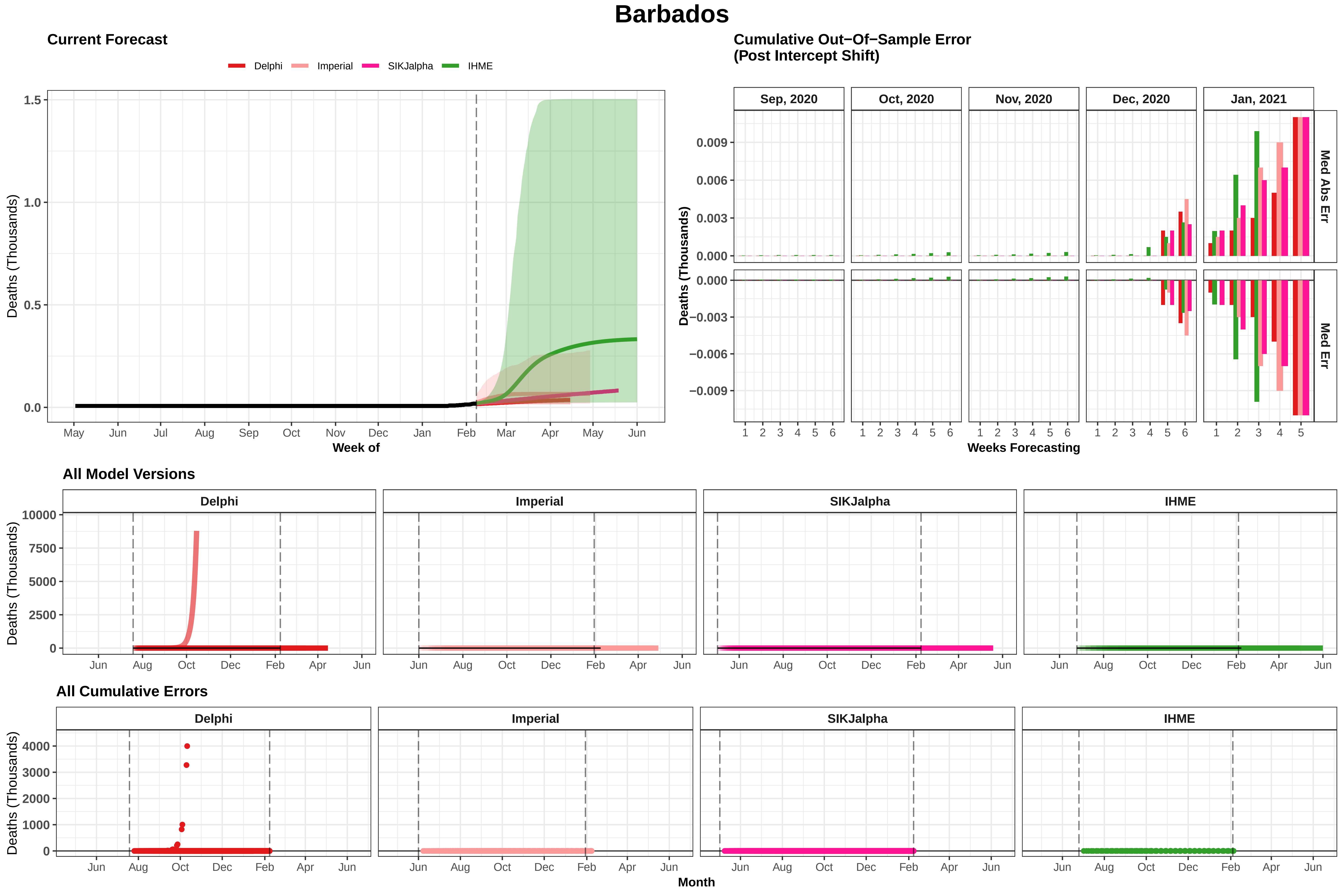

Barbados
Current Forecast
Delphi
Imperial
SIKJalpha
IHME
Deaths (Thousands)
Week of
Cumulative Out−Of−Sample Error
(Post Intercept Shift)
Sep, 2020
Oct, 2020
Nov, 2020
Dec, 2020
Jan, 2021
Med Abs Err
Med Err
Weeks Forecasting
All Model Versions
All Cumulative Errors
Month

Sao Tome and Principe

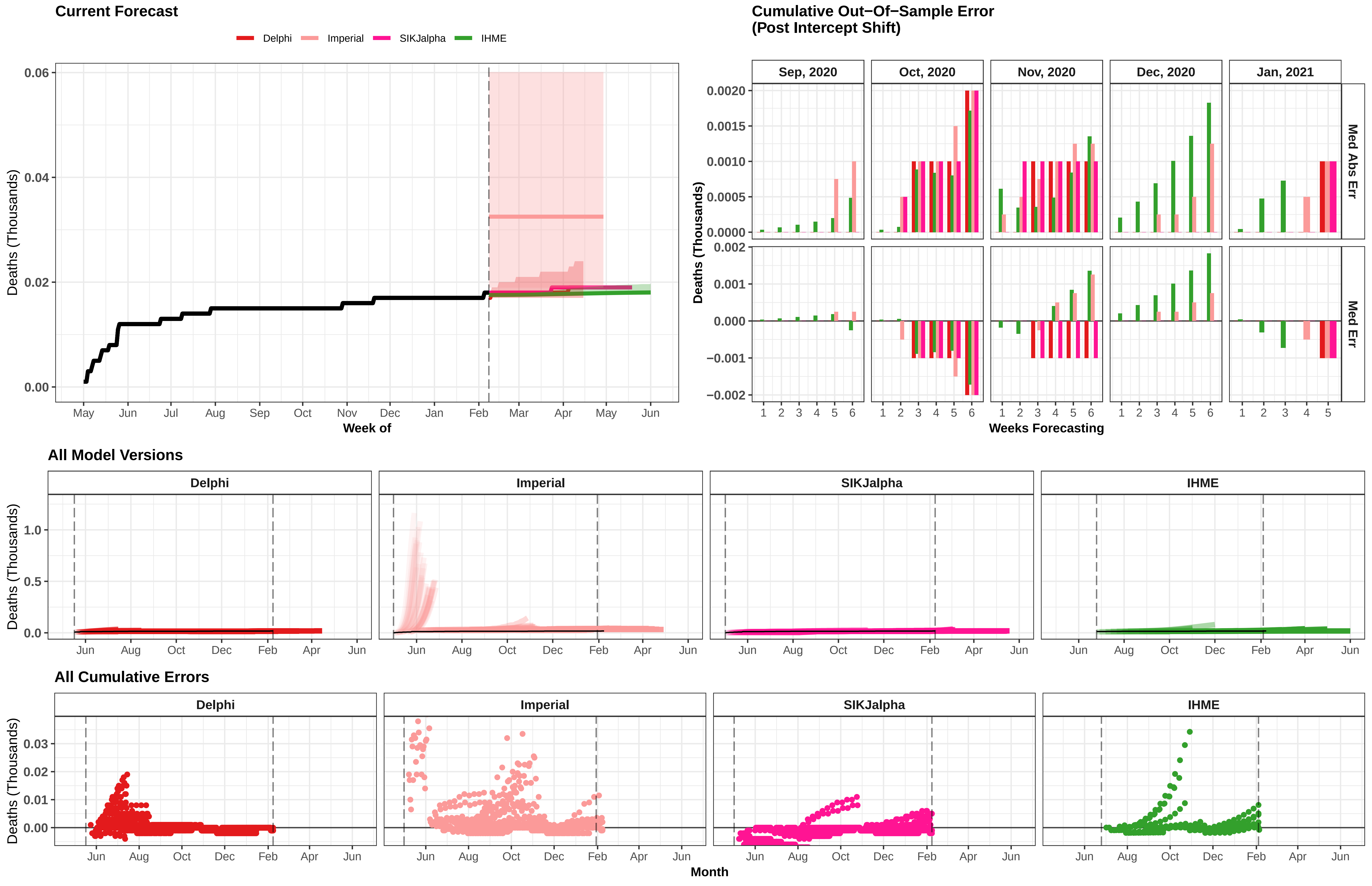

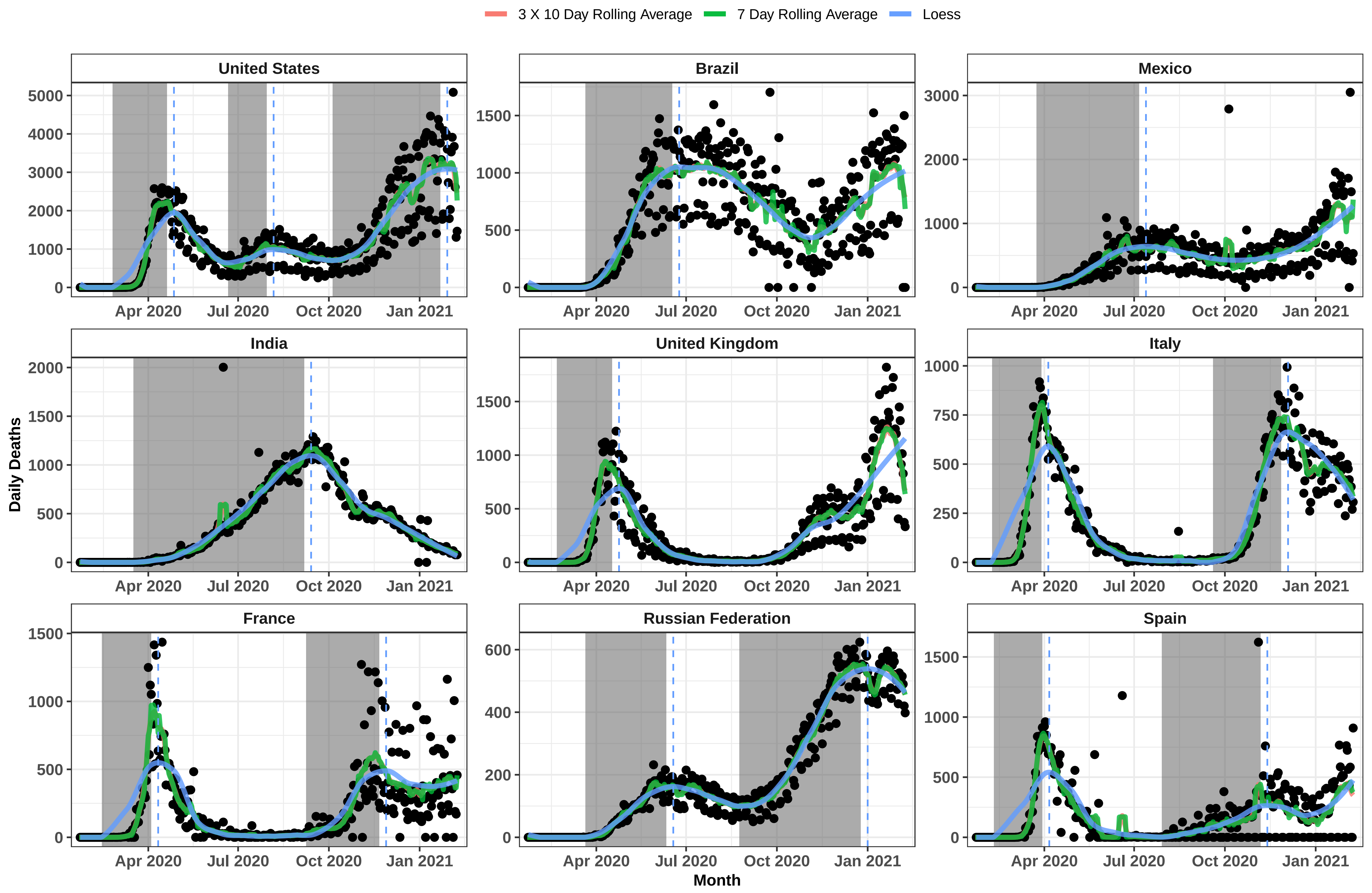

3 X 10 Day Rolling Average
7 Day Rolling Average
Loess
United States
Brazil
Mexico
India
United Kingdom
Italy
France
Russian Federation
Spain
Daily Deaths
Month
Apr 2020
Jul 2020
Oct 2020
Jan 2021

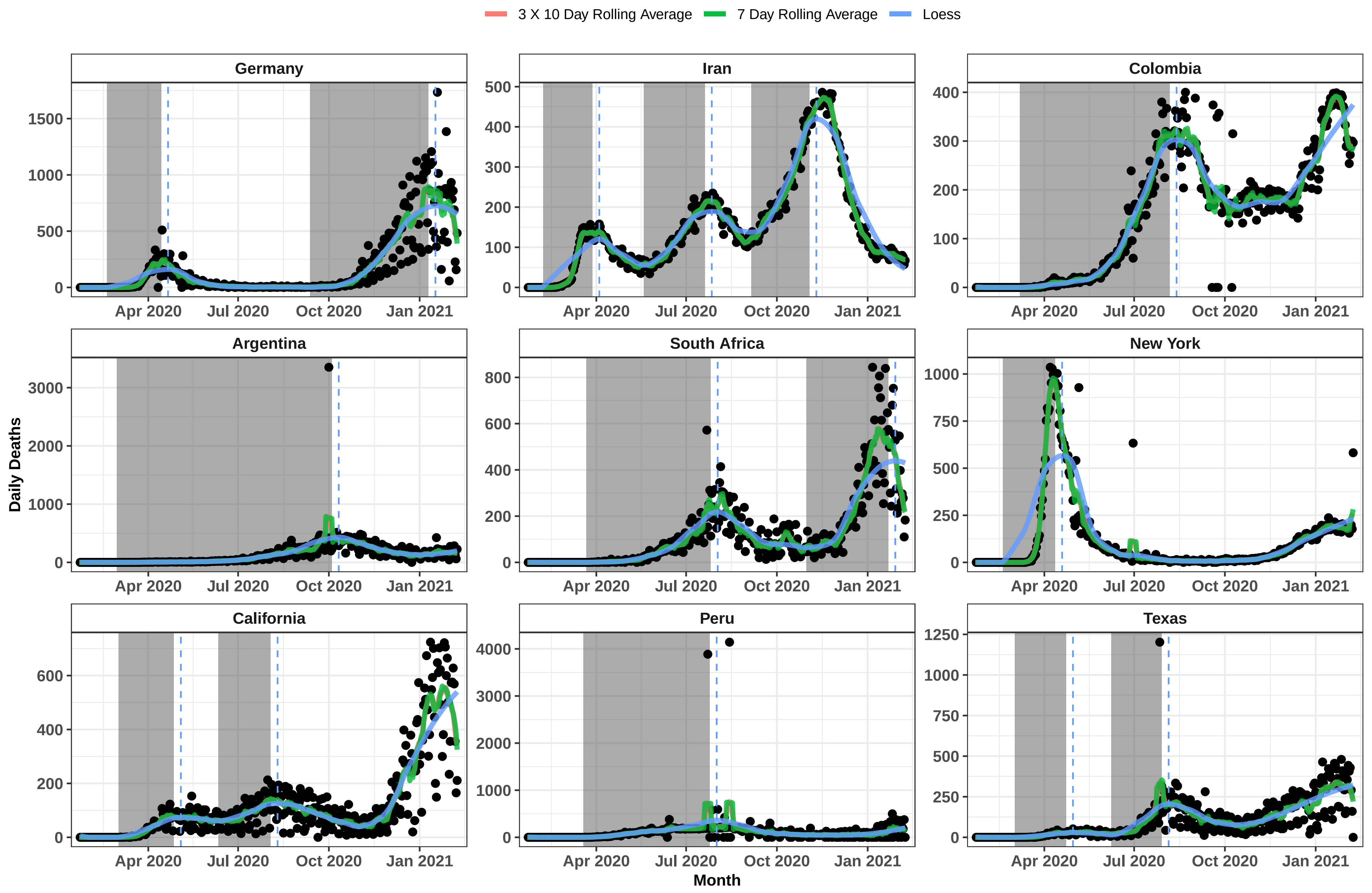

3 X 10 Day Rolling Average
7 Day Rolling Average
Loess
Germany
Iran
Colombia
Argentina
South Africa
New York
California
Peru
Texas
Daily Deaths
Month
Apr 2020
Jul 2020
Oct 2020
Jan 2021

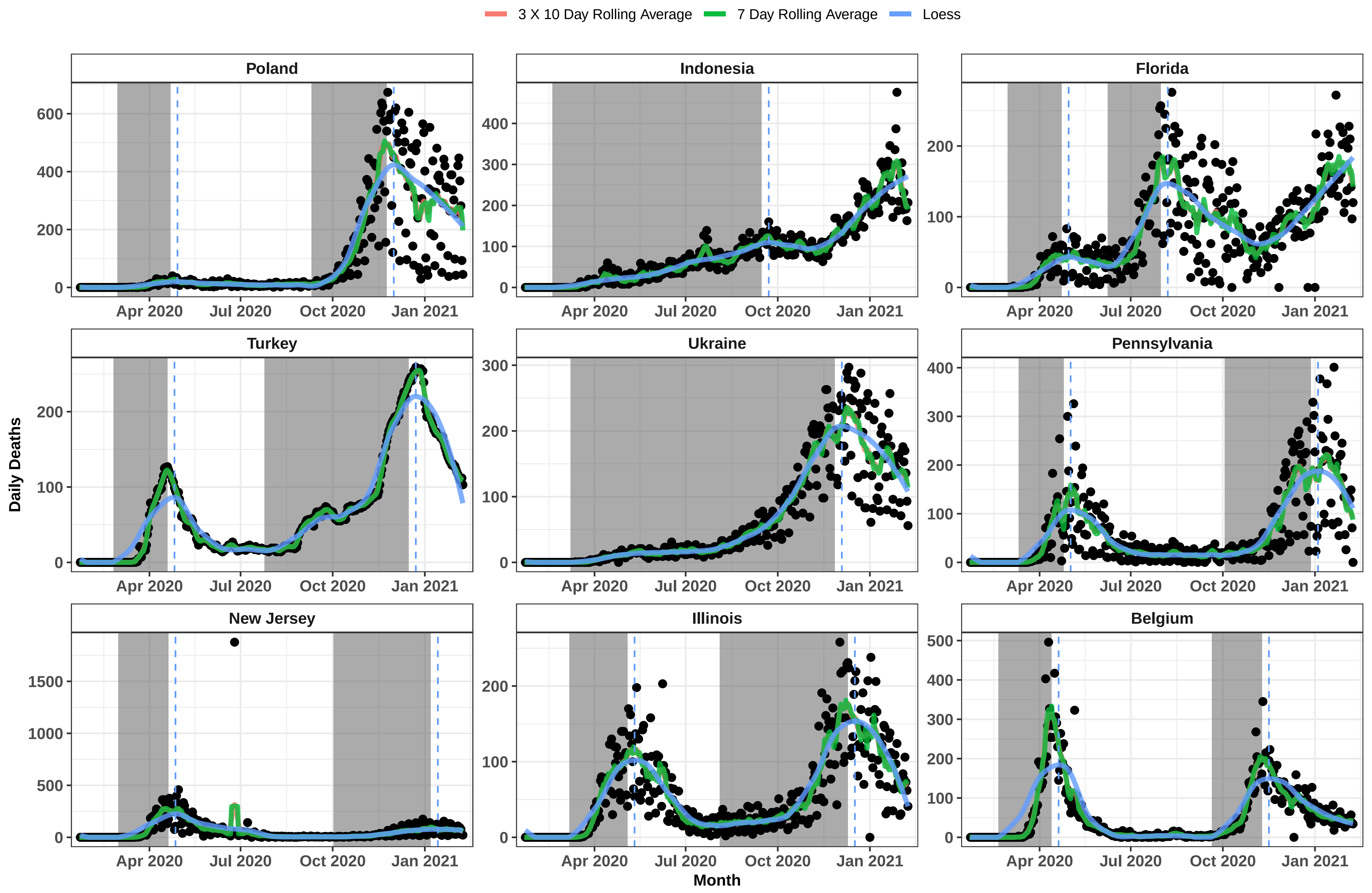

3 X 10 Day Rolling Average
7 Day Rolling Average
Loess
Poland
Indonesia
Florida
Turkey
Ukraine
Pennsylvania
New Jersey
Illinois
Belgium
Daily Deaths
Month
Apr 2020
Jul 2020
Oct 2020
Jan 2021

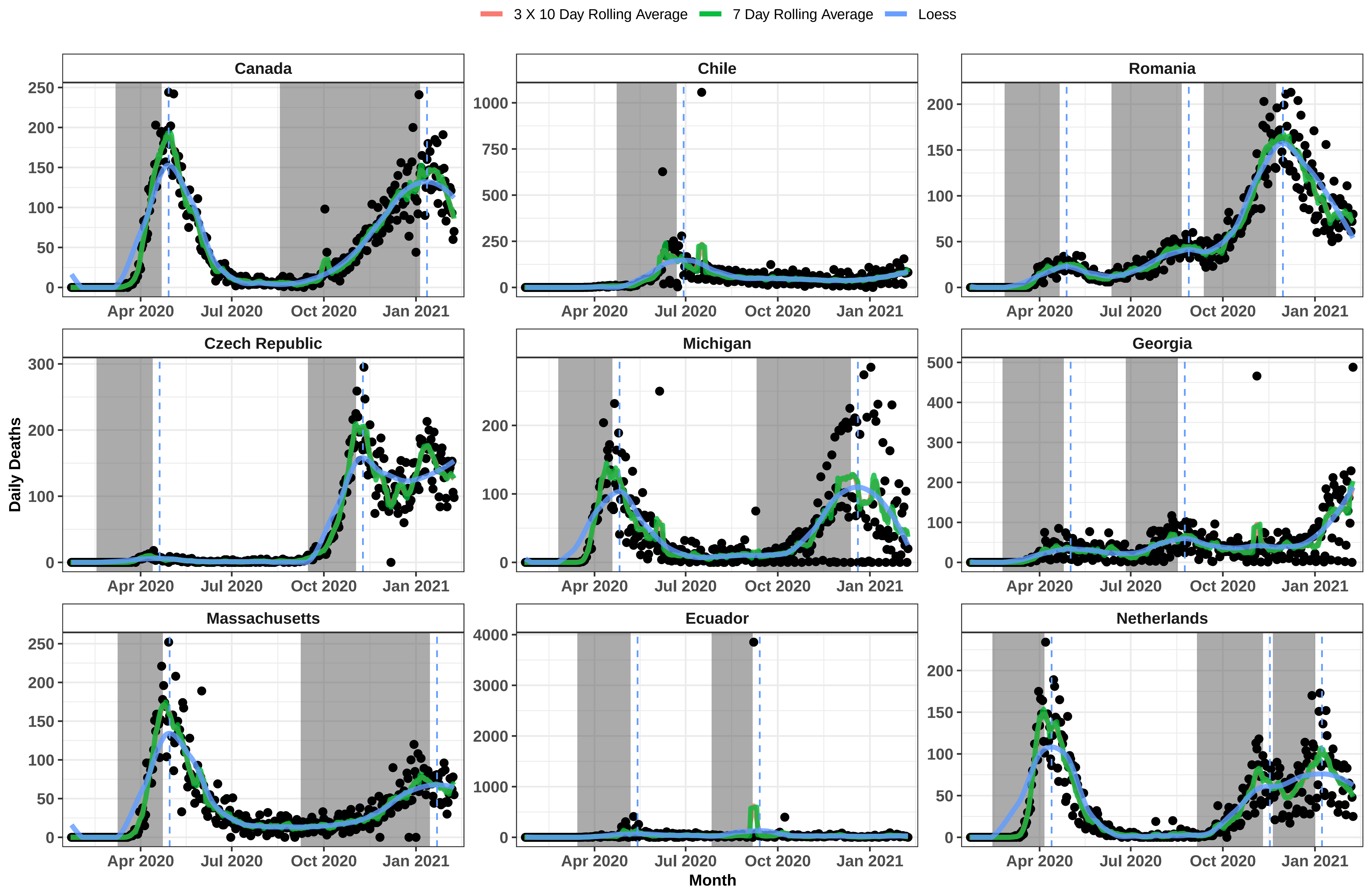

3 X 10 Day Rolling Average
7 Day Rolling Average
Loess
Canada
Chile
Romania
Czech Republic
Michigan
Georgia
Massachusetts
Ecuador
Netherlands
Daily Deaths
Month
Apr 2020
Jul 2020
Oct 2020
Jan 2021

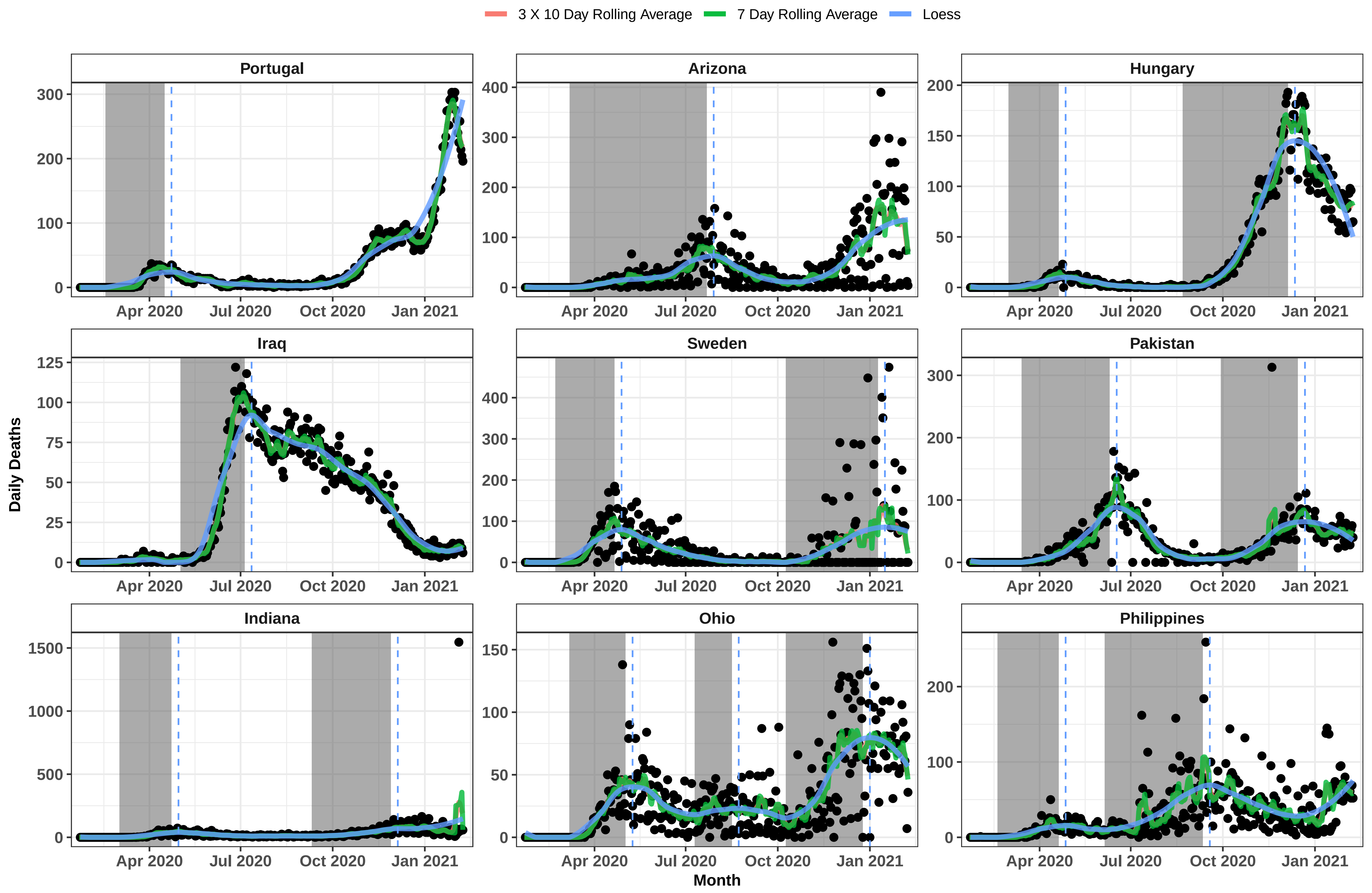
3 X 10 Day Rolling Average
7 Day Rolling Average
Loess
Portugal
Arizona
Hungary
Iraq
Sweden
Pakistan
Indiana
Ohio
Philippines
Daily Deaths
Month
Apr 2020
Jul 2020
Oct 2020
Jan 2021

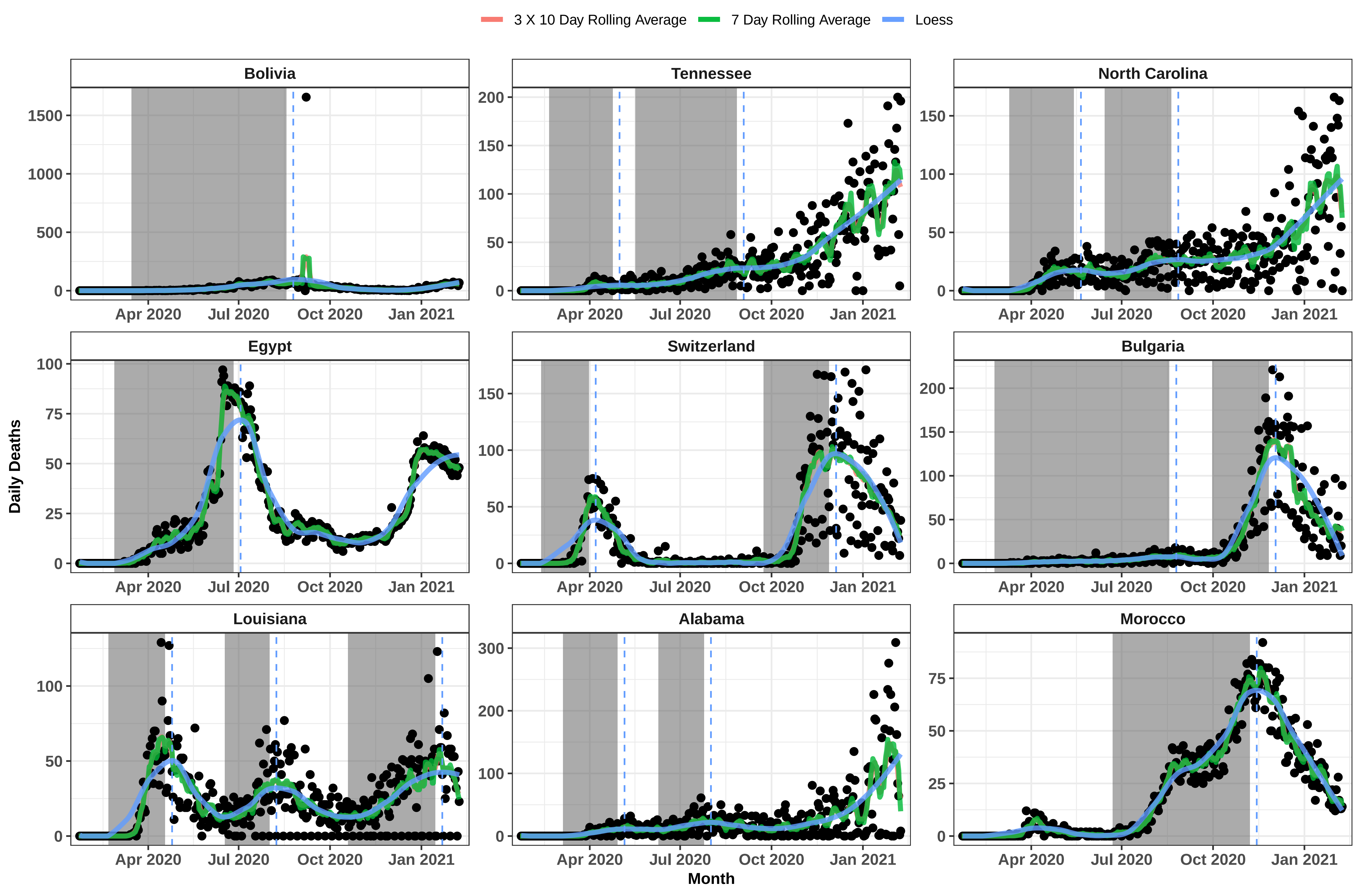
3 X 10 Day Rolling Average
7 Day Rolling Average
Loess
Bolivia
Tennessee
North Carolina
Egypt
Switzerland
Bulgaria
Louisiana
Alabama
Morocco
Daily Deaths
Month
Apr 2020
Jul 2020
Oct 2020
Jan 2021

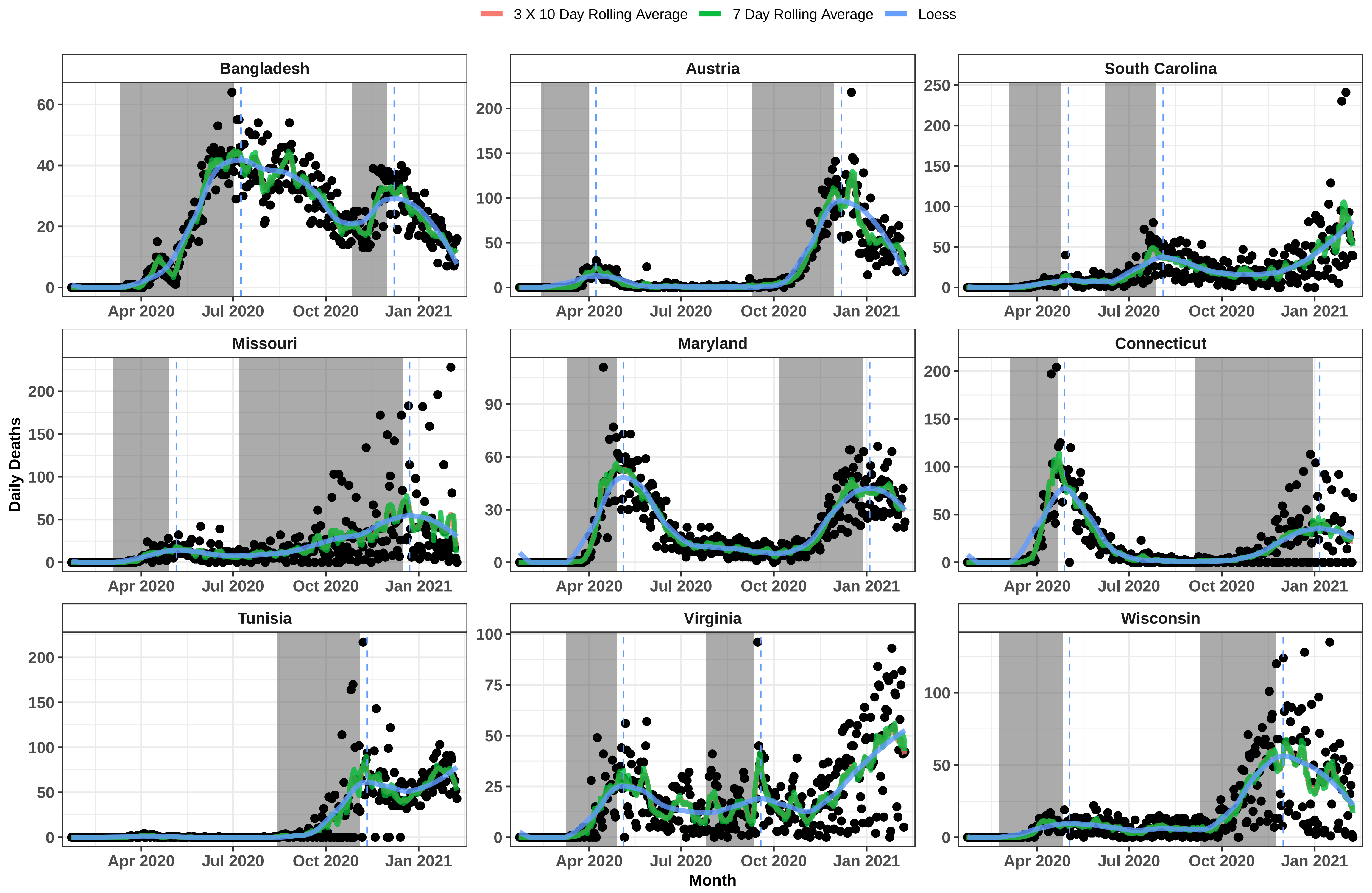

3 X 10 Day Rolling Average
7 Day Rolling Average
Loess
Bangladesh
Austria
South Carolina
Missouri
Maryland
Connecticut
Tunisia
Virginia
Wisconsin
Daily Deaths
Month
Apr 2020
Jul 2020
Oct 2020
Jan 2021

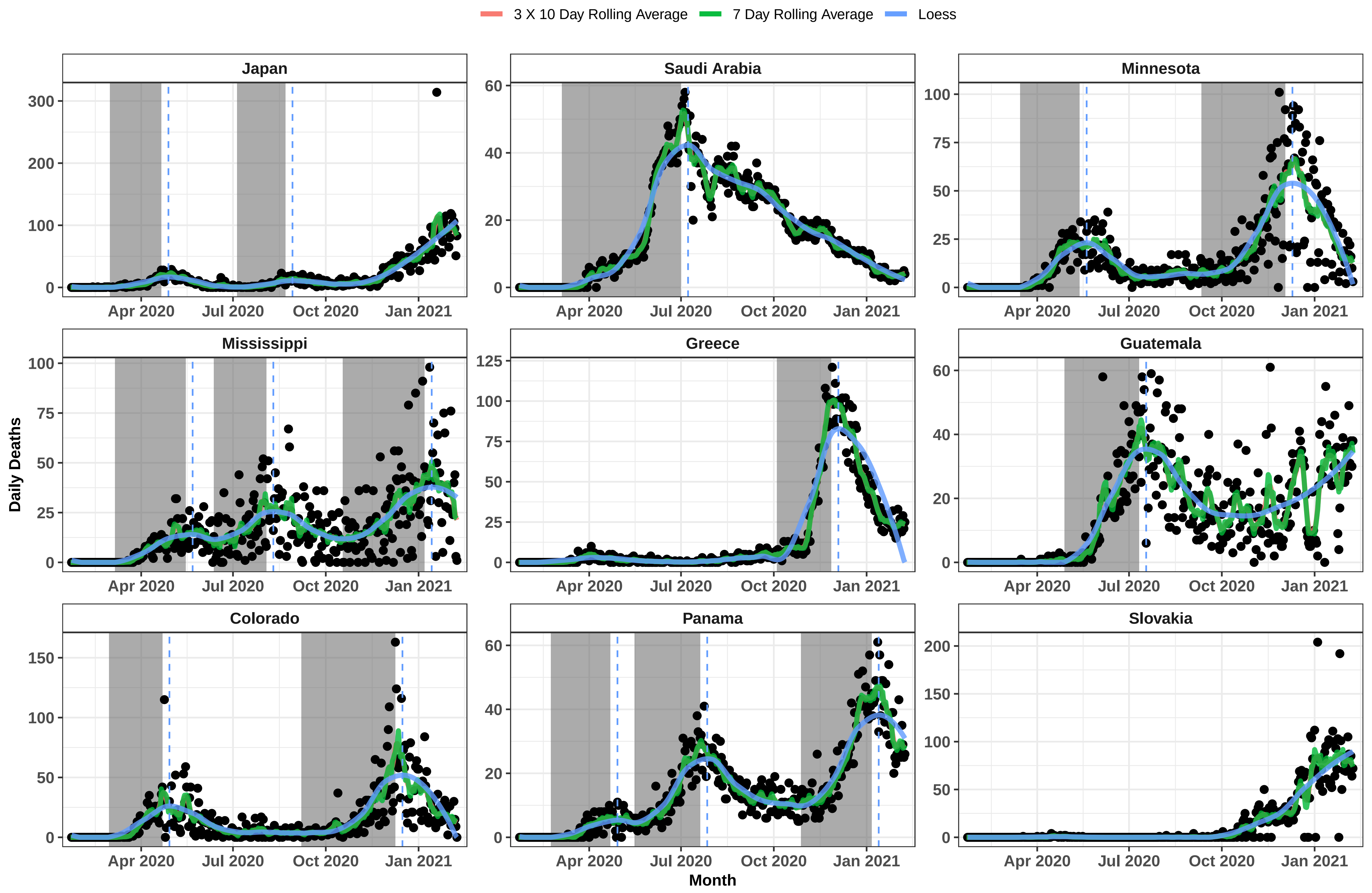

3 X 10 Day Rolling Average
7 Day Rolling Average
Loess
Japan
Saudi Arabia
Minnesota
Mississippi
Greece
Guatemala
Colorado
Panama
Slovakia
Daily Deaths
Month
Apr 2020
Jul 2020
Oct 2020
Jan 2021

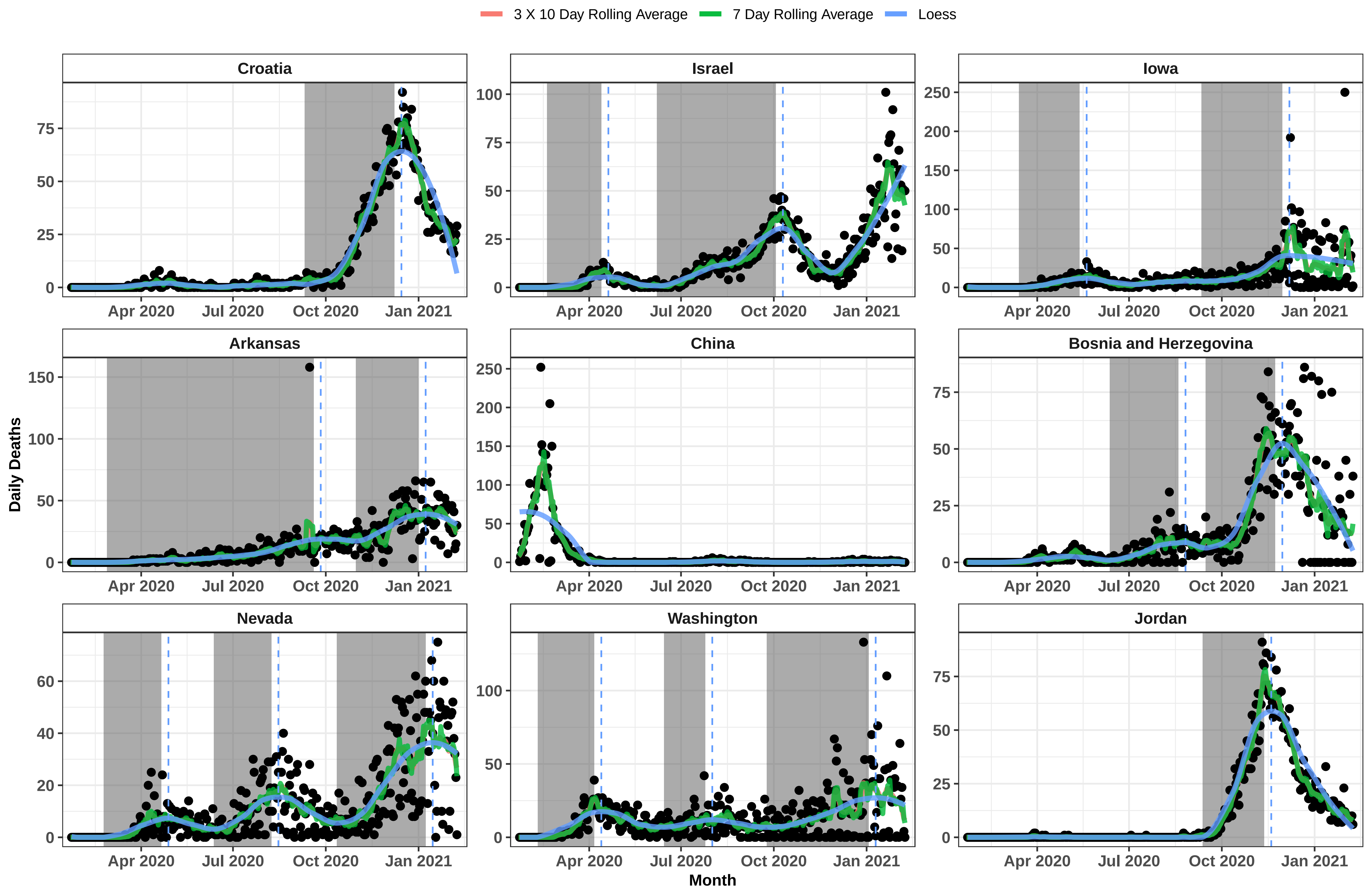
3 X 10 Day Rolling Average
7 Day Rolling Average
Loess
Croatia
Israel
Iowa
Arkansas
China
Bosnia and Herzegovina
Nevada
Washington
Jordan
Daily Deaths
Month
Apr 2020
Jul 2020
Oct 2020
Jan 2021

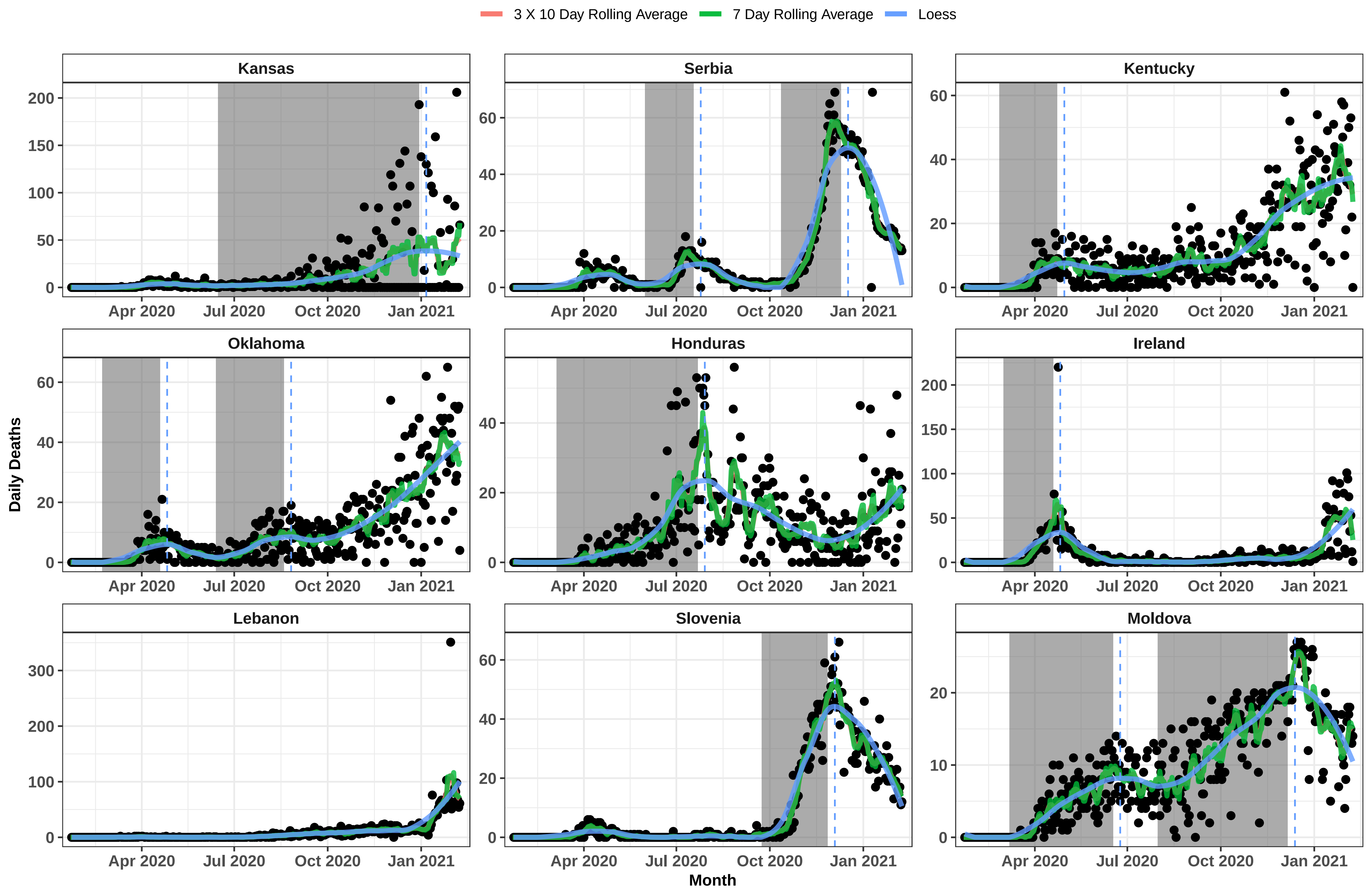

3 X 10 Day Rolling Average
7 Day Rolling Average
Loess
Kansas
Serbia
Kentucky
Oklahoma
Honduras
Ireland
Lebanon
Slovenia
Moldova
Daily Deaths
Month
Apr 2020
Jul 2020
Oct 2020
Jan 2021

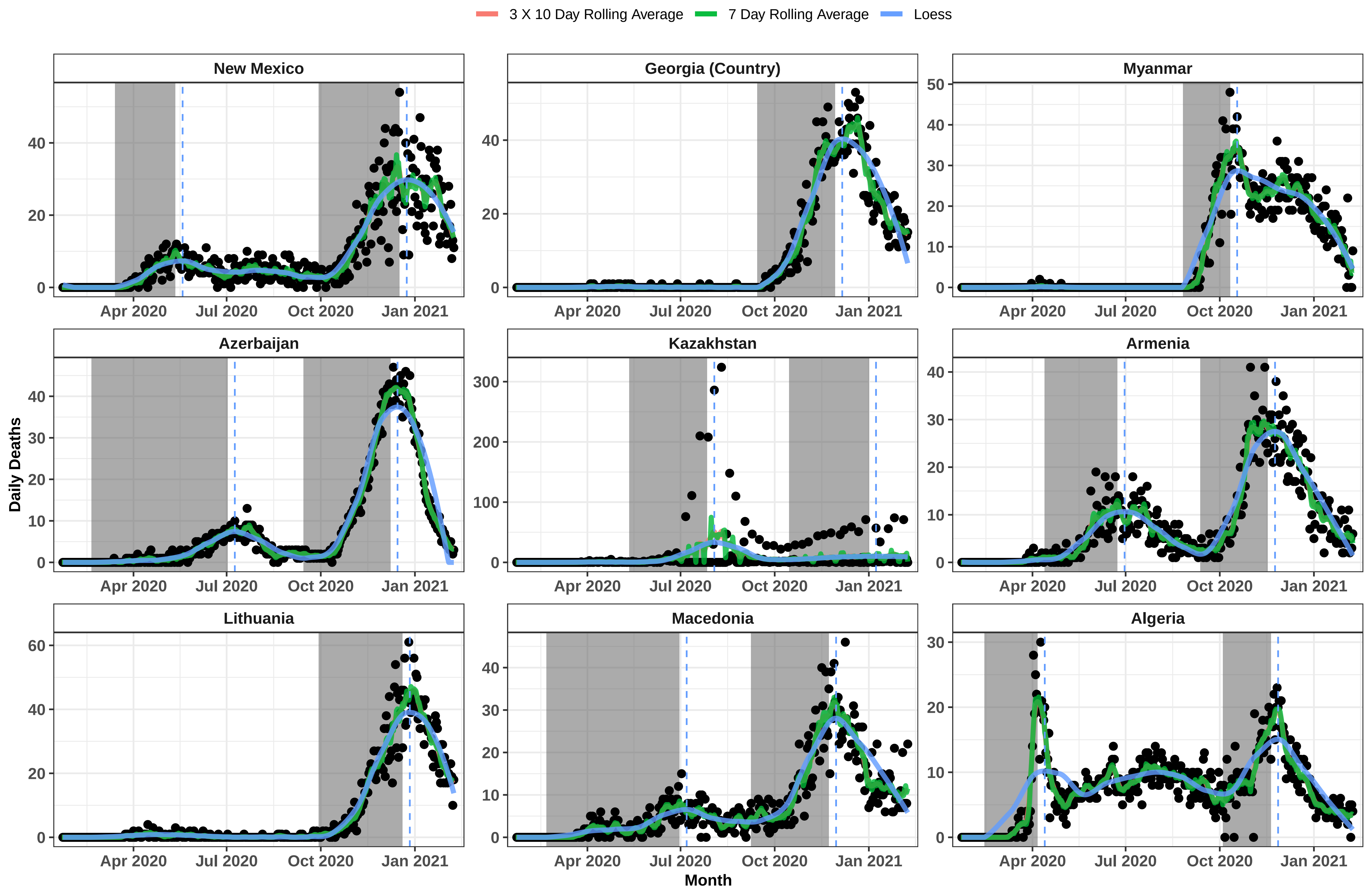

3 X 10 Day Rolling Average
7 Day Rolling Average
Loess
New Mexico
Georgia (Country)
Myanmar
Azerbaijan
Kazakhstan
Armenia
Lithuania
Macedonia
Algeria
Daily Deaths
Month
Apr 2020
Jul 2020
Oct 2020
Jan 2021

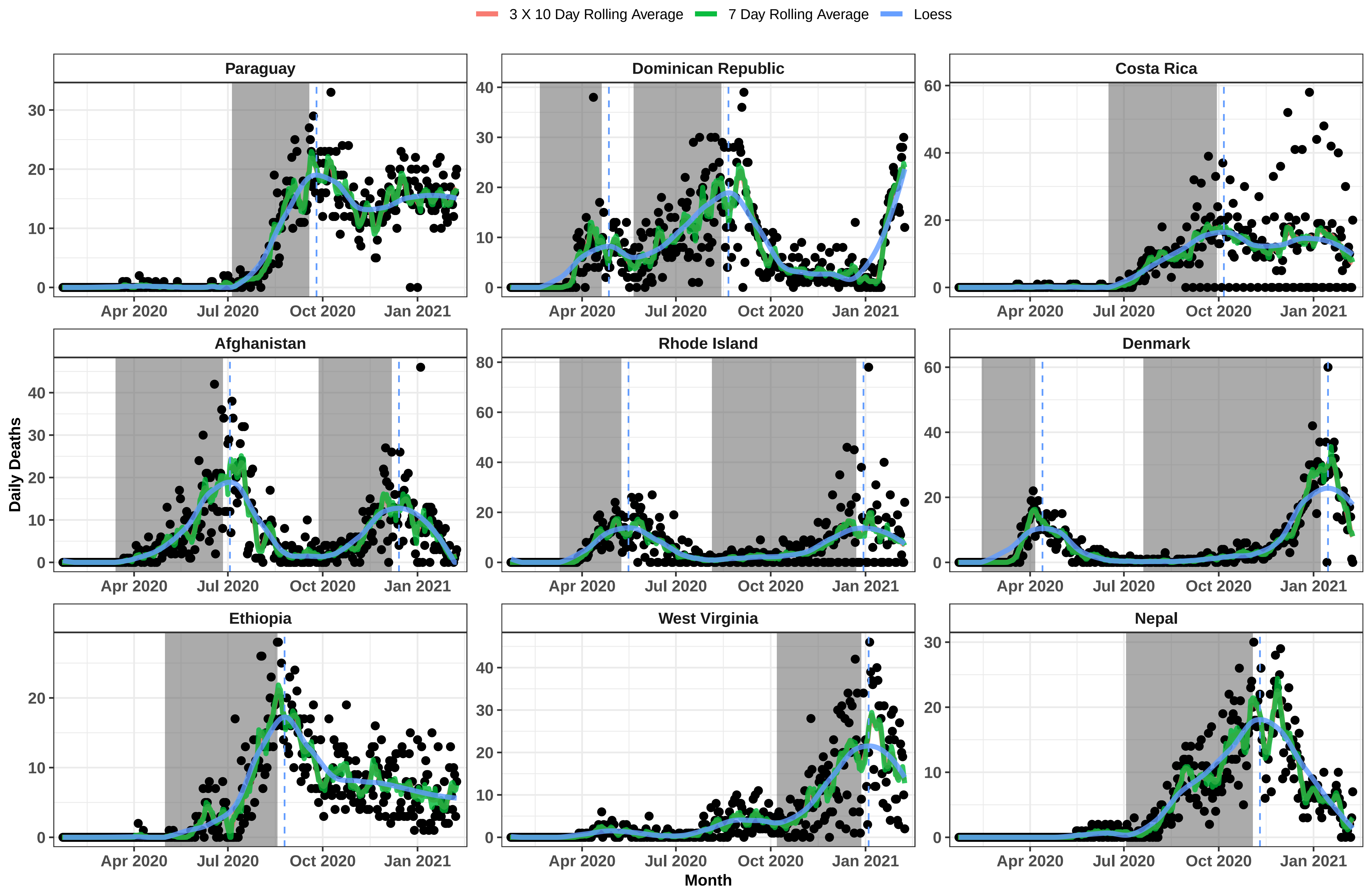

3 X 10 Day Rolling Average
7 Day Rolling Average
Loess
Paraguay
Dominican Republic
Costa Rica
Afghanistan
Rhode Island
Denmark
Ethiopia
West Virginia
Nepal
Daily Deaths
Month
Apr 2020
Jul 2020
Oct 2020
Jan 2021

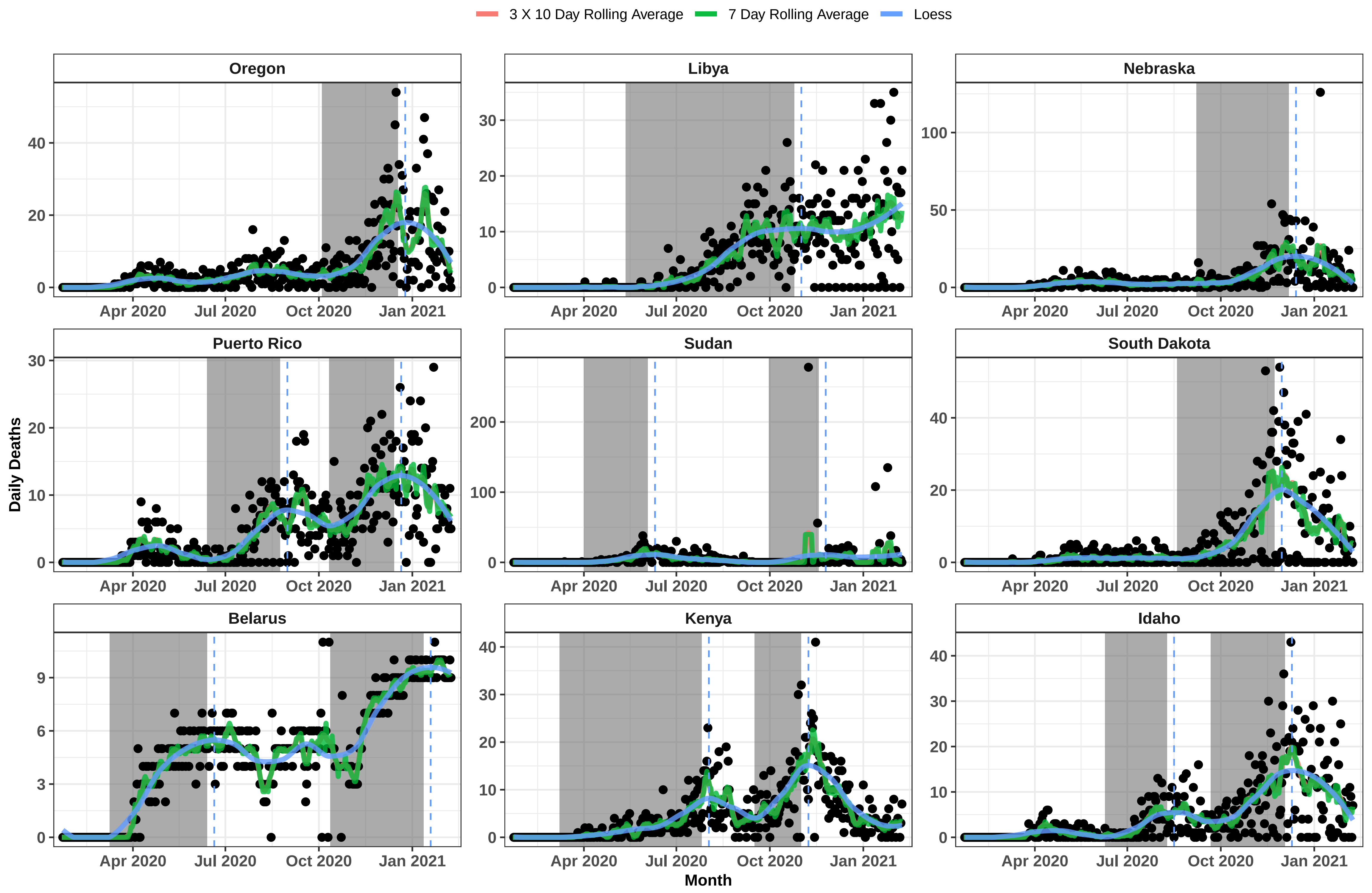

3 X 10 Day Rolling Average
7 Day Rolling Average
Loess
Oregon
Libya
Nebraska
Puerto Rico
Sudan
South Dakota
Belarus
Kenya
Idaho
Daily Deaths
Month
Apr 2020
Jul 2020
Oct 2020
Jan 2021

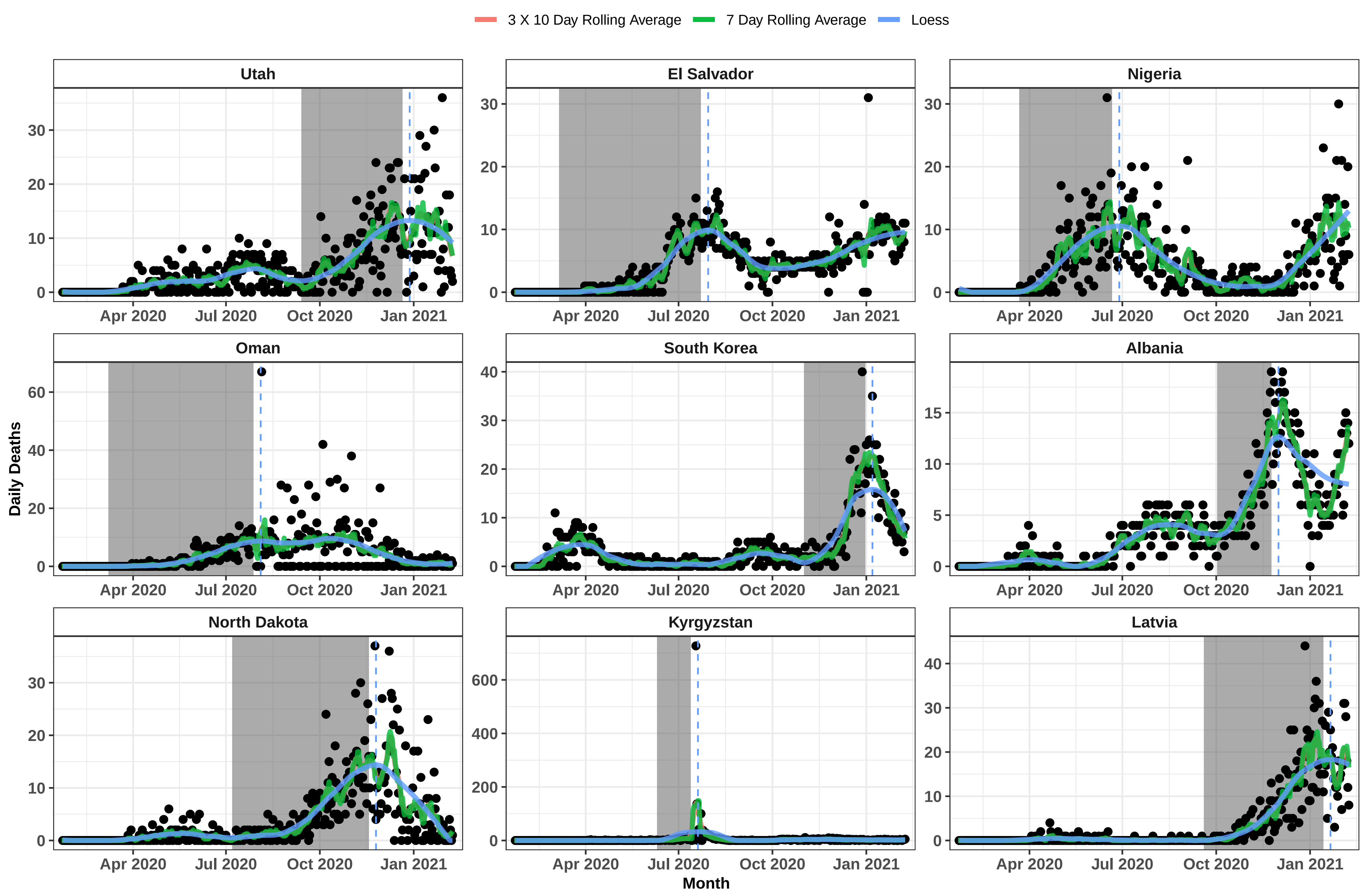

3 X 10 Day Rolling Average
7 Day Rolling Average
Loess
Utah
El Salvador
Nigeria
Oman
South Korea
Albania
North Dakota
Kyrgyzstan
Latvia
Daily Deaths
Month
Apr 2020
Jul 2020
Oct 2020
Jan 2021

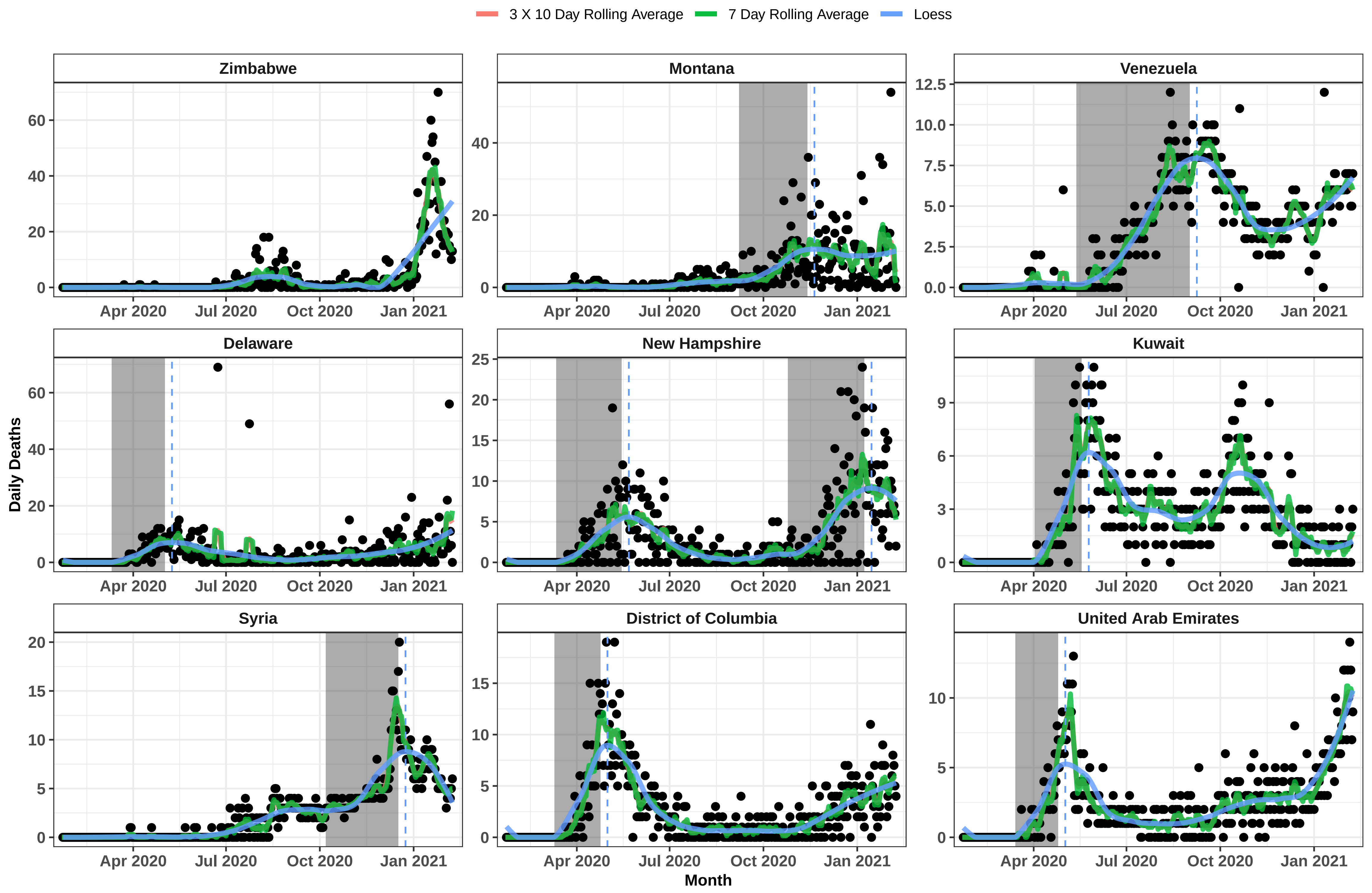

3 X 10 Day Rolling Average
7 Day Rolling Average
Loess
Zimbabwe
Montana
Venezuela
Delaware
New Hampshire
Kuwait
Syria
District of Columbia
United Arab Emirates
Daily Deaths
Month
Apr 2020
Jul 2020
Oct 2020
Jan 2021

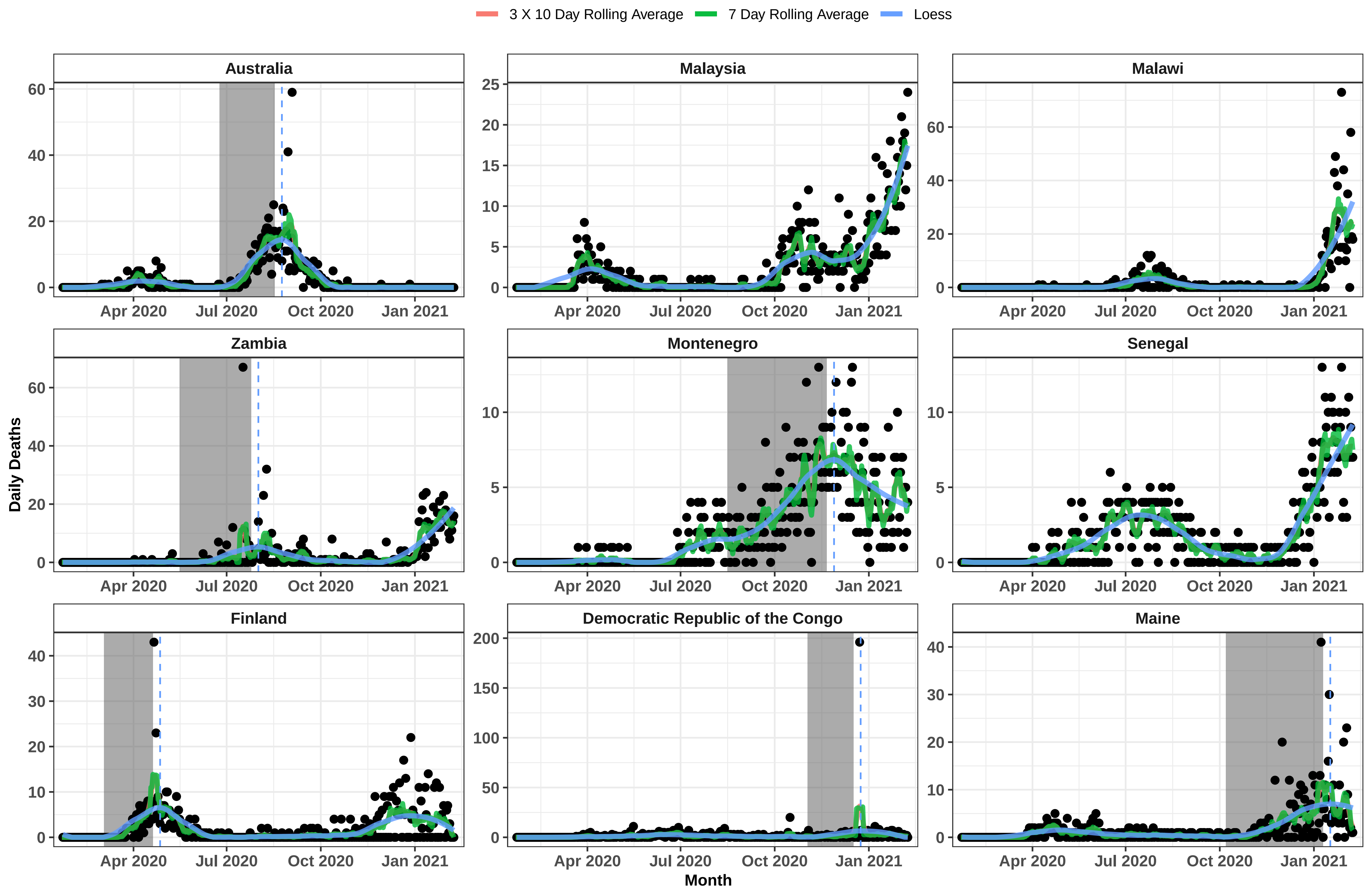

3 X 10 Day Rolling Average
7 Day Rolling Average
Loess
Australia
Malaysia
Malawi
Zambia
Montenegro
Senegal
Finland
Democratic Republic of the Congo
Maine
Daily Deaths
Month
Apr 2020
Jul 2020
Oct 2020
Jan 2021

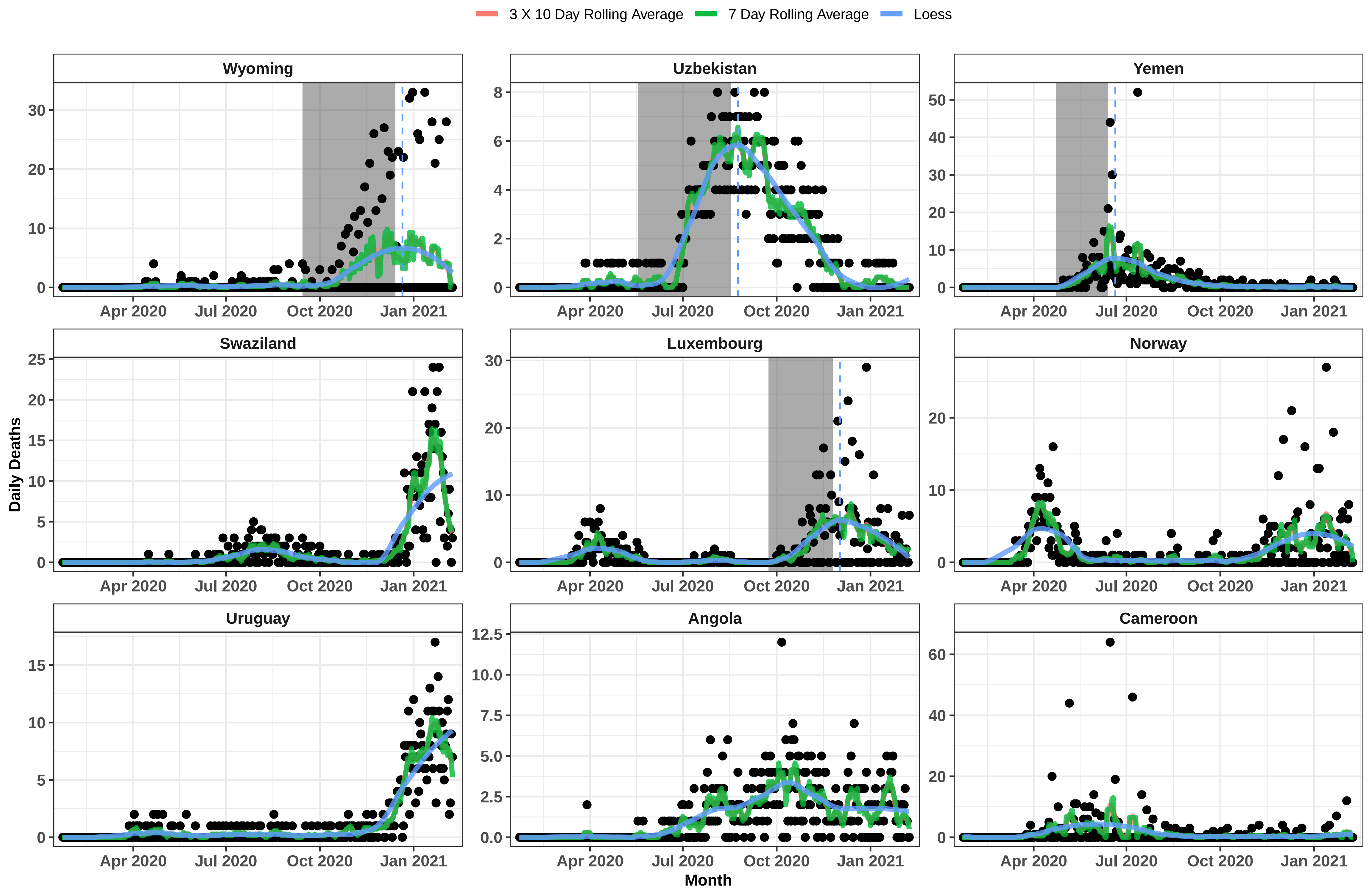

3 X 10 Day Rolling Average
7 Day Rolling Average
Loess
Wyoming
Uzbekistan
Yemen
Swaziland
Luxembourg
Norway
Uruguay
Angola
Cameroon
Daily Deaths
Month
Apr 2020
Jul 2020
Oct 2020
Jan 2021

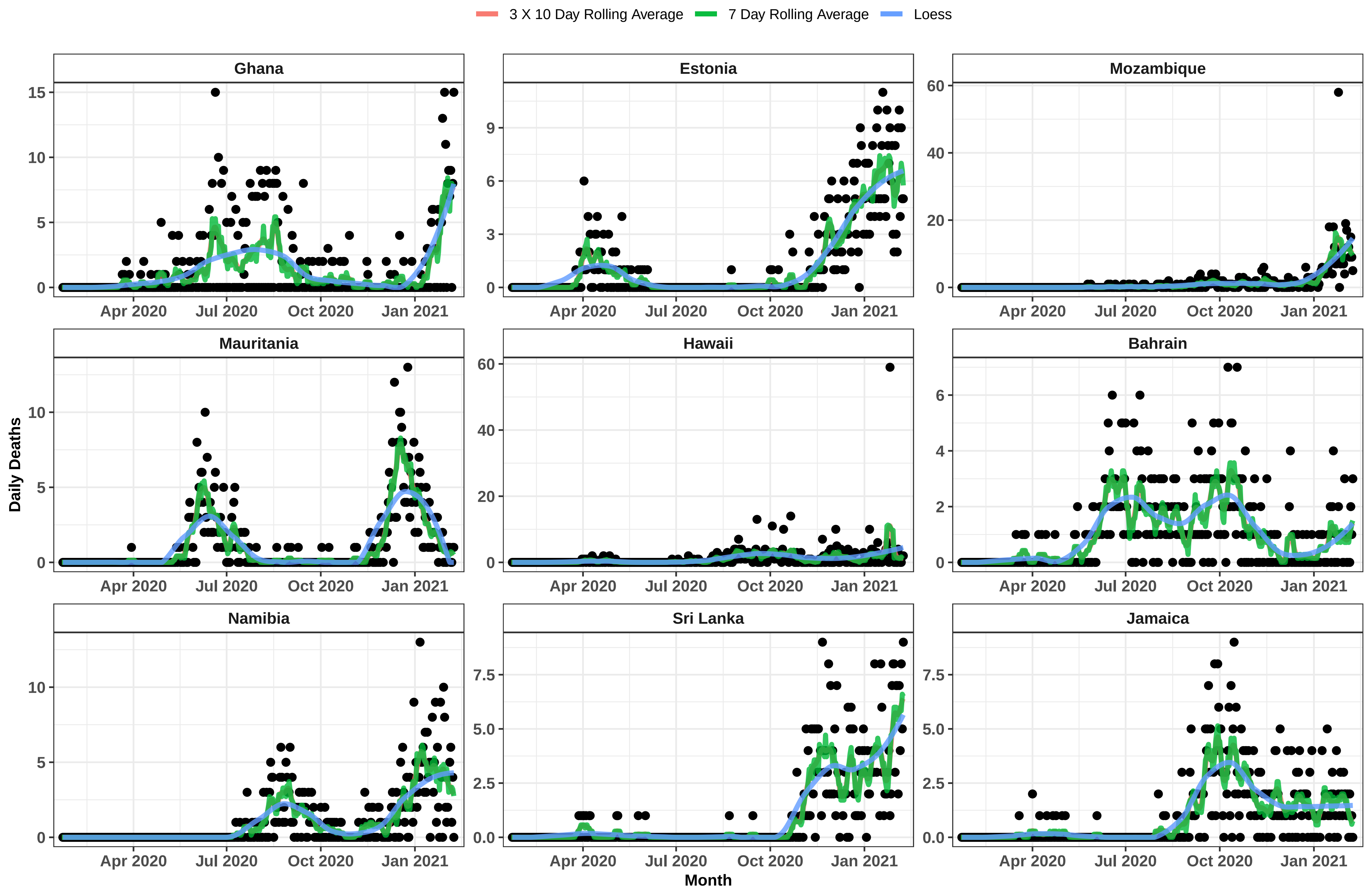
3 X 10 Day Rolling Average
7 Day Rolling Average
Loess
Ghana
Estonia
Mozambique
Mauritania
Hawaii
Bahrain
Namibia
Sri Lanka
Jamaica
Daily Deaths
Month
Apr 2020
Jul 2020
Oct 2020
Jan 2021

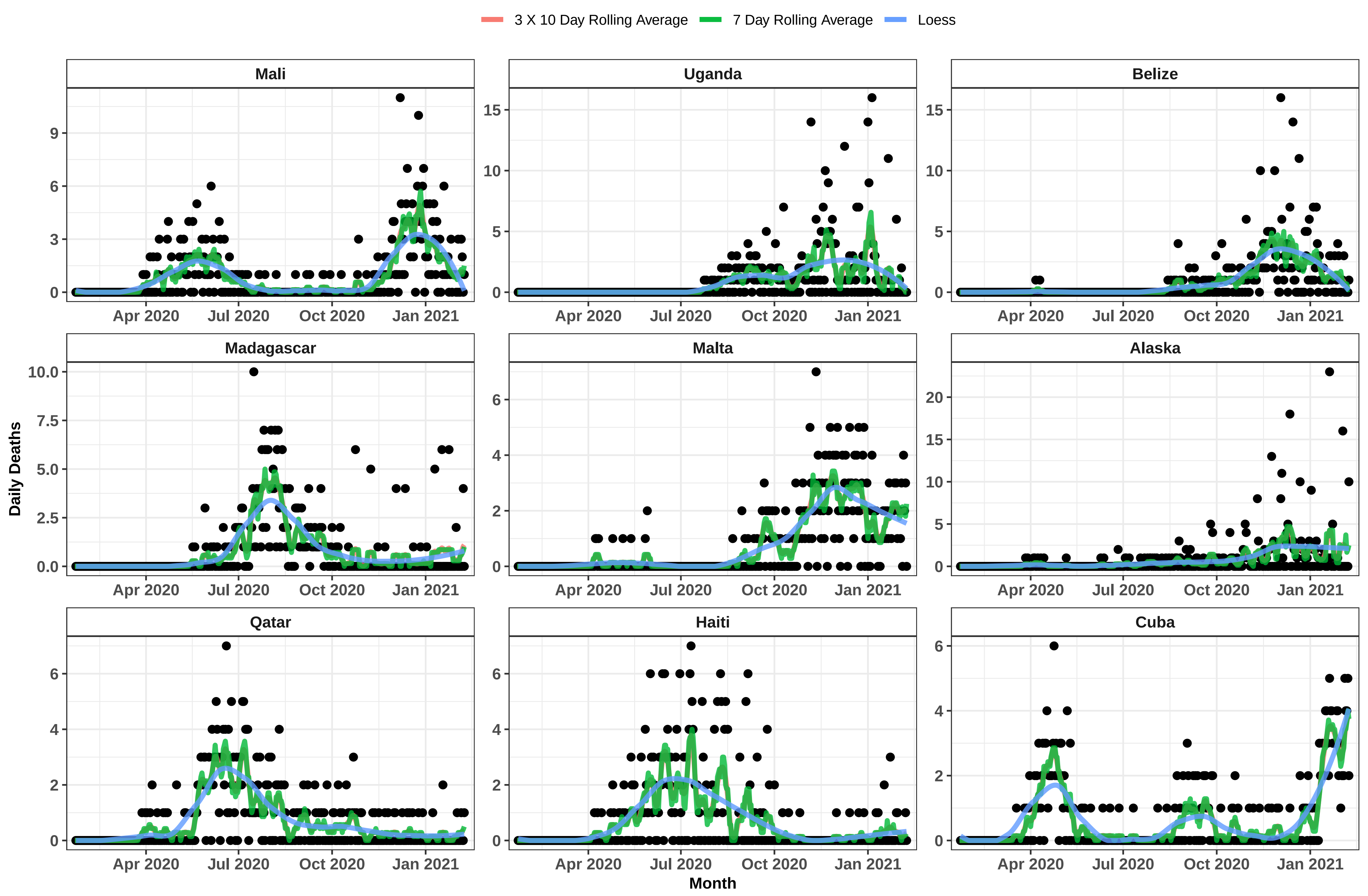

3 X 10 Day Rolling Average
7 Day Rolling Average
Loess
Mali
Uganda
Belize
Madagascar
Malta
Alaska
Qatar
Haiti
Cuba
Daily Deaths
Month
Apr 2020
Jul 2020
Oct 2020
Jan 2021

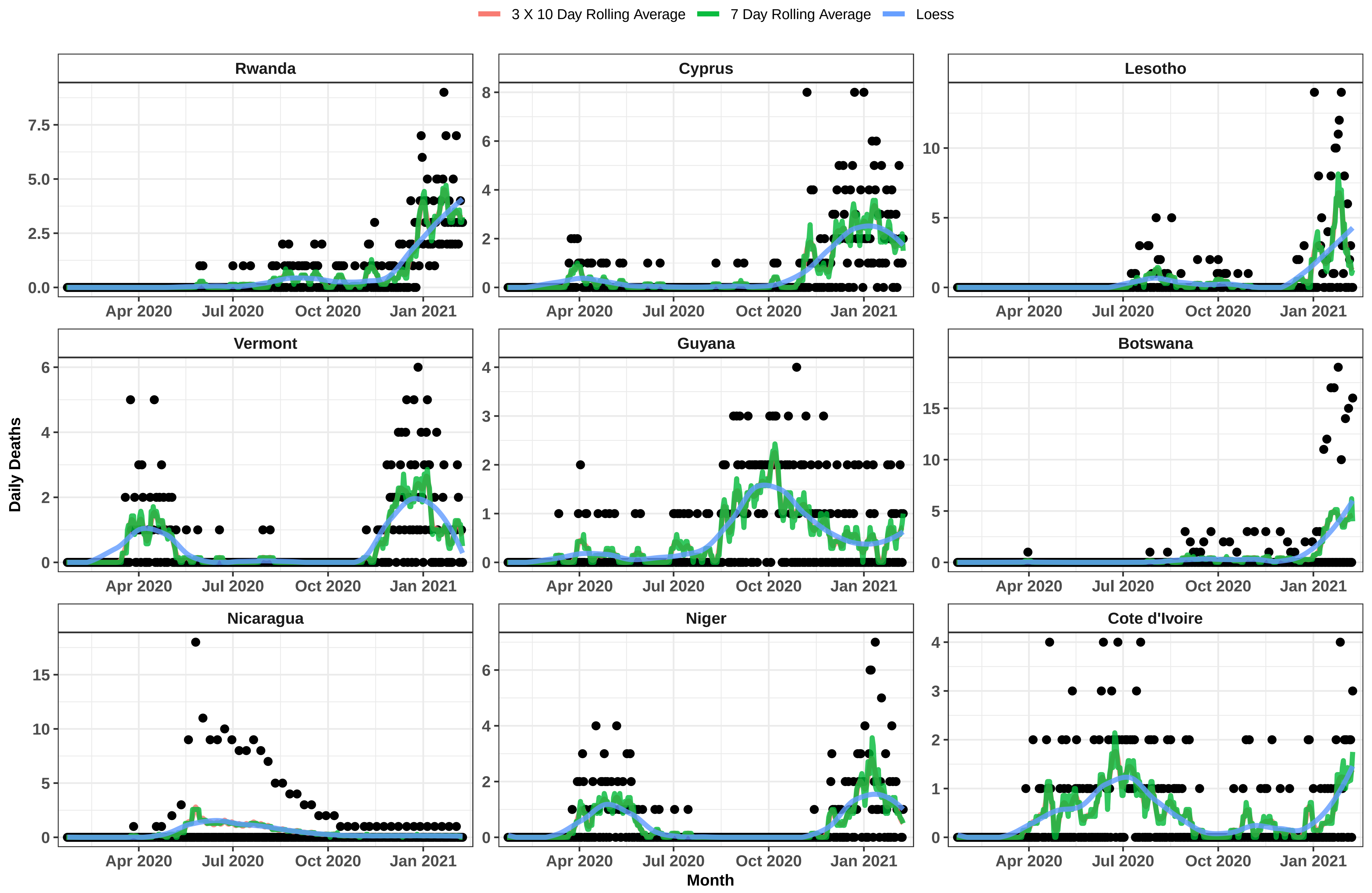

3 X 10 Day Rolling Average
7 Day Rolling Average
Loess
Rwanda
Cyprus
Lesotho
Vermont
Guyana
Botswana
Nicaragua
Niger
Cote d'Ivoire
Daily Deaths
Month
Apr 2020
Jul 2020
Oct 2020
Jan 2021

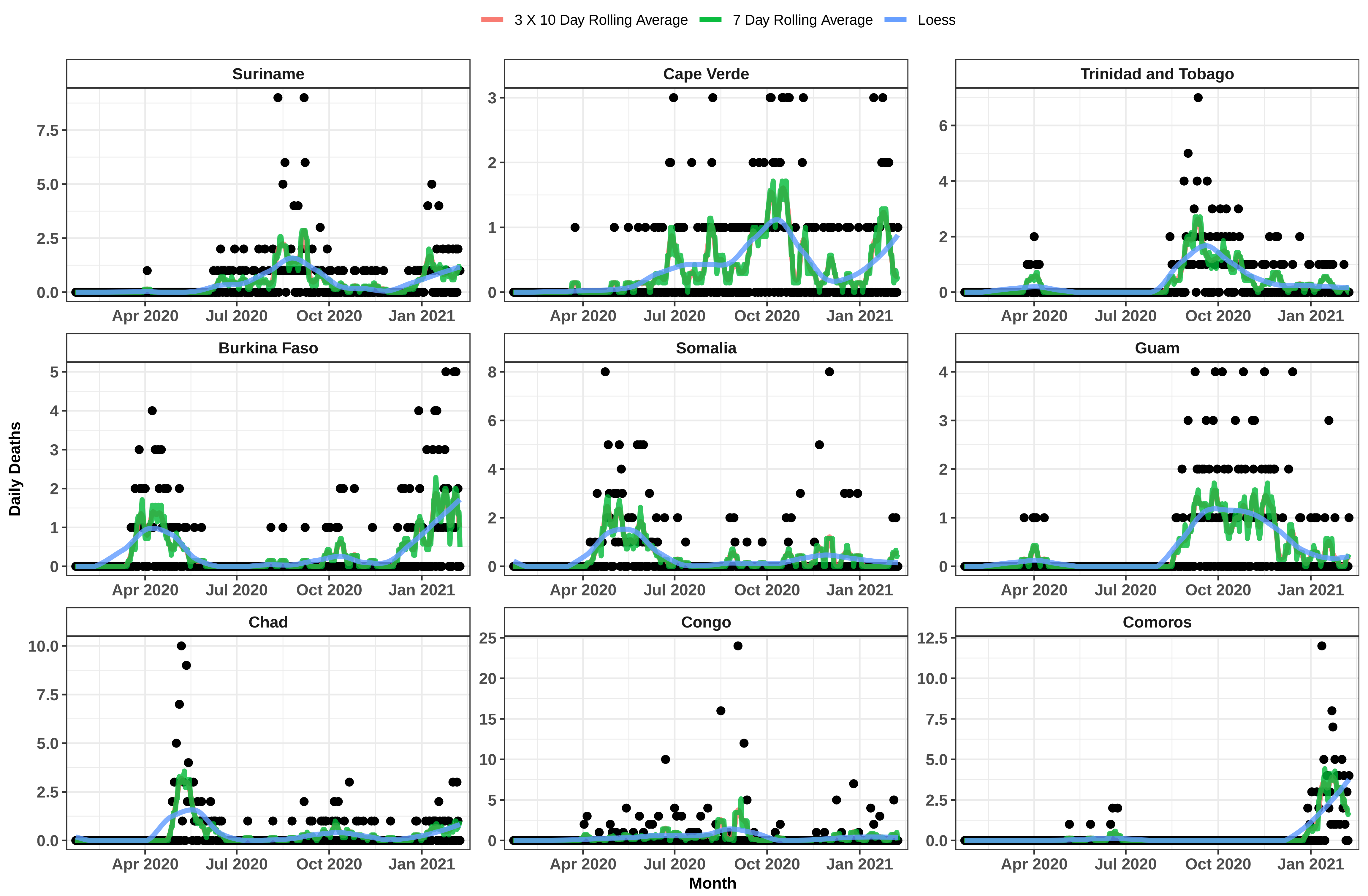
3 X 10 Day Rolling Average
7 Day Rolling Average
Loess
Suriname
Cape Verde
Trinidad and Tobago
Burkina Faso
Somalia
Guam
Chad
Congo
Comoros
Daily Deaths
Month
Apr 2020
Jul 2020
Oct 2020
Jan 2021

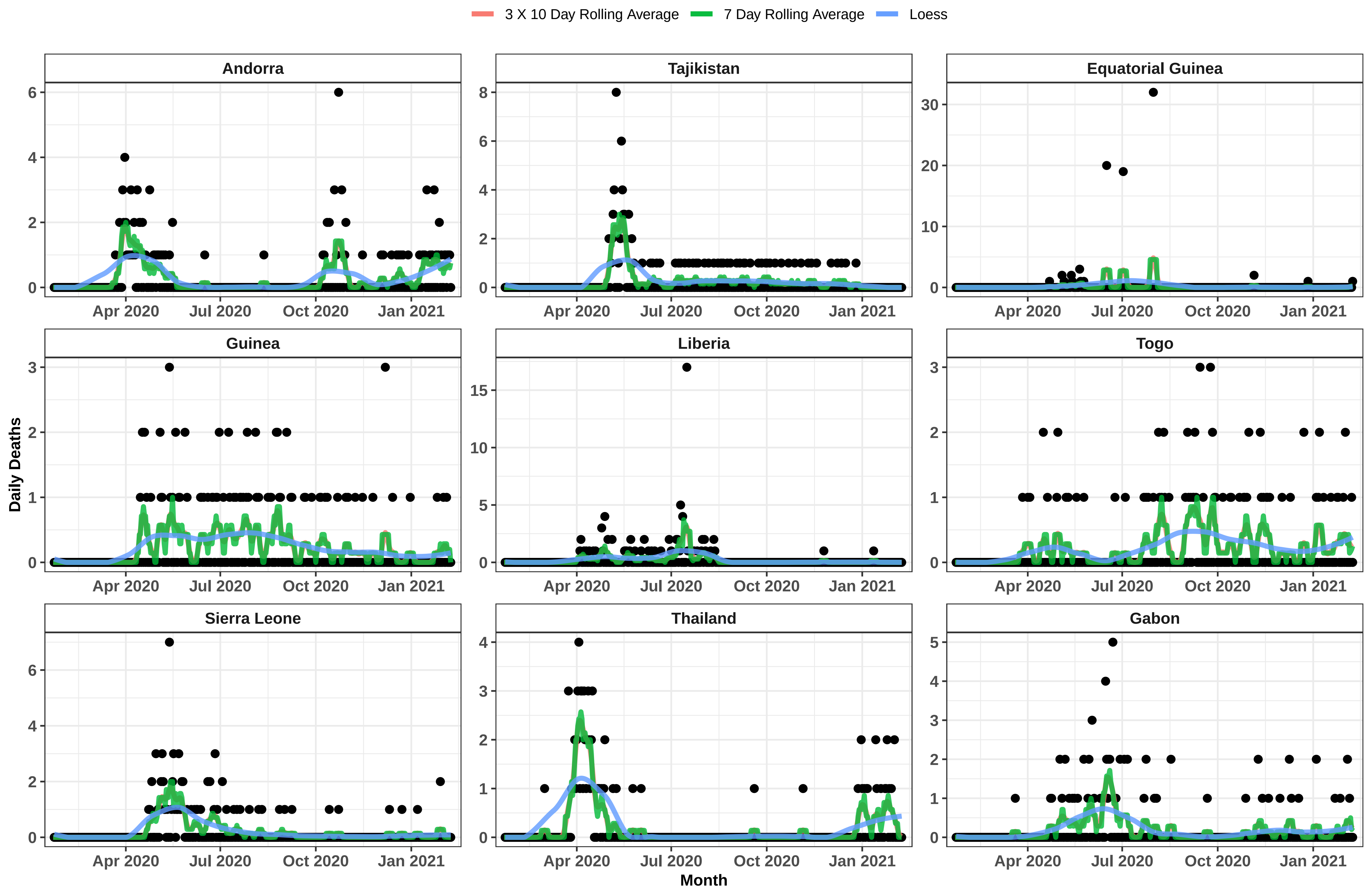

3 X 10 Day Rolling Average
7 Day Rolling Average
Loess
Andorra
Tajikistan
Equatorial Guinea
Guinea
Liberia
Togo
Sierra Leone
Thailand
Gabon
Daily Deaths
Month
Apr 2020
Jul 2020
Oct 2020
Jan 2021

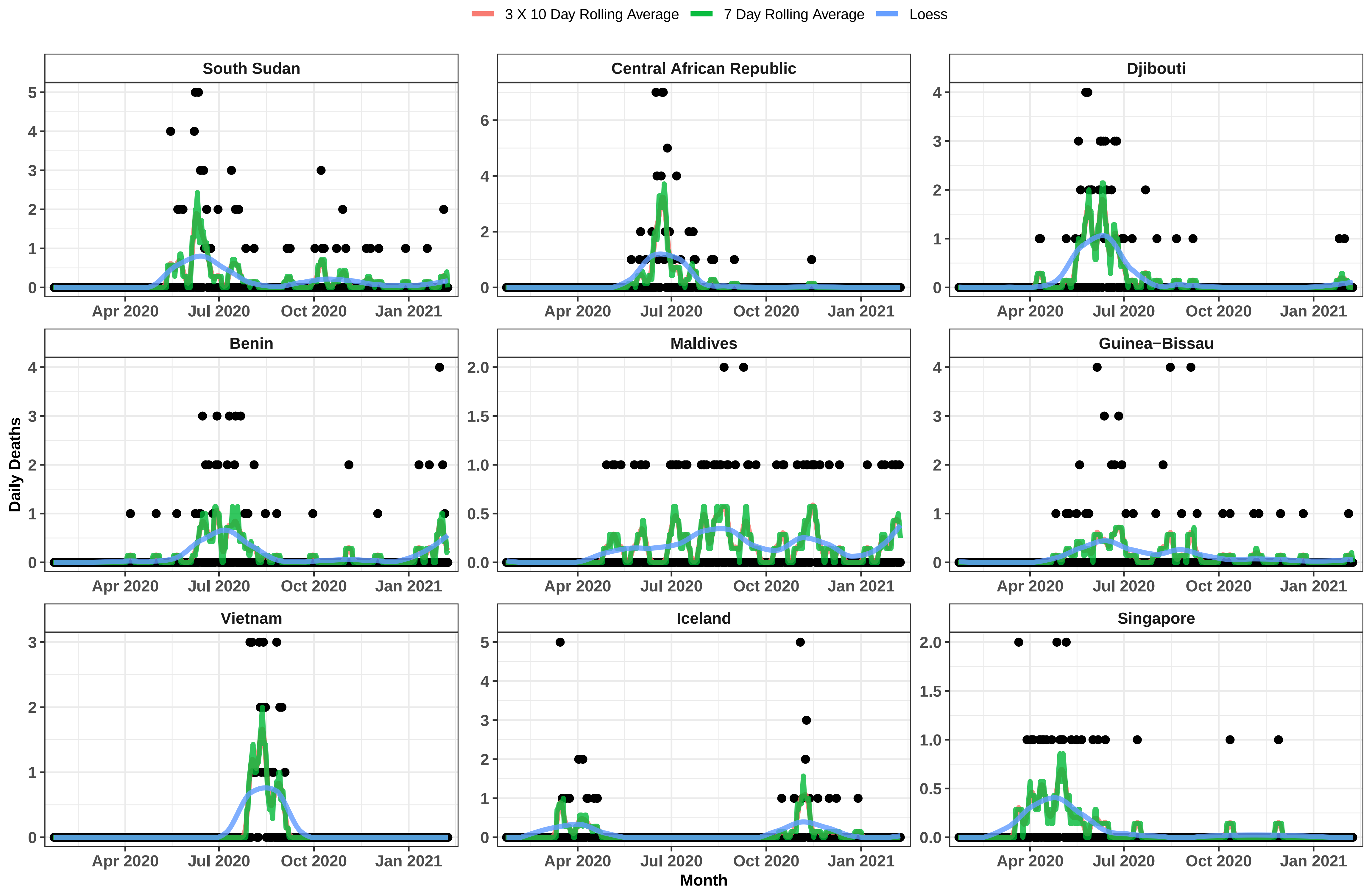

3 X 10 Day Rolling Average
7 Day Rolling Average
Loess
South Sudan
Central African Republic
Djibouti
Benin
Maldives
Guinea−Bissau
Vietnam
Iceland
Singapore
Daily Deaths
Month
Apr 2020
Jul 2020
Oct 2020
Jan 2021

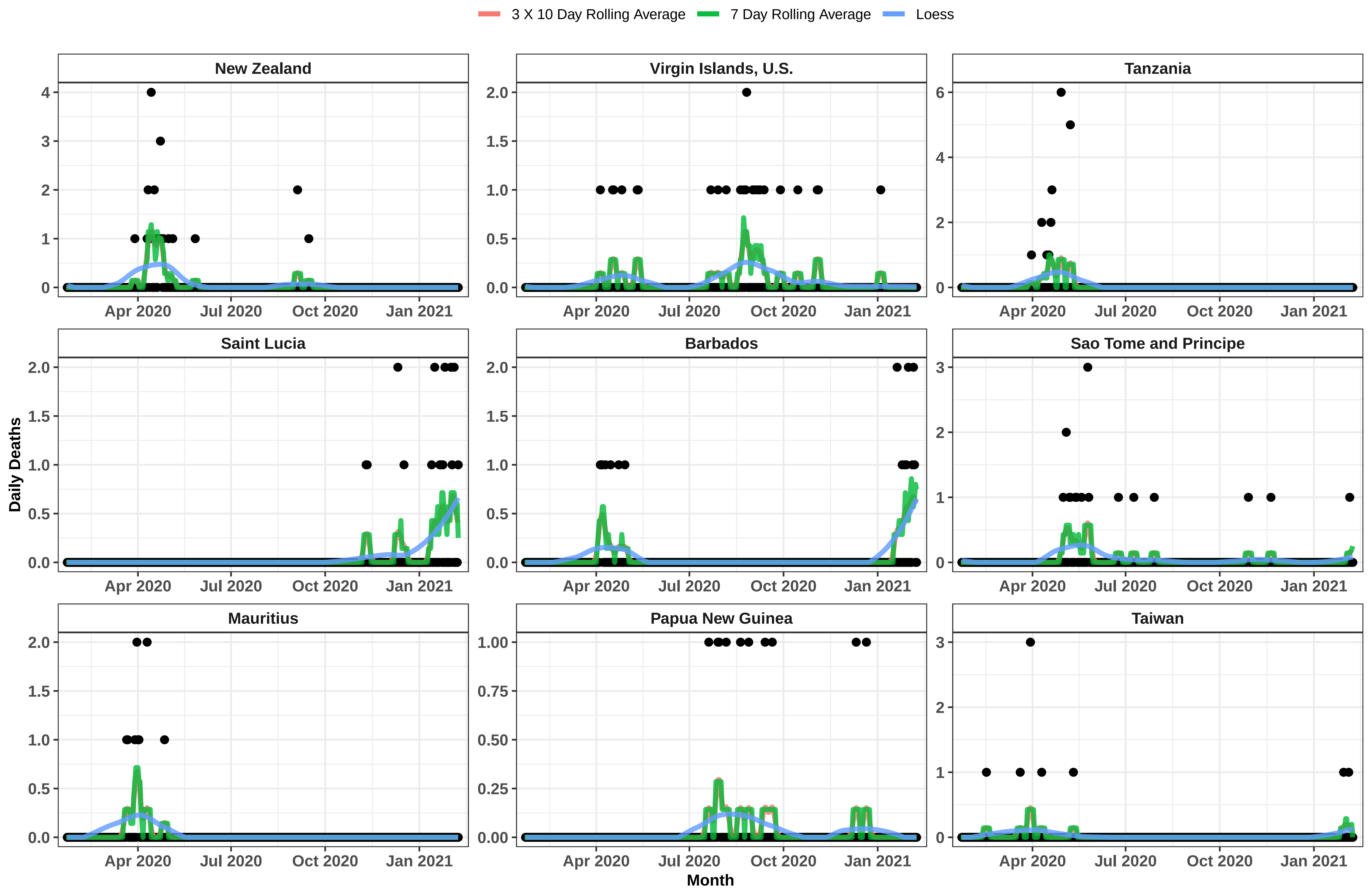

3 X 10 Day Rolling Average
7 Day Rolling Average
Loess
New Zealand
Virgin Islands, U.S.
Tanzania
Saint Lucia
Barbados
Sao Tome and Principe
Mauritius
Papua New Guinea
Taiwan
Daily Deaths
Month
Apr 2020
Jul 2020
Oct 2020
Jan 2021

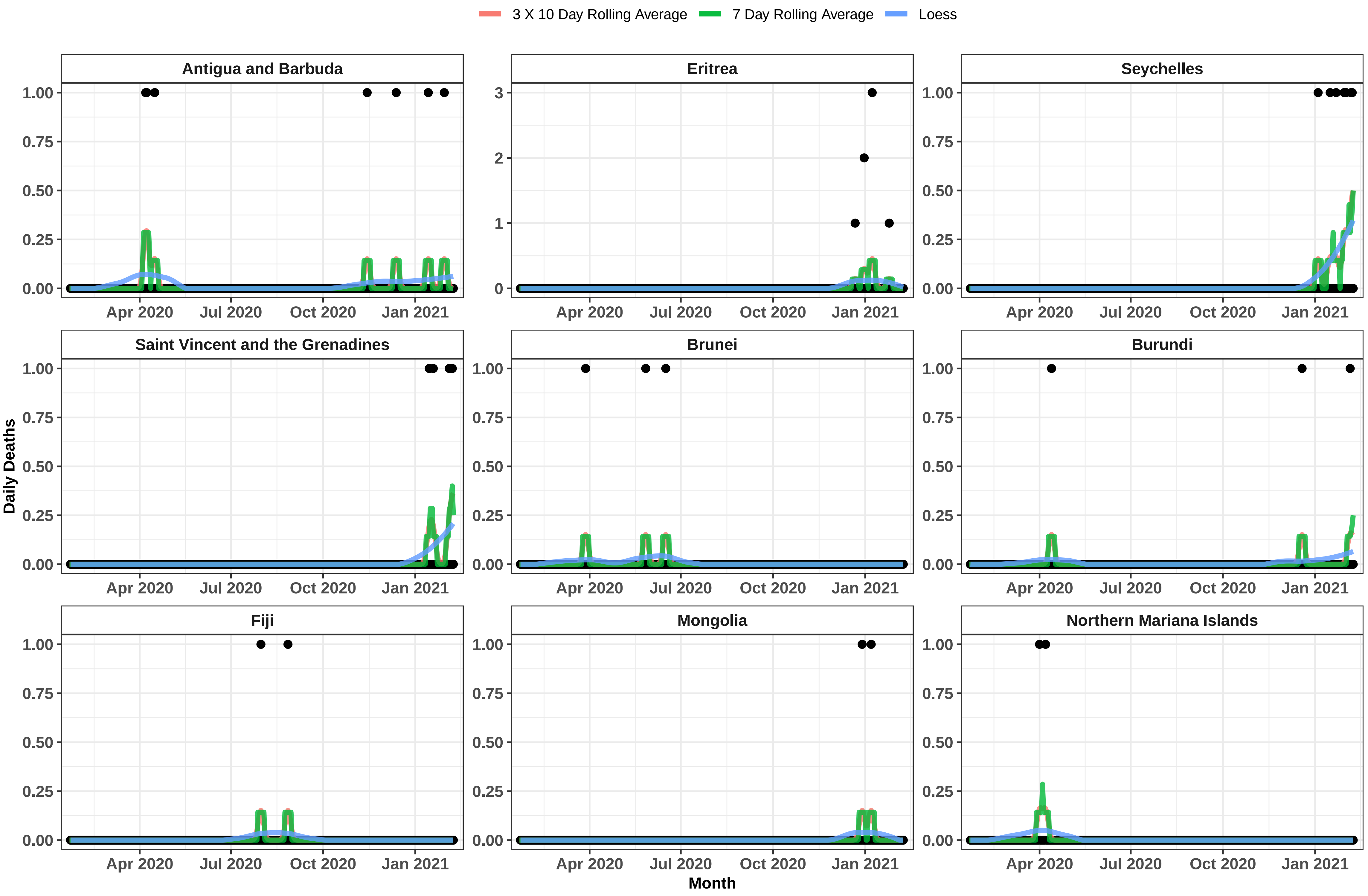

3 X 10 Day Rolling Average
7 Day Rolling Average
Loess
Antigua and Barbuda
Eritrea
Seychelles
Saint Vincent and the Grenadines
Brunei
Burundi
Fiji
Mongolia
Northern Mariana Islands
Daily Deaths
Month
Apr 2020
Jul 2020
Oct 2020
Jan 2021
0.00
0.25
0.50
0.75
1.00
0
1
2
3

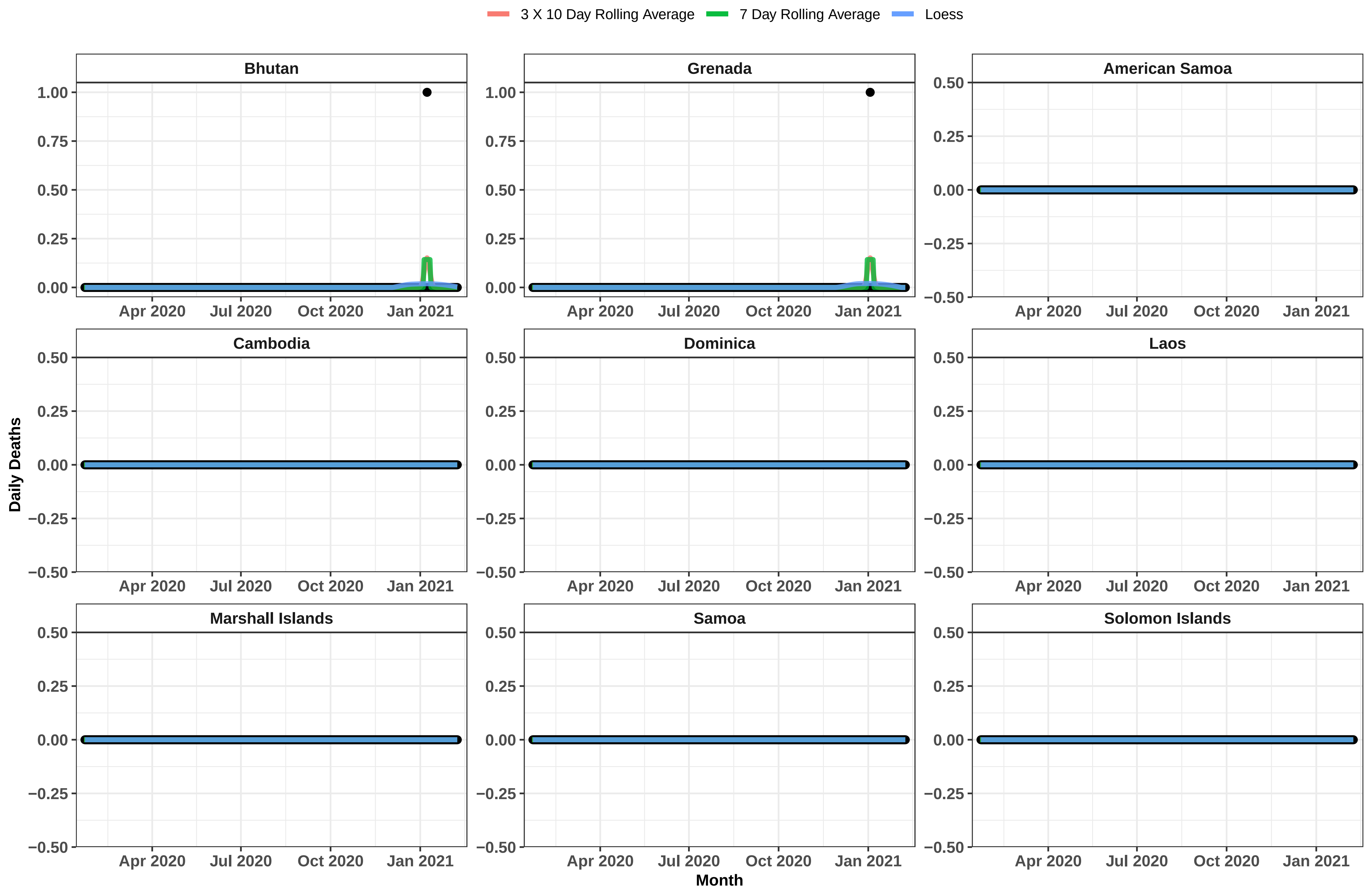
3 X 10 Day Rolling Average
7 Day Rolling Average
Loess
Bhutan
Grenada
American Samoa
Cambodia
Dominica
Laos
Marshall Islands
Samoa
Solomon Islands
Daily Deaths
Month
Apr 2020
Jul 2020
Oct 2020
Jan 2021

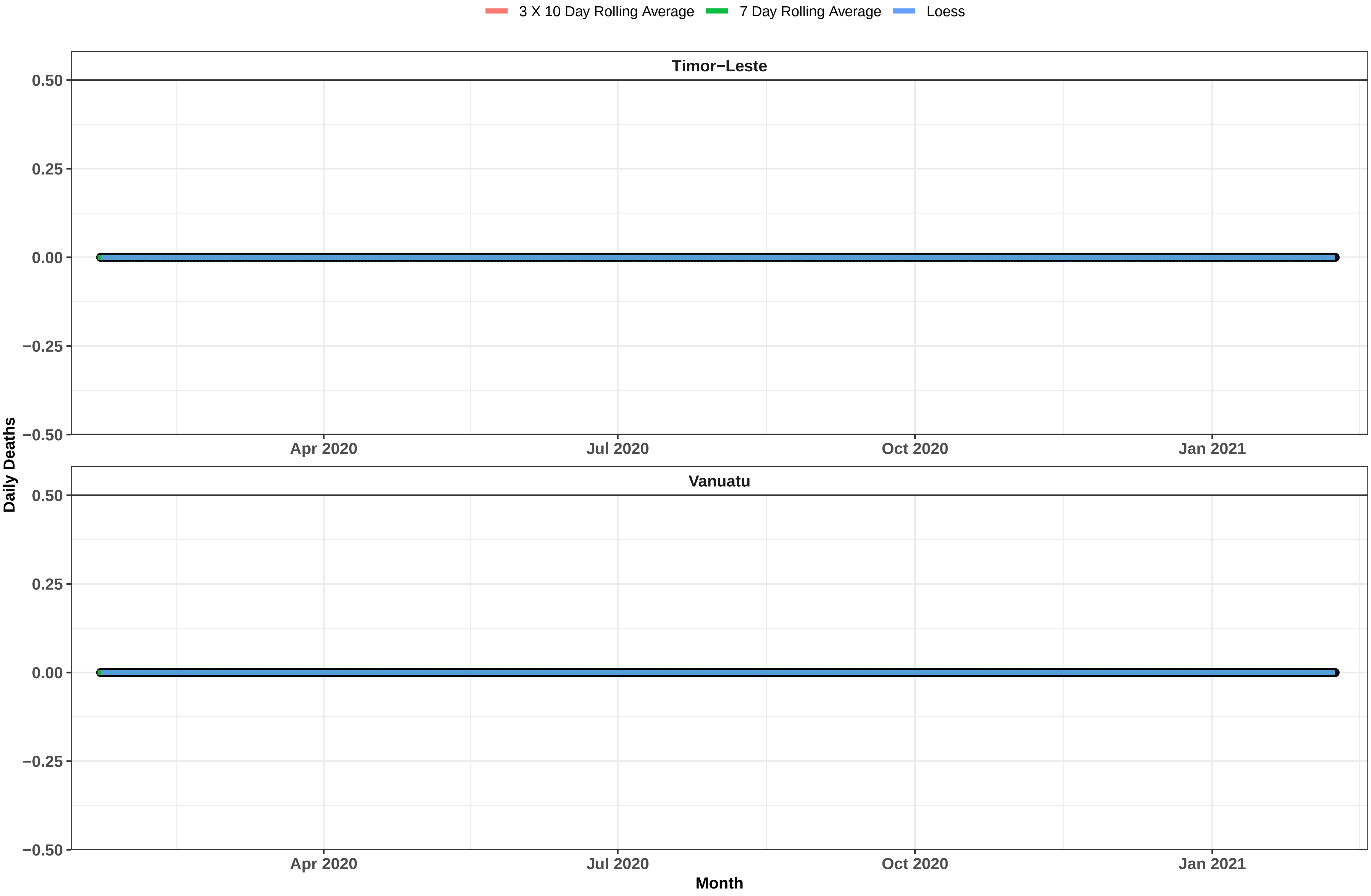
3 X 10 Day Rolling Average
7 Day Rolling Average
Loess
Timor−Leste
Vanuatu
Daily Deaths
0.50
0.25
0.00
−0.25
−0.50
Apr 2020
Jul 2020
Oct 2020
Jan 2021
Month

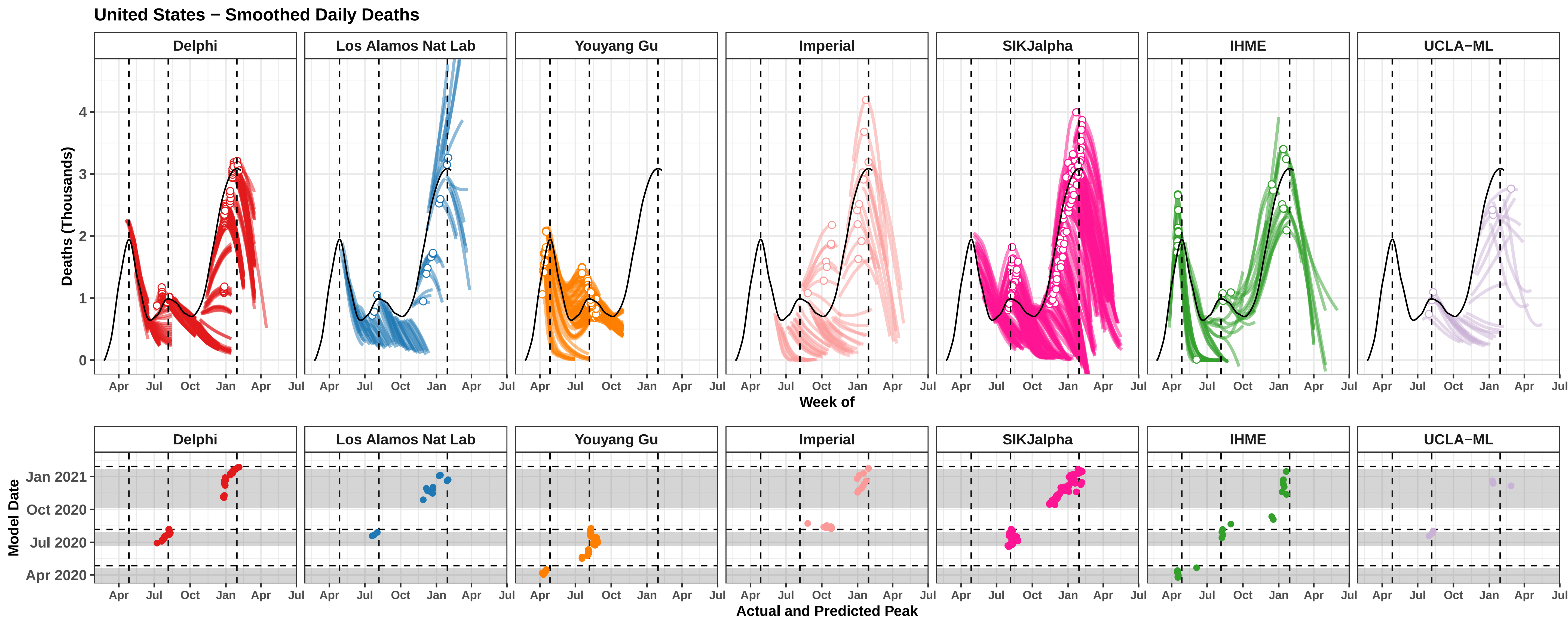
United States − Smoothed Daily Deaths
Delphi
Los Alamos Nat Lab
Youyang Gu
Imperial
SIKJalpha
IHME
UCLA−ML
Deaths (Thousands)
0
1
2
3
4
Apr
Jul
Oct
Jan
Apr
Jul
Week of
Model Date
Apr 2020
Jul 2020
Oct 2020
Jan 2021
Actual and Predicted Peak

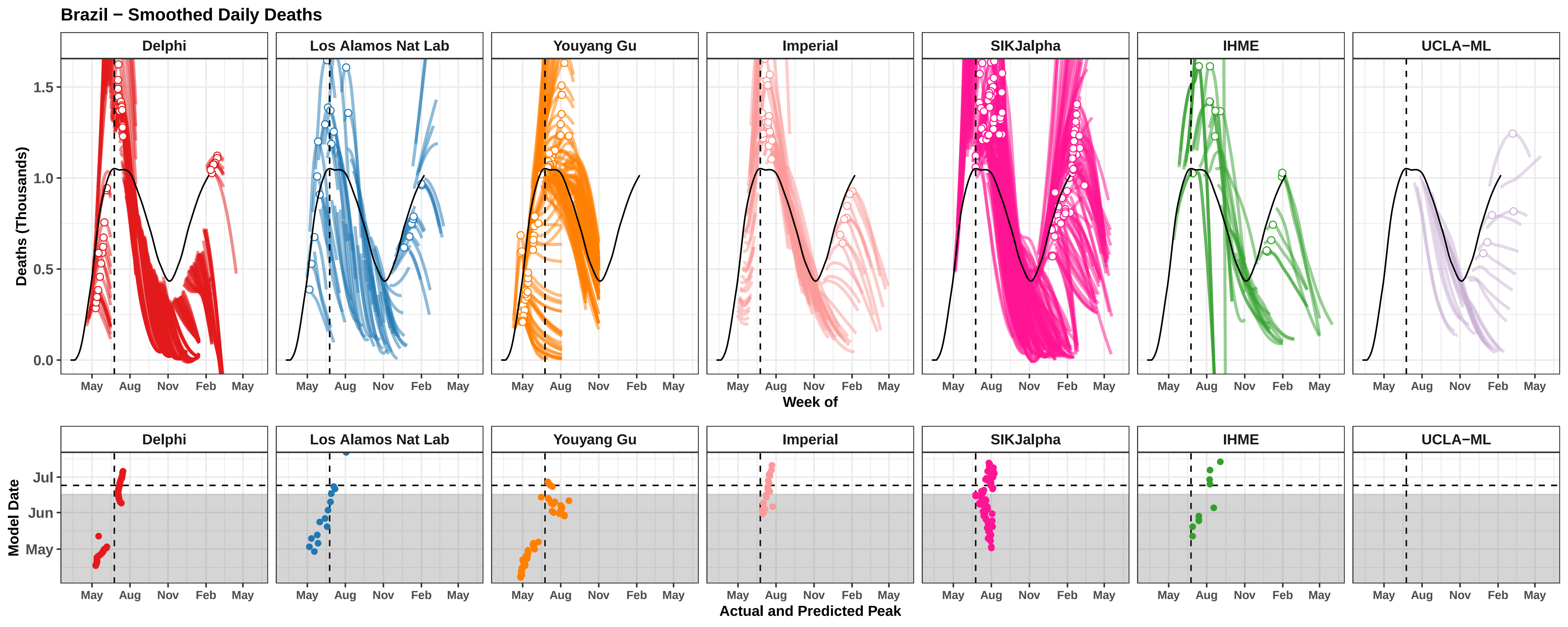
Brazil − Smoothed Daily Deaths
Delphi
Los Alamos Nat Lab
Youyang Gu
Imperial
SIKJalpha
IHME
UCLA−ML
Deaths (Thousands)
1.5
1.0
0.5
0.0
May
Aug
Nov
Feb
May
Week of
Model Date
Jul
Jun
May
Actual and Predicted Peak

Mexico − Smoothed Daily Deaths

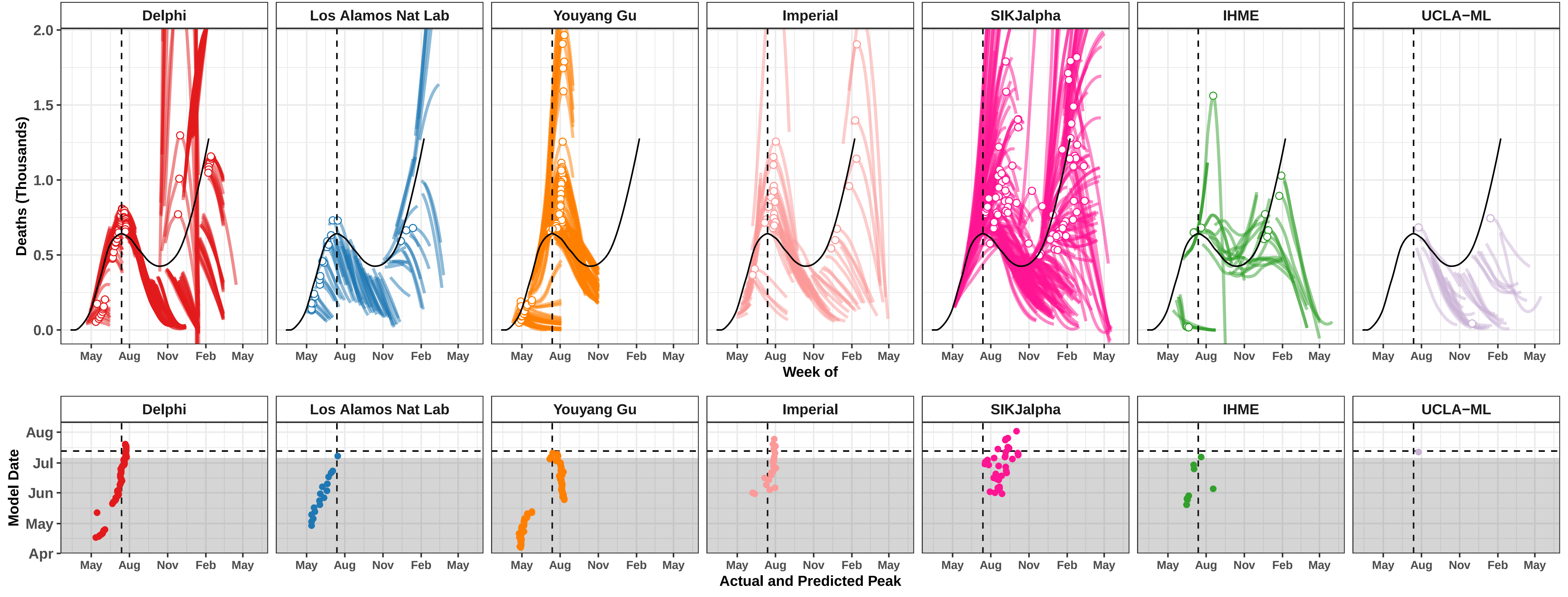

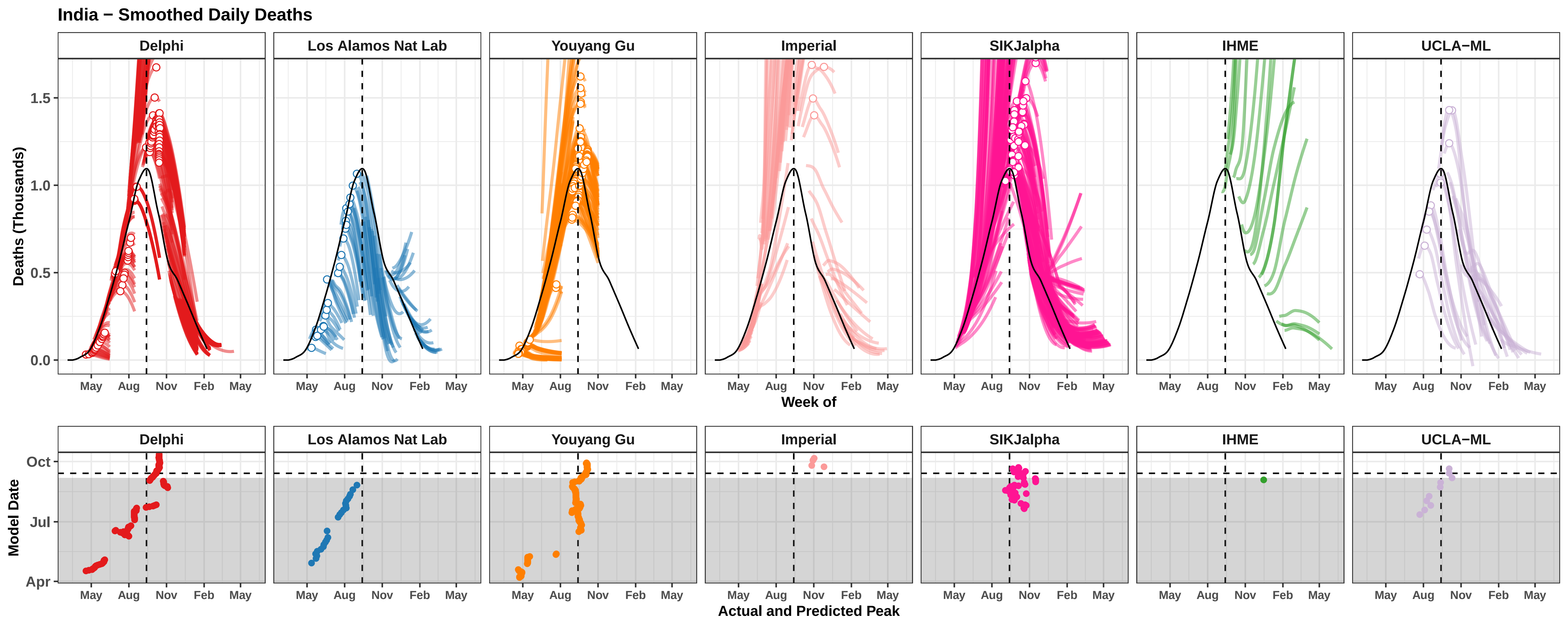

India − Smoothed Daily Deaths
Delphi
Los Alamos Nat Lab
Youyang Gu
Imperial
SIKJalpha
IHME
UCLA−ML
Deaths (Thousands)
1.5
1.0
0.5
0.0
May
Aug
Nov
Feb
May
Week of
Model Date
Oct
Jul
Apr
Actual and Predicted Peak

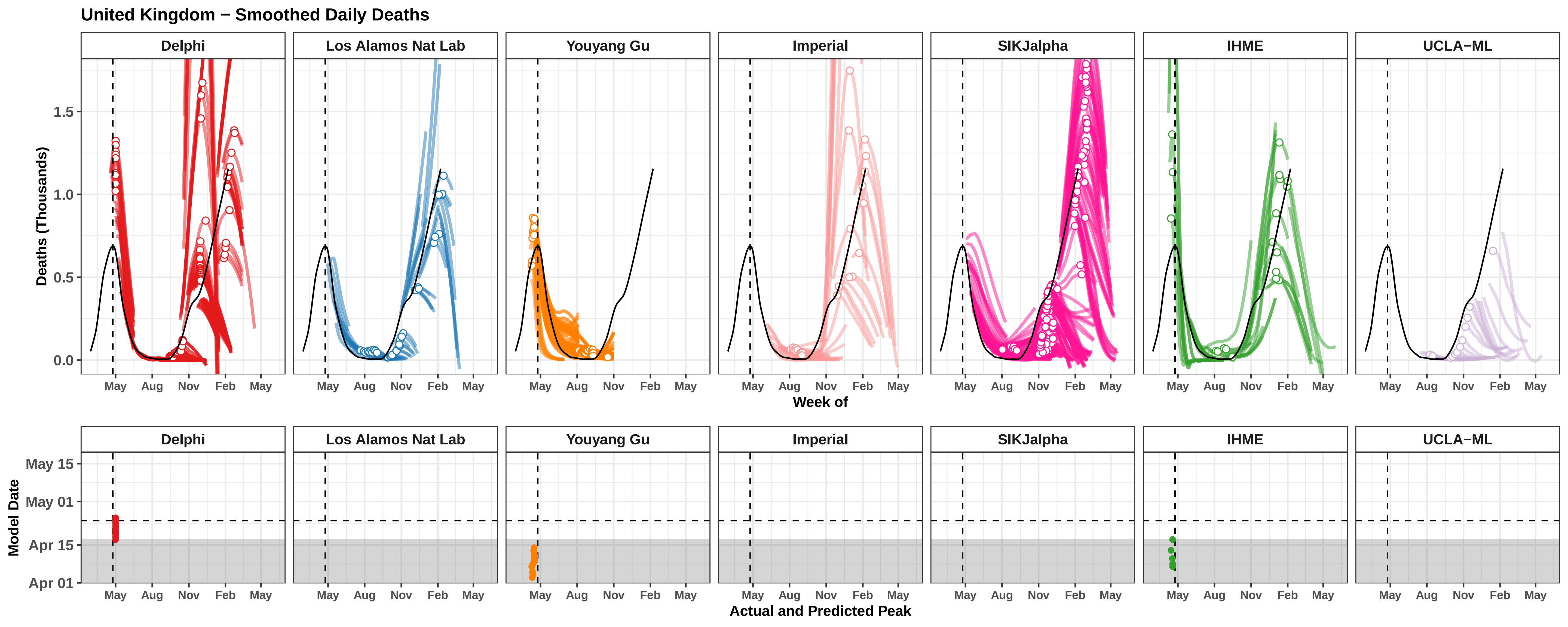

United Kingdom − Smoothed Daily Deaths
Delphi
Los Alamos Nat Lab
Youyang Gu
Imperial
SIKJalpha
IHME
UCLA−ML
Deaths (Thousands)
0.0
0.5
1.0
1.5
May
Aug
Nov
Feb
May
Week of
Model Date
May 15
May 01
Apr 15
Apr 01
Actual and Predicted Peak

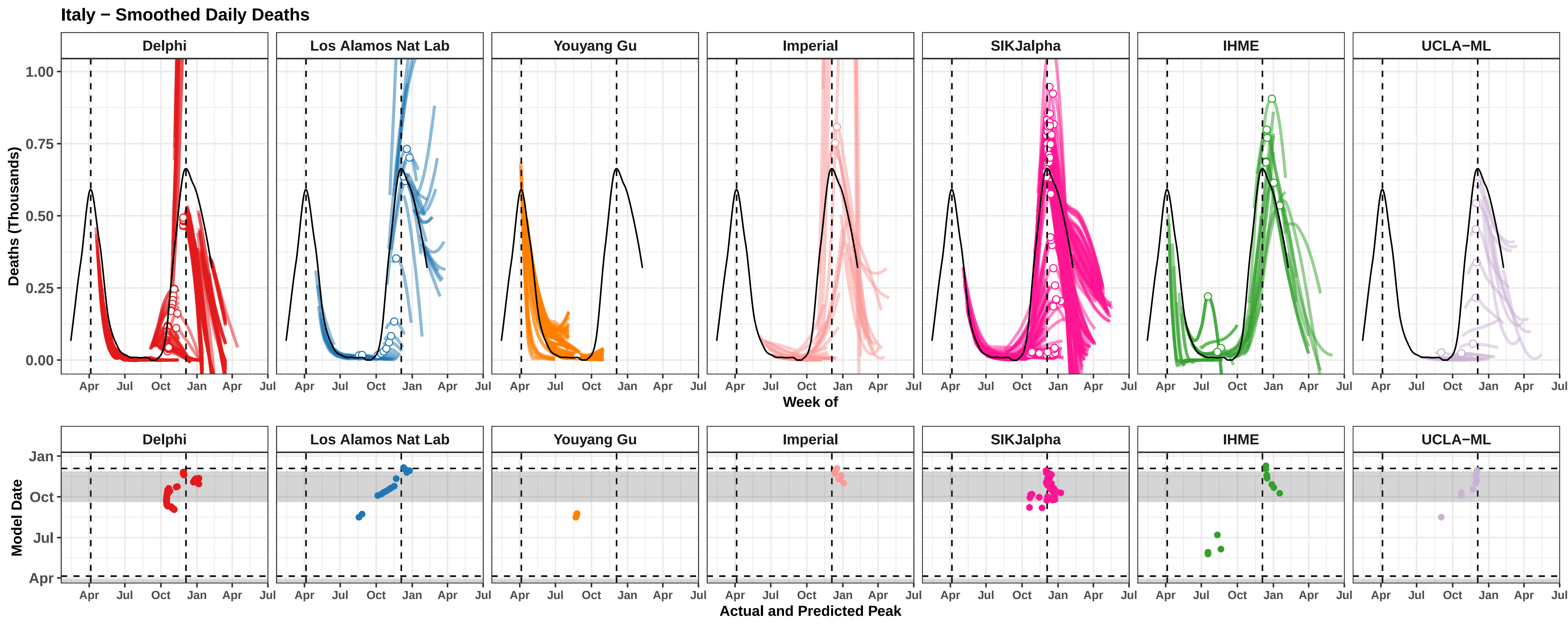

Italy − Smoothed Daily Deaths
Delphi
Los Alamos Nat Lab
Youyang Gu
Imperial
SIKJalpha
IHME
UCLA−ML
Deaths (Thousands)
1.00
0.75
0.50
0.25
0.00
Apr Jul Oct Jan Apr Jul
Week of
Model Date
Jan
Oct
Jul
Apr
Actual and Predicted Peak

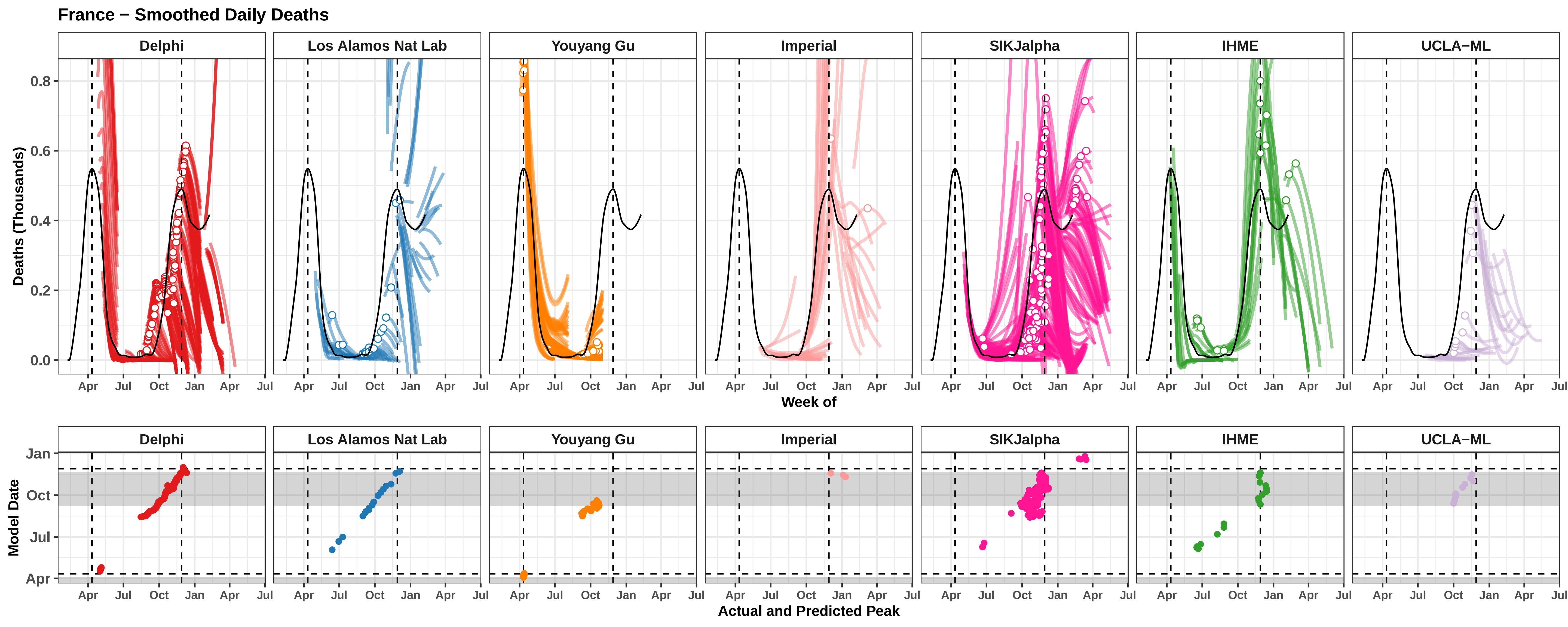
France − Smoothed Daily Deaths
Delphi
Los Alamos Nat Lab
Youyang Gu
Imperial
SIKJalpha
IHME
UCLA−ML
Deaths (Thousands)
0.0
0.2
0.4
0.6
0.8
Apr
Jul
Oct
Jan
Week of
Model Date
Actual and Predicted Peak

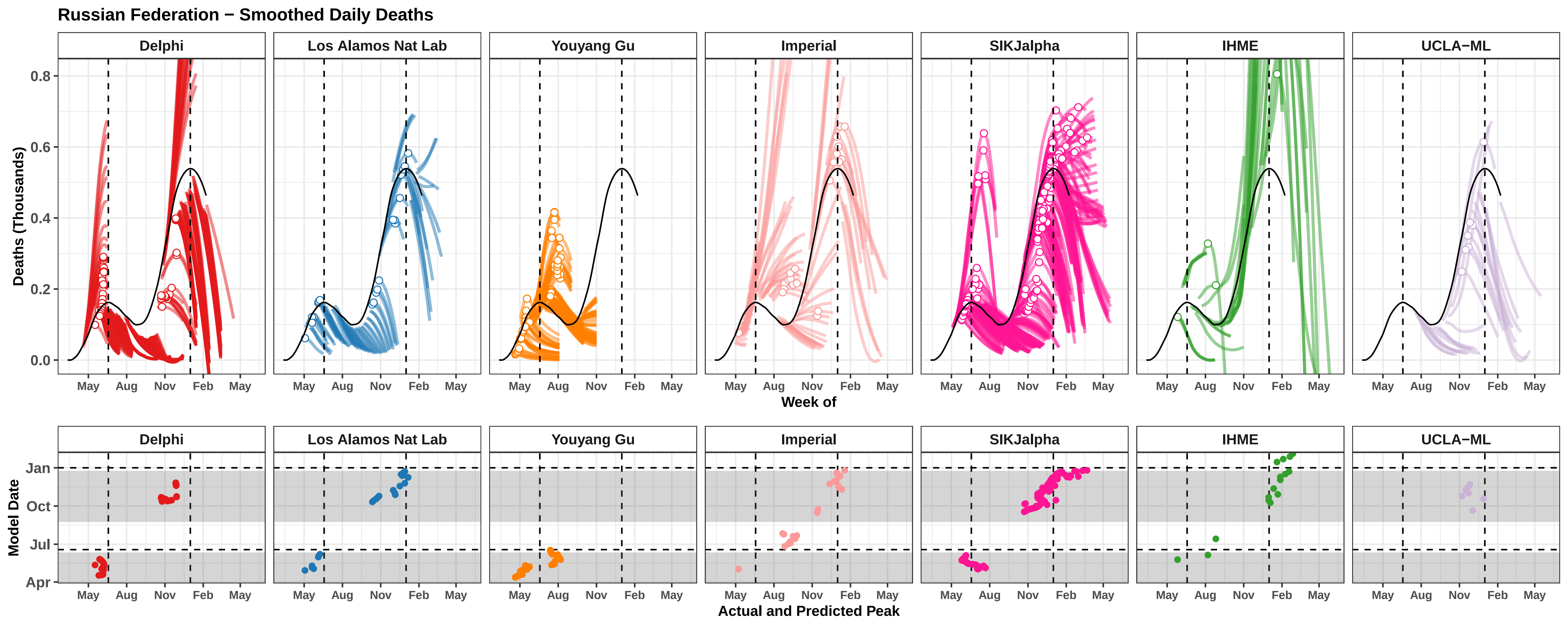

Russian Federation − Smoothed Daily Deaths
Delphi
Los Alamos Nat Lab
Youyang Gu
Imperial
SIKJalpha
IHME
UCLA−ML
Deaths (Thousands)
0.0
0.2
0.4
0.6
0.8
May
Aug
Nov
Feb
May
Week of
Model Date
Apr
Jul
Oct
Jan
Actual and Predicted Peak

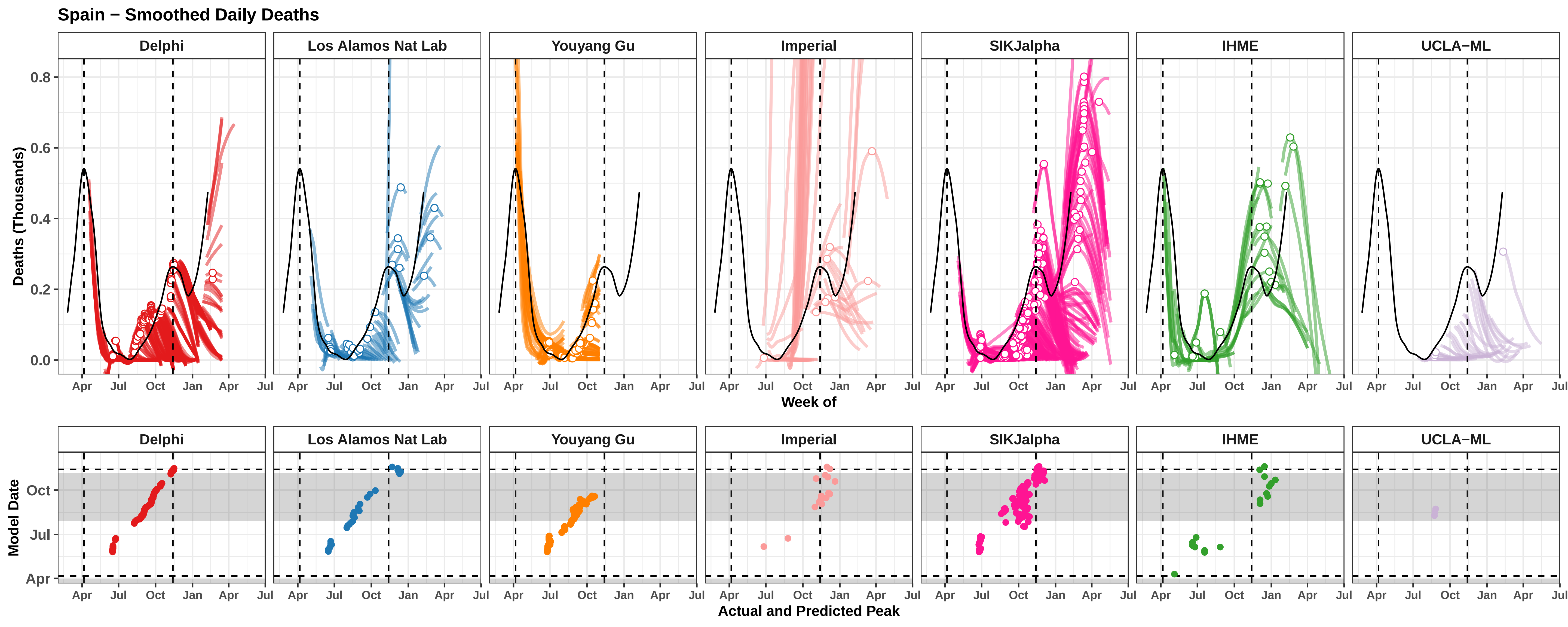
Spain − Smoothed Daily Deaths
Delphi
Los Alamos Nat Lab
Youyang Gu
Imperial
SIKJalpha
IHME
UCLA−ML
Deaths (Thousands)
0.0
0.2
0.4
0.6
0.8
Apr
Jul
Oct
Jan
Week of
Model Date
Actual and Predicted Peak

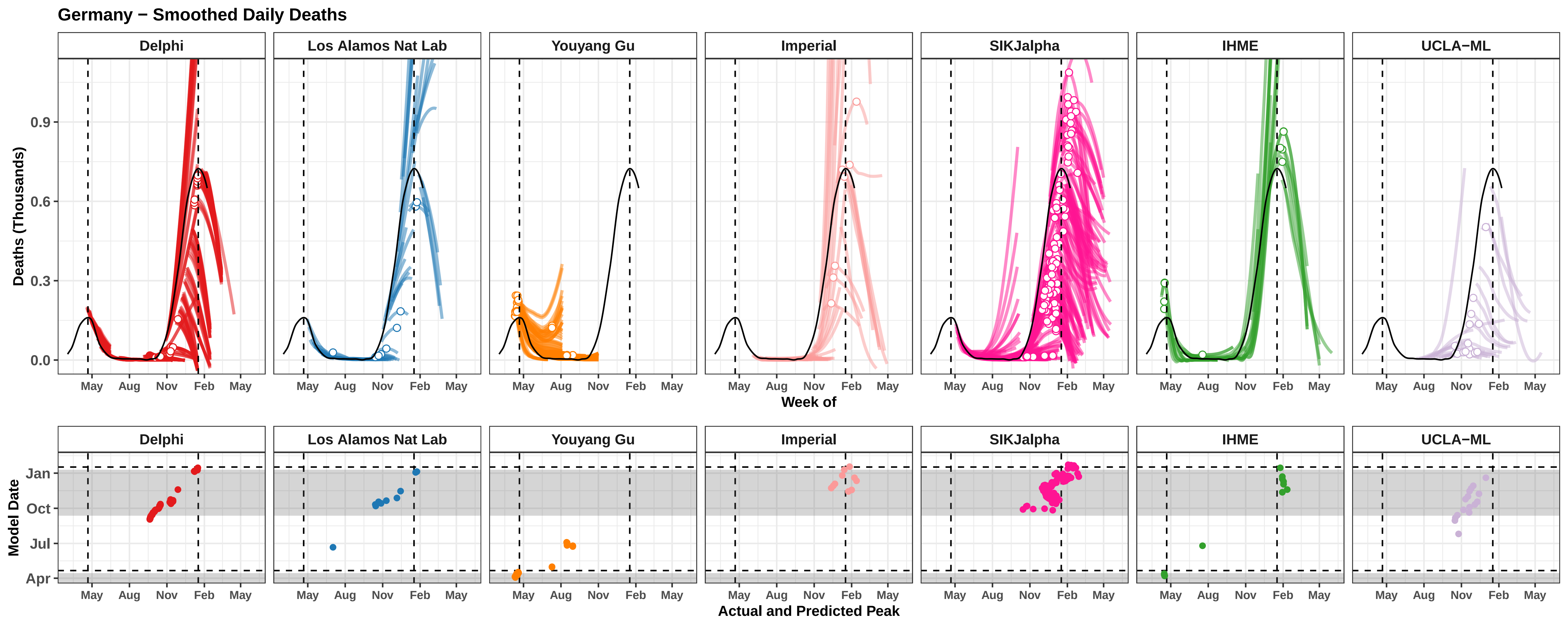

Germany − Smoothed Daily Deaths
Delphi
Los Alamos Nat Lab
Youyang Gu
Imperial
SIKJalpha
IHME
UCLA−ML
Deaths (Thousands)
0.0
0.3
0.6
0.9
May
Aug
Nov
Feb
May
Week of
Model Date
Apr
Jul
Oct
Jan
Actual and Predicted Peak

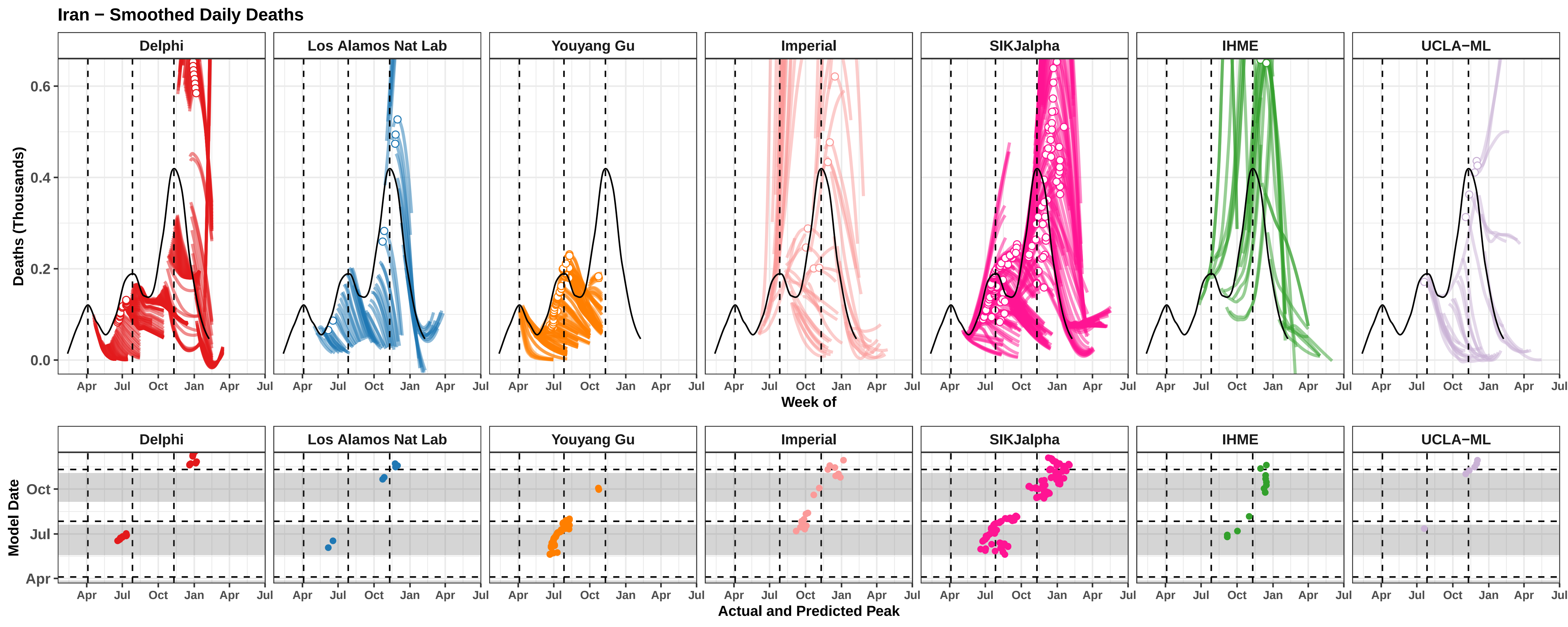

Iran − Smoothed Daily Deaths
Delphi
Los Alamos Nat Lab
Youyang Gu
Imperial
SIKJalpha
IHME
UCLA−ML
Deaths (Thousands)
0.0
0.2
0.4
0.6
Apr
Jul
Oct
Jan
Week of
Model Date
Actual and Predicted Peak

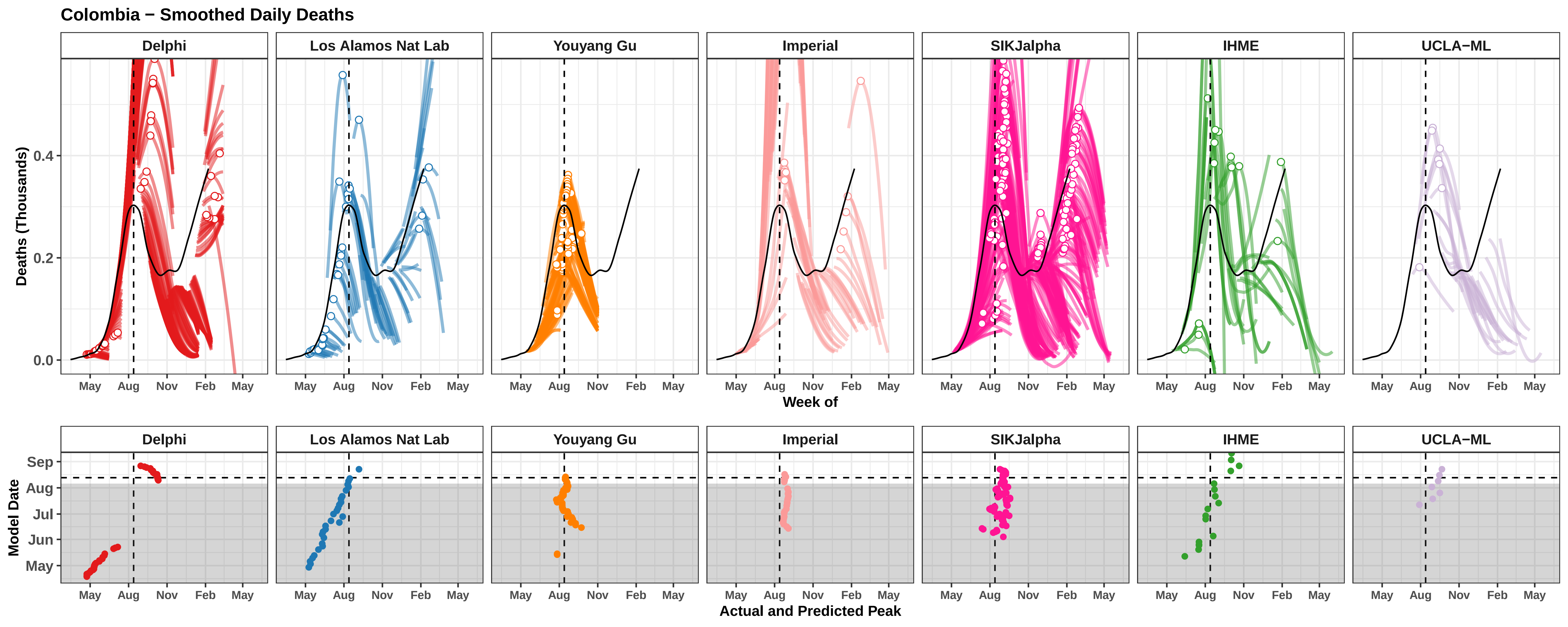

Colombia − Smoothed Daily Deaths
Delphi
Los Alamos Nat Lab
Youyang Gu
Imperial
SIKJalpha
IHME
UCLA−ML
Deaths (Thousands)
0.0
0.2
0.4
May Aug Nov Feb May
Week of
Model Date
May
Jun
Jul
Aug
Sep
Actual and Predicted Peak

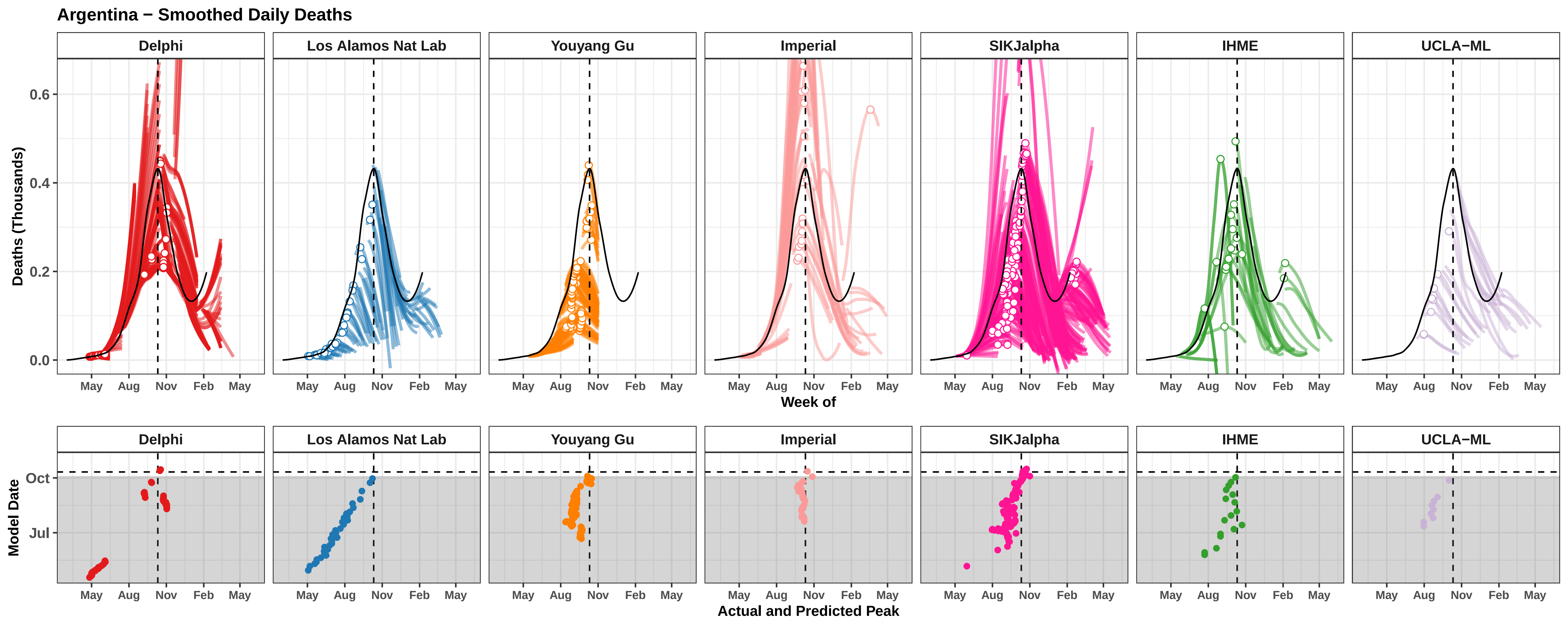

Argentina − Smoothed Daily Deaths
Delphi
Los Alamos Nat Lab
Youyang Gu
Imperial
SIKJalpha
IHME
UCLA−ML
Deaths (Thousands)
0.0
0.2
0.4
0.6
May
Aug
Nov
Feb
May
Week of
Model Date
Oct
Jul
Actual and Predicted Peak

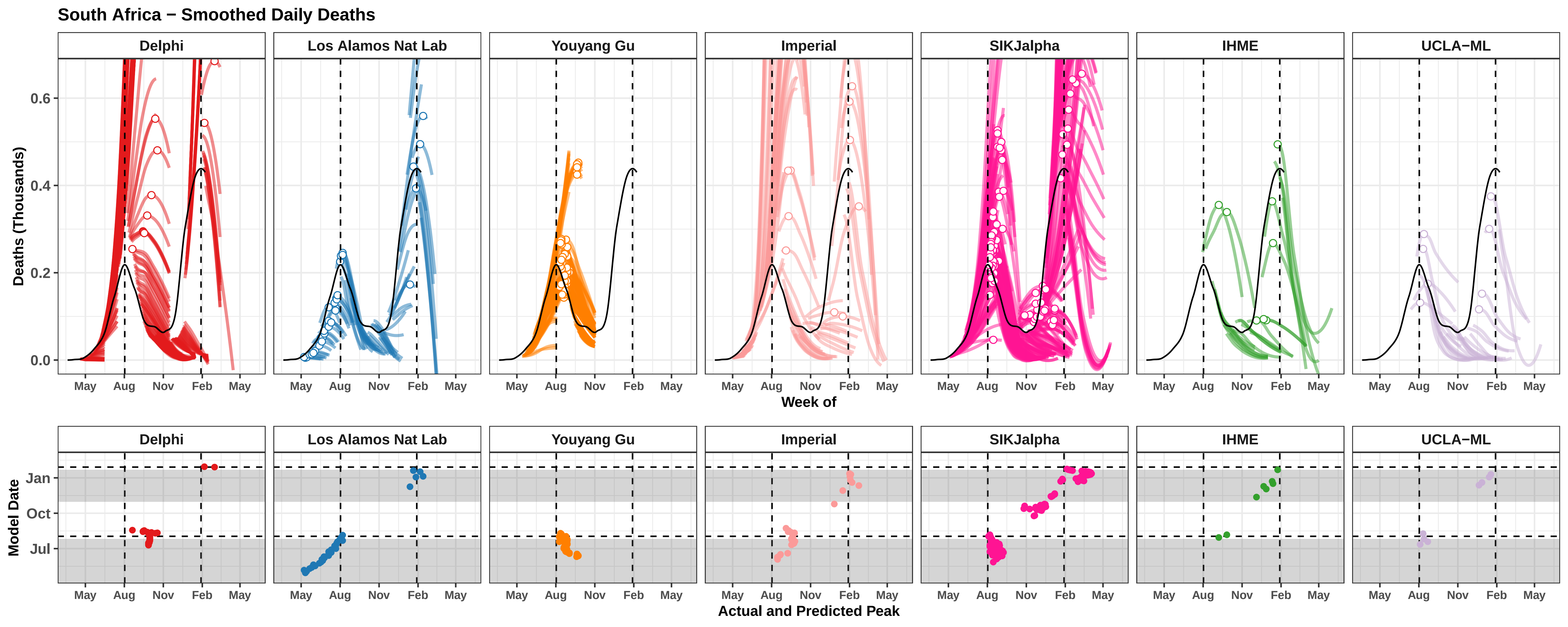
South Africa − Smoothed Daily Deaths
Delphi
Los Alamos Nat Lab
Youyang Gu
Imperial
SIKJalpha
IHME
UCLA−ML
Deaths (Thousands)
0.0
0.2
0.4
0.6
May
Aug
Nov
Feb
May
Week of
Model Date
Jul
Oct
Jan
Actual and Predicted Peak

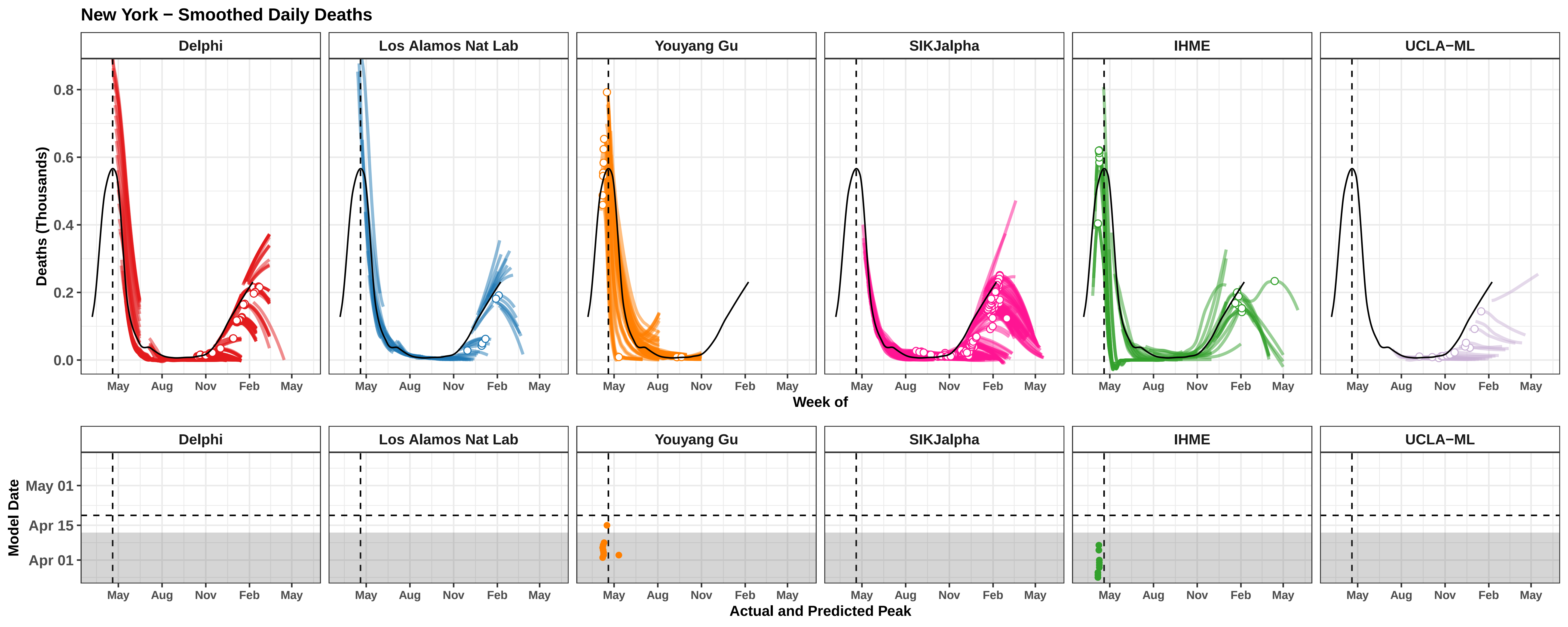
New York − Smoothed Daily Deaths
Delphi
Los Alamos Nat Lab
Youyang Gu
SIKJalpha
IHME
UCLA−ML
Deaths (Thousands)
0.0
0.2
0.4
0.6
0.8
May
Aug
Nov
Feb
May
Week of
Model Date
May 01
Apr 15
Apr 01
Actual and Predicted Peak

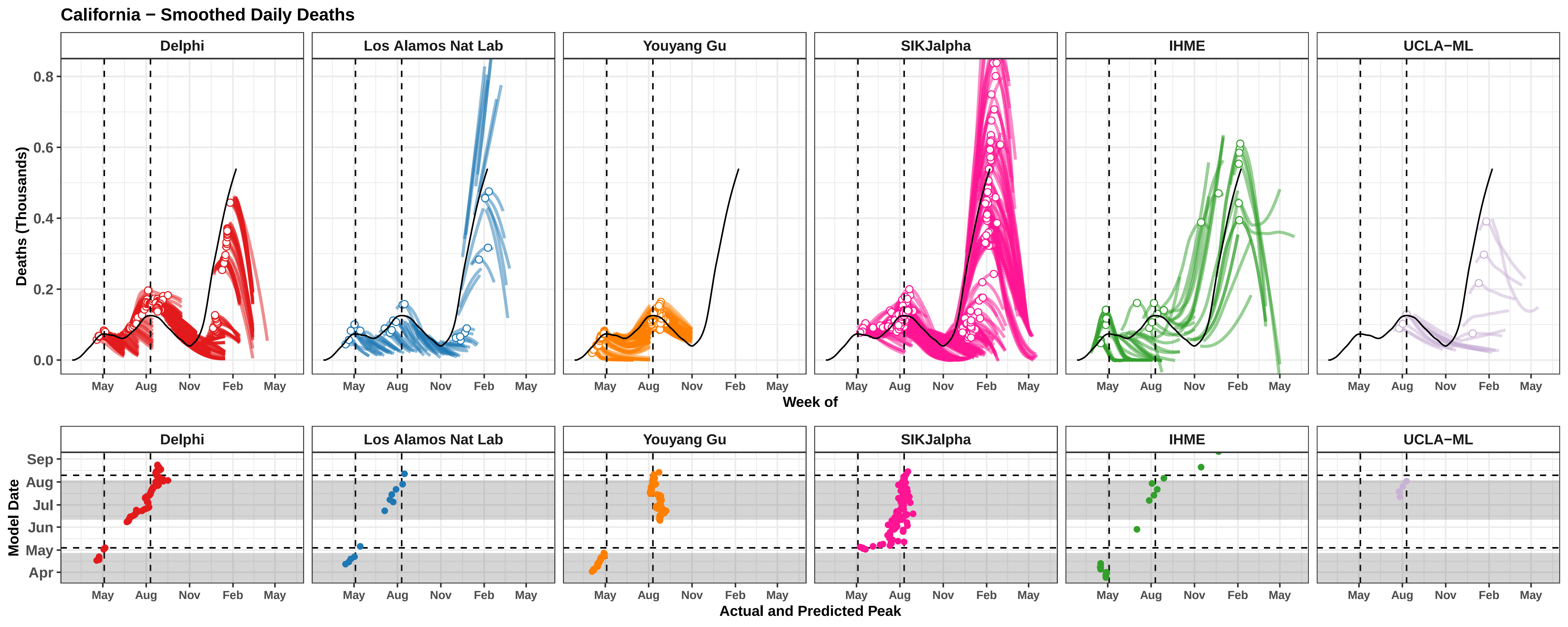

California − Smoothed Daily Deaths
Delphi
Los Alamos Nat Lab
Youyang Gu
SIKJalpha
IHME
UCLA−ML
Deaths (Thousands)
0.0
0.2
0.4
0.6
0.8
May
Aug
Nov
Feb
May
Week of
Model Date
Apr
May
Jun
Jul
Aug
Sep
Actual and Predicted Peak

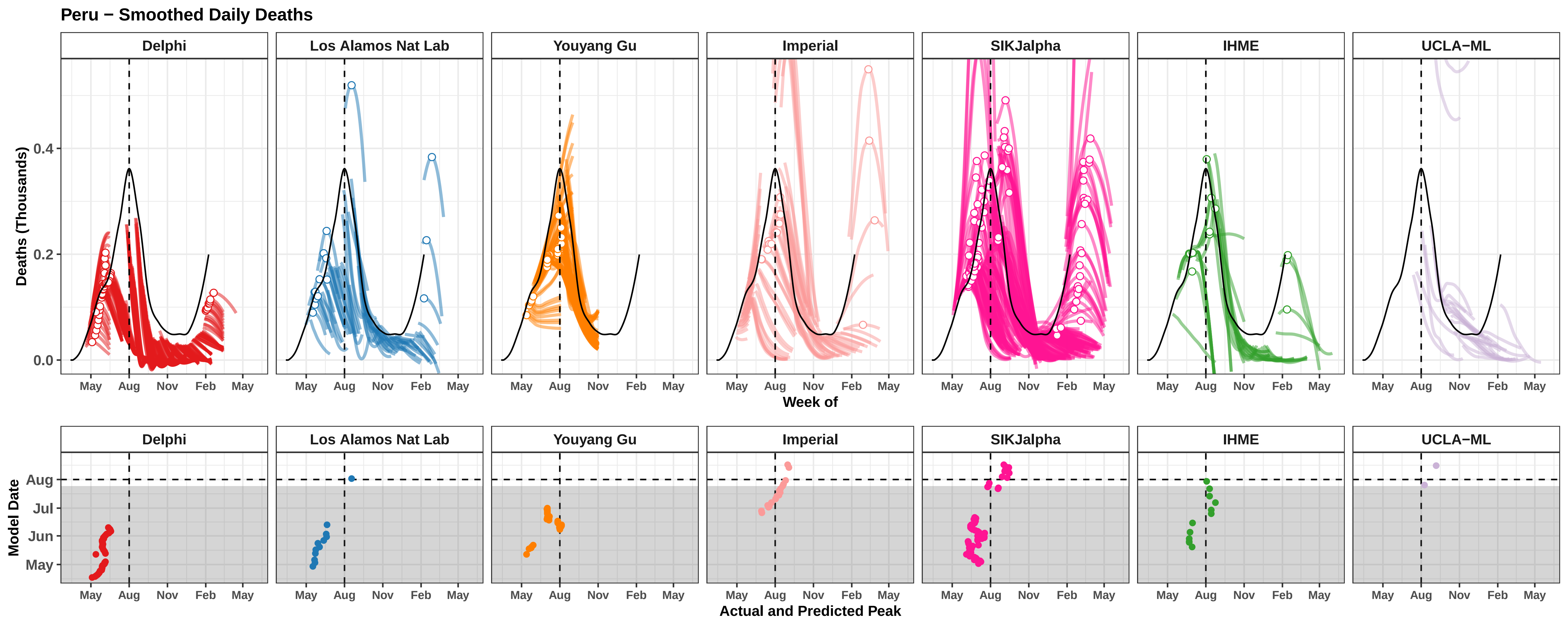

Peru − Smoothed Daily Deaths
Delphi
Los Alamos Nat Lab
Youyang Gu
Imperial
SIKJalpha
IHME
UCLA−ML
Deaths (Thousands)
0.0
0.2
0.4
May
Aug
Nov
Feb
May
Week of
Model Date
May
Jun
Jul
Aug
Actual and Predicted Peak

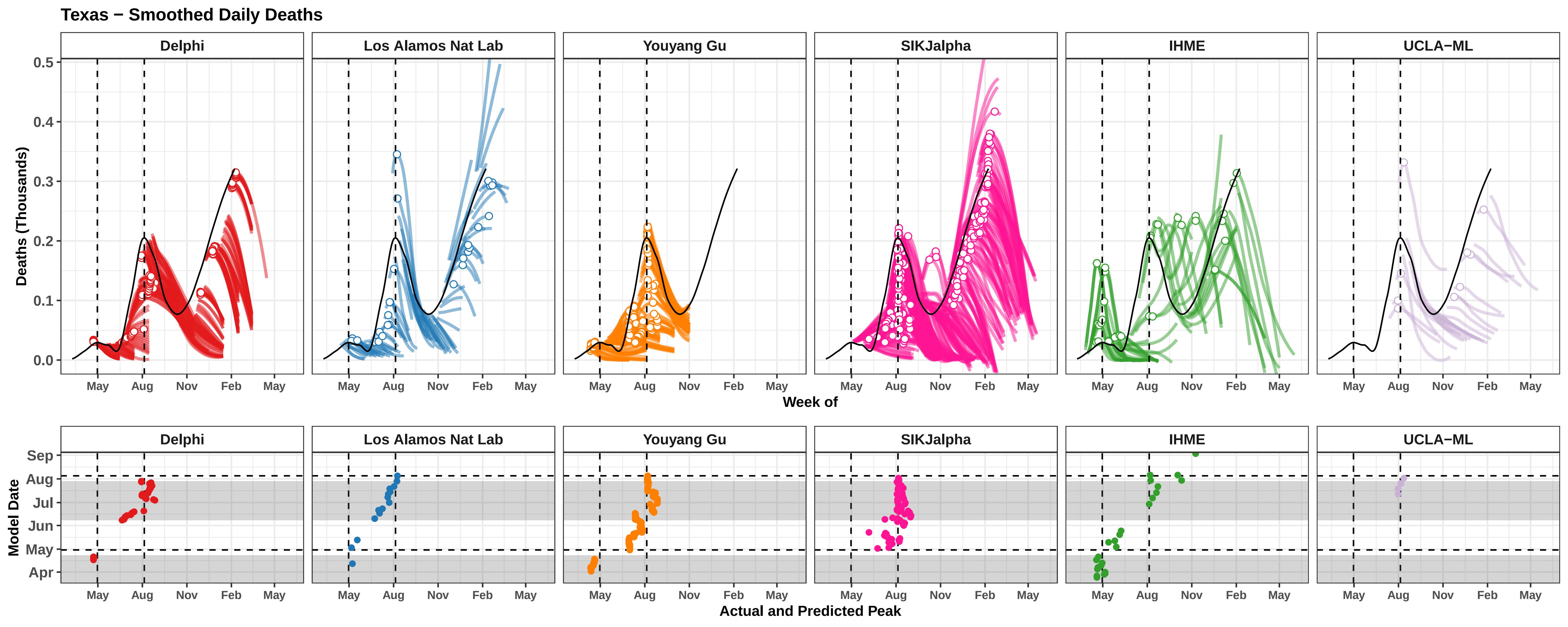

Texas − Smoothed Daily Deaths
Delphi
Los Alamos Nat Lab
Youyang Gu
SIKJalpha
IHME
UCLA−ML
Deaths (Thousands)
0.0
0.1
0.2
0.3
0.4
0.5
May
Aug
Nov
Feb
May
Week of
Model Date
Apr
May
Jun
Jul
Aug
Sep
Actual and Predicted Peak

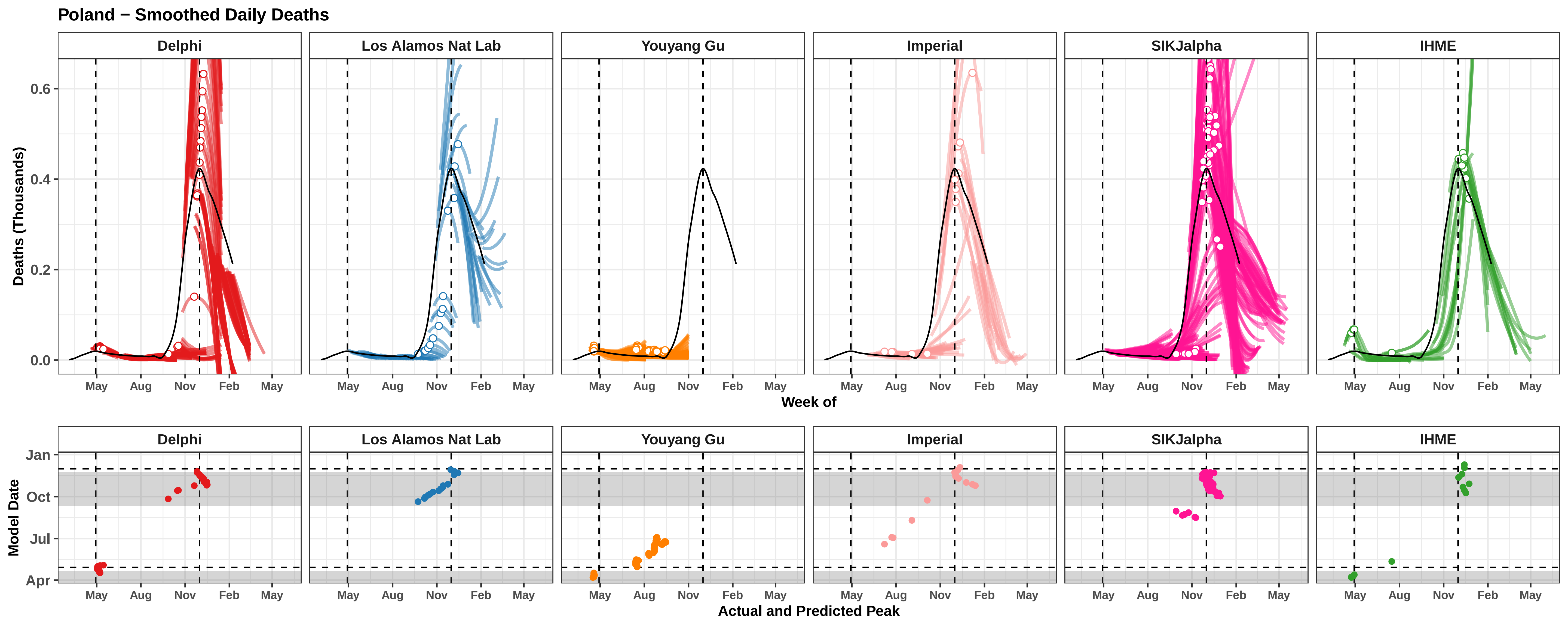

Poland − Smoothed Daily Deaths
Delphi
Los Alamos Nat Lab
Youyang Gu
Imperial
SIKJalpha
IHME
Deaths (Thousands)
0.0
0.2
0.4
0.6
May
Aug
Nov
Feb
May
Week of
Model Date
Jan
Oct
Jul
Apr
Actual and Predicted Peak

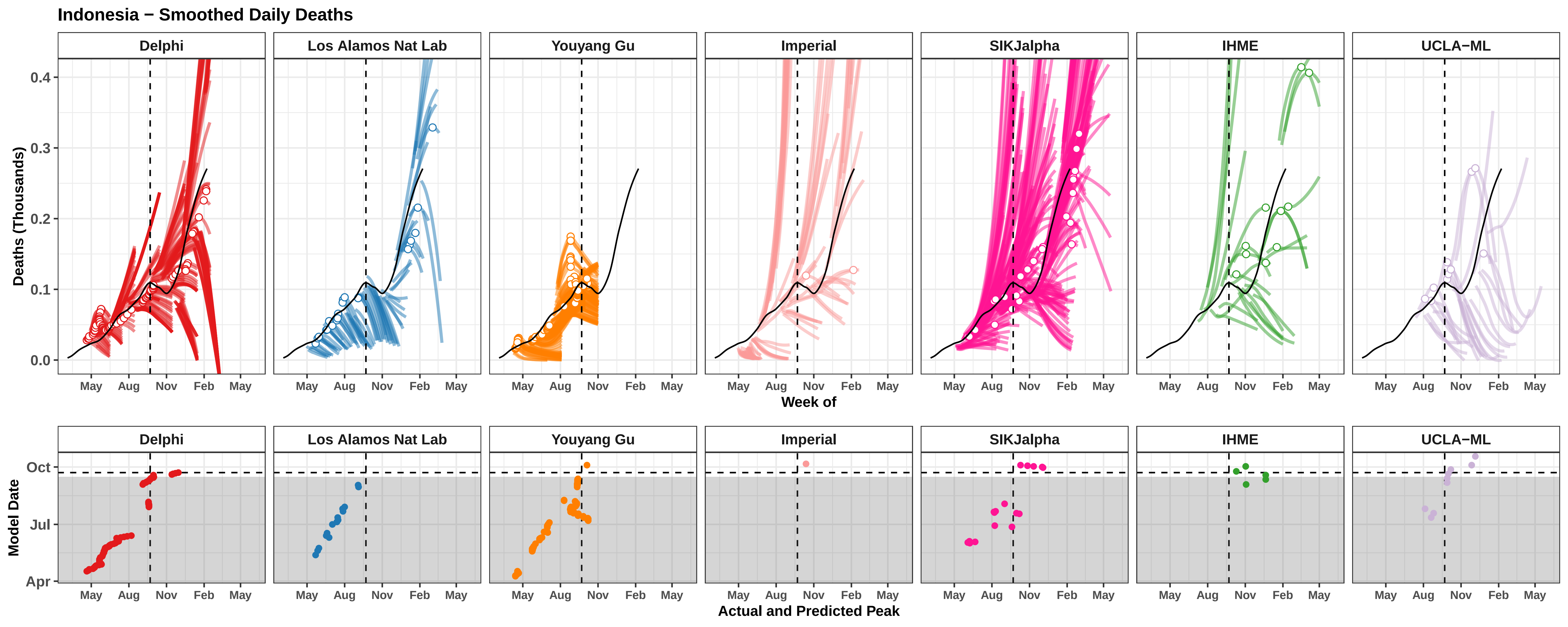

Indonesia − Smoothed Daily Deaths
Delphi
Los Alamos Nat Lab
Youyang Gu
Imperial
SIKJalpha
IHME
UCLA−ML
Deaths (Thousands)
0.0
0.1
0.2
0.3
0.4
May
Aug
Nov
Feb
May
Week of
Model Date
Apr
Jul
Oct
Actual and Predicted Peak

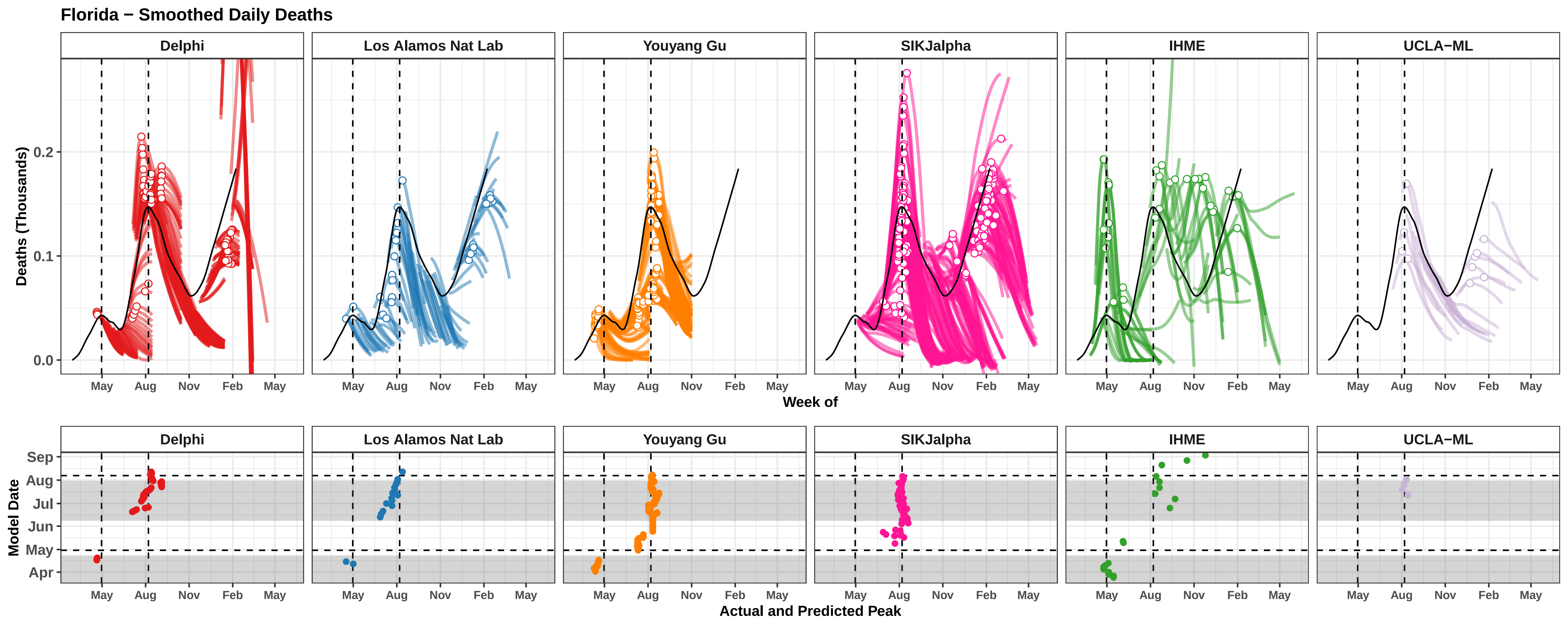

Florida − Smoothed Daily Deaths
Delphi
Los Alamos Nat Lab
Youyang Gu
SIKJalpha
IHME
UCLA−ML
Deaths (Thousands)
0.0
0.1
0.2
May
Aug
Nov
Feb
May
Week of
Model Date
Sep
Aug
Jul
Jun
May
Apr
Actual and Predicted Peak

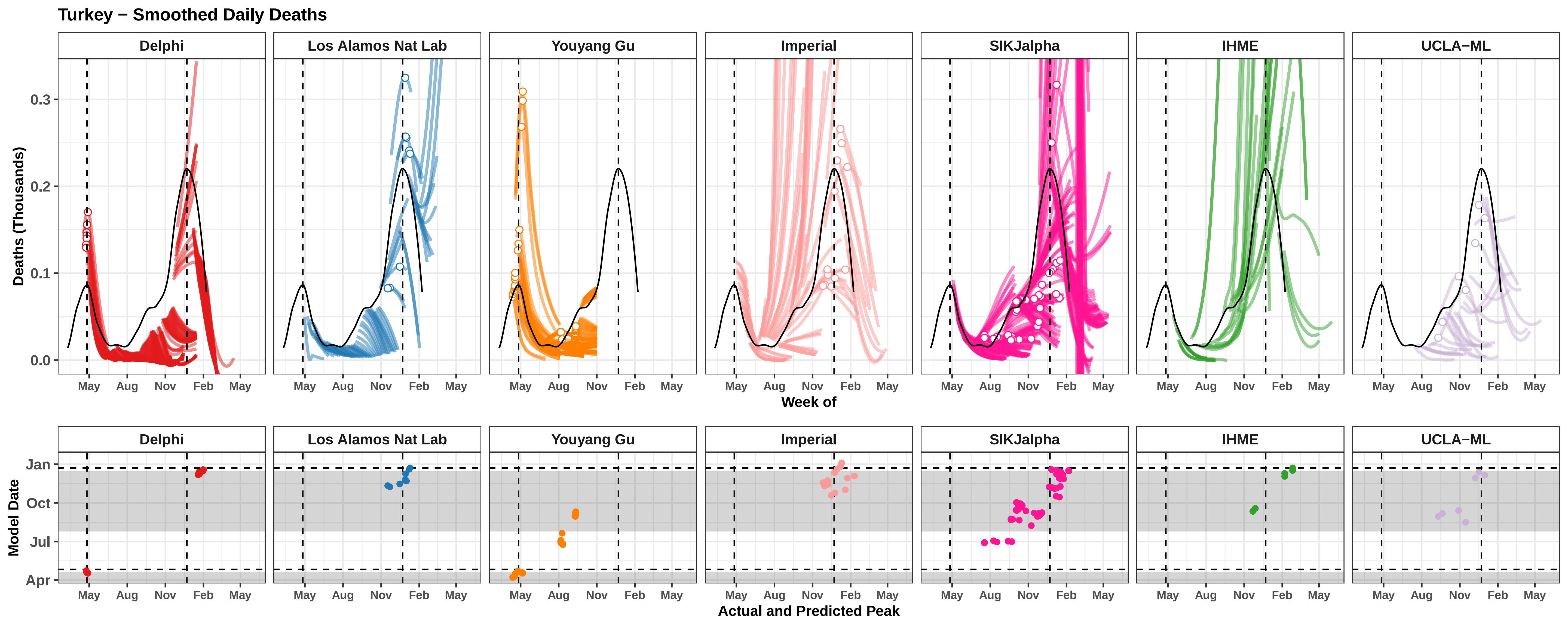

Turkey − Smoothed Daily Deaths
Delphi
Los Alamos Nat Lab
Youyang Gu
Imperial
SIKJalpha
IHME
UCLA−ML
Deaths (Thousands)
0.0
0.1
0.2
0.3
May
Aug
Nov
Feb
May
Week of
Model Date
Jan
Oct
Jul
Apr
Actual and Predicted Peak

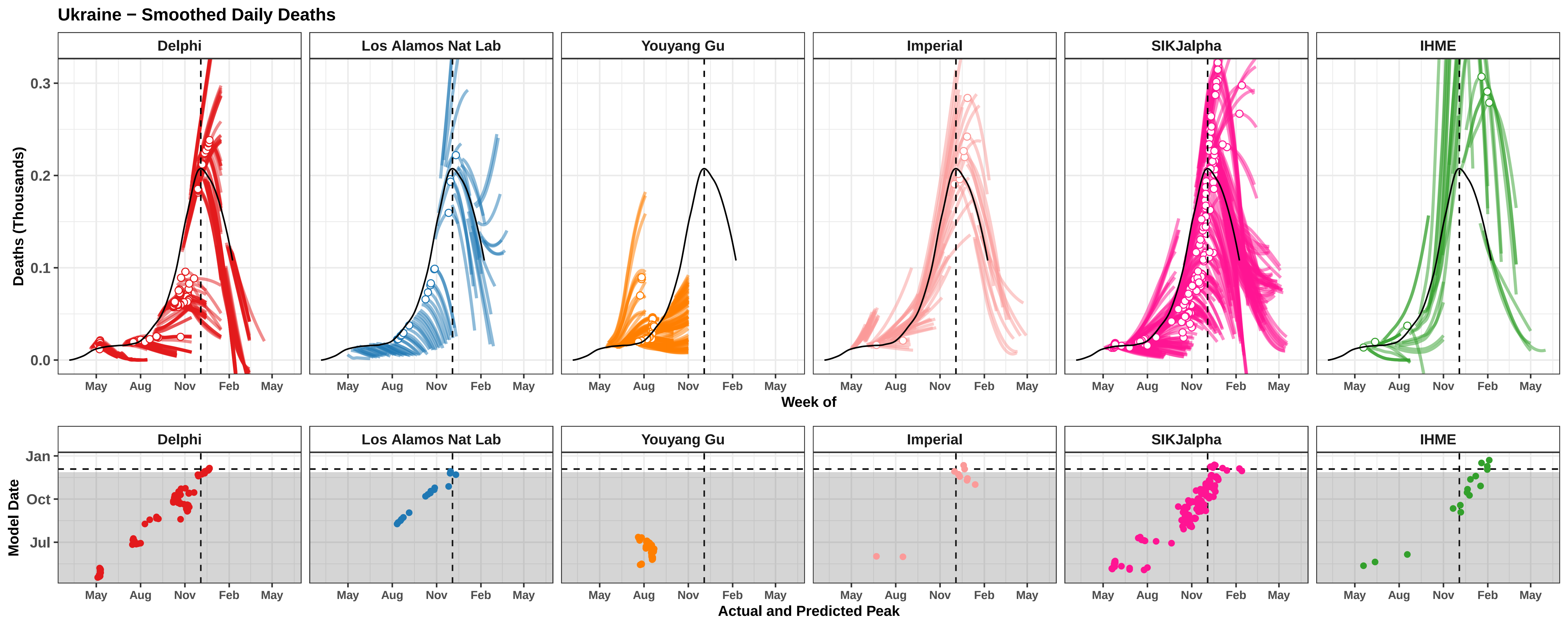
Ukraine − Smoothed Daily Deaths
Delphi
Los Alamos Nat Lab
Youyang Gu
Imperial
SIKJalpha
IHME
Deaths (Thousands)
0.0
0.1
0.2
0.3
May
Aug
Nov
Feb
May
Week of
Model Date
Jan
Oct
Jul
Actual and Predicted Peak

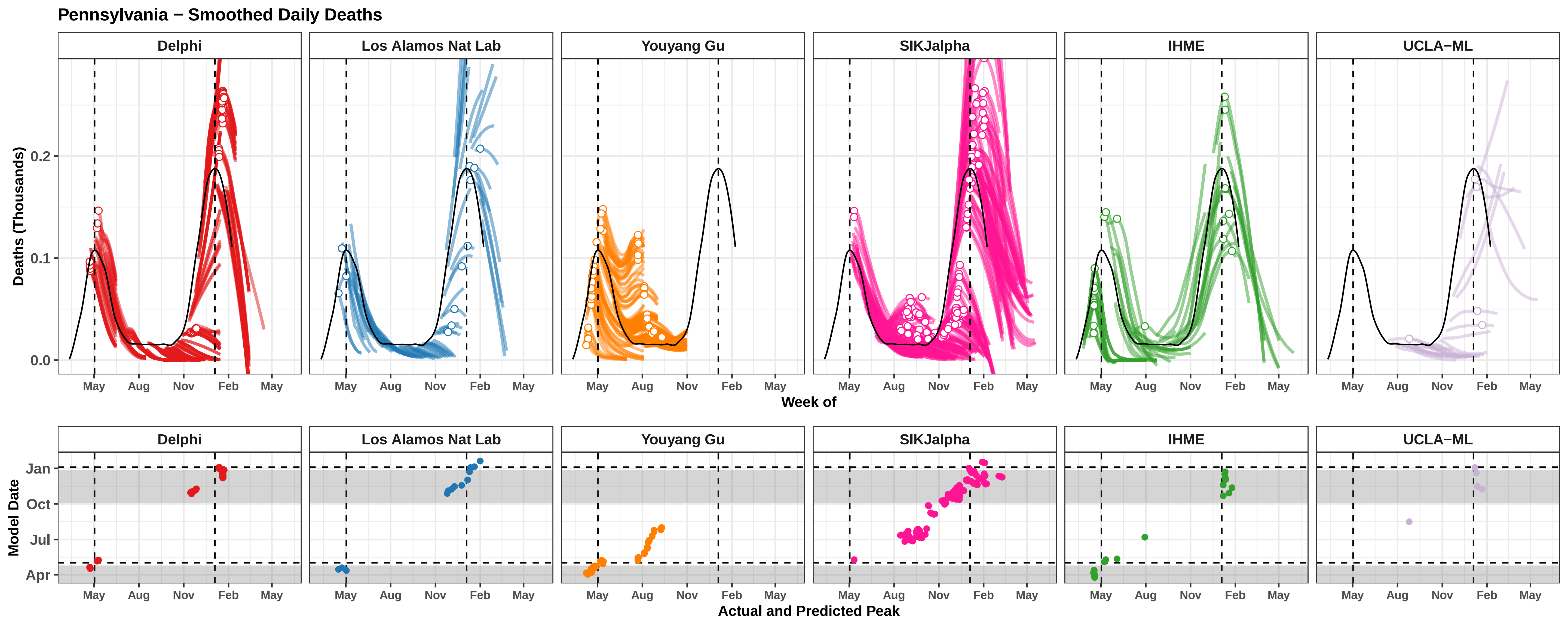

Pennsylvania − Smoothed Daily Deaths
Delphi
Los Alamos Nat Lab
Youyang Gu
SIKJalpha
IHME
UCLA−ML
Deaths (Thousands)
0.0
0.1
0.2
May
Aug
Nov
Feb
Week of
Model Date
Apr
Jul
Oct
Jan
Actual and Predicted Peak

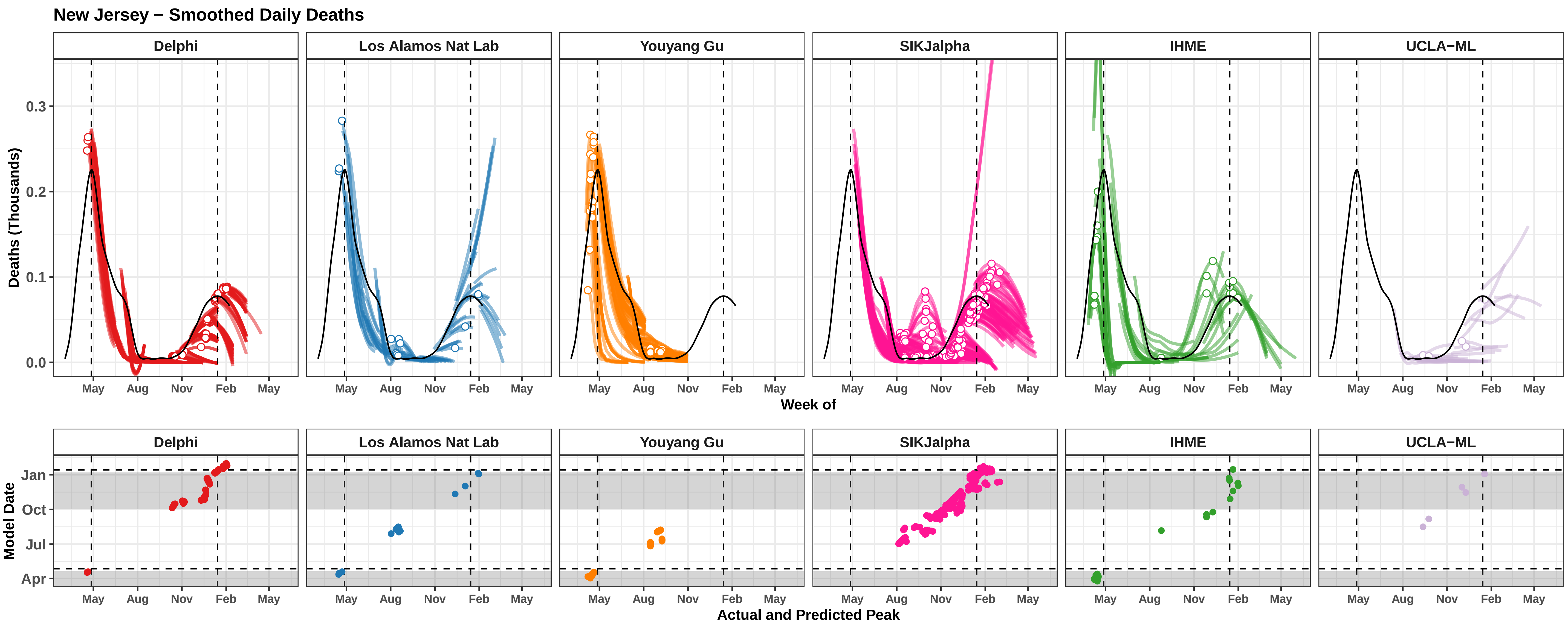
New Jersey − Smoothed Daily Deaths
Delphi
Los Alamos Nat Lab
Youyang Gu
SIKJalpha
IHME
UCLA−ML
Deaths (Thousands)
0.0
0.1
0.2
0.3
May
Aug
Nov
Feb
May
Week of
Model Date
Apr
Jul
Oct
Jan
Actual and Predicted Peak

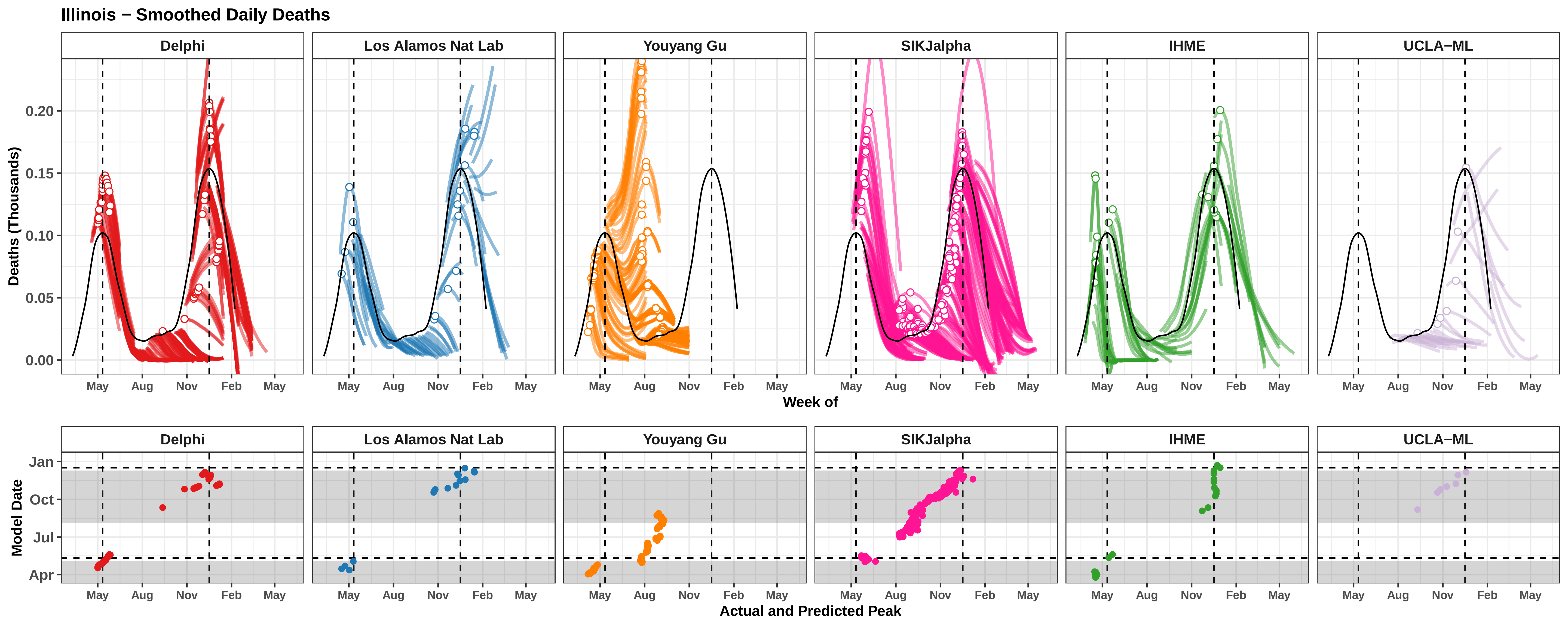

Illinois − Smoothed Daily Deaths
Delphi
Los Alamos Nat Lab
Youyang Gu
SIKJalpha
IHME
UCLA−ML
Deaths (Thousands)
0.00
0.05
0.10
0.15
0.20
May
Aug
Nov
Feb
May
Week of
Model Date
Jan
Oct
Jul
Apr
Actual and Predicted Peak

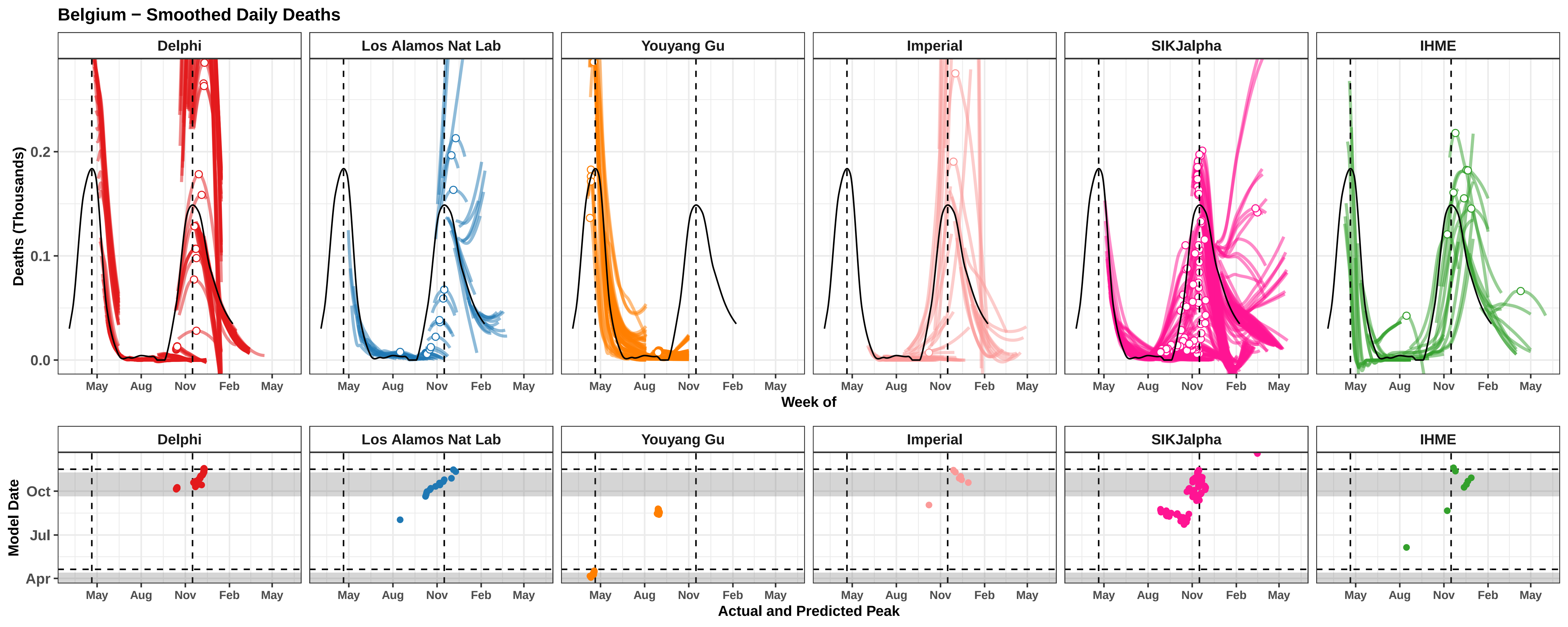
Belgium − Smoothed Daily Deaths
Delphi
Los Alamos Nat Lab
Youyang Gu
Imperial
SIKJalpha
IHME
Deaths (Thousands)
0.0
0.1
0.2
May
Aug
Nov
Feb
May
Week of
Model Date
Apr
Jul
Oct
Actual and Predicted Peak

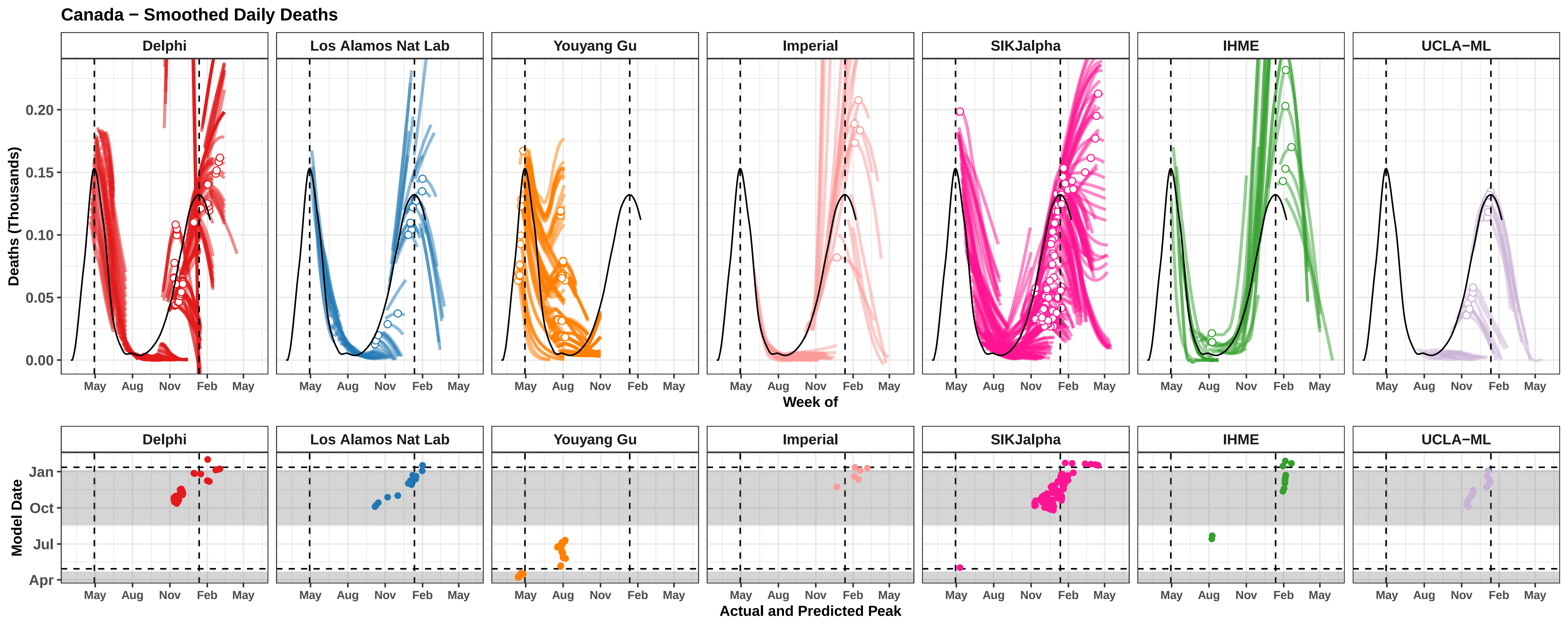

Canada − Smoothed Daily Deaths
Delphi
Los Alamos Nat Lab
Youyang Gu
Imperial
SIKJalpha
IHME
UCLA−ML
Deaths (Thousands)
0.00
0.05
0.10
0.15
0.20
May
Aug
Nov
Feb
May
Week of
Model Date
Apr
Jul
Oct
Jan
Actual and Predicted Peak

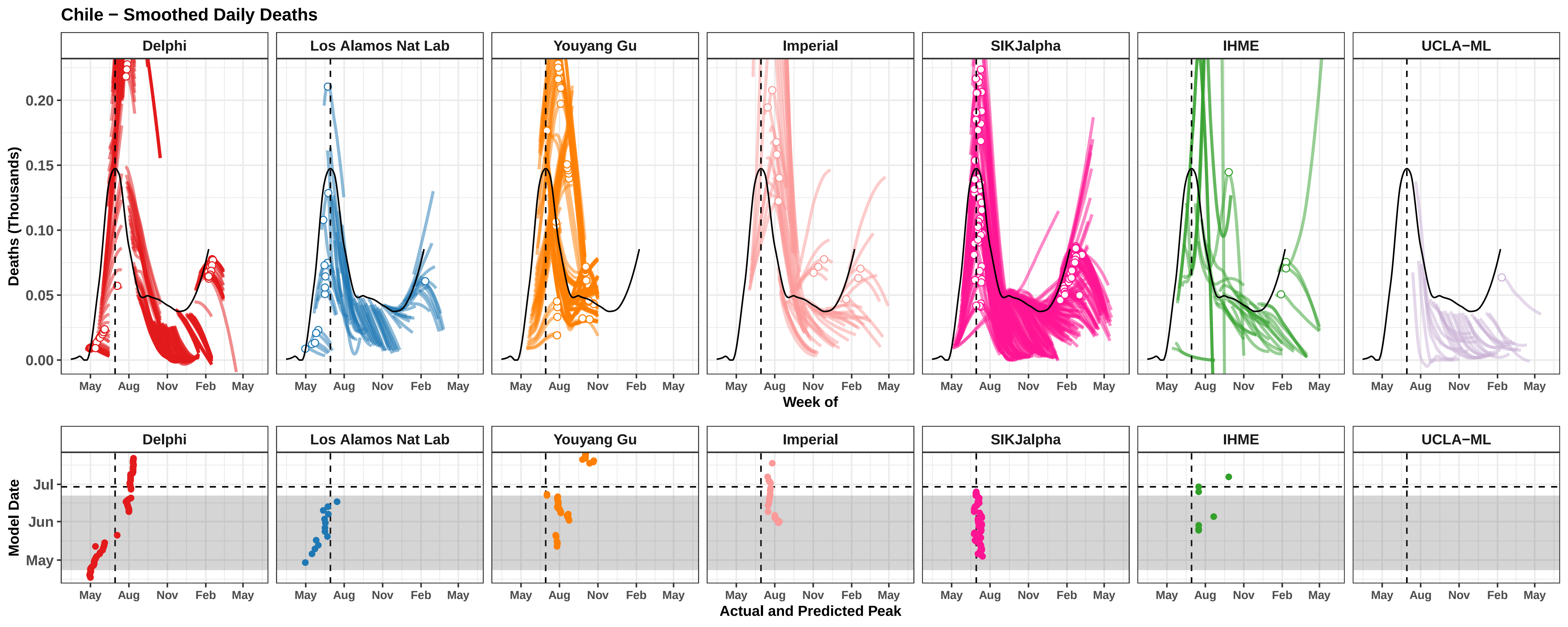
Chile − Smoothed Daily Deaths
Delphi
Los Alamos Nat Lab
Youyang Gu
Imperial
SIKJalpha
IHME
UCLA−ML
Deaths (Thousands)
0.00
0.05
0.10
0.15
0.20
May
Aug
Nov
Feb
May
Week of
Model Date
May
Jun
Jul
Actual and Predicted Peak

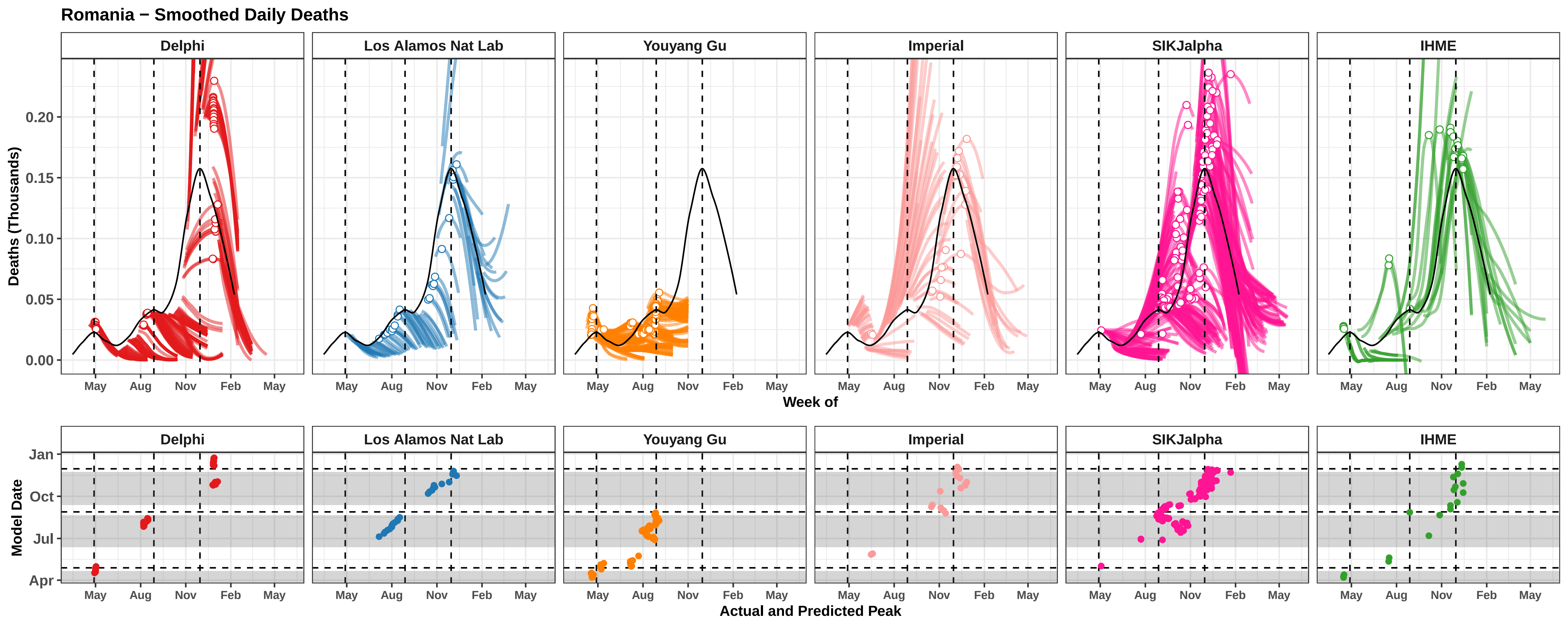

Romania − Smoothed Daily Deaths
Delphi
Los Alamos Nat Lab
Youyang Gu
Imperial
SIKJalpha
IHME
Deaths (Thousands)
0.00
0.05
0.10
0.15
0.20
May
Aug
Nov
Feb
May
Week of
Model Date
Jan
Oct
Jul
Apr
Actual and Predicted Peak

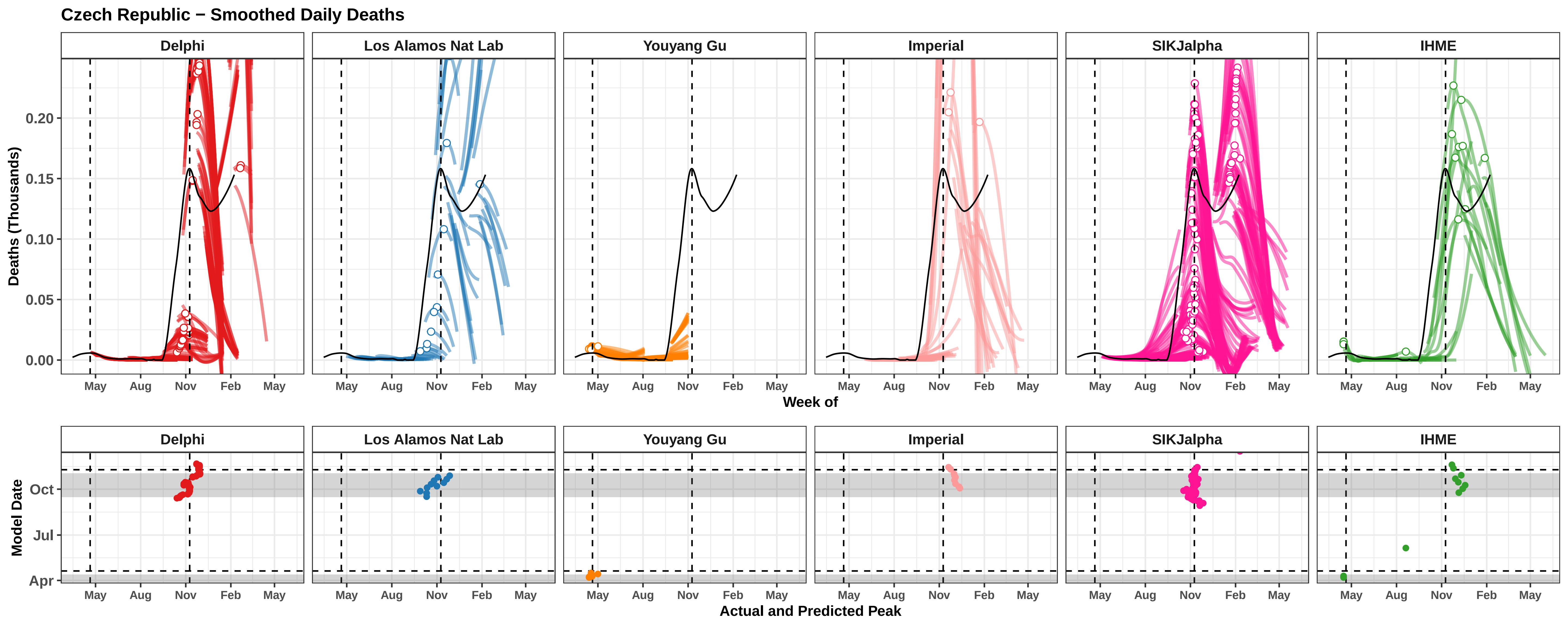

Czech Republic − Smoothed Daily Deaths
Delphi
Los Alamos Nat Lab
Youyang Gu
Imperial
SIKJalpha
IHME
Deaths (Thousands)
0.00
0.05
0.10
0.15
0.20
May
Aug
Nov
Feb
May
Week of
Model Date
Apr
Jul
Oct
Actual and Predicted Peak

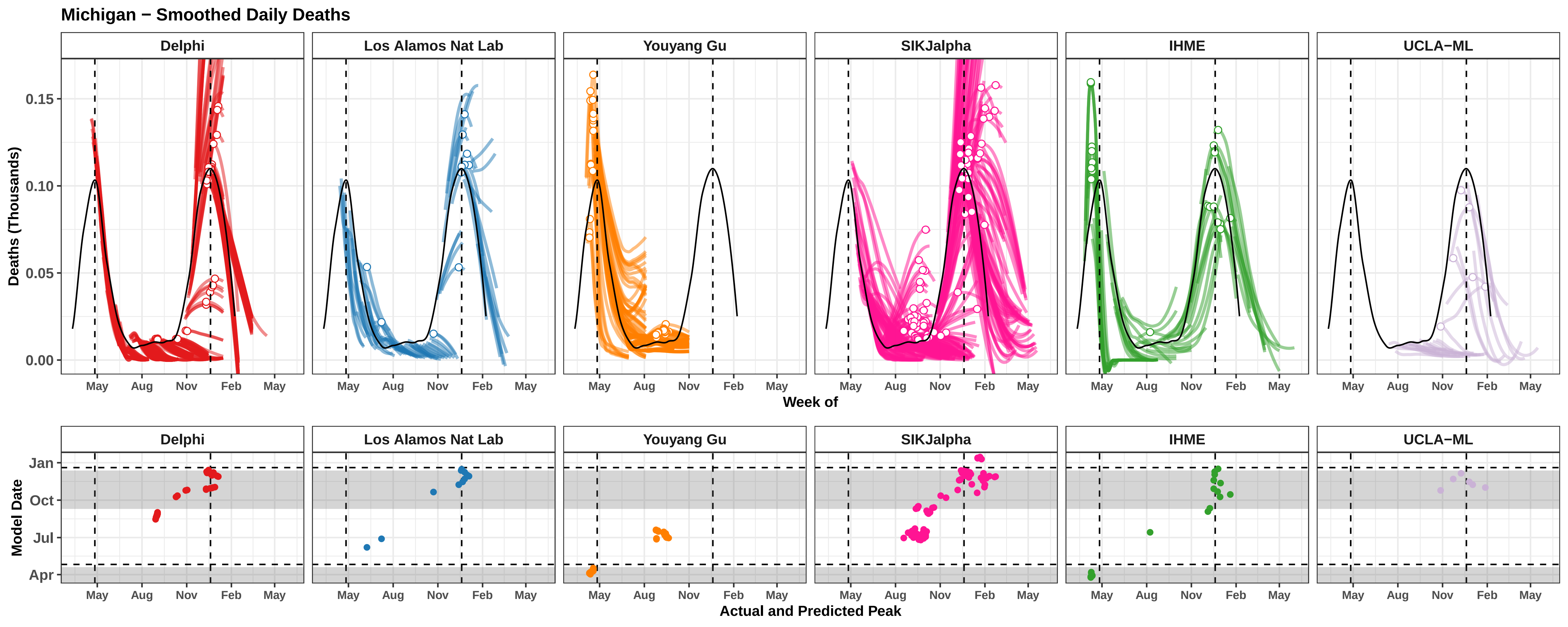

Michigan − Smoothed Daily Deaths
Delphi
Los Alamos Nat Lab
Youyang Gu
SIKJalpha
IHME
UCLA−ML
Deaths (Thousands)
0.15
0.10
0.05
0.00
May
Aug
Nov
Feb
May
Week of
Model Date
Jan
Oct
Jul
Apr
Actual and Predicted Peak

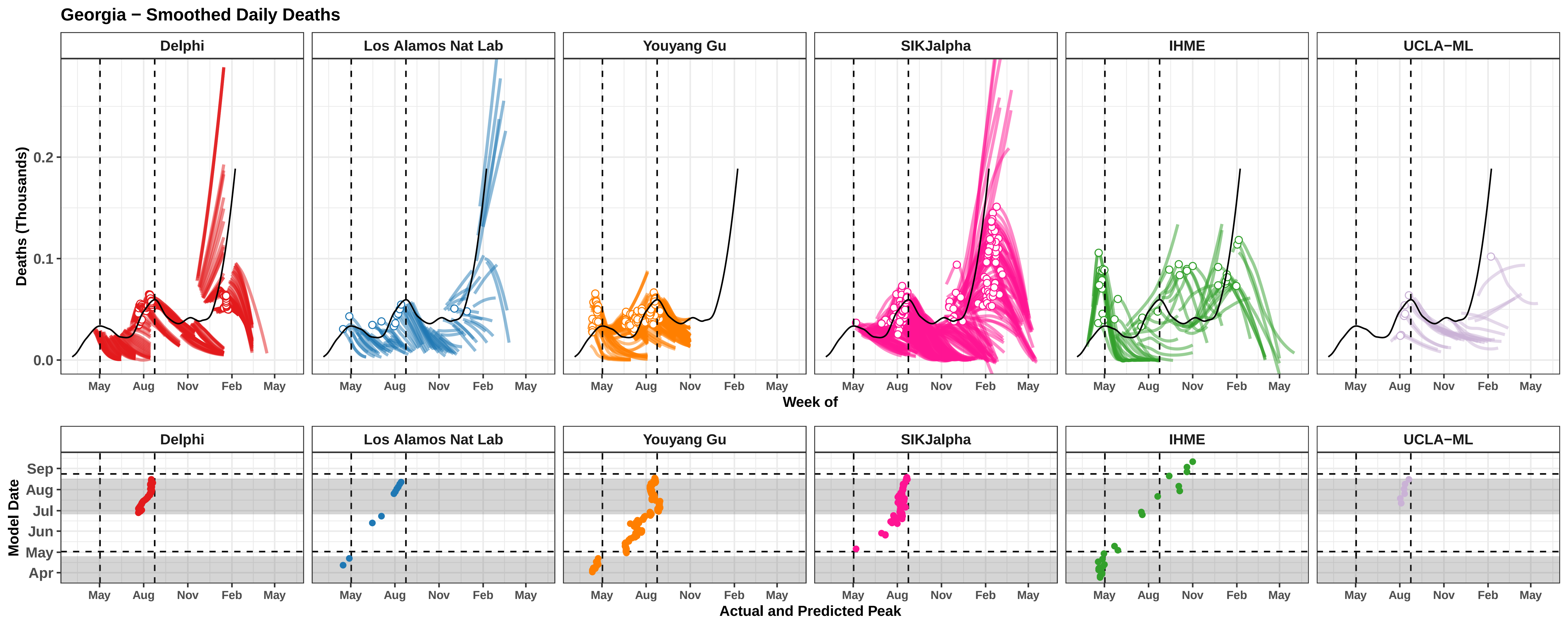

Georgia − Smoothed Daily Deaths
Delphi
Los Alamos Nat Lab
Youyang Gu
SIKJalpha
IHME
UCLA−ML
Deaths (Thousands)
0.0
0.1
0.2
May
Aug
Nov
Feb
May
Week of
Model Date
Apr
May
Jun
Jul
Aug
Sep
Actual and Predicted Peak

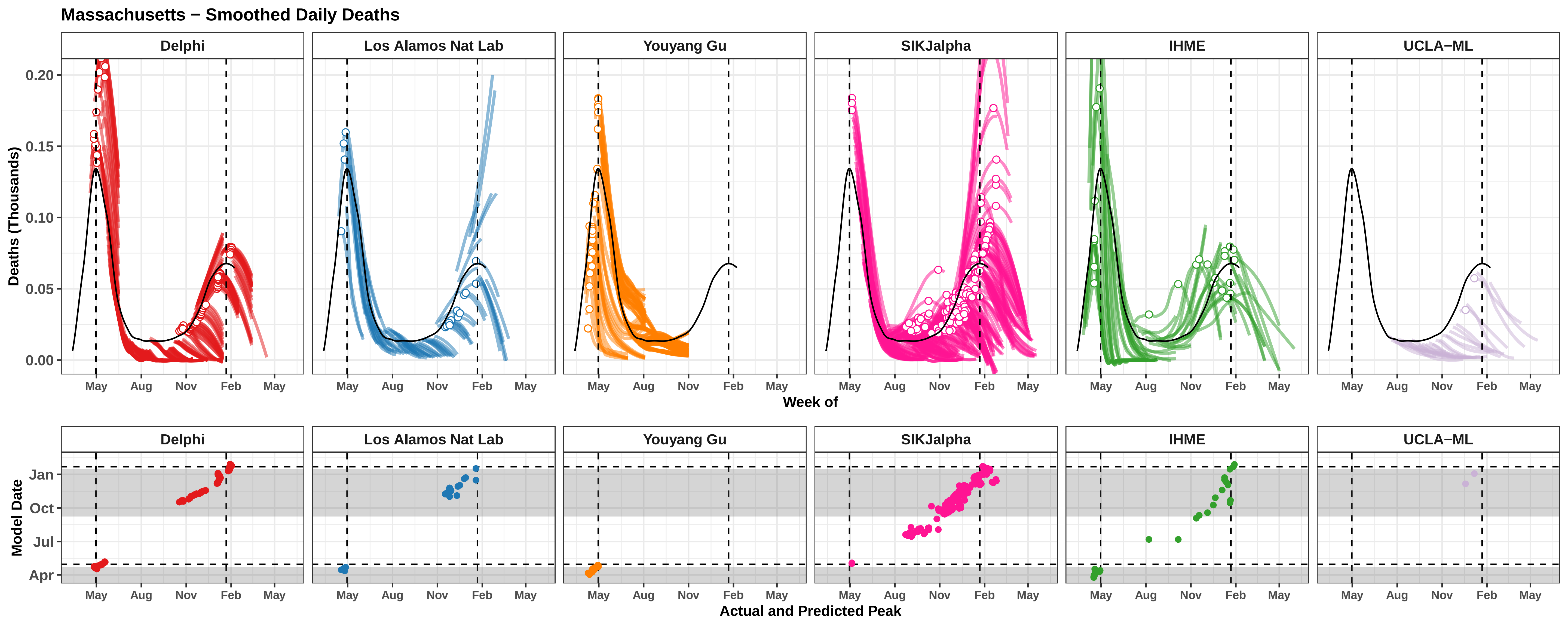

Massachusetts − Smoothed Daily Deaths
Delphi
Los Alamos Nat Lab
Youyang Gu
SIKJalpha
IHME
UCLA−ML
Deaths (Thousands)
0.00
0.05
0.10
0.15
0.20
May
Aug
Nov
Feb
May
Week of
Model Date
Apr
Jul
Oct
Jan
Actual and Predicted Peak

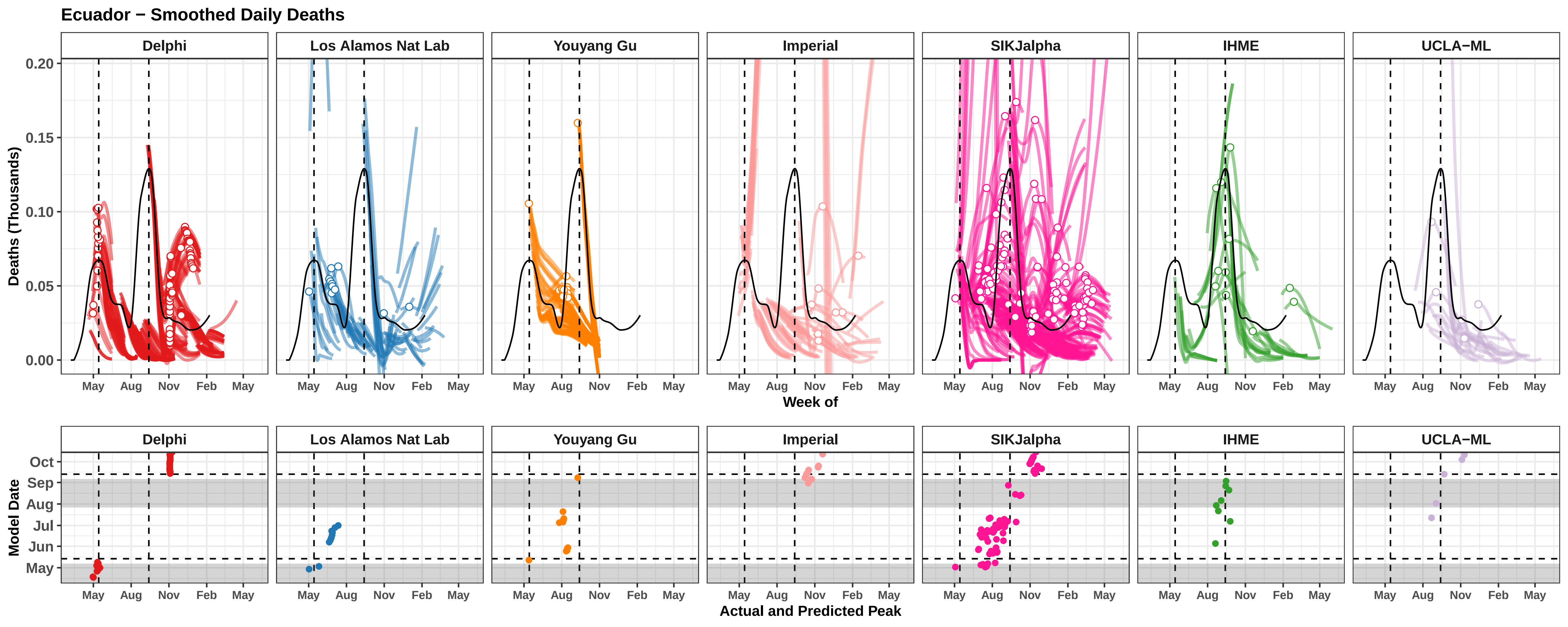

Ecuador − Smoothed Daily Deaths
Delphi
Los Alamos Nat Lab
Youyang Gu
Imperial
SIKJalpha
IHME
UCLA−ML
Deaths (Thousands)
0.00
0.05
0.10
0.15
0.20
May
Aug
Nov
Feb
May
Week of
Model Date
Oct
Sep
Aug
Jul
Jun
May
Actual and Predicted Peak

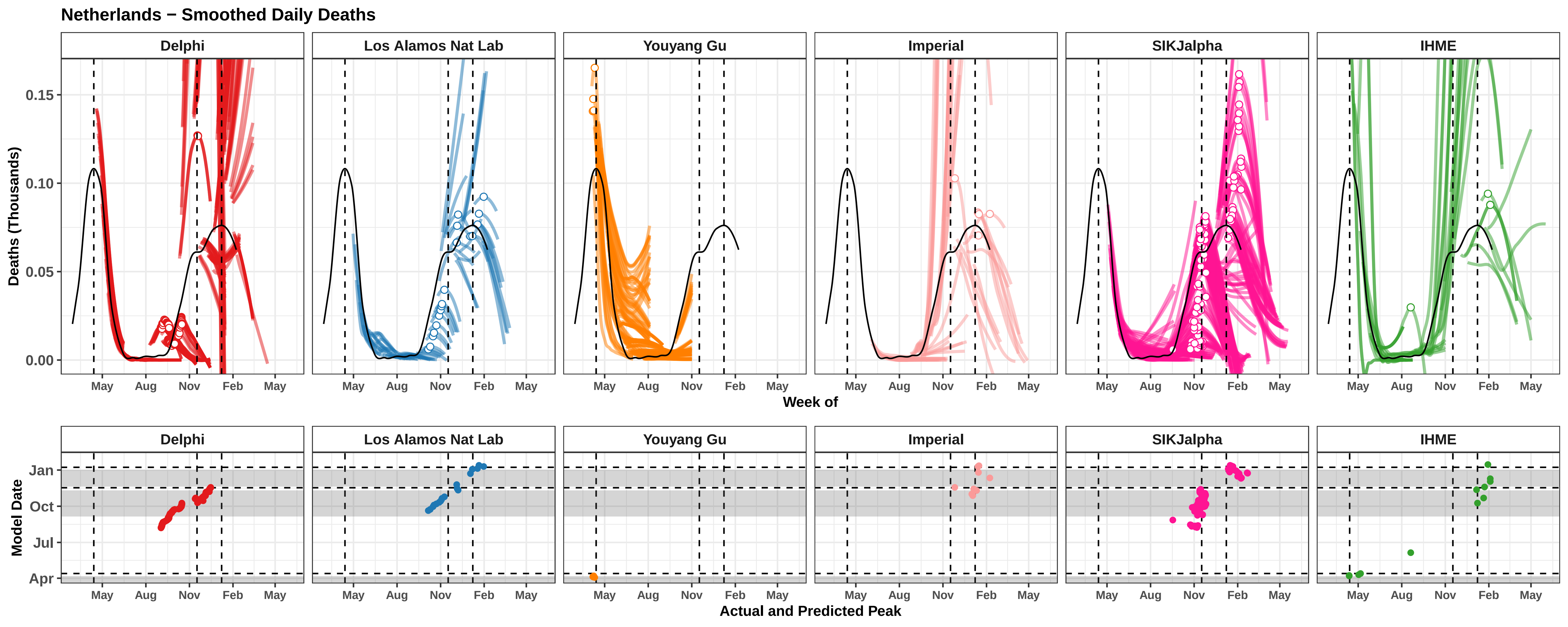

Netherlands − Smoothed Daily Deaths
Delphi
Los Alamos Nat Lab
Youyang Gu
Imperial
SIKJalpha
IHME
Deaths (Thousands)
0.15
0.10
0.05
0.00
May
Aug
Nov
Feb
May
Week of
Model Date
Jan
Oct
Jul
Apr
Actual and Predicted Peak

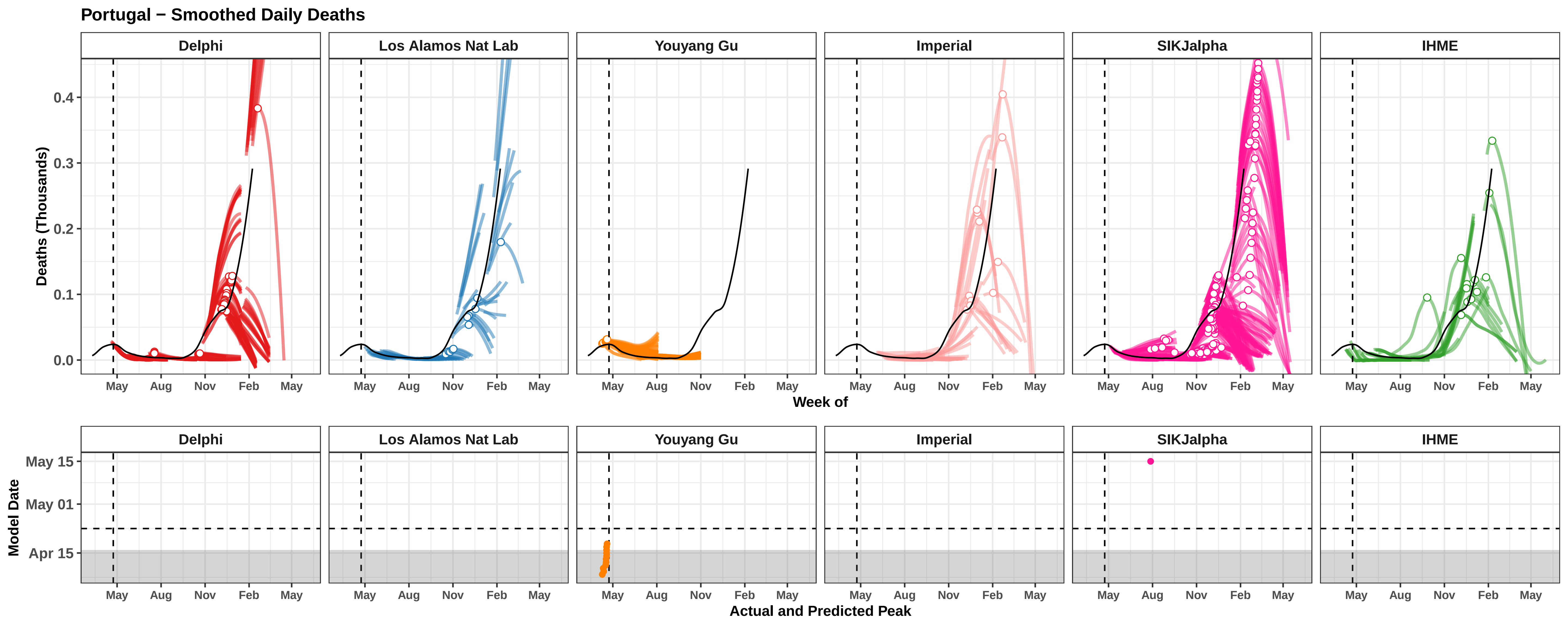

Portugal − Smoothed Daily Deaths
Delphi
Los Alamos Nat Lab
Youyang Gu
Imperial
SIKJalpha
IHME
Deaths (Thousands)
0.0
0.1
0.2
0.3
0.4
May
Aug
Nov
Feb
May
Week of
Model Date
May 15
May 01
Apr 15
Actual and Predicted Peak

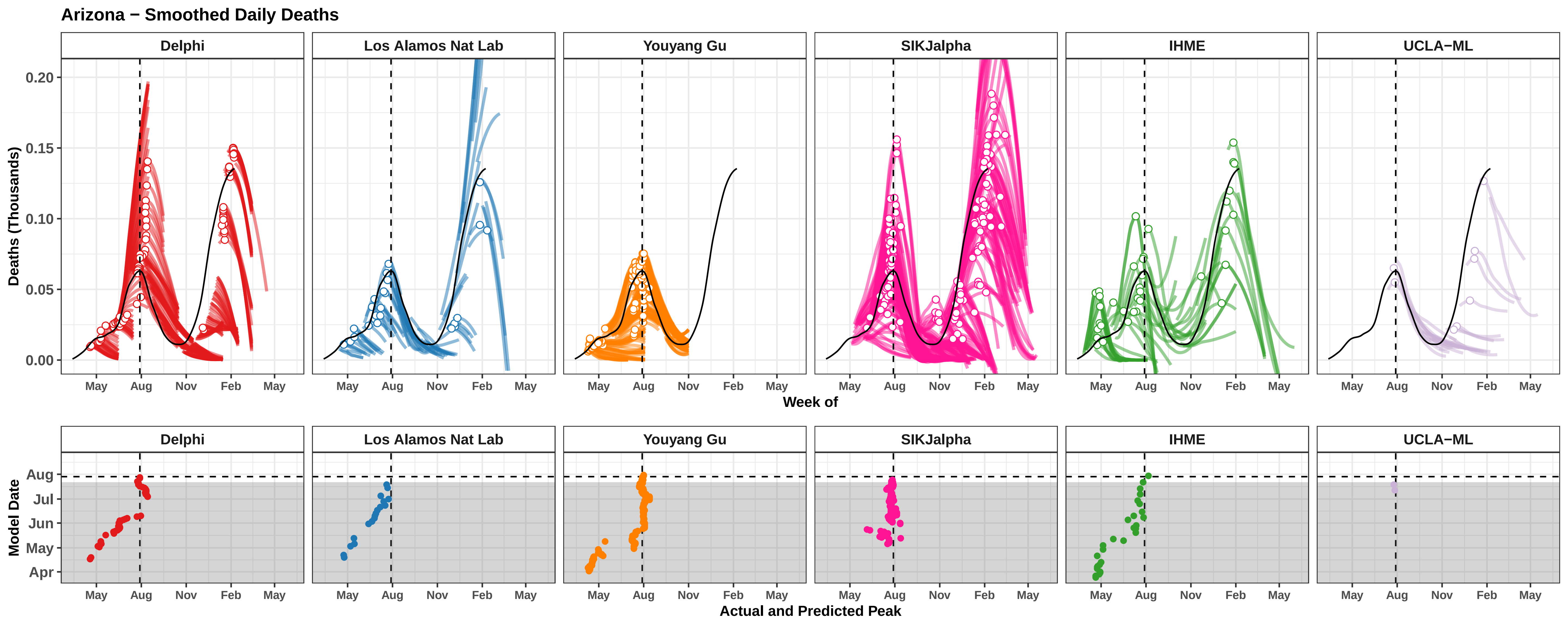

Arizona − Smoothed Daily Deaths
Delphi
Los Alamos Nat Lab
Youyang Gu
SIKJalpha
IHME
UCLA−ML
Deaths (Thousands)
0.00
0.05
0.10
0.15
0.20
May
Aug
Nov
Feb
May
Week of
Model Date
Apr
May
Jun
Jul
Aug
Actual and Predicted Peak

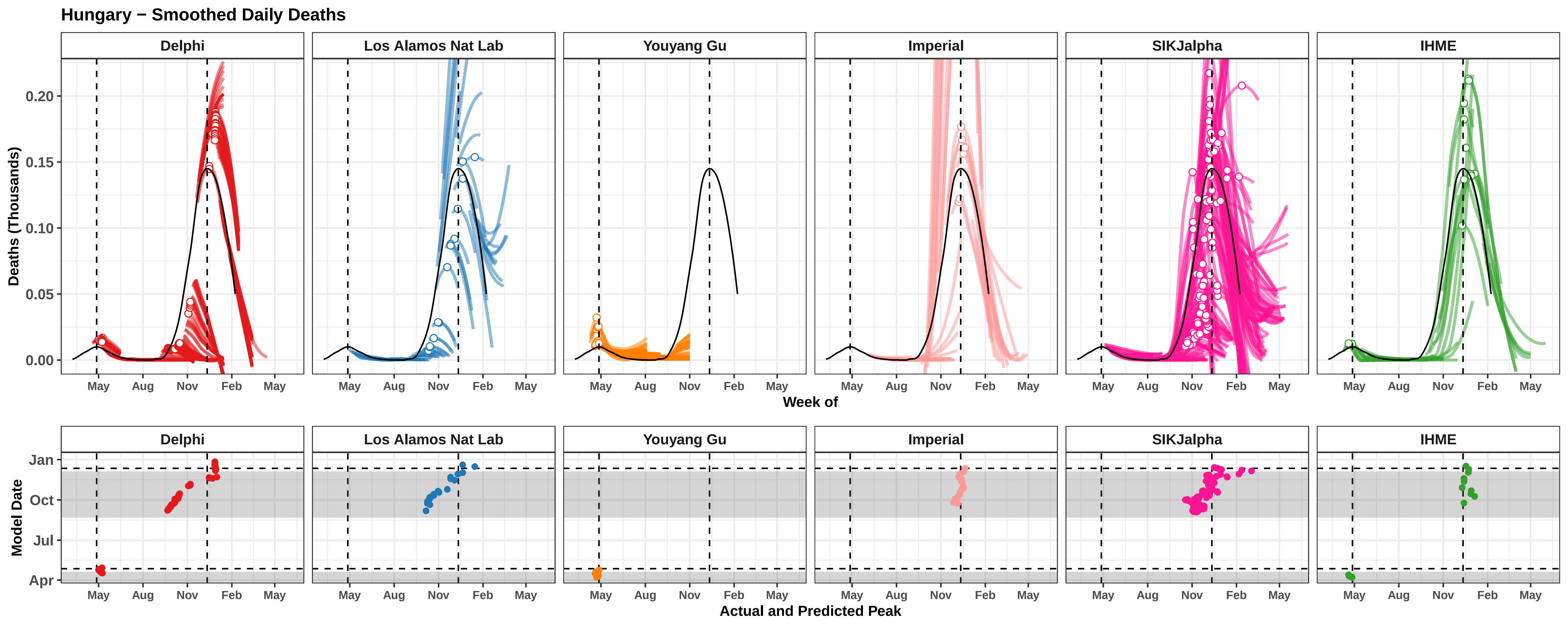

Hungary − Smoothed Daily Deaths
Delphi
Los Alamos Nat Lab
Youyang Gu
Imperial
SIKJalpha
IHME
Deaths (Thousands)
0.00
0.05
0.10
0.15
0.20
May
Aug
Nov
Feb
May
Week of
Model Date
Jan
Oct
Jul
Apr
Actual and Predicted Peak

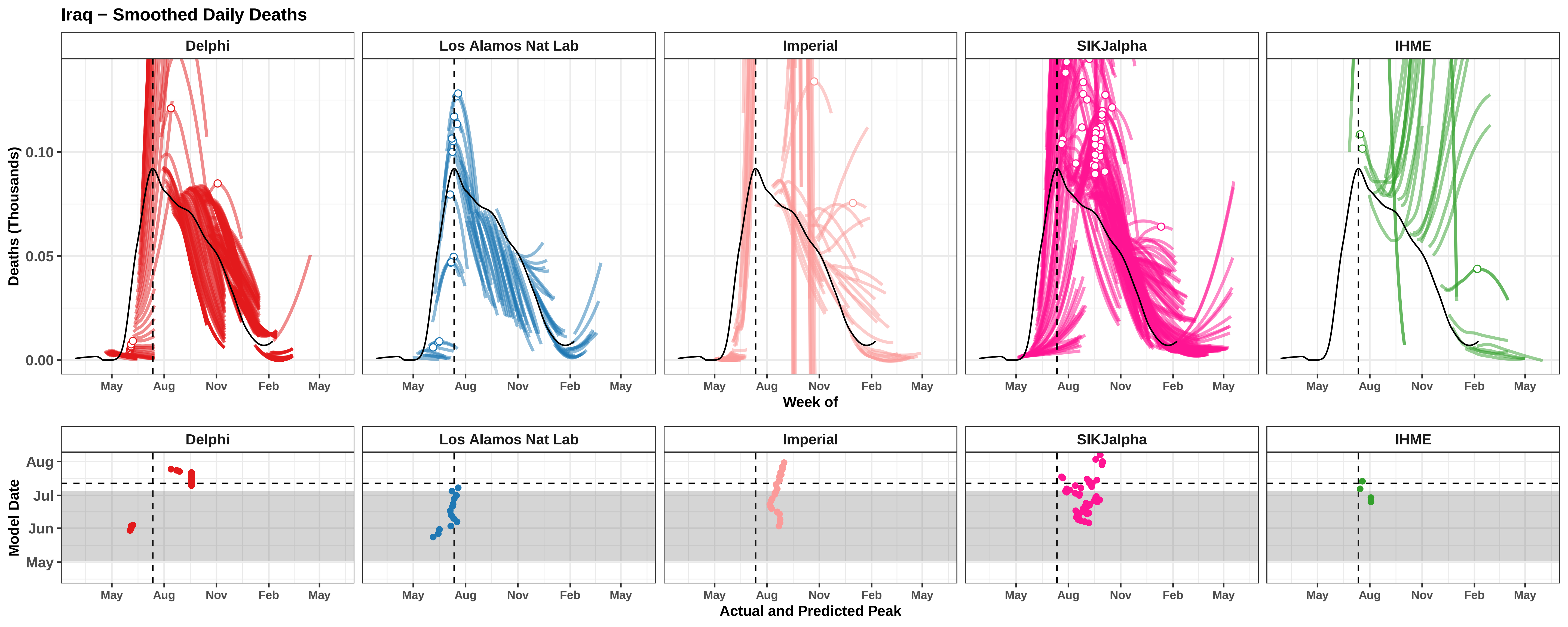

Iraq − Smoothed Daily Deaths
Delphi
Los Alamos Nat Lab
Imperial
SIKJalpha
IHME
Deaths (Thousands)
0.10
0.05
0.00
May
Aug
Nov
Feb
May
Week of
Model Date
Aug
Jul
Jun
May
Actual and Predicted Peak

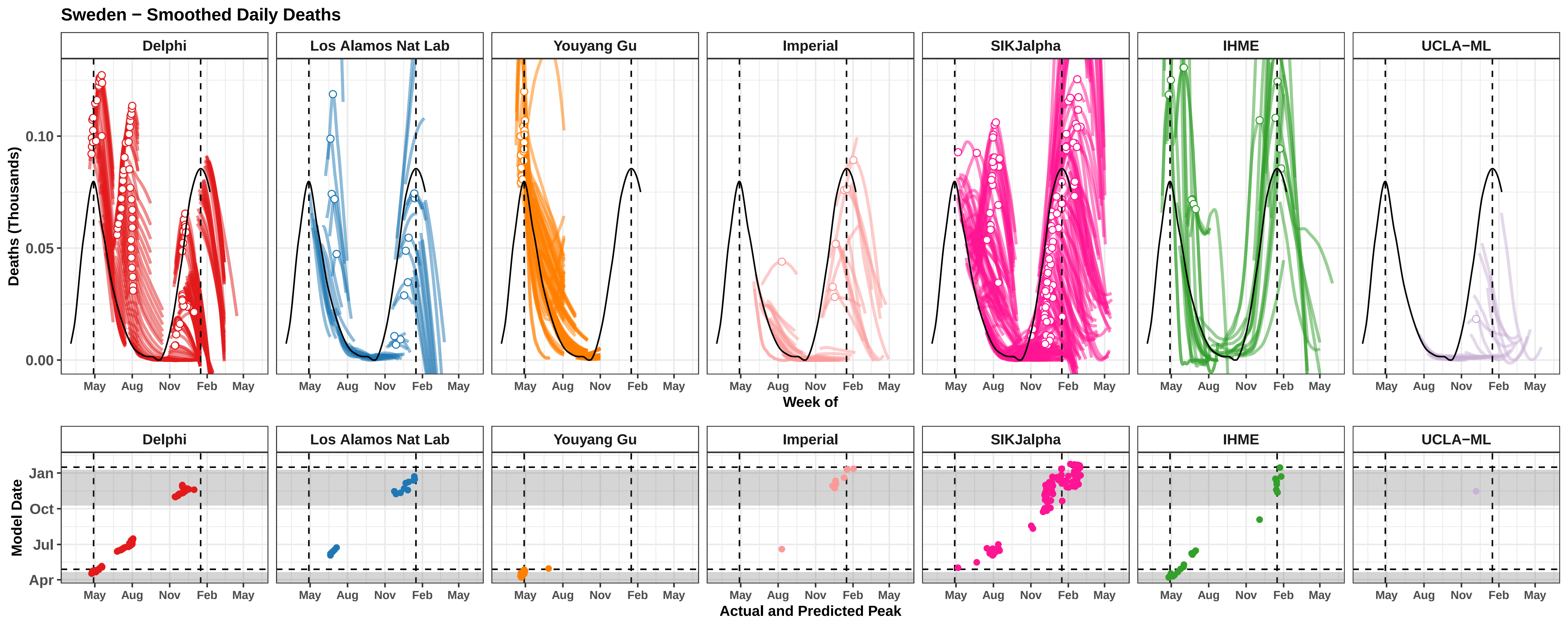

Sweden − Smoothed Daily Deaths
Delphi
Los Alamos Nat Lab
Youyang Gu
Imperial
SIKJalpha
IHME
UCLA−ML
Deaths (Thousands)
0.10
0.05
0.00
May
Aug
Nov
Feb
May
Week of
Model Date
Jan
Oct
Jul
Apr
Actual and Predicted Peak

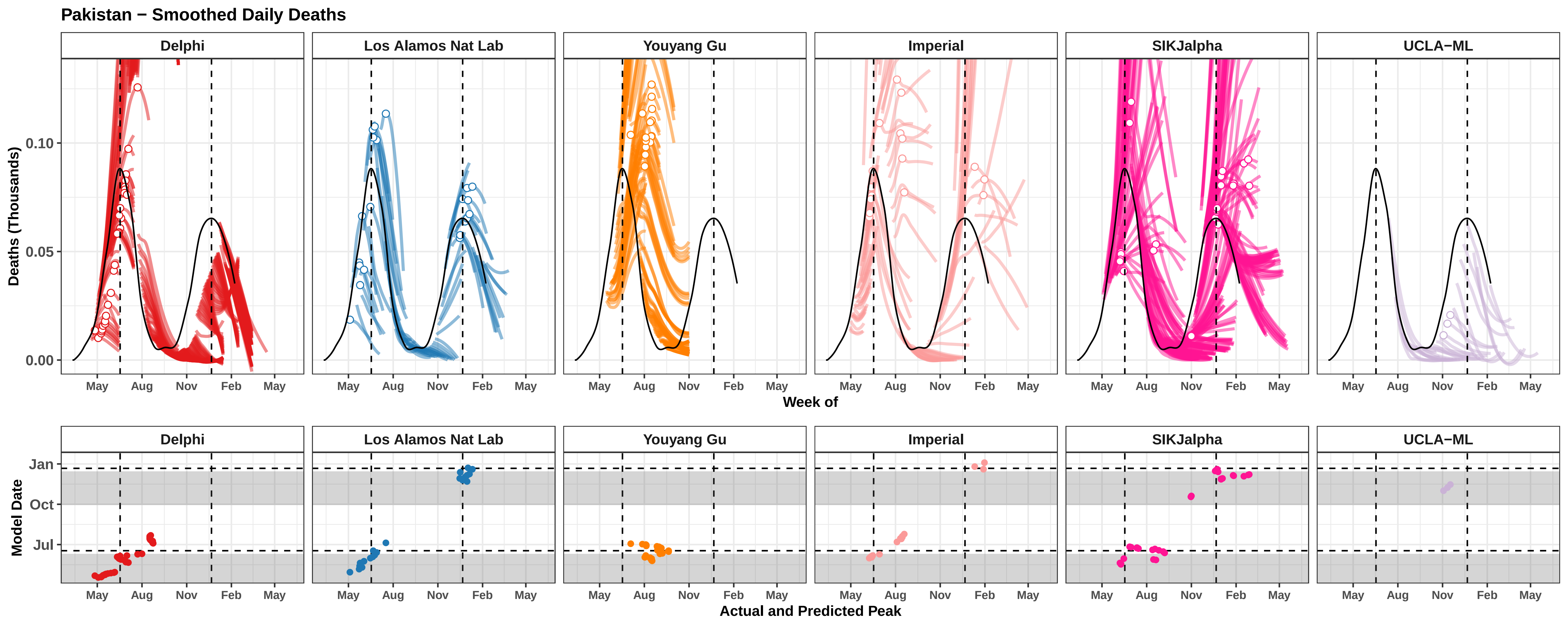

Pakistan − Smoothed Daily Deaths
Delphi
Los Alamos Nat Lab
Youyang Gu
Imperial
SIKJalpha
UCLA−ML
Deaths (Thousands)
0.10
0.05
0.00
May
Aug
Nov
Feb
May
Week of
Model Date
Jan
Oct
Jul
Actual and Predicted Peak

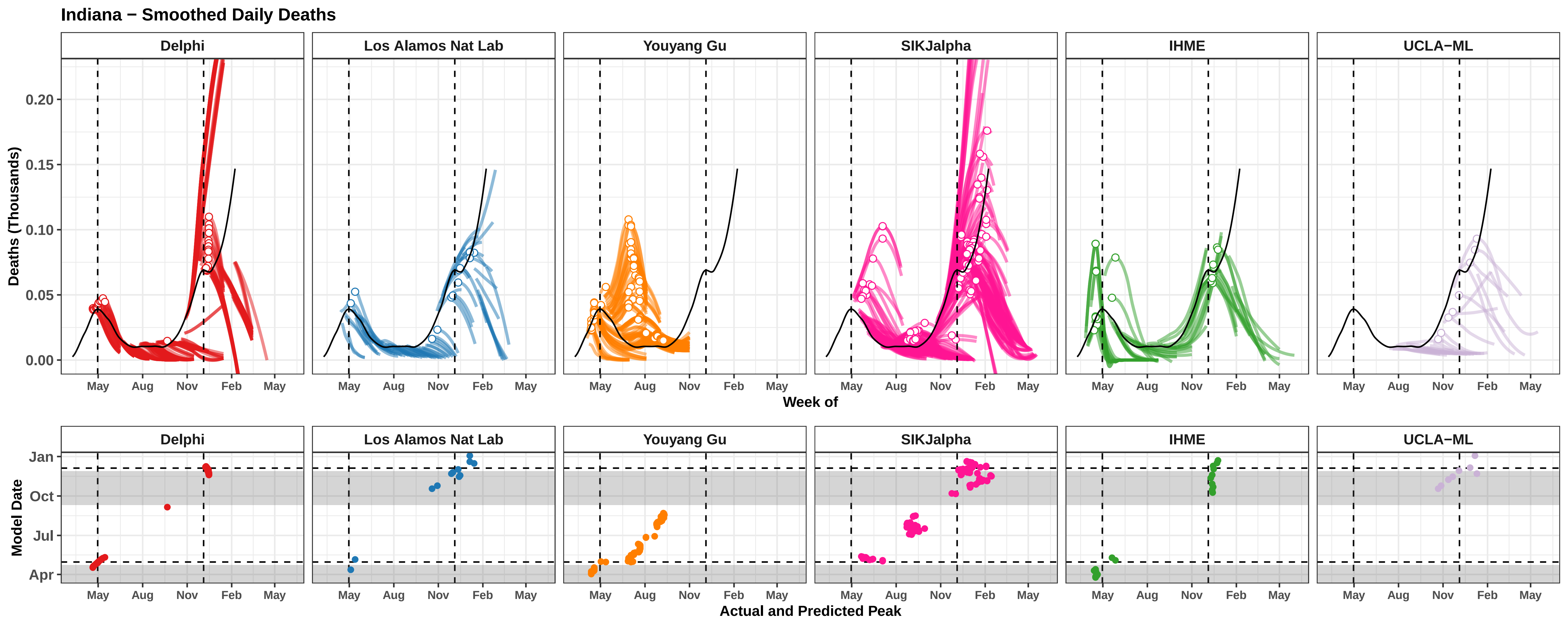

Indiana − Smoothed Daily Deaths
Delphi
Los Alamos Nat Lab
Youyang Gu
SIKJalpha
IHME
UCLA−ML
Deaths (Thousands)
0.20
0.15
0.10
0.05
0.00
May
Aug
Nov
Feb
May
Week of
Model Date
Jan
Oct
Jul
Apr
Actual and Predicted Peak

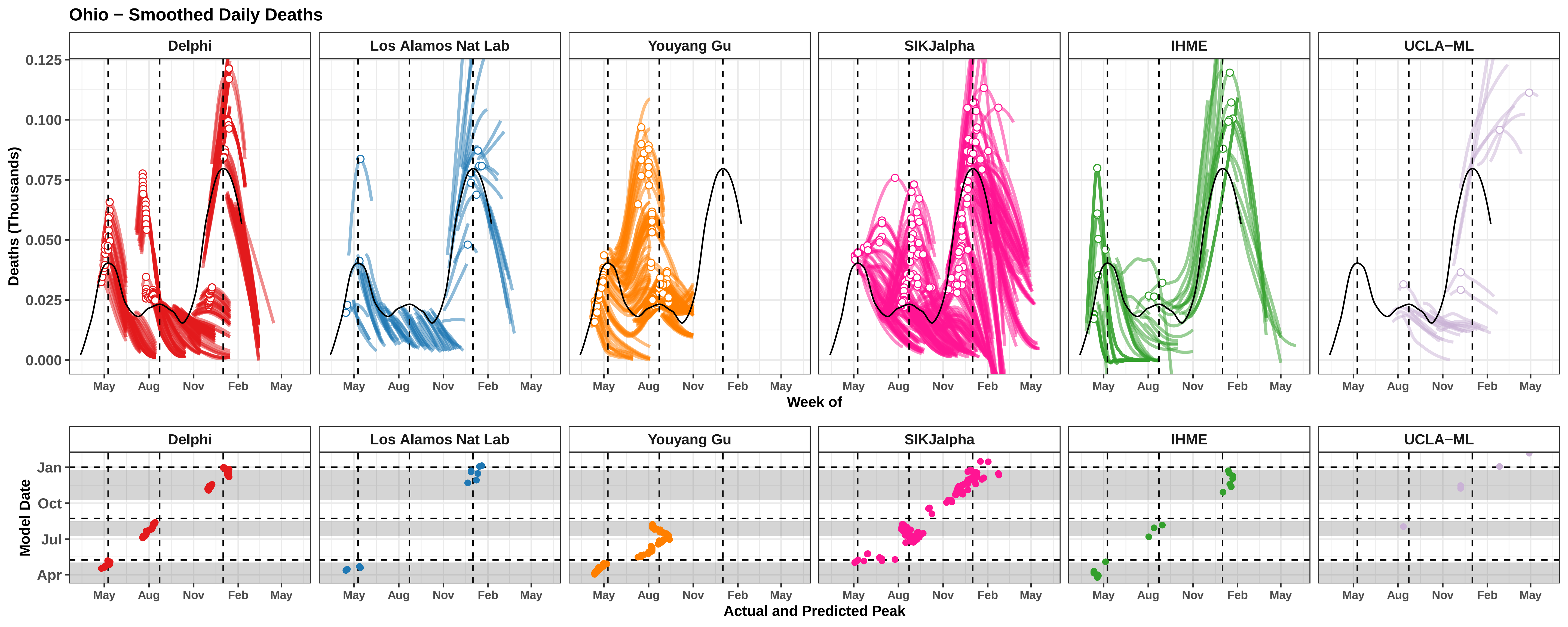
Ohio − Smoothed Daily Deaths
Delphi
Los Alamos Nat Lab
Youyang Gu
SIKJalpha
IHME
UCLA−ML
Deaths (Thousands)
0.125
0.100
0.075
0.050
0.025
0.000
May
Aug
Nov
Feb
May
Week of
Model Date
Jan
Oct
Jul
Apr
Actual and Predicted Peak

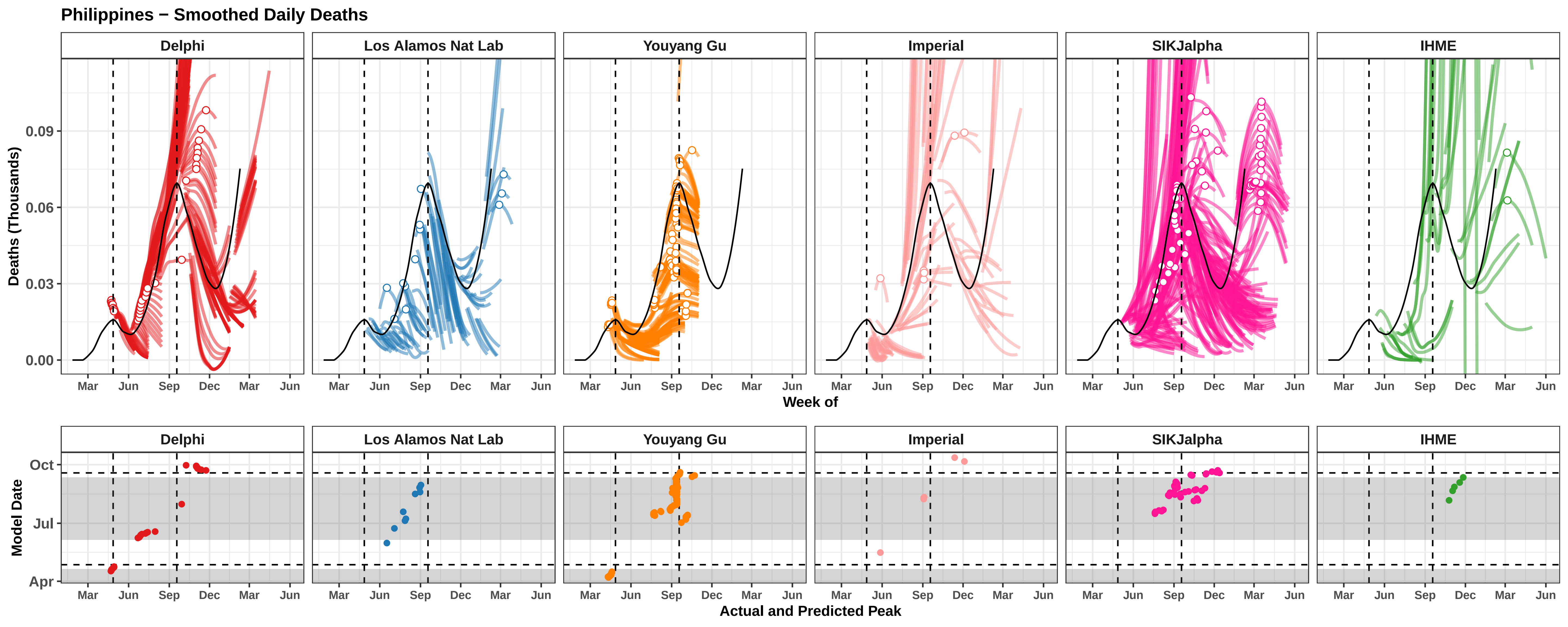

Philippines − Smoothed Daily Deaths
Delphi
Los Alamos Nat Lab
Youyang Gu
Imperial
SIKJalpha
IHME
Deaths (Thousands)
0.00
0.03
0.06
0.09
Mar
Jun
Sep
Dec
Week of
Model Date
Apr
Jul
Oct
Actual and Predicted Peak

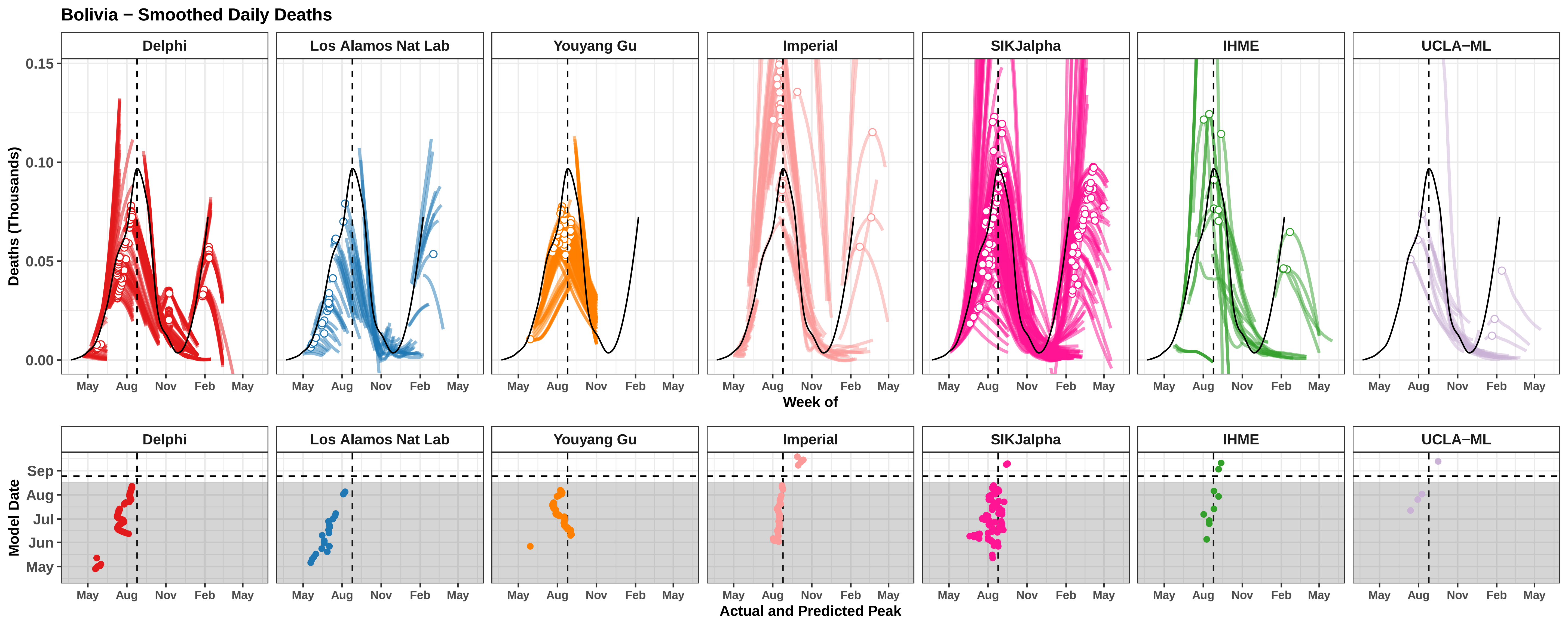
Bolivia − Smoothed Daily Deaths
Delphi
Los Alamos Nat Lab
Youyang Gu
Imperial
SIKJalpha
IHME
UCLA−ML
Deaths (Thousands)
0.15
0.10
0.05
0.00
May
Aug
Nov
Feb
May
Week of
Model Date
Sep
Aug
Jul
Jun
May
Actual and Predicted Peak

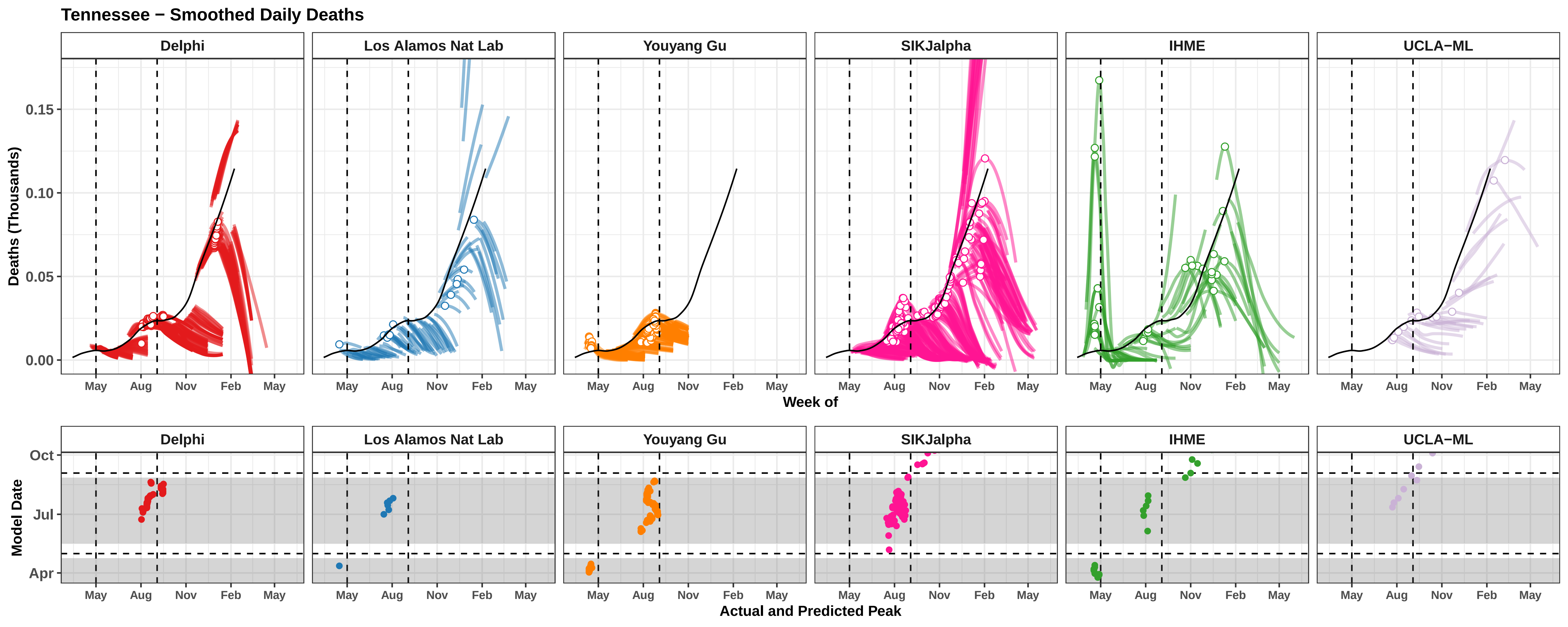

Tennessee − Smoothed Daily Deaths
Delphi
Los Alamos Nat Lab
Youyang Gu
SIKJalpha
IHME
UCLA−ML
Deaths (Thousands)
0.00
0.05
0.10
0.15
May
Aug
Nov
Feb
May
Week of
Model Date
Oct
Jul
Apr
Actual and Predicted Peak

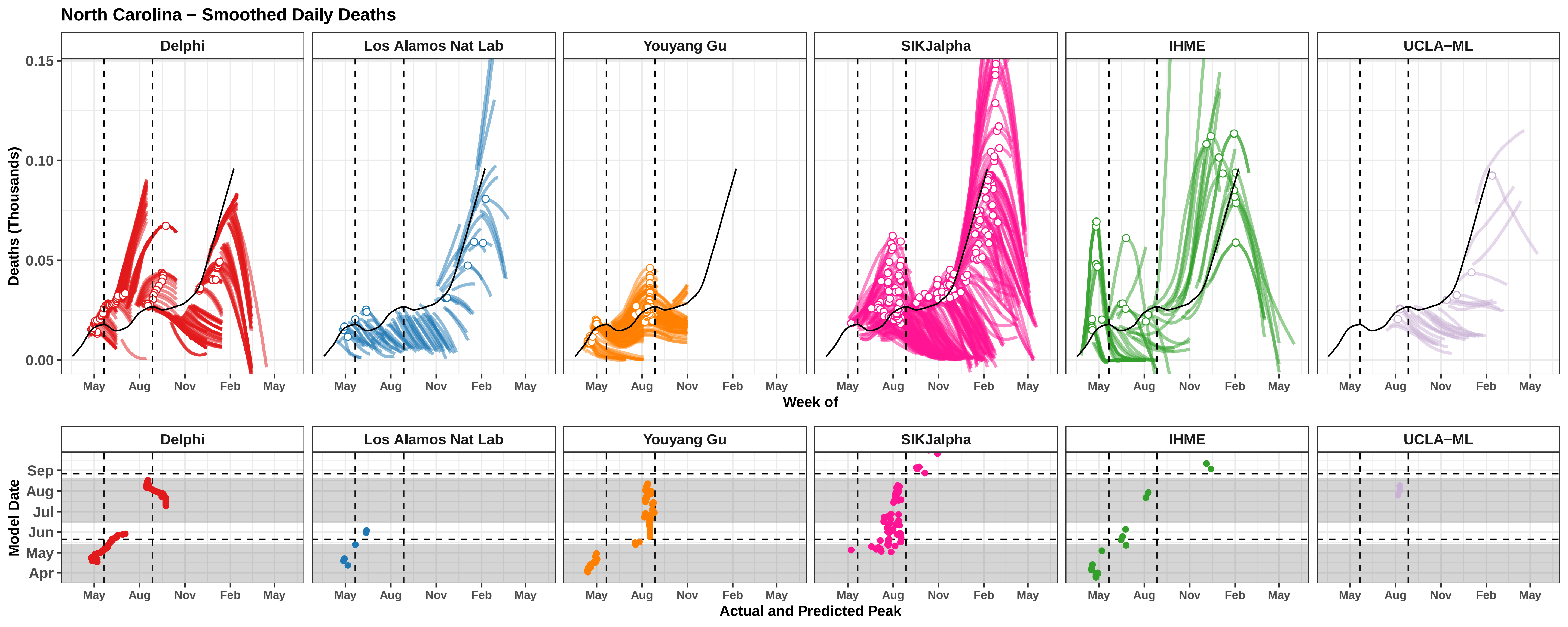

North Carolina − Smoothed Daily Deaths
Delphi
Los Alamos Nat Lab
Youyang Gu
SIKJalpha
IHME
UCLA−ML
Deaths (Thousands)
0.15
0.10
0.05
0.00
May
Aug
Nov
Feb
May
Week of
Model Date
Sep
Aug
Jul
Jun
May
Apr
Actual and Predicted Peak

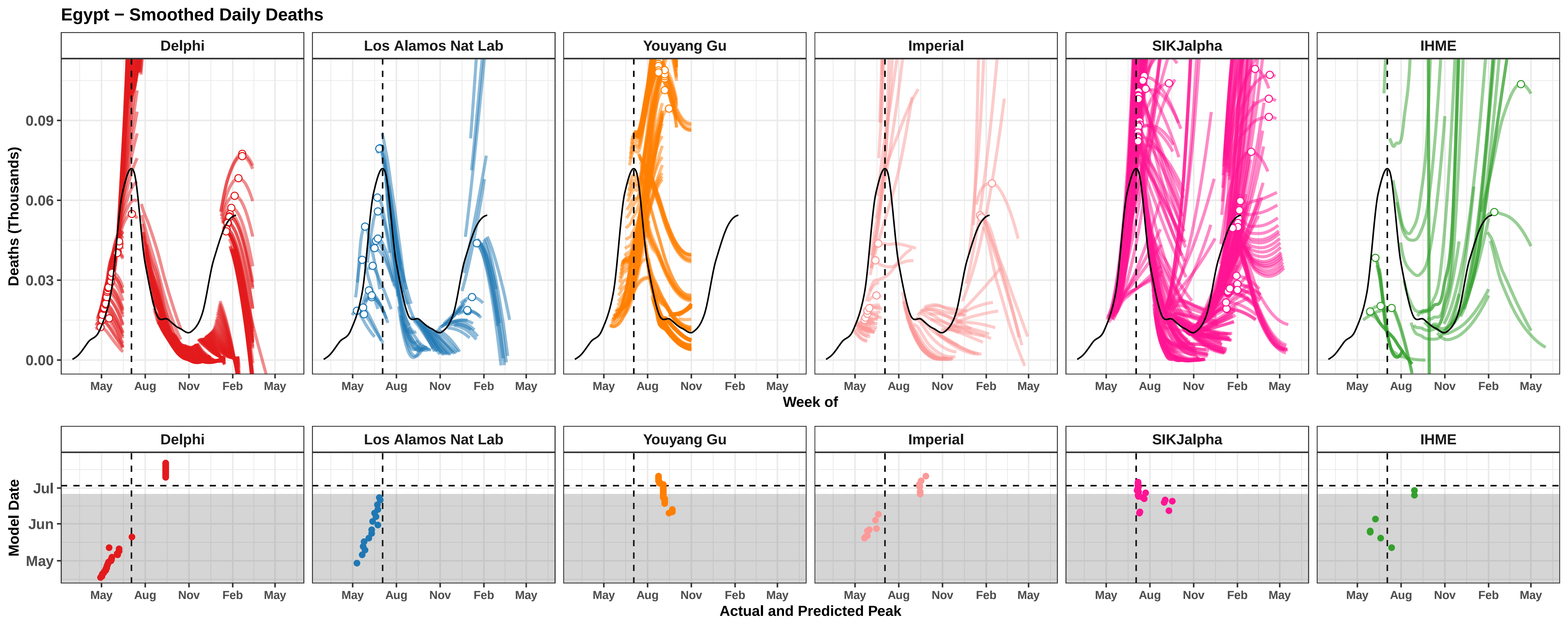
Egypt − Smoothed Daily Deaths
Delphi
Los Alamos Nat Lab
Youyang Gu
Imperial
SIKJalpha
IHME
Deaths (Thousands)
0.00
0.03
0.06
0.09
May
Aug
Nov
Feb
May
Week of
Model Date
May
Jun
Jul
Actual and Predicted Peak

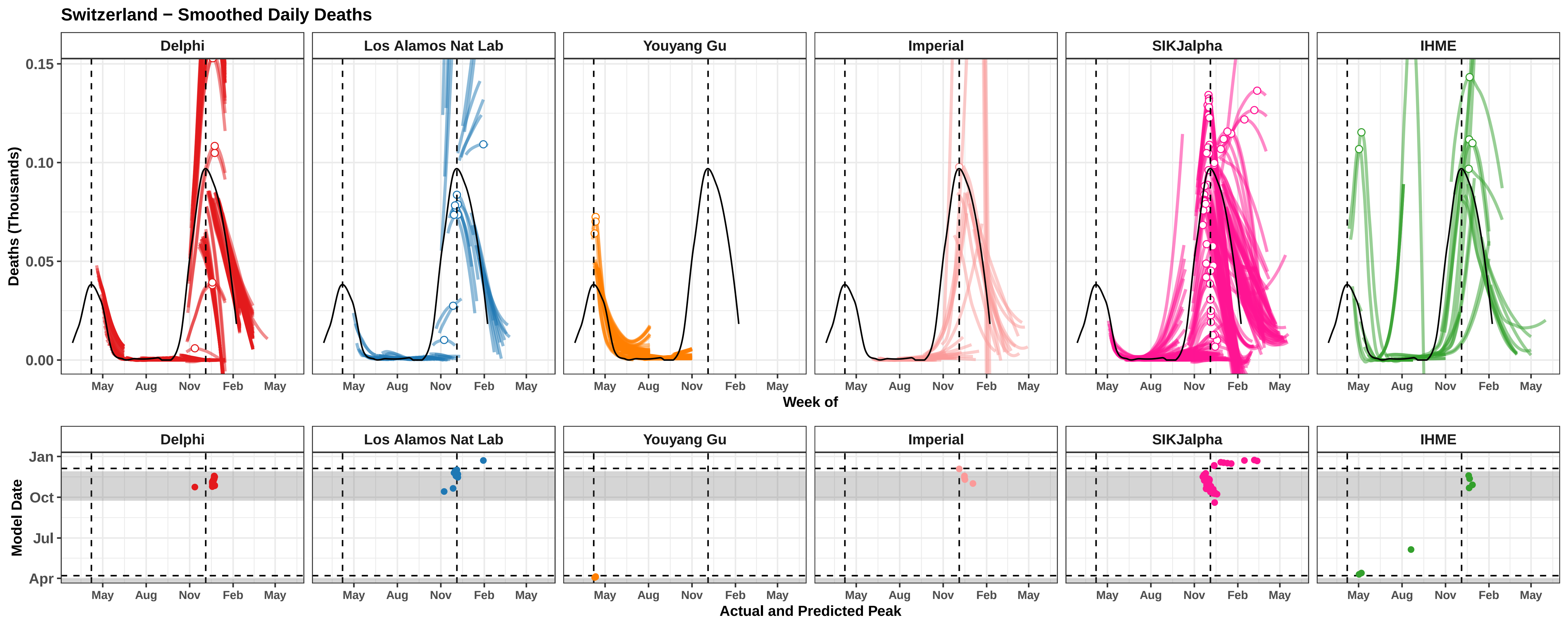

Switzerland − Smoothed Daily Deaths
Delphi
Los Alamos Nat Lab
Youyang Gu
Imperial
SIKJalpha
IHME
Deaths (Thousands)
0.15
0.10
0.05
0.00
May
Aug
Nov
Feb
May
Week of
Model Date
Jan
Oct
Jul
Apr
Actual and Predicted Peak

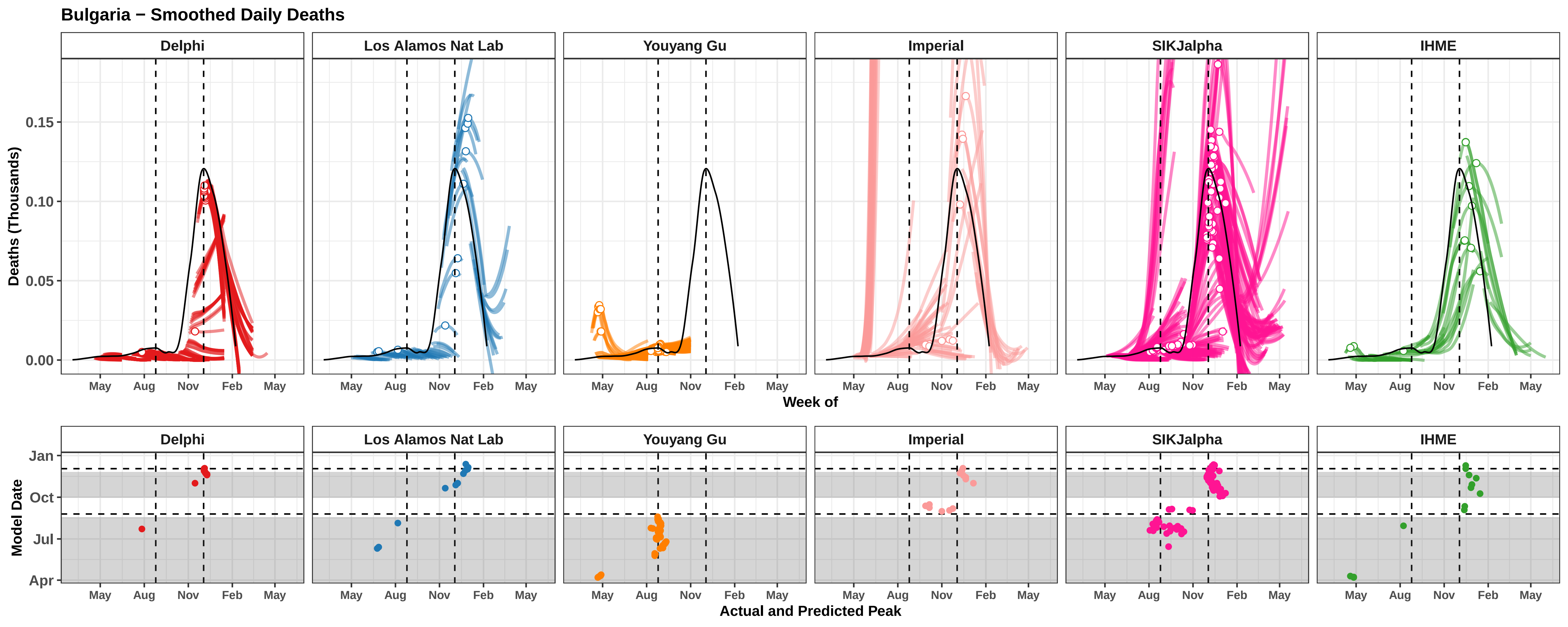

Bulgaria − Smoothed Daily Deaths
Delphi
Los Alamos Nat Lab
Youyang Gu
Imperial
SIKJalpha
IHME
Deaths (Thousands)
0.00
0.05
0.10
0.15
May
Aug
Nov
Feb
May
Week of
Model Date
Jan
Oct
Jul
Apr
Actual and Predicted Peak

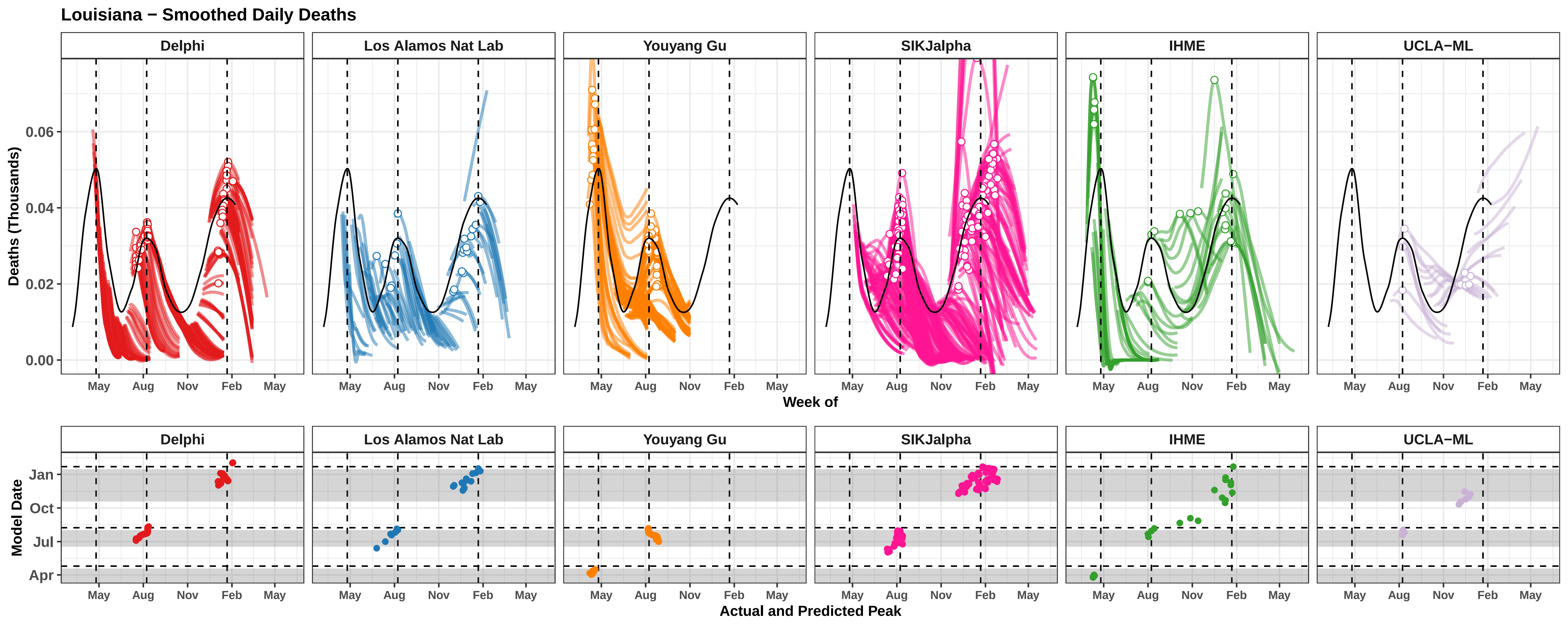
Louisiana − Smoothed Daily Deaths
Delphi
Los Alamos Nat Lab
Youyang Gu
SIKJalpha
IHME
UCLA−ML
Deaths (Thousands)
0.06
0.04
0.02
0.00
May
Aug
Nov
Feb
May
Week of
Model Date
Jan
Oct
Jul
Apr
Actual and Predicted Peak

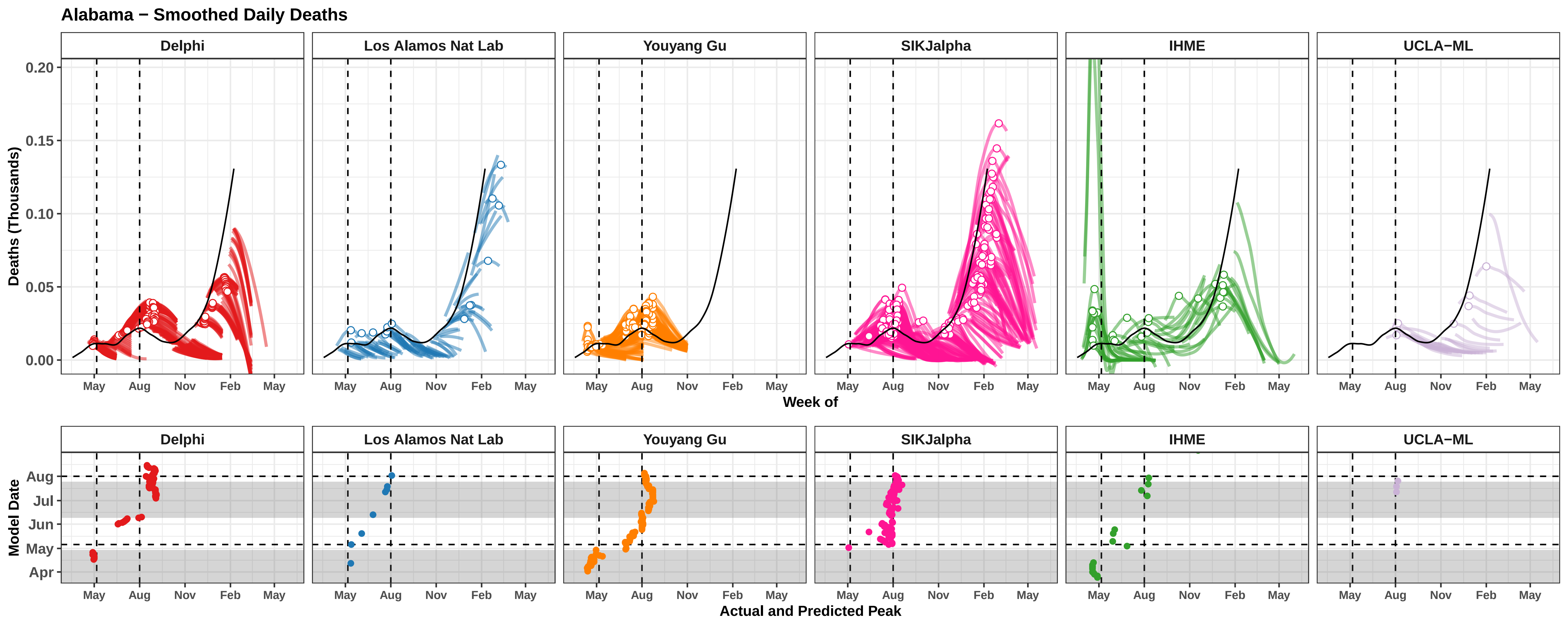

Alabama − Smoothed Daily Deaths
Delphi
Los Alamos Nat Lab
Youyang Gu
SIKJalpha
IHME
UCLA−ML
Deaths (Thousands)
0.00
0.05
0.10
0.15
0.20
May
Aug
Nov
Feb
May
Week of
Model Date
Apr
May
Jun
Jul
Aug
Actual and Predicted Peak

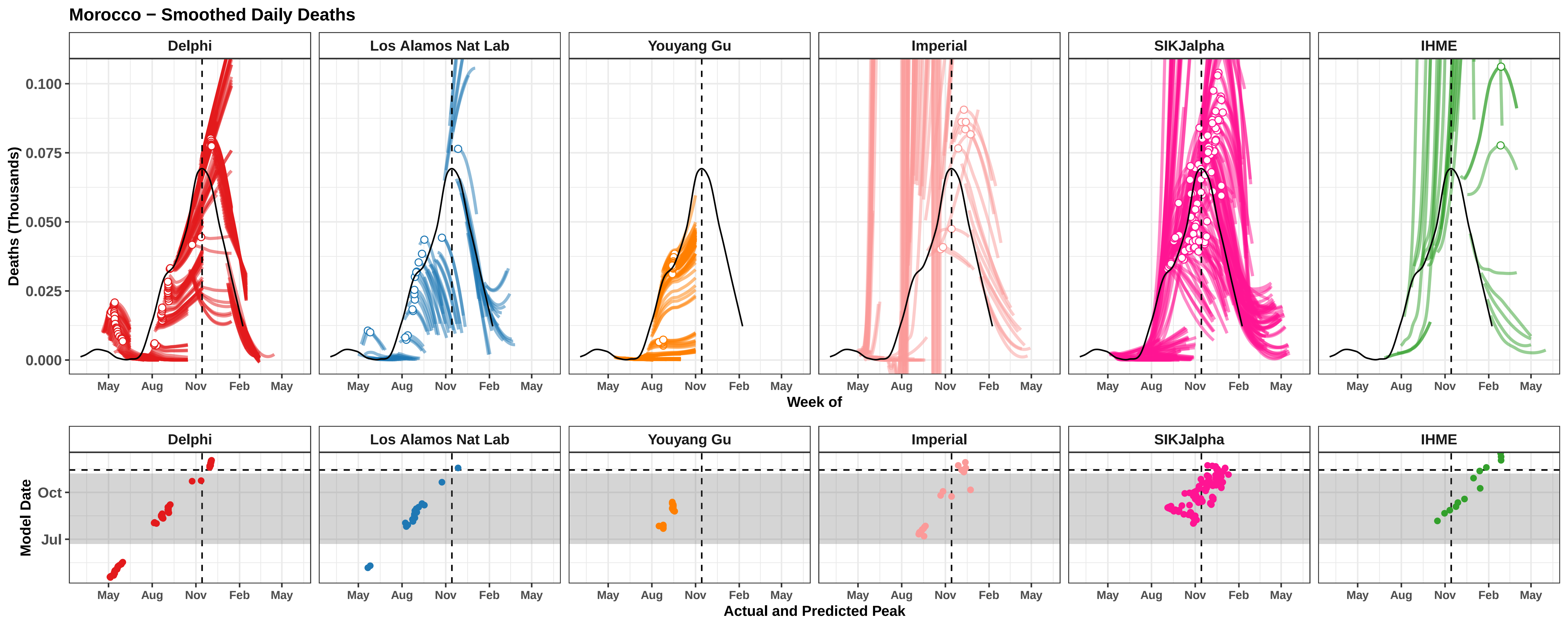
Morocco − Smoothed Daily Deaths
Delphi
Los Alamos Nat Lab
Youyang Gu
Imperial
SIKJalpha
IHME
Deaths (Thousands)
0.100
0.075
0.050
0.025
0.000
May
Aug
Nov
Feb
May
Week of
Model Date
Oct
Jul
Actual and Predicted Peak

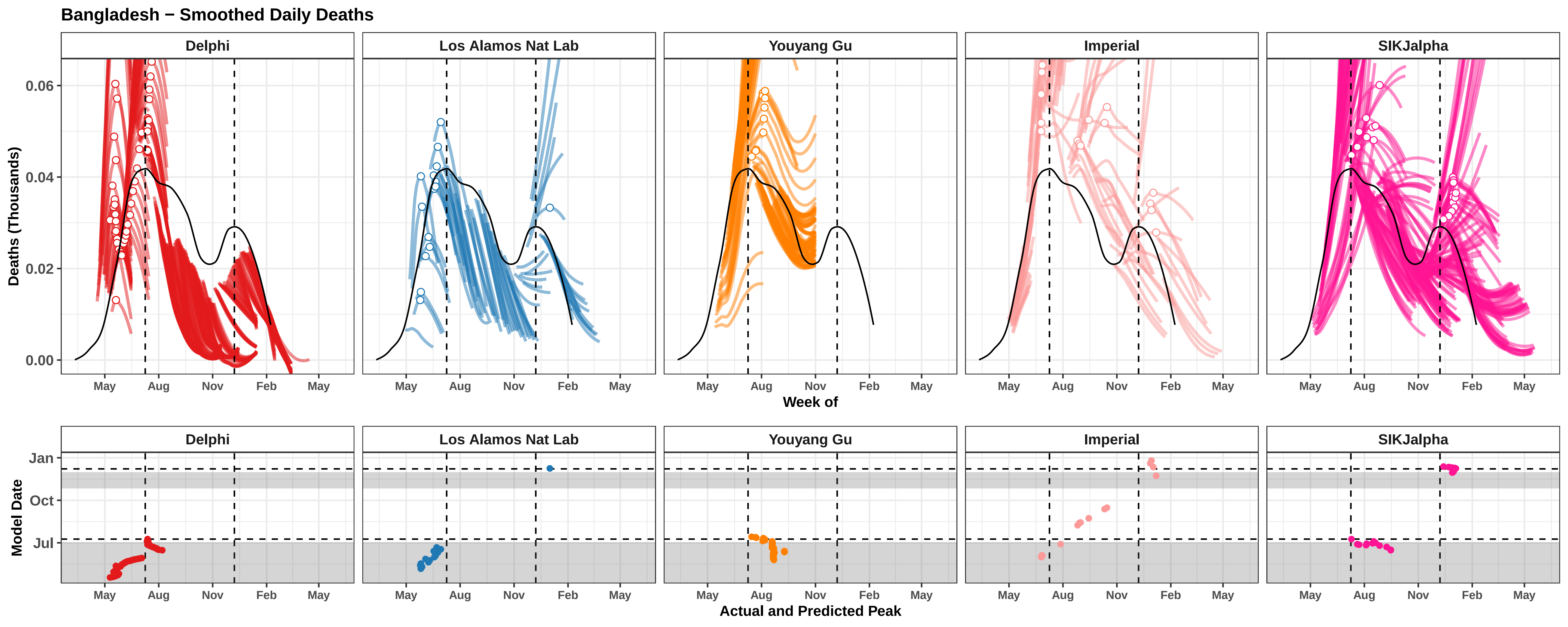

Bangladesh − Smoothed Daily Deaths
Delphi
Los Alamos Nat Lab
Youyang Gu
Imperial
SIKJalpha
Deaths (Thousands)
0.06
0.04
0.02
0.00
May
Aug
Nov
Feb
May
Week of
Model Date
Jan
Oct
Jul
Actual and Predicted Peak

Austria − Smoothed Daily Deaths

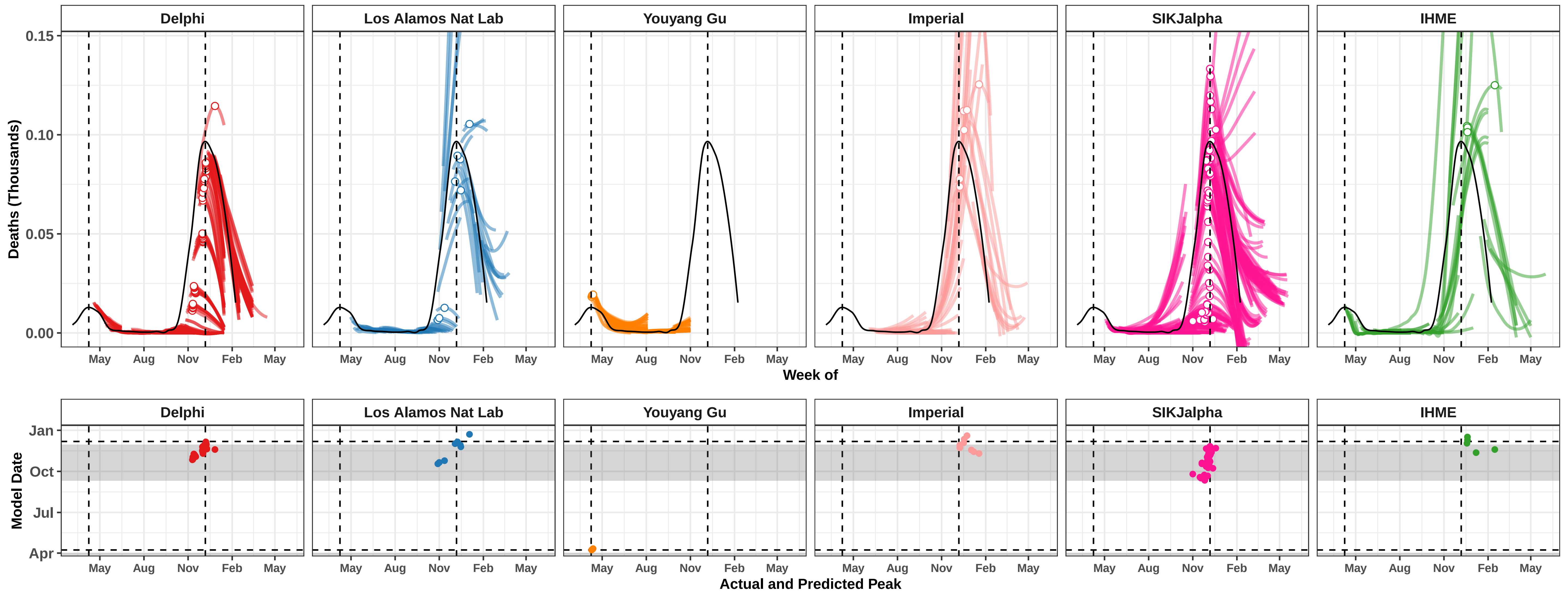

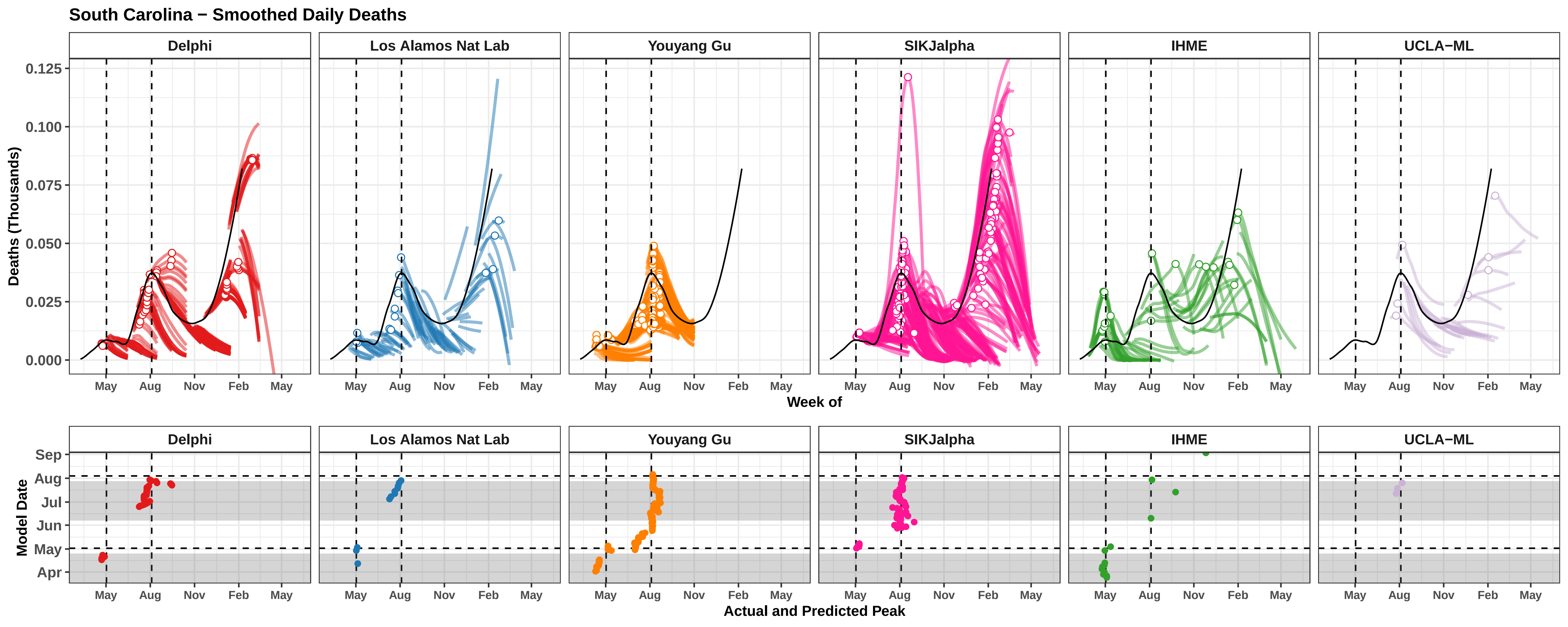

South Carolina − Smoothed Daily Deaths
Delphi
Los Alamos Nat Lab
Youyang Gu
SIKJalpha
IHME
UCLA−ML
Deaths (Thousands)
0.125
0.100
0.075
0.050
0.025
0.000
May
Aug
Nov
Feb
May
Week of
Model Date
Sep
Aug
Jul
Jun
May
Apr
Actual and Predicted Peak

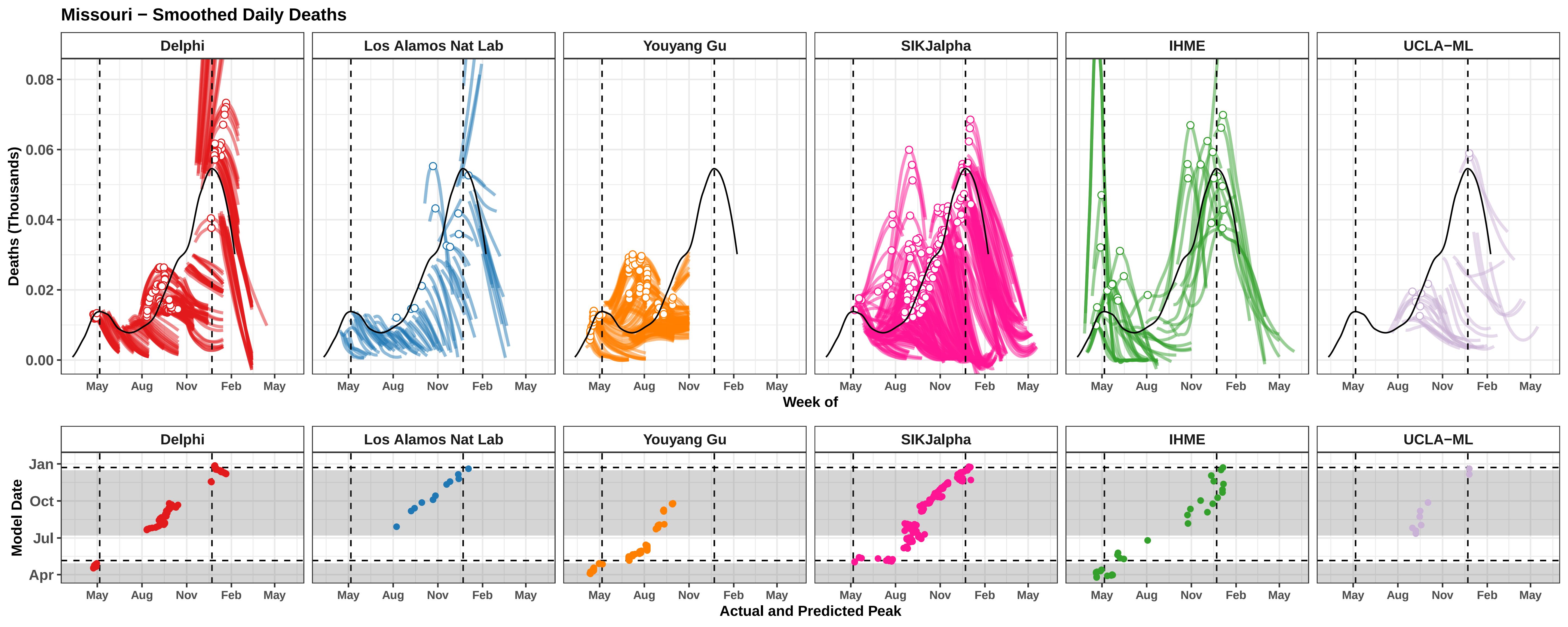

Missouri − Smoothed Daily Deaths
Delphi
Los Alamos Nat Lab
Youyang Gu
SIKJalpha
IHME
UCLA−ML
Deaths (Thousands)
0.00
0.02
0.04
0.06
0.08
May
Aug
Nov
Feb
May
Week of
Model Date
Jan
Oct
Jul
Apr
Actual and Predicted Peak

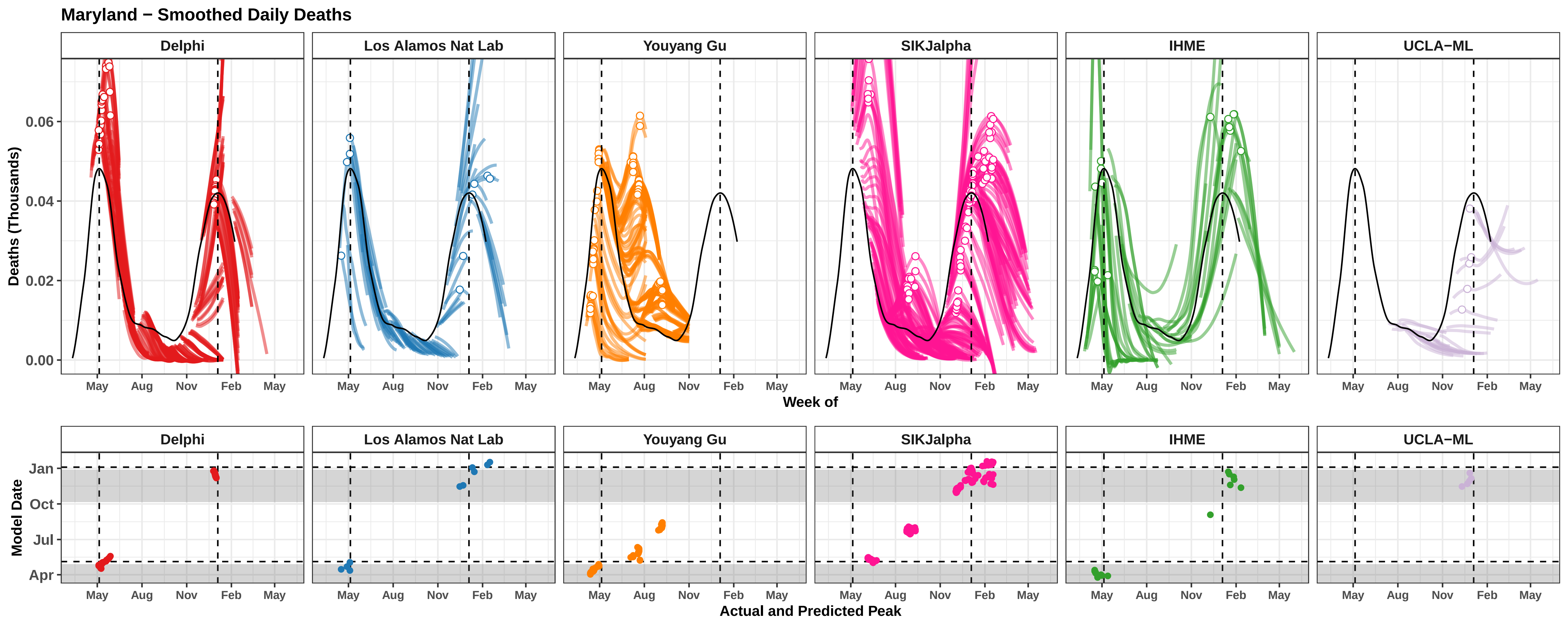

Maryland − Smoothed Daily Deaths
Delphi
Los Alamos Nat Lab
Youyang Gu
SIKJalpha
IHME
UCLA−ML
Deaths (Thousands)
0.00
0.02
0.04
0.06
May
Aug
Nov
Feb
May
Week of
Model Date
Apr
Jul
Oct
Jan
Actual and Predicted Peak

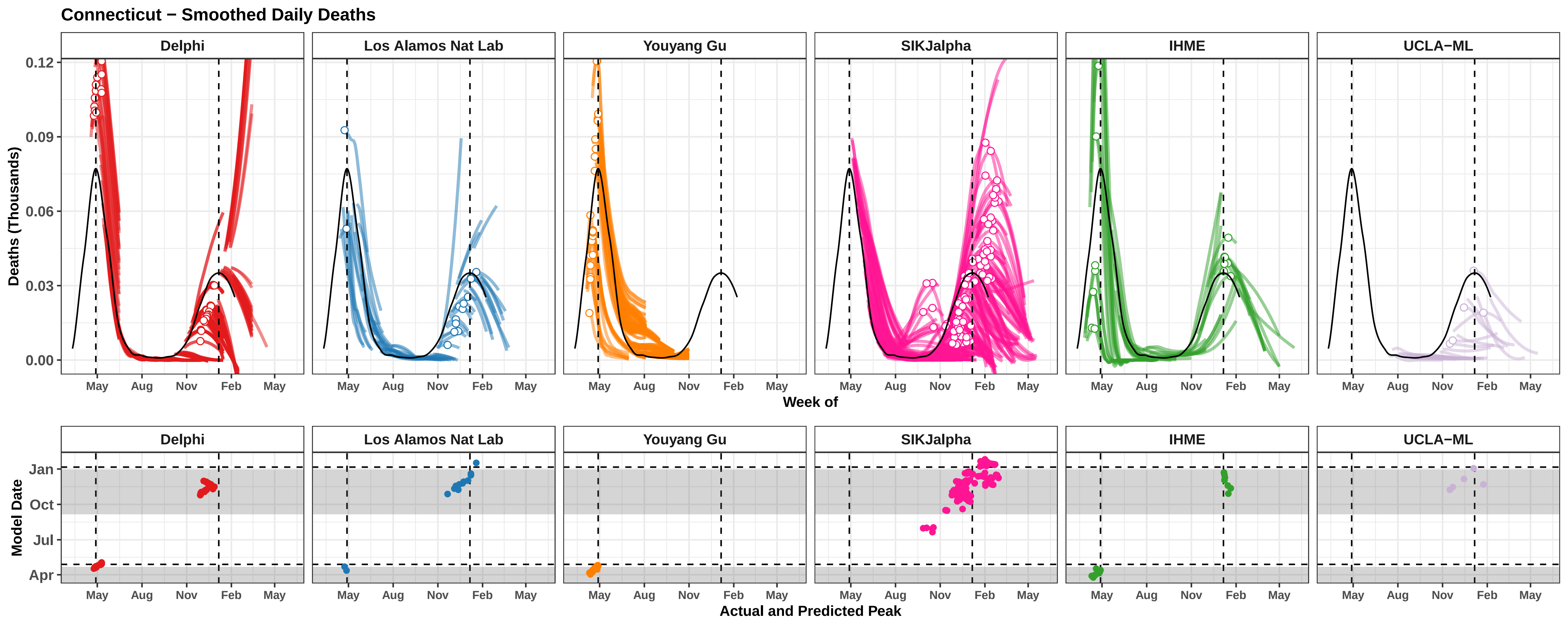

Connecticut − Smoothed Daily Deaths
Delphi
Los Alamos Nat Lab
Youyang Gu
SIKJalpha
IHME
UCLA−ML
Deaths (Thousands)
0.12
0.09
0.06
0.03
0.00
May
Aug
Nov
Feb
May
Week of
Model Date
Jan
Oct
Jul
Apr
Actual and Predicted Peak

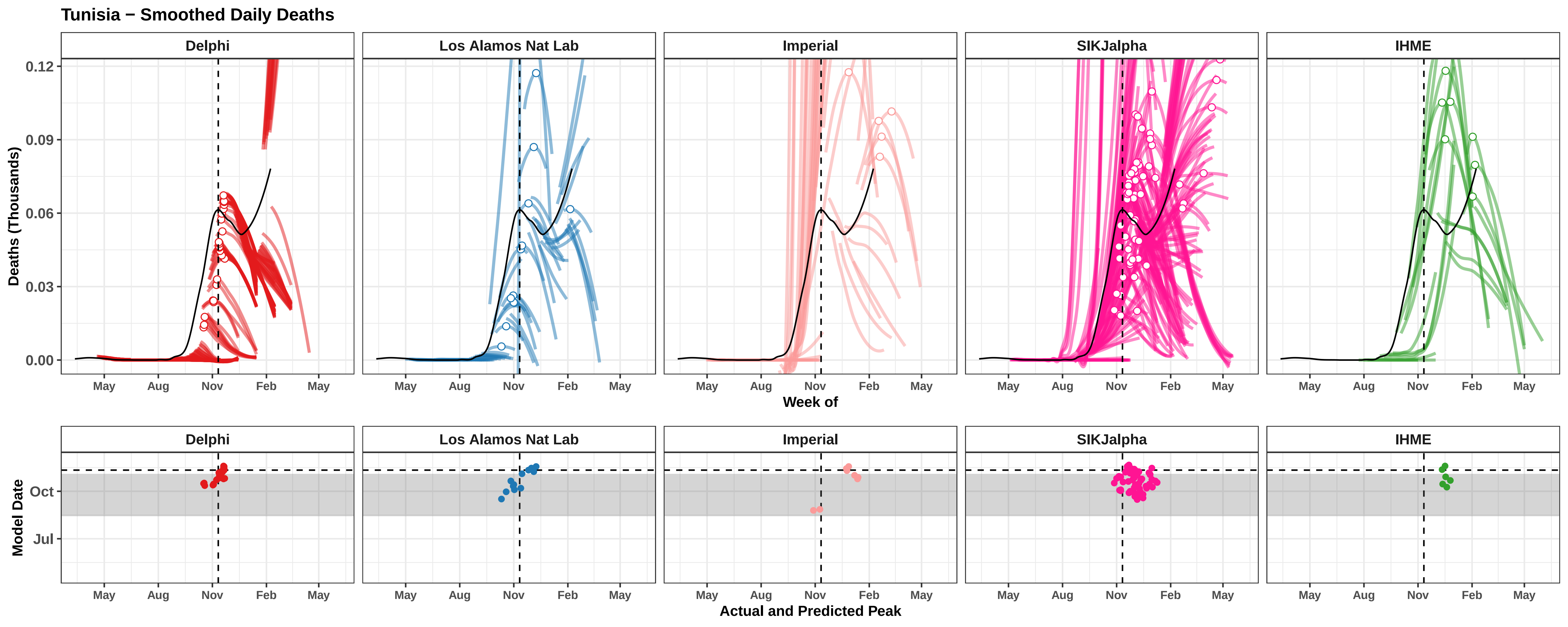

Tunisia − Smoothed Daily Deaths
Delphi
Los Alamos Nat Lab
Imperial
SIKJalpha
IHME
Deaths (Thousands)
0.12
0.09
0.06
0.03
0.00
May
Aug
Nov
Feb
May
Week of
Model Date
Oct
Jul
Actual and Predicted Peak

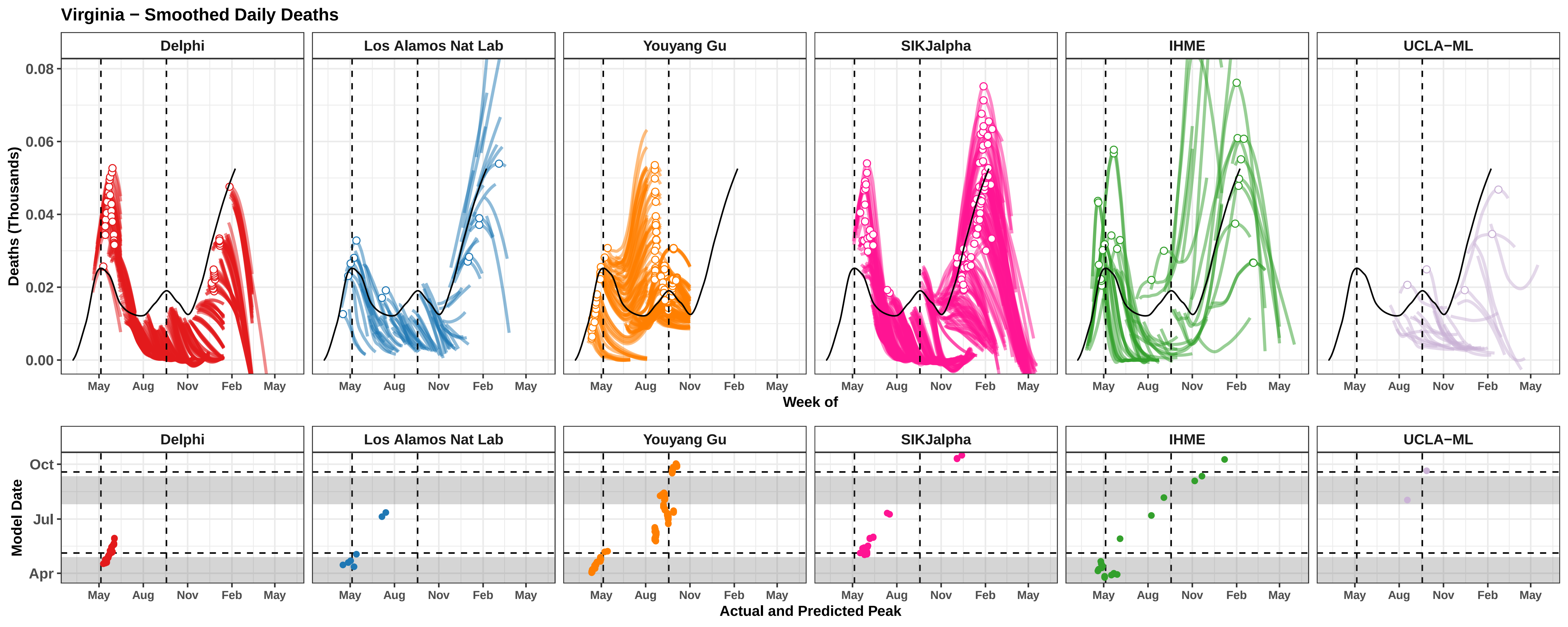

Virginia − Smoothed Daily Deaths
Delphi
Los Alamos Nat Lab
Youyang Gu
SIKJalpha
IHME
UCLA−ML
Deaths (Thousands)
0.00
0.02
0.04
0.06
0.08
May
Aug
Nov
Feb
May
Week of
Model Date
Apr
Jul
Oct
Actual and Predicted Peak

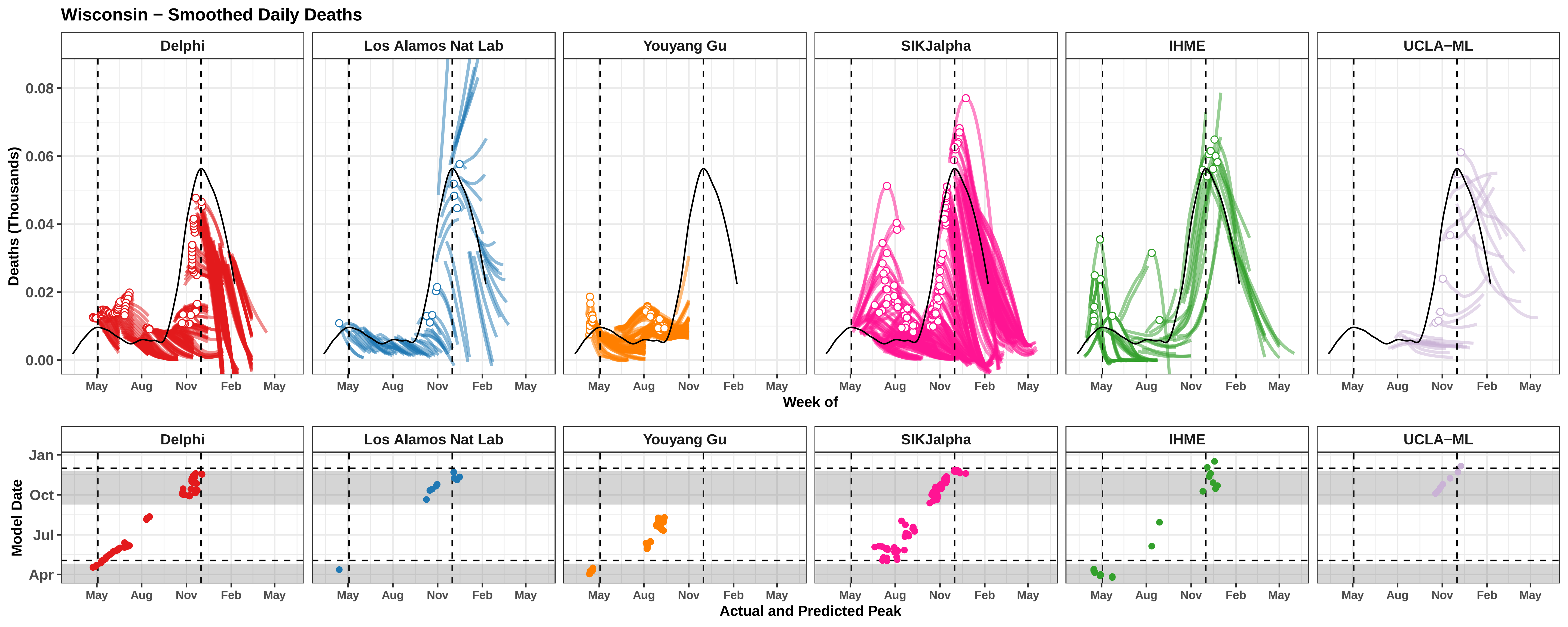

Wisconsin − Smoothed Daily Deaths
Delphi
Los Alamos Nat Lab
Youyang Gu
SIKJalpha
IHME
UCLA−ML
Deaths (Thousands)
0.00
0.02
0.04
0.06
0.08
May
Aug
Nov
Feb
May
Week of
Model Date
Apr
Jul
Oct
Jan
Actual and Predicted Peak

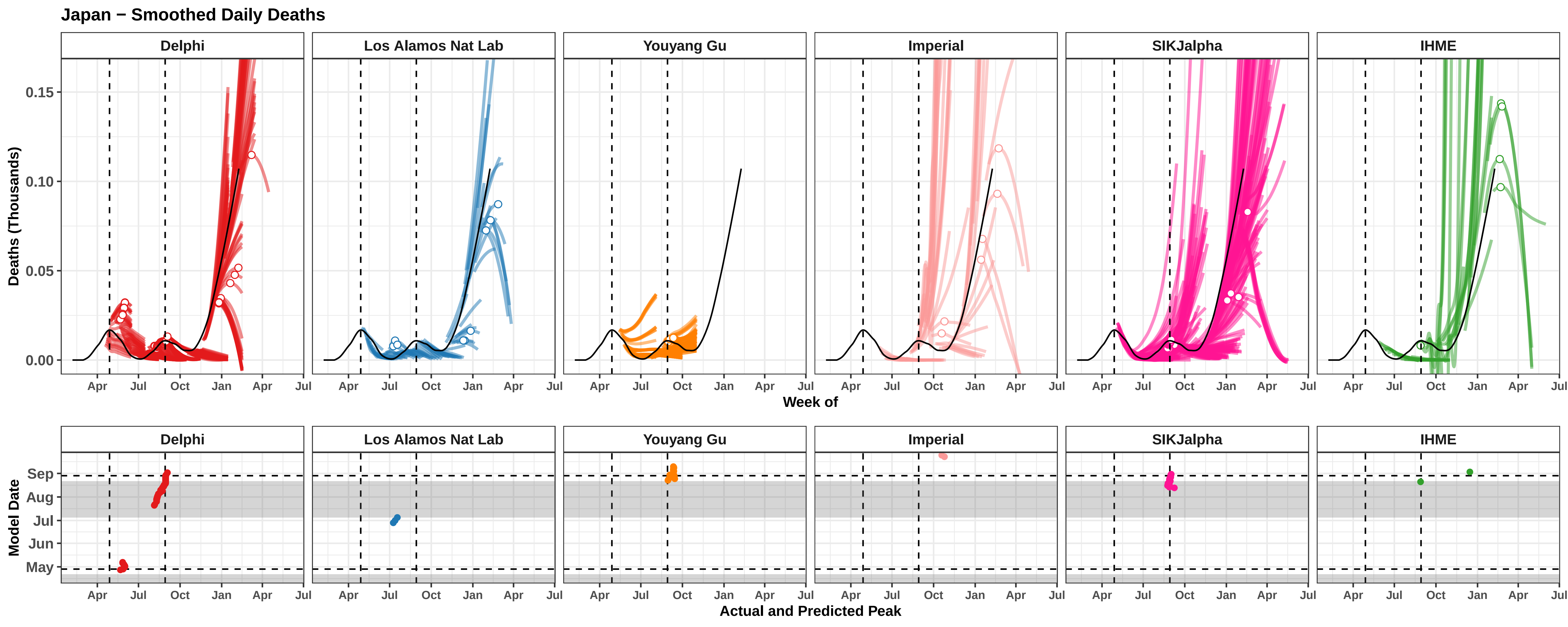
Japan − Smoothed Daily Deaths
Delphi
Los Alamos Nat Lab
Youyang Gu
Imperial
SIKJalpha
IHME
Deaths (Thousands)
0.00
0.05
0.10
0.15
Apr
Jul
Oct
Jan
Apr
Jul
Week of
Model Date
May
Jun
Jul
Aug
Sep
Actual and Predicted Peak

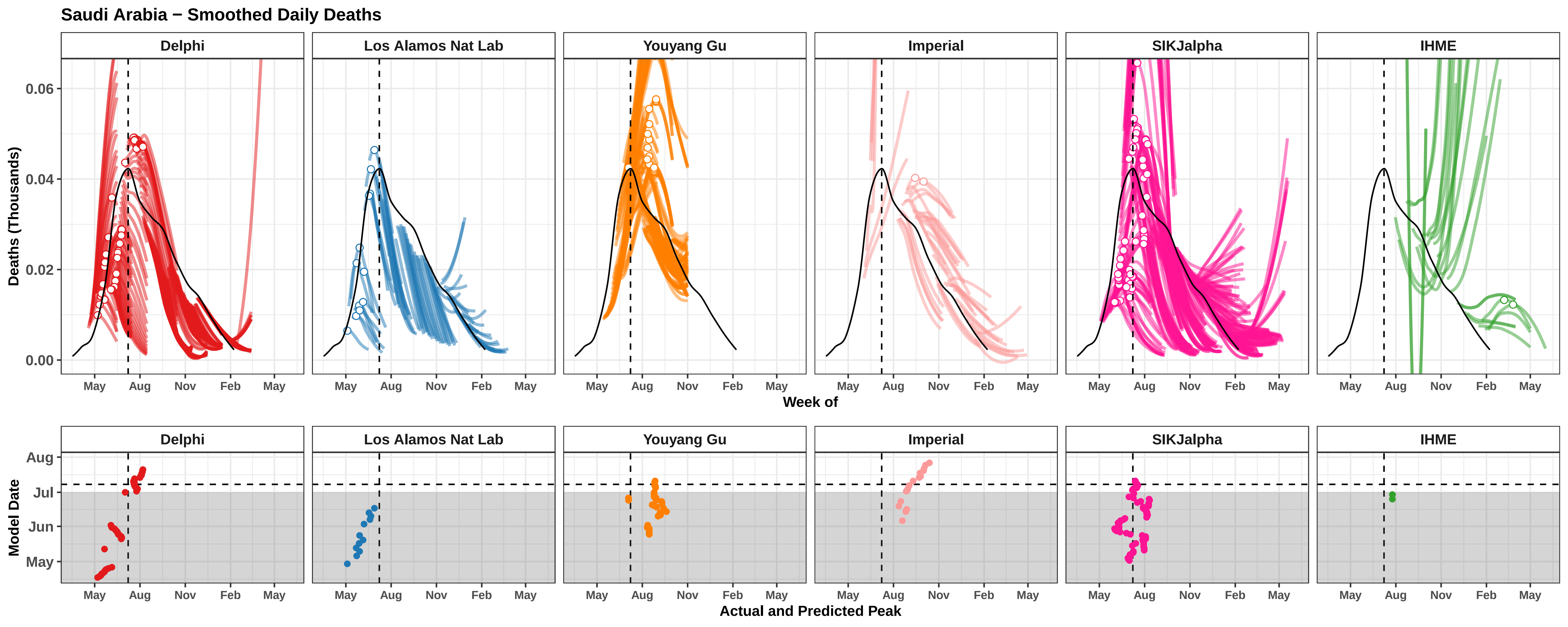
Saudi Arabia − Smoothed Daily Deaths
Delphi
Los Alamos Nat Lab
Youyang Gu
Imperial
SIKJalpha
IHME
Deaths (Thousands)
0.06
0.04
0.02
0.00
May
Aug
Nov
Feb
May
Week of
Model Date
Aug
Jul
Jun
May
Actual and Predicted Peak

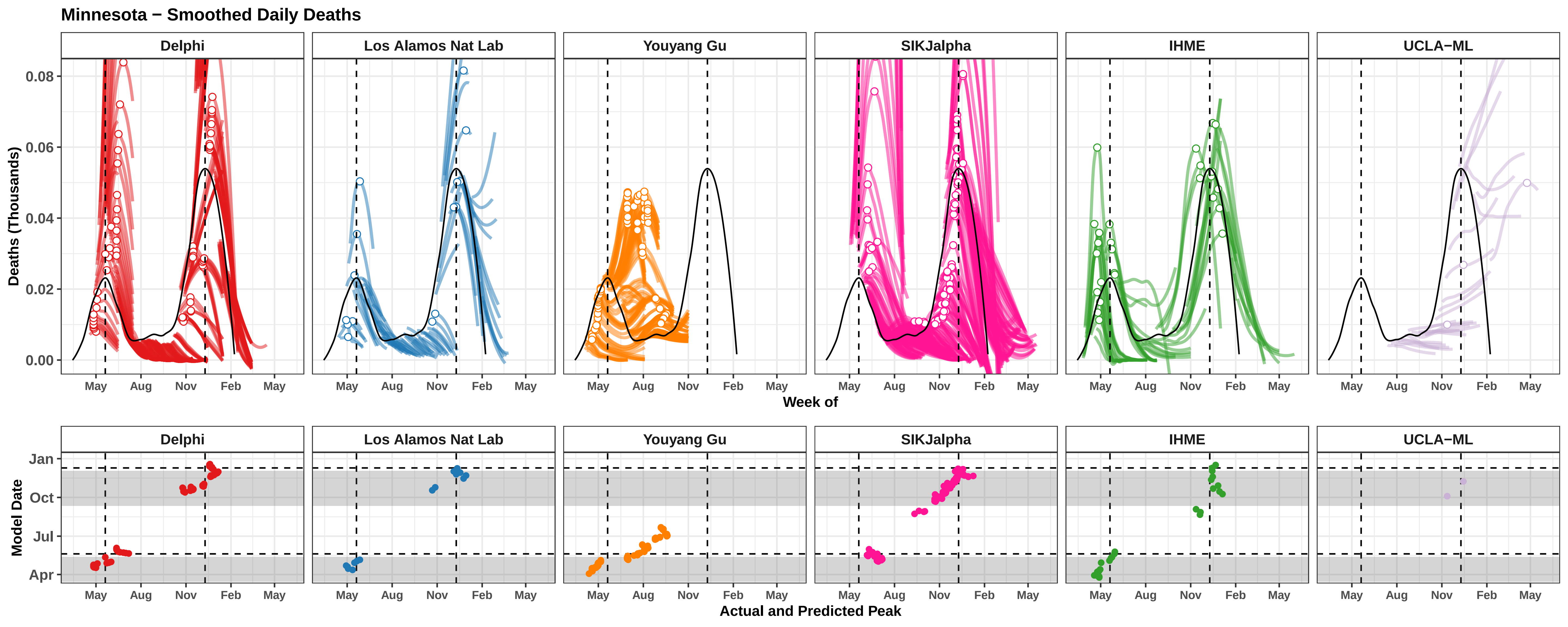
Minnesota − Smoothed Daily Deaths
Delphi
Los Alamos Nat Lab
Youyang Gu
SIKJalpha
IHME
UCLA−ML
Deaths (Thousands)
0.08
0.06
0.04
0.02
0.00
May
Aug
Nov
Feb
May
Week of
Model Date
Jan
Oct
Jul
Apr
Actual and Predicted Peak

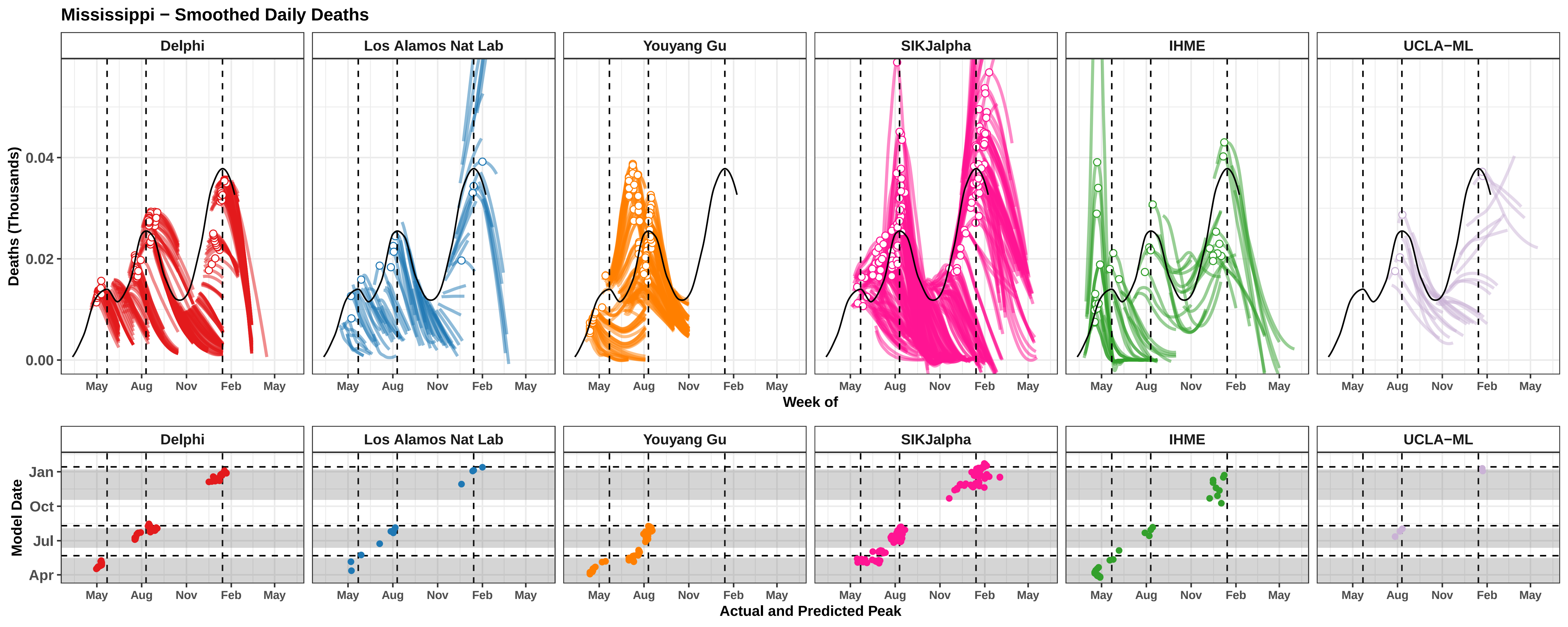

Mississippi − Smoothed Daily Deaths
Delphi
Los Alamos Nat Lab
Youyang Gu
SIKJalpha
IHME
UCLA−ML
Deaths (Thousands)
0.00
0.02
0.04
May
Aug
Nov
Feb
Week of
Model Date
Apr
Jul
Oct
Jan
Actual and Predicted Peak

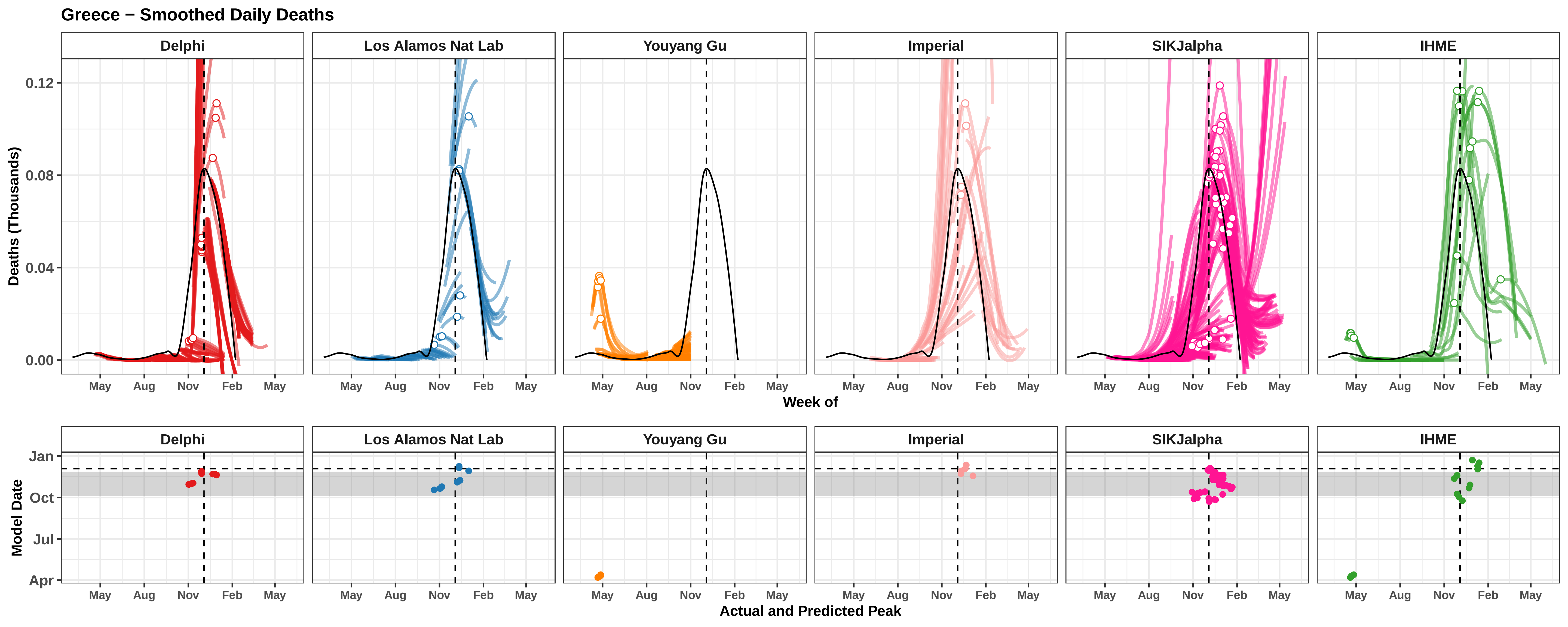

Greece − Smoothed Daily Deaths
Delphi
Los Alamos Nat Lab
Youyang Gu
Imperial
SIKJalpha
IHME
Deaths (Thousands)
0.12
0.08
0.04
0.00
May
Aug
Nov
Feb
May
Week of
Model Date
Jan
Oct
Jul
Apr
Actual and Predicted Peak

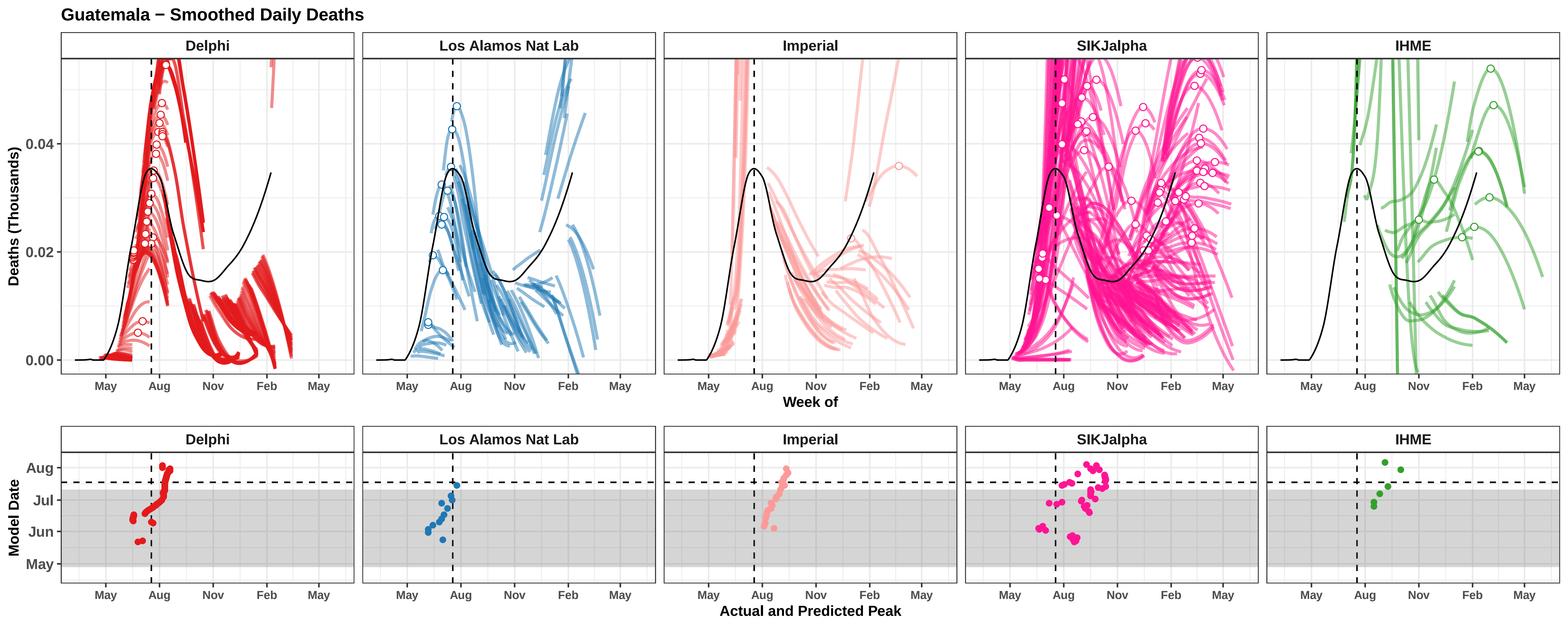

Guatemala − Smoothed Daily Deaths
Delphi
Los Alamos Nat Lab
Imperial
SIKJalpha
IHME
Deaths (Thousands)
0.00
0.02
0.04
May
Aug
Nov
Feb
May
Week of
Model Date
Aug
Jul
Jun
May
Actual and Predicted Peak

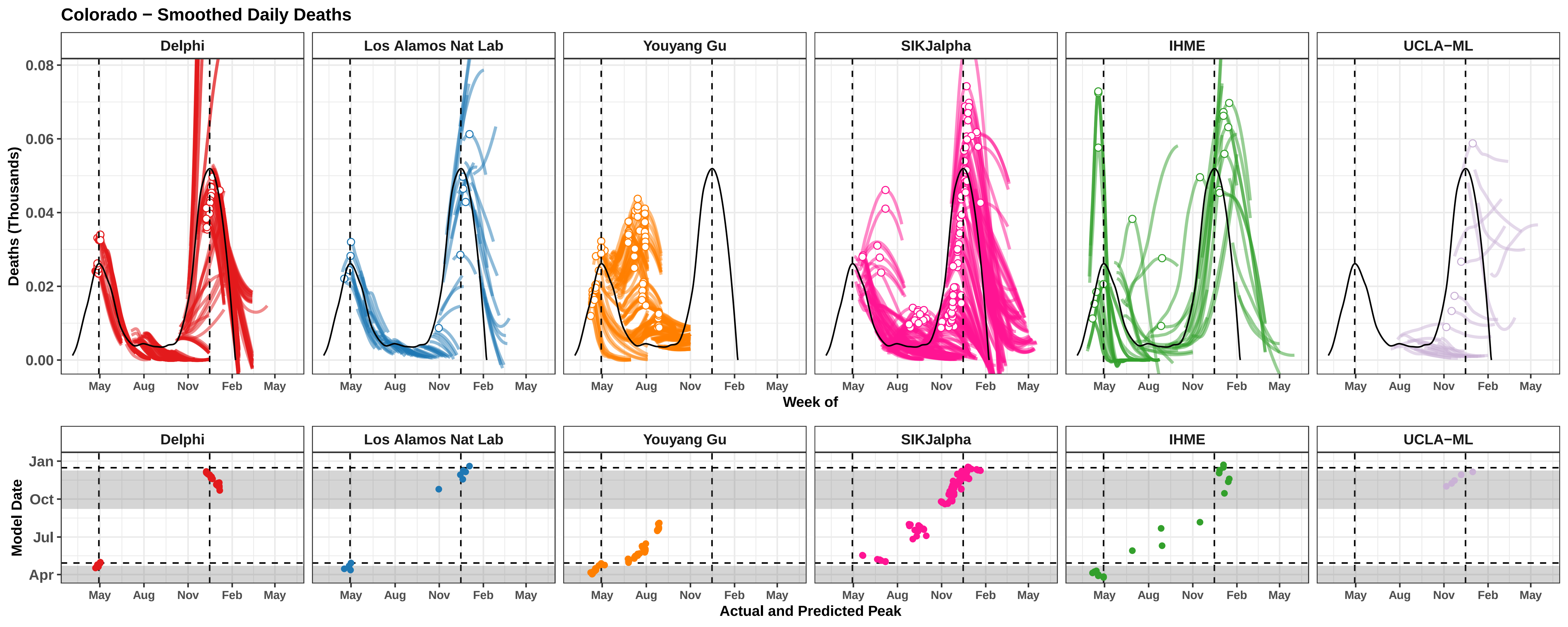

Colorado − Smoothed Daily Deaths
Delphi
Los Alamos Nat Lab
Youyang Gu
SIKJalpha
IHME
UCLA−ML
Deaths (Thousands)
0.00
0.02
0.04
0.06
0.08
May
Aug
Nov
Feb
May
Week of
Model Date
Jan
Oct
Jul
Apr
Actual and Predicted Peak

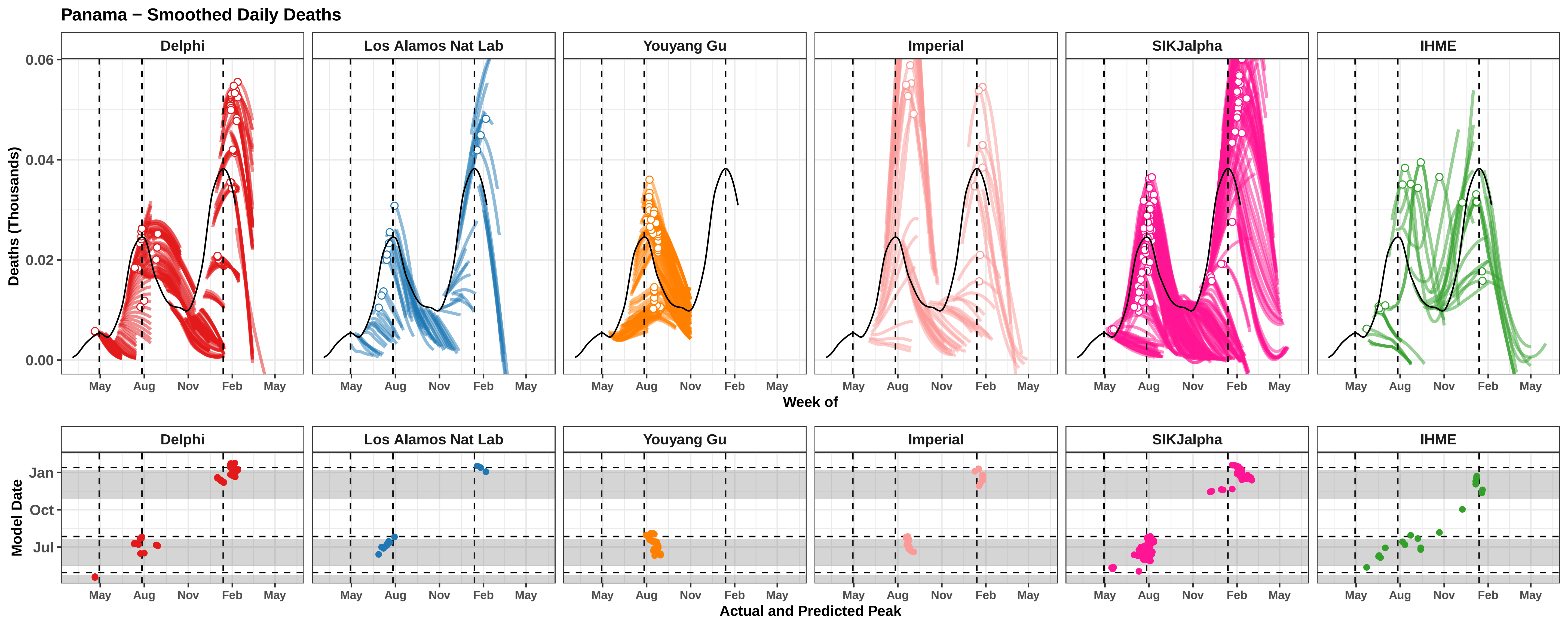

Panama − Smoothed Daily Deaths
Delphi
Los Alamos Nat Lab
Youyang Gu
Imperial
SIKJalpha
IHME
Deaths (Thousands)
0.06
0.04
0.02
0.00
May
Aug
Nov
Feb
May
Week of
Model Date
Jan
Oct
Jul
Actual and Predicted Peak

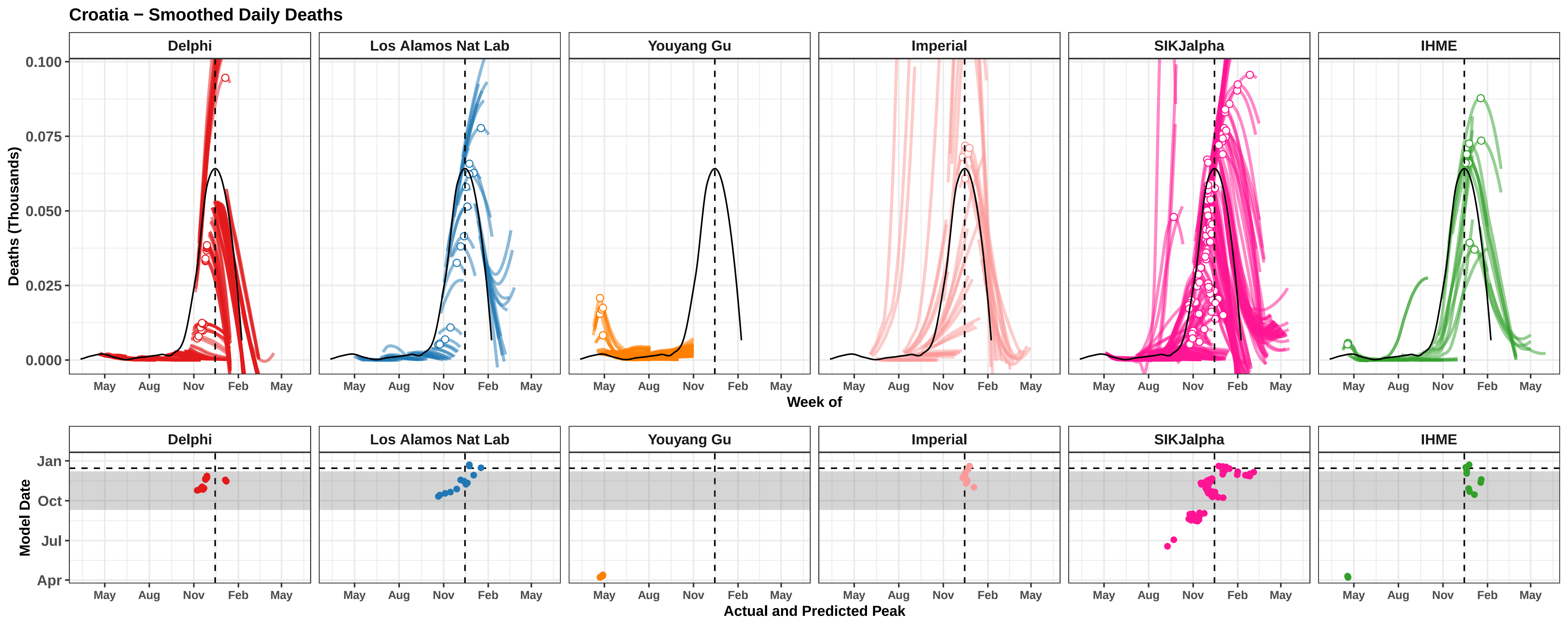
Croatia − Smoothed Daily Deaths
Delphi
Los Alamos Nat Lab
Youyang Gu
Imperial
SIKJalpha
IHME
Deaths (Thousands)
0.100
0.075
0.050
0.025
0.000
May
Aug
Nov
Feb
Week of
Model Date
Jan
Oct
Jul
Apr
Actual and Predicted Peak

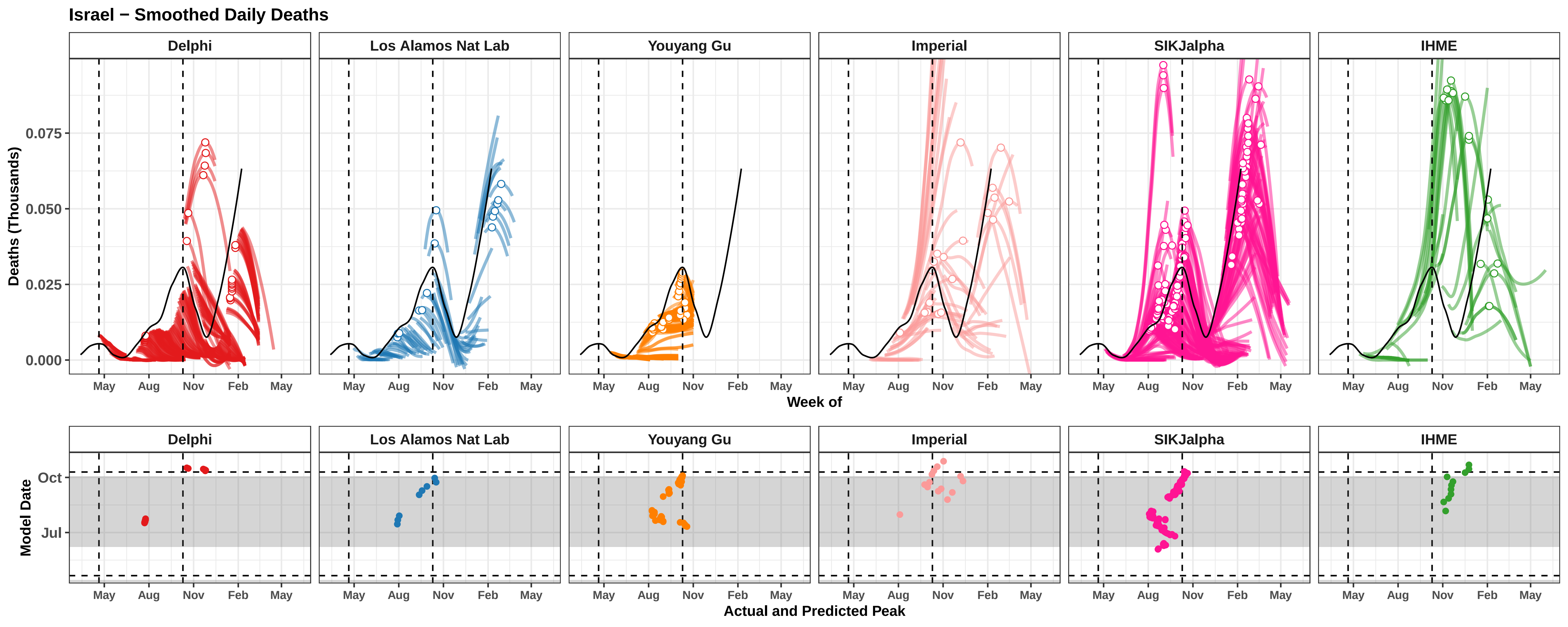
Israel − Smoothed Daily Deaths
Delphi
Los Alamos Nat Lab
Youyang Gu
Imperial
SIKJalpha
IHME
Deaths (Thousands)
0.000
0.025
0.050
0.075
May
Aug
Nov
Feb
May
Week of
Model Date
Oct
Jul
Actual and Predicted Peak

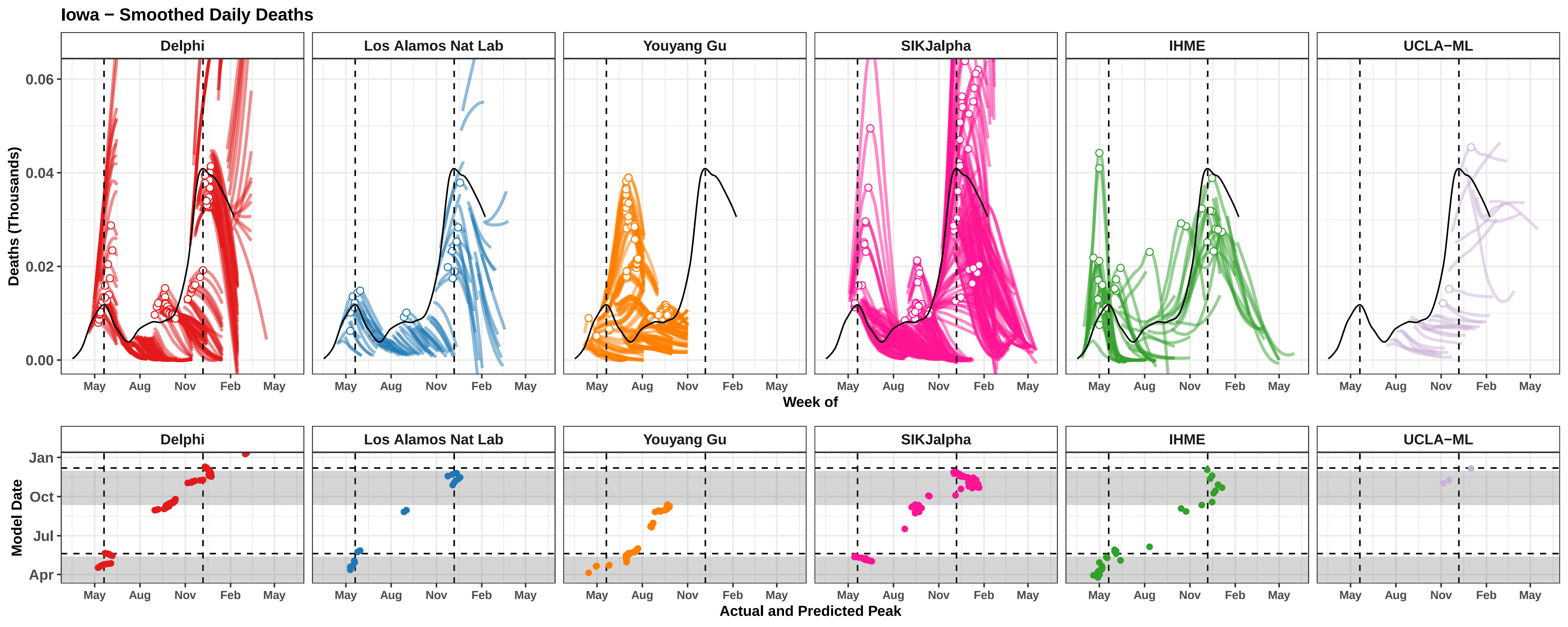

Iowa − Smoothed Daily Deaths
Delphi
Los Alamos Nat Lab
Youyang Gu
SIKJalpha
IHME
UCLA−ML
Deaths (Thousands)
0.00
0.02
0.04
0.06
May
Aug
Nov
Feb
May
Week of
Model Date
Jan
Oct
Jul
Apr
Actual and Predicted Peak

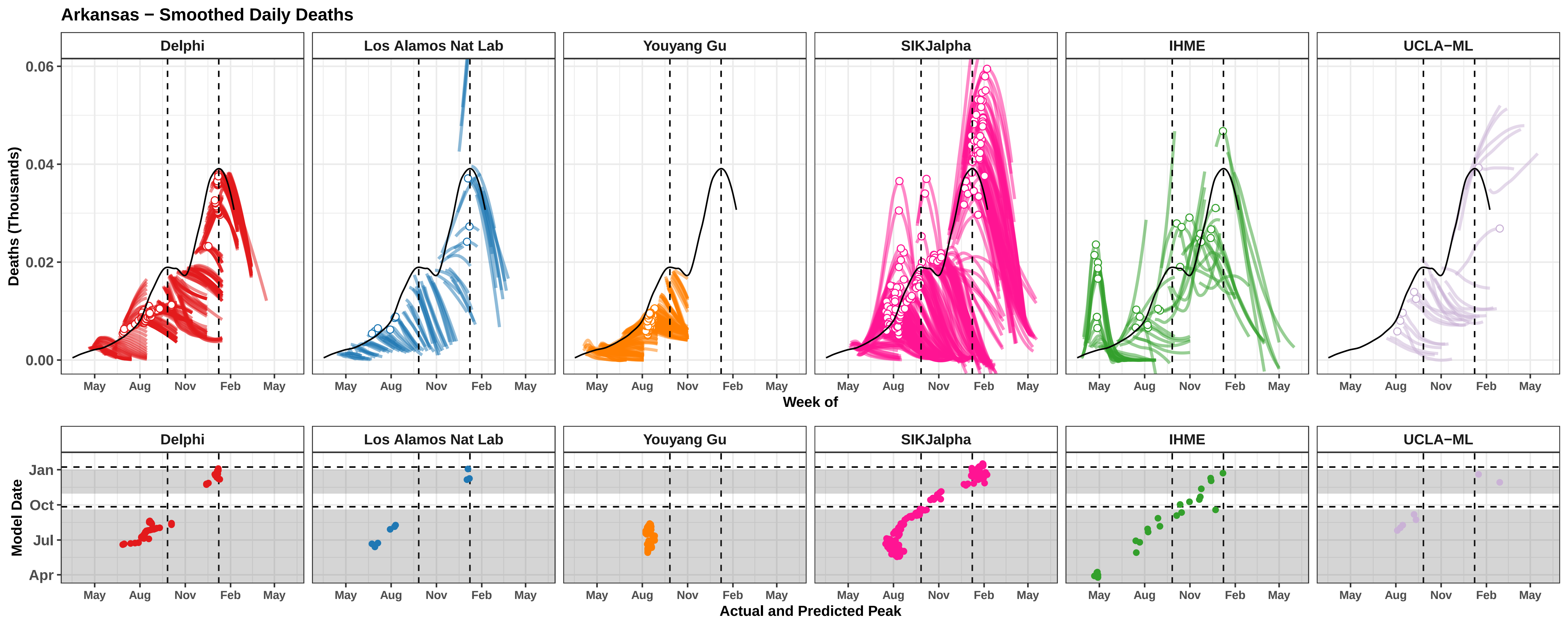

Arkansas − Smoothed Daily Deaths
Delphi
Los Alamos Nat Lab
Youyang Gu
SIKJalpha
IHME
UCLA−ML
Deaths (Thousands)
0.00
0.02
0.04
0.06
May
Aug
Nov
Feb
May
Week of
Model Date
Apr
Jul
Oct
Jan
Actual and Predicted Peak

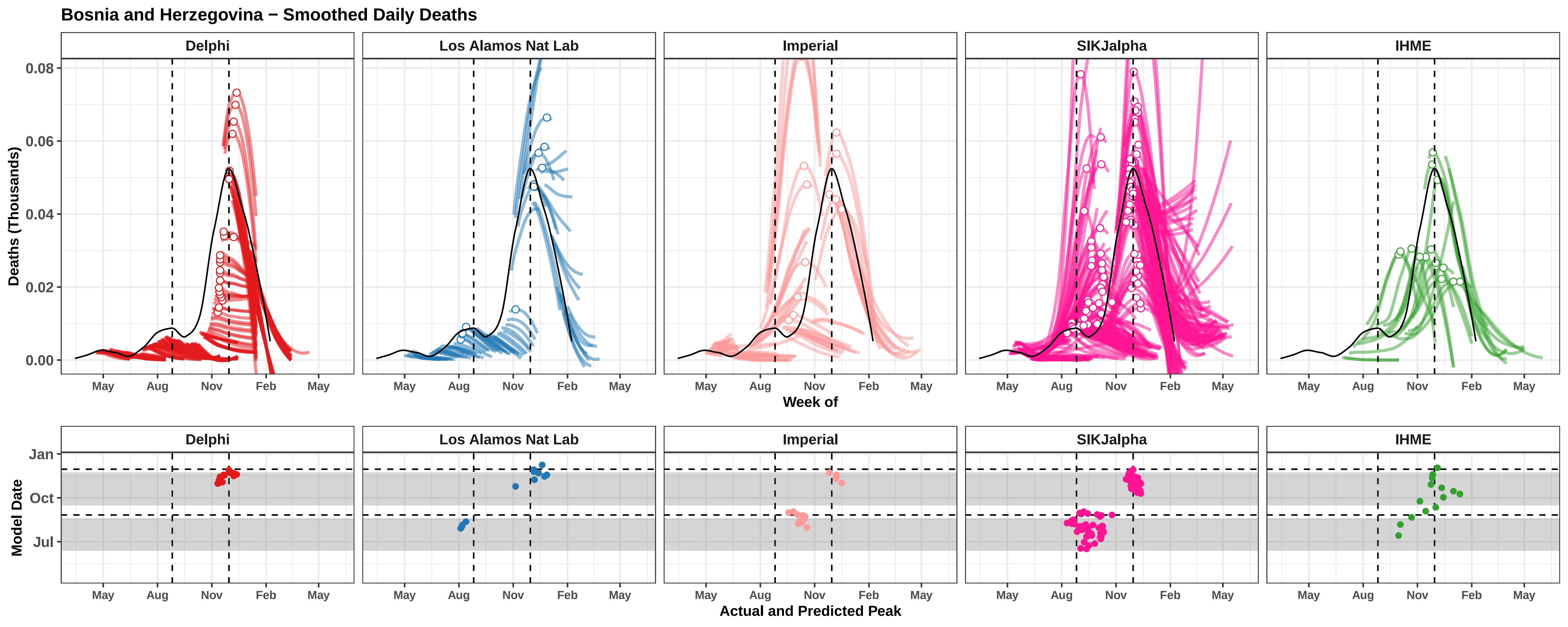
Bosnia and Herzegovina − Smoothed Daily Deaths
Delphi
Los Alamos Nat Lab
Imperial
SIKJalpha
IHME
Deaths (Thousands)
0.08
0.06
0.04
0.02
0.00
May
Aug
Nov
Feb
May
Week of
Model Date
Jan
Oct
Jul
Actual and Predicted Peak

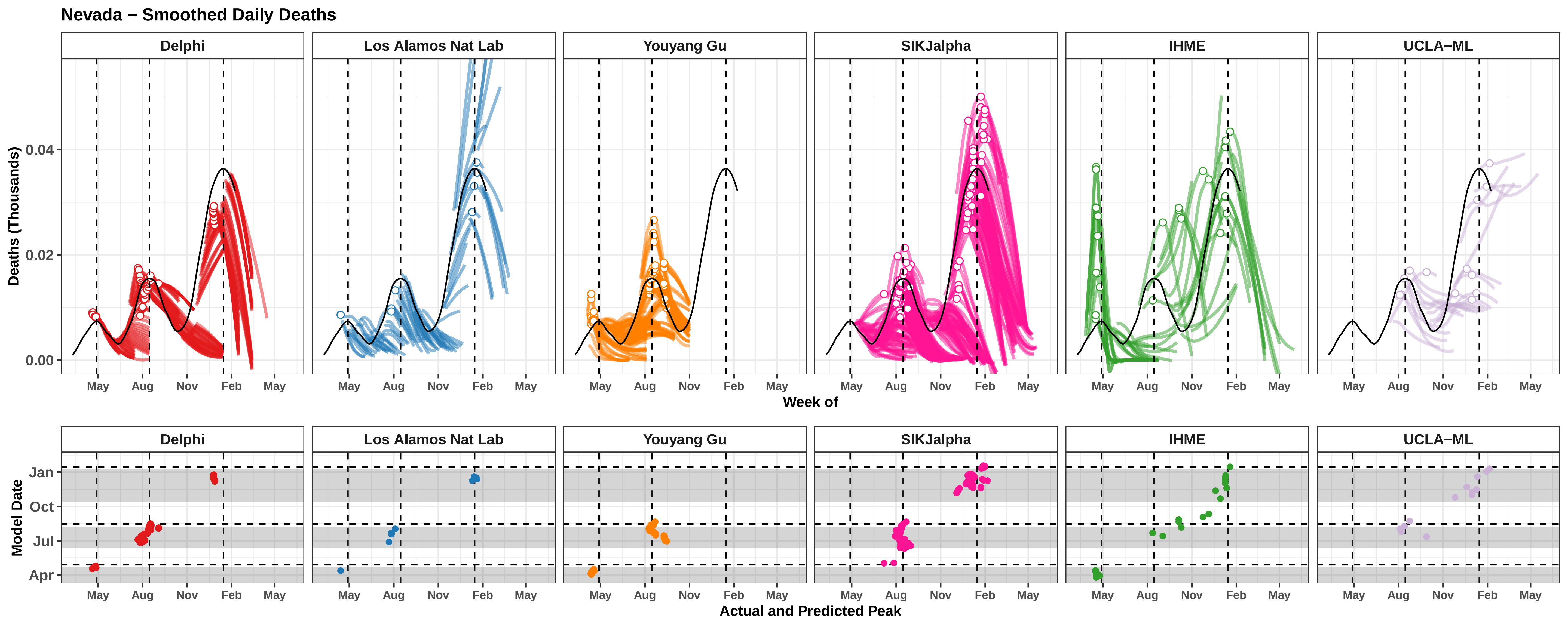

Nevada − Smoothed Daily Deaths
Delphi
Los Alamos Nat Lab
Youyang Gu
SIKJalpha
IHME
UCLA−ML
Deaths (Thousands)
0.00
0.02
0.04
May
Aug
Nov
Feb
May
Week of
Model Date
Apr
Jul
Oct
Jan
Actual and Predicted Peak

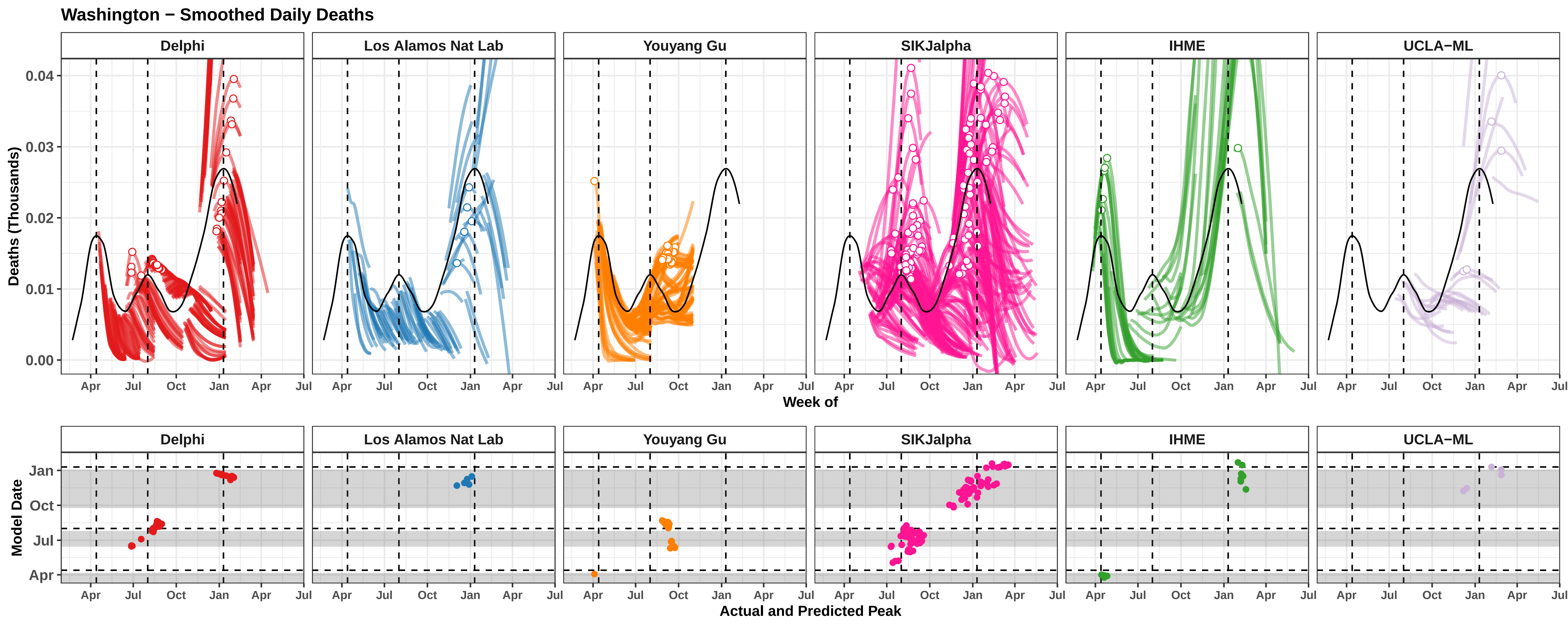

Washington − Smoothed Daily Deaths
Delphi
Los Alamos Nat Lab
Youyang Gu
SIKJalpha
IHME
UCLA−ML
Deaths (Thousands)
0.00
0.01
0.02
0.03
0.04
Apr
Jul
Oct
Jan
Week of
Model Date
Actual and Predicted Peak

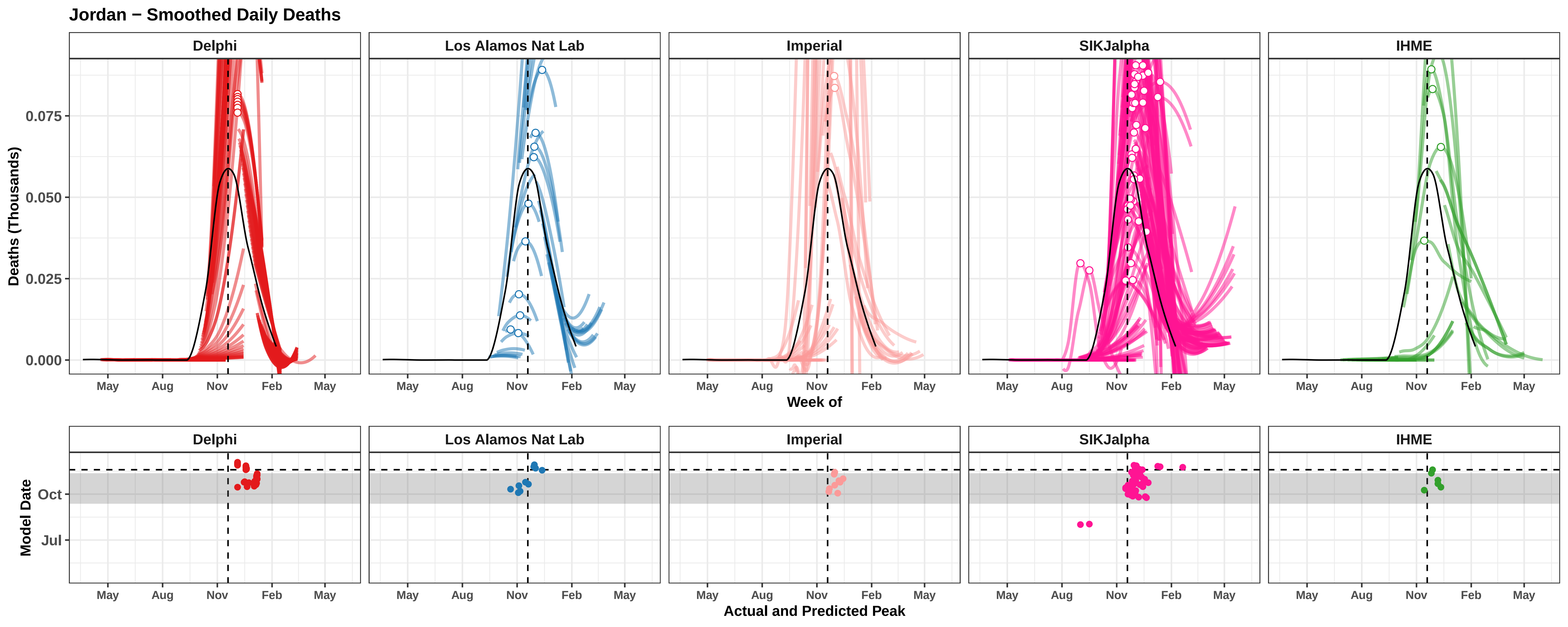

Jordan − Smoothed Daily Deaths
Delphi
Los Alamos Nat Lab
Imperial
SIKJalpha
IHME
Deaths (Thousands)
0.075
0.050
0.025
0.000
May
Aug
Nov
Feb
May
Week of
Model Date
Oct
Jul
Actual and Predicted Peak

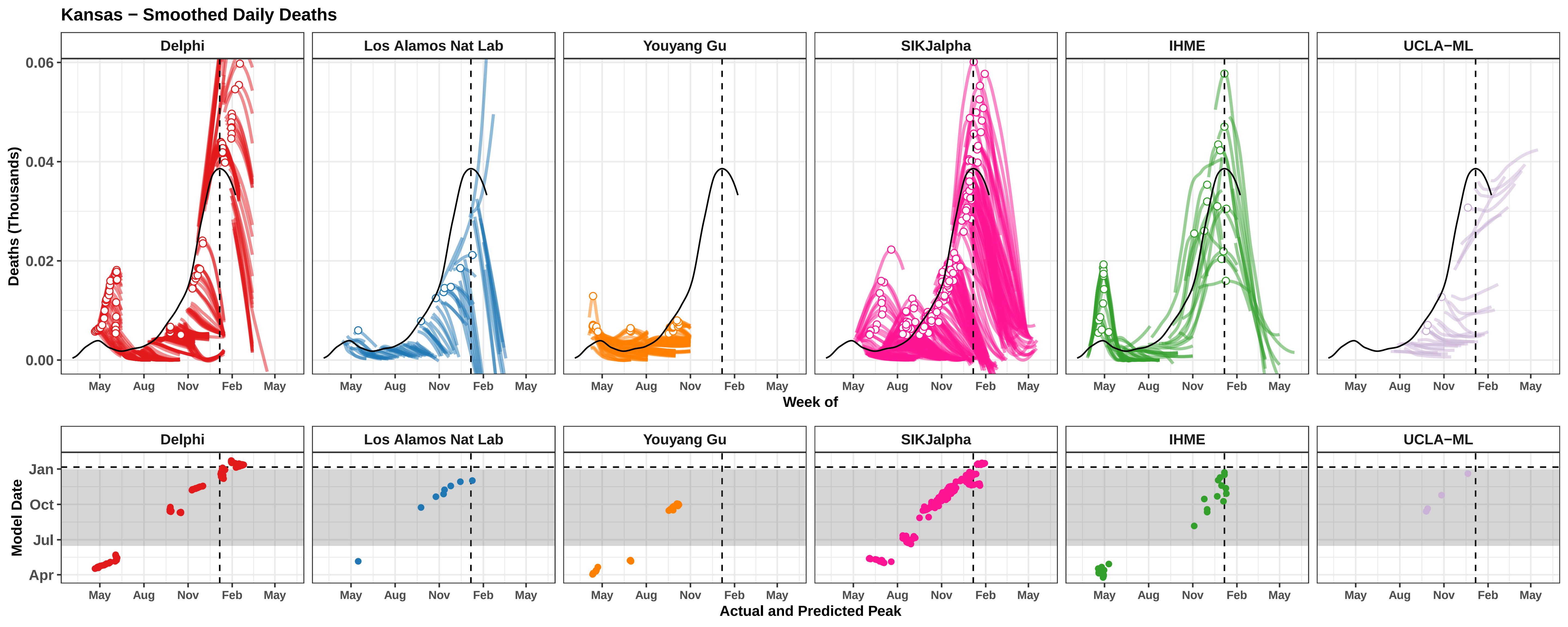

Kansas − Smoothed Daily Deaths
Delphi
Los Alamos Nat Lab
Youyang Gu
SIKJalpha
IHME
UCLA−ML
Deaths (Thousands)
0.06
0.04
0.02
0.00
May
Aug
Nov
Feb
May
Week of
Model Date
Jan
Oct
Jul
Apr
Actual and Predicted Peak

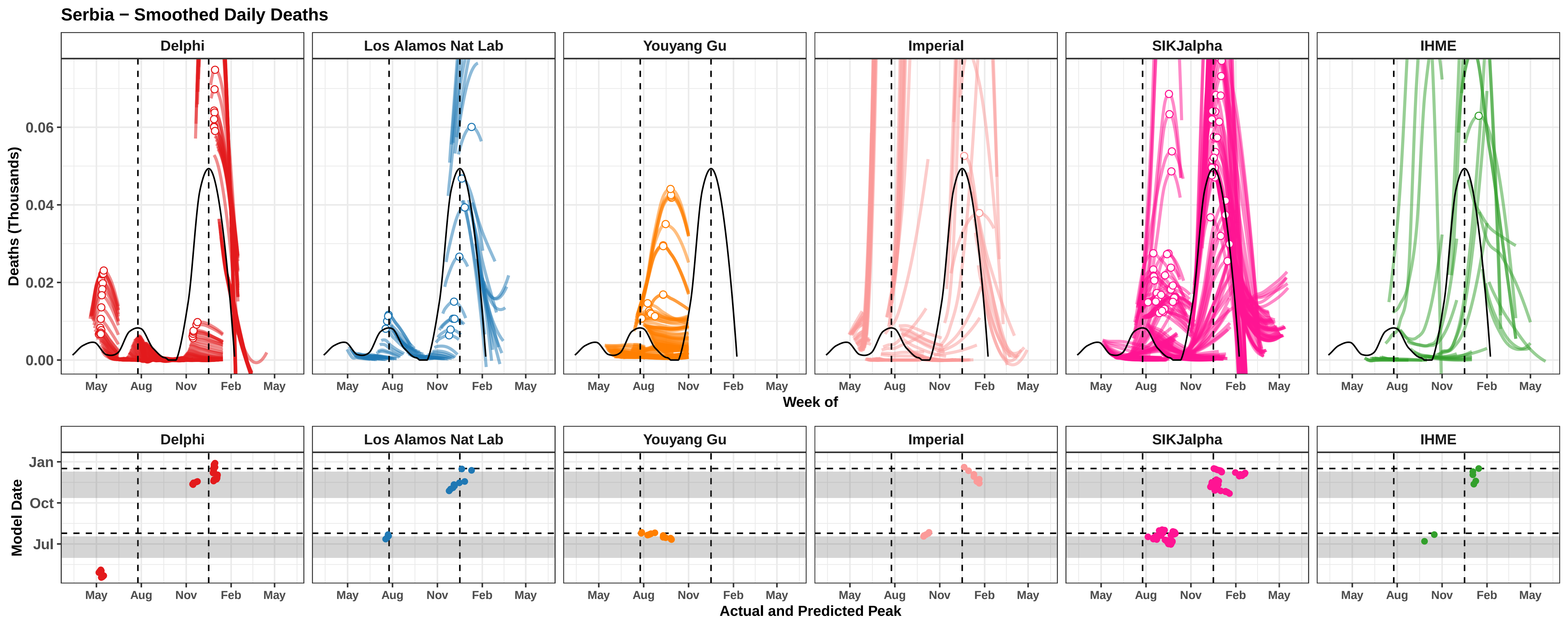

Serbia − Smoothed Daily Deaths
Delphi
Los Alamos Nat Lab
Youyang Gu
Imperial
SIKJalpha
IHME
Deaths (Thousands)
0.00
0.02
0.04
0.06
May
Aug
Nov
Feb
May
Week of
Model Date
Jan
Oct
Jul
Actual and Predicted Peak

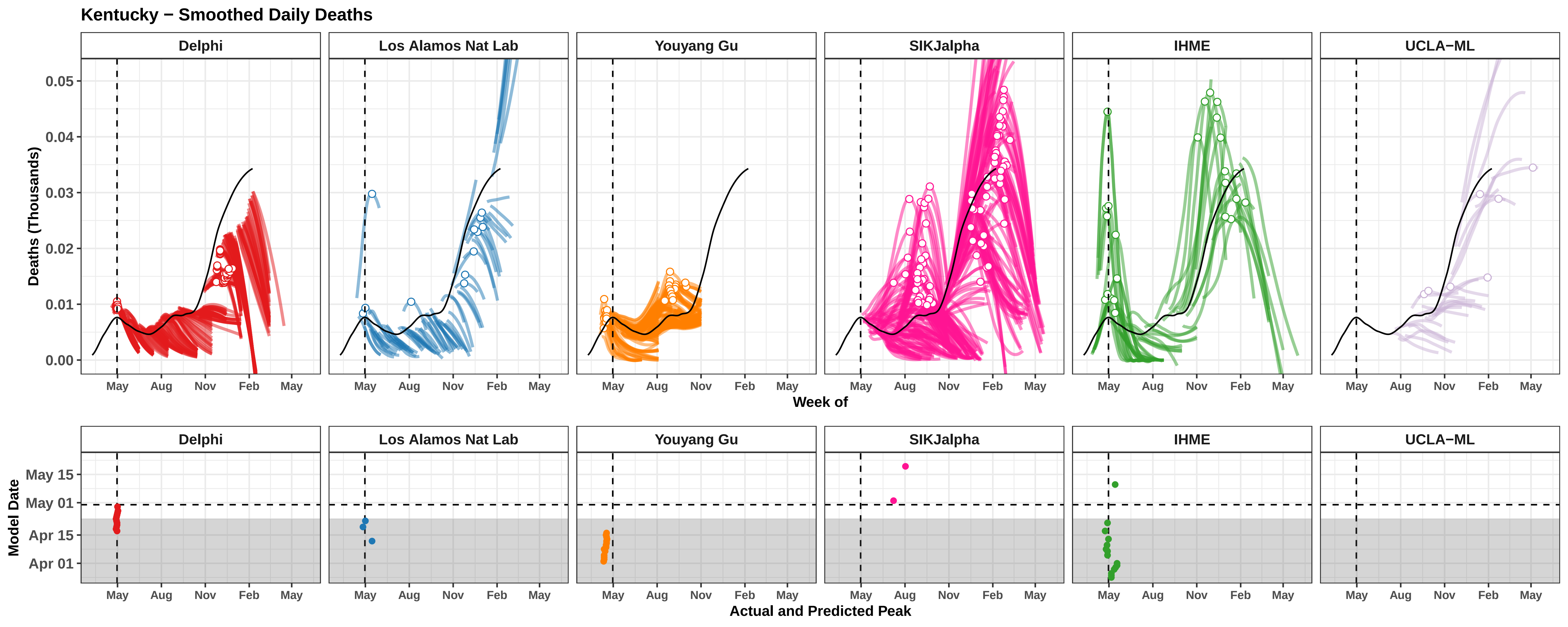

Kentucky − Smoothed Daily Deaths
Delphi
Los Alamos Nat Lab
Youyang Gu
SIKJalpha
IHME
UCLA−ML
Deaths (Thousands)
0.05
0.04
0.03
0.02
0.01
0.00
May
Aug
Nov
Feb
May
Week of
Model Date
May 15
May 01
Apr 15
Apr 01
Actual and Predicted Peak

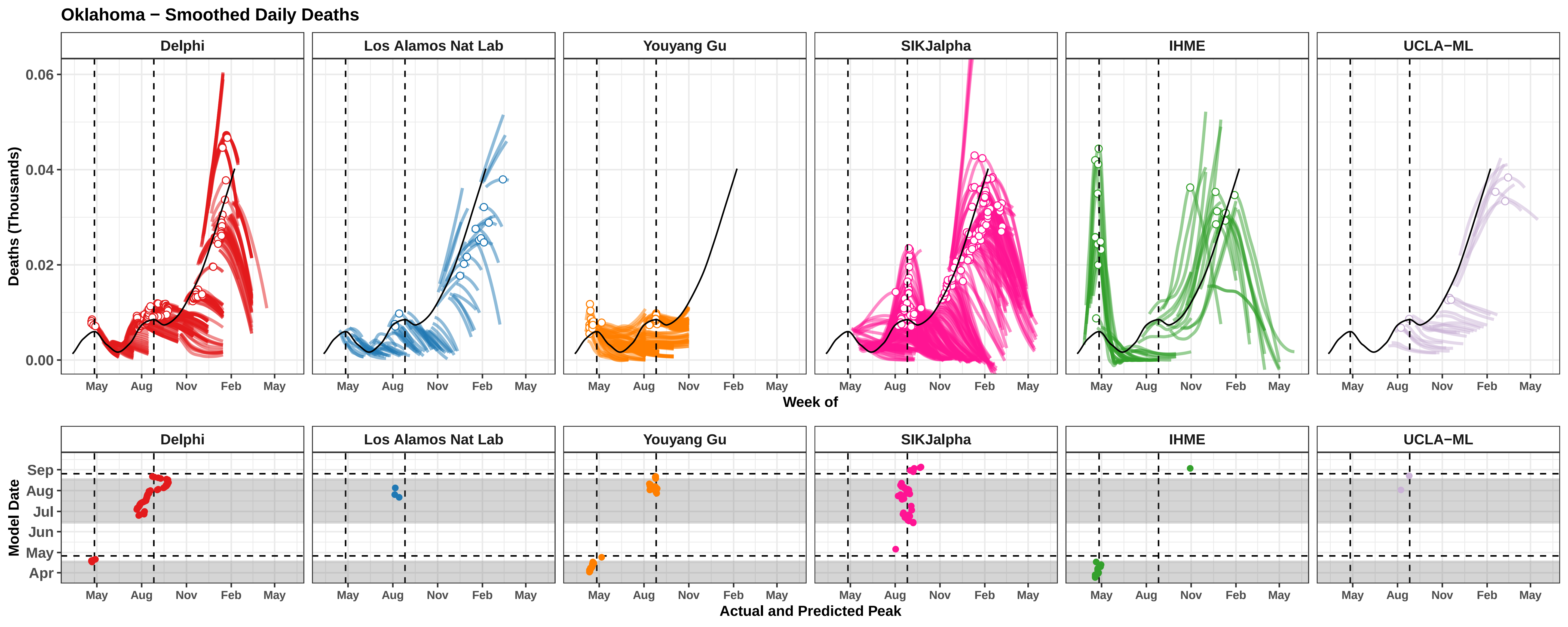

Oklahoma − Smoothed Daily Deaths
Delphi
Los Alamos Nat Lab
Youyang Gu
SIKJalpha
IHME
UCLA−ML
Deaths (Thousands)
0.06
0.04
0.02
0.00
May
Aug
Nov
Feb
May
Week of
Model Date
Sep
Aug
Jul
Jun
May
Apr
Actual and Predicted Peak

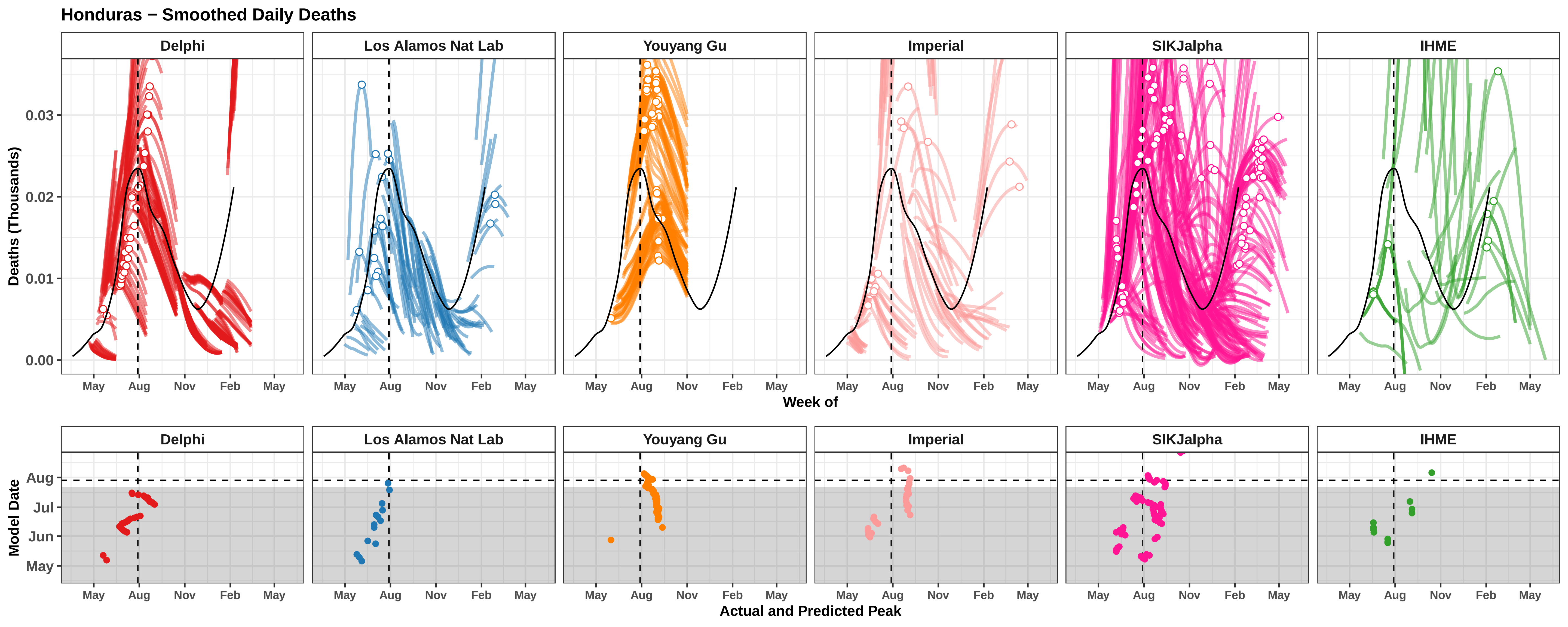

Honduras − Smoothed Daily Deaths
Delphi
Los Alamos Nat Lab
Youyang Gu
Imperial
SIKJalpha
IHME
Deaths (Thousands)
0.00
0.01
0.02
0.03
May
Aug
Nov
Feb
May
Week of
Model Date
May
Jun
Jul
Aug
Actual and Predicted Peak

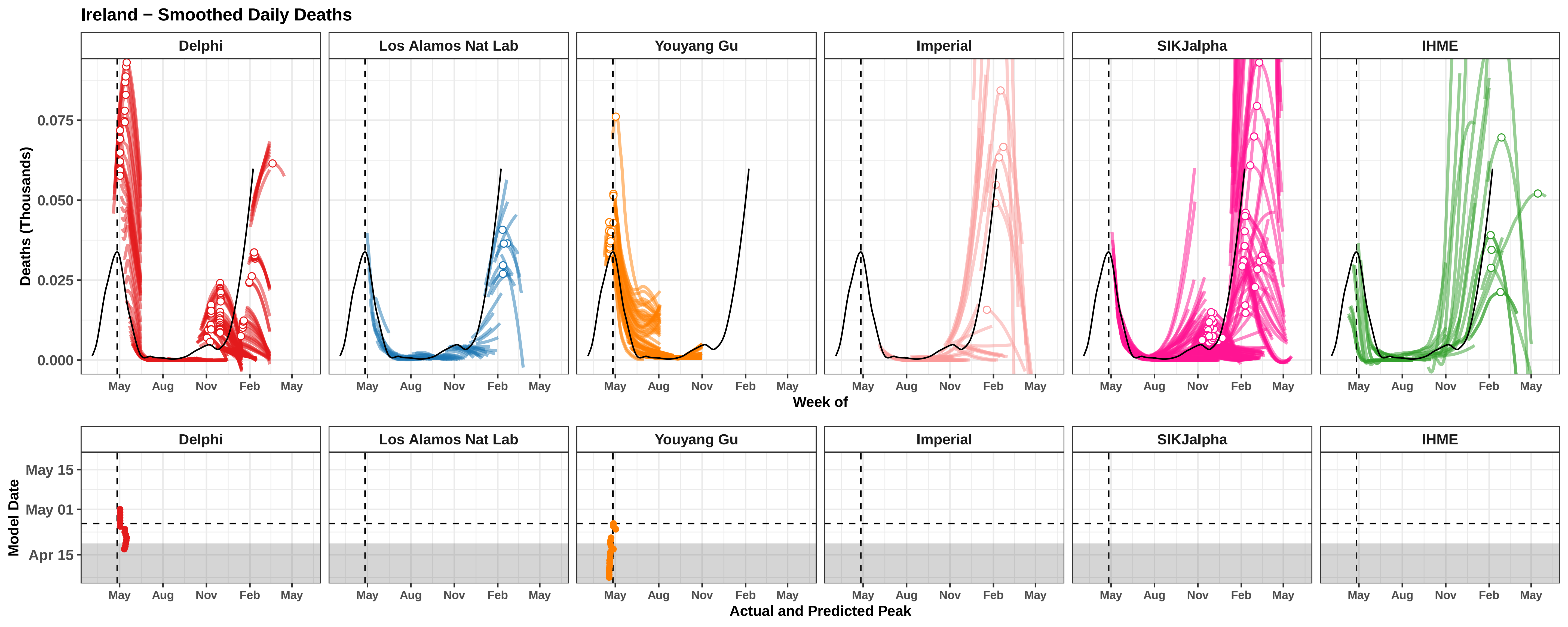

Ireland − Smoothed Daily Deaths
Delphi
Los Alamos Nat Lab
Youyang Gu
Imperial
SIKJalpha
IHME
Deaths (Thousands)
0.075
0.050
0.025
0.000
May
Aug
Nov
Feb
Week of
Model Date
May 15
May 01
Apr 15
Actual and Predicted Peak

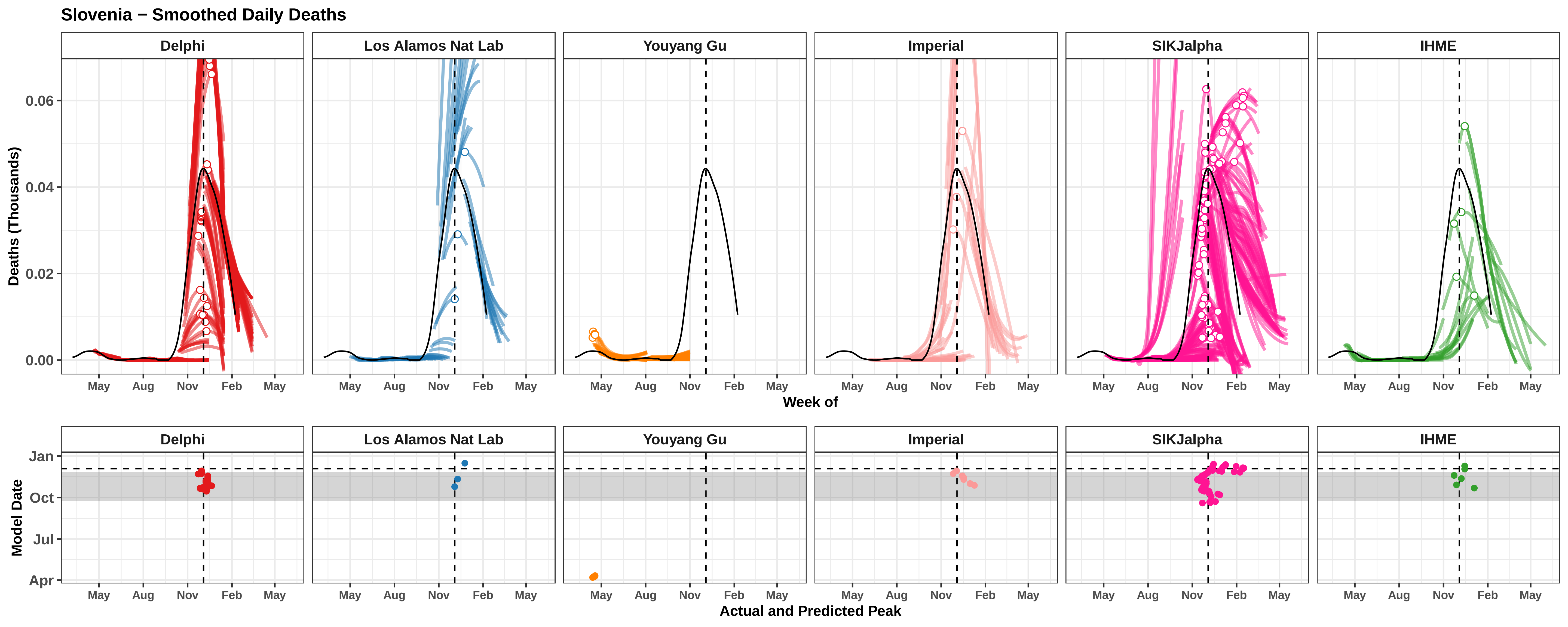

Slovenia − Smoothed Daily Deaths
Delphi
Los Alamos Nat Lab
Youyang Gu
Imperial
SIKJalpha
IHME
Deaths (Thousands)
0.06
0.04
0.02
0.00
May
Aug
Nov
Feb
May
Week of
Model Date
Jan
Oct
Jul
Apr
Actual and Predicted Peak

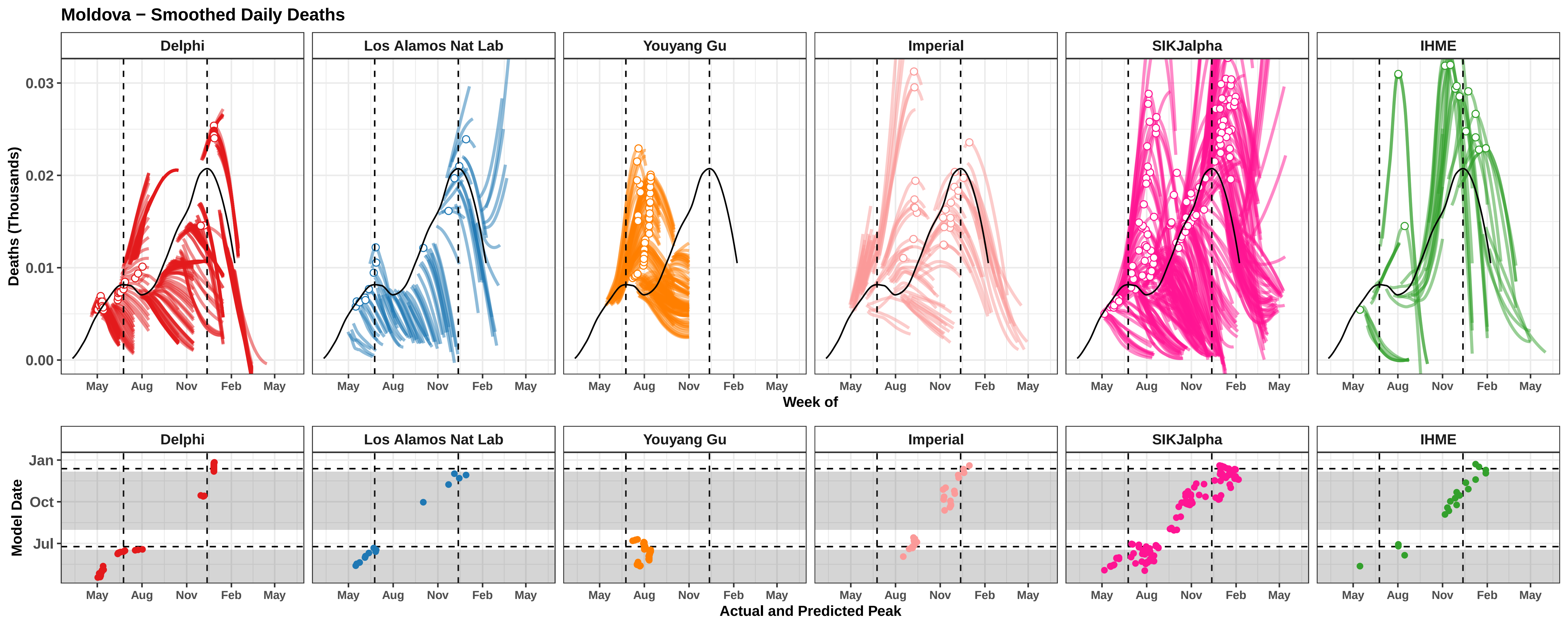

Moldova − Smoothed Daily Deaths
Delphi
Los Alamos Nat Lab
Youyang Gu
Imperial
SIKJalpha
IHME
Deaths (Thousands)
0.00
0.01
0.02
0.03
May
Aug
Nov
Feb
May
Week of
Model Date
Jan
Oct
Jul
Actual and Predicted Peak

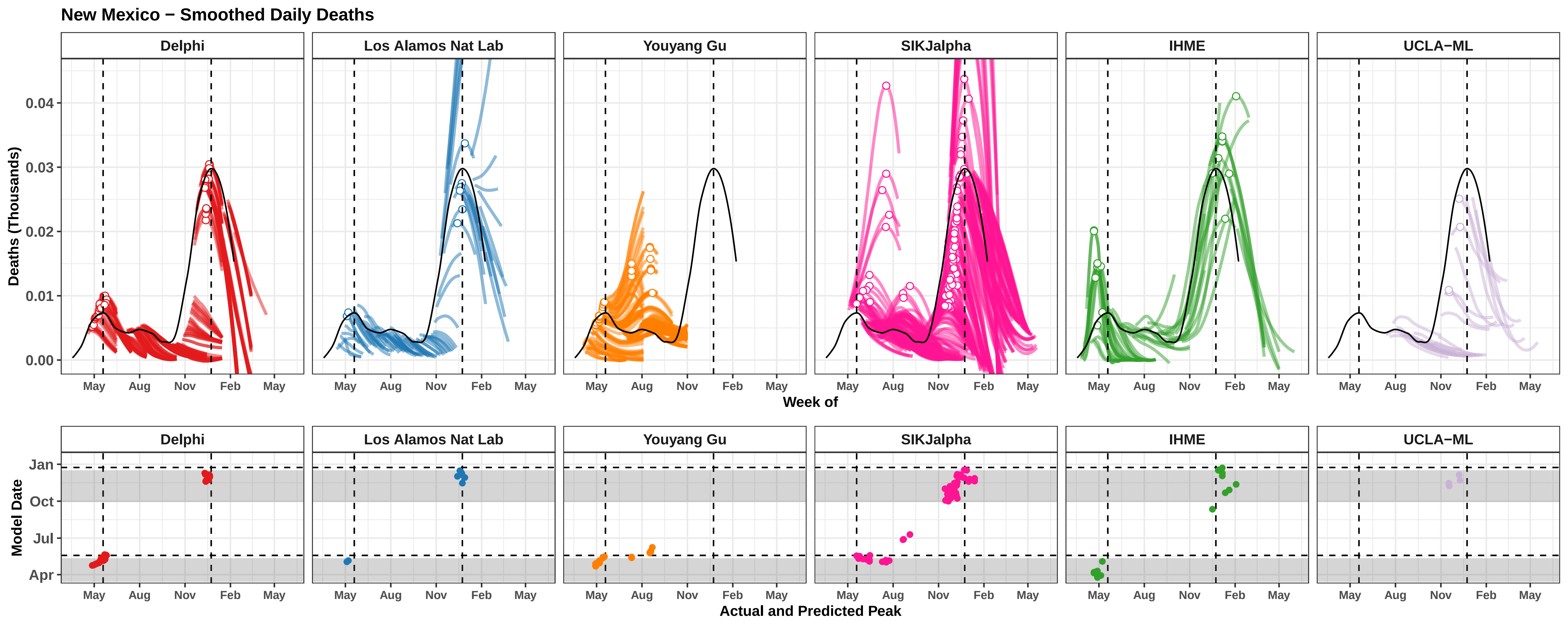

New Mexico − Smoothed Daily Deaths
Delphi
Los Alamos Nat Lab
Youyang Gu
SIKJalpha
IHME
UCLA−ML
Deaths (Thousands)
0.00
0.01
0.02
0.03
0.04
May
Aug
Nov
Feb
May
Week of
Model Date
Jan
Oct
Jul
Apr
Actual and Predicted Peak

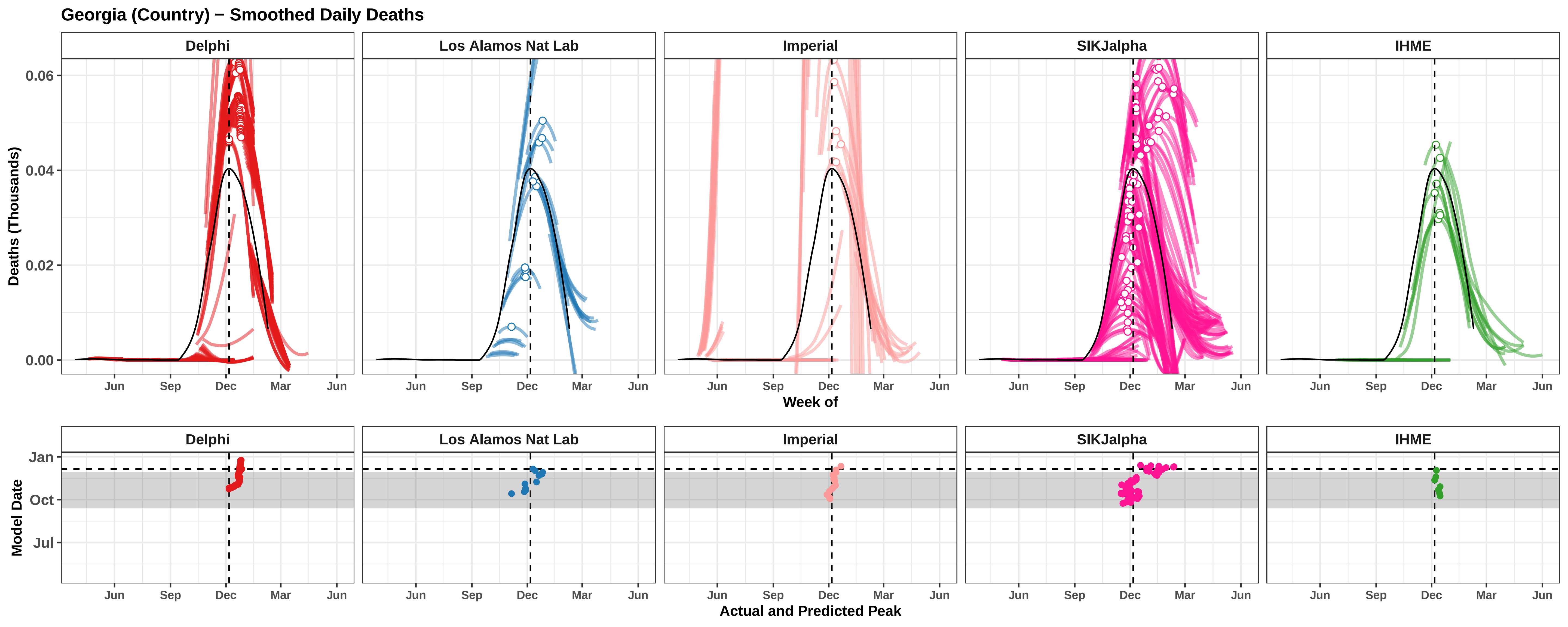

Georgia (Country) − Smoothed Daily Deaths
Delphi
Los Alamos Nat Lab
Imperial
SIKJalpha
IHME
Deaths (Thousands)
0.06
0.04
0.02
0.00
Jun
Sep
Dec
Mar
Jun
Week of
Model Date
Jan
Oct
Jul
Actual and Predicted Peak

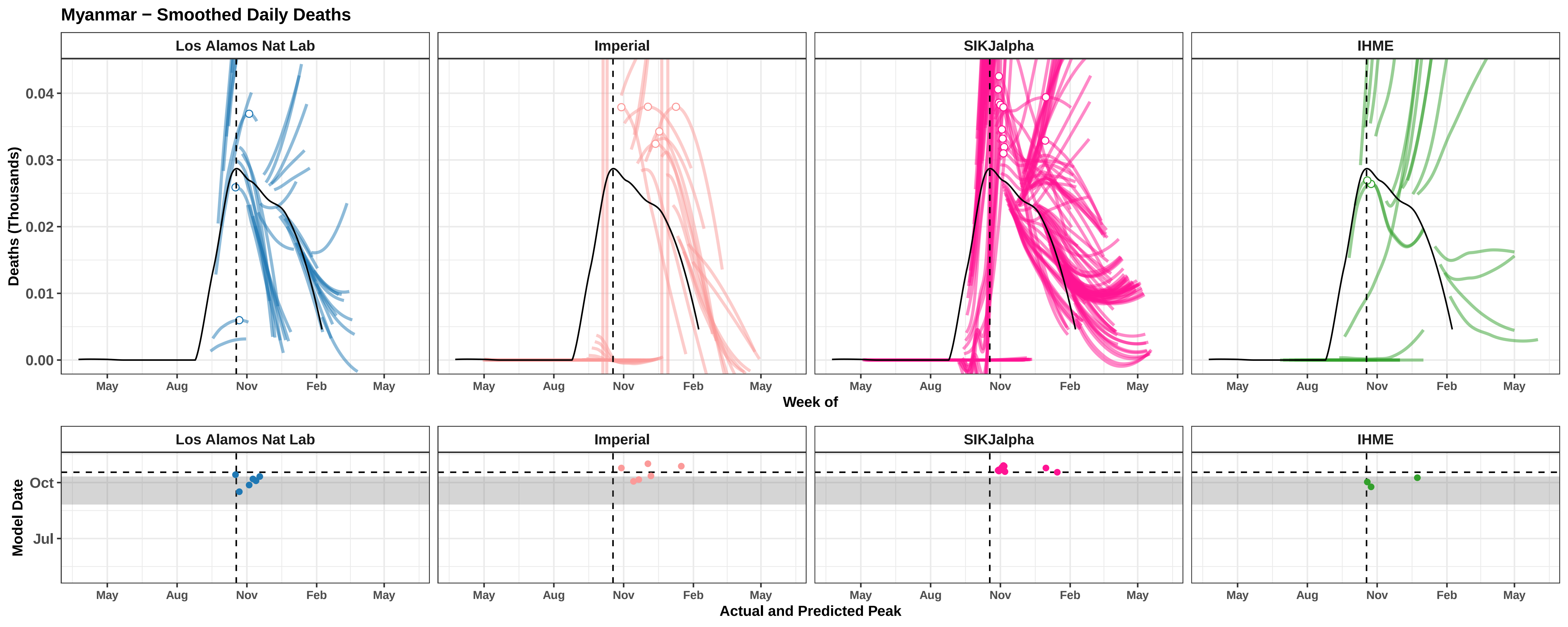
Myanmar − Smoothed Daily Deaths
Los Alamos Nat Lab
Imperial
SIKJalpha
IHME
0.04
0.03
0.02
0.01
0.00
Deaths (Thousands)
May
Aug
Nov
Feb
May
Week of
Oct
Jul
Model Date
Actual and Predicted Peak

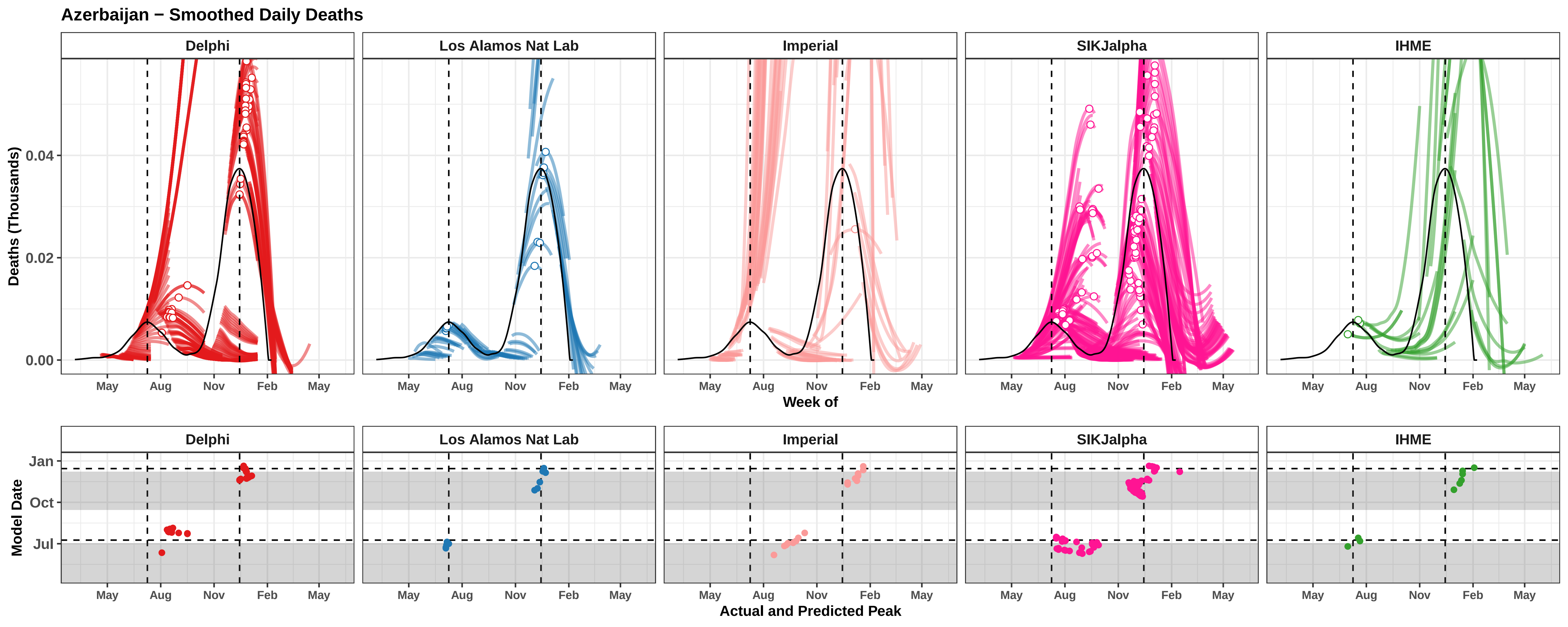

Azerbaijan − Smoothed Daily Deaths
Delphi
Los Alamos Nat Lab
Imperial
SIKJalpha
IHME
Deaths (Thousands)
0.00
0.02
0.04
May
Aug
Nov
Feb
May
Week of
Model Date
Jan
Oct
Jul
Actual and Predicted Peak

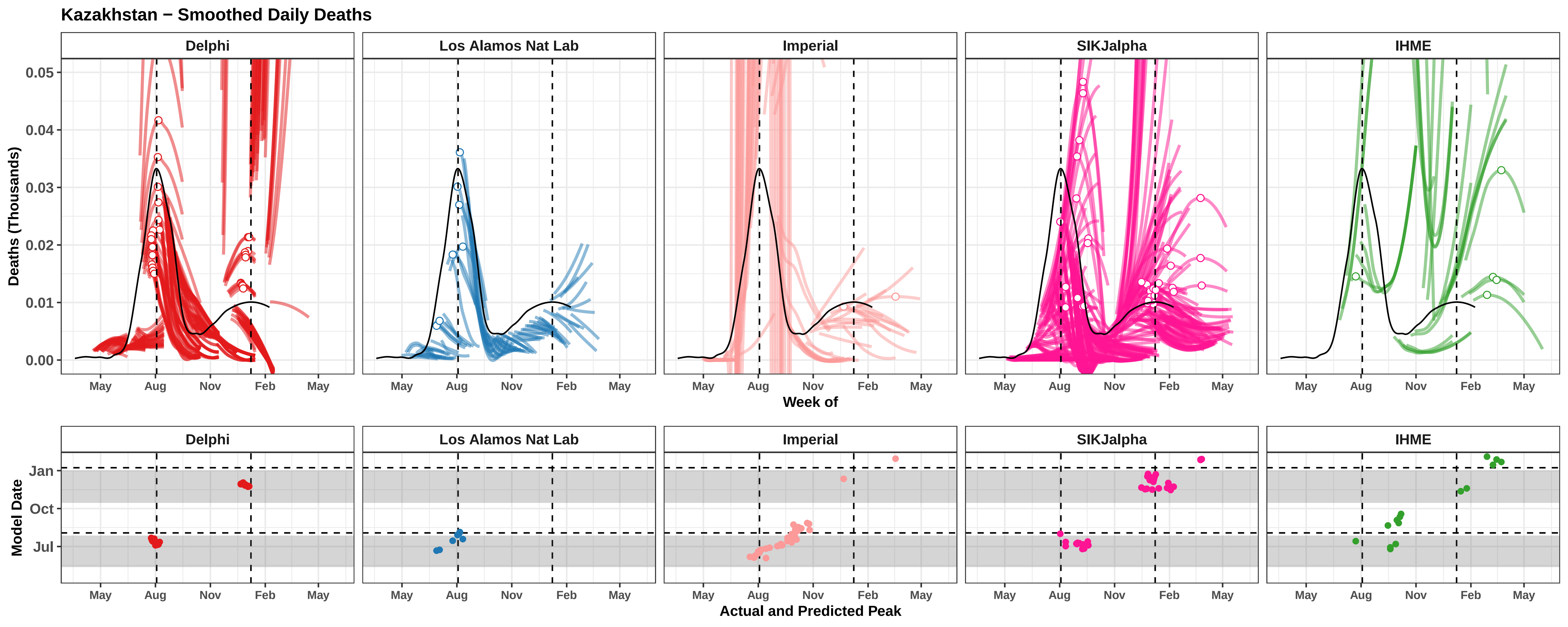
Kazakhstan − Smoothed Daily Deaths
Delphi
Los Alamos Nat Lab
Imperial
SIKJalpha
IHME
Deaths (Thousands)
0.00
0.01
0.02
0.03
0.04
0.05
May
Aug
Nov
Feb
May
Week of
Model Date
Jan
Oct
Jul
Actual and Predicted Peak

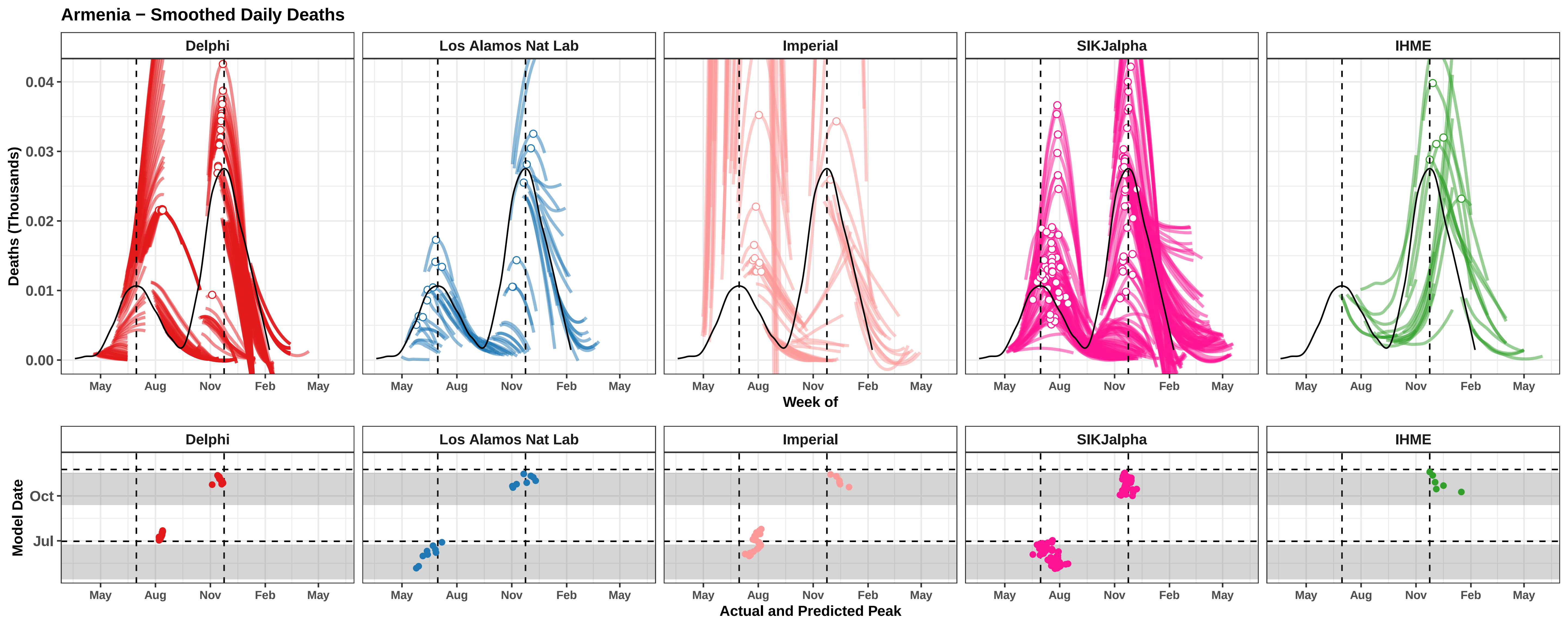

Armenia − Smoothed Daily Deaths
Delphi
Los Alamos Nat Lab
Imperial
SIKJalpha
IHME
Deaths (Thousands)
0.00
0.01
0.02
0.03
0.04
May
Aug
Nov
Feb
May
Week of
Model Date
Jul
Oct
Actual and Predicted Peak

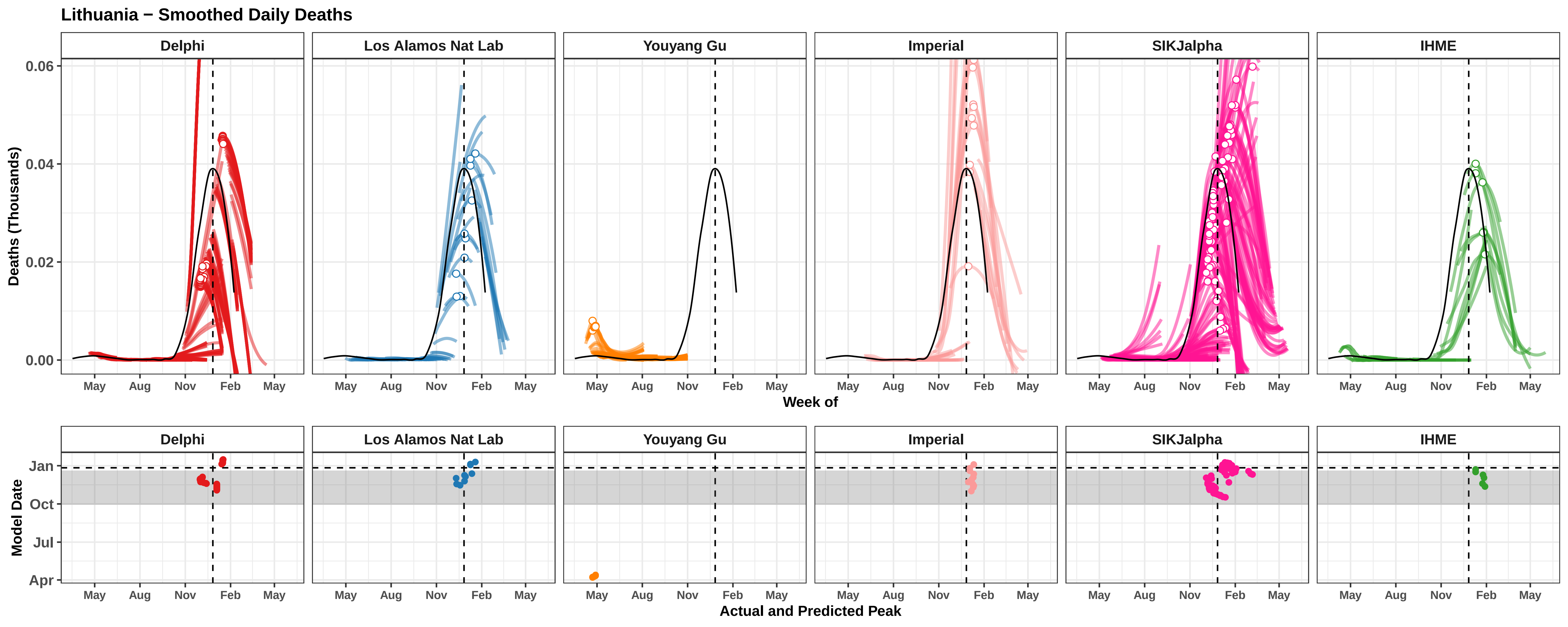

Lithuania − Smoothed Daily Deaths
Delphi
Los Alamos Nat Lab
Youyang Gu
Imperial
SIKJalpha
IHME
Deaths (Thousands)
0.06
0.04
0.02
0.00
May
Aug
Nov
Feb
May
Week of
Model Date
Jan
Oct
Jul
Apr
Actual and Predicted Peak

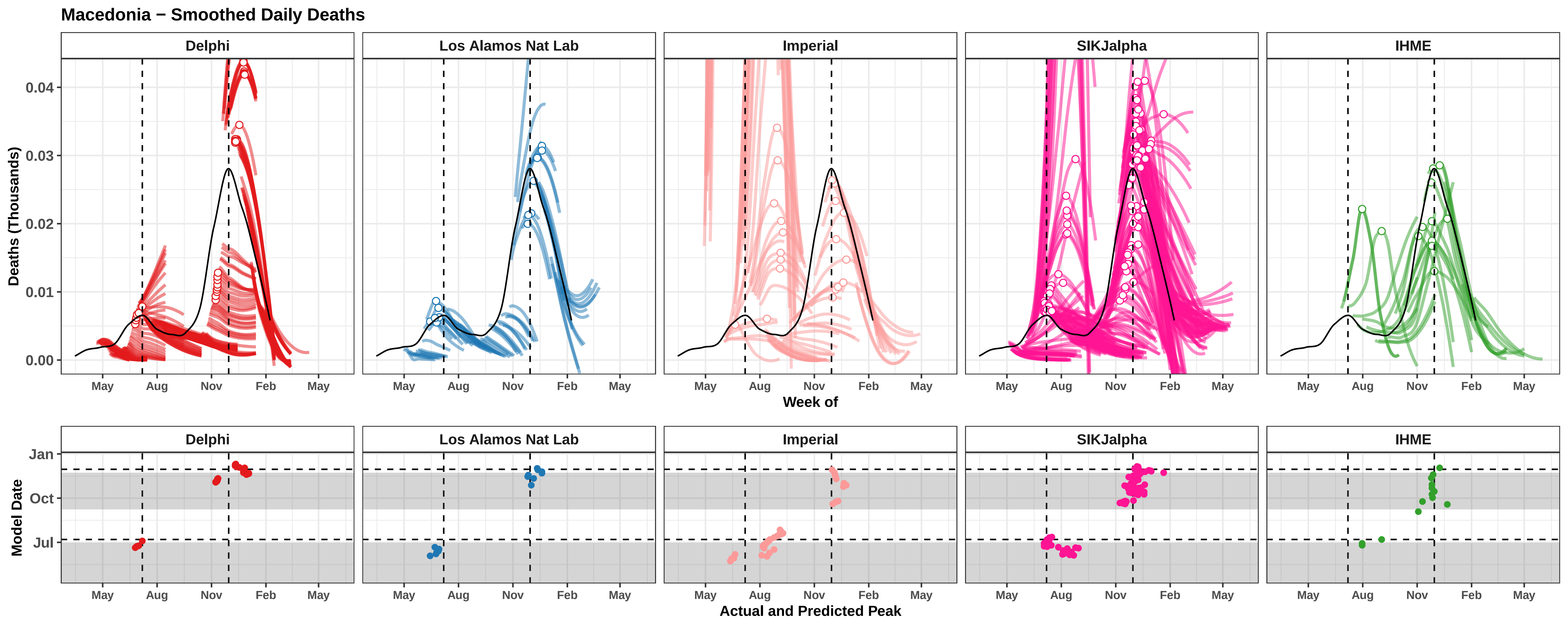
Macedonia − Smoothed Daily Deaths
Delphi
Los Alamos Nat Lab
Imperial
SIKJalpha
IHME
Deaths (Thousands)
0.00
0.01
0.02
0.03
0.04
May
Aug
Nov
Feb
May
Week of
Model Date
Jan
Oct
Jul
Actual and Predicted Peak

Algeria − Smoothed Daily Deaths

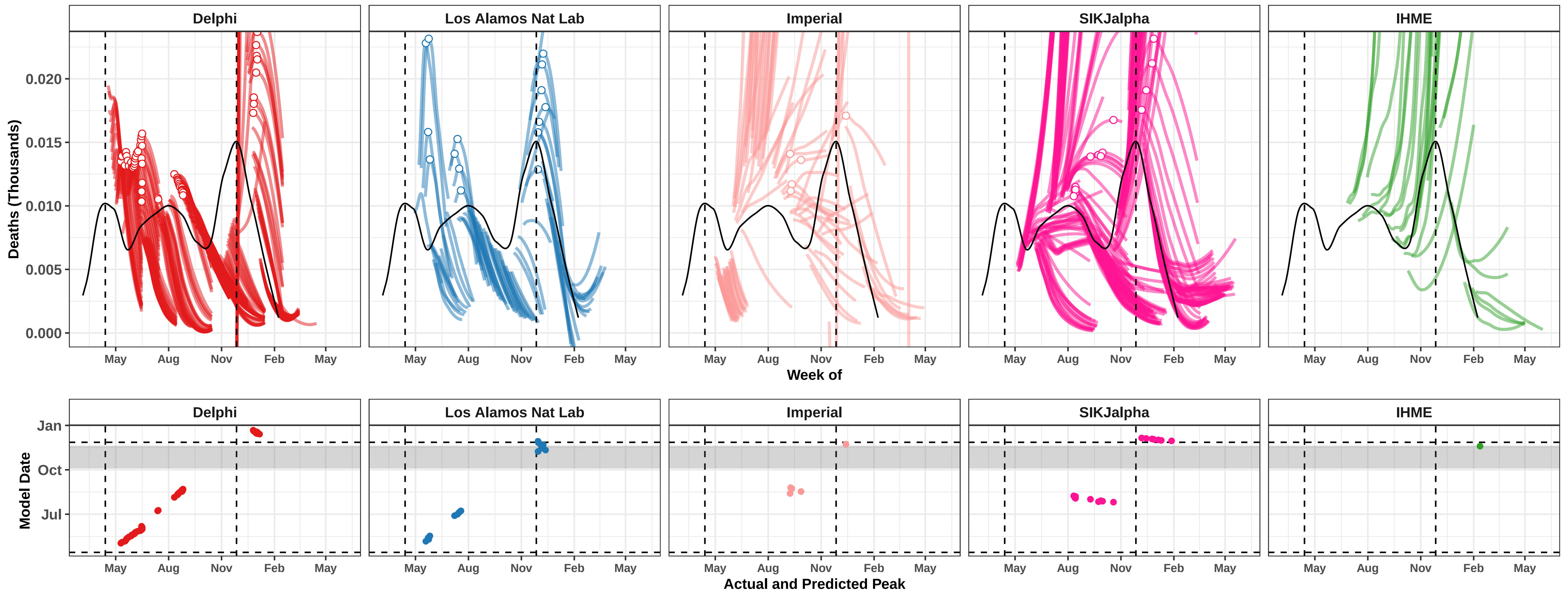

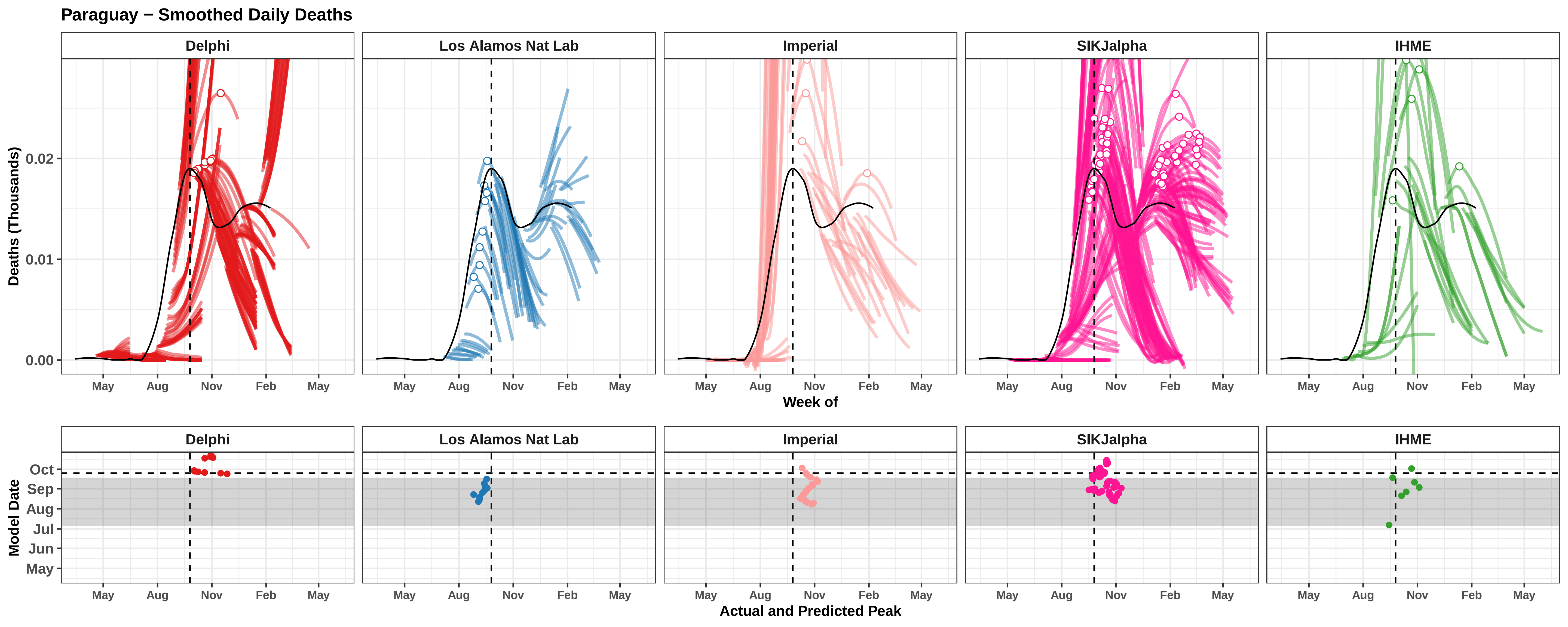
Paraguay − Smoothed Daily Deaths
Delphi
Los Alamos Nat Lab
Imperial
SIKJalpha
IHME
Deaths (Thousands)
0.00
0.01
0.02
May
Aug
Nov
Feb
May
Week of
Model Date
Oct
Sep
Aug
Jul
Jun
May
Actual and Predicted Peak

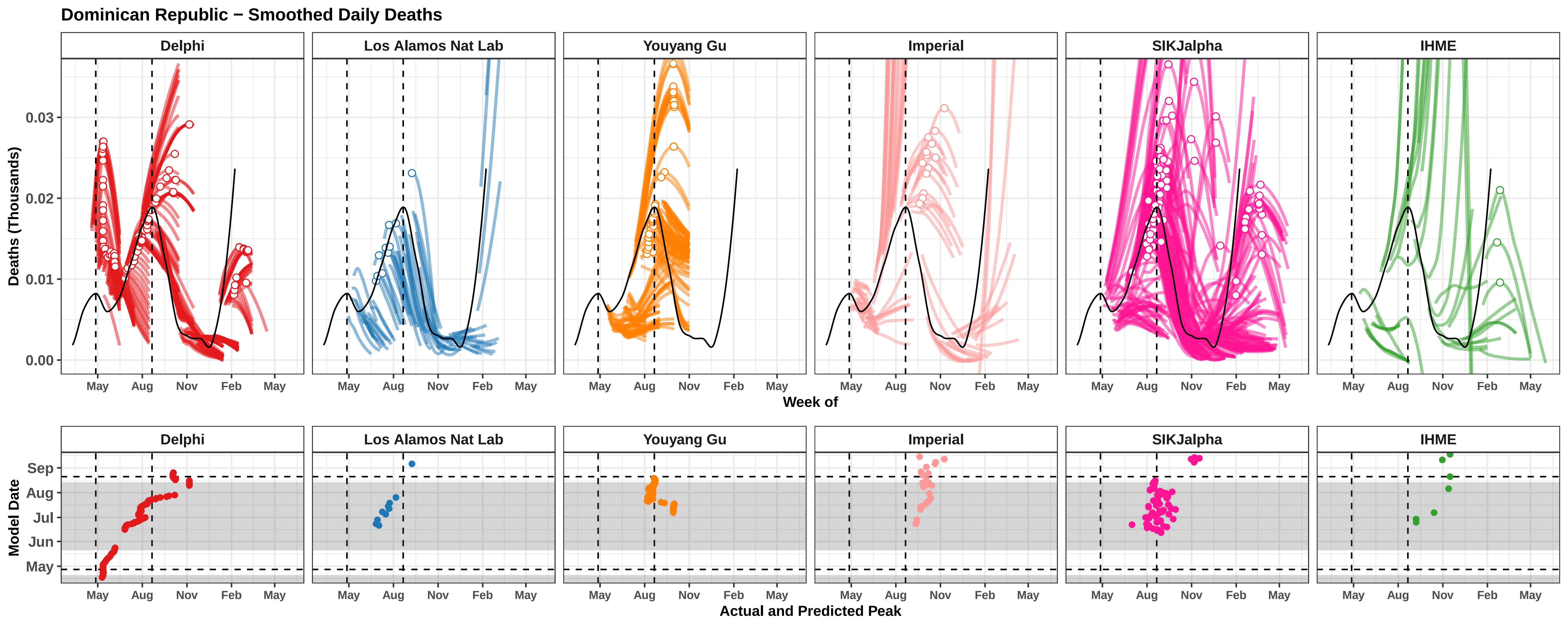

Dominican Republic − Smoothed Daily Deaths
Delphi
Los Alamos Nat Lab
Youyang Gu
Imperial
SIKJalpha
IHME
Deaths (Thousands)
0.00
0.01
0.02
0.03
May
Aug
Nov
Feb
May
Week of
Model Date
Sep
Aug
Jul
Jun
May
Actual and Predicted Peak

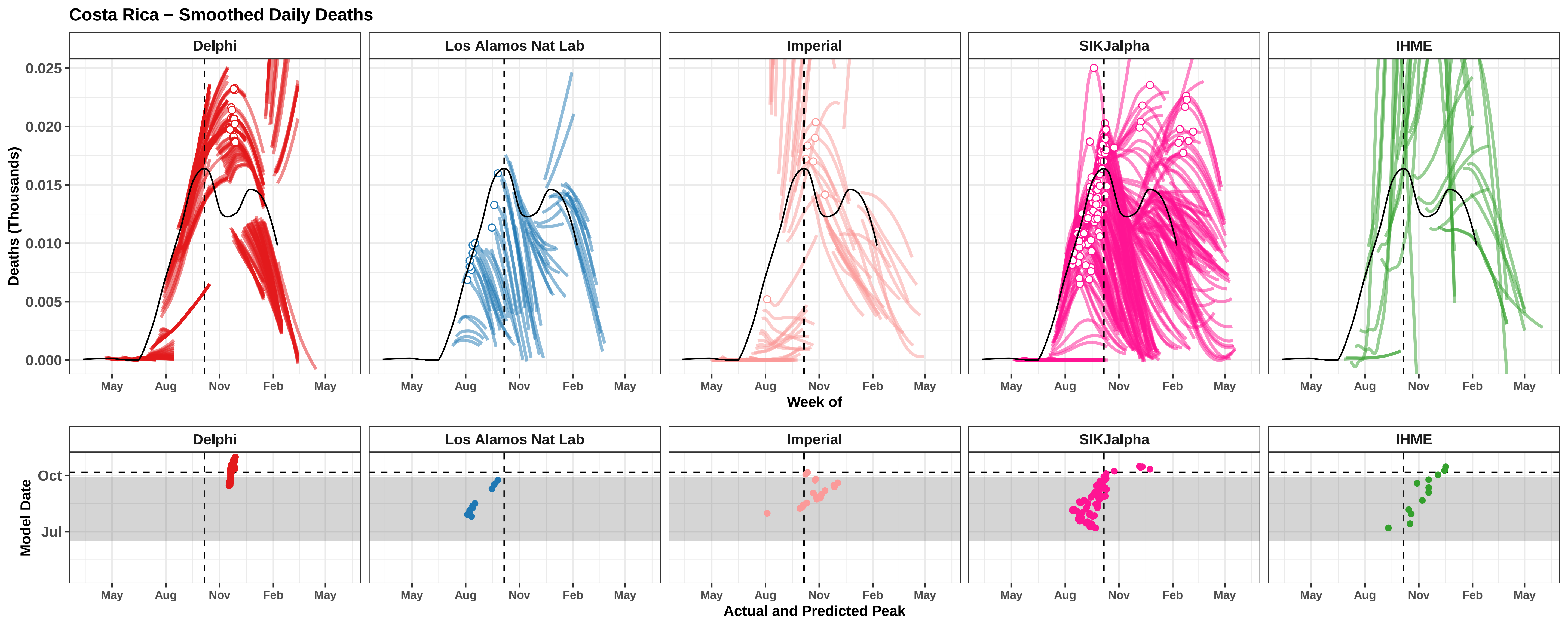

Costa Rica − Smoothed Daily Deaths
Delphi
Los Alamos Nat Lab
Imperial
SIKJalpha
IHME
Deaths (Thousands)
0.025
0.020
0.015
0.010
0.005
0.000
May
Aug
Nov
Feb
May
Week of
Model Date
Oct
Jul
Actual and Predicted Peak

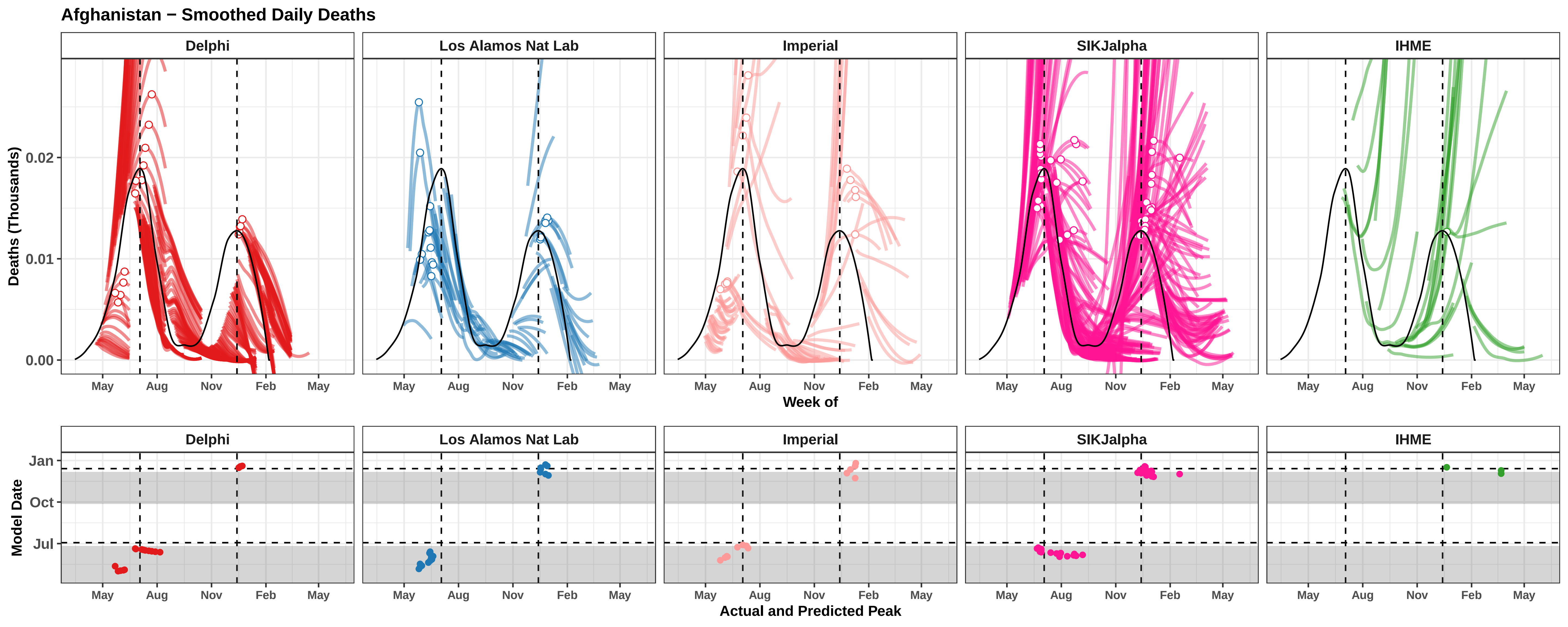
Afghanistan − Smoothed Daily Deaths
Delphi
Los Alamos Nat Lab
Imperial
SIKJalpha
IHME
Deaths (Thousands)
0.00
0.01
0.02
May
Aug
Nov
Feb
May
Week of
Model Date
Jan
Oct
Jul
Actual and Predicted Peak

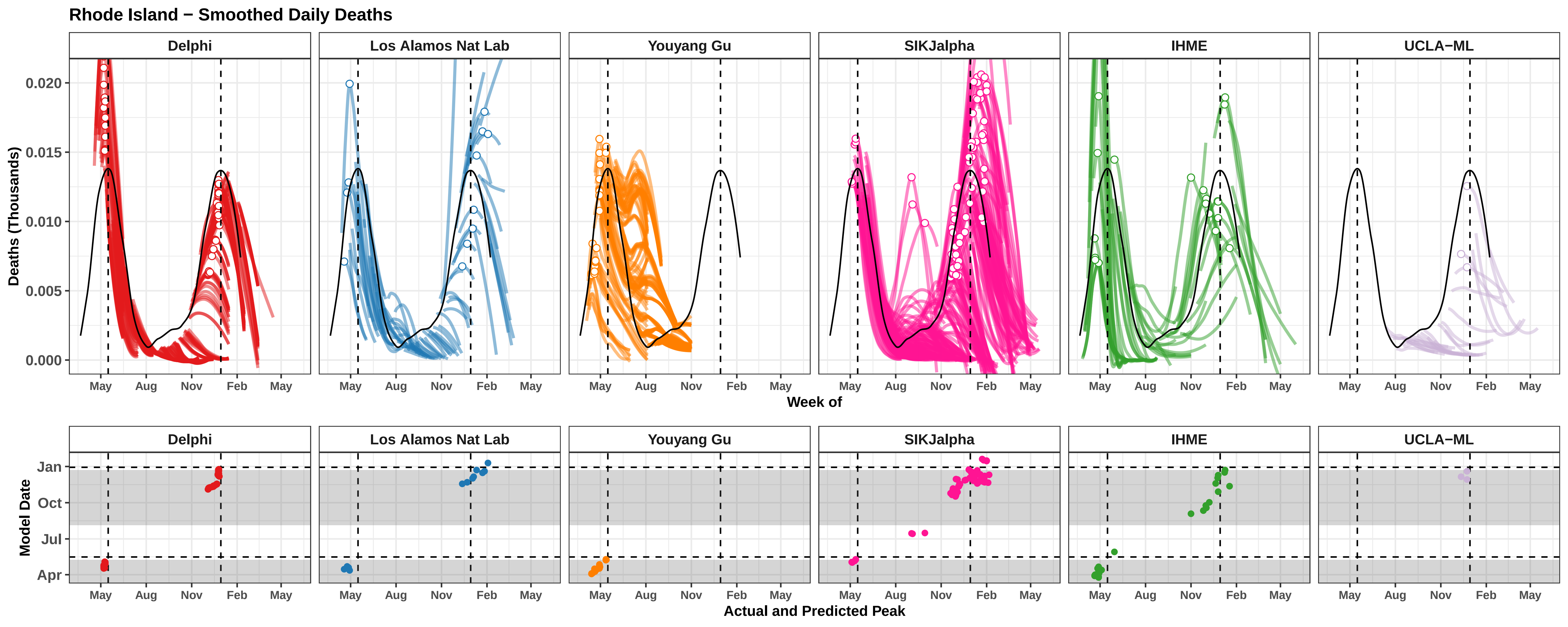

Rhode Island − Smoothed Daily Deaths
Delphi
Los Alamos Nat Lab
Youyang Gu
SIKJalpha
IHME
UCLA−ML
Deaths (Thousands)
0.020
0.015
0.010
0.005
0.000
May
Aug
Nov
Feb
May
Week of
Model Date
Jan
Oct
Jul
Apr
Actual and Predicted Peak

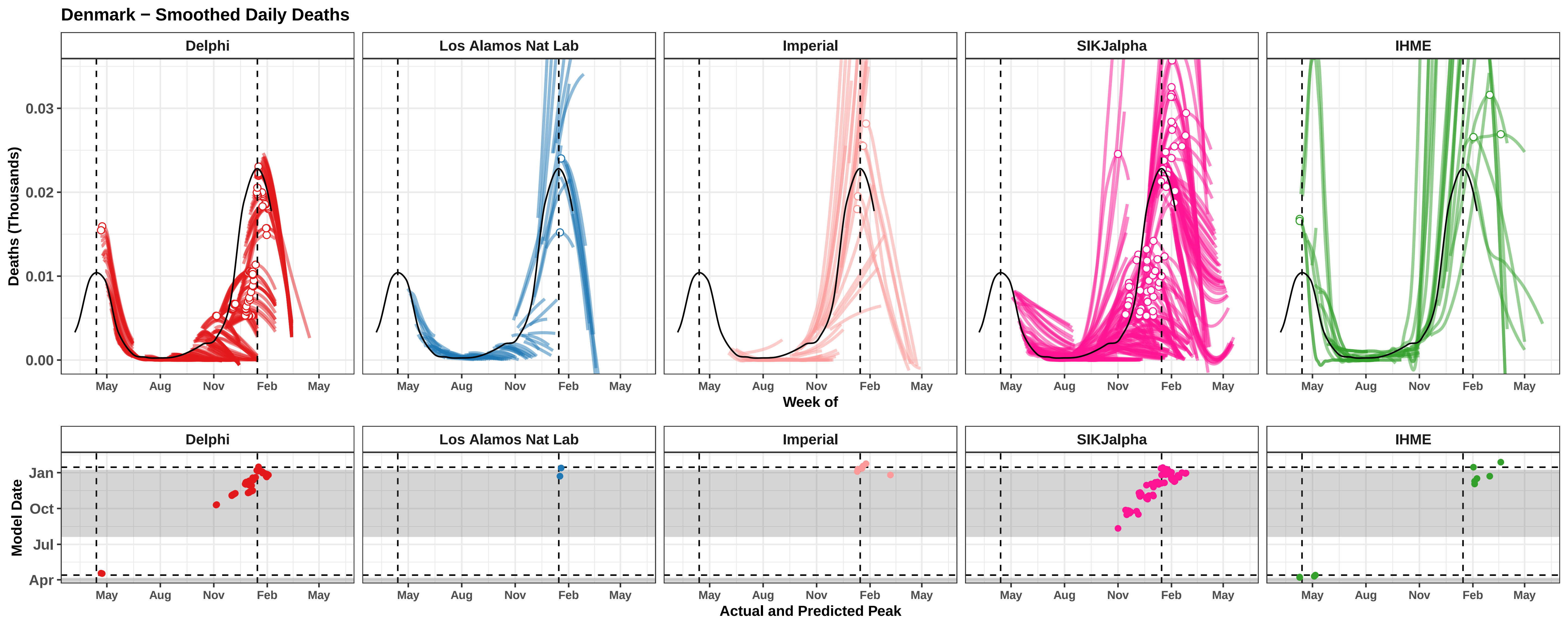

Denmark − Smoothed Daily Deaths
Delphi
Los Alamos Nat Lab
Imperial
SIKJalpha
IHME
Deaths (Thousands)
0.03
0.02
0.01
0.00
May
Aug
Nov
Feb
May
Week of
Model Date
Jan
Oct
Jul
Apr
Actual and Predicted Peak

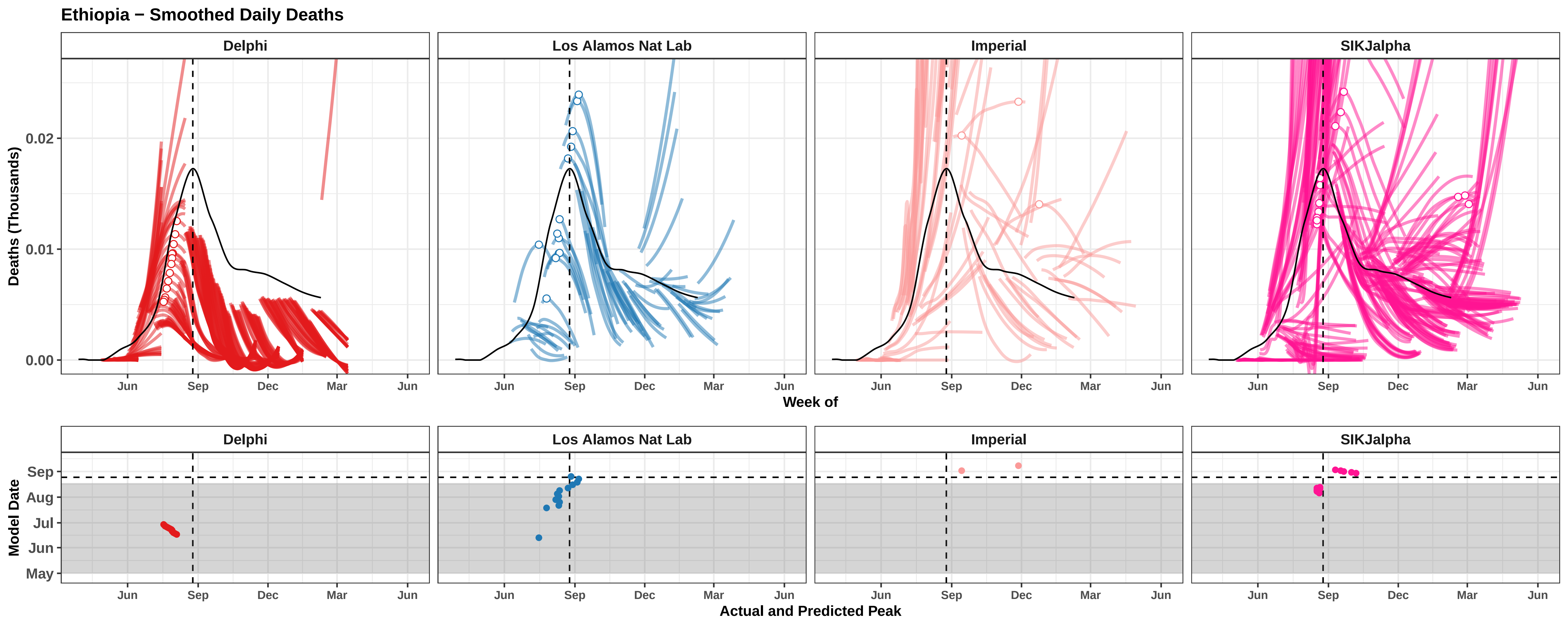

Ethiopia − Smoothed Daily Deaths
Delphi
Los Alamos Nat Lab
Imperial
SIKJalpha
Deaths (Thousands)
0.00
0.01
0.02
Jun
Sep
Dec
Mar
Jun
Week of
Model Date
May
Jun
Jul
Aug
Sep
Actual and Predicted Peak

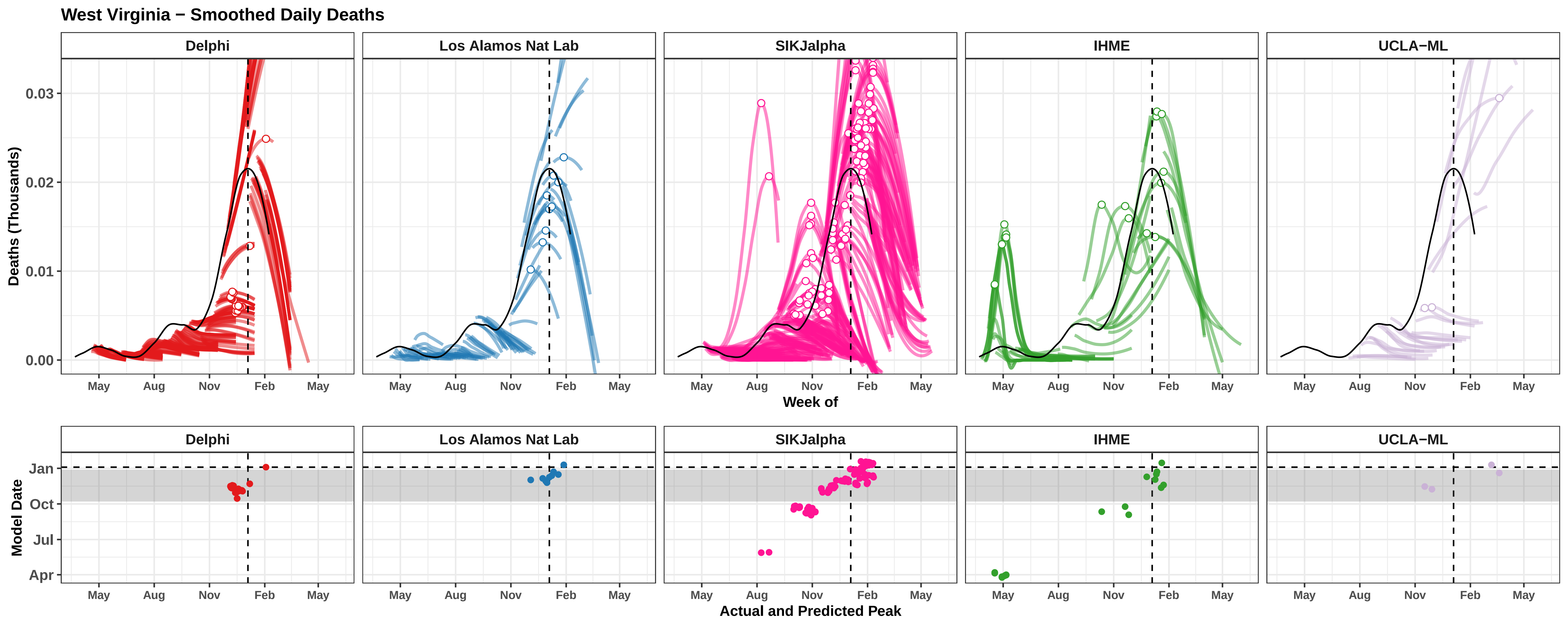

West Virginia − Smoothed Daily Deaths
Delphi
Los Alamos Nat Lab
SIKJalpha
IHME
UCLA−ML
Deaths (Thousands)
0.03
0.02
0.01
0.00
May
Aug
Nov
Feb
May
Week of
Model Date
Jan
Oct
Jul
Apr
Actual and Predicted Peak

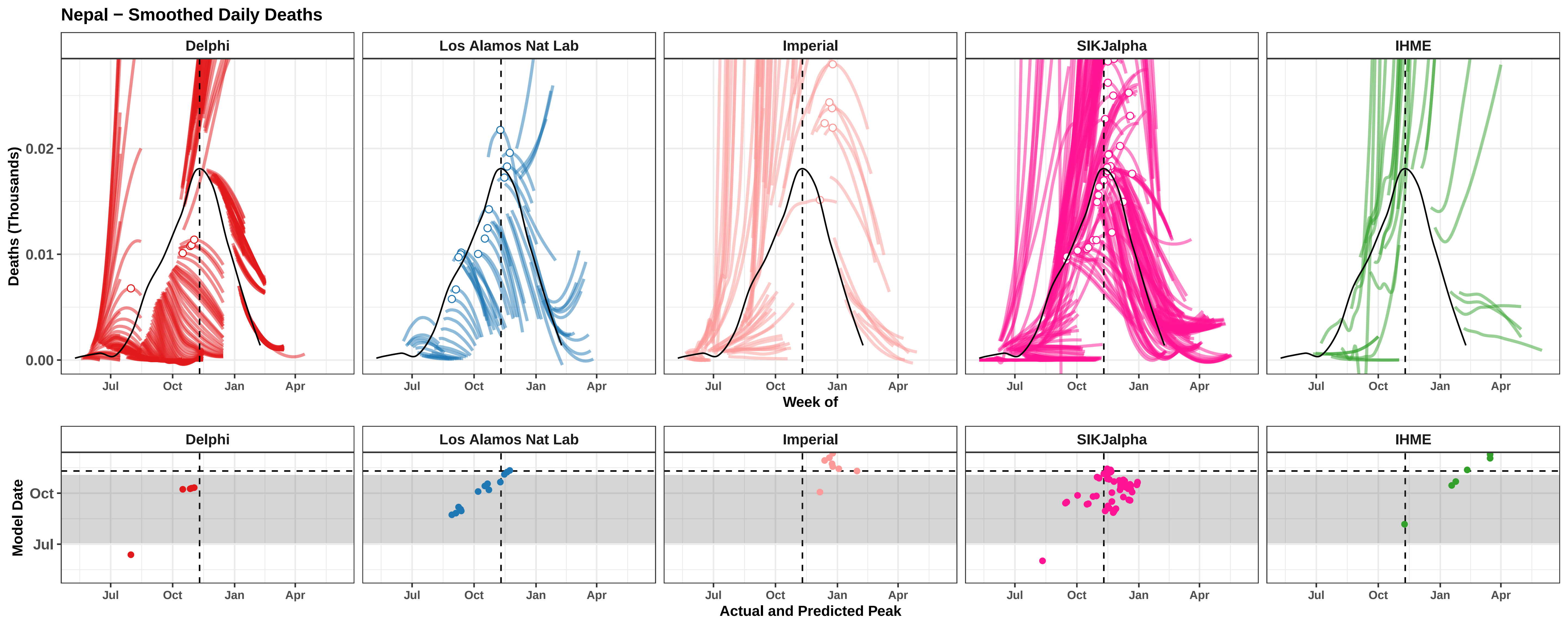
Nepal − Smoothed Daily Deaths
Delphi
Los Alamos Nat Lab
Imperial
SIKJalpha
IHME
Deaths (Thousands)
0.00
0.01
0.02
Jul
Oct
Jan
Apr
Week of
Model Date
Oct
Jul
Actual and Predicted Peak

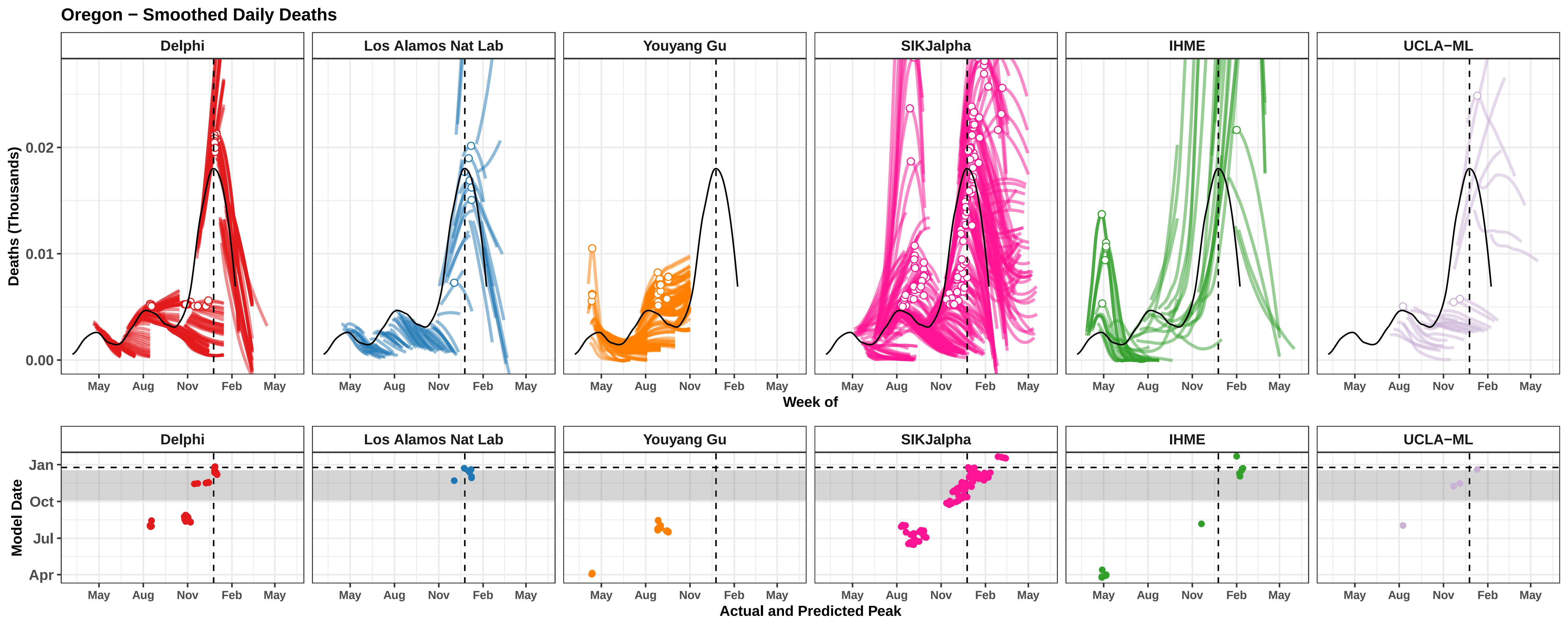

Oregon − Smoothed Daily Deaths
Delphi
Los Alamos Nat Lab
Youyang Gu
SIKJalpha
IHME
UCLA−ML
Deaths (Thousands)
0.00
0.01
0.02
May
Aug
Nov
Feb
Week of
Model Date
Apr
Jul
Oct
Jan
Actual and Predicted Peak

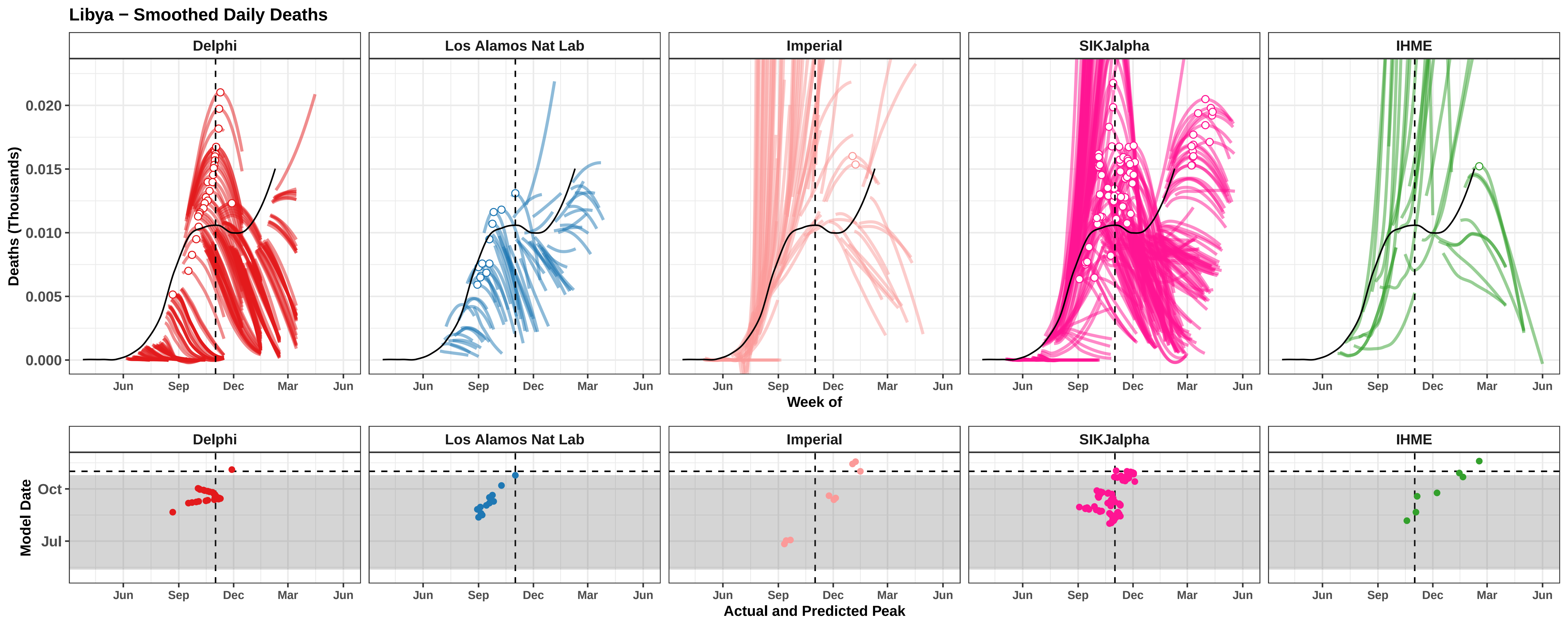

Libya − Smoothed Daily Deaths
Delphi
Los Alamos Nat Lab
Imperial
SIKJalpha
IHME
Deaths (Thousands)
0.000
0.005
0.010
0.015
0.020
Jun
Sep
Dec
Mar
Jun
Week of
Model Date
Oct
Jul
Actual and Predicted Peak

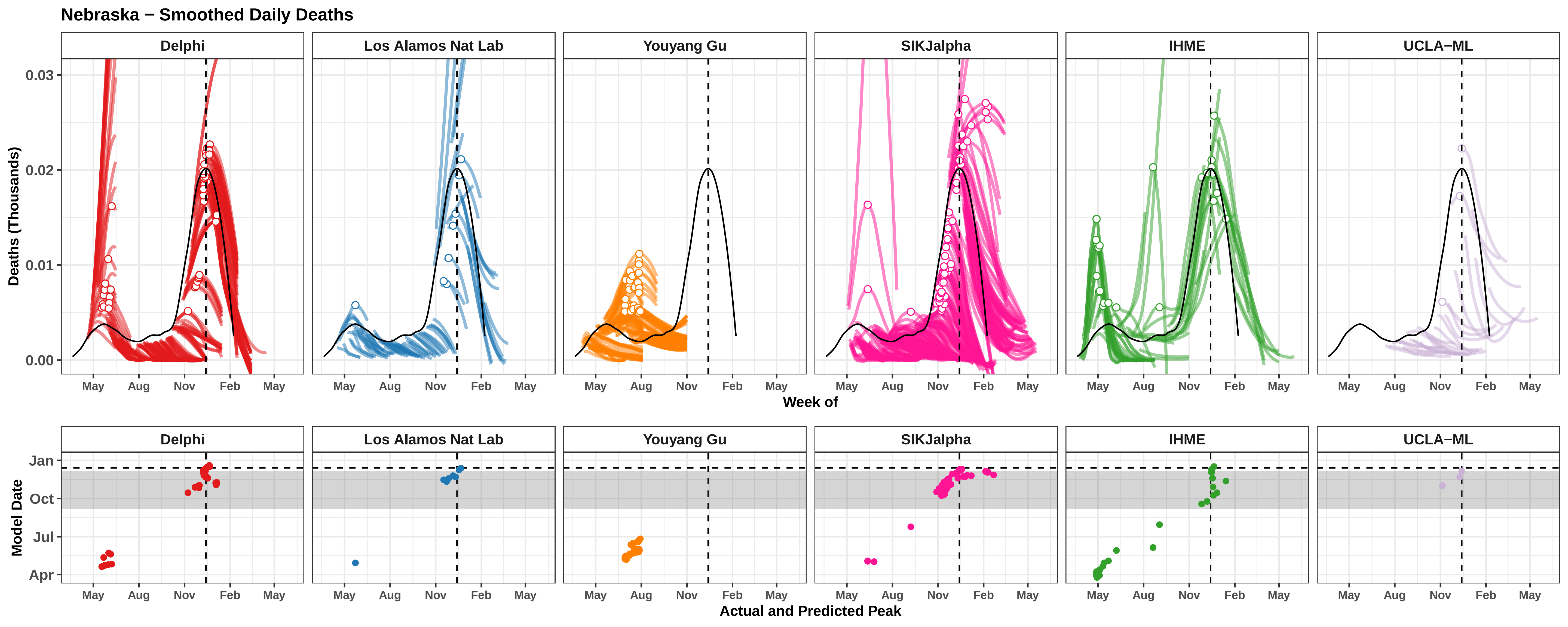
Nebraska − Smoothed Daily Deaths
Delphi
Los Alamos Nat Lab
Youyang Gu
SIKJalpha
IHME
UCLA−ML
Deaths (Thousands)
0.00
0.01
0.02
0.03
May
Aug
Nov
Feb
May
Week of
Model Date
Jan
Oct
Jul
Apr
Actual and Predicted Peak

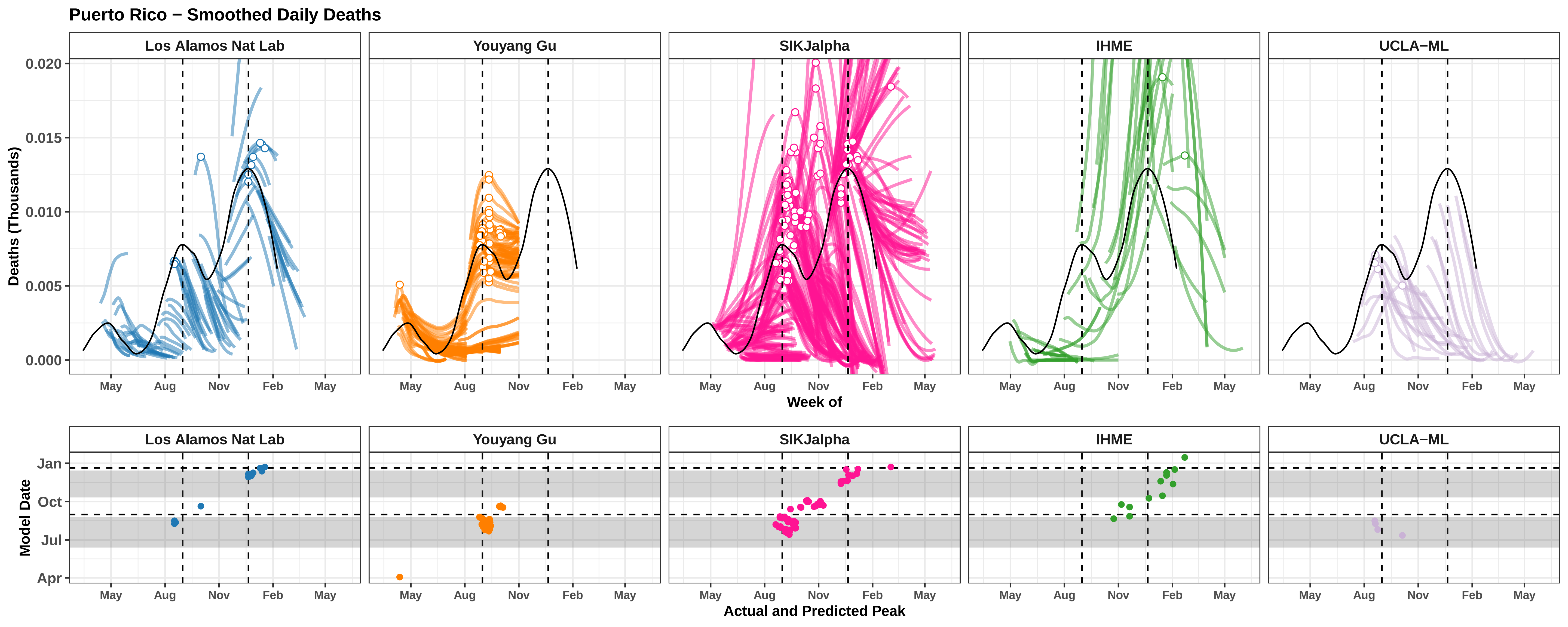

Puerto Rico − Smoothed Daily Deaths
Los Alamos Nat Lab
Youyang Gu
SIKJalpha
IHME
UCLA−ML
Deaths (Thousands)
0.020
0.015
0.010
0.005
0.000
May
Aug
Nov
Feb
May
Week of
Model Date
Jan
Oct
Jul
Apr
Actual and Predicted Peak

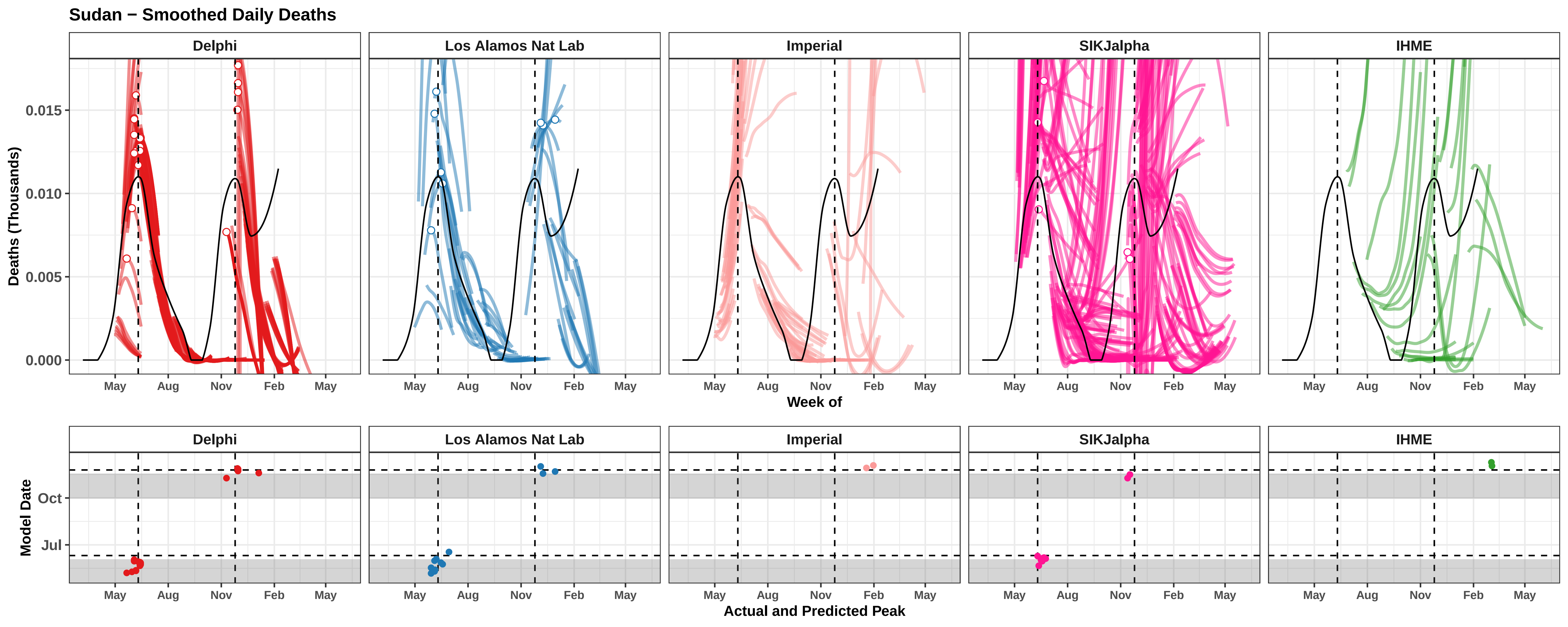

Sudan − Smoothed Daily Deaths
Delphi
Los Alamos Nat Lab
Imperial
SIKJalpha
IHME
Deaths (Thousands)
0.015
0.010
0.005
0.000
May
Aug
Nov
Feb
May
Week of
Model Date
Oct
Jul
Actual and Predicted Peak

South Dakota − Smoothed Daily Deaths

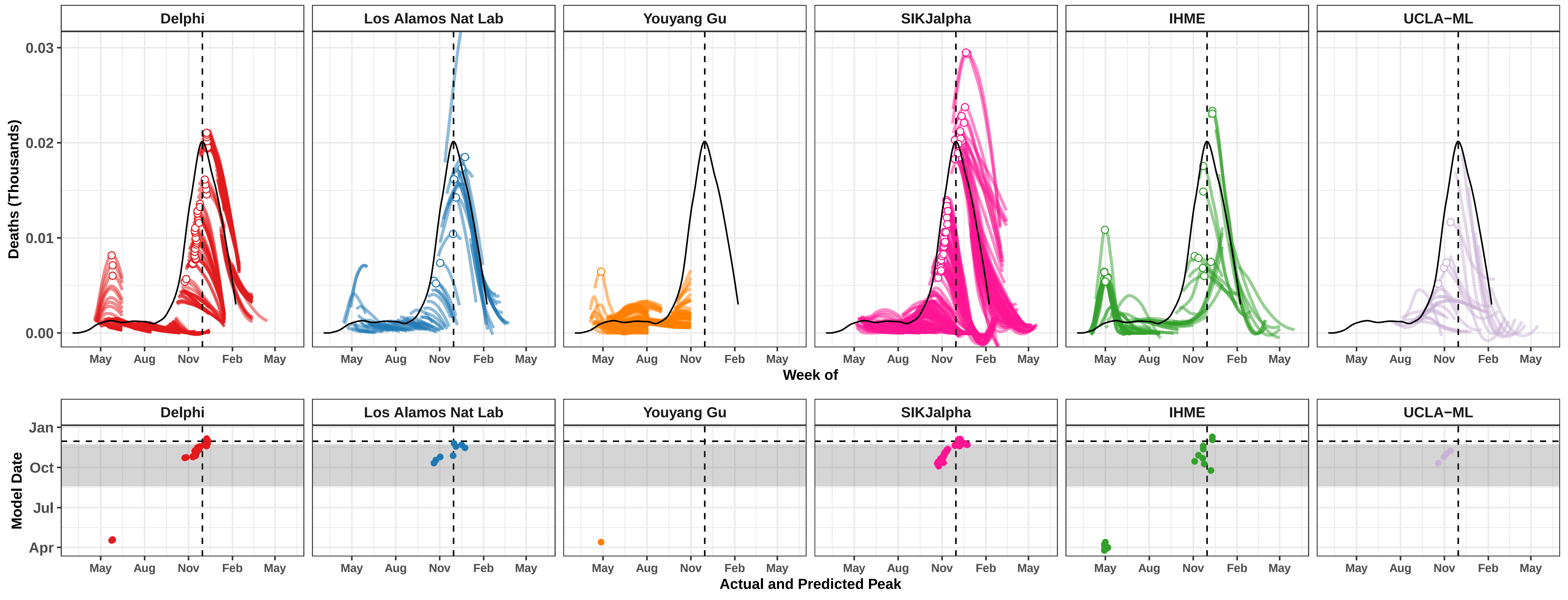

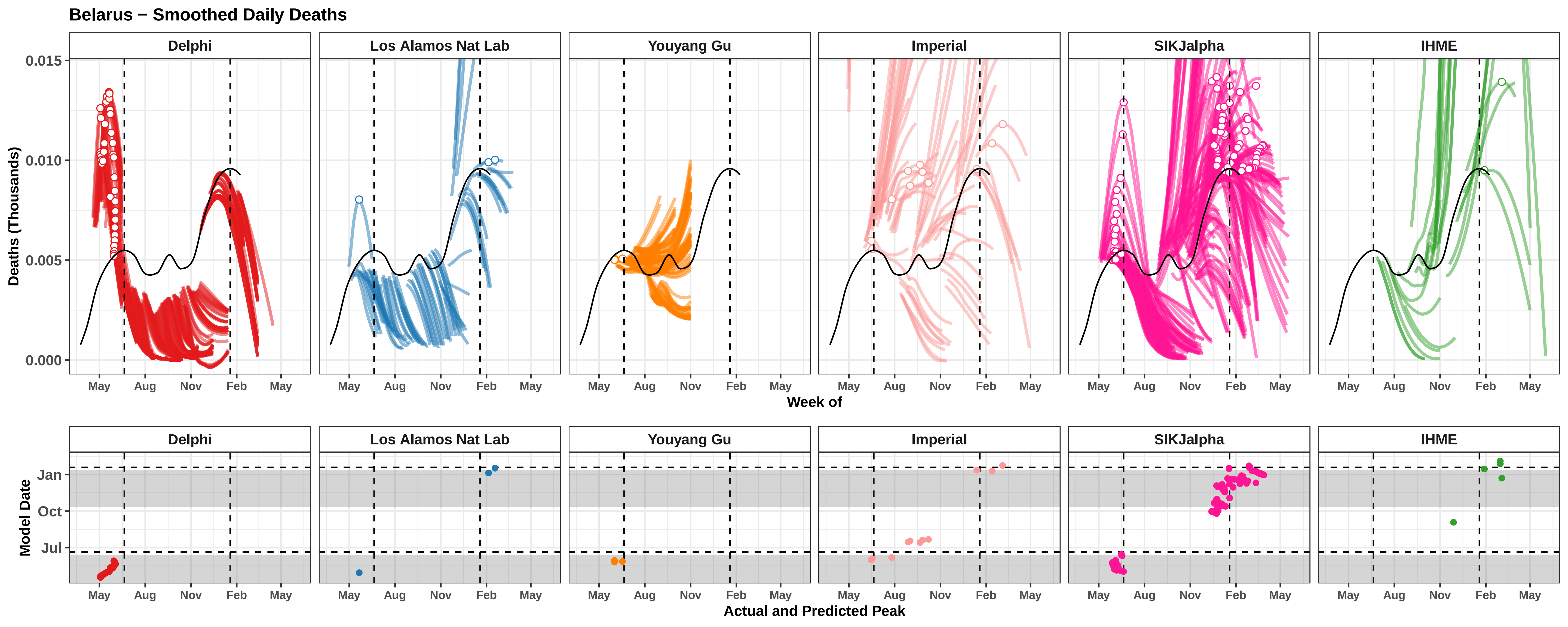

Belarus − Smoothed Daily Deaths
Delphi
Los Alamos Nat Lab
Youyang Gu
Imperial
SIKJalpha
IHME
Deaths (Thousands)
0.015
0.010
0.005
0.000
May
Aug
Nov
Feb
May
Week of
Model Date
Jan
Oct
Jul
Actual and Predicted Peak

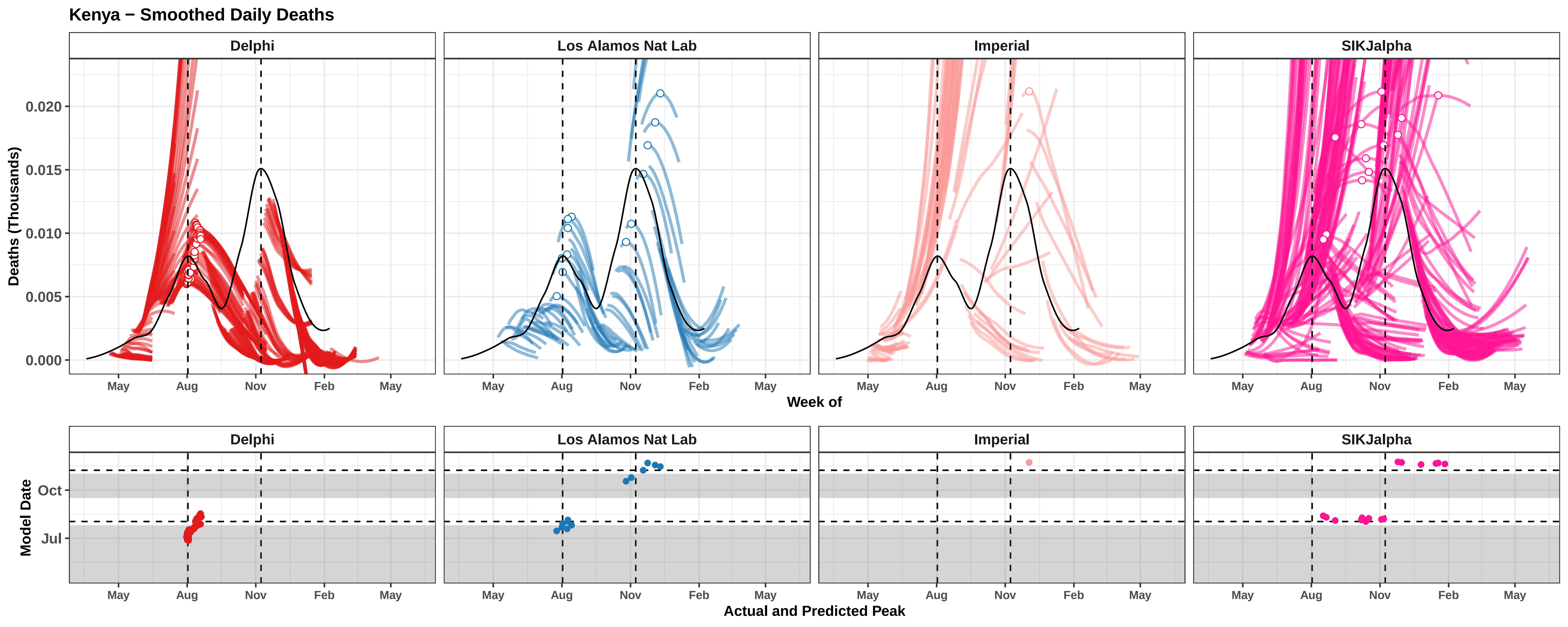

Kenya − Smoothed Daily Deaths
Delphi
Los Alamos Nat Lab
Imperial
SIKJalpha
Deaths (Thousands)
0.020
0.015
0.010
0.005
0.000
May
Aug
Nov
Feb
May
Week of
Model Date
Oct
Jul
Actual and Predicted Peak

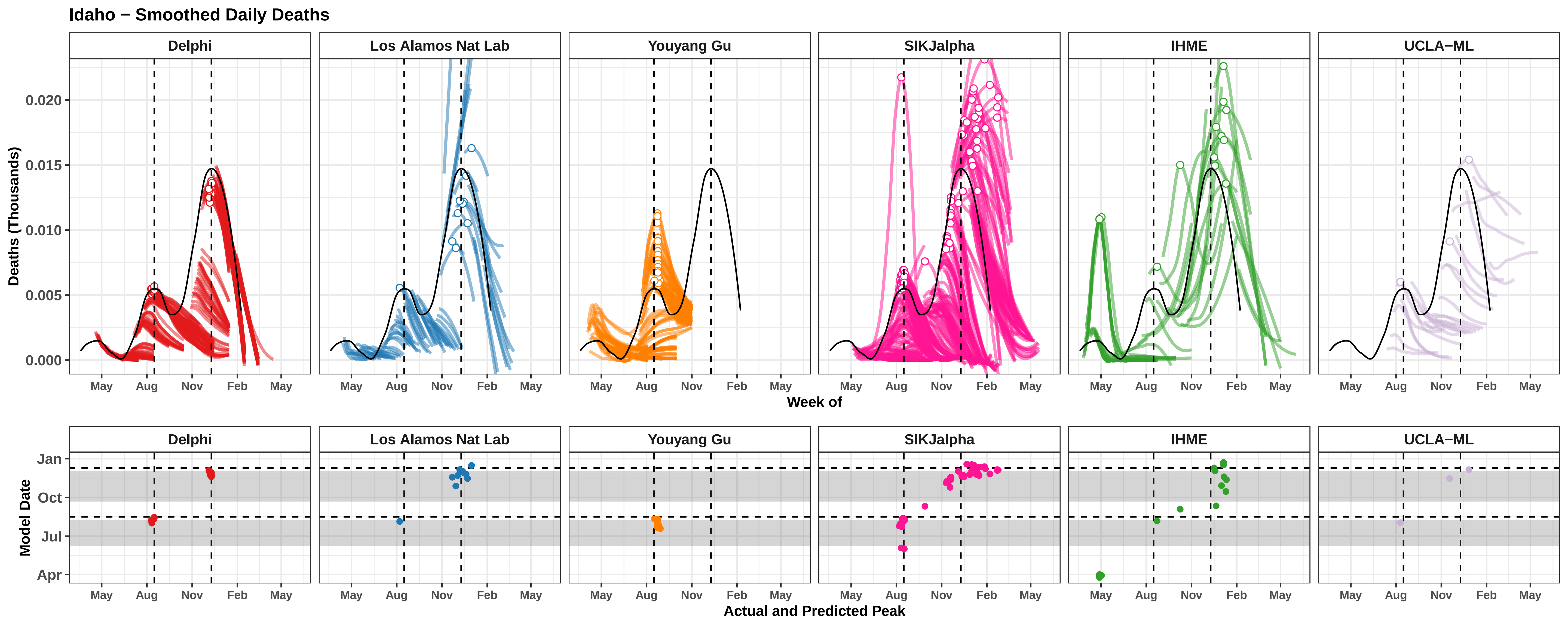

Idaho − Smoothed Daily Deaths
Delphi
Los Alamos Nat Lab
Youyang Gu
SIKJalpha
IHME
UCLA−ML
Deaths (Thousands)
0.020
0.015
0.010
0.005
0.000
May
Aug
Nov
Feb
May
Week of
Model Date
Jan
Oct
Jul
Apr
Actual and Predicted Peak

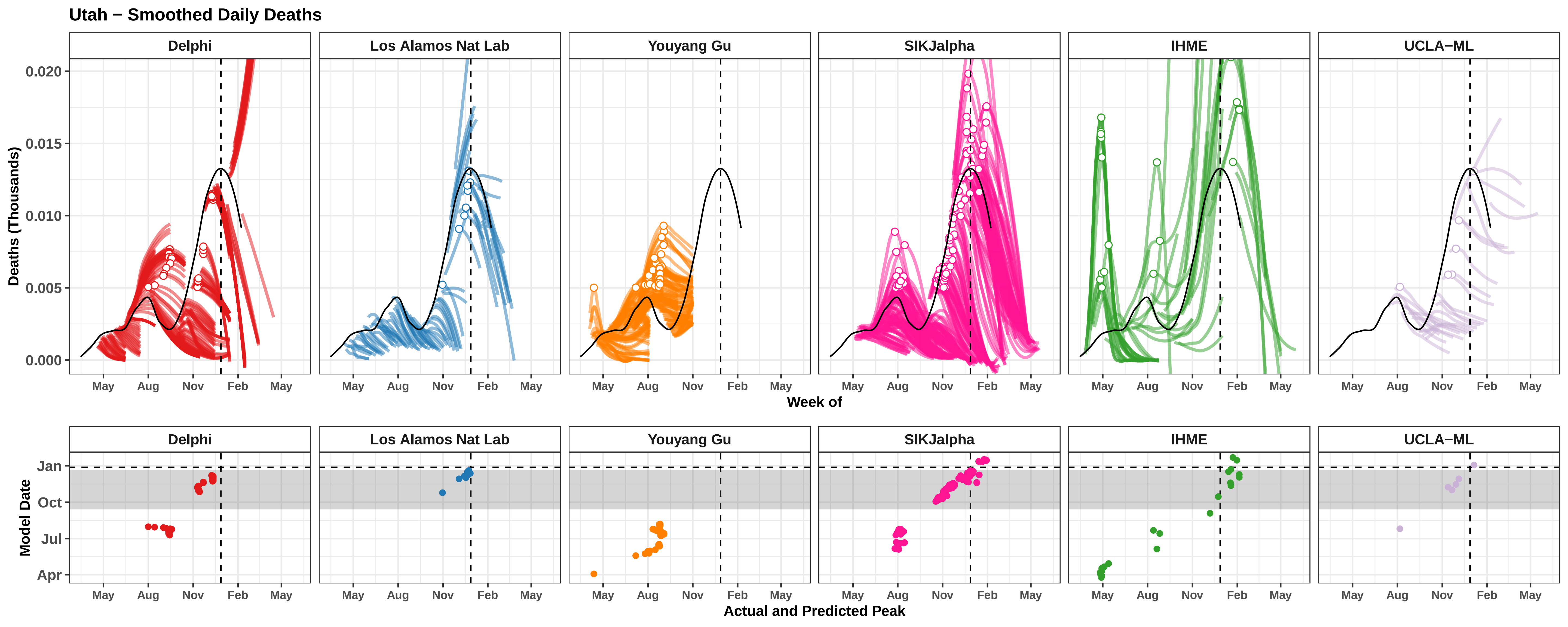

Utah − Smoothed Daily Deaths
Delphi
Los Alamos Nat Lab
Youyang Gu
SIKJalpha
IHME
UCLA−ML
Deaths (Thousands)
0.000
0.005
0.010
0.015
0.020
May
Aug
Nov
Feb
May
Week of
Model Date
Apr
Jul
Oct
Jan
Actual and Predicted Peak

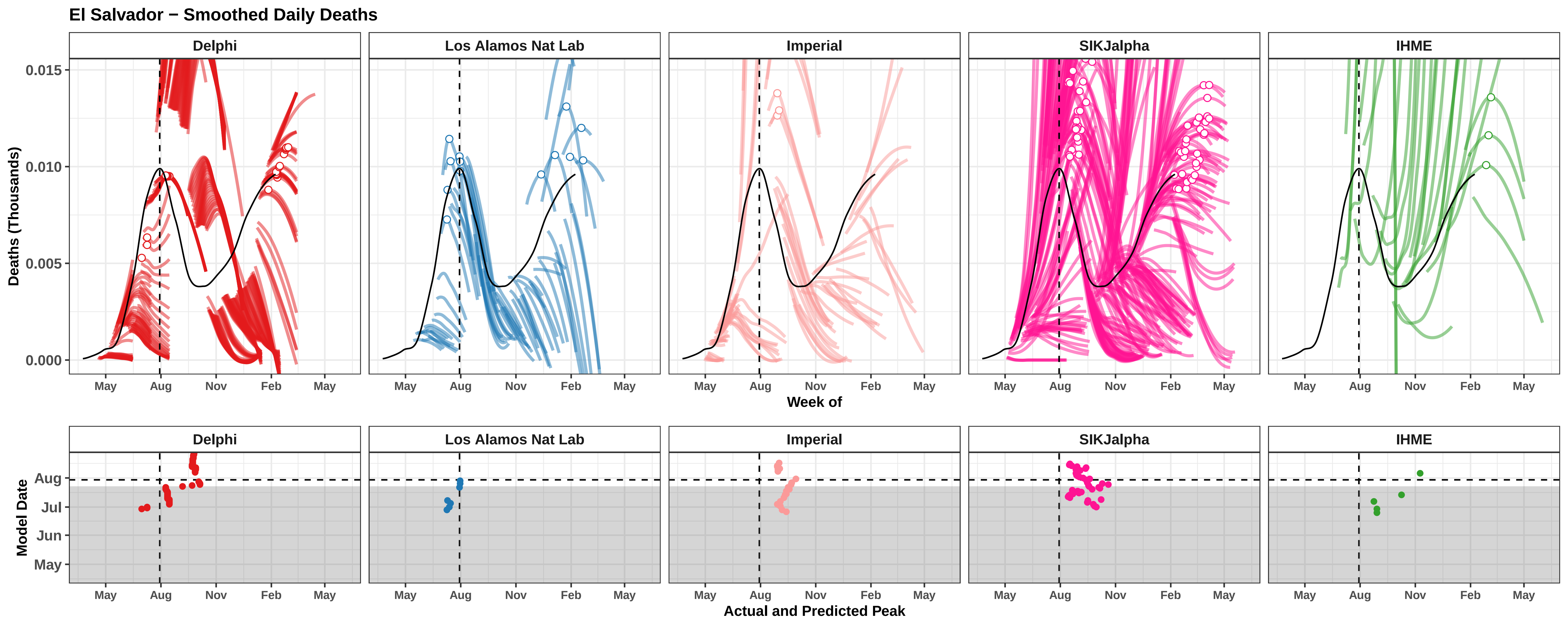

El Salvador − Smoothed Daily Deaths
Delphi
Los Alamos Nat Lab
Imperial
SIKJalpha
IHME
Deaths (Thousands)
0.015
0.010
0.005
0.000
May
Aug
Nov
Feb
May
Week of
Model Date
Aug
Jul
Jun
May
Actual and Predicted Peak

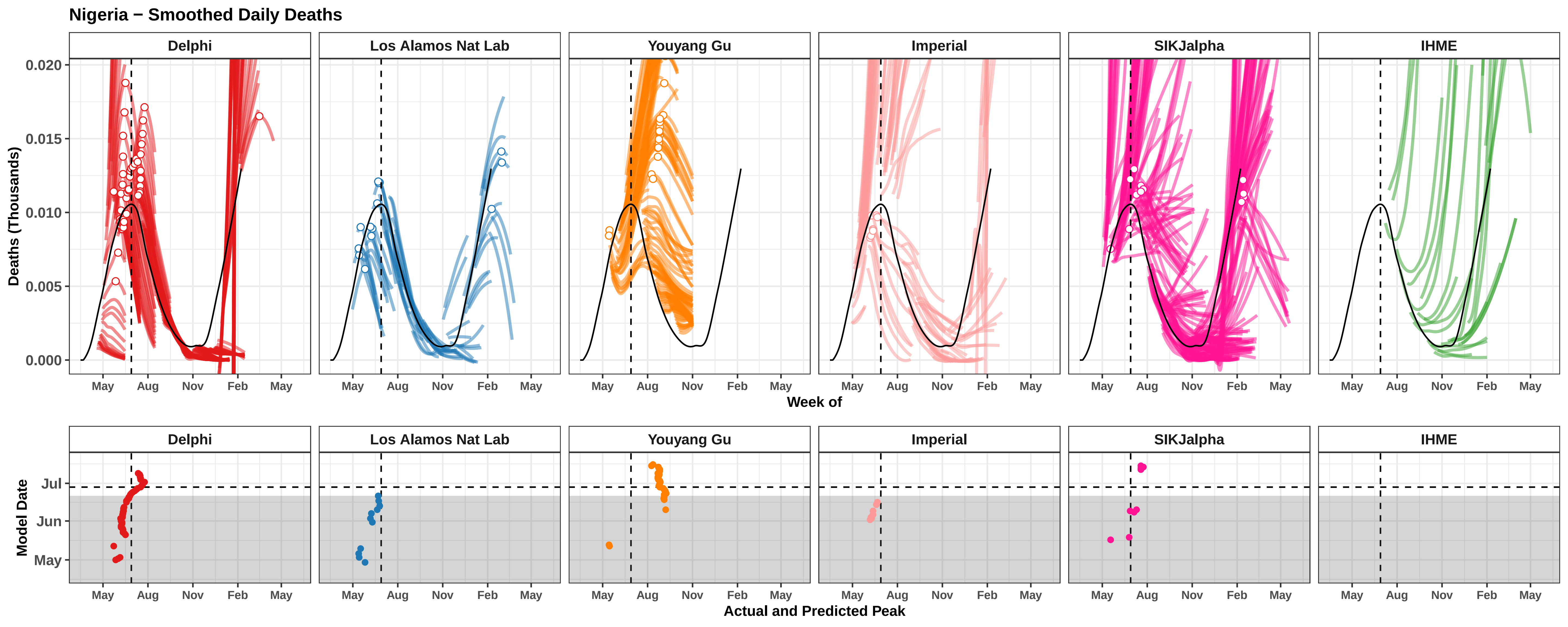

Nigeria − Smoothed Daily Deaths
Delphi
Los Alamos Nat Lab
Youyang Gu
Imperial
SIKJalpha
IHME
Deaths (Thousands)
0.000
0.005
0.010
0.015
0.020
May
Aug
Nov
Feb
May
Week of
Model Date
Jul
Jun
May
Actual and Predicted Peak

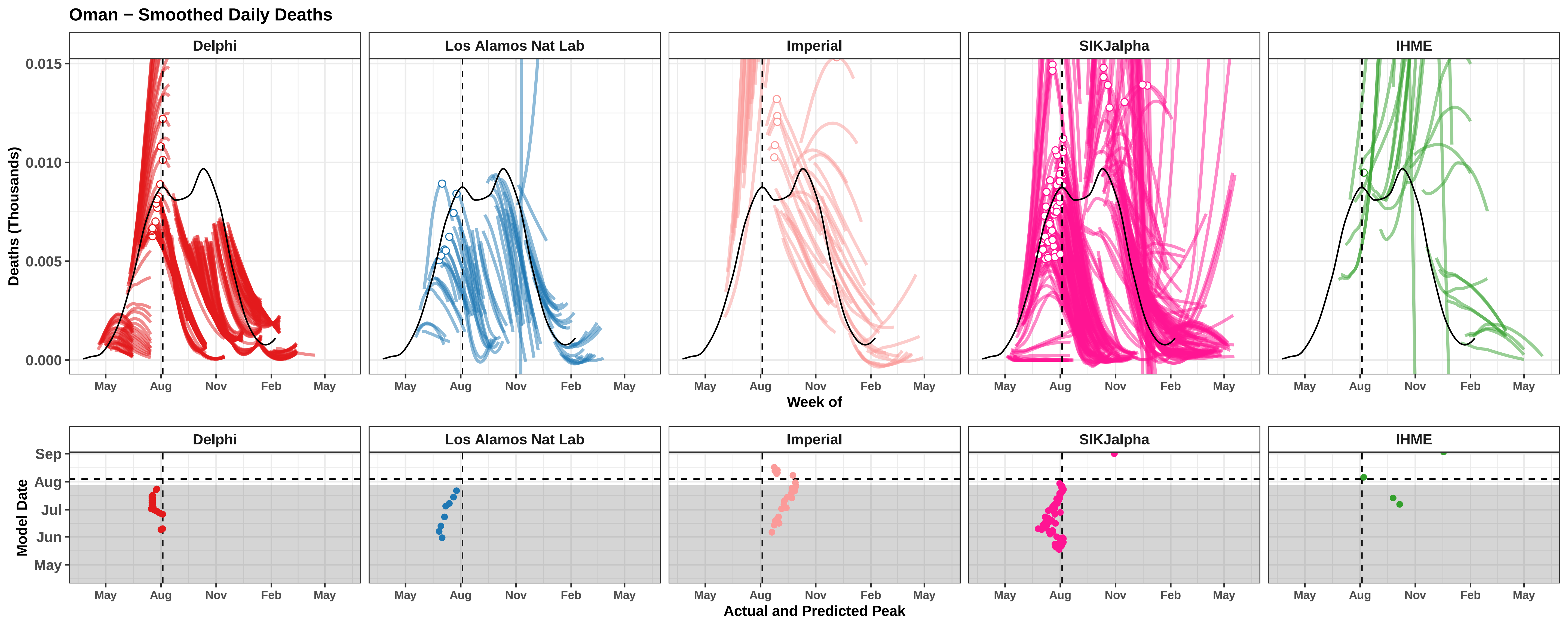

Oman − Smoothed Daily Deaths
Delphi
Los Alamos Nat Lab
Imperial
SIKJalpha
IHME
Deaths (Thousands)
0.015
0.010
0.005
0.000
May
Aug
Nov
Feb
May
Week of
Model Date
Sep
Aug
Jul
Jun
May
Actual and Predicted Peak

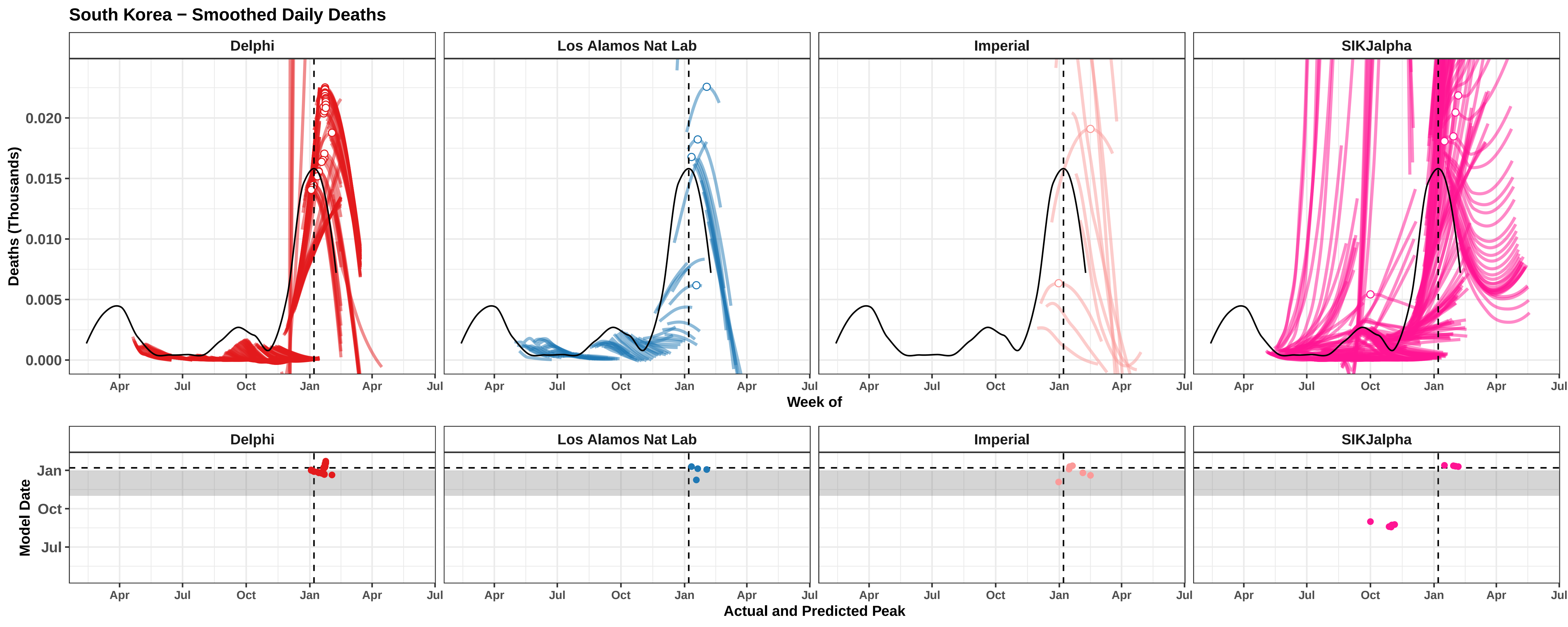
South Korea − Smoothed Daily Deaths
Delphi
Los Alamos Nat Lab
Imperial
SIKJalpha
Deaths (Thousands)
0.000
0.005
0.010
0.015
0.020
Apr
Jul
Oct
Jan
Apr
Jul
Week of
Model Date
Jan
Oct
Jul
Actual and Predicted Peak

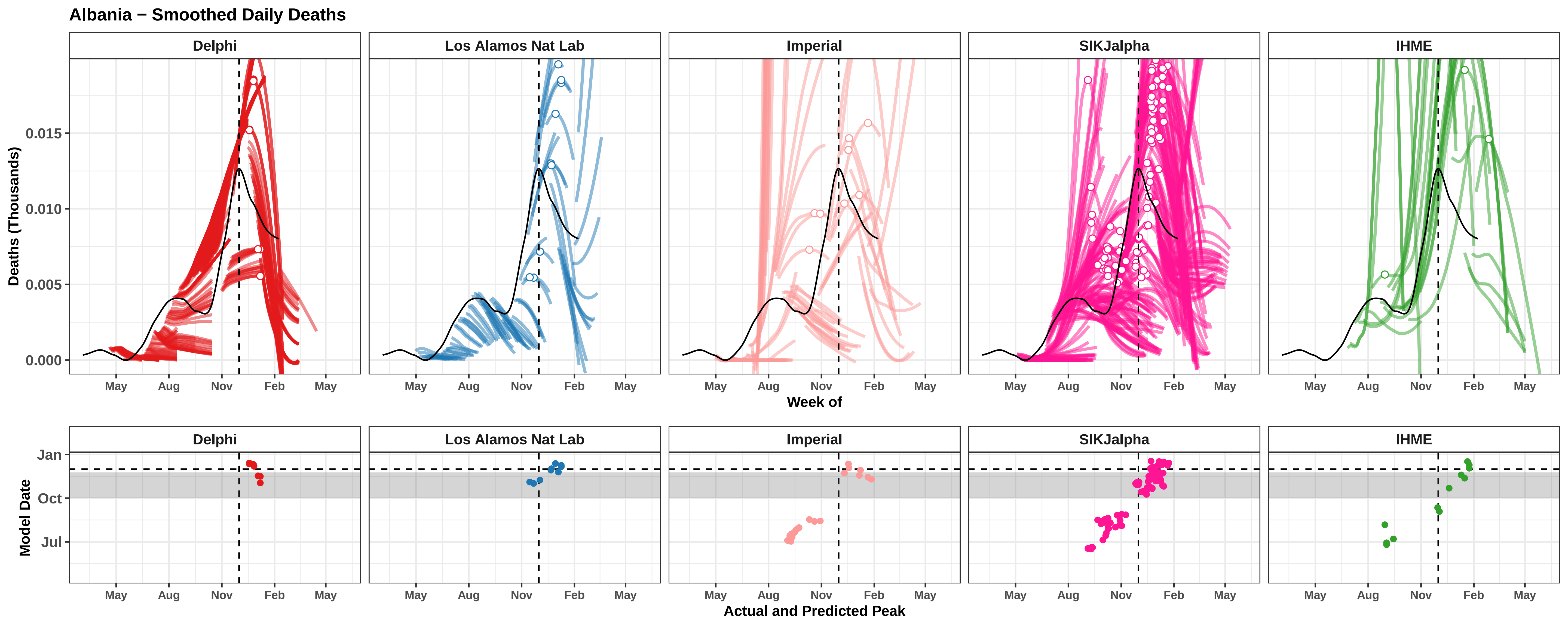
Albania − Smoothed Daily Deaths
Delphi
Los Alamos Nat Lab
Imperial
SIKJalpha
IHME
Deaths (Thousands)
0.000
0.005
0.010
0.015
May
Aug
Nov
Feb
May
Week of
Model Date
Jan
Oct
Jul
Actual and Predicted Peak

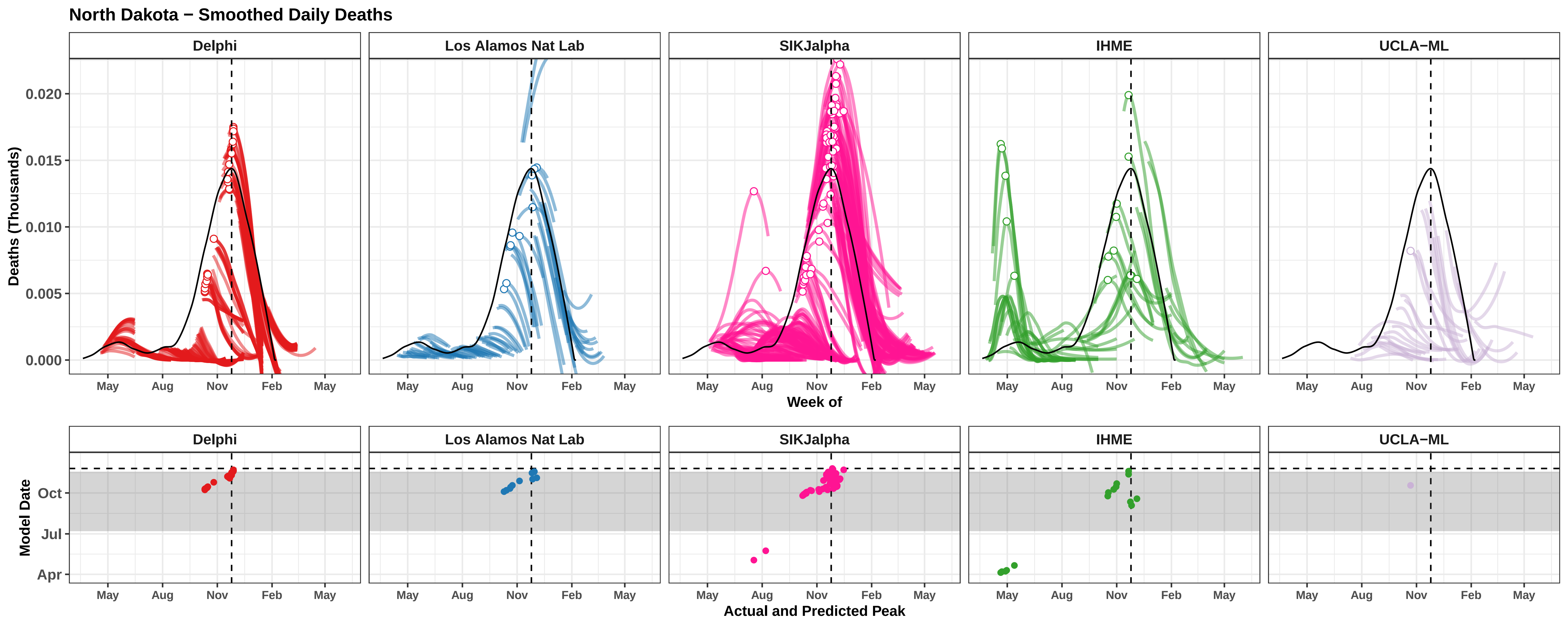

North Dakota − Smoothed Daily Deaths
Delphi
Los Alamos Nat Lab
SIKJalpha
IHME
UCLA−ML
Deaths (Thousands)
0.020
0.015
0.010
0.005
0.000
May
Aug
Nov
Feb
May
Week of
Model Date
Oct
Jul
Apr
Actual and Predicted Peak

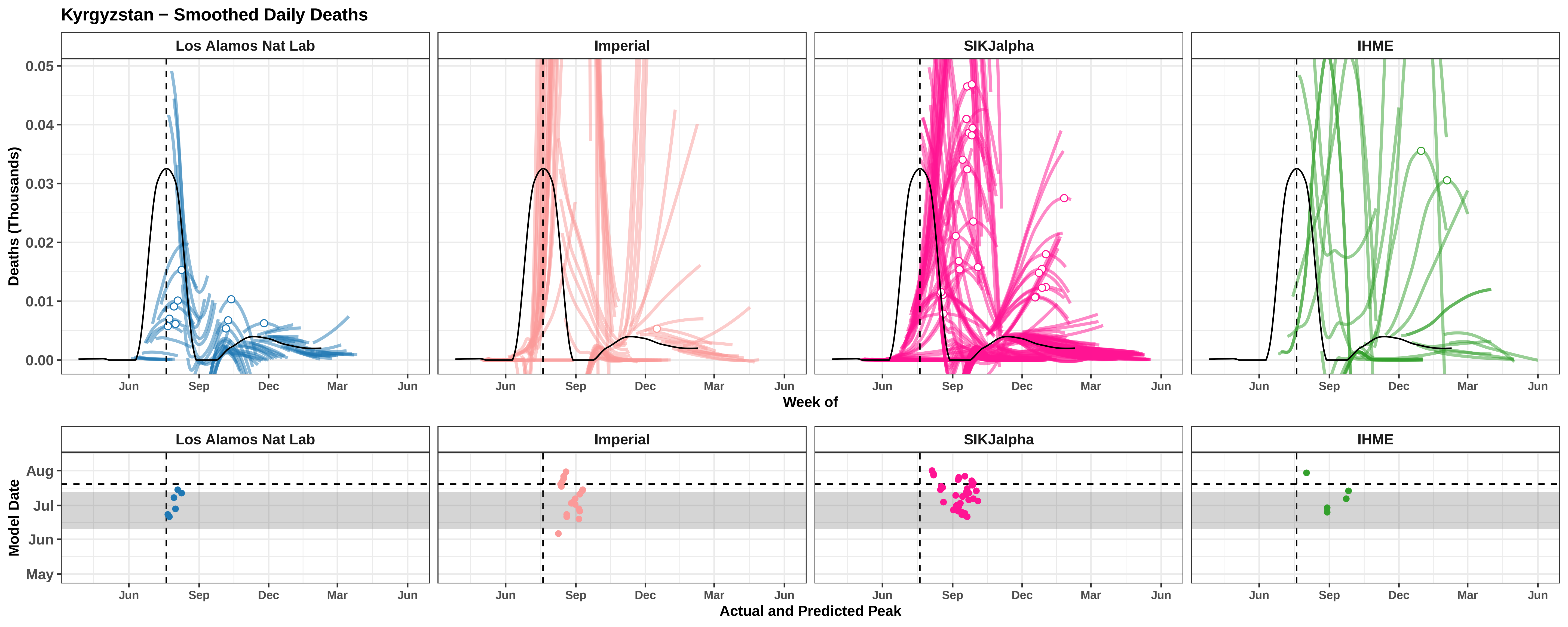
Kyrgyzstan − Smoothed Daily Deaths
Los Alamos Nat Lab
Imperial
SIKJalpha
IHME
Deaths (Thousands)
0.05
0.04
0.03
0.02
0.01
0.00
Jun
Sep
Dec
Mar
Jun
Week of
Model Date
Aug
Jul
Jun
May
Actual and Predicted Peak

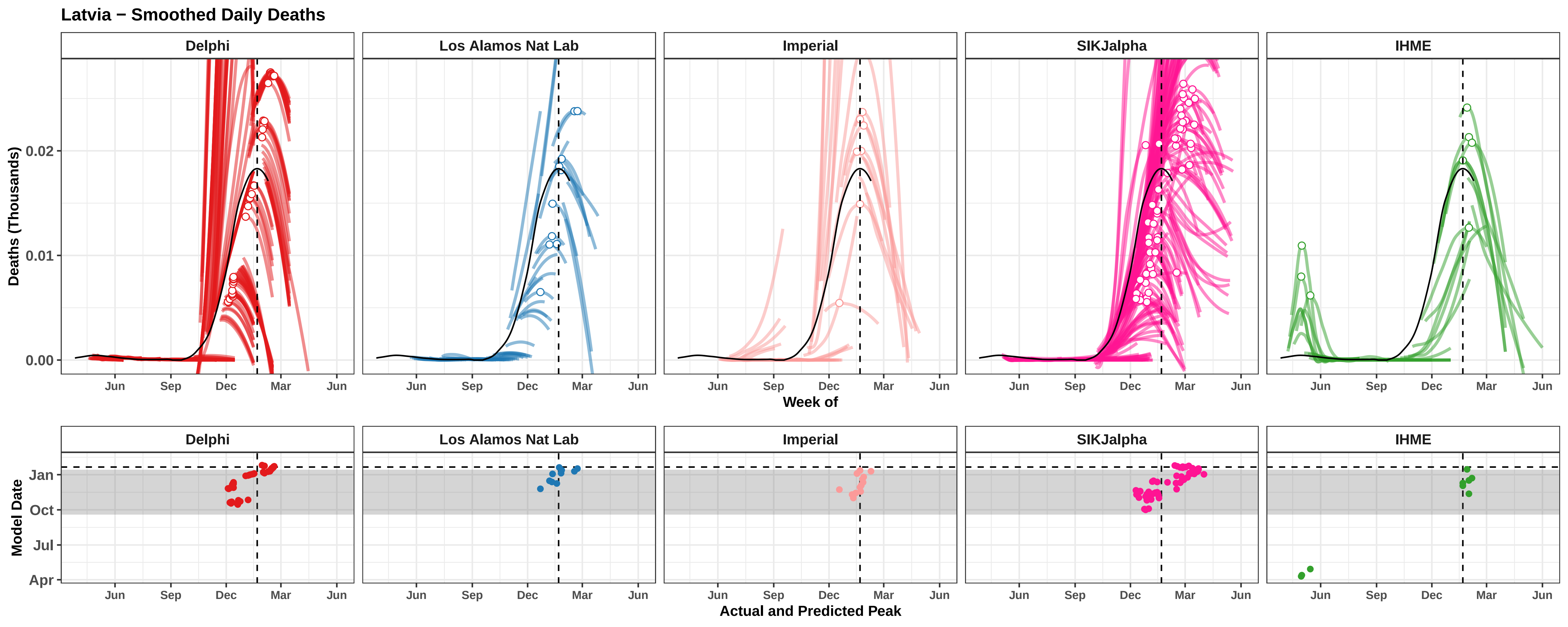
Latvia − Smoothed Daily Deaths
Delphi
Los Alamos Nat Lab
Imperial
SIKJalpha
IHME
Deaths (Thousands)
0.00
0.01
0.02
Jun
Sep
Dec
Mar
Week of
Model Date
Apr
Jul
Oct
Jan
Actual and Predicted Peak

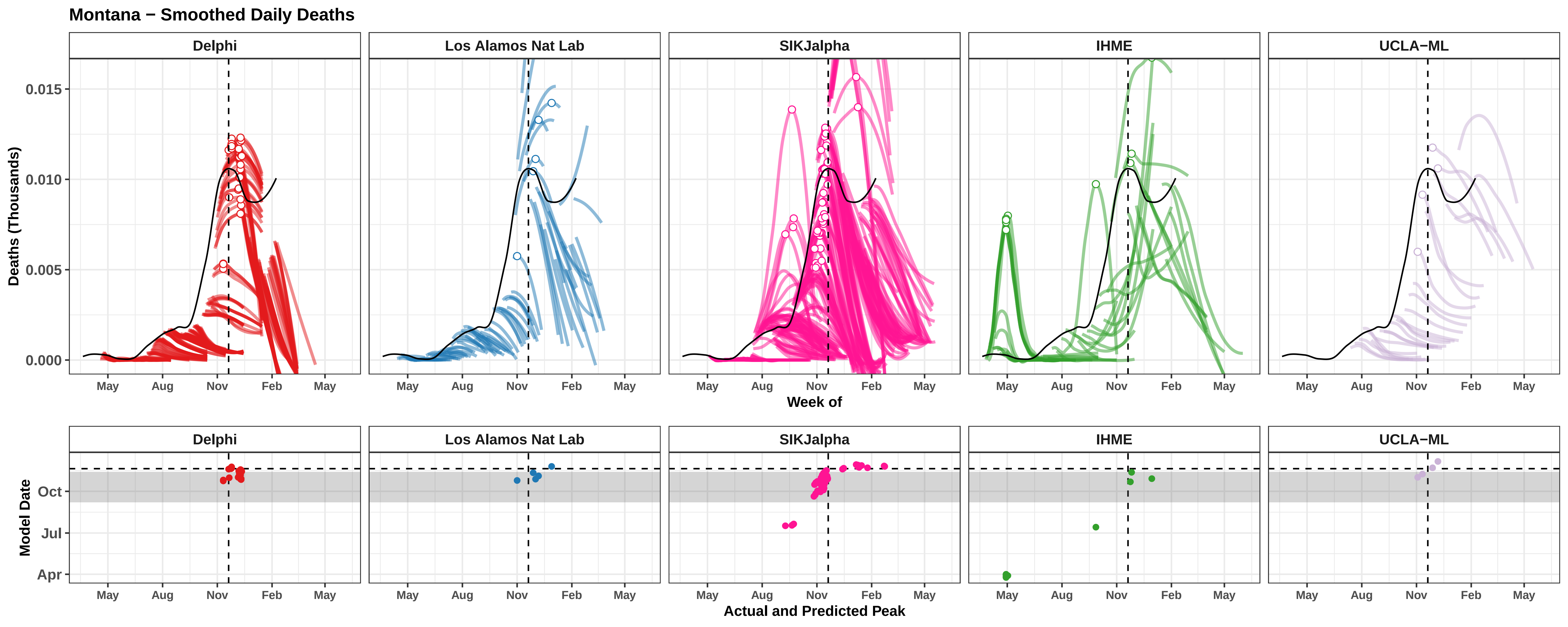

Montana − Smoothed Daily Deaths
Delphi
Los Alamos Nat Lab
SIKJalpha
IHME
UCLA−ML
Deaths (Thousands)
0.000
0.005
0.010
0.015
May
Aug
Nov
Feb
May
Week of
Model Date
Apr
Jul
Oct
Actual and Predicted Peak

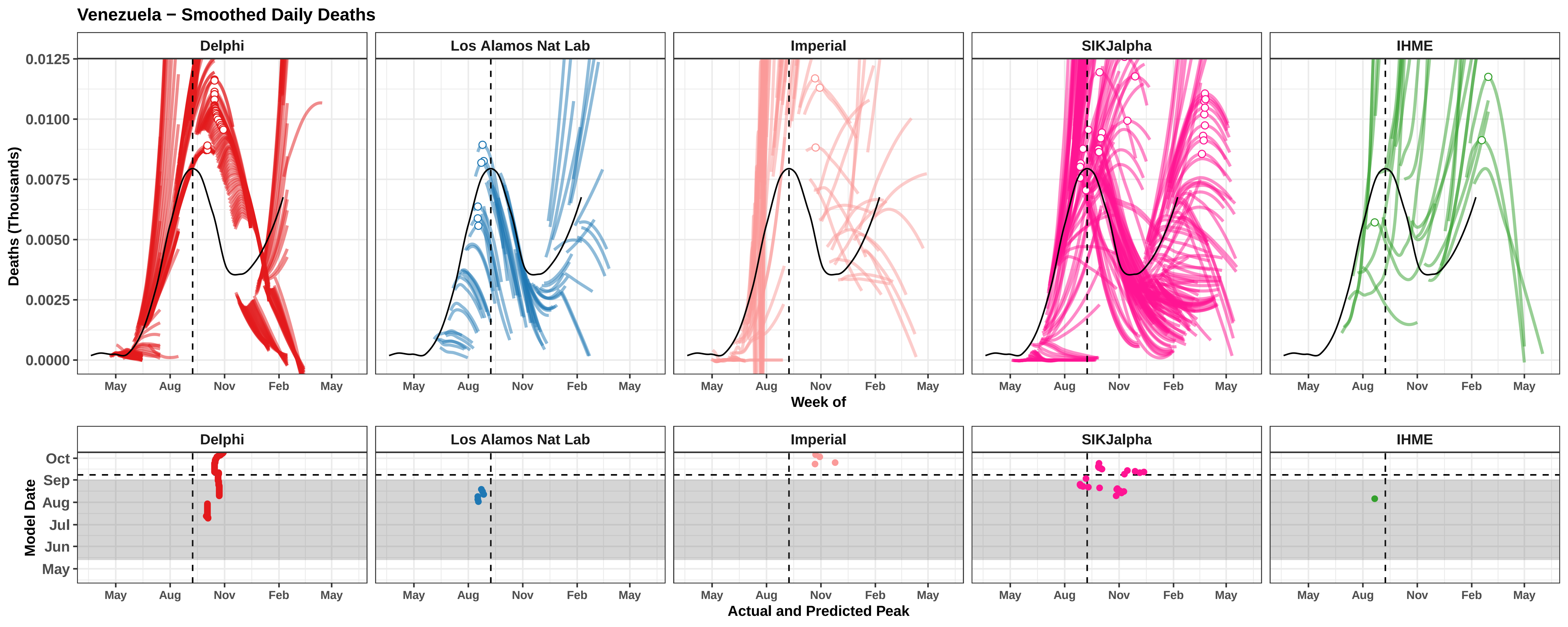
Venezuela − Smoothed Daily Deaths
Delphi
Los Alamos Nat Lab
Imperial
SIKJalpha
IHME
Deaths (Thousands)
0.0125
0.0100
0.0075
0.0050
0.0025
0.0000
May
Aug
Nov
Feb
May
Week of
Model Date
Oct
Sep
Aug
Jul
Jun
May
Actual and Predicted Peak

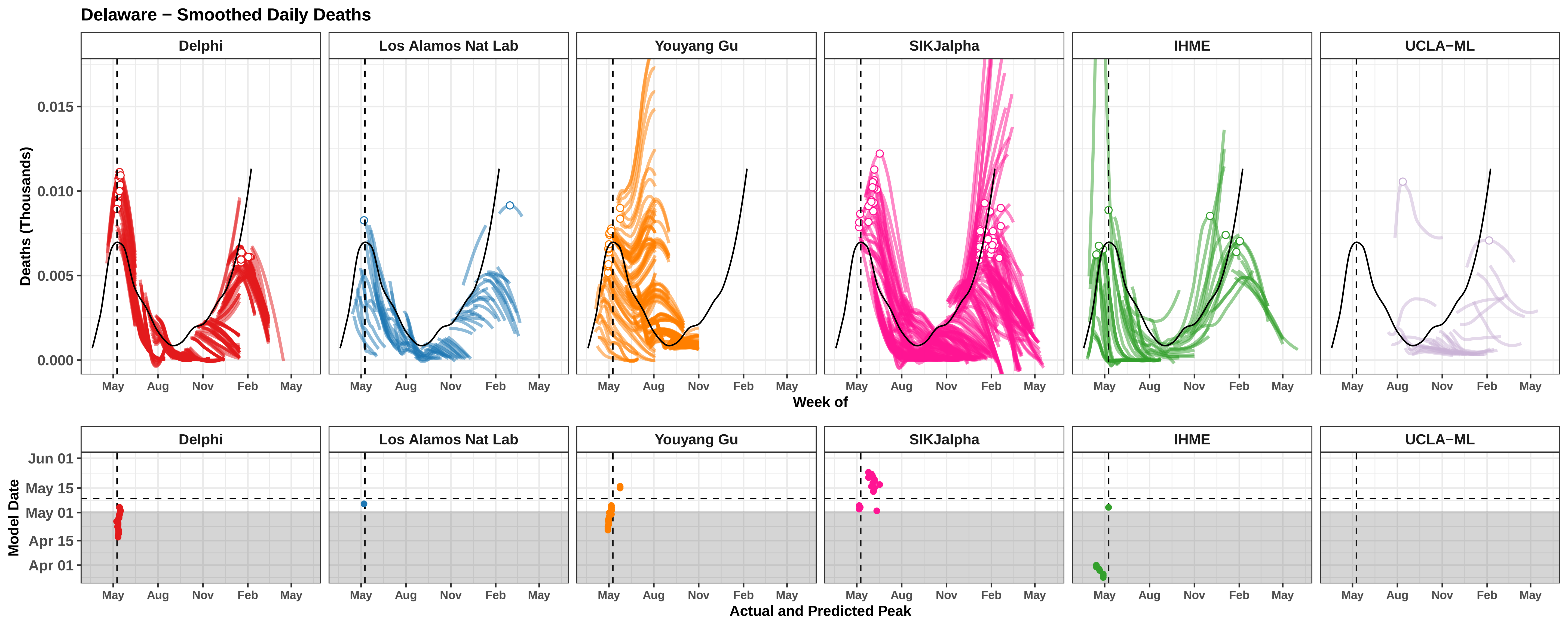

Delaware − Smoothed Daily Deaths
Delphi
Los Alamos Nat Lab
Youyang Gu
SIKJalpha
IHME
UCLA−ML
0.015
0.010
0.005
0.000
Deaths (Thousands)
May
Aug
Nov
Feb
May
Week of
Jun 01
May 15
May 01
Apr 15
Apr 01
Model Date
Actual and Predicted Peak

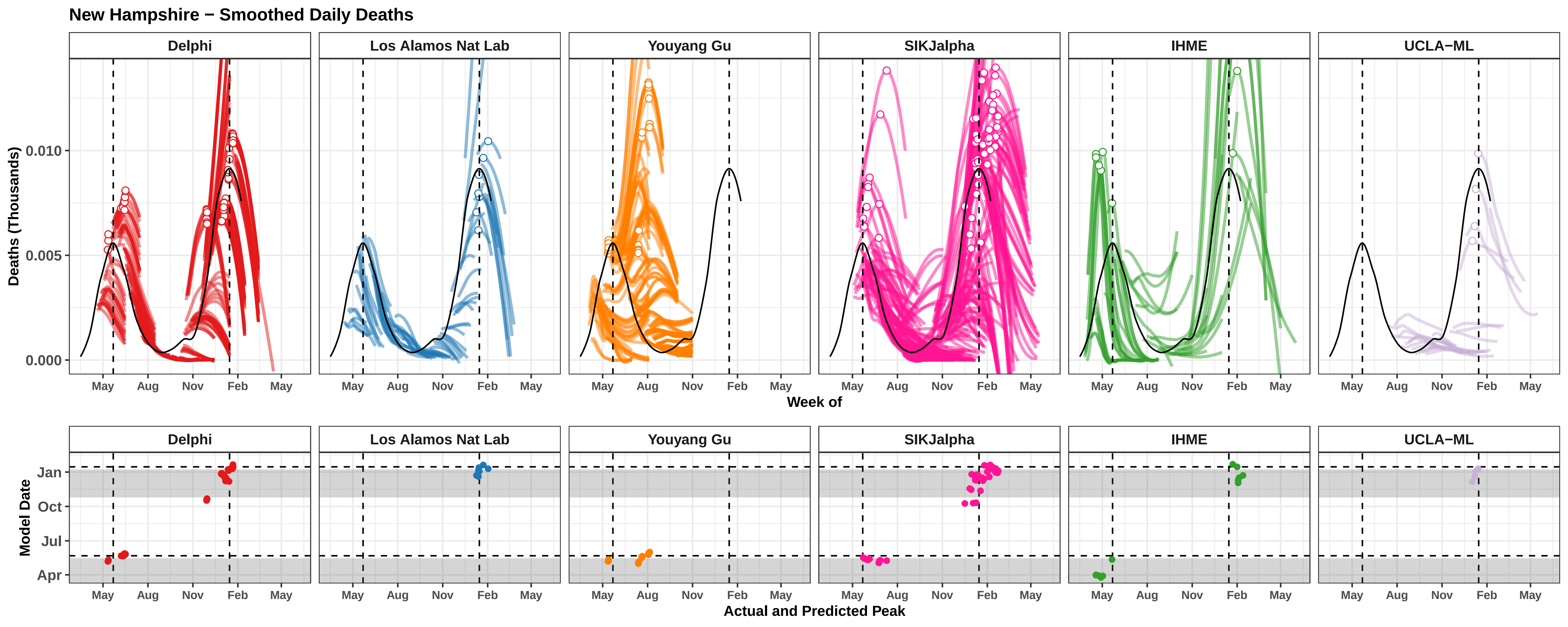
New Hampshire − Smoothed Daily Deaths
Delphi
Los Alamos Nat Lab
Youyang Gu
SIKJalpha
IHME
UCLA−ML
Deaths (Thousands)
0.000
0.005
0.010
May
Aug
Nov
Feb
May
Week of
Model Date
Apr
Jul
Oct
Jan
Actual and Predicted Peak

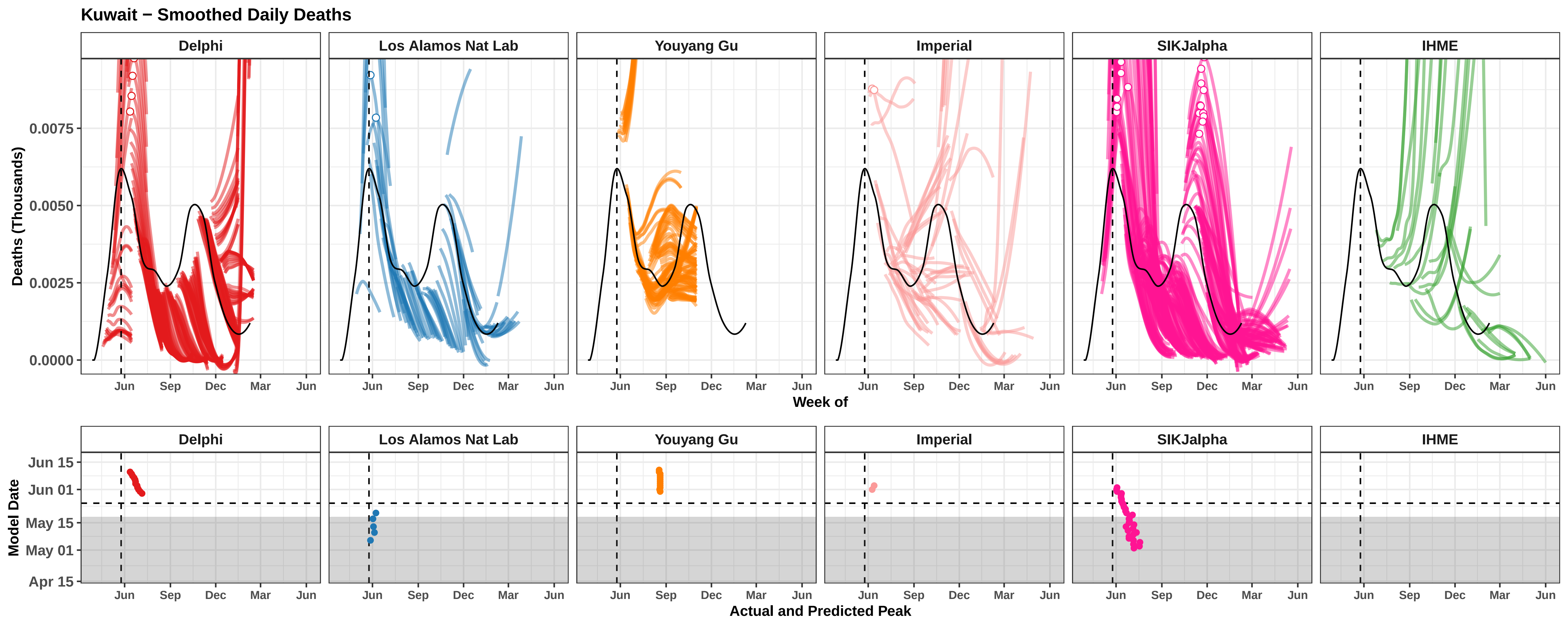
Kuwait − Smoothed Daily Deaths
Delphi
Los Alamos Nat Lab
Youyang Gu
Imperial
SIKJalpha
IHME
Deaths (Thousands)
0.0075
0.0050
0.0025
0.0000
Jun
Sep
Dec
Mar
Week of
Model Date
Jun 15
Jun 01
May 15
May 01
Apr 15
Actual and Predicted Peak

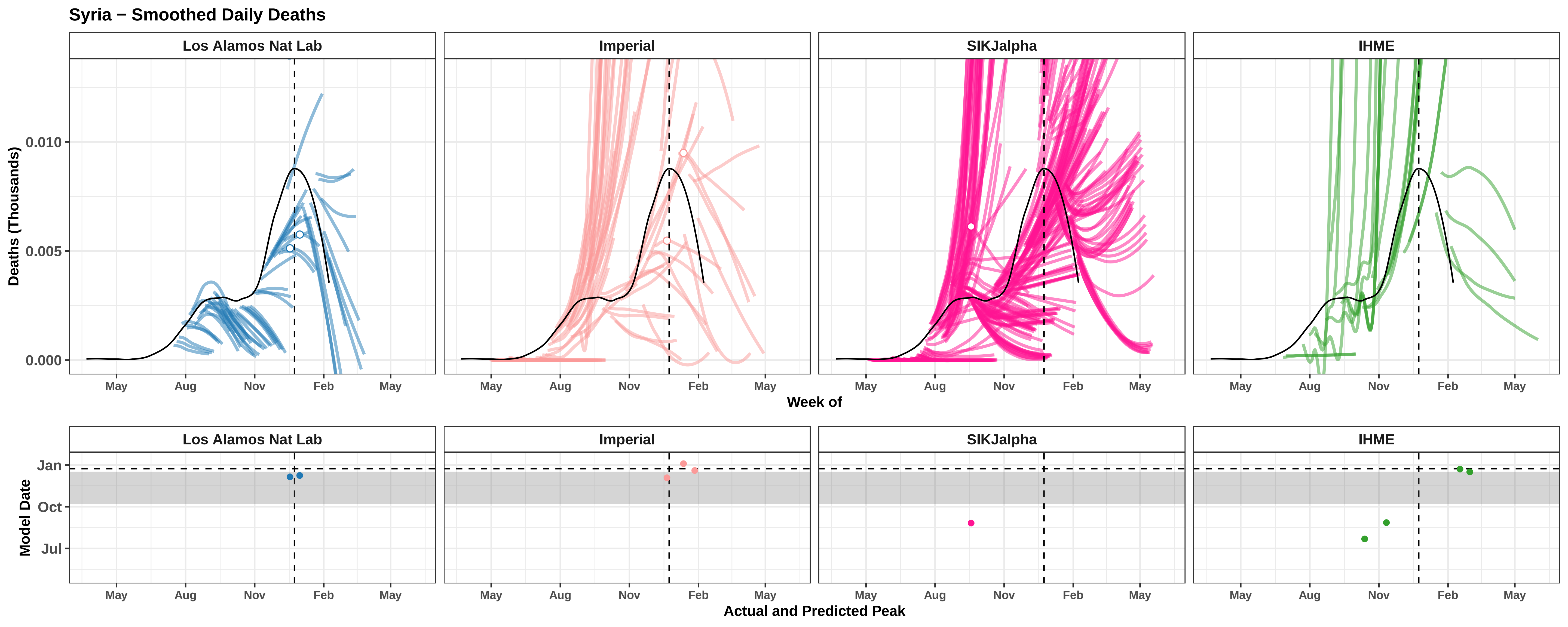

Syria − Smoothed Daily Deaths
Los Alamos Nat Lab
Imperial
SIKJalpha
IHME
Deaths (Thousands)
0.010
0.005
0.000
May
Aug
Nov
Feb
May
Week of
Model Date
Jan
Oct
Jul
Actual and Predicted Peak

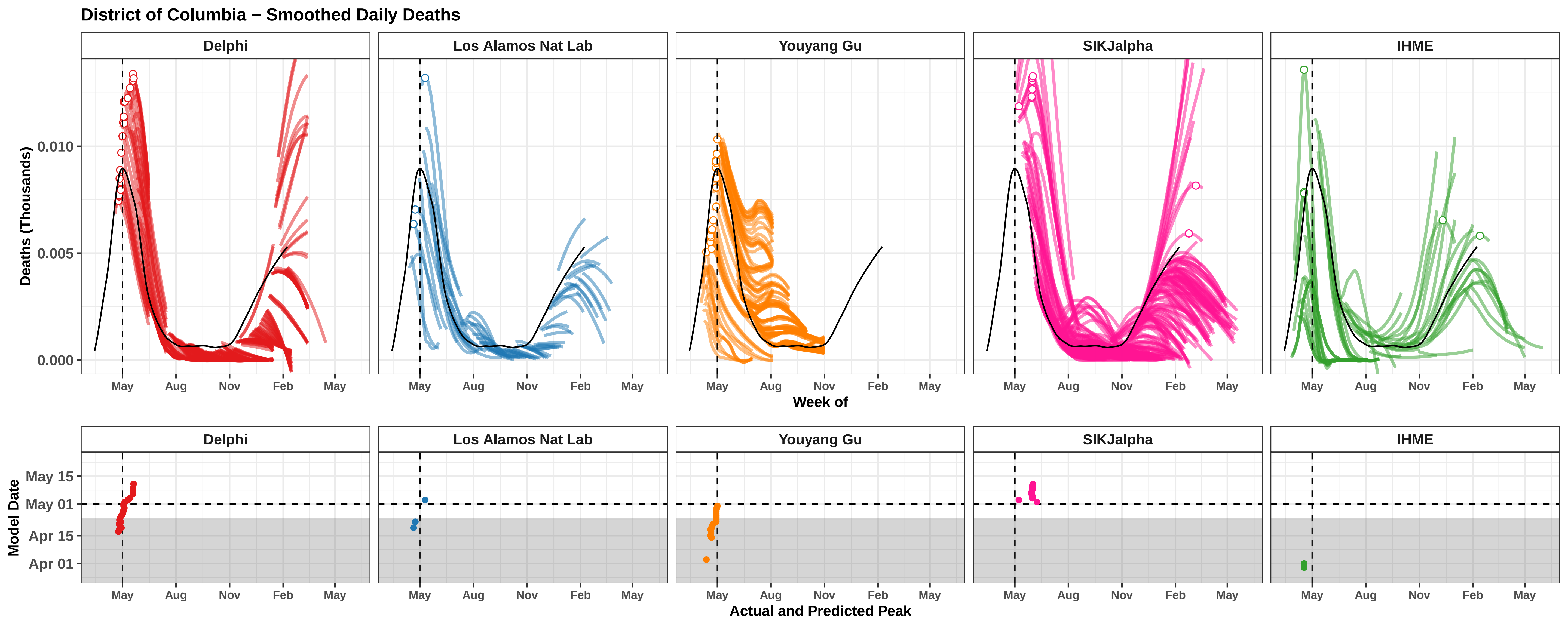

District of Columbia − Smoothed Daily Deaths
Delphi
Los Alamos Nat Lab
Youyang Gu
SIKJalpha
IHME
Deaths (Thousands)
0.010
0.005
0.000
May
Aug
Nov
Feb
May
Week of
Model Date
May 15
May 01
Apr 15
Apr 01
Actual and Predicted Peak

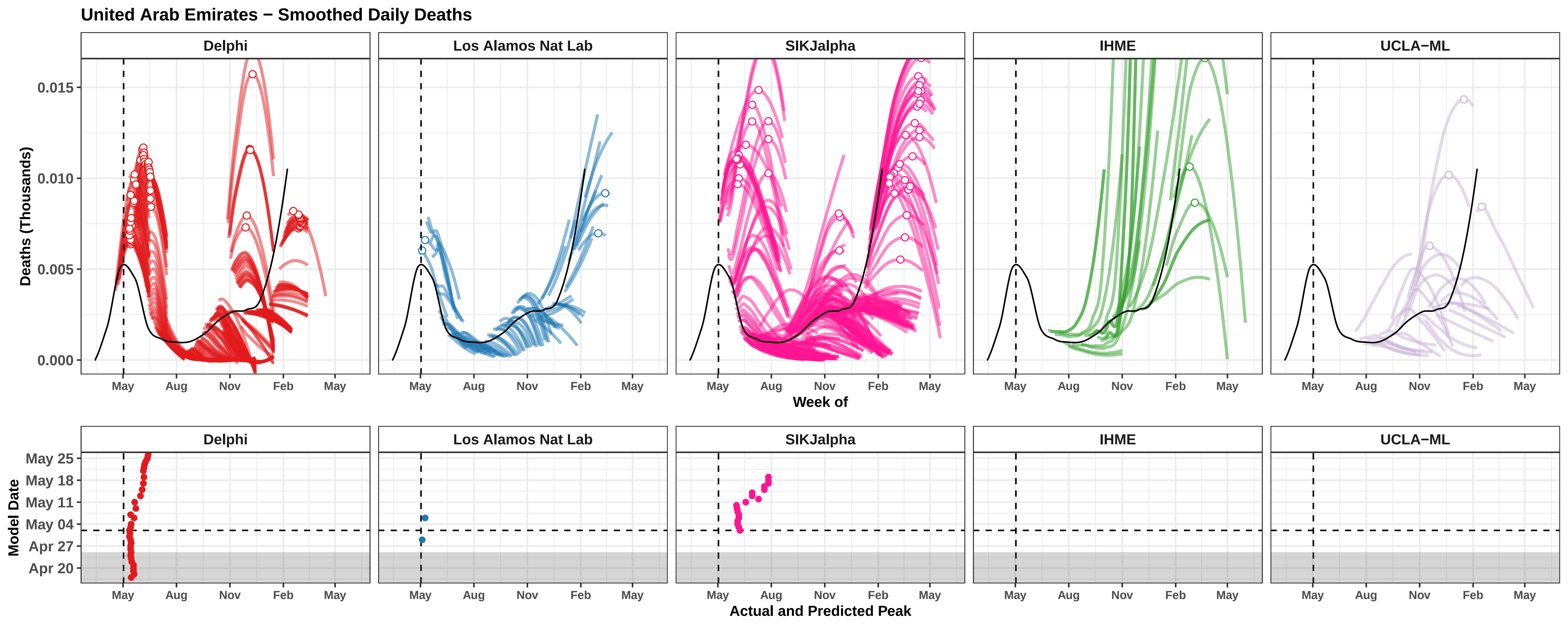

United Arab Emirates − Smoothed Daily Deaths
Delphi
Los Alamos Nat Lab
SIKJalpha
IHME
UCLA−ML
Deaths (Thousands)
0.015
0.010
0.005
0.000
May
Aug
Nov
Feb
May
Week of
Model Date
May 25
May 18
May 11
May 04
Apr 27
Apr 20
Actual and Predicted Peak

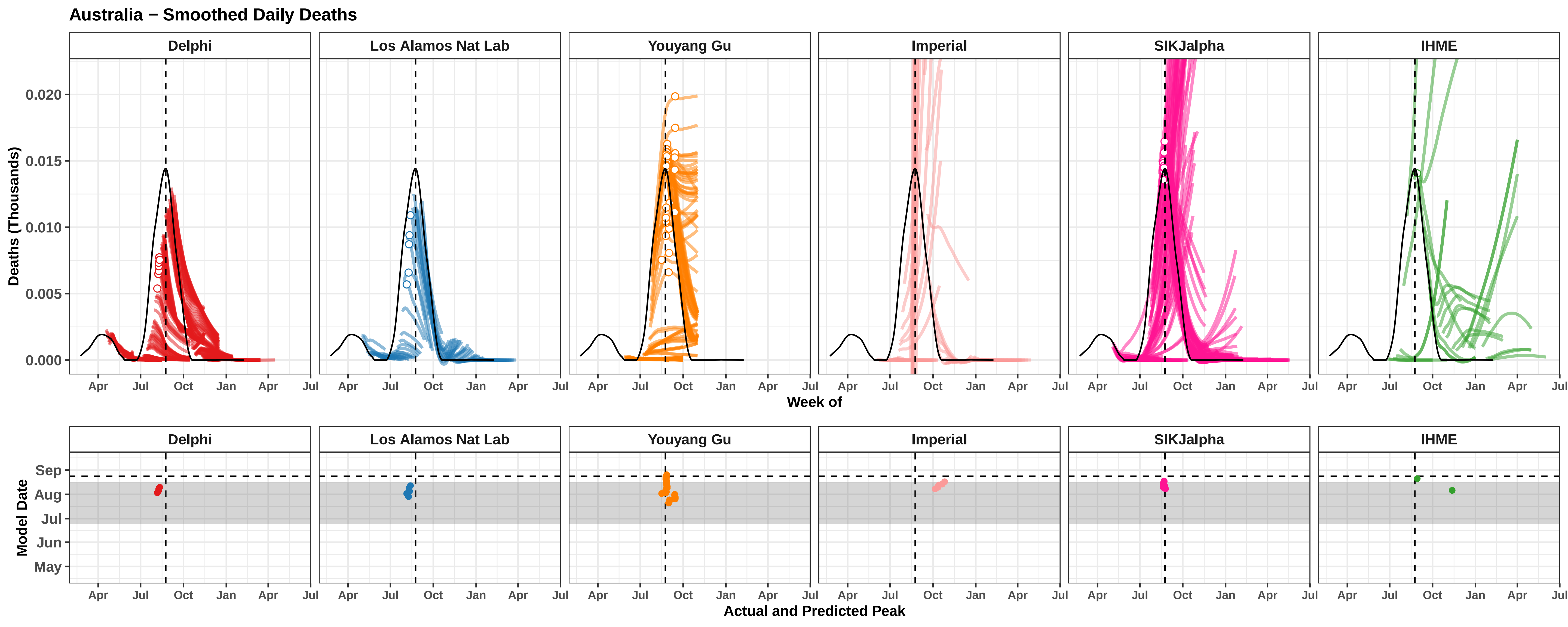
Australia − Smoothed Daily Deaths
Delphi
Los Alamos Nat Lab
Youyang Gu
Imperial
SIKJalpha
IHME
Deaths (Thousands)
0.000
0.005
0.010
0.015
0.020
Apr
Jul
Oct
Jan
Apr
Jul
Week of
Model Date
May
Jun
Jul
Aug
Sep
Actual and Predicted Peak

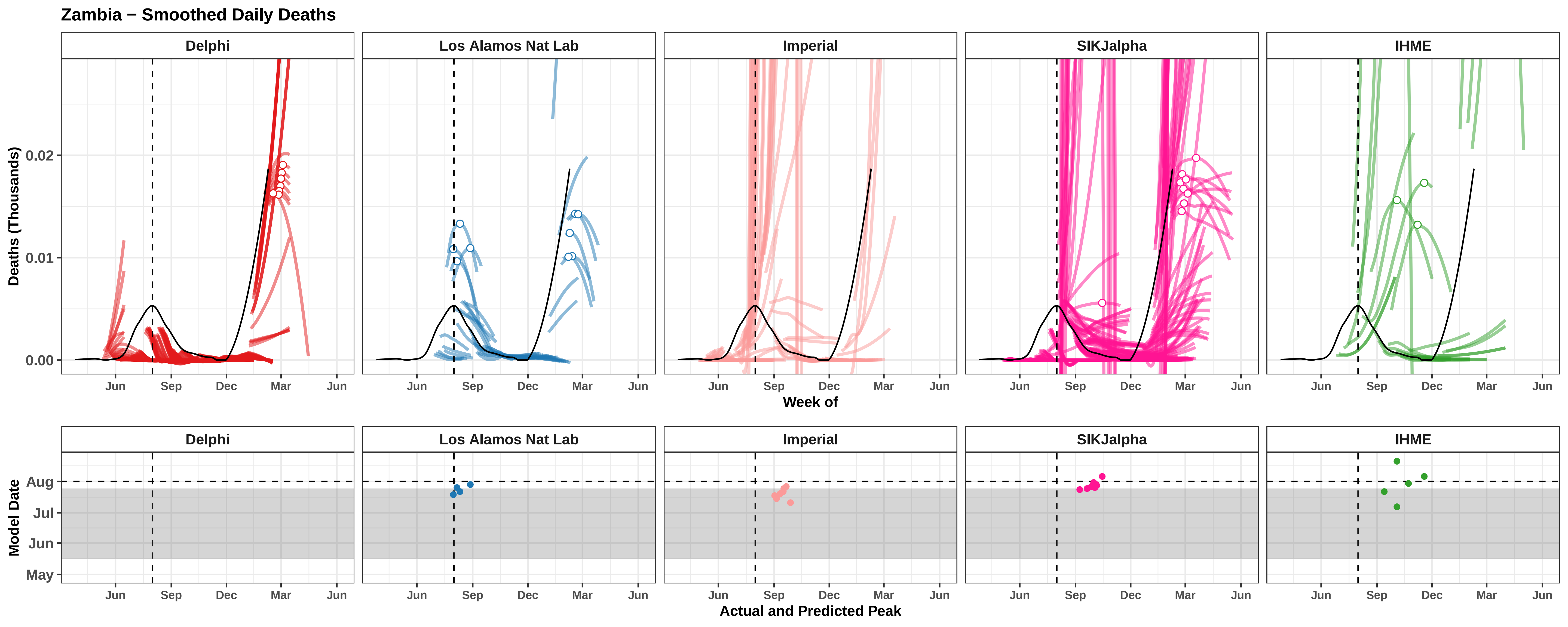
Zambia − Smoothed Daily Deaths
Delphi
Los Alamos Nat Lab
Imperial
SIKJalpha
IHME
Deaths (Thousands)
0.00
0.01
0.02
Jun
Sep
Dec
Mar
Jun
Week of
Model Date
Aug
Jul
Jun
May
Actual and Predicted Peak

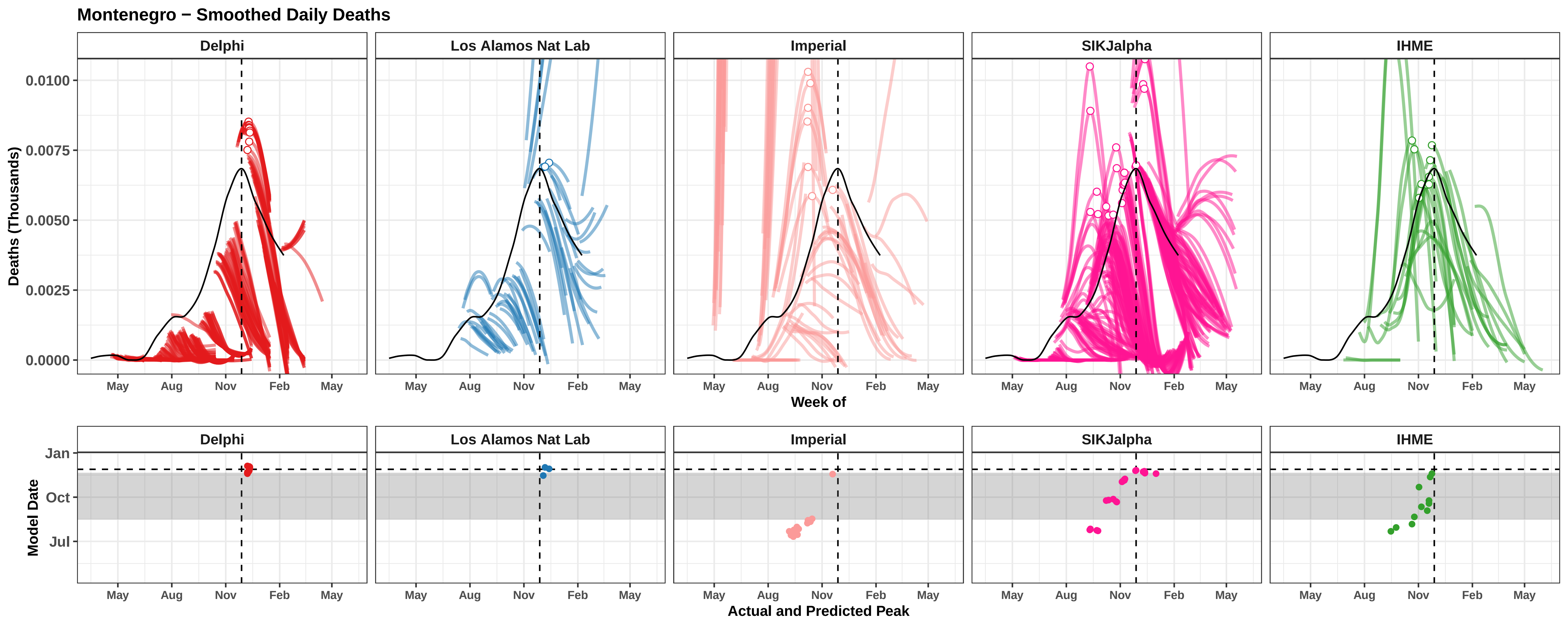

Montenegro − Smoothed Daily Deaths
Delphi
Los Alamos Nat Lab
Imperial
SIKJalpha
IHME
Deaths (Thousands)
0.0100
0.0075
0.0050
0.0025
0.0000
May
Aug
Nov
Feb
May
Week of
Model Date
Jan
Oct
Jul
Actual and Predicted Peak

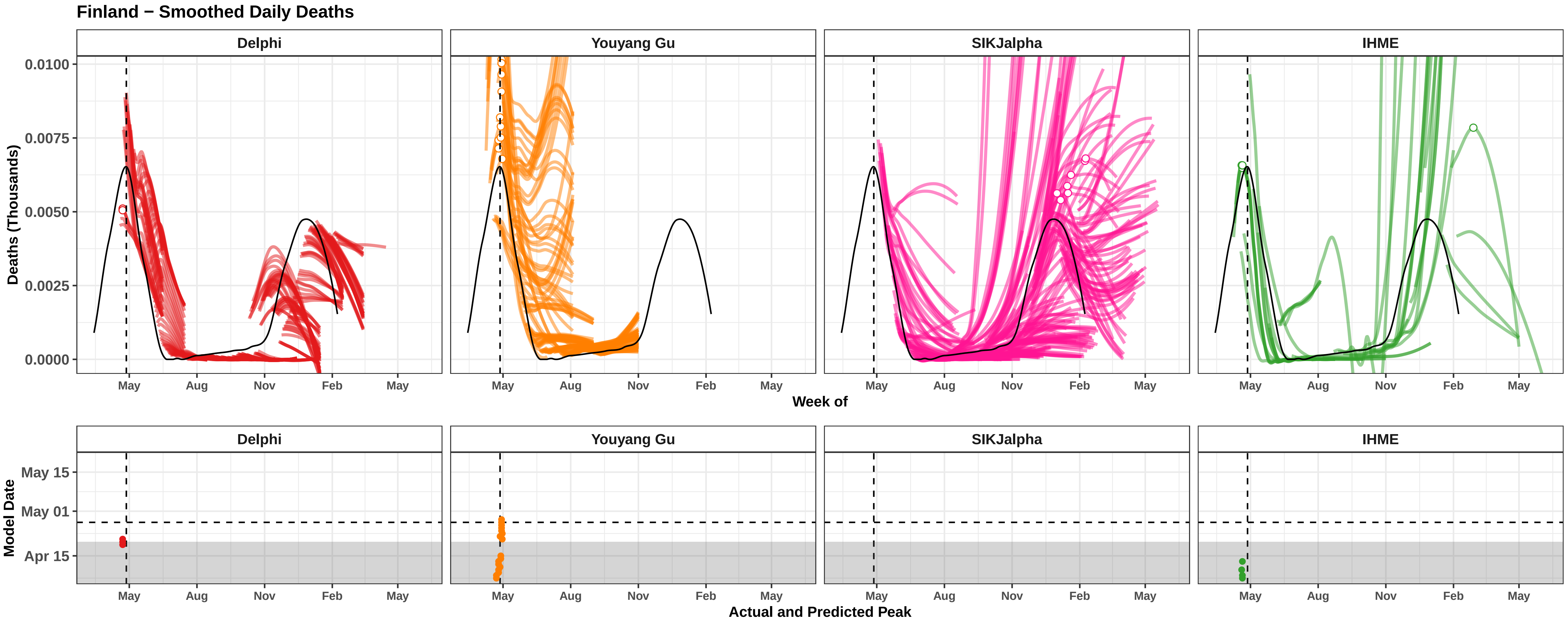

Finland − Smoothed Daily Deaths
Delphi
Youyang Gu
SIKJalpha
IHME
Deaths (Thousands)
0.0100
0.0075
0.0050
0.0025
0.0000
May
Aug
Nov
Feb
May
Week of
Model Date
May 15
May 01
Apr 15
Actual and Predicted Peak

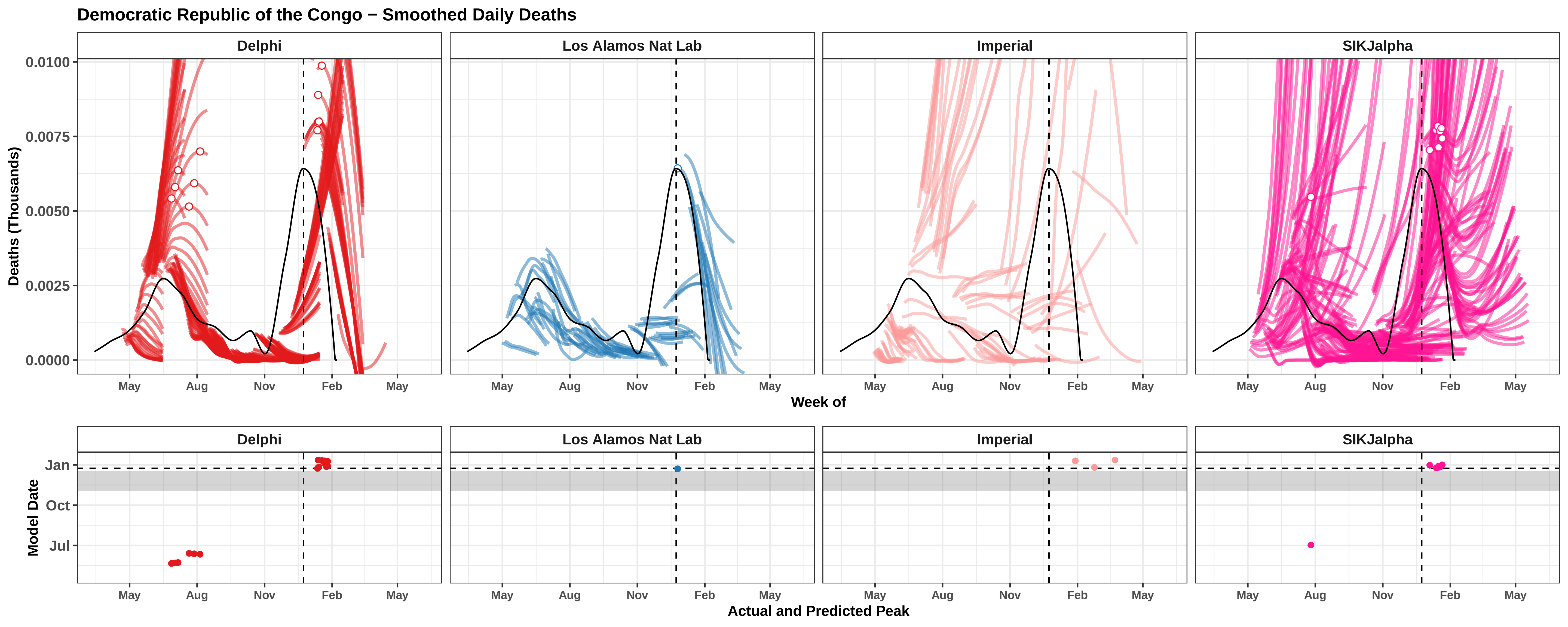

Democratic Republic of the Congo − Smoothed Daily Deaths
Delphi
Los Alamos Nat Lab
Imperial
SIKJalpha
Deaths (Thousands)
0.0100
0.0075
0.0050
0.0025
0.0000
May
Aug
Nov
Feb
May
Week of
Model Date
Jan
Oct
Jul
Actual and Predicted Peak

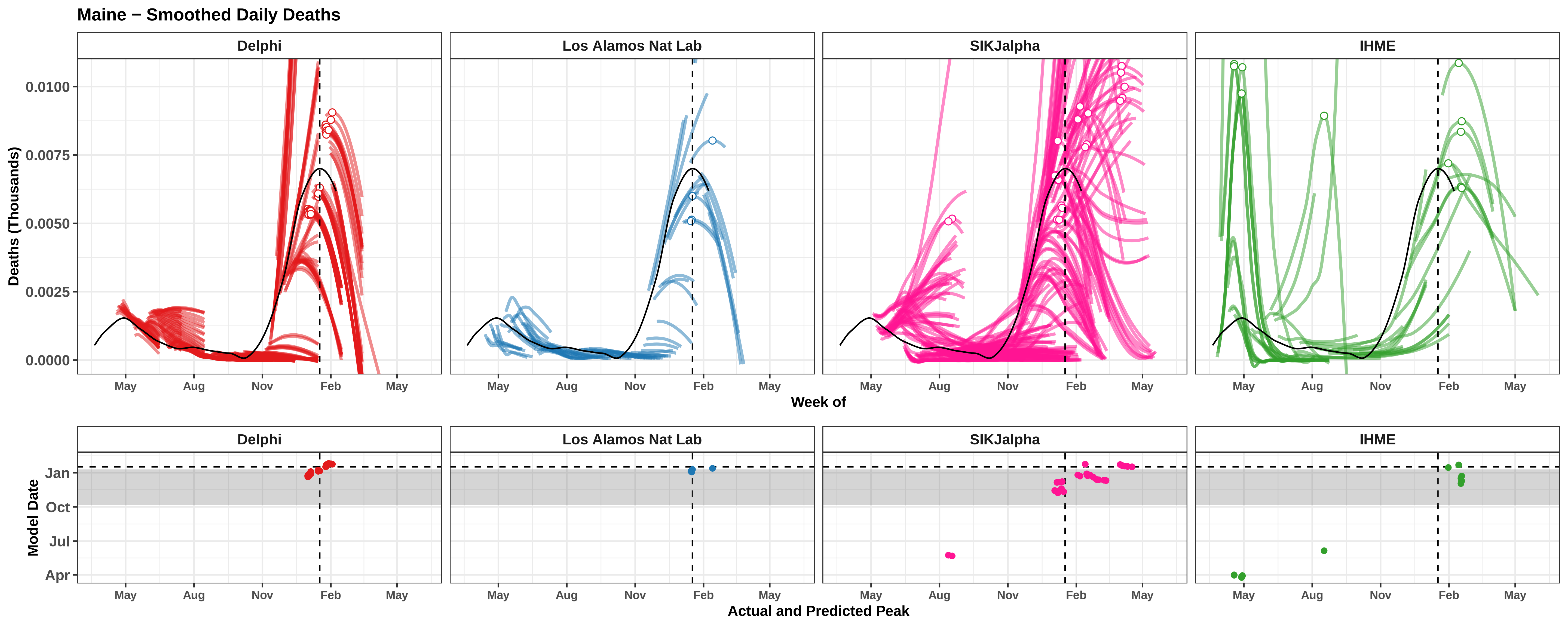

Maine − Smoothed Daily Deaths
Delphi
Los Alamos Nat Lab
SIKJalpha
IHME
Deaths (Thousands)
0.0100
0.0075
0.0050
0.0025
0.0000
May
Aug
Nov
Feb
May
Week of
Model Date
Jan
Oct
Jul
Apr
Actual and Predicted Peak

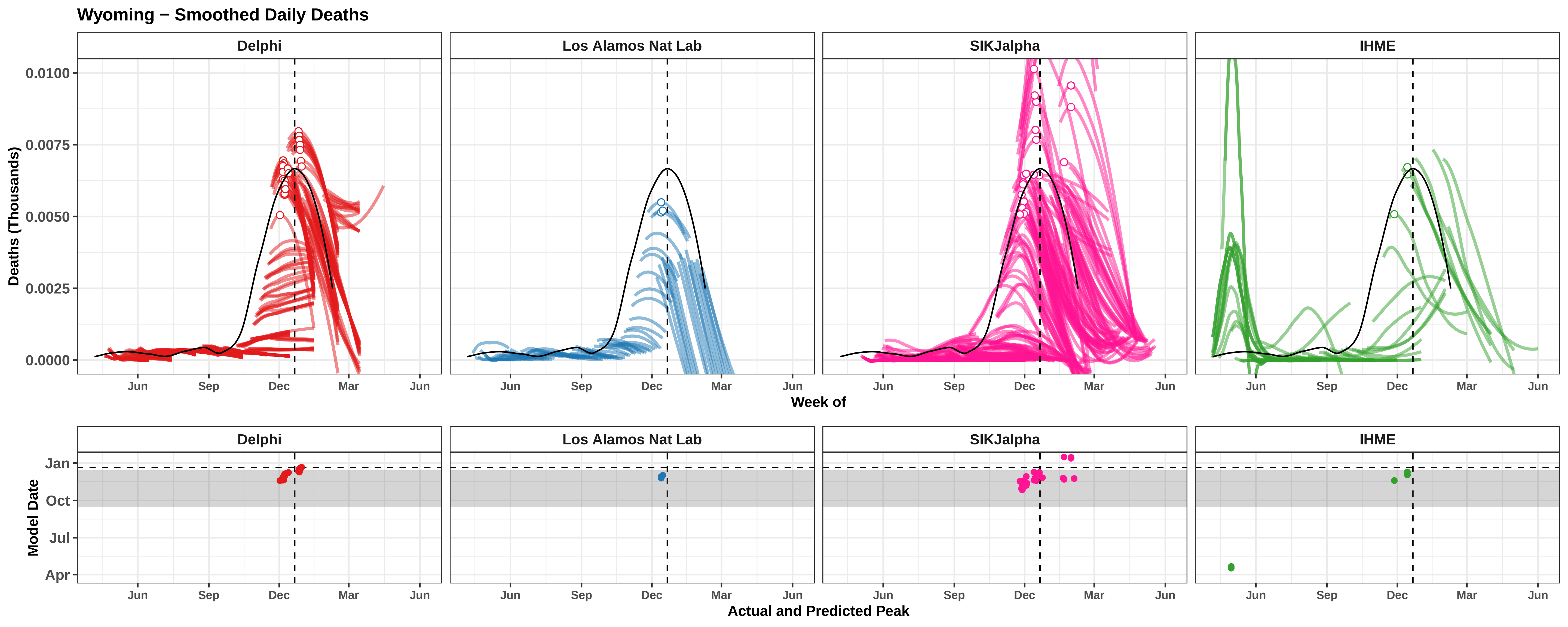
Wyoming − Smoothed Daily Deaths
Delphi
Los Alamos Nat Lab
SIKJalpha
IHME
Deaths (Thousands)
0.0100
0.0075
0.0050
0.0025
0.0000
Jun
Sep
Dec
Mar
Jun
Week of
Model Date
Jan
Oct
Jul
Apr
Actual and Predicted Peak

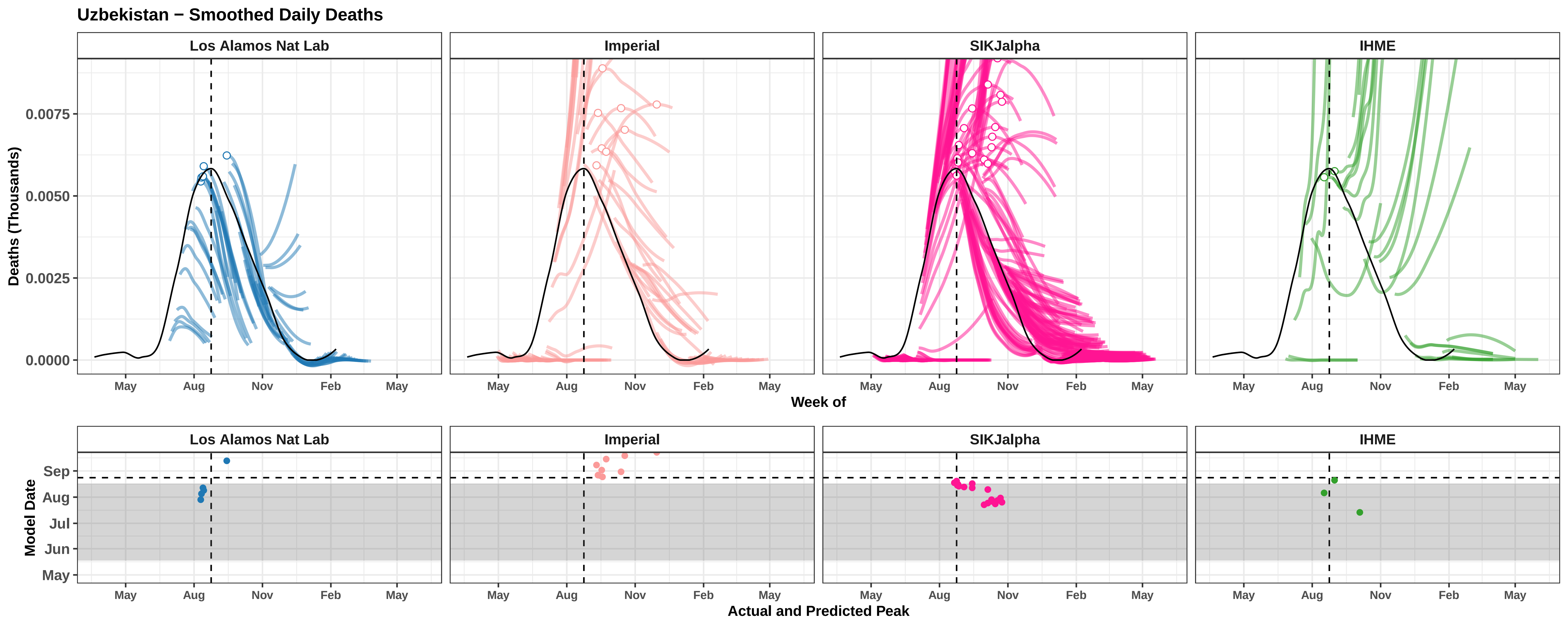
Uzbekistan − Smoothed Daily Deaths
Los Alamos Nat Lab
Imperial
SIKJalpha
IHME
Deaths (Thousands)
0.0075
0.0050
0.0025
0.0000
May
Aug
Nov
Feb
May
Week of
Model Date
Sep
Aug
Jul
Jun
May
Actual and Predicted Peak

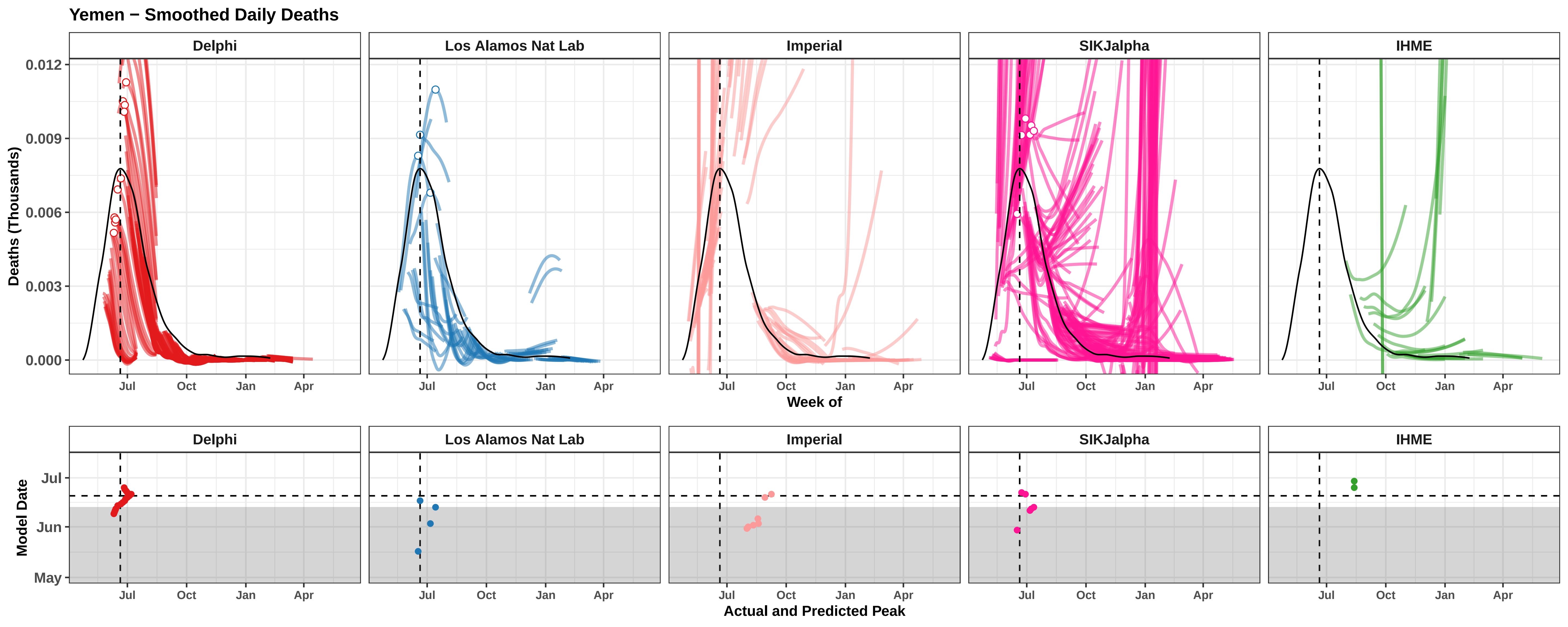

Yemen − Smoothed Daily Deaths
Delphi
Los Alamos Nat Lab
Imperial
SIKJalpha
IHME
Deaths (Thousands)
0.012
0.009
0.006
0.003
0.000
Jul
Oct
Jan
Apr
Week of
Model Date
Jul
Jun
May
Actual and Predicted Peak

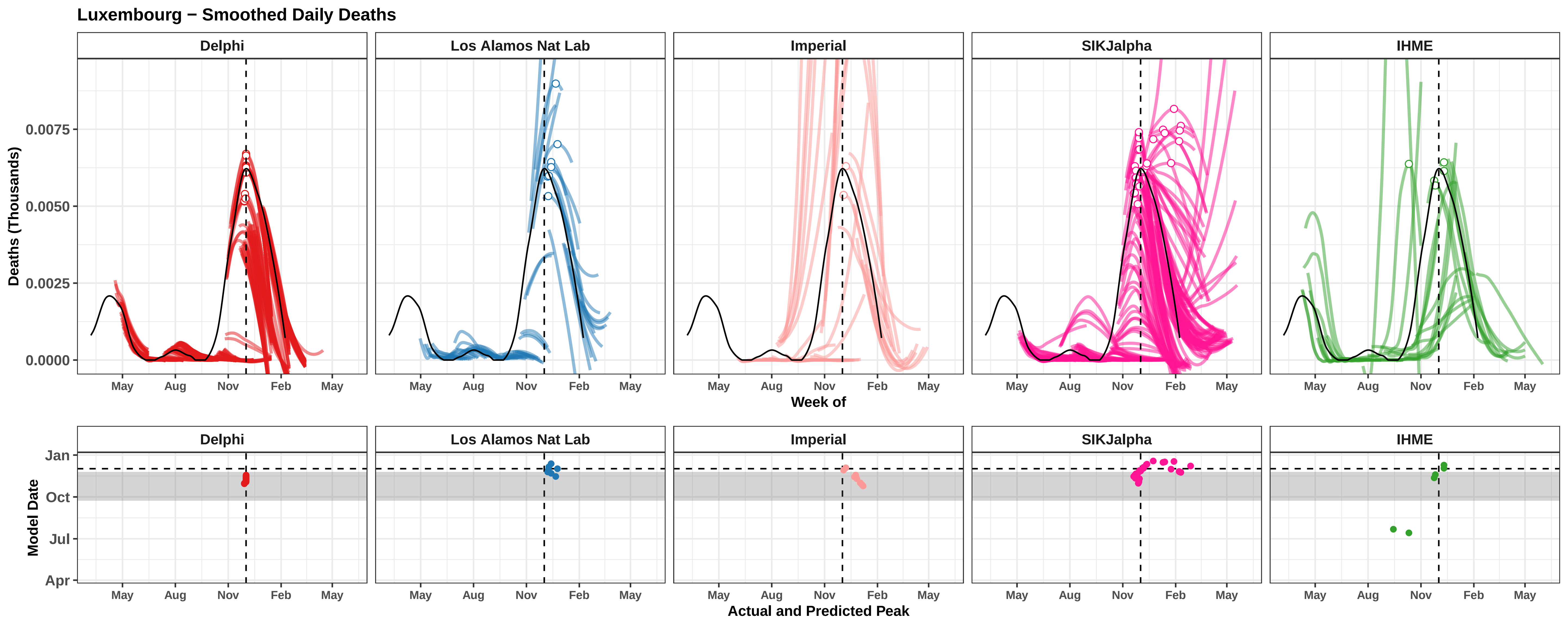

Luxembourg − Smoothed Daily Deaths
Delphi
Los Alamos Nat Lab
Imperial
SIKJalpha
IHME
Deaths (Thousands)
0.0075
0.0050
0.0025
0.0000
May
Aug
Nov
Feb
May
Week of
Model Date
Jan
Oct
Jul
Apr
Actual and Predicted Peak
